# Integrated multi-omics analysis reveals insights into Chinese forest musk deer (*Moschus berezovskii*) genome evolution and musk synthesis

**DOI:** 10.3389/fcell.2023.1156138

**Published:** 2023-05-09

**Authors:** Hui Feng, Tingyin Feng, Yidi Mo, Suli Sun, Lu Wang, Chunbin Lu, Chengli Feng, Ke Xing, Zhijian Su

**Affiliations:** ^1^ Shaanxi Institute of Zoology, Xi’an, Shaanxi, China; ^2^ School of Life Sciences, Guangzhou University, Guangzhou, China; ^3^ Department of Cell Biology, Jinan University, Guangzhou, China; ^4^ Department of Developmental Biology and Regenerative Medicine, Jinan University, Guangzhou, China; ^5^ National Engineering Research Center of Genetic Medicine, Jinan University, Guangzhou, China

**Keywords:** *Moschus berezovskii*, musk gland, integrated analysis, single cell sequencing, cell composition

## Abstract

Among the artiodactyls, male animals belonging to the Family Moschidae have a unique tissue, the musk gland, with the capability of musk synthesis. However, the genetic basis of musk gland formation and musk production are still poorly understood. Here, musk gland tissues from two juvenile and three adult Chinese forest musk deer (*Moschus berezovskii*) were utilized to analyze genomic evolution events, evaluate mRNA profiles and investigate cell compositions. By performing genome reannotation and comparison with 11 ruminant genomes, three expanded gene families were identified in the *Moschus berezovskii* genome. Transcriptional analysis further indicated that the musk gland displayed a prostate-like mRNA expression pattern. Single-cell sequencing revealed that the musk gland is composed of seven distinguishable cell types. Among them, sebaceous gland cells and luminal epithelial cells play important roles in musk synthesis, while endothelial cells master the regulation of cell-to-cell communication. In conclusion, our study provides insights into musk gland formation and the musk-synthesizing process.

## 1 Introduction

The Chinese forest musk deer, also called the dwarf musk deer, belongs to the Order Artiodactyla, Suborder Ruminantia*,* Family Moschidae and Genus *Moschus*. Comparedwith the animals of the same Order, the males of this species have a unique musk gland that produces musk for the purpose of attracting females during the breeding season (Yang et al., 2003). In general, the formation of musk undergoes two stages: secretion from the columnar epithelial cells of the musk gland and then fermentation by the microbiota in the musk gland to become a solid substance with strong fragrance (Li et al., 2018). The component analysis indicated that musk contains macrocyclic ketones, pyridine derivatives, steroids, proteins, fatty acids and ester compounds (Li et al., 2016). Pharmacological studies have further demonstrated that musk, especially the main active ingredient muscone, has immune regulatory, neuroprotective, cardioprotective and antioxidative activities (Liu et al., 2021). Therefore, from ancient times to the present day, musk has always been a precious raw material and is widely used in traditional medicine. In China alone, more than 884 traditional Chinese medicine (TCM) prescriptions use musk as a major ingredient, and the capacity requirement planning is nearly 1,500 kg per year (Meng et al., 2012; Liu et al., 2021).

To demonstrate the origin of the musk gland and the synthesis mechanism of musk, many studies focused on the molecular characteristics of the genome, transcriptomic comparisons of different tissues and microbiota changes during musk formation have been performed in depth. The genome of Chinese forest musk deer (*Moschus berezovskii*) and wild Siberian musk deer (*Moschus moschiferus*) is approximately 2.72 Gb and 3.10 Gb, respectively. The phylogenetic analysis indicated that these musk deer are closer to Bovidae than to Cervidae (Fan Z. et al., 2018; Zhou et al., 2019; Yi et al., 2020). Moreover, most potential musk-secreting regulatory genes screened by bioinformatics were related to steroidogenesis and enriched in the pathways of steroid hormone metabolism (Xu et al., 2017; Fan Z. et al., 2018; Zhou et al., 2019; Yi et al., 2020; Jie et al., 2021; Yang et al., 2021). The subsequent investigation also suggested that sex steroids may play a role in promoting musk synthesis (Fan M. et al., 2018). To date, the underlying mechanism of musk synthesis remains largely unknown.

In the present study, we compared two musk deer genomes with nine other Ruminantia species and compared the mRNA profiles of the musk gland between juvenile and adult Chinese forest musk deer based on bulk transcriptome and single-cell sequencing. Through an integrated analysis, we aimed to identify 1) the molecular characteristics of the musk deer genome during the evolutionary origination of the musk gland; 2) the genetic signatures of the musk gland and the difference in mRNA profiles between the juvenile and adult phases; and 3) the cell composition, potential function, and cell-to-cell interaction of the musk gland.

## 2 Materials and methods

### 2.1 Animals, criteria and ethics approval statement

The Chinese forest musk deer for this experiment were housed and raised on a farm of captive forest musk in Hekou town, Feng County, Baoji city, Shaanxi Province. The animals were individually housed in the animal facilities of the farm with free access to food and water. During the musk-secreting period (April to June 2021), three adult males (6–8 years old) and two underage males (8–10 months) were selected for the experiment. The selection criteria for adult males included the following: 1) they were able to produce musk the previous year, and 2) they had appeared anorexic for 1–2 days. This study was carried out in accordance with the recommendations of the Institution of Animal Care and the Ethics Committee of Shaanxi Institute of Zoology (Northwest Institute of Endangered Zoological Species). The collection of samples and experimental protocols were approved by the Animal Ethics Committee of Shaanxi Institute of Zoology.

### 2.2 Sample collection and bulk RNA sequencing

The Chinese forest musk deer were anesthetized using the commercial anesthetic Lu-Mian-Ning containing 100 mg/mL xylazine hydrochloride (Huamu animal health product company, Jilin, China). A surgical construction of stoma below the musk gland mouth was made and approximated 50 mg gland was collected. Meanwhile, approximated 100 mg muscle tissue was isolated from the back of thigh. These tissues were rinsed with phosphate buffered saline (PBS) solution, put into 10 mL tissue storage solution and stored at 4°C immediately. After the operation, the wounds were disinfected, sutured and treated with the anti-inflammatory drugs.

The tissues were transported back to the laboratory and the mRNA was isolated by TRIzol (Qiagen, Hilden, the Netherlands) and a MicroPoly (A) Purist kit (Thermo Scientific, Shanghai, China) following the manufacturer’s instructions. RNA-seq libraries with an insert size of 250-bp were prepared using the Illumina standard RNA-seq library preparation pipeline and sequenced on the Illumina HiSeq 2500 platform, with 150-bp paired-end reads generated.

### 2.3 Genomic data analysis

The genomic sequences and annotation of Chinese forest musk deer were downloaded from the Ruminant Genome Project (RGP) v 2.0 database (Fu et al., 2022). Other reference genomic sequences and annotations were downloaded from EMSEMBL Release 106 (https://asia.ensembl.org/index.html) and the NCBI database (http://www.ncbi.nlm.nih.gov). Gene prediction was performed by the BRAKER2 v2.1.5 pipeline with default parameters by integrating *ab initio* gene prediction, RNA-seq-based prediction, and predictions based on protein sequences of *Bos taurus* and *Ovis aries* (Hoff et al., 2019). The completeness of the prediction was then evaluated using BUSCO v5.2.2 (Manni et al., 2021). Gene functions were subsequently analyzed by searching against public databases, including NCBI’s nonredundant (NR), Eukaryotic Orthologous Groups of proteins (KOG), Gene Ontology (GO), Kyoto Encyclopedia of Genes and Genomes (KEGG) and UniProtKB/TrEMBL. The conserved motifs and functional domains of each gene model were predicted by InterProScan version (5.59–91.0) against all available databases (Jones et al., 2014). For comparative genomics analysis, gene families of orthologs across all 11 ruminants were first identified using OrthoFinder v2.4.1 based on precomputed BLAST results with default settings (Emms and Kelly, 2019). The expansion and contraction of the gene families were subsequently quantified using CAFÉ v4.2.1 based on the inferred phylogenomic history (De Bie et al., 2006).

### 2.4 Bulk RNA-sequencing data analysis

The raw data obtained from sequencing were subjected to quality examination by using FastQC v0.11.9 (Andrews, 2023), and Trimmomatic v0.40 (Bolger et al., 2014) was used to remove reads with low-quality scores (<20), containing adapters, and/or containing more than 5% N bases. To quantify the gene expression, the remaining clean reads were mapped to the reference genome by HISAT2 v2.2.1 (Kim et al., 2019), and Stringtie v2.2.0 (Pertea et al., 2015) was employed for quantitative analysis. We performed batch effect correction by ComBat-seq (Zhang et al., 2020), which uses a negative binomial regression model that retains the integer nature of count data. The expression difference analysis was then performed in the R package DEseq2 with a model based on the negative binomial distribution. Differentially expressed genes (DEGs) were selected according to the following criteria: |log2-fold change| >2 and FDR <0.05 (Love et al., 2014). Overlap analysis for different DEG sets was performed by the R package UpSetR v1.4.0 (Conway et al., 2017). Functional enrichment analysis of DEGs against the Gene Ontology and KEGG databases was performed with the R package clusterProfiler v4.0 (Wu et al., 2021). Tissue expression profiles of *Bos taurus* were downloaded from the NCBI database with GEO accession number GSE128075 (Fang et al., 2020). Three musk gland samples of adult musk deer were sequenced and used as biological replicates. After normalization, the expression similarities between musk gland and *Bos taurus* tissue were measured by the Spearman correlation between each pair of samples.

### 2.5 Preparation of muscle and musk gland cell suspensions

To eliminate possible contamination from blood cells, freshly isolated muscle and musk gland were cultured with DMEM/F-12 medium containing 2.2 g/L HEPES, 0.1% BSA, and 0.7 g/L sodium bicarbonate, pH 7.4. The tissues were minced into 0.2–0.5 cm pieces with scissors and then digested with 1 mg/mL collagenase IV (Sigma-Aldrich, Shanghai, China) in DMEM/F-12 medium at 34°C for 20–30 min with slow shaking. After allowing the undigested tissue to settle, the dispersed cells were filtered through a 30 μm pore nylon mesh. Cell viability, assayed by 0.4% Trypan blue staining, was above 90% for cells from controls.

### 2.6 Single-cell RNA-Sequencing (scRNA-Seq)

10× Genomics single-cell RNA-seq (scRNA-seq), involving analysis of transcriptomes on a cell-by-cell basis through the use of microfluidic partitioning to capture single cells and prepare next-generation sequencing cDNA libraries, was performed by the GENE DENOVO company (Guangzhou, China). Briefly, cellular suspensions were loaded on a 10× Genomics GemCode Single-cell instrument to generate single-cell Gel Bead-In-EMlusion (GEMs), and sequencing libraries were constructed with Chromium Next GEM Single Cell 3’ Reagent Kits v3.1. The resultant libraries were sequenced using a 2 × 150 paired-end sequencing protocol on an Illumina NovaSeq 6000 platform.

### 2.7 scRNA-Seq data analysis

The single-cell sequencing data were analyzed by Cell Ranger v3.0.2 (Ewels et al., 2020) to generate the feature-barcode expression matrix. The feature-barcode expression matrices were subsequently exported to the R package Seurat v6.0.2 for downstream analysis (Hao et al., 2021). For quality control, cells with an unusually high or low number of unique molecular identifiers (UMIs) (UMIs >5,000 or UMIs <200) or with more than 10% mitochondrial genes were filtered out.

Data from adults and juveniles were integrated by a regularized negative binomial regression approach, Sctransform, based on 2,000 highly variable genes (Choudhary and Satija, 2022). Unsupervised cell clustering was performed by an algorithm based on shared nearest neighbor (SNN) graphs and modular optimization with a resolution of 0.5. It first calculates k-nearest neighbors and constructs SNN graphs and then optimizes the modularity function to identify specific class clusters. For each cluster, the marker genes were identified by the FindAllMarkers function with the Wilcox rank sum test with criteria |log2FoldChange| >0.25 and adjusted *p-value* < 0.05, and clusters with similar profiles were subsequently merged for further analysis. The annotation of cell type was performed by literature-reported markers and the Cellkb database (https://www.cellkb.com/). The dimension reduction and visualization of profiles was carried out using uniform manifold approximation and projection (UMAP) (Becht et al., 2019).

To assess the performance of the gene set corresponding to different samples and cell types, the R package scGSVA was downloaded from GitHub (https://github.com/guokai8/scGSVA). The pathway scores of each single cell were calculated, and a nonparametric unsupervised analysis method was applied to evaluate the results of gene set enrichment (Hänzelmann et al., 2013).

Cell-to-cell communication analysis was performed by NATAMI (https://github.com/asrhou/NATMI) (Hou et al., 2020). First, the normalized expression value of each individual cell was used as input data. Then, the ExtractEdges function was used to calculate the expression and specificity of ligand–receptor interactions. Finally, the predicted ligand-receptor interactions were loaded into R, and only interactions with both ligand and receptor detection rates >0.1 were included in further analysis.

### 2.8 Quantitative real-time polymerase chain reaction (RT-PCR)

The primers used in the current study are listed in Supplementary Table S1. For mRNA RT-PCR, the total RNAs from the cells and tissues were extracted and used as templates for cDNA synthesis. The reverse transcriptase reactions contained 400 ng of total RNA, 4 μL of 5× buffer, 2 μL of 10× nucleic acid mix, 2 μL of reverse transcriptase mixture, and nuclease-free water. The cDNA was diluted 1:3, and 2 μL of the diluted template was used per 20 μL of the RT-PCR assay. All PCRs were performed using a Bio-Rad CFX Connect Real-Time system, and the data were collected using Bio-Rad CFX Manager software (version 2.0). The relative expression levels of the targeted mRNAs were normalized against the expression of *Gapdh*. The fold changes in the expression between the treatments and controls were calculated by the 2^−ΔΔCt^ method. The efficiency of RT-PCR performance for target genes was between 100% and 105%. All data were derived from three different independent experiments.

### 2.9 Statistical analyses

Data are expressed as the mean ± standard error of the mean (S.E.M.). For comparisons of two groups, Student’s t*-*test was used values were considered significant at *p* < 0.05.

## 3 Results

### 3.1 Potential markers for musk gland formation based on genome analysis

To better understand the genetic mechanism underlying the origin of the musk gland, we first profiled the transcriptomes of muscles and musk glands from adult Chinese forest musk deer. Then, we reannotated the genome based on the reported genome data (Fan M. et al., 2018). A total of 20,845 protein-coding genes and 198,773 noncoding genes were identified. The completeness of the genome was measured by BUSCO (Benchmarking universal Single-Copy Orthologs) scores above 90.5%, indicating that our annotation was reliable for downstream analysis (Supplementary Table S2).

**FIGURE 1:**
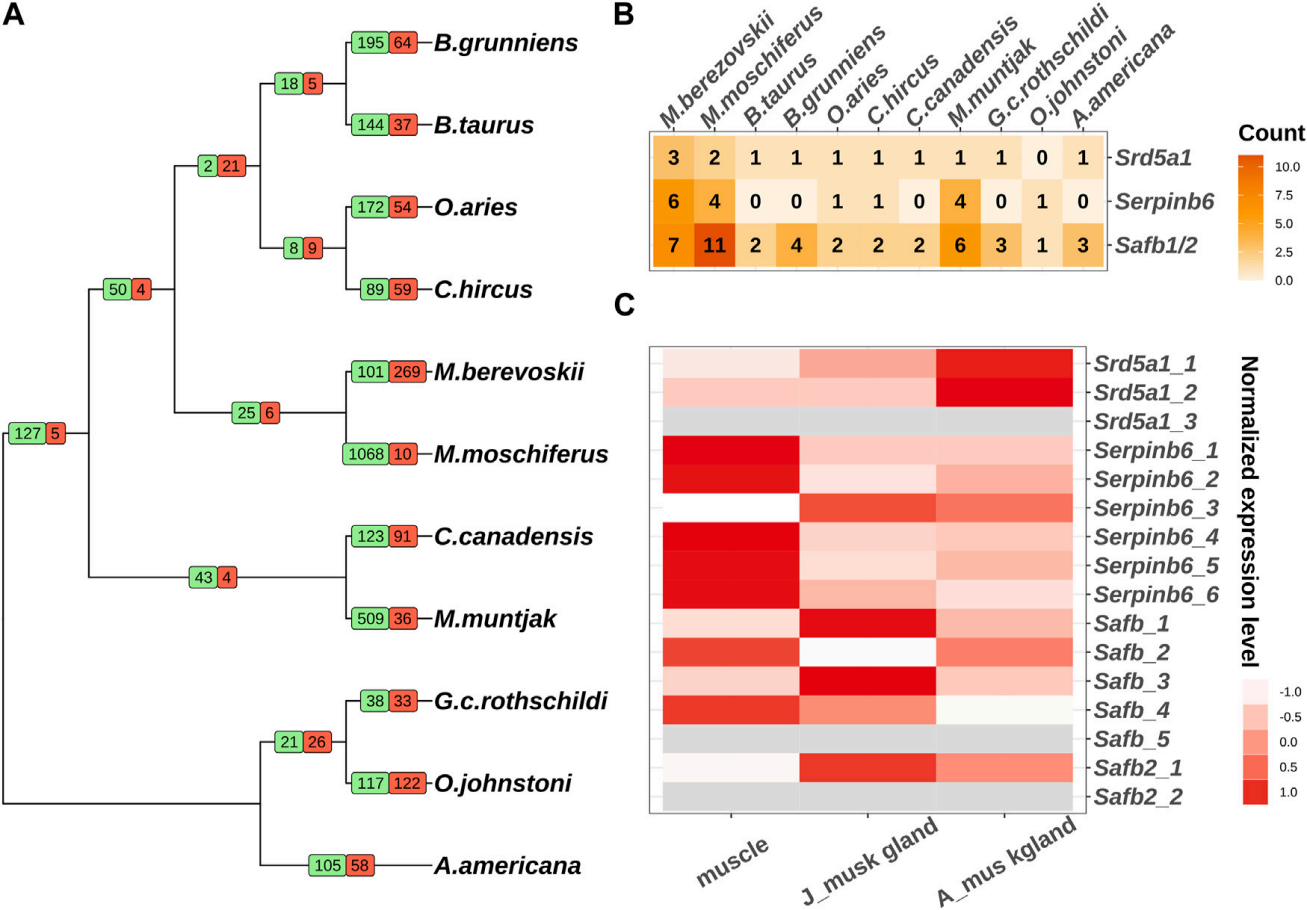
Comparative genomic analysis of 11 ruminants. **(A)** Gene family expansion and contraction of 11 ruminants. Branch numbers indicate the number of gene families that have expanded (green) and contracted (red) after the split from the common ancestor. **(B)** The copy numbers of *Srd5a1*, *Serpinb6* and *Safb1/2* in 11 ruminant genomes. **(C)** The expression pattern of individual copies of *Srd5a1*, *Serpinb6* and *Safb1/2* in muscles and musk glands of juvenile and adult Chinese forest musk deer. The grey colour represents the mRNA expression can’t be detected.

The emergence of specific functions and/or physiological traits is generally associated with changes in lineage-specific gene families. To explore the evolutionary events of the putative genes involved in musk gland formation, we conducted an analysis of gene family expansion and contraction in 11 ruminant genomes. In total, 25 gene families and 6 gene families were expanded and contracted in Moschus, respectively (Figure 1A; Supplementary Figure S1)*.* The genes involved in these families were selected to perform KEGG and GO analyses. The significantly enriched processes included transmembrane transport, sex differentiation, hydrolase activity and neuron projection development (Table 1).

**TABLE 1:** The functional enrichment analysis of the expanded and contracted gene families.

KEGG ID	Description	*p*-value
GO:0034764	Positive regulation of transmembrane transport	1.34E−03
GO:0007548	Sex differentiation	1.80E−03
GO:0051345	Positive regulation of hydrolase activity	9.32E−03
GO:0010975	Regulation of neuron projection development	9.89E−03

Next, we investigated the genes in sex differentiation pathway due to the close relationship between musk gland and musk deer reproductive behavior. Three steroid 5 alpha-reductase 1 (*Srd5a1*) orthologs, six serpin family B member 6 (*Serpinb6*) orthologs, five scaffold attachment Factor B (*Safb*) orthologs and two scaffold attachment Factor B2 (*Safb2*) orthologs were identified in the Chinese forest musk deer genome (Figure 1B). Among these, the expression of two *Srd5a1* orthologs, four *Safb1* orthologs, one *Safb2* and all *Serpinb6* orthologs could be detected in the muscle and musk gland (Figure 1C). It is well documented that *Srd5a1* plays a central role in steroidogenesis and sexual differentiation, while *Safb1/2* are corepressors of estrogen receptor alpha (Jiang et al., 2006). Moreover, the Serpinb6 family may play a critical role in gonad development, gametogenesis, and fertilization (Charron et al., 2006). Therefore, these genes, with high-level expressions in the musk gland, were likely molecular markers of the musk gland and regulatory factors of the musk-secreting function in musk deer. It is interesting that the numbers of these gene orthologs in the genomes of human and mouse are similar to other genomes from Ruminantia (Figure 1B; Supplementary Table S3). Collectively, the gene family expansion that occurred before the divergence of Moschidae may contribute to musk gland formation.

### 3.2 Bulk-transcriptomic analysis of musk glands at different developmental stages

**FIGURE 2:**
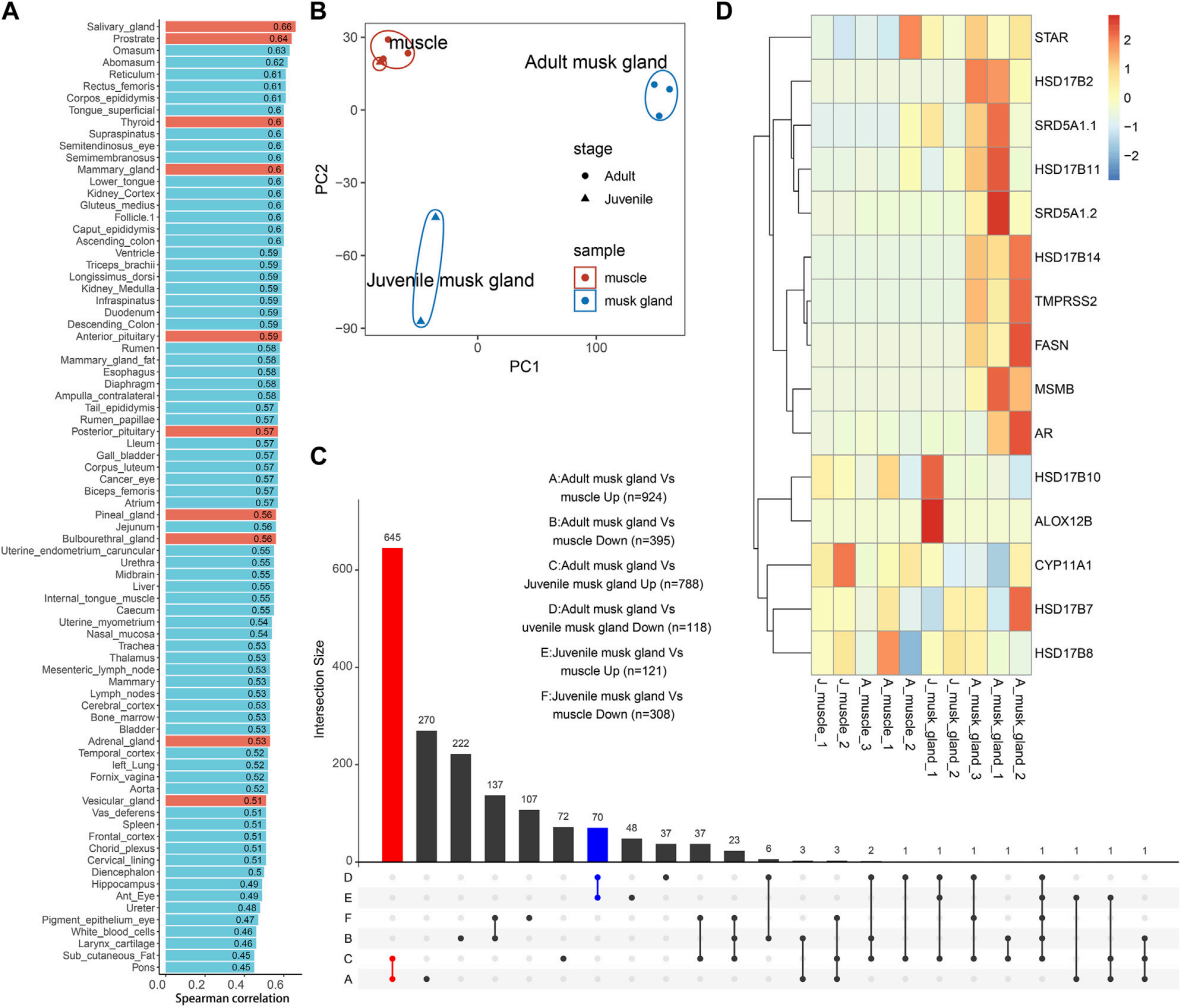
Bulk transcriptomic profiles of muscles and musk glands of adult and juvenile Chinese forest musk deer *M. berezovskii*. **(A)** Spearman correlation of expression profiles compared between adult musk glands and various *B. taurus* tissues. Glands of *B. taurus* are shown in red. **(B)** PCA plot of expression profiles of adult and juvenile musk glands and muscle samples. The colors of the points represent different tissue types, and the shapes of the points represent different sample stages. **(C)** UpSet plots showing the number of shared differentially expressed genes (|log2FoldChange|>1 and *p* <0.05) detected from different pairwise comparisons. The adult musk gland-specifically upregulated genes are shown in red (adult musk gland compared against juvenile musk glands and muscles, *n* = 645); the juvenile musk gland-specifically upregulated genes are shown in blue (juvenile musk gland compared against adult musk glands and muscles, *n* = 70). **(D)** Heatmap plot of the expression pattern of genes related to fatty acid synthesis and steroid hormone metabolism.

To further demonstrate the characteristics of the musk gland, the transcriptomes of muscles and musk glands collected from three adults and two juveniles were sequenced. A total of approximately 21,630 genes were detected, and principal component analysis (PCA) indicated that the samples were clearly divided into muscle, adult musk gland and juvenile musk gland groups (Figure 2B; Supplementary Table S4).

Based on the previous phylogenetic analysis, we compared the transcriptomes of adult musk glands and a dataset covering 92 bovine tissues (Supplementary Table S2). After analysis by Spearman correlation, the average coefficients among musk gland and various glands are from 0.45 to 0.66 (Figure 2A). As expected, the expression profile of the musk gland was closer to those of various glands than other tissues. To detect the differentially expressed genes (DEGs) involved in musk gland function, we then compared the musk gland data between the adult and the juvenile. For the upregulated genes in adults, a total of 645 genes were identified (Figure 2C; Supplementary Table S5) and were significantly enriched in the signaling pathways of fatty acid synthesis, energy metabolism and cell adhesion (Supplementary Table S6). The mainly enriched genes included Hydroxysteroid 17-beta dehydrogenase 6 (*Hsd17b6*)*,* Hydroxysteroid 17-beta dehydrogenase 14 (*Hsd17b14*)*,* Hydroxysteroid 17-beta dehydrogenase 10 (*Hsd17b10*)*,* Steroid 5-alpha-reductase 1 (*Srd5a1*) and Fatty acid synthase (*Fasn*) (Figure 2D). For the juveniles, only 70 upregulated genes were identified (Figure 2C; Supplementary Table S7). Among them, Arachidonate 12-lipoxygenase 12R Type (*Alox12b*), which is involved in lipid metabolism and mucus secretion regulation, was the most notable marker.

**FIGURE 3:**
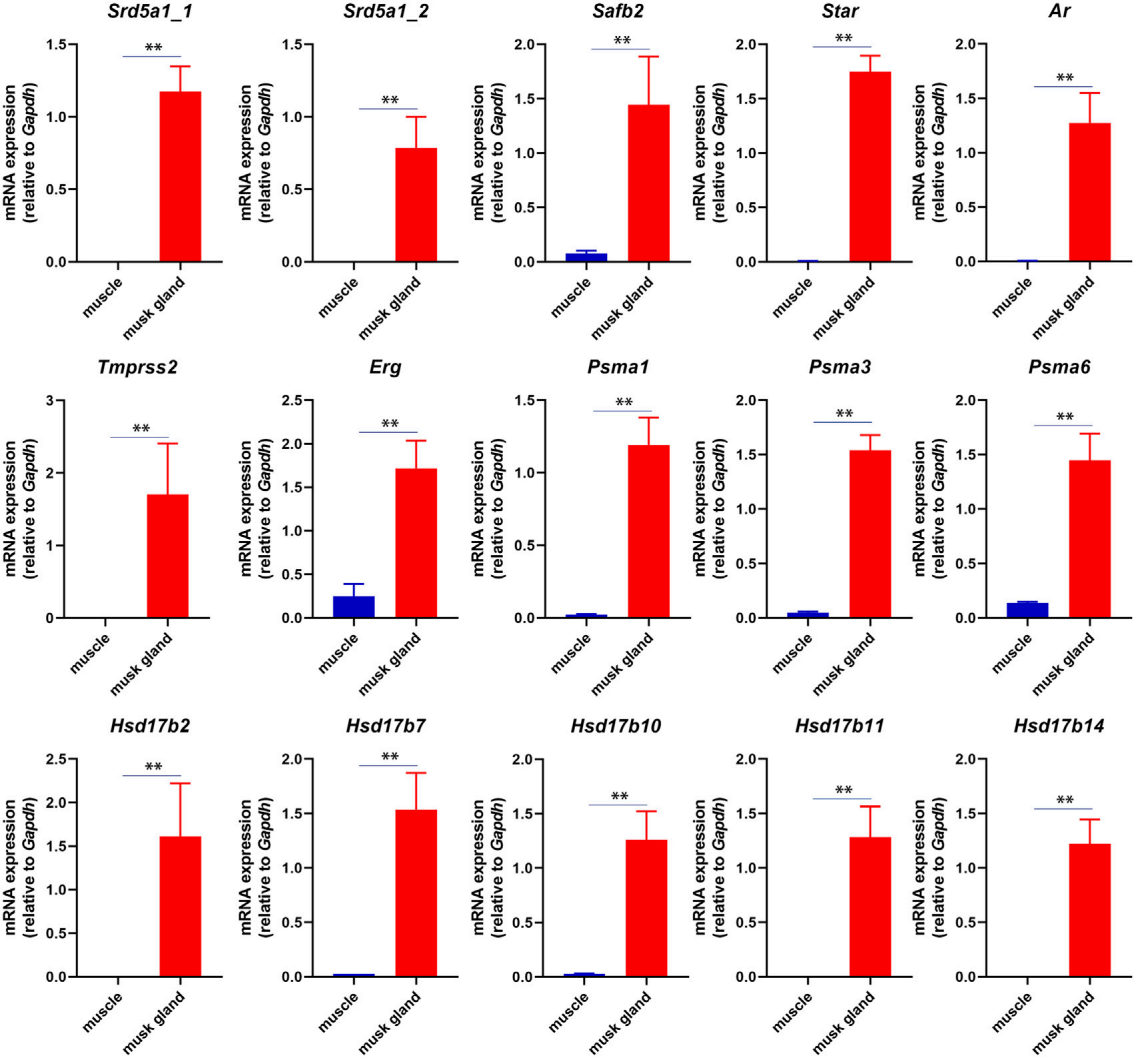
RT-PCR analysis of mRNA expression in musk gland and muscle. All quantitative data were obtained from three independent experiments and presented as the mean ± S.E.M. (**p < 0.01).

In musk deer, the musk gland is located between the abdomen and the genitals, similar to the location of prostate in mammals (Feng et al., 2023). Considering the location, the physiological function and the gene expression pattern, we speculated that the function of the musk gland was similar to that of the prostate gland. The RT-PCR results indeed indicated that several typical prostate marker genes, such as androgen receptor (*Ar*), prostate specific membrane antigens (*Psmas*), transmembrane serine protease 2 (*Tmprss2*) and ETS transcription factor (*Erg*), were robustly expressed in musk gland (Figure 3).

In short, these results suggested that the musk gland displayed a unique gene expression pattern that contained prostate marker genes.

### 3.3 Cell compositions of the musk gland

**FIGURE 4:**
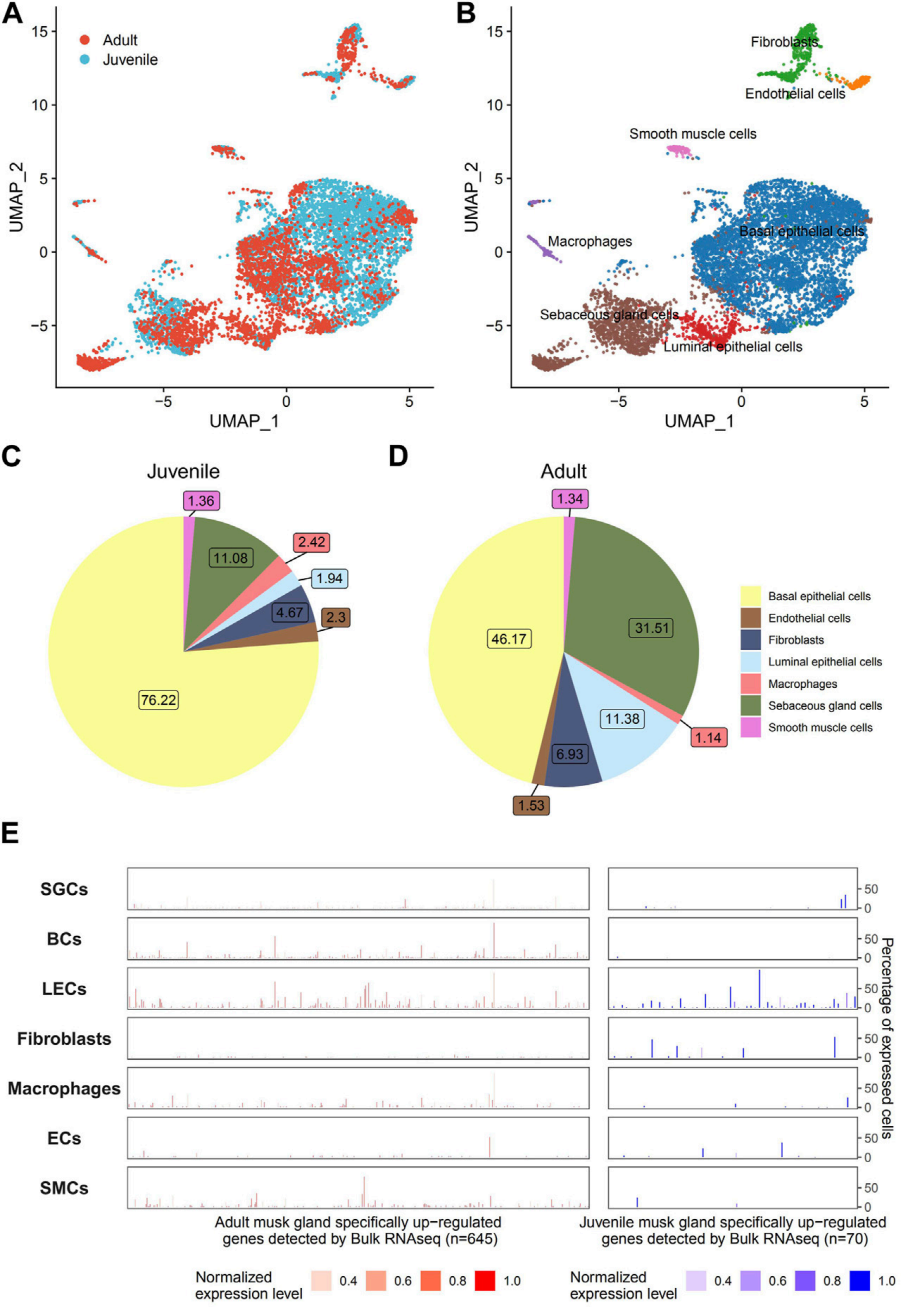
Single-cell transcriptomic profiles of musk glands of adult and juvenile Chinese forest musk deer. **(A)** UMAP projection of 8,465 musk gland cells, colored by phase. **(B)** Colored by cell type. **(C,D)** Cell type compositions of adult and juvenile musk glands. **(E)** The cell type level expression patterns of adult (left, *n* = 645) and juvenile (right, *n* = 70) musk glands specifically upregulated genes (detected by bulk RNA-seq). The height of the bar represents the percentage of expressed cells, and the color represents the normalized expression level.

To address cell composition and function, musk glands isolated from musk deer were subjected to single-cell sequencing. A total of 5,349 and 3,296 cells from juvenile and adult musk glands, respectively, were captured, with approximately 13,891 and 11,942 genes for the juvenile and adult musk glands, respectively. The cells from the two samples were combined and analyzed by Seurat SCTtrasform. Data were visualized by UMAP. With graph-based classification, 14 cell clusters were found and contained seven distinguishable cell types (Figures 4A, B; Supplementary Figures S2, S3). These cell types are basal epithelial cells (BCs), sebaceous gland cells (SGCs), luminal epithelial cells (LECs), fibroblasts, macrophages, endothelial cells (ECs) and smooth muscle cells (SMCs). Detailed analysis indicated that the highest proportions of cell types were BCs, SGCs and fibroblasts in the musk glands of juveniles and BCs, SGCs and LECs in those of adults (Figures 4C, D). During the development of the musk gland, the numbers of 2 cell types, namely, SGCs and LECs, steeply increased 2.84- and 5.93-fold, respectively. Importantly, the DEGs identified from the bulk transcriptomic profiles of musk glands at different developmental stages were mainly enriched in LECs. These results suggested that LECs may play critical roles in musk gland differentiation. (Figure 4E).

### 3.4 The potential regulatory pattern of musk gland cells

**FIGURE 5:**
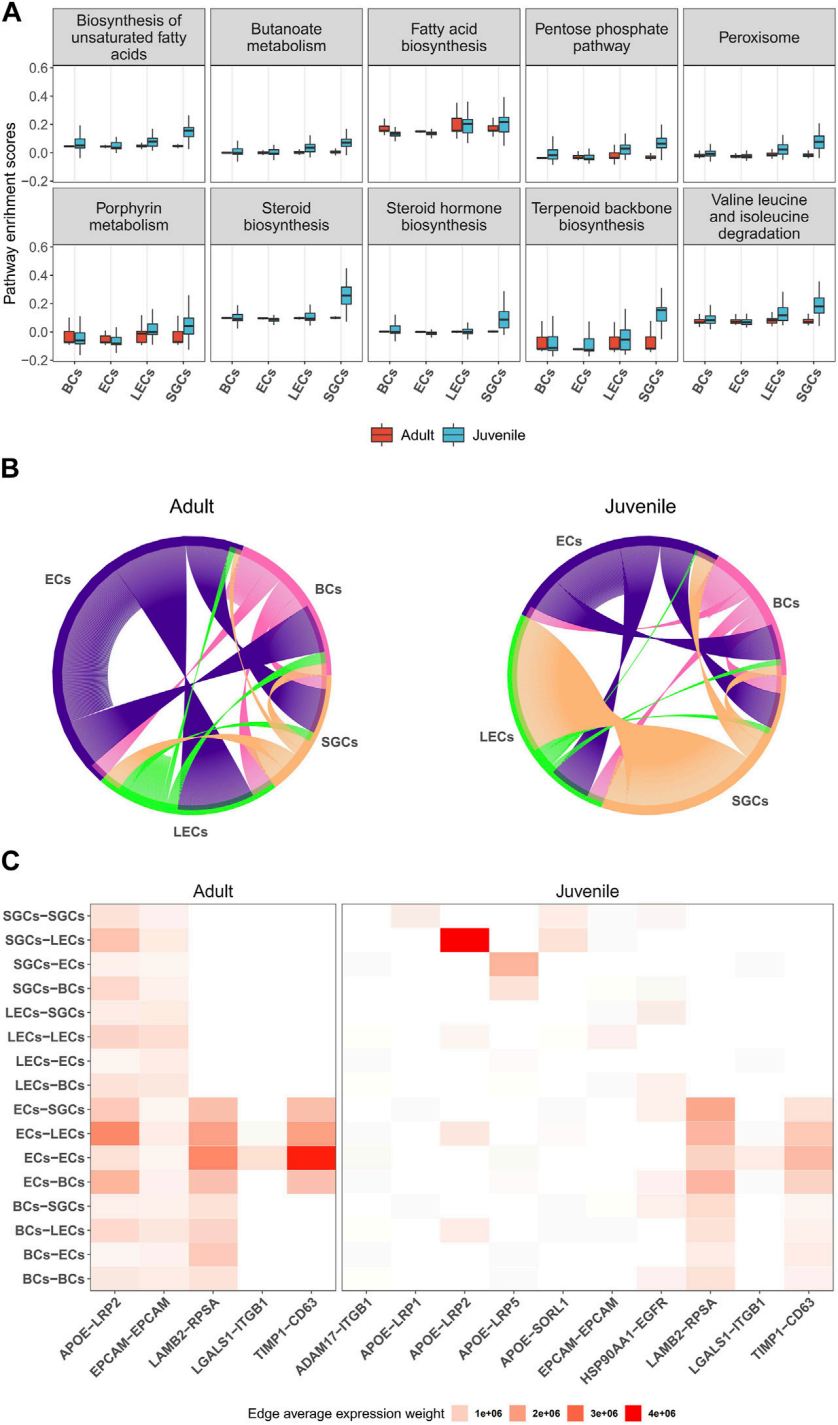
Characteristics and cell-to-cell columniations of 4 main musk gland cell types. **(A)** Boxplots depicting the distribution of single-cell GSVA scores of pathways in different cell types from adult and juvenile samples. **(B)** Chord diagrams plotting the amount of inferred ligand-receptor signaling across different cell types. Each line represents a ligand-receptor interaction with both ligand and receptor detection rate >0.1. **(C)** Heatmap of ligand-receptor average expression weight across different cell types.

Next, we investigated the potential regulatory functions of different cells in the musk gland by single-cell gene set variation analysis (scGSVA). Overall, the coordinated gene activity of the juvenile musk gland was significantly higher than that of the adult musk gland (Figure 5A). The most active signaling pathways in juveniles were unsaturated fatty acid biosynthesis, the pentose phosphate pathway and steroid biosynthesis. In adults, the pathways of fatty acid biosynthesis, porphyrin metabolism and terpenoid backbone biosynthesis were enriched. Among the 4 cell types, especially in juveniles, all signaling pathways related to biosynthesis were significantly activated in SGCs and LECs. These data strongly indicated that SGCs and LECs were most likely the main locations of musk synthesis.

To investigate the multicellular interaction of the musk gland, we constructed cell-to-cell communication networks using the NATMI program. The results indicated that the 4 cell types have substantial potential to communicate with each other in both juveniles and adults (Figure 5B). The ECs are likely to be the main signal sender in the microenvironment of the musk gland. With the maturation of the musk gland, the interaction strength gradually increased from the ECs to the other cells. In contrast, SGCs displayed significantly decreased interaction strength with LECs in adults compared with juveniles.

Based on the gene expression levels of the ligands and the receptors, the activities of intercellular communication in adults were found to be more vigorous than those in juveniles (Figure 5C). Interestingly, the most active signaling pathway is APOE-LRP2, which mediates cholesterol uptake and lipid delivery. In addition, our analysis suggested that ECs modulate cell behavior mainly by increasing the paracrine signaling of LAMB2-RPSA and TIMP1-CD63 (Figure 5C). Taken together, ECs exhibit a core status of regulating intercellular communication in musk gland development and function.

## 4 Discussion

There are six families in the suborder Ruminantia, including Tragulidae, Giraffidae, Antilocapridae, Moschidae, Cervidae, and Bovidae. Unlike other families, male animals belonging to the Moschidae have a unique tissue, the musk gland, with the capability of musk synthesis. Musk is a legendary ingredient of traditional oriental medicines and the fragrance industry; therefore, understanding the genetic basis of musk sac gland formation and regulatory networks of musk secretion can establish a solid foundation for increasing musk production.

Transcription factors contribute significantly to phenotypic variation (Konstantinides et al., 2018). In this study, we compared the genomics of 11 ruminants, including two species of Moschidae. We did not detect any obvious changes in the expansion or convergence of transcription factor genes before the divergence of Chinese forest musk deer and wild Siberian musk deer. However, at least two important gene-duplication events occurred in the *Srd5a1* and *Safb* families. *Srd5a1* is a rate-limiting enzyme in the androgen synthesis pathway that catalyzes the conversion of testosterone into the more potent androgen dihydrotestosterone (DHT). *Safb1/2* are transcriptional corepressors that repress estrogen receptor alpha-mediated transactivation and regulate cellular hormone metabolic processes (Hong et al., 2012). Due to the positive effect on musk secretion by sex steroids, the duplications of these positively selected genes may play important roles in the formation of the musk gland and the musk synthesis (Fan M. et al., 2018).

The analysis of molecular phylogeny indicated that Bovidae and Moschidae diverged from a common ancestor (Fan Z. et al., 2018; Zhou et al., 2019; Yi et al., 2020). After comparison with all tissues of cows, we found that the mRNA expression pattern of the musk gland was the closest to that of the prostate. As in the prostate, many genes in the musk gland, whose functions are related to androgen regulation, lipid synthesis and material secretion, were highly expressed. However, the expression of several prostate-specific genes, such as Kallikrein 4 (*Klk4*), NK3 homeobox 1 (*Nkx3.1*) and solute carrier family 45 member 3 (*Slc45a3*), was barely detected in the musk gland.

The production of androgen is a multistep catalytic process. In humans and rodents, the substrate cholesterol is transferred into the mitochondrial inner membrane by steroidogenic acute regulatory protein (StAR) and converted into pregnenolone by the cholesterol side-chain cleavage cytochrome P450 enzyme (CYP11A1). Pregnenolone is then converted to testosterone by 3β-hydroxysteroid dehydrogenase (HSD3B), cytochrome P450 17-alpha-hydroxylase/17,20-lyase (CYP17A1) and type 3/5 17β-hydroxysteroid dehydrogenase (HSD17B3/5) in the smooth endoplasmic reticulum (Li et al., 2022). Steroid 5α-reductases, including SRD5A1 and SRD5A2, are able to further convert testosterone into DHT. Immunohistochemical experiments have confirmed that steroidogenic enzymes, such as CYP11A1, HSD3B and CYP17A1, are expressed in immature and mature musk glands (Yang et al., 2021). In this study, *Star*, *Srd5a1* and multiple *Hsd17b* enzyme genes were also detected in musk glands by RT-PCR. These findings suggested the following: 1) the musk gland may possess an intact androgen-synthesized pathway, while these enzymes may exist in 1 cell or disperse in different cells; 2) the androgens, participating in the regulation of musk synthesis and secretion, may be provided by the Leydig cells in the testis and/or the musk gland itself.

The chemical components of the mature musk include macrocyclic ketone compounds, pyridine compounds, sex hormone derivatives, proteins, fatty acids, inorganic salts, and a complex microbiota (Li et al., 2016; Li et al., 2018). Our single-cell sequencing revealed that the musk gland mainly consists of 4 cell types, namely, BCs, LECs, ECs and SGCs. In these cells, metabolic processes related to musk synthesis, such as porphyrin metabolism, steroid hormone biosynthesis, terpenoid synthesis, fatty acid biosynthesis and ATP generation, were highly active. A previous study reported that the musk produced from unmated males has more muscone and cholesterol (Li et al., 2018). Indeed, our results also indicated that the expression of active genes in SGCs and LECs of juvenile musk glands was significantly higher than that in adult musk glands.

Cell-to-cell interactions are critical for communication between different cells and are the fundamental basis of cellular function implementation. Our results indicated that the interaction strength regulated by the ECs is obviously elevated during the development of the musk gland, suggesting that the cells play a central role in the function of the musk gland. For BCs, the dominant cell type in juvenile and adult musk glands, no obvious regulatory functions were detected based on our integrated analysis. As they expressed multiple cell adhesion molecules, such as EPCAM, BCs may perform functions to maintain ductal integrity and facilitate cell–cell communication (Gires et al., 2020).

In summary, we compared the musk deer genome and analyzed the characteristics of the musk gland by integrated multiomics technologies. The musk gland displays a prostate-like gene expression pattern. There are four dominant cell types in the musk gland that implement different functions. SGCs and LECs play important roles in musk synthesis. BCs and ECs provide essential functions in regulating cell-to-cell communication and maintaining the stability of the musk gland microenvironment, respectively. Our study lays a foundation for the understanding of musk gland formation and scale production of musk.

## Data Availability

RNA sequencing data are available on the Bioproject database of the National Center for Biotechnology Information: PRJNA928235 (https://www.ncbi.nlm.nih.gov/bioproject/).

## References

[B1] Andrews S. (2023). FastQC: A quality control tool for high throughput sequence data. Available online: http://www.bioinformatics.babraham.ac.uk/projects/fastqc (accessed January 4th, 2023).

[B2] Becht E. McInnes L. Healy J. Dutertre C. A. Kwok I. W. H. Ng L. G. (2019). Dimensionality reduction for visualizing single-cell data using UMAP. Nat. Biotechnol. 37, 38–44. 10.1038/nbt.4314 30531897

[B3] Bolger A. M. Lohse M. Usadel B. (2014). Trimmomatic: A flexible trimmer for Illumina sequence data. Bioinformatics 30, 2114–2120. 10.1093/bioinformatics/btu170 24695404 PMC4103590

[B4] Charron Y. Madani R. Nef S. Combepine C. Govin J. Khochbin S. (2006). Expression of serpinb6 serpins in germ and somatic cells of mouse gonads. Mol. Reprod. Dev. 73 (1), 9–19. 10.1002/mrd.20385 16175637

[B5] Choudhary S. Satija R. (2022). Comparison and evaluation of statistical error models for scRNA-seq. Genome Biol. 23, 27. 10.1186/s13059-021-02584-9 35042561 PMC8764781

[B6] Conway J. R. Lex A. Gehlenborg N. (2017). UpSetR: an R package for the visualization of intersecting sets and their properties. Bioinformatics 33, 2938–2940. 10.1093/bioinformatics/btx364 28645171 PMC5870712

[B7] De Bie T. Cristianini N. Demuth J. P. Hahn M. W. (2006). Cafe: A computational tool for the study of gene family evolution. Bioinformatics 22, 1269–1271. 10.1093/bioinformatics/btl097 16543274

[B8] Emms D. M. Kelly S. (2019). OrthoFinder: Phylogenetic orthology inference for comparative genomics. Genome Biol. 20, 238. 10.1186/s13059-019-1832-y 31727128 PMC6857279

[B9] Ewels P. A. Peltzer A. Fillinger S. Patel H. Alneberg J. Wilm A. (2020). The nf-core framework for community-curated bioinformatics pipelines. Nat. Biotechnol. 38, 276–278. 10.1038/s41587-020-0439-x 32055031

[B10] Fan M. Zhang M. Shi M. Zhang T. Qi L. Yu J. (2018a). Sex hormones play roles in determining musk composition during the early stages of musk secretion by musk deer (*Moschus berezovskii*). Endocr. J. 65, 1111–1120. 10.1507/endocrj.EJ18-0211 30175720

[B11] Fan Z. Li W. Jin J. Cui K. Yan C. Peng C. (2018b). The draft genome sequence of forest musk deer (*Moschus berezovskii*). Gigascience 7, giy038. 10.1093/gigascience/giy038 29635287 PMC5906906

[B12] Fang L. Cai W. Liu S. Canela-Xandri O. Gao Y. Jiang J. (2020). Comprehensive analyses of 723 transcriptomes enhance genetic and biological interpretations for complex traits in cattle. Genome Res. 30, 790–801. 10.1101/gr.250704.119 32424068 PMC7263193

[B13] Feng H. Wang L. Cao F. Ma j. Tang j. Feng C. (2023). Forest musk deer (*Moschus berezovskii*) in China: Research and protection. J. Vertebrate Biol. 72 (22067), 1–13. 10.1080/10255842.2022.2163849

[B14] Fu W. Wang R. Nanaei H. A. Wang J. Hu D. Jiang Y. (2022). RGD v2.0: A major update of the ruminant functional and evolutionary genomics database. Nucleic Acids Res. 50, D1091–d1099. 10.1093/nar/gkab887 34643708 PMC8728256

[B15] Gires O. Pan M. Schinke H. Canis M. Baeuerle P. A. (2020). Expression and function of epithelial cell adhesion molecule EpCAM: Where are we after 40 years? Cancer Metastasis Rev. 39, 969–987. 10.1007/s10555-020-09898-3 32507912 PMC7497325

[B16] Hänzelmann S. Castelo R. Guinney J. (2013). Gsva: Gene set variation analysis for microarray and RNA-seq data. BMC Bioinforma. 14, 7. 10.1186/1471-2105-14-7 PMC3618321 23323831

[B17] Hao Y. Hao S. Andersen-Nissen E. Mauck W. M. Zheng S. Butler A. (2021). Integrated analysis of multimodal single-cell data. Cell 184, 3573–3587.e29. 10.1016/j.cell.2021.04.048 34062119 PMC8238499

[B18] Hoff K. J. Lomsadze A. Borodovsky M. Stanke M. (2019). Whole-genome annotation with BRAKER. Methods Mol. Biol. 1962, 65–95. 10.1007/978-1-4939-9173-0_5 31020555 PMC6635606

[B19] Hong E. A. Gautrey H. L. Elliott D. J. Tyson-Capper A. J. (2012). SAFB1- and SAFB2-mediated transcriptional repression: Relevance to cancer. Biochem. Soc. Trans. 40, 826–830. 10.1042/bst20120030 22817742

[B20] Hou R. Denisenko E. Ong H. T. Ramilowski J. A. Forrest A. R. R. (2020). Predicting cell-to-cell communication networks using NATMI. Nat. Commun. 11, 5011. 10.1038/s41467-020-18873-z 33024107 PMC7538930

[B21] Jiang S. Meyer R. Kang K. Osborne C. K. Wong J. Oesterreich S. (2006). Scaffold attachment factor SAFB1 suppresses estrogen receptor alpha-mediated transcription in part via interaction with nuclear receptor corepressor. Mol. Endocrinol. 20, 311–320. 10.1210/me.2005-0100 16195251

[B22] Jie H. Xu Z. Gao J. Li F. Chen Y. Zeng D. (2021). Differential expression profiles of microRNAs in musk gland of unmated and mated forest musk deer (*Moschus berezovskii*). Peer J. 9, e12710. 10.7717/peerj.12710 35036174 PMC8710055

[B23] Jones P. Binns D. Chang H. Y. Fraser M. Li W. McAnulla C. (2014). InterProScan 5: Genome-scale protein function classification. Bioinformatics 30, 1236–1240. 10.1093/bioinformatics/btu031 24451626 PMC3998142

[B24] Kim D. Paggi J. M. Park C. Bennett C. Salzberg S. L. (2019). Graph-based genome alignment and genotyping with HISAT2 and HISAT-genotype. Nat. Biotechnol. 37, 907–915. 10.1038/s41587-019-0201-4 31375807 PMC7605509

[B25] Konstantinides N. Kapuralin K. Fadil C. Barboza L. Satija R. Desplan C. (2018). Phenotypic convergence: Distinct transcription factors regulate common terminal features. Cell 174, 622–635. 10.1016/j.cell.2018.05.021 29909983 PMC6082168

[B26] Li D. Chen B. Zhang L. Gaur U. Ma T. Jie H. (2016). The musk chemical composition and microbiota of Chinese forest musk deer males. Sci. Rep. 6, 18975. 10.1038/srep18975 26744067 PMC4705530

[B27] Li Y. Zhang T. Qi L. Yang S. Xu S. Cha M. (2018). Microbiota changes in the musk gland of male forest musk deer during musk maturation. Front. Microbiol. 9, 3048. 10.3389/fmicb.2018.03048 30619139 PMC6297183

[B28] Li Z. H. Lu J. D. Li S. J. Chen H. L. Su Z. J. (2022). Generation of leydig-like cells: Approaches, characterization, and challenges. Asian J. Androl. 24, 335–344. 10.4103/aja202193 35017389 PMC9295467

[B29] Liu K. Xie L. Deng M. Zhang X. Luo J. Li X. (2021). Zoology, chemical composition, pharmacology, quality control and future perspective of musk (Moschus): A review. Chin. Med. 16, 46. 10.1186/s13020-021-00457-8 34147113 PMC8214773

[B30] Love M. I. Huber W. Anders S. (2014). Moderated estimation of fold change and dispersion for RNA-seq data with DESeq2. Genome Biol. 15, 550. 10.1186/s13059-014-0550-8 25516281 PMC4302049

[B31] Manni M. Berkeley M. R. Seppey M. Simão F. A. Zdobnov E. M. (2021). BUSCO update: Novel and streamlined workflows along with broader and deeper phylogenetic coverage for scoring of eukaryotic, prokaryotic, and viral genomes. Mol. Biol. Evol. 38, 4647–4654. 10.1093/molbev/msab199 34320186 PMC8476166

[B32] Meng X. Liu D. Feng J. Meng Z. (2012). Asian medicine: Exploitation of plants. Science 335, 1168–1169. 10.1126/science.335.6073.1168-b 22403370

[B33] Pertea M. Pertea G. M. Antonescu C. M. Chang T. C. Mendell J. T. Salzberg S. L. (2015). StringTie enables improved reconstruction of a transcriptome from RNA-seq reads. Nat. Biotechnol. 33, 290–295. 10.1038/nbt.3122 25690850 PMC4643835

[B34] Wu T. Hu E. Xu S. Chen M. Guo P. Dai Z. (2021). clusterProfiler 4.0: A universal enrichment tool for interpreting omics data. Innov. (Camb). 2, 100141. 10.1016/j.xinn.2021.100141 PMC8454663 34557778

[B35] Xu Z. Jie H. Chen B. Gaur U. Wu N. Gao J. (2017). Illumina-based de novo transcriptome sequencing and analysis of Chinese forest musk deer. J. Genet. 96, 1033–1040. 10.1007/s12041-017-0872-x 29321364

[B36] Yang J. Peng G. Shu F. Dong D. Zheng X. Zhu C. (2021). Characteristics of steroidogenesis-related factors in the musk gland of Chinese forest musk deer (*Moschus berezovskii*). J. Steroid Biochem. Mol. Biol. 212, 105916. 10.1016/j.jsbmb.2021.105916 34010686

[B37] Yang Q. S. Meng X. X. Xia L. Feng Z. J. (2003). Conservation status and causes of decline of musk deer (*Moschus spp.*) in China. Biol. Conserv. 109, 333–342. 10.1016/s0006-3207(02)00159-3

[B38] Yi L. Dalai M. Su R. Lin W. Erdenedalai M. Luvsantseren B. (2020). Whole-genome sequencing of wild Siberian musk deer (*Moschus moschiferus*) provides insights into its genetic features. BMC Genomics 21, 108. 10.1186/s12864-020-6495-2 32005147 PMC6995116

[B39] Zhang Y. Parmigiani G. Johnson W. E. (2020). ComBat-seq: Batch effect adjustment for RNA-seq count data. Nar. Genom Bioinform 2, lqaa078. lqaa078. 10.1093/nargab/lqaa078 33015620 PMC7518324

[B40] Zhou C. Zhang W. Wen Q. Bu P. Gao J. Wang G. (2019). Comparative genomics reveals the genetic mechanisms of musk secretion and adaptive immunity in Chinese forest musk deer. Genome Biol. Evol. 11, 1019–1032. 10.1093/gbe/evz055 30903183 PMC6450037

