# Supplementary material for: Integrated multi-omics analysis reveals insights into Chinese forest musk deer (Moschus berezovskii) genome evolution and musk synthesis
Source: Front Cell Dev Biol. 2023 May 9;11:1156138. doi: 10.3389/fcell.2023.1156138 (PMC10203155; doi:10.3389/fcell.2023.1156138)
Supplement: Supplementary file 1 [file DataSheet1.zip › Data Sheet 1/4-Supplementary Information_2023_0412.pdf]

Supplementary Information for

Integrated multi-omics analysis reveals insights into Chinese forest musk deer (*Moschus berezovskii*) genome evolution and musk synthesis

Hui Feng[1#], Tingyin Feng[2#], Yidi Mo[3#], Suli Sun[2], Lu Wang[1], Chunbin Lu[4], Chengli Feng[*1], Ke Xing[*2], Zhijian Su[*1,3,5]

1. Shaanxi Institute of Zoology, Xi'an, Shaanxi 710032, China.
2. School of Life Sciences, Guangzhou University, Guangzhou 510655, China;
3. Department of Cell Biology, Jinan University, Guangzhou 510632, China;
4. Department of Developmental Biology and Regenerative Medicine, Jinan University, Guangzhou, 510632, China

5 National Engineering Research Center of Genetic Medicine, Jinan University, Guangzhou 510632, China.

[#] These Authors have contributed equally to the work.

***To whom correspondence should be addressed:**

Zhijian Su:

No. 601, Huangpu Blvd., Department of Cell Biology, School of Life Science and Technology, Jinan University, Guangzhou, Guangdong, China, 510632;
Phone: 86-13751850387
E-mail: tjnuszj@jnu.edu.cn

This PDF file includes:
Figures S1 to S3
Tables S1 to S7

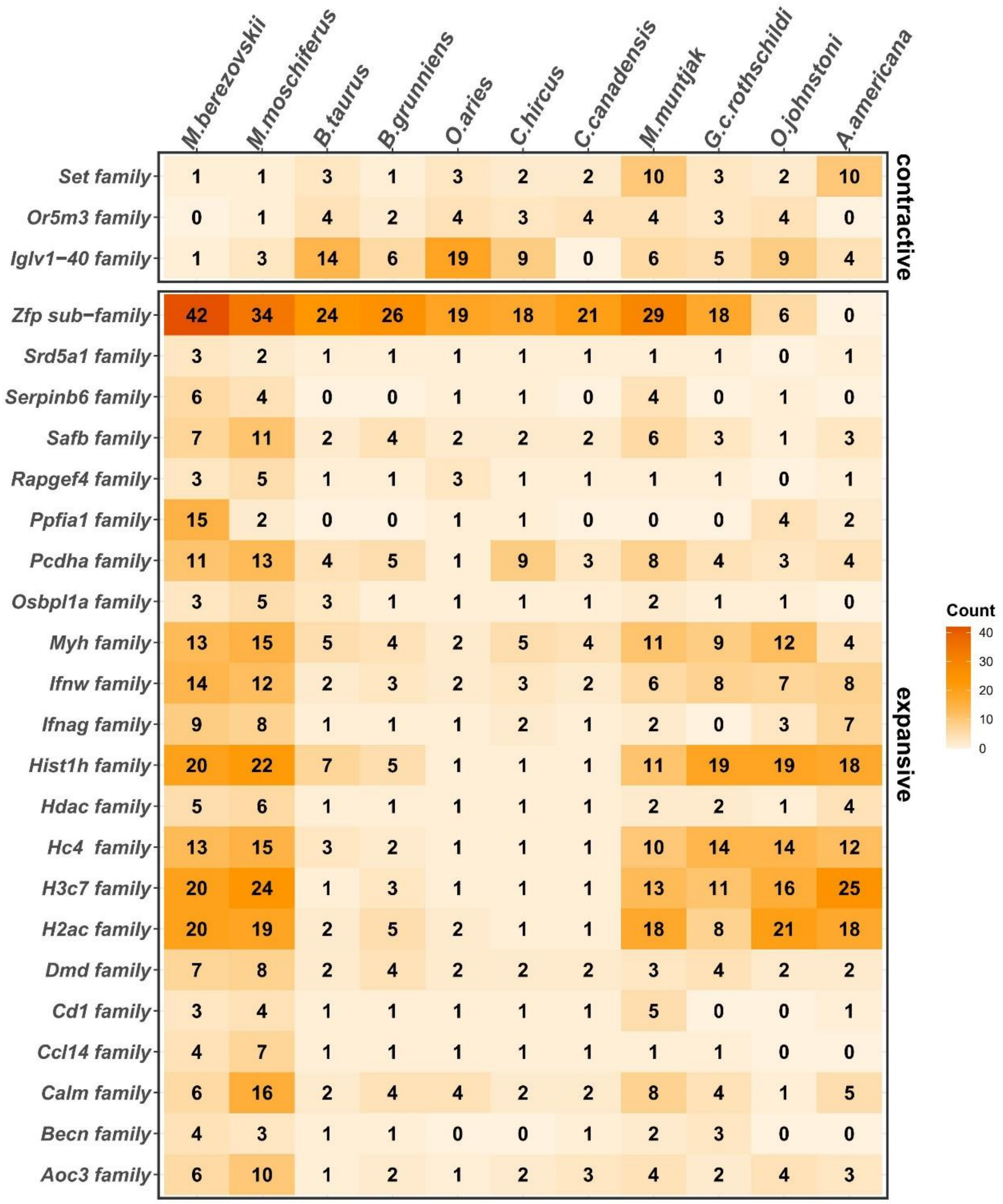

Figure S1. The copy numbers of expansive and contractive families in 11 ruminant genomes

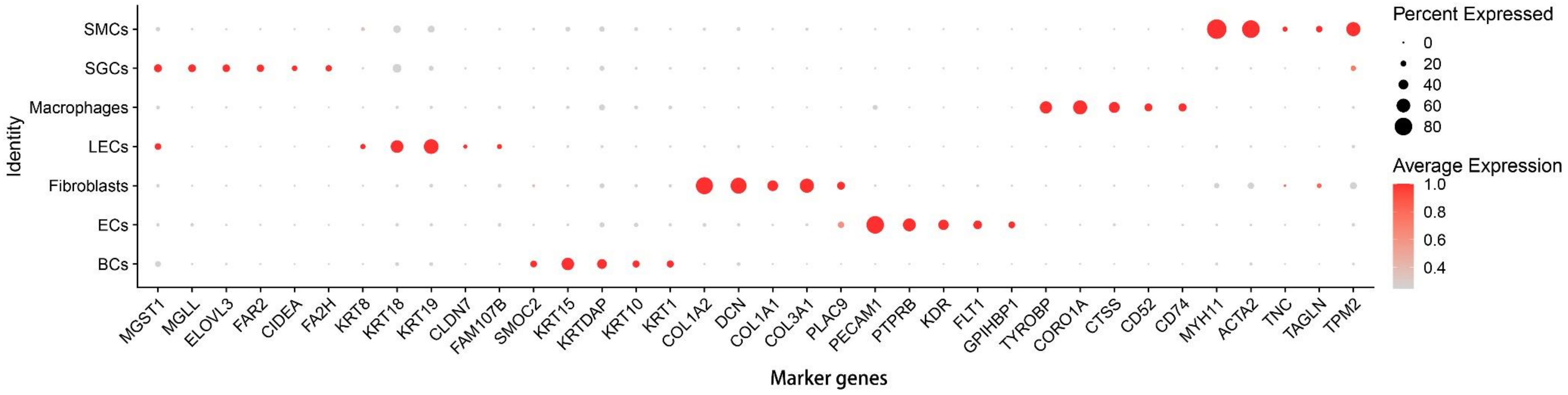

Figure S2. The marker genes used for cell type identification in the musk gland

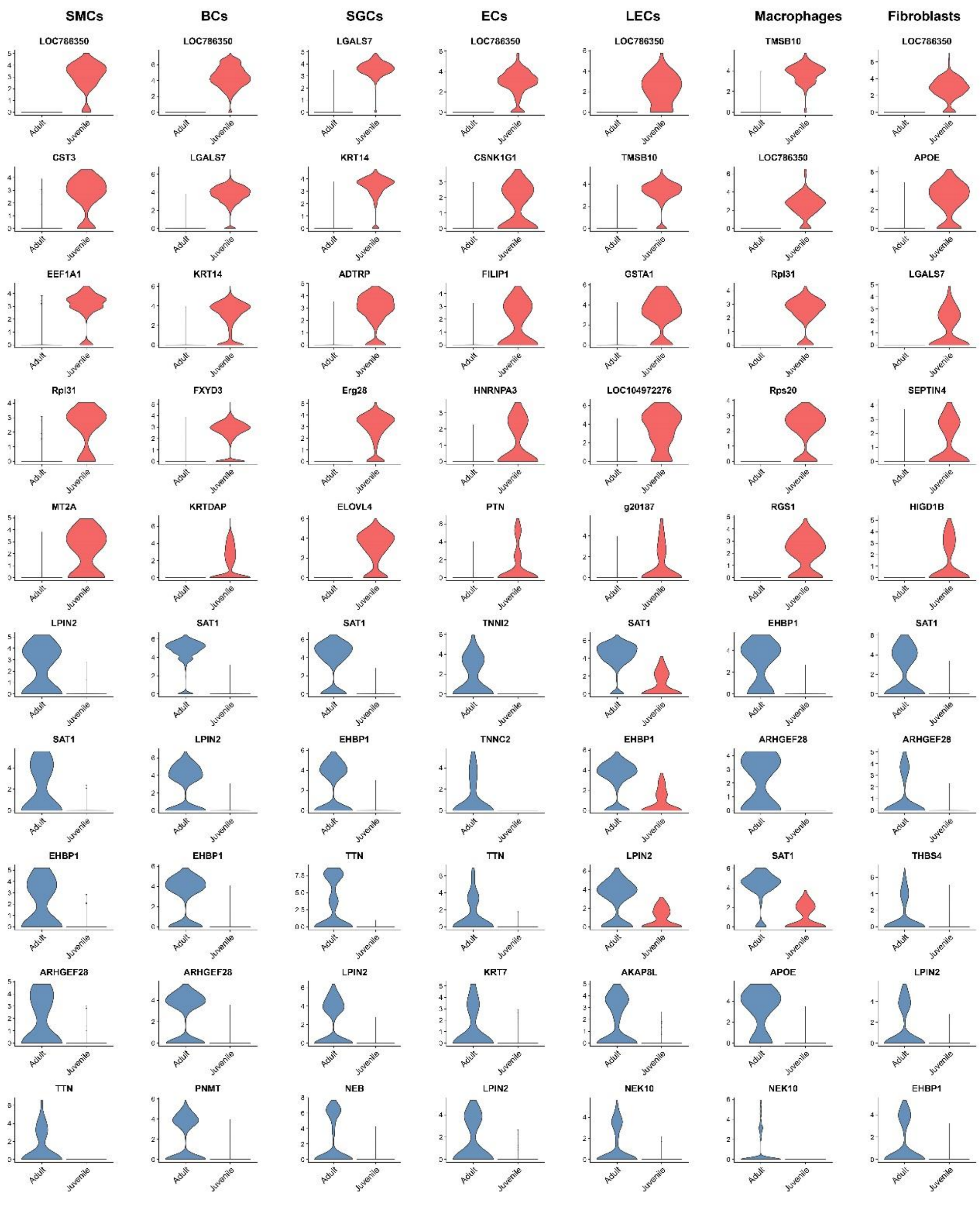

Figure S3. The expression distribution of top 10 DEGs in each cell type between the adult and the juvenile.

The Y-axis represents the scaled expression levels of DEG genes.

Table S1. The list of RT-PCR primers

Gene name	Forward (5'-3')	Reverse (5'-3')
Ar	CTGGAGCACTGGACGAGGT	CTTAATGCGGGCATGAGG
Erg	TCTGTCGCACCTCCACTACC	TGGGTGGTTCGTAAGGTAAATC
Gapdh	TCACCATCTTCCAGGAGCAA	TCAAGTGAGCCCCAGCCT
Hsd17b2	CAAGGTTGCGATTCTGGGT	ACCTTCTCCTTCACTTTGCTGT
Hsd17b7	ATCAGGGTCTGTATTCCAGTGTAGT	CATTGTGAGGTGTCAGCGTG
Hsd17b10	ATCATCAACACAGCCAGCG	TTATCTGGGAGGGTGGTCAA
Hsd17b11	TTGAGGAAACAGCCACCG	CTGAGGGTCTTGTGTAGCGAA
Hsd17b14	GACTCGGGAGGAAGATGTGG	TGTGGAGGTGGGTGGTAGC
Psma1	TCAAACAAGGTTCAGCCACA	TCAGCAGTAAGTCCCGCAA
Psma 3	GTTTCATATGGTTATTGGGGCT	CACGGCAGGTCATTTCTTTC
Psma 6	CAGGTACAGAGGGCACGCTA	AAATCATACAACAACCAAGAGGC
Safb2	TGAAAACGCCTATTCGGGCA	TAGCGGCTCTGAGCACAAAA
Srd5a1_1	TTCGGAGAAGTCGTGGAGTG	GGATAATCTTCAAATTTCTCATGGT
Srd5a1_2	GGAGTGGTGCGGCTATGC	TGTTTTCGAACGTACTTGTGATG
Star	ACGAGGTGCTGAGTAAAGTGATC	TCCTTGACATTGGGGTTCC
Tmprss2	GTGCTCAGGGATGGAGTGTG	TAGAGGCGAACACAGCGGT

Table S2. The details of the 11 ruminant genomes

Organism name	Common Name	Genome assembly version	BUSCO
Antilocapra americana	Pronghorn	GSC_phorn_1.0 (GCA_007570785.1)	80.40%
Bos grunniens	Yak	LU_Bosgru_v3.0 (GCA_005887515.1)	93.00%
Bos taurus	Cow	ARS-UCD1.2 (GCA_002263795.2)	99.20%
Capra hircus	Goat	ARS1 (GCA_001704415.1)	99.20%
Cervus canadensis	Elk	ASM1932006v1 (GCA_019320065.1)	99.20%
Giraffa camelopardalis rothschildi	Giraffa	ASM1759144v1 (GCA_017591445.1)	87.80%
Moschus berezovskii	Dwarf musk deer	FMD（GCA_006459085.1）	90.50%
Moschus moschiferus	Siberian musk deer	MosMos_v2_BIUU_UCD (GCA_004024705.2)	98.40%
Muntiacus muntjak	Red muntjac	UCB_Mmun_1.0 (GCA_008782695.1)	85.90%
Okapia johnstoni	Okapi	Okapi (RGP)	45.10%
Ovis aries	Sheep	Oar_rambouillet_v1.0 (GCA_002742125.1)	97.60%

Table S3. The comparison of gene numbers

Gene	Human	Mouse	M. berezovskii	M.moschiferus
Serpinb6	**2**	**5**	**6**	**4**
Safb1/2	**2**	**2**	**7**	**11**
Srd5a1	**1**	**1**	**3**	**2**

Table S4. The comparison of DEGs in muscle between the adult and the juvenile.

Table S5. The lists of upregulated DEGs in the musk gland of the adult.

Table S7. The lists of upregulated DEGs in the musk gland of the juvenile.

These tables (4/5/7) are provided by separated attachments.

Table S6. The KEGG analysis of upregulating DEGs in adult musk gland compared to the juvenile

KEGG ID	Description	Enrichment Fold	*P*-value
bta00350	**Tyrosine metabolism**	6.65	7.02E-05
bta00062	**Fatty acid elongation**	6.39	9.58E-04
bta01040	**Biosynthesis of unsaturated fatty acids**	6.18	1.13E-03
bta05033	**Nicotine addiction**	5.56	6.44E-04
bta00260	**Glycine, serine and threonine metabolism**	4.94	1.22E-03
bta00480	**Glutathione metabolism**	4.78	2.40E-04
bta00982	**Drug metabolism - cytochrome P450**	4.19	1.33E-03
bta05150	**Staphylococcus aureus infection**	3.46	6.02E-04
bta04530	**Tight junction**	3.33	7.02E-05
bta04514	**Cell adhesion molecules**	3.25	9.58E-04
