# Supplementary material for: Integrated multi-omics analysis reveals insights into Chinese forest musk deer (Moschus berezovskii) genome evolution and musk synthesis
Source: Front Cell Dev Biol. 2023 May 9;11:1156138. doi: 10.3389/fcell.2023.1156138 (PMC10203155; doi:10.3389/fcell.2023.1156138)
Supplement: Supplementary file 1 [file DataSheet1.zip › Data Sheet 1/Table S1 RT-PCR primers_2023_RE_C.pdf]

Table 1. The list of RT-PCR primers

Gene name	Forward (5'-3')	Reverse (5'-3')
Ar	CTGGAGCACTGGACGAGGT	CTTAATGCGGGCATGAGG
Erg	TCTGTCGCACCTCCACTACC	TGGGTGGTTCGTAAGGTAAATC
Gapdh	TCACCATCTTCCAGGAGCAA	TCAAGTGAGCCCCAGCCT
Hsd17b2	CAAGGTTGCGATTCTGGGT	ACCTTCTCCTTCACTTTGCTGT
Hsd17b7	ATCAGGGTCTGTATTCCAGTGTAGT	CATTGTGAGGTGTCAGCGTG
Hsd17b10	ATCATCAACACAGCCAGCG	TTATCTGGGAGGGTGGTCAA
Hsd17b11	TTGAGGAAACAGCCACCG	CTGAGGGTCTTGTGTAGCGAA
Hsd17b14	GACTCGGGAGGAAGATGTGG	TGTGGAGGTGGGTGGTAGC
Psma1	TCAAACAAGGTTCAGCCACA	TCAGCAGTAAGTCCCGCAA
Psma 3	GTTTCATATGGTTATTGGGGCT	CACGGCAGGTCATTTCTTTC
Psma 6	CAGGTACAGAGGGCACGCTA	AAATCATACAACAACCAAGAGGC
Safb2	TGAAAACGCCTATTCGGGCA	TAGCGGCTCTGAGCACAAAA
Srd5a1_1	TTCGGAGAAGTCGTGGAGTG	GGATAATCTTCAAATTTCTCATGGT
Srd5a1_2	GGAGTGGTGCGGCTATGC	TGTTTTCGAACGTACTTGTGATG
Star	ACGAGGTGCTGAGTAAAGTGATC	TCCTTGACATTGGGGTTCC
Tmprss2	GTGCTCAGGGATGGAGTGTG	TAGAGGCGAACACAGCGGT
