# Supplementary material for: Integrated multi-omics analysis reveals insights into Chinese forest musk deer (Moschus berezovskii) genome evolution and musk synthesis
Source: Front Cell Dev Biol. 2023 May 9;11:1156138. doi: 10.3389/fcell.2023.1156138 (PMC10203155; doi:10.3389/fcell.2023.1156138)
Supplement: Supplementary file 1 [file DataSheet1.zip › Data Sheet 1/Table S2 The details of the 11 ruminant genomes_RE_C.pdf]

Table S2. The details of the 11 ruminant genomes

Organism name	Common Name	Genome assembly version	BUSCO
Antilocapra americana	Pronghorn	GSC_phorn_1.0 (GCA_007570785.1)	80.40%
Bos grunniens	Yak	LU_Bosgru_v3.0 (GCA_005887515.1)	93.00%
Bos taurus	Cow	ARS-UCD1.2 (GCA_002263795.2)	99.20%
Capra hircus	Goat	ARS1 (GCA_001704415.1)	99.20%
Cervus canadensis	Elk	ASM1932006v1 (GCA_019320065.1)	99.20%
Giraffa camelopardalis rothschildi	Giraffa	ASM1759144v1 (GCA_017591445.1)	87.80%
Moschus berezovskii	Dwarf musk deer	FMD（GCA_006459085.1）	90.50%
Moschus moschiferus	Siberian musk deer	MosMos_v2_BIUU_UCD (GCA_004024705.2)	98.40%
Muntiacus muntjak	Red muntjac	UCB_Mmun_1.0 (GCA_008782695.1)	85.90%
Okapia johnstoni	Okapi	Okapi (RGP)	45.10%
Ovis aries	Sheep	Oar_rambouillet_v1.0 (GCA_002742125.1)	97.60%
