# Supplementary material for: Integrated multi-omics analysis reveals insights into Chinese forest musk deer (Moschus berezovskii) genome evolution and musk synthesis
Source: Front Cell Dev Biol. 2023 May 9;11:1156138. doi: 10.3389/fcell.2023.1156138 (PMC10203155; doi:10.3389/fcell.2023.1156138)
Supplement: Supplementary file 1 [file DataSheet1.zip › Data Sheet 1/Table S3 The comparison of gene numbers.pdf]

Table S3. The comparison of gene numbers

Gene	Human	Mouse	*M. berezovskii*	*M.moschiferus*
Serpinb6	**2**	**5**	**6**	**4**
Safb1/2	**2**	**2**	**7**	**11**
Srd5a1	**1**	**1**	**3**	**2**
