# Supplementary material for: Integrated multi-omics analysis reveals insights into Chinese forest musk deer (Moschus berezovskii) genome evolution and musk synthesis
Source: Front Cell Dev Biol. 2023 May 9;11:1156138. doi: 10.3389/fcell.2023.1156138 (PMC10203155; doi:10.3389/fcell.2023.1156138)
Supplement: Supplementary file 1 [file DataSheet1.zip › Data Sheet 1/Table S4_2023_The comparison of muscles_RE_C.pdf]

gene name	log2FoldChange	p-value	p-value adj
MAP3K3	24.43107469	1.82E-26	3.25E-22
TDH	22.98974747	2.20E-25	1.96E-21
ENSMMSG00000024060	23.17708596	6.94E-25	4.13E-21
EML4	23.47935059	1.01E-24	4.50E-21
NEDD4L	24.53680217	2.34E-19	6.94E-16
FLNB	24.96656809	2.04E-19	6.94E-16
CADM1	23.74902192	3.28E-19	8.35E-16
TLE3	25.17640744	1.34E-18	2.99E-15
MICAL3	23.53995072	1.74E-18	3.44E-15
DENND4A	24.13063398	2.16E-18	3.85E-15
BSDC1	24.04364275	2.50E-18	4.04E-15
ITGB4	23.53746414	1.66E-17	2.47E-14
TCF7L2	23.50071697	2.48E-17	3.39E-14
ZNF385A	23.95058304	5.02E-17	6.39E-14
TCHH	20.63570639	1.01E-16	1.20E-13
PTPRE	20.85868111	1.53E-16	1.70E-13
CBLC	22.35867315	2.64E-16	2.77E-13
SLC23A1	19.69845206	5.08E-14	5.03E-11
KRT80	21.71858494	6.05E-14	5.68E-11
PLD1	20.26413613	1.52E-13	1.36E-10
ENSMMSG00000007596	20.96964523	9.73E-13	8.26E-10
ESYT2	12.74961042	7.85E-12	6.36E-09
ENSMMSG00000004558	-23.46809856	3.24E-11	2.51E-08
MYCBP2	28.14895972	1.69E-10	1.26E-07
GABPB2	21.33383468	2.79E-10	1.99E-07
ZFR2	20.76596971	4.21E-10	2.88E-07
RXRG	21.85729164	5.21E-10	3.32E-07
CC2D2A	21.73492814	5.19E-10	3.32E-07
CDK5RAP2	-26.59451962	8.02E-10	4.93E-07
CDK5RAP2	26.95012892	9.67E-10	5.75E-07
ENSMMSG00000012826	20.60563635	1.91E-09	1.07E-06
G36462	-25.98555901	1.92E-09	1.07E-06
RAPGEF2	26.38620109	2.14E-09	1.16E-06
DLG2	24.66034487	4.11E-09	2.16E-06
RYR3	20.99116187	4.27E-09	2.18E-06
HERC1	25.70640166	5.46E-09	2.70E-06
CUL9	25.29215731	9.55E-09	4.60E-06
CNTNAP2	21.20706967	1.41E-08	6.45E-06
SPTAN1	25.01130216	1.39E-08	6.45E-06
URGCP	24.96906357	1.47E-08	6.55E-06
PALS2	11.09564645	1.71E-08	7.43E-06
SLC5A11	20.7398041	2.53E-08	1.05E-05
CAST	-24.1098576	2.54E-08	1.05E-05
MYO18A	11.87781968	2.59E-08	1.05E-05
SUPT6H	24.35438844	3.28E-08	1.30E-05
TTN	17.9756535	3.90E-08	1.51E-05
WDFY3	24.05078006	4.85E-08	1.84E-05
SAMD4A	11.2672269	5.28E-08	1.96E-05
SLF1	23.84584784	6.30E-08	2.29E-05
ARHGEF11	23.75985048	7.02E-08	2.50E-05
G23955	-17.8641222	9.33E-08	3.26E-05
SLC5A3	11.19534049	9.70E-08	3.33E-05
CATHL4	-23.01056627	1.06E-07	3.57E-05
GOT1	23.31330621	1.23E-07	4.06E-05
REPS2	21.10222655	1.27E-07	4.12E-05
ATG16L1	20.63651207	1.39E-07	4.38E-05
ENSMMSG00000012869	21.16379733	1.40E-07	4.38E-05

SEMA4F	23.15772209	1.49E-07	4.58E-05
KCNH7	21.11369835	1.81E-07	5.38E-05
MTMR1	22.99855752	1.81E-07	5.38E-05
STAB1	22.96835668	1.88E-07	5.50E-05
PRPF40A	20.63790354	2.30E-07	6.62E-05
TTLL5	22.69575183	2.62E-07	7.42E-05
KRT5	10.1766925	2.99E-07	8.34E-05
L3MBTL1	21.71735713	3.21E-07	8.79E-05
ZNF568	22.5113452	3.27E-07	8.84E-05
KRT19	5.097285084	3.49E-07	9.28E-05
DNMBP	22.3847063	3.81E-07	1.00E-04
THOC5	22.11288085	4.73E-07	0.000122109
DENND1A	22.11779722	5.24E-07	0.000133405
SOCS7	22.05441698	5.65E-07	0.000140482
ACSM3	6.642621784	5.67E-07	0.000140482
STRA6	7.246098768	6.05E-07	0.000147689
NAV2	21.92395034	6.58E-07	0.000158613
MAP3K12	21.85096532	7.17E-07	0.000170523
AZGP1	5.740277686	1.60E-06	0.00037478
ENSMMSG00000007424	6.807714487	1.76E-06	0.00040755
ADCY4	21.02051749	1.87E-06	0.000426912
ENSMMSG00000010011	20.8667582	2.22E-06	0.000495049
RIPK4	10.03326148	2.22E-06	0.000495049
RNF222	8.727982748	2.30E-06	0.00050696
ESYT3	10.08460865	2.47E-06	0.000536856
G10873	20.74863304	2.54E-06	0.00053857
F11	20.75718184	2.51E-06	0.00053857
FNIP2	20.51930937	3.28E-06	0.000687522
ZFC3H1	12.41212445	3.72E-06	0.000770084
SP140L	10.08146101	4.49E-06	0.000910037
ABCC11	5.793621657	4.45E-06	0.000910037
EPN3	7.82761824	5.91E-06	0.001183298
FAR2	8.446352712	6.31E-06	0.001250548
IGSF3	-19.55401519	6.42E-06	0.001258397
NRCAM	19.88652372	6.51E-06	0.001261388
CUX2	10.11080664	7.38E-06	0.001414112
SNX31	7.905268073	7.55E-06	0.001431784
KMO	8.796737205	7.87E-06	0.001477607
CERS4	4.078585243	9.42E-06	0.001749685
RTTN	10.95613506	9.88E-06	0.001816107
USP34	12.33680236	1.01E-05	0.001829232
BPTF	-19.13102402	1.03E-05	0.0018592
SYNE1	10.82502782	1.07E-05	0.001915447
KRT7	5.868578933	1.16E-05	0.002047046
MBOAT2	4.44400479	1.23E-05	0.002151797
EVPL	6.045484461	1.36E-05	0.002349796
ADH1C	6.950494092	1.37E-05	0.002356736
PRKCB	8.48599206	1.58E-05	0.00267727
IL17RE	4.700336254	1.65E-05	0.002781399
CDC42BPA	11.35961843	1.69E-05	0.002808513
PGAP1	8.807783906	1.77E-05	0.002897567
CES3	9.201427801	1.77E-05	0.002897567
LRP2	5.835415268	2.07E-05	0.003362015
GALNT3	7.980131004	2.12E-05	0.003406091
IL37	18.7202226	2.22E-05	0.00352989
SRD5A1	4.490775837	2.26E-05	0.003564971
FNBP1	9.705279911	2.96E-05	0.004628268
DLG1	9.747209529	4.51E-05	0.006984305

ENSMMSG00000016516	6.212588307	4.60E-05	0.007068869
PKHD1	9.039013334	5.18E-05	0.007896246
MAP4K5	8.749005109	5.95E-05	0.008910329
LPO	6.095395635	5.92E-05	0.008910329
PROM2	5.023791264	6.89E-05	0.010242056
PSAT1	5.348156567	7.46E-05	0.010983769
RYR2	9.457969313	8.23E-05	0.012031973
KRT8	5.75827823	8.38E-05	0.012152022
CCDC159	4.969004273	8.65E-05	0.01243174
LOC534967	4.030072732	8.99E-05	0.012817157
GGT1	5.154167695	9.26E-05	0.01309869
FER1L5	7.137665269	9.44E-05	0.013243537
CMTM4	8.120469863	0.000105018	0.014625464
CXADR	8.543832956	0.000105922	0.014636889
G5323	9.600231455	0.000107041	0.01467775
CDC14A	8.92563002	0.000112392	0.015063843
ADGRV1	8.427471353	0.000111981	0.015063843
TJP3	4.65811679	0.000111189	0.015063843
ENSMMSG00000001475	7.797263242	0.000116375	0.015481405
SLC22A31	9.102358286	0.000120119	0.015861079
LAD1	5.995902305	0.000128064	0.016785758
LOC786350	-16.56851696	0.00012964	0.016868338
TMEM245	10.42333507	0.000131325	0.01696381
KRT10	5.748804382	0.000133013	0.01705826
FASN	4.656348782	0.000139366	0.017745252
PSPN	16.93459399	0.000141522	0.017887925
ENSMMSG00000016501	10.49086038	0.000142493	0.017887925
KRTDAP	-16.48456906	0.000146745	0.018292902
AFF3	8.017971174	0.000147978	0.018318489
ENSMMSG00000001053	-16.53803092	0.000151904	0.01867471
ARHGAP21	9.393239584	0.000156901	0.019156935
GRIN1	5.401024607	0.000162606	0.019718427
STXBP5	8.213948966	0.000168203	0.020221626
REEP6	4.183431534	0.000169592	0.020221626
KRT18	4.872599713	0.000170158	0.020221626
SLC36A2	16.68943869	0.000183446	0.021656372
KRT74/KRT71	-16.42826457	0.000184968	0.02169234
ENSMMSG00000003316	8.165507932	0.000198151	0.02308655
CFAP52	-15.33564772	0.000214603	0.024840982
ABCC3	7.884490337	0.000222317	0.025567917
KCNK2	8.340664929	0.000243346	0.027806907
FGFR2	7.226145135	0.000250974	0.028495928
SPTBN1	10.06374828	0.000257975	0.029077183
ELMO3	6.410286341	0.000259356	0.029077183
SAA4	-16.09924589	0.00026649	0.029690291
BAIAP2L1	5.744768546	0.000268671	0.029747388
LRRC43	8.537713178	0.000273612	0.030107496
CALML5	-15.75422775	0.000283036	0.030953399
MYO9A	8.925856234	0.000290299	0.031554033
G23224	7.156810981	0.000297921	0.032186272
ELF5	5.953759742	0.000303934	0.032442725
EPCAM	4.451502209	0.000302205	0.032442725
ADGRB3	9.773093126	0.000311178	0.032598795
DLG2	7.684671448	0.000312711	0.032598795
FNBP1	9.5920215	0.000312145	0.032598795
SFN	5.222688054	0.000308415	0.032598795
HSD17B2	4.813473105	0.000317389	0.032894064
IL4I1	7.805652229	0.000333506	0.034364637

ATP6V1G3	7.051790793	0.000347513	0.035602079
CRYBG2	5.209007696	0.000354082	0.036067838
FARP2	8.186469646	0.000370843	0.037560507
CIDEA	4.505221653	0.000374091	0.037675434
HCST	7.464170239	0.000377263	0.037781408
ABCC12	6.452408315	0.000406897	0.04052147
GGT5	3.622085053	0.00041174	0.040550672
MFSD4A	3.821645338	0.00041117	0.040550672
H2AC21	7.074217069	0.000416908	0.04083403
PCDH18	7.867779428	0.000424355	0.040889467
GALNT8	5.639679723	0.000423496	0.040889467
KRT17	8.048801087	0.000421727	0.040889467
ARHGAP8	8.064734399	0.000435458	0.041733765
TFAP2A	7.970046868	0.000444473	0.042369938
ENSBTAG00000045746	5.199587971	0.000452086	0.042866454
SS18	9.221784674	0.000463139	0.043452149
LRRC26	7.917277601	0.00046244	0.043452149
GOLIM4	7.482848777	0.000481889	0.044974578
SLC37A4	9.075698044	0.000485539	0.045079303
EHF	8.353610729	0.000514484	0.047519113
DIAPH3	7.033790054	0.000521843	0.047950358
ARHGEF11	5.287587356	0.000530228	0.048223699
CLMN	8.972034792	0.000529228	0.048223699
CAMK2D	8.88872023	0.000541953	0.049028747
FXYD3	5.594565908	0.000547246	0.049028747
EPS8L1	8.053520786	0.000550081	0.049028747
TRPV6	6.977305323	0.000548366	0.049028747
SH3D21	4.7019095	0.000558834	0.049561056
C23H6ORF132	3.762715477	0.000563035	0.049686446
ACE2	6.915053072	0.000566951	0.049785523
GPR176	8.579541327	0.000572415	0.05001894
ENSMMSG00000019635	9.821083265	0.000590943	0.051386092
GCA	8.217106401	0.000621447	0.053776295
LOC513210	8.583399613	0.000633156	0.054524788
TRPM1	3.734140018	0.000644975	0.055275627
CYSRT1	7.238951556	0.000653424	0.055731719
DSG2	7.587423914	0.000661769	0.056174706
RASEF	7.285222426	0.000680863	0.057521602
GINS1	7.295402564	0.000714073	0.058186231
ZNF677	8.283245638	0.000712906	0.058186231
NRK	7.95798485	0.000724635	0.058186231
S100A9	6.052988357	0.000706751	0.058186231
GPX2	4.573027697	0.000716578	0.058186231
KRT23	5.237433922	0.000719349	0.058186231
ASNS	2.615435275	0.000713475	0.058186231
SHANK2	7.791268998	0.000700356	0.058186231
DHCR24	3.594055065	0.000713386	0.058186231
RDH16	5.360439347	0.000724534	0.058186231
CDH1	3.568681991	0.000695264	0.058186231
VPS36	15.27692349	0.000750227	0.059971085
MTMR3	6.932691274	0.00078251	0.062272397
PLA2G7	3.593834222	0.000790388	0.062619845
KRT83	7.618940875	0.000813913	0.064198273
SLC30A3	8.018097993	0.000828363	0.064697707
ARR3	8.090185772	0.000831133	0.064697707
PLPPR2	4.202510614	0.000830586	0.064697707
PRR15L	7.033507447	0.000886542	0.068710848
KLF12	8.737462792	0.000916026	0.070081876

COL4A6	7.390006839	0.000914043	0.070081876
MAL2	7.299463865	0.000915733	0.070081876
WNT6	6.175648864	0.000934735	0.07043408
ZMAT3	7.607682859	0.000925861	0.07043408
SLC7A5	3.157361143	0.000933882	0.07043408
RCE1	3.722375837	0.000936434	0.07043408
EPS8L2	4.297779957	0.000941155	0.070491695
KLHDC9	6.642937819	0.000950238	0.070874216
GSDMA	8.243774783	0.000972009	0.072195941
ESRP1	7.156446151	0.000988237	0.07304699
ABHD3	4.767530876	0.000996955	0.07304699
AKR1A1	3.264410168	0.000995284	0.07304699
EPHX2	5.017827993	0.000999858	0.07304699
FAM83C	7.558887103	0.001025829	0.074638461
ARL14	-14.89768474	0.00103791	0.074906015
THRSP	5.106424296	0.001035642	0.074906015
DLK2	7.392330446	0.001054007	0.075406001
MGAT5B	6.937632184	0.001057528	0.075406001
GCGR	3.887121269	0.001057028	0.075406001
TNFRSF11A	7.286692526	0.001063392	0.075522011
SPOCK1	7.49648879	0.001074476	0.076006414
ITPR1	7.312469373	0.001095809	0.076603475
GABRB3	8.649528303	0.001090322	0.076603475
ENSBTAG00000000835	4.134979828	0.001092264	0.076603475
FAM25A	5.496117482	0.001125739	0.078388374
LY6D	-14.51618883	0.001130677	0.078425864
CREB3L4	4.342439119	0.001141667	0.07888124
DGAT2L6	4.165229823	0.001180938	0.081279545
TRIM29	6.506712349	0.001215312	0.082384719
KYNU	6.834206635	0.001205486	0.082384719
ACSL1	5.012482011	0.00120764	0.082384719
DSP	3.207809364	0.001215482	0.082384719
TBC1D23	8.714917656	0.001268984	0.085685252
ENSBTAG00000052139	7.925223639	0.001284044	0.086374996
MAST3	8.035577053	0.001301927	0.08724866
CLDN3	6.857431267	0.001311284	0.087546591
LMAN1L	6.890130044	0.001321904	0.087926342
PLS1	7.232813346	0.001354487	0.089147242
ASCL2	7.383375487	0.001355262	0.089147242
NUPR1	2.84808732	0.001351475	0.089147242
MOXD1	4.694584907	0.001362306	0.089281147
G8781	8.424798672	0.001368044	0.089328764
POLQ	8.563920503	0.001375085	0.08946079
MOV10	8.665607801	0.001408728	0.091006429
GABRB3	8.402890896	0.001409053	0.091006429
COL13A1	-14.49127349	0.001419968	0.091380323
PRELID2	7.403158918	0.001430736	0.091512129
ACBD7	4.770660202	0.001437417	0.091512129
KCNK1	7.299854548	0.001436981	0.091512129
DDAH1	8.214280616	0.001446248	0.091746691
ENSMMSG00000019393	9.21743896	0.001480716	0.093340417
NHSL1	9.362713646	0.001481843	0.093340417
HIST1H2AP	8.12880919	0.001502327	0.094297458
IL20RA	7.579119549	0.001516149	0.094666243
FLVCR2	4.700545548	0.001518823	0.094666243
KRT14	6.373221261	0.001539682	0.095631971
SEC14L4	4.211956328	0.001545159	0.095638922
RIMS1	8.518477069	0.001559721	0.096206151

CST6	4.338172543	0.001574372	0.096774995
GRB7	5.176782862	0.001581823	0.096853622
CYP4F21	7.752233231	0.001586517	0.096853622
GXYLT2	6.526675501	0.001621313	0.098304507
ACOT7	4.127245315	0.001618227	0.098304507
ACER3	9.160143851	0.00164291	0.098703342
OGFRL1	8.761599011	0.001634965	0.098703342
PBX3	-14.29735504	0.001644502	0.098703342
RBMS3	8.159224256	0.001695533	0.101424754
SUSD2	5.369826785	0.001736149	0.103507005
STEAP2	7.301423522	0.001769259	0.105129378
RPL7A	9.010086898	0.001786008	0.105421804
NKPD1	4.47886916	0.001784441	0.105421804
495184/LOC101732638/LO(	6.977476693	0.001796887	0.105713874
RFX2	-14.22974209	0.001805225	0.105855061
IL18	5.451734771	0.001845807	0.107879844
STYK1	7.960285769	0.001886311	0.109886841
PIWIL2	8.059315594	0.001927707	0.111932593
PTPN6	7.843687465	0.001950167	0.112869101
DEGS2	3.964866501	0.002035218	0.117410341
ENO4	6.66253663	0.00205005	0.117884487
DAB2IP	9.156229872	0.002060956	0.118130564
GSTA3	6.27685178	0.002094677	0.119678578
TPD52L1	6.694907631	0.002115286	0.120469905
RPS20	8.590511427	0.002135082	0.121210119
G10398	8.760379591	0.002155605	0.12162929
DNASE1	3.250654934	0.002156112	0.12162929
CRYBA1	7.822720812	0.00216708	0.121862332
DNM1	8.149301987	0.002189986	0.12276317
CXCR5	7.994870252	0.002204207	0.122788105
GALNT6	3.829437962	0.002197552	0.122788105
KLC3	4.637911972	0.002216951	0.123113313
FYB1	7.239792637	0.002223858	0.123113316
ENSBTAG00000052865	8.51484824	0.002248204	0.123336079
LOC104972276	6.056521829	0.002248638	0.123336079
PRODH	3.171670619	0.002239044	0.123336079
CTRC	6.172225922	0.002338273	0.127388899
LSR	3.945182646	0.002350996	0.127388899
ARHGEF5	6.620579337	0.002337583	0.127388899
LOC507550	5.668198943	0.002351113	0.127388899
TEAD1	9.459006558	0.002388758	0.128147803
PRKAG2	6.285205719	0.002380317	0.128147803
ATP13A5	3.951710912	0.002393875	0.128147803
BCAT2	3.274223834	0.002376296	0.128147803
CD2	6.06810101	0.002423073	0.128552669
GARNL3	6.986302298	0.002420359	0.128552669
LRP2BP	4.020868747	0.002419093	0.128552669
EPHB6	6.416470204	0.002438622	0.12899368
ZNF296	4.24510273	0.002484854	0.1310503
HAUS8	2.858050675	0.002496025	0.131251165
ABCC3	3.075025041	0.00253619	0.13297094
PAG1	8.817710341	0.002597131	0.135766718
ZNF789	7.243066657	0.002623631	0.136751025
IRX3	3.681058928	0.002635485	0.136827344
GLYATL2	4.042469469	0.002640447	0.136827344
IRX5	4.544351571	0.002655883	0.137228325
ATF7IP2	6.452999893	0.002727216	0.1405068
FAM83H	3.395354161	0.002742339	0.140878763

AMZ2	5.395675657	0.002790425	0.142937113
CGN	3.555917624	0.00285917	0.14603888
H2BC3	7.981276935	0.002918768	0.148657049
LOC526524	8.981214956	0.002996172	0.15109412
SPECC1	7.766743229	0.002988082	0.15109412
LRRC8E	5.930545115	0.003000523	0.15109412
PKP1	6.061489075	0.00299986	0.15109412
XDH	7.926178373	0.003011982	0.151243912
THSD7A	7.889452023	0.003064775	0.153462581
G33412	8.891604714	0.003099365	0.154547597
ACHE	7.602596672	0.003103783	0.154547597
SERPINA1	6.581984261	0.003201643	0.158095546
RASSF3	7.455808238	0.003188945	0.158095546
ATP13A4	6.704137418	0.00319636	0.158095546
MYO5B	2.902160408	0.003243787	0.159734104
ESPN	4.553512762	0.003259729	0.16007694
S100A8	6.942104274	0.003381016	0.165123272
EFNA3	6.683613316	0.003378539	0.165123272
DLEC1	4.527148048	0.003391838	0.165199177
IGFBP2	5.78012019	0.003475586	0.168816902
ZNF331	6.670564578	0.003496001	0.169147399
ADAMTSL2	3.89760254	0.003501368	0.169147399
SLC2A10	3.666867808	0.00354889	0.170624286
EFS	3.019835592	0.003551083	0.170624286
G34686	6.766765268	0.003594274	0.171773544
NAV2	8.818891967	0.003590634	0.171773544
GPLD1	7.183220878	0.003654696	0.174031484
ENSMMSG00000003905	7.104675407	0.003661046	0.174031484
CTNNA3	7.568985168	0.00370891	0.175371445
SCUBE3	8.522852327	0.003705283	0.175371445
CFAP251	7.461300027	0.003735057	0.175406806
FGG	7.624420977	0.003738403	0.175406806
ENSMMSG00000020761	8.657740651	0.003749018	0.175406806
SIPA1L1	5.2826695	0.003748565	0.175406806
STXBP2	3.430068464	0.003772881	0.176061208
RNF208	4.729792435	0.003807001	0.176269099
ENSMMSG00000021931	6.321865536	0.003806185	0.176269099
RAP1GAP	3.146919693	0.003792556	0.176269099
FAM131C	6.915499921	0.003895003	0.179876485
RPL23A	7.935644926	0.003908551	0.18003575
DSC2	6.738730829	0.003967473	0.182278811
ARHGEF16	3.757482115	0.003987968	0.182749424
RHBDL1	3.045583645	0.004046279	0.18471339
NAPRT	3.243469512	0.00405155	0.18471339
ENO1	4.590280483	0.004139956	0.188262385
APC	8.739607491	0.004187556	0.189942428
GSDMB	2.963457108	0.004204888	0.190244523
CLDN7	3.028258367	0.00427408	0.192885446
SPTBN2	4.051243496	0.00429344	0.193269834
SDF2L1	3.511099228	0.004326755	0.194278923
HAL	6.933770778	0.004345211	0.19440073
CCDC113	6.359447199	0.00435913	0.19440073
IQGAP2	3.340577233	0.004362184	0.19440073
PTGDS	3.843285742	0.004424594	0.196690294
ODF3B	6.484358524	0.004459658	0.197483852
ST14	3.574588174	0.004464602	0.197483852
CRACR2A	4.802940345	0.004495779	0.198370706
C11H2ORF68	6.976787255	0.004620176	0.201252998

G8963	5.812874385	0.004628842	0.201252998
DNPH1	8.508894105	0.004613776	0.201252998
FAM221A	7.269126437	0.004613722	0.201252998
HMGB3	6.126573287	0.004600411	0.201252998
GSTA1	2.797497383	0.004588275	0.201252998
ENSMMSG00000012191	3.100646096	0.004643092	0.2013814
DAB2IP	6.681919073	0.004657476	0.201514971
GJB1	5.745482363	0.004710465	0.203314143
SMPD3	2.924426854	0.00473274	0.203782191
NEBL	6.990823576	0.004803539	0.206332276
ENSMMSG00000006719	7.612921151	0.004839178	0.206371272
PCOLCE	6.047512537	0.004816126	0.206371272
RASEF	6.421160222	0.004833789	0.206371272
STXBP5L	9.247598687	0.004860816	0.206799295
OPN4	7.613441831	0.004913177	0.20803393
TNNT2	4.968993763	0.004905179	0.20803393
TMEM184A	3.887403132	0.004930051	0.208253751
CEACAM19	7.052664066	0.005065163	0.213455317
ZNF135	6.390811097	0.005128103	0.214585829
COCH	6.777207844	0.005116247	0.214585829
GRHL2	6.421554503	0.005106662	0.214585829
ESRP2	3.201908307	0.005158541	0.21535399
DCLRE1C	2.897266539	0.005184958	0.215951073
MOCOS	3.325919553	0.005250405	0.21816718
ATP6V1B1	5.960108787	0.005373843	0.222260132
TNK1	3.308981496	0.005370067	0.222260132
CACNA1C	7.282451455	0.005466684	0.225576653
ENSMMSG00000020416	12.22993248	0.005523154	0.227380455
PCDH11X	7.467092401	0.005538332	0.227479983
SLFN11	7.162405179	0.005646629	0.231394955
FUT2	6.559757253	0.005675805	0.231408601
RAB3C	7.028534012	0.005685906	0.231408601
SORL1	2.728145127	0.005666605	0.231408601
LOC509911	7.819070081	0.005743416	0.23321669
ENSBTAG00000003528	6.717067402	0.005833069	0.235782959
RYR3	11.89858526	0.005830493	0.235782959
TNFSF8	9.014698723	0.005876514	0.235934105
GPLD1	6.906633841	0.005853475	0.235934105
ARHGEF38	6.675616497	0.005865037	0.235934105
ENSBTAG00000034391	6.248184787	0.00592065	0.236770972
ENSMMSG00000020461	8.0657819	0.005955646	0.236770972
DUOX2	6.833126854	0.005990335	0.236770972
FZD10	4.534355445	0.005985169	0.236770972
SLC9A7	6.007555254	0.005955017	0.236770972
CLDN7	3.14709764	0.005952086	0.236770972
STAP2	3.650354481	0.005965818	0.236770972
QRICH2	6.385101233	0.00612553	0.240514771
SH2D4A	5.942965925	0.00610419	0.240514771
GABRR2	5.355071869	0.006116096	0.240514771
MAGI2	6.205628952	0.006154916	0.241137447
LOC783604	2.91983451	0.006198687	0.242319713
IRF6	4.077934408	0.006234404	0.243182682
MLANA	7.387366514	0.006313039	0.243456267
SLC10A6	6.231964395	0.006292692	0.243456267
FSD1L	6.889587173	0.006323362	0.243456267
KHK	5.958545296	0.006315989	0.243456267
GALNT7	4.468497589	0.006304201	0.243456267
SRCIN1	4.071850458	0.006293447	0.243456267

MAPK8IP2	4.082337331	0.006339896	0.24356677
SLC6A9	3.311725453	0.006383705	0.244722436
SLC6A2	5.041669183	0.006437628	0.246260002
SCUBE1	4.074543112	0.006510856	0.248527867
RND2	4.245251286	0.006543831	0.249252826
IFI6	3.971070617	0.006578072	0.250022823
LOC527744	6.454170737	0.006625319	0.250946682
MDFI	5.226355468	0.006630533	0.250946682
DGKZ	6.318592103	0.006662947	0.251639164
CENPE	6.165164452	0.006725442	0.253462427
LOC508879	3.541739213	0.006802395	0.255821722
SHTN1	4.309290131	0.006821205	0.255989044
NF1	11.48698555	0.006892242	0.257570461
ACSL5	3.362359019	0.006886813	0.257570461
RHOV	4.800005025	0.006955111	0.259376167
G25813	6.102930474	0.007021058	0.260744542
DNAAF3/TNNI3	3.276930003	0.007019152	0.260744542
ENSMMSG00000011038	4.457048304	0.007102398	0.262670843
ALOXE3	6.728117966	0.00709504	0.262670843
CENPP	5.787078495	0.007172943	0.264257011
ENSMMSG00000007447	6.838358585	0.007229737	0.264257011
FSIP1	6.895630271	0.007191075	0.264257011
LOC104968411	4.083129755	0.007207665	0.264257011
TLDC2	5.769769054	0.007205745	0.264257011
MROH6	3.86110382	0.007244171	0.264257011
ANO4	3.894908798	0.007249056	0.264257011
TMEM102	2.938001422	0.007313024	0.266044813
DYNC1I2	5.383774591	0.007339904	0.26647887
KCNK7	4.064008543	0.007355492	0.266502049
SERTAD4	6.001173722	0.007430683	0.267835137
CDS1	6.067438068	0.007418325	0.267835137
CELSR1	3.389779764	0.007437361	0.267835137
CABCOCO1	6.978244313	0.007521122	0.269773966
PCSK4	4.356772905	0.007521466	0.269773966
RNF180	7.00487842	0.007578158	0.271261533
UBXN10	6.34267262	0.007652215	0.273363488
TRARG1	7.784070058	0.007751116	0.276342796
SCNN1A	4.764992839	0.007783136	0.276930488
RBMS3	7.230677463	0.007812753	0.277430541
LARGE2	4.731865072	0.007840194	0.277851493
SDC1	3.215871171	0.007894375	0.279216513
CFAP69	5.594556324	0.00801419	0.282892983
MALT1	7.615257221	0.008156915	0.28562466
OTOP3	6.102943089	0.008209099	0.28562466
G34733	5.90771319	0.00819621	0.28562466
NOX5	6.185105093	0.008153435	0.28562466
ADGRF3	5.911474254	0.008197233	0.28562466
SBK2	11.66348286	0.008138187	0.28562466
TMEM45B	3.370872739	0.008188795	0.28562466
CDCP1	5.439439316	0.00821976	0.28562466
G5403	7.041257954	0.008255837	0.285673805
IMPG2	7.607053581	0.008276479	0.285673805
KALRN	11.64266897	0.008252544	0.285673805
ARRDC5	3.39672517	0.008285278	0.285673805
GCLC	3.112930866	0.008355625	0.287543179
ENSBTAG00000004061	7.290389386	0.008418473	0.289001841
G11978	6.405436758	0.008446649	0.289001841
SPDEF	5.236921058	0.008444559	0.289001841

COL7A1	2.687520356	0.008468714	0.289201711
ZFPM2	7.976319491	0.008504556	0.289870383
WWC1	3.952078235	0.008525193	0.290019248
SLC25A21	6.382349492	0.008574224	0.291131645
PALM3	2.892303254	0.008590771	0.291138928
SSPO	5.871081441	0.008637256	0.292158873
MTUS2	5.96657333	0.008724583	0.293997014
SORBS2	4.779527066	0.008723533	0.293997014
MYH10	7.465966688	0.008749914	0.294294289
OSBPL3	3.198455241	0.008814947	0.295923246
TNS4	5.620477577	0.008834162	0.296010843
PAPPA	6.096432526	0.008865307	0.296497099
SF3A3	7.993600415	0.008928038	0.296672214
KCTD14	5.348083276	0.008921802	0.296672214
ELOVL6	4.64061792	0.008937113	0.296672214
CYB561	3.470340151	0.008930908	0.296672214
ALKBH6	3.497285497	0.009073873	0.300652156
ARG1	6.088947657	0.009209127	0.301768195
G27922	7.52175671	0.00916952	0.301768195
HBQ1	5.850088113	0.009204451	0.301768195
GJB2	5.512382504	0.009197197	0.301768195
SLC52A3	5.683093607	0.00915902	0.301768195
RBM47	4.060072322	0.009152445	0.301768195
DSC3	5.774326103	0.009278583	0.303486264
PTPN7	6.039446751	0.009380868	0.304354426
TLE6	5.213301842	0.00936271	0.304354426
WDR17	3.825934697	0.009332114	0.304354426
KCTD1	3.183589624	0.009382395	0.304354426
IL17RC	2.408019589	0.009390493	0.304354426
TTC12	3.629880026	0.009438023	0.305070153
DLG3	3.317192204	0.009446804	0.305070153
MEGF11	6.434101281	0.00951385	0.306679721
MCF2L2	6.613263418	0.009566243	0.307407716
PRSS27	6.253327127	0.009570923	0.307407716
MLPH	6.239946803	0.009604276	0.307924136
BAIAP2L2	5.997801382	0.009649825	0.308829046
CLIC3	4.037101158	0.009674958	0.309078491
TMEM254	6.008536567	0.009738379	0.310548022
GUCY2D	6.992812505	0.009797426	0.311873049
MAPK4	5.111028521	0.009825842	0.311973135
ENPP5	4.446214266	0.009835572	0.311973135
DENND1C	3.320052235	0.009928022	0.314346228
ENSMMSG00000000727	6.545965913	0.009986393	0.315633764
A2ML1	6.387566837	0.01004084	0.31673029
SHANK2	4.979175039	0.010056622	0.31673029
KLB	7.659646444	0.010075302	0.316758961
FOXA1	5.439015596	0.010113721	0.317407033
CD86	6.145952067	0.010192244	0.318749016
DYNC2H1	6.071435928	0.010179297	0.318749016
SLC5A3	6.695222345	0.010241187	0.31971874
UGCG	5.397507224	0.010268095	0.319998353
CFAP43	5.477559412	0.010429764	0.322850463
SOWAHC	4.668131928	0.010411511	0.322850463
H2AJ	2.781752088	0.010432058	0.322850463
S100A2	3.786382	0.010400242	0.322850463
BARX2	3.8238969	0.010473951	0.323025361
SESN3	3.593552443	0.010460553	0.323025361
G38	3.196999313	0.010513208	0.323676069

TENM1	6.188407223	0.01060921	0.325531298
DENND2D	2.624426729	0.01060999	0.325531298
PI15	5.785159759	0.010671891	0.326867934
SREBF1	3.70847995	0.010700912	0.3271946
G28932	7.62034466	0.010830437	0.33019721
ENSBTAG00000034921	7.622687827	0.010836159	0.33019721
PTPRE	3.529497994	0.010858091	0.330300891
G3545	7.863815586	0.01101694	0.332297742
FST	7.040162549	0.011013829	0.332297742
STK19	3.23874548	0.010964906	0.332297742
FUT1	5.407088711	0.010989819	0.332297742
TMPRSS13	5.682029814	0.010958804	0.332297742
RDM1	5.774249028	0.011067292	0.333252599
G5614	7.76796344	0.011165702	0.33496743
SLC11A2	6.728048613	0.011151062	0.33496743
FAM184B	-10.97702868	0.011210928	0.33496743
SLIT1	7.07903538	0.011211888	0.33496743
SERPINB8	3.120152734	0.011218196	0.33496743
AGR2	6.500145732	0.011341321	0.335831198
G1218	7.682573976	0.011335311	0.335831198
NR6A1	7.552030796	0.011322912	0.335831198
EPB41L4B	3.87241919	0.011275713	0.335831198
SHROOM3	3.910882527	0.011289285	0.335831198
ENSBTAG00000046857	5.512383264	0.011376161	0.33630423
PLEKHH2	6.882601428	0.011449542	0.336824385
ZNF831	5.875365087	0.011450442	0.336824385
CCND1	2.774675551	0.011445337	0.336824385
ACP3	6.901808086	0.011489928	0.337429081
CA8	5.763799258	0.011666838	0.34206094
SUGCT	6.061007645	0.011761211	0.344261663
G27253	6.189302227	0.011874456	0.34700663
IL17RC	5.387561197	0.011918721	0.347730152
LOC514978	6.004949485	0.012138239	0.351853762
FMO5	6.650288191	0.012100014	0.351853762
RPL12	7.601142407	0.012157117	0.351853762
FAT1	5.903008056	0.012141414	0.351853762
PHGDH	3.533794533	0.012158752	0.351853762
CREB3L1	3.342111304	0.012198475	0.352431138
LRRC72	8.23994152	0.01225384	0.353457849
GAS7	6.783586936	0.012433547	0.355763094
GABRB1	7.942043814	0.012384583	0.355763094
LGI2	4.695099873	0.012404231	0.355763094
TTYH3	6.864773874	0.01242234	0.355763094
ACSBG1	7.171312694	0.012424732	0.355763094
GALE	2.292694436	0.012461954	0.356004474
NPAS3	7.607532111	0.012530572	0.356475822
CA12	6.739026019	0.012517644	0.356475822
ENSBTAG00000037882	3.62693754	0.012538446	0.356475822
KMT2D	6.675728571	0.012623041	0.357171955
CES1	3.235971581	0.012591891	0.357171955
CCDC125	2.405612718	0.012617723	0.357171955
ACOX2	3.51705931	0.012659315	0.357630662
G2304	7.164845076	0.012808496	0.359720719
ENSMMSG00000011755	7.309617589	0.012816057	0.359720719
SH3PXD2A	10.96750987	0.012834196	0.359720719
DSG2	5.792786632	0.012774588	0.359720719
LOC538060	3.103800336	0.012786946	0.359720719
POU2F3	5.167880795	0.012920357	0.361567167

CPSF7	8.409563907	0.012993822	0.362216887
TMEM229B	5.642325417	0.013004533	0.362216887
ASIC5	5.829677806	0.012977363	0.362216887
CSTB	3.19962167	0.013073566	0.363571575
CRELD2	2.662275424	0.013124468	0.364418641
ENSMMSG00000022209	5.572534355	0.013214596	0.366350529
CILP2	-9.256428056	0.013282193	0.36765274
HSD17B14	3.943807815	0.013314489	0.367975319
CERT1	4.364651436	0.013484789	0.372105032
FA2H	6.6919133	0.01351175	0.372272723
CXCL9	5.229935998	0.013546156	0.37264473
UCMA	6.350358483	0.013702828	0.375217517
ENSBTAG00000050361	6.700139229	0.013679113	0.375217517
RAB25	4.865209809	0.013686326	0.375217517
DTNB	3.352376385	0.013811284	0.377607276
SIDT1	3.918556603	0.013846799	0.377998527
LSS	3.239024396	0.01393115	0.37971969
PERP	2.83127882	0.013998856	0.380982603
ENSMMSG00000017509	7.721048185	0.014057373	0.381991964
MYO6	5.120527005	0.014088685	0.382260116
PKLR	5.480810254	0.014355453	0.388478978
SPINK5	6.907172639	0.01440506	0.388478978
MATK	4.795904782	0.014393679	0.388478978
HP	3.642002295	0.01437116	0.388478978
KIF19	6.943076253	0.014470374	0.389063174
ENSBTAG00000038755	7.069515761	0.014468353	0.389063174
AOX2	5.617134692	0.014646021	0.393192724
PLCB1	6.116206852	0.014690227	0.393786459
S100A12	3.405112954	0.014738494	0.394487071
ALOX12E	6.452772184	0.014885854	0.396052591
TRPM5	5.450453848	0.014873933	0.396052591
C15H11ORF52	4.096493194	0.014871743	0.396052591
ENTPD3	4.228306869	0.014828763	0.396052591
CENPP	5.488026321	0.014943819	0.397002272
G28354	6.337693751	0.014988026	0.397584153
SSU72	6.210060456	0.015084609	0.399551615
ZSWIM5	6.946713924	0.0151439	0.400526951
BRCA2	6.158733241	0.015216825	0.400672269
ADTRP	6.055572972	0.015205782	0.400672269
TRPS1	2.923703186	0.015192904	0.400672269
LCP1	3.061342438	0.015262181	0.400682834
ERBB3	3.02230873	0.015244298	0.400682834
G7842	6.935182385	0.015497294	0.405921435
HSPA4L	3.172400065	0.015507265	0.405921435
FAM228A	5.940984633	0.015646117	0.408955534
CD7	5.210976634	0.015748558	0.410468723
BNIPL	5.044283927	0.015770232	0.410468723
NEK10	5.786822032	0.015796115	0.410468723
HOOK1	3.788940708	0.015786473	0.410468723
PPP2R2B	4.306735994	0.01595071	0.413882621
TPSB2	5.711790431	0.015997972	0.414505591
UBAP1	6.740840966	0.016209728	0.419382605
GGT6	5.84318813	0.016251592	0.419856348
CRTC1	7.253887091	0.016294007	0.420342947
MGST1	3.195067197	0.016425025	0.423110552
TMEM262	5.699777157	0.016571191	0.424422486
KLK5	6.425289585	0.016506095	0.424422486
IGSF5	6.390534143	0.016565216	0.424422486

RNPEP	2.146964131	0.016568149	0.424422486
G17325	7.261263633	0.016771481	0.42777566
ENSBTAG00000018669	2.88750687	0.016798102	0.42777566
SLC12A6	3.866214047	0.016729774	0.42777566
RENBP	2.649325435	0.016792132	0.42777566
TM7SF2	3.427612879	0.017072432	0.434058911
GRIK4	5.399680564	0.017093535	0.434058911
TEX49	5.475161847	0.017169309	0.435362882
G8561	8.325460817	0.017247406	0.435656693
SGIP1	6.428578298	0.017268733	0.435656693
CELA1	6.710462471	0.017278654	0.435656693
DNAH17	10.49881539	0.017220687	0.435656693
OLFML2A	-7.355423862	0.017328606	0.435683686
ICA1	3.515346556	0.017313088	0.435683686
CTNNA2	6.551814414	0.017393311	0.436063923
ROBO2	3.744308901	0.017409248	0.436063923
STXBP2	5.618643244	0.017417116	0.436063923
PROCA1	6.791162137	0.017578338	0.439483099
SLC1A3	7.472362353	0.017630792	0.440177175
GIGYF1	5.739416874	0.017675334	0.440672037
PTPRF	2.9971405	0.017710883	0.440941616
EFNA5	3.911588321	0.017772487	0.441858237
CLDN8	4.289899238	0.017914608	0.444771319
ASAH2	5.747428829	0.018001	0.446294621
JPT1	3.167093261	0.018081653	0.447671592
DOCK1	7.093141972	0.018114748	0.447868917
MAP4K4	5.278257278	0.018176454	0.448772114
CD274	7.273465991	0.018224497	0.449335931
FDXR	2.368669623	0.018262378	0.449647986
CRABP2	2.688955222	0.018305034	0.450076613
ENSMMSG00000001395	5.770719513	0.018370575	0.451065925
ENSMMSG00000016955	6.170237681	0.018439007	0.451849025
LOC520016	7.29208206	0.018453163	0.451849025
HS3ST3A1	6.607096851	0.018547213	0.453528963
ENSMMSG00000018383	7.155325406	0.018696526	0.456553801
GLYAT	6.427567449	0.018772304	0.45777715
GABRB3	6.270845915	0.018837994	0.458751487
ZNF81	7.319368566	0.018921462	0.460155492
FAT3	7.34085921	0.019150097	0.462560474
SH3TC2	10.33320684	0.019059116	0.462560474
G7318	3.335805742	0.019132237	0.462560474
SGPP2	4.719204933	0.019105648	0.462560474
ALDH3A2	2.53992933	0.01909106	0.462560474
IMPG2	5.909356142	0.019224316	0.463724852
RHBG	5.189472169	0.01925611	0.463864087
MCTP1	6.372486643	0.019318007	0.464727126
ELOVL7	4.387066477	0.019352702	0.464750454
DBI	2.777730253	0.01937112	0.464750454
RNF180	7.03198972	0.019471459	0.466529884
IL1R2	4.95886485	0.019717514	0.471158718
ACLY	2.218252779	0.019692047	0.471158718
G27165	6.217129367	0.019803604	0.471950593
NFE2L3	3.830117485	0.019803241	0.471950593
G15068	7.275566407	0.020007548	0.4749062
G36688	6.918478586	0.019956142	0.4749062
GLDN	7.001305663	0.01998406	0.4749062
GTF2IRD1	7.260041935	0.020064656	0.474996763
TC2N	3.270715716	0.020051963	0.474996763

ENSMMSG00000012883	6.183333519	0.020107629	0.475382758
RORB	6.029500461	0.020214751	0.47670635
CLSTN3	3.469689853	0.020217099	0.47670635
TRIM14	3.112383265	0.02032445	0.478604544
LRMDA	5.392263622	0.020363962	0.478902366
LAMB3	3.149775012	0.020413977	0.479446048
FGD2	3.856690159	0.020642282	0.48353392
LPCAT2	2.195203647	0.020626781	0.48353392
MYO5A	6.870507895	0.020738708	0.485155139
DNAAF1	3.58334825	0.02087714	0.487753476
DBNDD1	4.851398715	0.020946017	0.488232482
ENSMMSG00000017862	5.654881183	0.020972769	0.488232482
ENSBTAG00000049339	3.744179948	0.020979809	0.488232482
G13126	7.148037568	0.021007204	0.488232625
PCDHB5	5.731266268	0.021053773	0.488677803
KCNN2	6.229195495	0.021276673	0.49289267
PLEKHS1	6.271583952	0.021290663	0.49289267
ITGB8	6.012913802	0.021620364	0.498755104
SLCO2A1	2.374383368	0.021655809	0.498755104
S100A14	2.678662269	0.021648964	0.498755104
EPPK1	3.421385491	0.021644217	0.498755104
RASSF10	4.692726584	0.021720773	0.499605794
CAPG	3.241589492	0.021920956	0.50356051
MMP23	3.191919139	0.022005273	0.504846833
TMEM40	3.41098987	0.022103236	0.506442531
FAM166B	6.376832576	0.022253867	0.506549699
RBM46	5.131061674	0.022255865	0.506549699
CTBP2	6.03580501	0.022213538	0.506549699
CHCHD6	4.516877773	0.022221715	0.506549699
LOC536148	3.132888446	0.022278412	0.506549699
GATM	3.637716206	0.022154444	0.506549699
EPHA1	3.940130463	0.022466957	0.510185952
BOLA-DOB	5.179828533	0.02252035	0.510378625
PGRMC1	2.733258988	0.022532704	0.510378625
CADM2	8.039080116	0.022566113	0.510486719
AIG1	2.967225527	0.022627301	0.511222142
CYP27B1	5.989660068	0.02269462	0.512094039
MIF	2.618553598	0.022738324	0.512431569
TP53I13	3.521618761	0.022885509	0.514794229
STEAP4	2.488735999	0.022900921	0.514794229
SH3PXD2A	7.115532728	0.023029764	0.517038496
MYO7A	6.059807639	0.023140798	0.518877828
STAM2	4.504106055	0.023260603	0.520908938
EEF1A1	5.610084998	0.023425728	0.523292019
FAM107B	3.831111363	0.023423173	0.523292019
SPTLC2	1.901722764	0.023480803	0.523865818
TMEM232	5.838160533	0.023603588	0.525946945
FANCB	6.410654616	0.023747024	0.528482454
LOC511161	6.272244154	0.02386366	0.530415957
ENSMMSG00000001219	6.069133592	0.023906404	0.530538116
ENSMMSG00000012069	3.459154813	0.02392868	0.530538116
FIGNL2	5.233678317	0.024141146	0.534204634
PLA2G4B	1.993086644	0.024153985	0.534204634
CDC42BPG	2.930796393	0.024240058	0.535443962
DNAI7	6.243947169	0.024273344	0.535515627
SLC5A10	3.373752493	0.024319455	0.535869714
COL20A1	6.986350549	0.024534247	0.538605271
ENSBTAG00000055197	5.111176333	0.024513528	0.538605271

CAPS	2.228909392	0.024519505	0.538605271
ST6GAL1	3.601021617	0.024607635	0.539551916
FNDC10	2.537369333	0.024712995	0.540576598
IQGAP2	3.948430299	0.024715019	0.540576598
LOC112441540	5.909767922	0.024831783	0.542464898
VPS13C	6.020655059	0.025105584	0.547105297
IKZF3	4.77429713	0.025091866	0.547105297
ACOT4	3.705238778	0.025177306	0.547330072
RAB15	2.871362862	0.02515	0.547330072
LAMC3	5.731242892	0.025276811	0.548823906
ENSMMSG00000002670	5.742583395	0.025391529	0.550644028
LOC281376	3.01362172	0.025490339	0.551445117
SEMA4A	3.273555209	0.025473673	0.551445117
RUNX2	6.313473578	0.025607233	0.553302457
SLC2A1	3.24662004	0.025662482	0.553824935
NRN1	3.472428321	0.025869239	0.556938467
ENSMMSG00000005233	3.012395371	0.025838862	0.556938467
RPL27	3.45920826	0.025946247	0.557658896
PXMP4	2.586034624	0.025965269	0.557658896
DOCK10	6.084556317	0.026074164	0.558880238
TMEM144	5.891989793	0.026115837	0.558880238
PIP5K1B	3.087031957	0.026116192	0.558880238
TCAF2	2.481557753	0.026199981	0.560001039
ASPRV1	6.176134445	0.026321577	0.561926259
HHAT	5.104951079	0.026525889	0.565610632
SNORC	6.186787183	0.026590811	0.565641769
ENSMMSG00000005111	4.681673563	0.026559952	0.565641769
MAGI2	6.501571925	0.026653962	0.565751618
G28044	9.769739432	0.02665945	0.565751618
GATA3	2.386836535	0.026834753	0.568794653
G621	7.790650413	0.026954814	0.570660938
ORC1	5.594373231	0.027030436	0.571211344
DERL3	2.201179188	0.027044899	0.571211344
HSD3B1	5.572646147	0.027170673	0.5719715
EEF2KMT	3.143949111	0.027177149	0.5719715
CLDN4	3.516790277	0.027126185	0.5719715
SPNS3	4.140488532	0.027288325	0.572958405
SLC22A15	5.657376692	0.027279729	0.572958405
AVP	7.025587497	0.027514507	0.574267899
ENSMMSG00000023935	4.902227177	0.027576199	0.574267899
SLC1A1	4.916446485	0.02751278	0.574267899
EVA1A	5.292711397	0.027499207	0.574267899
SH3BP1	2.303515567	0.027566818	0.574267899
CBX4	2.056807735	0.02738869	0.574267899
FAM110A	2.648037716	0.027538297	0.574267899
LOC616957	6.651684796	0.027700457	0.57618243
PRRG3	3.94436996	0.027837019	0.577674855
TLCD1	2.270451007	0.02783104	0.577674855
SCNN1B	5.498854147	0.027870994	0.577707364
G31195	3.526092631	0.028079072	0.579325848
PAWR	3.020377082	0.0280742	0.579325848
SYT5	2.447481191	0.028053285	0.579325848
CRYBG1	2.481683871	0.028040828	0.579325848
STK32A	6.615978922	0.028181766	0.579934943
SGIP1	5.915949856	0.028173688	0.579934943
NEBL	2.263052783	0.028271259	0.579934943
PARP8	2.810794793	0.028256977	0.579934943
TRPM1	4.732908221	0.028246591	0.579934943

EPSTI1	5.998020291	0.028342785	0.580733885
NSDHL	2.138685008	0.028385734	0.58094615
DKKL1	3.313360842	0.028443046	0.581451532
G8349	9.644156837	0.028672603	0.583974007
WFIKKN1	3.865508001	0.028697477	0.583974007
TCF19	3.427869333	0.028684047	0.583974007
ARHGEF37	3.238492476	0.028625723	0.583974007
SPSB2	2.936054605	0.028746559	0.584122629
ENSBTAG00000048049	2.808169448	0.028770317	0.584122629
G26306	5.841728593	0.028947771	0.587056835
ATP6V1C2	2.849292451	0.028980815	0.587059094
DDAH1	6.996626035	0.029138186	0.589138397
TPBG	2.214196837	0.029149561	0.589138397
PRR15	6.23101352	0.029223247	0.589958778
RIMS1	5.133967264	0.029408297	0.592220894
SLC22A18	5.410207092	0.029434966	0.592220894
SIAE	4.217645072	0.029412227	0.592220894
RNASEH2A	2.504522518	0.029552259	0.593910445
SMIM6	4.430400083	0.029587645	0.593951985
IFT122	5.4445527	0.029690299	0.59467333
ELOVL1	2.490345841	0.029662027	0.59467333
ARAP2	6.158060766	0.029781447	0.595829491
WDR87	5.091766665	0.02987982	0.596557428
HDHD3	3.064329166	0.029884763	0.596557428
ACY1	1.937908946	0.029929808	0.596788324
UNC5D	5.445114531	0.03002498	0.597520134
TRAPPC5	2.589629306	0.030033549	0.597520134
ENSMMSG00000003889	3.325198901	0.030081246	0.597801883
ENSMMSG00000018693	6.923313292	0.030185869	0.599213038
ASTN2	7.142906532	0.030359957	0.600837145
SH2D2A	6.048659377	0.03043735	0.600837145
KLHL32	5.679128038	0.030469919	0.600837145
DRD1	4.566305894	0.030331465	0.600837145
FARS2	9.53879264	0.030461402	0.600837145
SERP1	1.983726898	0.030459445	0.600837145
KCNB2	5.828072299	0.030518843	0.601136905
SEPTIN10	4.611274877	0.030645126	0.602958072
SARDH	3.981689442	0.030803556	0.605407042
MYL6B	6.81412169	0.030928107	0.605524788
RBFOX3	6.371174478	0.030930455	0.605524788
MYO9A	9.511199152	0.030945421	0.605524788
PRLR	3.985438554	0.030938479	0.605524788
EPB41L4B	3.032293712	0.031027801	0.606471032
ENSMMSG00000016618	6.458704502	0.031137317	0.607324128
C8H9ORF152	5.409278672	0.031139586	0.607324128
RAB3D	2.591478592	0.03126687	0.609140134
PFAS	2.599280164	0.031431679	0.611682425
CPA4	4.638044449	0.031477436	0.611904874
ENSMMSG00000015652	5.9208033	0.03164149	0.612422586
MPZL3	2.843059304	0.031632762	0.612422586
PARD6B	4.221451705	0.031597503	0.612422586
ACOT8	2.20525818	0.031561048	0.612422586
G25092	4.133469421	0.031685216	0.612603745
GPR75	6.216932451	0.031887168	0.614163921
TNNI3K	6.075245664	0.031903724	0.614163921
CNIH2	5.421280475	0.031813893	0.614163921
BEST2	5.197716898	0.031864553	0.614163921
MSI2	5.424730753	0.031980734	0.614982273

VEPH1	4.719391801	0.032075122	0.616132676
LOC534967	4.113609806	0.032198151	0.61714753
GULO	3.222253804	0.032227395	0.61714753
HDAC10	3.082753628	0.032231816	0.61714753
ALOX15B	4.880276975	0.032318729	0.618147699
G26296	6.391536048	0.032413714	0.619299963
ENSBTAG00000055143	5.122553159	0.032567138	0.619575021
SOX10	3.644469754	0.032479014	0.619575021
CELSR2	5.456564774	0.03255941	0.619575021
LGALS3	2.614389607	0.032564772	0.619575021
CDC14A	2.988097135	0.03279111	0.623170924
ENSBTAG00000021359	2.015723346	0.032884067	0.624271977
CRB2	6.143085434	0.032952095	0.624897913
SPINT1	3.616939805	0.032995629	0.625058537
LRRC1	2.031991766	0.033168824	0.627672466
GNAO1	5.569054675	0.033340508	0.630252274
ENSMMSG00000018395	5.177605958	0.033378009	0.630292779
NEFL	6.385634139	0.033554011	0.632276744
DGKE	6.429200968	0.033520406	0.632276744
CARD14	5.066419733	0.03368679	0.633575401
CDC25A	3.04250972	0.033694013	0.633575401
IL1RL2	5.091941564	0.033815278	0.635185603
TMEM79	2.271415343	0.033863744	0.635426415
CORO2A	4.299547731	0.034016272	0.637617321
CFAP54	5.397854168	0.03412963	0.639070153
RPS20	3.352229818	0.034294674	0.641486744
C107131750/H3C6/LOC115[illegible]	6.427475425	0.034487972	0.644426199
ID2	2.603533919	0.034717992	0.648044943
ATP1A1	2.210551825	0.034950491	0.651702357
AP1S3	5.207373066	0.035010997	0.652148423
NHSL1	6.157684248	0.035146119	0.653410513
RTL1	6.531600812	0.035152063	0.653410513
ENSBTAG00000048984	3.224317573	0.035342237	0.656261154
DDI1	6.801718389	0.035406262	0.656267785
NFXL1	-9.107468274	0.035416224	0.656267785
CCDC190	5.861346788	0.035607792	0.657439232
KLHL3	5.814811816	0.035539365	0.657439232
ACP4	3.838291628	0.035626966	0.657439232
UBXN11	3.21356675	0.035596664	0.657439232
NOXA1	2.76008339	0.035736845	0.658784899
G28047	6.848540798	0.035802186	0.659180952
ENSMMSG00000020416	9.249718182	0.035869265	0.659180952
ELOVL4	4.920473598	0.035840981	0.659180952
DBIL5	3.905052288	0.035945455	0.659900801
SHPK	1.9208098	0.03606685	0.661448212
ENSBTAG00000001051	5.818671068	0.036444391	0.665010061
H2AC4	3.832251078	0.036385883	0.665010061
PLPP2	2.752526933	0.036484901	0.665010061
PPP1R13B	2.437566498	0.036418844	0.665010061
SLC25A1	2.881158807	0.03634828	0.665010061
CYP4F2	4.138971391	0.036477499	0.665010061
SHISA8	6.578500505	0.036675726	0.665087993
ENSMMSG00000010418	6.686876851	0.036603618	0.665087993
ASIP	3.557345455	0.036615622	0.665087993
H2AC14	5.250226199	0.036630806	0.665087993
SPOCK1	4.997886716	0.036666243	0.665087993
CYP2D14	2.503462467	0.036765541	0.66536298
RPN2	2.137859482	0.036739575	0.66536298

S100G	3.693753799	0.036844644	0.666118271
TMC5	5.721711746	0.037004359	0.666976444
GMPPB	1.875166671	0.03697321	0.666976444
MAP6	5.683324283	0.036964627	0.666976444
RAPGEF6	7.503851168	0.037169241	0.669271612
LOC112441470	6.367699893	0.037336452	0.670815806
EEF1A1	3.036389161	0.03731474	0.670815806
SLC35G1	2.090117365	0.037367895	0.670815806
LACC1	2.353608001	0.037464657	0.671876229
GOLGB1	9.165383879	0.037593688	0.673512642
C17H4ORF33	2.91061357	0.037890803	0.677473877
NACA	3.238748742	0.037853937	0.677473877
HYI	3.024716714	0.038039717	0.678774776
NRG2	3.800070779	0.038002653	0.678774776
MTTP	6.845020457	0.038318622	0.683067764
SLC1A2	5.096510546	0.038376013	0.683407396
SLC25A22	2.193091682	0.038449662	0.684035604
PANX1	2.855468124	0.038531555	0.684809066
TIAM1	2.26305379	0.038629064	0.685858255
PGF	3.166199537	0.038807669	0.687659543
KIF25	3.181287401	0.038798384	0.687659543
IFNLR1	4.145108052	0.038854941	0.687813491
RPL37A	6.688507274	0.039086529	0.691226657
LMO1	2.418804775	0.039194345	0.692446377
ALG11	3.145230507	0.039350861	0.694523219
INAFM1	2.988810531	0.039405565	0.694800796
LMF1	2.862964049	0.039601977	0.697573955
POU6F2	7.616861015	0.040102335	0.704868799
RASSF6	3.701888637	0.040174279	0.704868799
CHDH	2.440243293	0.040110151	0.704868799
TKT	2.102783943	0.040156972	0.704868799
GRIA3	5.670712638	0.040270886	0.705869046
ARHGEF19	2.44101987	0.040332056	0.706246786
ALCAM	3.089523164	0.040554435	0.709443919
TPBGL	4.82736451	0.040611521	0.709746054
ZNF10	5.310806532	0.040788772	0.712145594
KLK9	5.525527105	0.040916262	0.713672494
PLEKHA4	1.954987164	0.040996139	0.714366742
TNN	5.453217931	0.041065921	0.714883898
CLDN1	3.826953206	0.041161297	0.715845149
AGER	3.661448116	0.041304118	0.717628858
YBX2	2.496777857	0.041488778	0.720135299
G37499	6.028163563	0.041603286	0.720919043
LIMK2	1.931437253	0.041614815	0.720919043
SYT17	5.822042979	0.041814012	0.722264131
NIPAL1	4.816576606	0.041755676	0.722264131
PRR36	3.513740997	0.041807927	0.722264131
FRMD5	5.534266469	0.042017839	0.725082284
PTGR1	2.793715035	0.042110076	0.725971188
SPTA1	6.706089065	0.042621322	0.729709032
G31688	6.455569166	0.042423037	0.729709032
RNF224	6.00074825	0.042695306	0.729709032
TEX14	3.736575514	0.042588518	0.729709032
C8G	3.79784725	0.042474658	0.729709032
DLGAP5	4.735656945	0.042469546	0.729709032
RASAL3	2.130676657	0.042680286	0.729709032
RNF112	5.778919845	0.042512806	0.729709032
EEF1A1	2.623460403	0.042584683	0.729709032

ODC1	2.112534858	0.042880112	0.732165585
FPGS	2.880566271	0.04300145	0.733534779
CUPIN1	4.455231548	0.043194539	0.735316437
DMGDH	4.191388932	0.043169099	0.735316437
ABHD14B	2.20046688	0.043229644	0.735316437
DBF4	5.346559328	0.043387749	0.736942613
OXCT2	5.660607242	0.043451634	0.736942613
LY6G6C	5.088657038	0.043490611	0.736942613
GLRX	2.686114548	0.043423144	0.736942613
ALB	5.651200311	0.043645728	0.738167693
G29176	5.455822184	0.043642719	0.738167693
ZNF414	2.908458725	0.043712216	0.738591432
LOC107131843	5.452267136	0.043777645	0.738996497
CNMD	5.746249754	0.043836365	0.739287641
CLSTN2	-4.753628798	0.044028068	0.740882317
CABP4	5.955264595	0.044086826	0.740882317
ZBTB42	2.504234236	0.044068666	0.740882317
ASAH1	2.30362656	0.044097169	0.740882317
SLC7A1	2.138015806	0.044144721	0.740982863
/H2AC13/H2AC16/H2AC17/	4.679729977	0.04422558	0.741641757
TMPPE	5.295281316	0.044320221	0.7418331
TTC7B	6.188780532	0.044319815	0.7418331
CCR9	6.638074044	0.044510803	0.743626588
VAV3	2.533160996	0.044474941	0.743626588
ANPEP	2.708358964	0.044710303	0.746260175
G18688	4.552085686	0.044756097	0.746325705
CARHSP1	2.445173696	0.0448127	0.746571204
RPL35	4.093548453	0.045004781	0.749071177
MYCBPAP	2.082345198	0.045092898	0.74983769
PRKG2	6.540566791	0.045228744	0.750875494
BDKRB2	-7.678420366	0.045239553	0.750875494
CFAP119	2.999729009	0.045479841	0.752761053
SLC13A2	5.044121501	0.045417524	0.752761053
GRIP1	7.640658462	0.045475736	0.752761053
PLEKHH3	4.814401522	0.045759649	0.756689705
DHCR7	1.925014602	0.04582009	0.756986957
MLH3	5.661166626	0.045897786	0.757568459
LIPN	6.440930695	0.045951579	0.757754718
GDF15	4.67422275	0.046067588	0.758441821
CATSPERG	2.52173268	0.04607834	0.758441821
G11359	6.730538887	0.046162594	0.759127678
G25201	2.858445388	0.046430949	0.762192146
TENM2	3.339531575	0.046434459	0.762192146
FST	3.625989363	0.046499973	0.762565328
CYBA	2.432914429	0.046564325	0.762918804
ENSMMSG00000007716	5.175154796	0.046709166	0.764589153
BATF3	5.250878277	0.046836773	0.764674869
ADAMTS18	4.612694385	0.046843092	0.764674869
RAPGEFL1	4.570791172	0.046809885	0.764674869
TENM4	1.693727367	0.046891186	0.764759643
IL2RB	5.494923969	0.047158904	0.768422869
ENSMMSG00000005912	6.57019291	0.047308357	0.769551938
TMEM53	2.800292623	0.047314536	0.769551938
G24926	4.967336502	0.047366693	0.769697966
NAP1L1	2.192481567	0.047446523	0.770292998
PPP1R9A	5.702792454	0.047607297	0.772199891
SCG5	5.066484534	0.047752793	0.772564628
BDH1	3.098214665	0.047720075	0.772564628

S100A4	2.708084442	0.047759801	0.772564628
IL1RN	3.900439207	0.047806113	0.772612671
MAP2	6.004364469	0.047988762	0.774862018
RPL31	4.833966364	0.048094054	0.775157867
CCNJL	5.036442519	0.048060254	0.775157867
TM6SF2	5.06141205	0.048210806	0.775637035
FOXM1	4.652466199	0.048187004	0.775637035
G11853	5.24339556	0.048555066	0.780471234
EPPK1	3.216117609	0.048599934	0.780488667
LRTM1	5.705944005	0.048835374	0.782326046
GUSB	2.678191668	0.048846005	0.782326046
SLC2A13	4.480866732	0.048821439	0.782326046
HEPACAM	5.434690934	0.049150768	0.785958055
VAMP1	2.989940001	0.049160958	0.785958055
ENSMMSG00000020843	6.128778265	0.049220093	0.786198369
RUNX1	1.842449602	0.049272781	0.786335356
G34422	5.950255338	0.049379145	0.787327947
GALM	3.160906462	0.049539671	0.78918157
AGMO	2.450557211	0.049609721	0.789446839
SCML4	4.876750268	0.049644895	0.789446839
NMB	5.173825182	0.050028942	0.792425643
ENSMMSG00000014498	4.722931728	0.050222497	0.792425643
ABHD1	2.841405786	0.0502323	0.792425643
TBX2	4.084586002	0.050115392	0.792425643
CCNB2	3.706199475	0.050111145	0.792425643
ZNF827	2.756660899	0.050167519	0.792425643
TMEM30B	2.861434551	0.050046785	0.792425643
MPG	2.758881846	0.050189205	0.792425643
DSG3	4.129928858	0.05002828	0.792425643
PAK6	4.358480361	0.050392154	0.79284248
FAT1	4.940741699	0.050386716	0.79284248
TBC1D16	2.484135218	0.050352312	0.79284248
RFX3	6.034102424	0.050467697	0.792923744
RAP1GAP	4.92205041	0.050530763	0.792923744
CDH3	2.102054584	0.050507636	0.792923744
CLCN2	1.995165909	0.050707501	0.794345272
BTD	2.024992052	0.050750953	0.794345272
C21H15ORF39	1.954118825	0.050755036	0.794345272
RADIL/MMD2	5.085416357	0.051088997	0.794392681
LOC506828	5.442750946	0.050984589	0.794392681
G17564	5.26848835	0.051067137	0.794392681
CCNB1	6.275404993	0.050858613	0.794392681
CDC42EP5	2.755832395	0.051159138	0.794392681
UGT1A1/UGT1A6	3.207384459	0.05092173	0.794392681
EDARADD	4.594305469	0.051131557	0.794392681
ATP2C2	2.423182581	0.051147285	0.794392681
S100A11	2.377047156	0.050936008	0.794392681
LYPD1	3.777621887	0.051399273	0.797426842
TMCC3	5.72485219	0.051492078	0.798171981
SYCP2	6.417271007	0.05161443	0.798679539
METTL7A	3.393845683	0.051601563	0.798679539
SPINK2B	5.765625164	0.051768036	0.799184966
PLCXD2	6.458047662	0.051727751	0.799184966
BMPER	5.483299474	0.051781591	0.799184966
BPIFB1	5.300076234	0.051835159	0.799319681
OVOS2	6.115503158	0.051996048	0.799737831
GRB14	2.941225558	0.052011158	0.799737831
SMPD2	2.236229842	0.05204173	0.799737831

APMAP	2.821934848	0.051965379	0.799737831
USP20	2.483768377	0.052150618	0.800720855
RPL10A	5.511536733	0.052201053	0.800805479
LTB4R	2.918609842	0.052252106	0.800899441
NIPAL2	1.825284655	0.052820865	0.808227241
PER2	2.230725316	0.052789401	0.808227241
RPS20	3.08408934	0.05304111	0.809637197
PDZK1IP1	-5.032758227	0.053049268	0.809637197
ENSMMSG00000007184	2.941573548	0.053045007	0.809637197
LRRC8B	2.078608116	0.053197103	0.811198941
BAIAP3	1.997375169	0.053244609	0.811229403
CAMK1	1.720812389	0.053310373	0.811537755
RASAL1	3.516117565	0.053475297	0.813353787
DCLRE1A	6.355004031	0.053726219	0.815083896
VSIG10L/ETFB	3.173602453	0.053691962	0.815083896
ALPL	1.855939572	0.053705627	0.815083896
TRPC1	5.77959999	0.054238145	0.815331444
APOA1	6.085522008	0.053817824	0.815331444
RIMS3	4.538313681	0.054235516	0.815331444
SPA17	2.870784334	0.053952976	0.815331444
SLC46A1	1.941198946	0.054035947	0.815331444
EBPL	3.443390286	0.054098616	0.815331444
ZSWIM3	2.716150015	0.053919992	0.815331444
IKBKE	1.899563949	0.054133384	0.815331444
PLCD1	2.846615096	0.053962085	0.815331444
G22444	2.33987595	0.054291396	0.815331444
GLTP	1.614943352	0.054007383	0.815331444
TSKU	1.958834517	0.054289999	0.815331444
G17202	6.380475332	0.054439556	0.81668756
ENSBTAG00000038978	2.740291714	0.054473326	0.81668756
PTPRR	2.740821861	0.054750697	0.819479423
ENSMMSG00000013041	1.99354997	0.054751486	0.819479423
GNAT3	6.574670406	0.054938106	0.821219618
ENSMMSG00000005265	5.256869916	0.05504632	0.821219618
NRG1	4.687710897	0.054961609	0.821219618
ARSL	2.771537719	0.055052028	0.821219618
ENSMMSG00000000945	6.930926165	0.055276093	0.821517319
SORCS1	6.559393191	0.055358059	0.821517319
MSI1	5.675817517	0.055394582	0.821517319
REPS2	4.792188145	0.055245367	0.821517319
KIF1A	2.42634877	0.055179468	0.821517319
CADPS2	2.844896818	0.055231712	0.821517319
KIAA0513	2.205273879	0.055306426	0.821517319
ENSMMSG00000015527	4.628760123	0.055729835	0.825472271
ENSMMSG00000008269	2.363435469	0.055796981	0.825472271
DNAH9	3.31049041	0.055800184	0.825472271
CEP290	8.429592735	0.055856048	0.825613521
PARD3	5.343507853	0.056005914	0.827141853
VRK2	4.988489538	0.056052247	0.827141853
G20663	5.285400906	0.056106198	0.827253171
ENSMMSG00000001049	2.836191354	0.056179751	0.827653092
DGKG	5.100975816	0.056369105	0.829756954
CYP2J2	5.381557757	0.056605081	0.831577358
DOCK9	5.6852138	0.056647659	0.831577358
F2RL1	3.084167224	0.056609295	0.831577358
RAB11FIP1	2.700498137	0.056679372	0.831577358
CADPS2	2.851683039	0.056750078	0.831930006
PRKAG2	4.30261842	0.056882154	0.833180996

PYROXD2	2.215346191	0.056946558	0.833439532
JAKMIP2	5.482838593	0.057067347	0.834522176
KBTBD11	4.267193167	0.057347011	0.837924446
SIGIRR	2.475433556	0.057535332	0.83998758
SREK1	3.832206806	0.057666614	0.841215269
HUNK	6.200691595	0.058055107	0.845006127
STAG3	4.083439996	0.057993352	0.845006127
SLC35F1	3.591751275	0.058068692	0.845006127
CNGA3	4.562595842	0.058243338	0.846856239
G13502	5.506547075	0.058382372	0.847259968
HCLS1	8.343410683	0.058413694	0.847259968
DDHD1	1.969266409	0.05831878	0.847259968
STYXL1	2.970828126	0.058667483	0.850098176
SQLE	2.207656144	0.058704749	0.850098176
ENSBTAG00000049554	6.481835416	0.058950272	0.850499229
FAM222A	4.631048773	0.058975541	0.850499229
G17203	2.782304002	0.059066422	0.850499229
LDOC1	4.813530671	0.058815847	0.850499229
GPRC5A	3.695919662	0.059061347	0.850499229
STARD10	2.532245295	0.05892041	0.850499229
DSTN	1.736665902	0.05886362	0.850499229
G31570	4.762669618	0.059252709	0.85249297
SPINT2	2.116700501	0.059333711	0.852969938
GRIN3A	5.463716656	0.059496932	0.854627162
NT5C	2.540666109	0.059587634	0.855240874
LOC516494	2.839408489	0.059697001	0.856121273
CPNE4	4.48668363	0.060069209	0.858695842
JMJD1C	6.083274085	0.060034126	0.858695842
B4GALNT3	2.785508575	0.059954459	0.858695842
MTA3	3.223789348	0.060052882	0.858695842
PGGHG	1.985894439	0.060176161	0.858847269
HEBP2	2.157914667	0.060147336	0.858847269
PSD4	2.188077913	0.060262992	0.859398481
CHMP4C	2.209198863	0.06044616	0.861321544
C9ORF116	2.555146972	0.060519333	0.861675427
RGR	5.127197064	0.060599448	0.862127502
SHANK2	3.903937332	0.060664111	0.862359205
G31501	6.362417566	0.060978765	0.866141413
TGFA	3.752400109	0.061128155	0.867572048
ZNF300	5.411920103	0.061328392	0.868082972
GJB3	3.72067474	0.061327862	0.868082972
PAXX	2.620447096	0.061308159	0.868082972
KIF21A	2.447008569	0.061358945	0.868082972
LHX8	4.608231281	0.061432525	0.868138136
PLCXD1	2.382417752	0.061460245	0.868138136
SLFNL1	4.898584493	0.06151473	0.868219782
PYGL	2.49399041	0.061634576	0.869223065
G32253	5.943130779	0.061831672	0.870325577
PLA2G2E	-8.086323003	0.061900121	0.870325577
XAF1	5.426302411	0.061937418	0.870325577
CATSPER2	4.042404504	0.061932539	0.870325577
PTTG1	3.263242308	0.06196133	0.870325577
GPR108	2.103264528	0.062054516	0.870325577
NCAPG	3.068208934	0.062021226	0.870325577
G28017	5.422689158	0.062149781	0.870976416
C12H13ORF46	5.6466737	0.062331825	0.872273801
BCL11A	3.280977251	0.062354481	0.872273801
SLC41A2	2.213621351	0.062389156	0.872273801

ASRGL1	6.408185917	0.06276854	0.875823631
SLC24A4	5.554242604	0.062790452	0.875823631
LOC617396	3.125169374	0.062757141	0.875823631
LPIN2	2.172710999	0.062846892	0.875925481
LPAR2	4.834700564	0.063116571	0.878996865
ABCC4	6.839350757	0.06333707	0.88089129
MPIG6B	5.488453979	0.063351432	0.88089129
FYB1	2.256404898	0.063608678	0.883778872
TFEC	5.307434368	0.063746326	0.883915563
FCER1A	3.394830912	0.063767273	0.883915563
SF3B2	2.294163171	0.063692147	0.883915563
PRRC2C	2.446608728	0.064079935	0.887559382
LOC785907	5.458931123	0.064335825	0.890411811
HNRNPC	5.968284133	0.064705671	0.890986104
GP2	5.534915017	0.064593475	0.890986104
LHB	2.580675086	0.064694958	0.890986104
CYP1A1	3.204874096	0.064777179	0.890986104
RAPGEFL1	2.632675733	0.064527773	0.890986104
RAB27B	2.787266684	0.064737443	0.890986104
NCOR1	3.664441502	0.064743528	0.890986104
SPSB1	2.247326158	0.064741987	0.890986104
FKBPL	2.370498264	0.06498554	0.893162863
NOSTRIN	2.112988366	0.065059202	0.893486391
FSD1	3.779120988	0.065247689	0.89538514
GALK1	1.993631512	0.06535239	0.895443284
MICALL2	2.119794404	0.065328147	0.895443284
NT5DC1	2.363893028	0.065738635	0.898579406
MCTP2	2.757806199	0.065738236	0.898579406
TRIM36	2.030817693	0.065782908	0.898579406
KRT16	4.121700347	0.065735333	0.898579406
SLC38A5	6.219089145	0.0658844	0.899276658
ENSMMSG00000022451	4.359886013	0.065936778	0.899302994
RAD18	5.616040615	0.066150645	0.901530115
FOXP1	4.491247515	0.066233555	0.901970481
NPAS4	5.601903532	0.066426111	0.903207882
TRPM2	2.96441713	0.0663946	0.903207882
PYGL	2.599241688	0.066476424	0.903207882
PGLS	1.833364566	0.066555651	0.903595611
OTOR	-5.077103069	0.066735349	0.903969852
MFSD5	1.737590088	0.066686958	0.903969852
EPB41L1	2.233518628	0.066671965	0.903969852
G7471	5.95078221	0.066884954	0.904748088
PTPRZ1	1.919992858	0.066894311	0.904748088
SLC37A2	4.043050275	0.067207384	0.908293277
FAIM2	4.598953509	0.067273591	0.908499274
ERG28	1.721852898	0.067445172	0.910126903
CKMT1A	4.343195129	0.067905017	0.915639051
G30207	6.330976599	0.068410736	0.919675553
ZNF618	6.077669087	0.068397782	0.919675553
BLNK	4.571582748	0.068323255	0.919675553
RPS24	3.096462881	0.068291553	0.919675553
C25H16ORF91	6.159545679	0.068575732	0.920505268
AGTRAP	2.042256738	0.06852568	0.920505268
ENSBTAG00000009620	2.323460148	0.068726401	0.921629208
CKB	2.179674452	0.068762866	0.921629208
EPPK1	3.090732965	0.068885179	0.922574911
SCAP	2.702439454	0.068938787	0.922599707
FAM92A	3.010917227	0.069130061	0.924465463

MYMK	3.849691455	0.069268668	0.925624651
ALPK1	2.534212743	0.06941029	0.926822342
NDUFB11	4.380452147	0.069653683	0.927421494
AMDHD1	5.105424037	0.069715292	0.927421494
IQCG	5.531582715	0.069579628	0.927421494
GPR87	5.033438256	0.06962177	0.927421494
CASKIN1	2.841568725	0.069684767	0.927421494
AIFM3	4.007715896	0.07000111	0.930529303
TYROBP	2.799003905	0.070056051	0.930565697
ADI1	2.268036297	0.070115205	0.930657964
G29720	5.233903198	0.070206593	0.931177621
C2CD4B	3.375891686	0.070365713	0.931901332
KARS1	2.518178666	0.070338632	0.931901332
TRIM9	4.818676363	0.070604123	0.93339841
ATP8B1	1.597741862	0.070635838	0.93339841
CHD3	2.38024179	0.070587635	0.93339841
TACC3	2.269648243	0.070922778	0.936495878
RHOT2	5.500805883	0.071040667	0.936664892
LRIT1	5.917365096	0.070999542	0.936664892
MKI67	4.305889595	0.071148857	0.937398028
BRINP1	-6.457249736	0.071283538	0.938073967
CASP3	2.516154448	0.071305409	0.938073967
RSPH10B	5.460152763	0.071616274	0.940775014
UNC93B1	1.927693393	0.071575522	0.940775014
PARD6G	2.787566309	0.071875905	0.942045315
DSCAML1	4.054745655	0.071906223	0.942045315
SSC5D	2.211667306	0.071924362	0.942045315
FKBP2	2.103120149	0.071848854	0.942045315
METTL7A	2.742898553	0.071977758	0.942052502
SCRN1	2.114928017	0.072181174	0.943709002
RASSF7	2.161055126	0.072261806	0.943709002
CEP170B	2.080741522	0.072263143	0.943709002
HTR3A	-7.780132283	0.072494263	0.945156633
PFKL	2.512996116	0.072460404	0.945156633
SEC11C	1.599463776	0.072533057	0.945156633
BCL2L14	4.319062314	0.072593237	0.945249854
DNAJC3	1.622835623	0.072794461	0.947178145
CLDN14	7.155029793	0.072942925	0.947726367
IL1RAP	1.979002762	0.072911364	0.947726367
HOXC4	4.827522191	0.073088901	0.948471629
NAGK	-5.815688649	0.073148562	0.948471629
PTK7	2.148531313	0.073159906	0.948471629
G13734	5.220582997	0.073522293	0.951094627
CERS6	4.463296528	0.073513312	0.951094627
B3GNT8	2.664613241	0.073513	0.951094627
HOOK2	2.618576842	0.073819606	0.954248226
SMARCD1	5.8788078	0.074245681	0.954643013
DCDC2	4.866277244	0.074440458	0.954643013
CDHR1	5.534108707	0.074106212	0.954643013
CDKN2B	4.275531036	0.07431781	0.954643013
PANX1	2.954727225	0.073990976	0.954643013
LPIN3	4.299423573	0.074492788	0.954643013
KLHL17	3.697821563	0.074405144	0.954643013
PEMT	2.315967921	0.074327913	0.954643013
MSMO1	2.196431116	0.074313944	0.954643013
ZCCHC17	2.469527184	0.074484113	0.954643013
MYOF	2.075726702	0.074251488	0.954643013
SPHK2	1.988476709	0.074080653	0.954643013

PTPRN2	6.984906618	0.074773643	0.957553855
G27342	3.530934216	0.075035422	0.960216386
G15775	4.796443881	0.075145451	0.960770504
SPATA6	2.890459357	0.075186517	0.960770504
LNP1	2.794977581	0.075442418	0.963349959
SOST	-7.642539795	0.075833278	0.967647832
ENSMMSG00000008614	5.54510098	0.076117466	0.970579363
SEC61B	1.703288415	0.07633512	0.972658939
SLC39A12	5.384712105	0.076596653	0.975279801
ENSBTAG00000054700	1.773416884	0.07665023	0.975279801
G22797	5.385446403	0.076836545	0.976953098
PRSS57	5.68720205	0.077119124	0.978452311
TMEM45A	1.704362755	0.077034019	0.978452311
ACTG2	1.816082083	0.077098723	0.978452311
ODF3	3.483908307	0.077186656	0.97861261
C1H3ORF33	2.560835341	0.077395421	0.980562032
RPL23A	4.853634718	0.077558801	0.981934086
ARMC2	6.084685234	0.077786656	0.982028988
LIPI	6.192452251	0.077782831	0.982028988
TONSL	2.003323692	0.077785308	0.982028988
PXYLP1	1.995232044	0.077670755	0.982028988
G7770	6.483694211	0.077936933	0.983229838
NME9	5.550332686	0.07802567	0.983245908
H2BC12	3.636777656	0.078048522	0.983245908
CTNNA3	4.402056668	0.07827891	0.98475642
MACIR	2.656375352	0.078272493	0.98475642
HSH2D	5.670132891	0.07847114	0.985053679
EMID1	3.262065904	0.078469369	0.985053679
ENSMMSG00000023767	2.353356968	0.078493671	0.985053679
ACADL	2.007439396	0.078523577	0.985053679
CDK14	3.224625148	0.07867483	0.986257045
NOP14	3.179299169	0.078801509	0.986566362
TRIP6	2.660019641	0.078810193	0.986566362
NECTIN1	1.827472567	0.078940547	0.987504695
GBP1	5.279127873	0.079226562	0.988693017
TREML2	6.368374189	0.079602209	0.988693017
GGN	4.329533056	0.079406528	0.988693017
G14088	5.077276192	0.079504984	0.988693017
G27631	4.522635688	0.079563484	0.988693017
TK1	2.944410831	0.079330632	0.988693017
SPC24	4.209735818	0.079474039	0.988693017
NAPRT	3.546363045	0.079626328	0.988693017
HOXD1	2.804832491	0.079486481	0.988693017
KRT15	4.408228513	0.079251679	0.988693017
TAF10	2.465742639	0.07964564	0.988693017
G25814	5.933997549	0.079785934	0.989745345
SHANK1	-4.795962597	0.080161018	0.993706749
TMEM121	4.364232144	0.080246606	0.994076439
SCAP	6.018647674	0.080508958	0.995770592
AWAT1	4.397078042	0.080539723	0.995770592
GNMT	1.751089316	0.080589049	0.995770592
SPPL2A	2.438758368	0.080606808	0.995770592
G5310	5.579399901	0.080767822	0.996479551
ZDHHC16	3.060952076	0.0809437	0.996479551
SYTL5	3.844823716	0.080793408	0.996479551
TMTC4	2.172230911	0.080916955	0.996479551
KCTD15	2.366111413	0.080881677	0.996479551
CBR3	2.380304994	0.08105048	0.997105493

MYBPC3	5.666377723	0.081161022	0.997776808
FHIT	3.53467555	0.081547147	0.999953367
MYOF	2.391677646	0.081394301	0.999953367
SLC27A4	1.868430663	0.081453963	0.999953367
TRAPPC6A	2.480605221	0.081562448	0.999953367
ENSBTAG00000035182	0	1	1
RHCG	0	1	1
AIPL1	0	1	1
RETN	1.661031843	0.712878856	1
SLC26A8	0	1	1
C11H2ORF81	0	1	1
RAX	0	1	1
G22585	0	1	1
SLC5A9	0	1	1
ENSBTAG00000050190	0	1	1
G28872	0	1	1
ENSMMSG00000021917	0	1	1
MLN	0	1	1
CACNG5	0	1	1
G16340	0	1	1
HBZ1	0	1	1
ANAPC15	-1.89919159	0.650648961	1
PRKRIP1	3.06283969	0.487845919	1
GDF3	5.146427654	0.244006087	1
ESR2	0	1	1
IL17C	2.088469608	0.641762723	1
SHEEP	0	1	1
PDCD1	-3.57569332	0.41645567	1
CRNN	0	1	1
G7510	5.234396779	0.23597606	1
ENSMMSG00000016442	0	1	1
HTR4	5.234396779	0.23597606	1
TPRG1	1.565334517	0.686009978	1
GJD3	-6.167730031	0.155282873	1
SHISAL2B	5.052767846	0.252766342	1
ELAVL3	0	1	1
FCGR3	4.95257824	0.262379653	1
RP1	0	1	1
OLFM3	5.3957131	0.221744226	1
APOA4	0	1	1
CATSPERB	5.470071439	0.215397012	1
KLK14	2.695021867	0.545800198	1
NONO	2.700675392	0.371842713	1
APOBEC3Z1	0	1	1
G13350	0	1	1
CLPS	0	1	1
IL12A	5.540787244	0.209484082	1
LOC112449619	4.95258634	0.262378866	1
LOC519202	5.395731278	0.221742658	1
TENM2	0.21028027	0.948133611	1
R3HDM2	0	1	1
ENSBTAG00000050686	0	1	1
ENSMMSG00000018597	0	1	1
RPS14	2.596330019	0.560934495	1
SHCBP1L	5.734250994	0.193914396	1
VWA5B1	-2.986744224	0.500799721	1
EEF1A1	-0.977868956	0.708075627	1
GOLT1A	0	1	1

BPIFB5	0	1	1
EPS8L3	-1.17337573	0.743520117	1
G34816	4.916354413	0.265917338	1
RNF8	-0.560603445	0.825442853	1
ENSMMSG00000008198	5.395176408	0.221790524	1
SOSTDC1	0	1	1
ENSMMSG00000019575	0	1	1
SLC16A8	0	1	1
FMN1	5.146455195	0.244003543	1
G16090	0	1	1
PGLYRP4	0	1	1
SIRPB2	0.771924955	0.773733784	1
BRINP1	0.320253468	0.938669007	1
GIPC2	5.516094902	0.211535136	1
SNTG1	5.76636319	0.191415055	1
LRAT	4.13794173	0.349993715	1
G23690	0	1	1
ENSBTAG00000053916	5.562288286	0.207709963	1
PTH2	-3.298106727	0.407873734	1
ENSMMSG00000018584	-5.87222262	0.17632005	1
LOC781423	0.780022336	0.853158813	1
NKTR	3.046979979	0.316166773	1
PTPRD	5.904809788	0.180912356	1
ENSMMSG00000010479	4.708552187	0.269179907	1
NELL2	0	1	1
ENSBTAG00000052102	4.951506723	0.262483826	1
CCR3	1.460121579	0.612558638	1
ERP27	4.602143787	0.297995716	1
MOGAT2	0	1	1
ENSMMSG00000001991	5.052778326	0.252765349	1
CATSPER3	0.082407371	0.98061406	1
ABCC8	1.016473192	0.808823312	1
MCEMP1	0.57955644	0.890353538	1
UBA2	-0.360659087	0.870403529	1
G6PC1	0.572219335	0.873293804	1
G2508	4.600937031	0.28017752	1
AP3B2	5.672050303	0.198824038	1
G7797	4.805322392	0.242235225	1
CHST13	4.728594661	0.284784918	1
CCL20	0	1	1
SLAMF6	5.165891771	0.242212886	1
GRHL3	0	1	1
NAV3	6.008254155	0.173350448	1
ENSMMSG00000008530	-0.546332203	0.835260501	1
CER1	0.035520162	0.991181713	1
FBXL19	-6.856334247	0.113774885	1
RPL23A	-0.607335804	0.853008254	1
TOMM20L	4.137804644	0.348432243	1
TEKT2	0	1	1
ENSMMSG00000010805	5.084091709	0.249812251	1
PLD5	5.762059214	0.152556962	1
LOC112445823/ZIC3	5.867870557	0.183671543	1
TBX21	4.950720166	0.229981573	1
FGD3	0	1	1
NEO1	0	1	1
ACOD1	6.057320445	0.169847837	1
DLX1	0	1	1
ATOH7	5.608229904	0.203956015	1

SVOP	0.56734634	0.894763865	1
ENSMMSG00000007600	4.336775683	0.327046513	1
UNC80	5.315737043	0.228720536	1
ENSMMSG00000001460	4.73862838	0.188768614	1
LOC514257	-1.04482868	0.725937988	1
KCNK1	-0.708805193	0.858290762	1
F2	0.581138553	0.843501006	1
GCK	5.442556523	0.217730128	1
ADCY8	0	1	1
TARM1	0	1	1
GATC	-0.689085312	0.846090709	1
G6759	1.510670747	0.704133777	1
RBM44	5.732802376	0.180513046	1
RPL12	5.627525999	0.202394225	1
BFSP1	5.904832737	0.180910652	1
FAM151A	2.019948862	0.563407708	1
BNC1	0.13898788	0.969834567	1
TMEM178B	1.259407595	0.766119852	1
RPSA	1.657424051	0.686392505	1
GPLD1	0	1	1
ENSMMSG00000016242	2.088456634	0.641764845	1
LIPN	0	1	1
ENSMMSG00000003445	4.413184091	0.30174442	1
G21058	5.143801064	0.200637759	1
CSRP3	6.120080863	0.165445779	1
SHANK1	4.952602872	0.262377259	1
PRTG	5.848496299	0.185131191	1
SYCP2	0.517492739	0.877105659	1
G32588	1.931426513	0.557108743	1
RPL12	5.144272758	0.201737426	1
SAPCD1	-0.586380555	0.834371008	1
PRICKLE2	5.234427088	0.235973326	1
OAZ3	3.958532092	0.232909854	1
PRL	1.274644949	0.74194885	1
ENSBTAG00000037932	-0.306483013	0.934219635	1
G850	6.195239716	0.160288341	1
G992	0	1	1
DNAAF6	0	1	1
ENSMMSG00000022392	0	1	1
CPA1	5.511708927	0.146625899	1
ENSBTAG00000049406	4.822475639	0.236910112	1
TBC1D7	4.463262196	0.312975045	1
RAB20	0.298679717	0.932474841	1
ASMT	-5.392752336	0.186985	1
OTOS	-0.840700753	0.841224719	1
TPM4	0.813286888	0.743729235	1
G30265	3.78262271	0.274352782	1
PKHD1	6.200529726	0.159929993	1
COL9A1	0	1	1
G11079	4.763382444	0.176683806	1
DPP6	2.209143033	0.590707907	1
AMDHD1	4.53763103	0.224691358	1
TXK	5.146474643	0.244001747	1
EXOSC2	-0.624438678	0.852577052	1
G29308	0.022058501	0.995800846	1
FLG2	-2.720717063	0.476305317	1
LOC613966	0.059757778	0.984089572	1
CXCL8	0	1	1

MRPL51	2.922214466	0.293056982	1
WNT5B	0.992355507	0.73949058	1
KCNQ3	-0.895984898	0.808638664	1
NEURL3	-0.853087003	0.816761394	1
G20477	2.307510195	0.503213349	1
TSPAN32	3.957967932	0.37163382	1
ENSMMSG00000000160	-1.068892347	0.782544902	1
CHRM4	0	1	1
ERFE	4.483924139	0.162418197	1
ENSBTAG00000049803	4.602003452	0.298010603	1
SORCS3	1.682254267	0.671631237	1
OTOP1	5.303923301	0.229764261	1
G10303	0	1	1
PLSCR5	5.143189	0.201369156	1
SLC35F4	6.072077111	0.168804936	1
CELF3	5.492181052	0.21353543	1
UQCC2	4.728009348	0.087096668	1
ENSBTAG00000054953	-5.694253743	0.150087204	1
G23606	0.404620483	0.889973529	1
SLC24A1	0	1	1
NAT8L	0.077097731	0.97685176	1
ALDH8A1	-7.010914739	0.097187998	1
CACNA1I	2.148917071	0.613041069	1
ZSCAN10	0.50457134	0.875017991	1
ESPNL	0.660125388	0.808867207	1
ADAMTS16	0.699271652	0.843737913	1
DPF1	-0.87818713	0.767020187	1
P3H3	2.269766396	0.593780623	1
TAT	-6.608472817	0.127562107	1
KLHL1	5.341072012	0.226493721	1
KCNB2	4.805636519	0.246619598	1
CTXND1	4.951336143	0.236857501	1
CPQ	5.652560764	0.154989547	1
MPIG6B	2.643292924	0.485726557	1
CAPN8	0	1	1
GCSAML	1.9068378	0.56756908	1
LOC282685	0.980444579	0.686117668	1
CCDC153	2.434165671	0.519798484	1
LSAMP	5.075501595	0.250619934	1
IL1A	-0.942244389	0.819574957	1
TBR1	-1.464802613	0.664690552	1
BECN1	-1.069053671	0.693648019	1
GPR88	-1.117816085	0.716182673	1
TSNAXIP1	1.005254991	0.786232026	1
NFKBIA	-1.609255813	0.6003799	1
TNNI3K	6.05705847	0.158105121	1
F8A1	3.942847321	0.373489397	1
MTHFD1L	-1.118984984	0.631716189	1
EDC3	0.239098907	0.924504996	1
ENSBTAG00000049343	0.252702894	0.939353845	1
CERS3	0	1	1
ABCG5	2.088482195	0.641760663	1
KRT34	0	1	1
RTL9	0	1	1
KCNF1	5.774570315	0.136767559	1
CYP3A24	6.360746907	0.149363022	1
BECN1	-1.161857341	0.726936371	1
SCN8A	0	1	1

ENSMMSG00000009878	5.635346796	0.20176373	1
PCNX2	5.052803895	0.252762928	1
MIXL1	0	1	1
CATHL5	3.165285553	0.476562195	1
SPATA46	-0.470219587	0.878143063	1
RHEX	3.480580426	0.432137855	1
LIPC	2.962274845	0.453428311	1
EID2	0.604713153	0.857123311	1
G3749	3.060700083	0.25969283	1
CDA	-4.891762744	0.11106109	1
SSLP1	-3.01672692	0.36456681	1
PKIB	-0.117480155	0.96287938	1
PHEX	4.135985167	0.327012135	1
PKD1L2	4.594726881	0.204553845	1
HTR7	-0.448315413	0.900703823	1
RPS3	3.799380862	0.370890921	1
F11	6.179184578	0.161379661	1
KIAA1549L	0	1	1
G35757	-2.39984244	0.572009479	1
S1PR4	-0.179833022	0.949389405	1
ENSMMSG00000023551	4.461691272	0.278724022	1
MUSK	2.224787432	0.568955251	1
PRPH	0	1	1
G9805	3.449701677	0.437147402	1
ENSBTAG00000054066	-0.281640973	0.919120535	1
ENSBTAG00000045577	1.247762683	0.763559464	1
VGF	2.629187074	0.45584394	1
SLAMF8	4.463285808	0.312972456	1
G37809	3.042726837	0.494131372	1
THL1/LOC112441458/CATH	0	1	1
ENSBTAG00000054230	-0.121549136	0.954458602	1
F7	-6.345733468	0.131633061	1
ZNF512B	-0.640823887	0.739198118	1
G25828	3.870304262	0.300957826	1
SMYD2	-0.643051638	0.781796309	1
TPH2	0	1	1
CALB1	1.933616116	0.556648105	1
G37679	5.523578363	0.087260043	1
CADPS	4.728577479	0.284786686	1
CFAP46	-5.403911737	0.178050161	1
GLRA2	6.26519836	0.155598401	1
G714	5.317343211	0.228578898	1
HSD17B6	0	1	1
LOC777598	0	1	1
ACE3	0.55810752	0.892311401	1
ENSMMSG00000014392	0	1	1
RPL27	3.449433248	0.363405976	1
GRM5	4.143618307	0.243011649	1
NR2E1	0	1	1
BFSP2	2.226127359	0.572344175	1
PRDM6	0	1	1
SP5	3.357341799	0.362329585	1
G23693	4.728582055	0.284786215	1
TTBK2	2.105320717	0.463973357	1
RPL23A	5.468987655	0.215488566	1
MTMR3	-7.356166366	0.089583047	1
POLR2G	-0.007530004	0.997653963	1
TSPAN8	0.106271628	0.971395206	1

CACNA1C	6.238453178	0.157378849	1
ZFP27	3.717234716	0.401861166	1
G12346	1.232473308	0.735152768	1
ANKRD66	3.931688697	0.34159939	1
PRSS2	-6.608476459	0.12390177	1
PCSK1	2.69500086	0.545803398	1
MRPL16	-0.870271827	0.756087137	1
CNTF	-0.030521542	0.99015128	1
OXTR	0.461790629	0.903349656	1
KPNA7	2.249996854	0.501705227	1
CD8A	-0.447373053	0.869925443	1
GRM7	5.987911724	0.17481842	1
PPP2R1A	6.544050847	0.137939529	1
IL22RA1	3.938041119	0.286314523	1
DLGAP1	4.601751186	0.135247149	1
CPNE7	0	1	1
CSPP1	4.353131442	0.094885991	1
SUCNR1	1.646364519	0.652639277	1
ENSMMSG00000004222	-1.332250118	0.703831837	1
S100A6	1.445601514	0.629928817	1
KRT13	-0.521588014	0.888638826	1
GMFG	0.626471694	0.793504213	1
G2250	0.187067256	0.954209029	1
G14363	2.870063865	0.389721585	1
FBN3	-4.020276536	0.266342272	1
MED12L	4.767792469	0.280772312	1
G9281	3.614161122	0.273271569	1
ENSMMSG00000023902	-0.412374356	0.882779254	1
TMEM30C	0	1	1
G13747	0	1	1
LOC617313	4.086145114	0.249223953	1
ENSBTAG00000051819	5.491209834	0.154124527	1
SHISA6	2.089879111	0.585823619	1
CXCL13	5.440781048	0.097971374	1
KRTAP8-1	0	1	1
TKTL1	0	1	1
PABPC1L	4.555901983	0.268958722	1
RXFP3	-2.199331171	0.527667631	1
SNX22	5.079666744	0.250228077	1
SCN5A	-1.508866722	0.70214999	1
CDRT4	1.427407836	0.736010427	1
FGF12	1.550275345	0.637162935	1
DEPDC1	4.198922375	0.342848589	1
G9394	0	1	1
G15584	0	1	1
TREM1	2.16906855	0.609086227	1
P2RX7	-0.250896805	0.936852874	1
SHISA6	4.313929881	0.32963172	1
EFCAB3	-1.149214788	0.710129122	1
LOC505326	1.196556501	0.698450155	1
PTX4	-2.772658622	0.42774104	1
UPB1	1.551925868	0.613629419	1
HMGCLL1	6.397348733	0.099699855	1
ENSMMSG00000010131	1.142083851	0.769276348	1
HS3ST2	4.355436711	0.165249779	1
ENSBTAG00000049399	6.410401446	0.103740547	1
CD4	0	1	1
PDE6H	4.555435639	0.20198401	1

SRD5A2	4.558571952	0.302642212	1
LCMT1	0	1	1
NKX2-8	0	1	1
C14ORF178	4.969109331	0.146754731	1
ARHGAP36	-1.615565743	0.583689308	1
G2358	4.726720538	0.235237023	1
NEU4	-6.521633089	0.132691832	1
CAMK1G	1.97801417	0.626389907	1
ENSBTAG00000055216	-1.463453047	0.721313098	1
KCTD8	0.888807993	0.8115665	1
ASIC1	0	1	1
ENSMMSG00000007777	4.458668256	0.158166168	1
SELO	5.380534378	0.097335493	1
NEUROD2	0	1	1
MDH1B	0	1	1
IQCH	3.711806363	0.357668875	1
ENSBTAG00000051768	-0.633864345	0.825623389	1
TESPA1	5.21783474	0.176626495	1
CCDC60	-3.988614653	0.252132342	1
SNAPIN	0.253309266	0.917909285	1
G27590	-0.082649875	0.980937623	1
DIO3	-0.503426591	0.820332145	1
SLC22A4	6.238462817	0.157378204	1
ZNF131	1.173029411	0.655640307	1
SDR9C7	0	1	1
RPL6	1.657505914	0.686961003	1
G13860	1.410855347	0.69584419	1
MDGA2	2.740211357	0.538937665	1
BNIP5	0	1	1
G20306	0	1	1
ENSMMSG00000008468	0	1	1
RIMS2	5.23237706	0.189894763	1
TRPC3	5.867102695	0.172541167	1
HBG	0	1	1
G34714	4.136374313	0.306620239	1
KRT82	0	1	1
UBE2Z	0.719048174	0.867316875	1
KCNA6	-1.35657456	0.744698128	1
MCOLN3	4.950443959	0.262587177	1
G27992	0.498358942	0.838450632	1
ENSMMSG00000002213	0	1	1
MMP3	0	1	1
LOC100295548	0	1	1
SPACA4	0	1	1
G36733	4.952609655	0.2623766	1
GNG8	2.941261108	0.387998545	1
RGS19	0	1	1
SHC4	4.148043469	0.34880353	1
MYO5A	0	1	1
RABIT	2.806315715	0.277793331	1
G31367	1.355319712	0.668145763	1
PLCL2	0.972282858	0.761383026	1
ARHGEF6	5.815103964	0.10820264	1
FAM83B	0	1	1
MYO16	3.802594773	0.34775539	1
LPAR5	0.629082793	0.829655233	1
FAM240B	6.544079413	0.137937804	1
ENSMMSG00000000936	5.394643665	0.105943395	1

PAQR5	-0.603617126	0.873560149	1
LMX1A	-0.90059091	0.830048005	1
STPG4	5.581550899	0.133817011	1
STS	0	1	1
TRAT1	3.948438639	0.28333555	1
GLIS1	4.253663101	0.286007023	1
ENSBTAG00000051226	3.189671905	0.194277134	1
FOXL1	-0.028790332	0.991850194	1
BNC1	0.874322659	0.804680549	1
ENSMMSG00000009558	3.173838785	0.429776492	1
ENSMMSG00000020494	-0.990852896	0.746567243	1
CXCR1	-0.089905721	0.971375401	1
CFAP74	-4.441771387	0.193569491	1
KLHDC8A	0.129751102	0.974271699	1
NANOS3	-2.968775053	0.312561118	1
RNF175	3.710391573	0.347630094	1
KCNG1	0	1	1
FOXR1	1.349381625	0.765669055	1
UNKL	0.970748295	0.659492203	1
CLCNKA	1.07688049	0.760978135	1
FMR1NB	-3.295648006	0.455461461	1
TMEM52B	4.631175038	0.19198341	1
DUOXA2	3.612887389	0.374022452	1
UTF1	0	1	1
ENSMMSG00000002843	0	1	1
TRHDE	0.639148991	0.875108782	1
MATN1	-0.970662092	0.743667434	1
G29764	0.763840363	0.75879917	1
FOLR1	-1.332225911	0.684471522	1
DIO3	-0.534914626	0.818033614	1
C2H2ORF72	5.462005041	0.21607911	1
RPL27	1.015700765	0.76577858	1
SCN1A	-0.616234352	0.832547668	1
NXPE2	4.602012438	0.29800965	1
RASA3	4.949751442	0.1997642	1
CLRN1	5.009183381	0.109375193	1
MAN2B2	0	1	1
GABRA1	4.667015604	0.169795289	1
LOC785168	4.368897568	0.173900252	1
CACNA1F	0	1	1
SCRT2	3.571857296	0.420817019	1
PIK3C2A	4.338489331	0.183441955	1
ENSMMSG00000011421	0	1	1
ENSMMSG00000014470	0.329007347	0.898856285	1
G17183	2.596330687	0.560934392	1
ZNF627	0.480906213	0.892087377	1
G21836	0	1	1
SH2D1B	-0.514781394	0.852618018	1
EDIL3	-0.660532492	0.809308522	1
MAS1	-0.384131019	0.851405736	1
CHRM1	-2.094231569	0.56560622	1
BEST1	-5.458690589	0.142759561	1
TNFSF14	-1.303875261	0.742442079	1
KRT20	1.659041273	0.713212525	1
G6877	2.422491008	0.280749506	1
G11451	3.042724666	0.494131686	1
SERPINE3	3.444948649	0.40463988	1
SPIN4	0.06852774	0.981978348	1

LOC785080/LOC511136	0	1	1
G21701	4.253577201	0.28451536	1
SNCB	6.360783242	0.149360688	1
ENSBTAG00000052851	0	1	1
ADCY10	5.415854777	0.168902705	1
LOC100299025	5.672636628	0.198777335	1
DDOST	6.880327781	0.118755044	1
EWSR1	0.84474253	0.731727178	1
ENSMMSG00000000682	-3.581070304	0.144016632	1
MMS22L	1.633368738	0.585333448	1
XKR6	5.014603609	0.146111174	1
SNTN	0	1	1
ANKUB1	0	1	1
G25959	2.867694795	0.347900949	1
PRR16	5.234437852	0.235972355	1
ENSMMSG00000010778	4.426639381	0.151161518	1
BCL7A	-7.455994617	0.085290772	1
CALCR	-1.000049923	0.770207307	1
ZNF683	5.5573053	0.208120144	1
DLGAP1	0.023956038	0.992892899	1
G31559	-1.059809411	0.652687311	1
RPL12	5.807418585	0.095267159	1
PPP4C	0.232262378	0.927696846	1
MYCN	-4.098399042	0.17248677	1
ZNF182	0.97966323	0.812973992	1
NRCAM	6.766159228	0.086157705	1
GDAP1L1	-0.171460206	0.950407642	1
TOX	4.571022993	0.168016035	1
SSUH2	-0.959865088	0.817285039	1
GLDC	6.119420873	0.085243406	1
GPR35	-0.921049378	0.759714325	1
GPR151	4.305053921	0.223544472	1
DGKI	2.761058173	0.535786313	1
G24872	3.832086324	0.221429974	1
ENSBTAG00000007280	5.073636805	0.104135878	1
ENSBTAG00000002089	1.402205924	0.619222716	1
ELOB	0	1	1
GBP1	4.410962854	0.268845245	1
INSYN2B	4.13576323	0.298507832	1
LYZ	-0.431132359	0.884731343	1
DUSP4	3.283857911	0.426343718	1
S100A3	2.640472358	0.439322661	1
CCNO	1.410197694	0.66393904	1
SRARP	-0.467628434	0.83389621	1
CD79A	0.611121501	0.835730921	1
UNC13C	-0.223951405	0.943955713	1
ALK	-0.916689551	0.74074073	1
DLGAP2	6.007777436	0.153733991	1
EFCAB6	6.10108467	0.088443882	1
KRTCAP3	4.021583307	0.260178617	1
ADAD2	0	1	1
CCDC175/RTN1	0.619276899	0.869360736	1
TMEM252	5.188619293	0.113474222	1
TMEM132B	4.600249676	0.233815802	1
TMEM74B	-0.587166757	0.83189979	1
TSHR	3.744387437	0.263499422	1
RNF165	-0.398058835	0.924556525	1
HOXA1	0.126869524	0.964316781	1

ERO1B	6.457973335	0.143216691	1
TMEM151A	3.499873649	0.359936839	1
TMEM132D	3.888584226	0.380196121	1
G9108	5.313335368	0.155231825	1
ENSMMSG00000024061	1.944573951	0.665467209	1
G25038	5.441734332	0.144925933	1
CSMD2	-0.470193123	0.864809585	1
ENSBTAG00000046589	0	1	1
ENSBTAG00000045936	4.553836557	0.158706096	1
G35802	-0.6237444	0.777156015	1
CLDN25	0.936435735	0.706654385	1
ENSMMSG00000006067	5.637267896	0.131568506	1
NAALADL1	-1.557947529	0.520654126	1
PAH	4.137930832	0.349995001	1
G6850	1.944580971	0.665466045	1
OTOGL	1.656580389	0.622563795	1
RBM4	3.232865193	0.467023266	1
ECHS1	1.382727754	0.595499877	1
CA13	4.591841027	0.179780809	1
CYP1A2	2.392814019	0.427122387	1
LRMDA	4.140603235	0.289171212	1
AGPAT4	0.402024635	0.891167342	1
DNTT	0	1	1
SPRY3	4.65210015	0.270391615	1
TLR9	3.177212866	0.413434813	1
RPRM	0.715896076	0.863620345	1
COL4A4	6.941669576	0.088095971	1
KCNT1	6.023916255	0.172226553	1
CAMK4	-1.395274901	0.661166258	1
G6552	-0.017071917	0.993632261	1
TECTB	2.608001191	0.519153525	1
GPR31	0	1	1
PCDH19	-0.123289982	0.959642092	1
RAB39A	4.087419521	0.355985291	1
G24502	1.977909208	0.598537703	1
UGT1A6/UGT1A1	2.001613903	0.556889195	1
CD69	5.030488821	0.126138491	1
NKX3-1	-3.708309183	0.398706745	1
ZNF483	2.596332386	0.560934129	1
G31133	4.719289007	0.167989659	1
G6984	-0.968920097	0.670001487	1
TMEM132E	3.015534835	0.457785591	1
ZBED9	-0.956241532	0.731592999	1
NXF1	-0.988271844	0.723422382	1
G34847	-1.092972773	0.600317812	1
ACAD10	0.322365439	0.884504698	1
GDF7	3.942856863	0.373488225	1
NPB	2.791947232	0.425152159	1
ARHGAP20	2.596333782	0.560933914	1
NRXN1	5.626153671	0.124628101	1
GNPDA1	0.959472469	0.743391882	1
LOC786914	3.218723753	0.322683044	1
G26069	0	1	1
DACH2	3.543924187	0.321287096	1
DAPP1	3.827807777	0.162241666	1
KLK15	-1.182894157	0.689730155	1
PCK1	-0.60282874	0.845444417	1
POU3F1	2.028777449	0.462454762	1

LOC104974975	6.315217104	0.143533044	1
TMEM240	0.239184696	0.942682886	1
GRID1	0.885545381	0.772636535	1
LAMP3	2.533513077	0.556909706	1
TRPV1	-0.050536078	0.983722535	1
RPS28	2.752673002	0.22386894	1
EPHA5	4.556309653	0.234032676	1
SHISA3	-0.866775928	0.609584108	1
ENSMMSG00000012861	-0.745032636	0.743010519	1
G17418	1.066592093	0.734096184	1
G31301	-0.036782712	0.98480626	1
KCNQ1	3.571856535	0.42081712	1
ENSMMSG00000013300	0	1	1
P2RY8/LOC112444479	-1.815434122	0.544688825	1
FOXN1	0	1	1
WNK3	0	1	1
RASGRF1	4.577449535	0.201616856	1
PPP2R2C	4.841825279	0.200789559	1
TMEM151B	1.649200606	0.642365417	1
ADORA2B	5.201861867	0.166026473	1
KCNAB1	-0.077348685	0.969416324	1
VSTM1	0.056600345	0.984836545	1
ENSMMSG00000000630	4.466369871	0.312634471	1
SPSB4	-0.173325536	0.939288108	1
GRM7	5.82530936	0.124876101	1
POU2F2	4.470765442	0.129793145	1
DOC2A	-0.688471613	0.840946008	1
KLHL4	-0.222831594	0.908774523	1
CYP8B1	5.344854316	0.095764624	1
G10831	-4.512388143	0.30135156	1
HYDIN	0	1	1
G18420	0	1	1
OPRL1	4.258620111	0.250404654	1
DNAH8	1.250758265	0.717073532	1
DCAF10	-0.009134998	0.997408048	1
FAM186B	0.075408878	0.977344016	1
SORCS1	3.888565595	0.380198436	1
HMGXB4	0.962813937	0.647940887	1
ADRA2C	0.645870503	0.798922875	1
G33161	0	1	1
G16257	2.676993534	0.486791281	1
LOC618826	4.211313506	0.286497479	1
ILDR2	-0.169082054	0.948336762	1
G29892	0	1	1
IL22RA1	2.637529848	0.43419198	1
EDAR	3.086084091	0.347785135	1
RPS21	-0.38262947	0.837588457	1
CRYBB1	-0.445824124	0.89009771	1
GRXCR2	-4.512425433	0.301347565	1
FAM163A	0.64888882	0.798993935	1
UTS2R	-2.238012337	0.382040369	1
G19225	-3.715335611	0.310245331	1
ENSBTAG00000000728	-1.05773625	0.784414947	1
TMEM163	1.008599834	0.733339925	1
G24981	2.640302681	0.427309527	1
ENSMMSG00000010048	3.408830847	0.259597769	1
NHS	4.002539683	0.229280158	1
C5H22ORF23	0.940892938	0.778214542	1

MGC134105	-0.702445864	0.851014644	1
SP110	3.305183225	0.3278783	1
SLC12A5	0	1	1
LOC508086	4.315686063	0.189744843	1
TMEM219	2.596333176	0.560934007	1
C22H3ORF18	-0.754198846	0.729865208	1
RAB37	0.190394629	0.939808628	1
GPR132	0.119616481	0.969380547	1
CFAP206	3.962010828	0.332539525	1
TRAM1L1	0.146950681	0.956803564	1
GTPBP2	-0.543006222	0.804099027	1
ENSMMSG00000006737	1.693726847	0.645385737	1
MEGF11	3.748305793	0.349384458	1
REC114	1.326460681	0.558283894	1
ATP4A	0	1	1
TRPA1	4.13035179	0.273654555	1
G29084	1.011163774	0.683550932	1
GPR25	1.216686752	0.681230693	1
TSKS	3.817899908	0.307591293	1
CLDN2	0.534744221	0.813678121	1
PRSS35	-0.384375815	0.91212516	1
TOX2	-3.299872812	0.317055522	1
SDCBP	6.017167269	0.14688789	1
G9204	4.203118908	0.160504831	1
G9999	2.440992916	0.524555602	1
RPSA	1.837518308	0.421752947	1
SEZ6L	3.455748189	0.43633051	1
CCL4	5.388737552	0.169087832	1
ASIC2	-1.46045995	0.618093702	1
KCNC3	2.596359583	0.560929931	1
LHCGR	0.088977223	0.967453538	1
G35822	0	1	1
ARHGEF11	1.257436993	0.626176881	1
UBASH3A	-0.544256484	0.837599326	1
G9238	6.019126422	0.148005761	1
TTC21A	-5.697434053	0.169524486	1
ENSMMSG00000019567	-1.051393517	0.637597558	1
EDA	-0.858841025	0.807779597	1
B3GNT7	0.813683476	0.746188956	1
TLL2	-0.507423719	0.858640209	1
FAM155A	6.02055845	0.093181174	1
ENSBTAG00000030189	5.659069554	0.120996713	1
CROCC2	0.507075266	0.904186861	1
CCL3	5.595756727	0.204970261	1
ADAMTS6	4.72858609	0.2847858	1
G27179	2.90879235	0.513720806	1
ZAN	2.613630567	0.367532493	1
COL11A1	-3.519993297	0.320727765	1
ARHGDIB	-0.684974067	0.681077037	1
PRSS42	0.777580151	0.709264866	1
ASIC4	1.297744292	0.584865417	1
FABP7	0.180912768	0.950465105	1
ENSMMSG00000012792	0.417307031	0.89473498	1
TSSK6	4.757538494	0.110464	1
CACNA1G	4.558559651	0.229037961	1
LINGO2	2.645213794	0.52862664	1
MGC139164	-1.322147687	0.619223422	1
CCDC86	0.557796134	0.797635674	1

CATSPERD	1.710443634	0.617191159	1
FUT6	0	1	1
LOC508834	3.120844193	0.481534696	1
DNAH5	6.101400097	0.096220053	1
TMEM151B	0.477011221	0.907714716	1
BTBD8	5.296545227	0.114425159	1
LOC618268	0	1	1
AKR1C4	-4.592033095	0.184646063	1
STRBP	-0.078632103	0.972530755	1
FAM166A	3.455768076	0.238364749	1
IGSF11	1.336201477	0.604350027	1
SYT5	0	1	1
TIGD3	2.950762461	0.267417289	1
GPR63	4.505548071	0.097713127	1
SALL4	6.586306077	0.095249726	1
MYRFL	6.675616415	0.13016613	1
CFAP300	4.951344564	0.109191357	1
PGLYRP3	0	1	1
SRD5A2	5.168469864	0.241976073	1
TMEM229A	0	1	1
CYP2C18	-4.290837885	0.321816756	1
FSTL4	0	1	1
ENSBTAG00000000027	0	1	1
G33078	5.647566298	0.188907038	1
PLA2G4B	1.588550784	0.533783902	1
ECEL1	-3.988992993	0.259734231	1
PROKR1	-0.463440409	0.863153723	1
RIMS1	3.474528191	0.36256443	1
PRMT9	3.879595804	0.172590242	1
CD38	-1.131472293	0.543748819	1
PRKG2	-0.663137218	0.785129242	1
LRRC10B	0.22546048	0.9514912	1
KCNJ15	5.690967809	0.14169598	1
ZNF165	1.772458959	0.401663539	1
GDPD4	0	1	1
ABCB11	1.108598025	0.750497427	1
G18737	4.452908405	0.146072484	1
MEP1B	4.865384706	0.112472029	1
ENSBTAG00000002629	0	1	1
FOXA1	0	1	1
LOC618463	4.371548247	0.278084417	1
ENSMMSG00000005462	2.549449116	0.465812711	1
SPACA9	0.751295593	0.782725597	1
ENSBTAG00000049596	-0.490173031	0.815280829	1
CFAP141	-1.949796455	0.630376564	1
G19336	0.05108376	0.979170023	1
ENSMMSG00000001100	0.813986831	0.781080729	1
PHYHIP	3.119582253	0.474452069	1
ATP6AP1L	0	1	1
G36932	-0.032722224	0.987477955	1
FGF22	2.373088981	0.485035322	1
SLC51B	0.705018204	0.746689645	1
GSTA2	-2.095184073	0.448552818	1
TMEM230	2.999699502	0.318341244	1
ENSBTAG00000053696	6.736377528	0.088694945	1
ZIM2	6.067979049	0.085193164	1
GNG13	0	1	1
C13H20ORF202	0	1	1

DCC	4.262178689	0.335537207	1
G31909	3.278184798	0.460686449	1
ACTBL2	3.120059211	0.477197954	1
INTU	6.509243504	0.140055044	1
GDF5	0.321180544	0.896485789	1
EXOSC1	-0.867461842	0.614960927	1
STAB2	4.493045021	0.131720387	1
TMPRSS6	0	1	1
HSP90AA1	-0.786142953	0.798704942	1
CC2D2A	-0.008373701	0.997463911	1
ITGAX	5.671619271	0.192803219	1
NCKAP5	4.461769488	0.23073753	1
SLC6A9	0	1	1
SCN5A	-4.706233539	0.203479261	1
LOC511498	-0.274333607	0.915613428	1
SDS	1.0567488	0.60657619	1
FBXO4	0.010329129	0.996148699	1
KRTAP3-1	0	1	1
GRM1	0	1	1
KCNH1	2.637643429	0.433933475	1
NODAL	3.451135939	0.379764836	1
ENSBTAG00000035777	5.311833684	0.08849565	1
GRIA1	3.110286798	0.429753828	1
ARL5C	0.166848239	0.940280828	1
INTS9	1.024423333	0.640511389	1
FSCN2	-0.584305977	0.781712546	1
TMEM147	0.379290034	0.836704411	1
BOD1	-0.391475631	0.853895892	1
LOC522428	1.012488808	0.642436334	1
AQP5/AQP6	2.470072351	0.436810862	1
LOC104974812	4.488940446	0.167406951	1
GYG2	3.0144426	0.290413556	1
ENSMMSG00000014172	0	1	1
LIPF	0	1	1
POLR3D	0.408463228	0.868977256	1
DPH2	2.266495491	0.471191921	1
CCR8	-0.031043748	0.986210938	1
G22107	-1.248321579	0.660591592	1
MAG	-0.41569984	0.834416442	1
ATG7	4.913346363	0.184988416	1
CYP2J2	0.617338184	0.747755908	1
CCDC188	-1.26983793	0.526044717	1
BOLA-DOA	-4.807355461	0.142528827	1
G34244	-4.572349771	0.101221474	1
IRAK4	0.395420676	0.891372711	1
CCDC184	4.349221085	0.227255378	1
TREML1	0.571011325	0.847497516	1
ADGRG5	0	1	1
ENSBTAG00000055295	4.296112948	0.201810201	1
G38487	0.262535319	0.918091181	1
PTGER2	0.699111517	0.817944021	1
CD79B	0.14608385	0.944801275	1
TMEM200C	-1.541376204	0.621550689	1
ALDH3A1	5.231173121	0.168899695	1
KRT71/KRT74	0	1	1
PTGES	0.858551338	0.660914378	1
CDH26	-0.846359836	0.780792024	1
ZNF335	1.499756206	0.512953482	1

CCR2	-0.270859731	0.913390716	1
ZNF157	-0.474492321	0.839815933	1
GPR20	5.690811519	0.137870954	1
G8591	1.643739289	0.620666865	1
NINL	5.316695892	0.2116323	1
NPTX2	4.320886545	0.173120002	1
ANKS1B	4.2943194	0.191092285	1
C17H22ORF31	5.316517118	0.207304771	1
MYO3B	0	1	1
LHFPL5	2.948576463	0.366348715	1
KRT81	0	1	1
ASGR1	5.05211228	0.148596285	1
KCTD16	-0.981679189	0.645174836	1
MMACHC	-0.707045818	0.676031806	1
ENSBTAG00000015464	-0.176947025	0.944523218	1
LRRC55	-0.242135539	0.907458843	1
G8702	0	1	1
TAGLN2	5.768185342	0.105611055	1
PCNX2	-0.707119729	0.832553418	1
CFAP43	3.04272787	0.494131222	1
FAM189A1	0	1	1
SPX	1.134091786	0.642463117	1
HTR3B	-5.501210719	0.092354956	1
CPQ	-0.83216718	0.818929259	1
STIL	3.056813104	0.432172885	1
OPN1SW	3.894980718	0.214481767	1
CD8B	1.158216293	0.698851523	1
PRDM6	2.612407658	0.505023826	1
LRGUK	1.974851015	0.604208895	1
GPBAR1	2.155291123	0.450749447	1
TLL1	0.097168039	0.967798105	1
ADM5	2.58981833	0.24024438	1
CALB2	0.117853827	0.962316335	1
IKZF1	0	1	1
PRND	-3.235626635	0.327736683	1
G18787	0	1	1
TMPRSS11F	0	1	1
ENSMMSG00000005241	-0.23283981	0.923343386	1
ZSCAN25	-1.0014508	0.730230597	1
NFASC	5.617374784	0.203214748	1
G10404	4.598011876	0.210380502	1
PNPLA1	5.232689167	0.195427489	1
LOC508646	0.721000721	0.843697187	1
SNX9	-1.341795758	0.506439583	1
ADCY1	6.056616435	0.141722347	1
LOC100139209	5.58331665	0.197694158	1
KCNH2	-1.752742962	0.538838004	1
LCP1	0.858909746	0.743090762	1
FBLL1	0.117875596	0.973373818	1
DYNLT4	-0.411049609	0.859926234	1
SLC45A1	1.783350618	0.466018158	1
ENSMMSG00000018571	2.487531205	0.408860514	1
WDR83OS	-0.817540345	0.797338172	1
PPP1R3G	-0.750394367	0.747168068	1
G13563	-0.758139475	0.789802875	1
SLC5A5	3.978165998	0.225325318	1
LGALS2	5.037648889	0.087010406	1
LHFPL7	2.410259954	0.589996561	1

PADI4	-0.349989616	0.890456193	1
ENSMMSG00000008560	4.075321514	0.217747061	1
ROS1	3.523940129	0.281601199	1
CAMK4	1.450974164	0.499020805	1
LHX1	-0.249793547	0.904746151	1
LOC528040	-0.808975443	0.64600613	1
ANO3	2.779472333	0.464838259	1
HCFC1R1	1.916466983	0.496967569	1
TMEM108	-0.460318009	0.836261512	1
KCNC2	0	1	1
COL9A3	1.416492501	0.550631618	1
VSTM2L	0.272892536	0.915108718	1
SLC36A3	2.644687292	0.515453139	1
ENSMMSG00000017065	-1.814770569	0.611916426	1
LHX6	-0.894118357	0.643450041	1
GRP	-1.686084597	0.55045347	1
G14492	-0.305480511	0.899813267	1
GPHA2	-0.251852853	0.941727634	1
CIP2A	-0.325024741	0.87617571	1
TMIGD3/ADORA3	4.534207045	0.190511431	1
DISP2	5.465033587	0.115703372	1
VPS13A	0.925455749	0.697494873	1
LOC522479	0	1	1
ACTL10	4.979220671	0.145194907	1
G3255	1.723424938	0.542880087	1
UBD	-0.529625283	0.75657673	1
G7925	1.677688656	0.483199174	1
KCNT1	0	1	1
CAPN12	4.647048973	0.172113061	1
RTKN2	1.767195559	0.383363222	1
GCNT3	4.843023486	0.142451298	1
C15H11ORF91	2.186159687	0.482257505	1
ENSBTAG00000000432	0.205983592	0.932669012	1
UPK1B	1.445726665	0.561590807	1
ZNF793	-1.283696944	0.555348717	1
ENSMMSG00000013246	4.731825158	0.106436288	1
SCAP	5.440676359	0.12567969	1
KRT28	0	1	1
SERPINA7	1.65514595	0.672241219	1
G6556	0	1	1
TASL	-0.960401061	0.714113539	1
ATP8A2	0	1	1
CACNA1E	5.862300804	0.095152796	1
EFHC2	4.910994774	0.137785148	1
TNFRSF13B	0.644712666	0.854038212	1
ENSBTAG00000048888	-2.070422157	0.456752582	1
CHD3	6.852293388	0.089675895	1
GRM2	-4.536220206	0.102823047	1
MOBP	4.879794112	0.131001239	1
ITIH5	-0.019916051	0.991419867	1
SLC44A2	0	1	1
LOC530077	5.708902981	0.164129044	1
ENSMMSG00000023504	-0.876783482	0.783626935	1
LOC532207	0	1	1
REN	-2.178580039	0.379570831	1
BCL2	5.102479138	0.083297973	1
PLEKHA7	0	1	1
SOGA3	3.063951259	0.345635178	1

LYPD4	1.376165982	0.71686513	1
SYAP1	1.255467637	0.556118489	1
NOTUM	-5.270323371	0.129663592	1
BICD1	-0.630097781	0.801198277	1
GPC5	5.334853554	0.146619266	1
STX1B	-3.98944044	0.269771272	1
ZDHHC3	1.088215803	0.703903804	1
TM4SF5	0	1	1
KCNH1	2.600398315	0.346012894	1
EDAR	-3.300824692	0.363538143	1
ENSMMSG00000005528	2.964256876	0.372064362	1
SNX20	-0.153649154	0.951216453	1
TMEM169	-2.957328461	0.35289365	1
HAVCR2	1.879210661	0.507630487	1
CDH10	-1.499417881	0.672371963	1
ZNF10	5.606369265	0.153089863	1
MAK	4.741512361	0.102946577	1
G31870	-0.504163008	0.763038622	1
NOP53	-0.44644792	0.848300683	1
C15H11ORF94	-0.154727731	0.948170468	1
FAM216B	5.701025834	0.18609239	1
LRRC52	0.006823719	0.997990406	1
CELF4	3.856878288	0.220411416	1
CP	5.168916593	0.085695028	1
LINGO4	2.745190301	0.33759568	1
KLHL35	1.779324525	0.486870058	1
LVRN	6.130082814	0.164752285	1
ENSBTAG00000021066	0.7652352	0.799639509	1
G7817	5.294052637	0.193112792	1
EFCC1	-0.172676943	0.944902894	1
TSPAN1	4.528662521	0.115554013	1
ENSMMSG00000003335	4.672464457	0.103781479	1
AHSP	-0.428407072	0.879302259	1
SPATC1L	0	1	1
TLR5	1.733290923	0.582046888	1
C8H9ORF43	2.732171908	0.254606953	1
CCDC62	0.292623687	0.907846717	1
PTGDR	-3.25911158	0.300089523	1
ENSMMSG00000024286	1.976742273	0.481404676	1
RPL23A	1.647098934	0.337073645	1
EPX	1.052309703	0.658670728	1
SAA1	3.669847995	0.324065869	1
PRDM9	3.895160469	0.212848776	1
G16206	0.742032756	0.803000256	1
G20836	2.012809187	0.389656153	1
CCL5	4.117376825	0.194177714	1
DOCK11	5.488490318	0.121725221	1
SPOCK2	1.169519237	0.619807152	1
TEX38	2.670754217	0.199086374	1
KCNE4	-0.670735693	0.697866564	1
LHX1	0.476173609	0.849337512	1
TPM3	2.744972959	0.273638288	1
G2117	5.229787349	0.137815525	1
SLC16A4	0.676678863	0.735216784	1
SOHLH2	5.334565559	0.082562939	1
SLC27A6	0.524819358	0.87387311	1
SFTA2	0	1	1
KCNIP3	0	1	1

IQCF1	-0.953852595	0.795854718	1
ZBED3	5.363329948	0.103798773	1
SUPT20H	0	1	1
GRIN3A	2.400039272	0.235702972	1
NAA40	-2.328151265	0.510212605	1
PIWIL2	0.203671603	0.939156795	1
DMRTA2	0	1	1
PRKAA1	0.401270544	0.817911564	1
PLAG1	5.07023728	0.084016712	1
ACMSD	2.619143356	0.484325462	1
DNAH5	5.440586815	0.122087663	1
STAP1	4.048611025	0.15628527	1
HCN3	4.291542906	0.107802395	1
PPBP	4.913114258	0.265992434	1
FAM169B	-0.179545961	0.944741772	1
SLC1A7	3.02923107	0.306956765	1
CDC14B	0.781519824	0.710996479	1
SAMD15	-0.710578253	0.67892255	1
TRABD2B	-0.139252064	0.957117661	1
AMZ1	-0.591459064	0.800910389	1
SLC5A5	4.481115396	0.170798069	1
UROC1	2.436739984	0.513110016	1
ARHGAP15	-0.011002326	0.996511603	1
CPA5	3.386952848	0.16801085	1
DMXL2	5.142393166	0.145205348	1
LOC100335205	2.218939223	0.500844991	1
PCNX2	1.413277481	0.590426156	1
RNF207	4.588445017	0.156033689	1
G2955	0.166271237	0.945920331	1
ITK	5.131952295	0.091261436	1
RND1	-1.449100455	0.437362681	1
NLRP1	1.020370223	0.665938728	1
TRIM71	0	1	1
SGSM1	4.349902613	0.295605905	1
ERC2	4.461987269	0.293842372	1
HELB	-0.168320395	0.933529204	1
ASIC1	-1.348653858	0.593533372	1
LOC524771	0	1	1
DEDD	-1.104743437	0.525636547	1
ENSMMSG00000012869	0	1	1
CD6	-1.247853705	0.61651965	1
RIMBP3B	-0.941800567	0.741800606	1
NOX4	0.045369967	0.98431835	1
G7366	5.843675396	0.093302402	1
COL28A1	4.316663187	0.32046237	1
CLEC4E	-0.869593593	0.763364195	1
CCR5	4.687383565	0.153288498	1
APCDD1L	0.512779747	0.855947673	1
DSG4	0	1	1
HCN1	-0.842750567	0.751846082	1
RBMS3	-0.489467714	0.844913956	1
C18H16ORF86	3.767147922	0.177416959	1
MFSD4B	-0.162479717	0.946559619	1
DAAM2	-0.375618936	0.819628647	1
RPL23A	2.790633269	0.187730177	1
SNTG1	0.275166201	0.917892678	1
PLCH1	4.117411872	0.194338521	1
SPIB	-0.993339687	0.778787299	1

G30140	3.207928403	0.265383836	1
G27756	-0.788402744	0.749686751	1
CEND1	1.17775053	0.596898981	1
ENSBTAG00000048693	0.055394265	0.982997604	1
ENSMMSG00000010261	4.291411431	0.175576247	1
LIPK	0	1	1
CFAP61	5.142561108	0.169632977	1
SKA1	4.315982225	0.329398933	1
PBLD	0	1	1
MYBL1	0.469454786	0.866145613	1
ASPHD2	-0.400822725	0.874842632	1
TMEM253	3.06022428	0.412696769	1
PEX5L	0.310395507	0.882819658	1
DYNLL2	0.3307168	0.852598915	1
NCAM2	-3.300656023	0.366130509	1
G9370	4.355566163	0.234546932	1
ENSMMSG00000001369	0	1	1
FLT3	5.266407816	0.094654773	1
PREX2	5.843644955	0.089383518	1
ENSBTAG00000027727	-0.041978859	0.983596614	1
CALR	2.716079666	0.212938415	1
KCNJ6	0.452114373	0.835567395	1
ENSMMSG00000020040	1.082474172	0.617977105	1
CXCL17	3.412450104	0.26925373	1
DHH	0.005751415	0.997679082	1
ADAMTS6	0.144652112	0.954859531	1
KCNE3	0.053750837	0.973002773	1
SH3GL1	-1.30284991	0.598709044	1
BPIFB2	0	1	1
CCNB3	0	1	1
ENSMMSG00000011810	0	1	1
COL2A1	-4.143241963	0.183254933	1
PPIA	4.125136045	0.145622095	1
RNF138	5.645670412	0.101594308	1
ENSBTAG00000031682	0	1	1
SSTR1	-0.327876439	0.865977214	1
YY1	6.008123937	0.155135721	1
TFAP2E	2.283662724	0.347488058	1
PRRG4	2.665044806	0.372416006	1
SLC15A2	0	1	1
CLDN20	-0.223835145	0.915385693	1
LIF	-0.373656286	0.890150916	1
AWAT2	3.27211599	0.270118915	1
LIN7A	-0.258958428	0.909953689	1
EEF1A1P5	-0.752295241	0.751986643	1
KLF8	5.557808635	0.110027232	1
NEIL2	0.695298129	0.729172819	1
RBPJL	3.458106519	0.288441382	1
EPHA7	3.600824574	0.416998218	1
HRH2	0	1	1
CELF2	-0.337525593	0.860526505	1
RALGPS2	4.307615054	0.302223711	1
NLRC3	0.26596607	0.928458818	1
RPL23A	0.841011299	0.59142851	1
DMBT1	6.030415496	0.107188147	1
SEPTIN3	1.120947335	0.630263883	1
4/LOC104968446/H2AC10/I	5.114372131	0.156909954	1
COL2A1	-2.129893843	0.272133892	1

CSMD2	-0.719117068	0.718433679	1
RNF138	0.9338073	0.712690563	1
PPP1R14B	0.889451949	0.726871867	1
EPB42	1.183770451	0.721536249	1
TPC3	-0.047272953	0.982146386	1
CACNA1H	5.050160166	0.149046914	1
ENSBTAG00000030922	1.270442671	0.639470216	1
G24632	3.107793006	0.298380583	1
G7372	3.11634184	0.457831683	1
ZNF536	2.335619439	0.431779029	1
HEPACAM2	0	1	1
CLEC4D	2.378836469	0.469205328	1
SAPCD2	-1.459551933	0.502303498	1
RGS3	0.461026027	0.83212597	1
ANKRD61	0.726390581	0.699302805	1
ARMC12	-0.215465277	0.917076073	1
NLGN4X	0	1	1
G28051	1.387084999	0.698990458	1
C20H5ORF34	-0.803098126	0.738446882	1
EMX1	4.263888001	0.107400178	1
ENSBTAG00000026637	-0.292567243	0.901180373	1
GLP1R	1.823278551	0.51693544	1
FXYD2	-0.377210128	0.805760003	1
DCHS2	-4.993997073	0.155276168	1
G13525	1.539424713	0.468460662	1
ITGAM	-0.295503052	0.878297582	1
ENSBTAG00000006141	0.285202196	0.929543642	1
ADRA1B	1.096504451	0.602654796	1
CACNA1C	-5.696955796	0.132550294	1
TG	0.382868354	0.875576774	1
UTP4	0.692408605	0.768543012	1
GSN	0.327900135	0.893590855	1
ZC3H12B	4.766707415	0.17380428	1
STAT4	0.249395458	0.911902164	1
DNM3	0.914225344	0.626979878	1
SH3GL3	-0.253821041	0.91342318	1
MPP3	1.571492032	0.393187846	1
G137	1.449992101	0.593783652	1
CLP1	-0.622239089	0.739138155	1
SPIN2B	-0.186542363	0.924498804	1
RPL12	2.918658854	0.264489792	1
PIWIL4	4.912460297	0.135095869	1
GLB1L2	5.041777772	0.11093085	1
ENSMMSG00000023554	1.833562833	0.391588537	1
KCNK2	1.833766019	0.466104895	1
HIVEP3	-6.389974581	0.101218691	1
C25H16ORF54	-0.007016144	0.997618102	1
ENSBTAG00000020155	2.643591612	0.491565903	1
NEFM	1.102463938	0.63155307	1
SCRT2	-1.451521225	0.51227953	1
SCN3A	-2.312427911	0.428791255	1
H3F3B/H3-3B/H3-3A	2.774322552	0.279115716	1
RASSF5	0.475472169	0.862411069	1
ADGRF1	0.625977908	0.840801784	1
G36403	0.384045167	0.851333594	1
EBF2	-0.96459935	0.608022268	1
PRIMA1	3.212362236	0.288345889	1
MAB21L4	0	1	1

RPL23A	0.847878239	0.638928415	1
G25829	0.566104667	0.765782691	1
IGSF1	-1.702560941	0.444522519	1
RGSL1	-0.686200439	0.792151943	1
DGKG	1.107014255	0.641879186	1
ADORA2B	-0.750518178	0.65337597	1
GPR19	-0.348689513	0.83758365	1
TTLL11	-0.268509287	0.893131811	1
XYLT1	0.017088778	0.992774678	1
SLITRK2	-0.005554894	0.997770896	1
CCL27	1.662502236	0.444936994	1
C1H21ORF62	4.324824702	0.236667359	1
MFSD2A	4.943063863	0.134758882	1
GGTA1	5.842999083	0.087536114	1
NR5A2	-1.490569117	0.475195398	1
AREG	5.331399828	0.082575375	1
SRD5A1	3.404963472	0.250070329	1
NOS1AP	-0.701436475	0.772399627	1
DLX5	0	1	1
FOXC2	-1.023370222	0.684816856	1
MASTL	-0.247289558	0.945996026	1
SMIM17	-0.315993689	0.872939857	1
G33760	0.677000289	0.732970916	1
ZNF655	0.436975303	0.83587051	1
RALGPS2	5.365497826	0.125032538	1
MARCHF1	4.881235525	0.130391356	1
LRRC34	4.168316264	0.140363242	1
TACR2	-1.597069551	0.558690539	1
PRDM16	0.031968164	0.986508209	1
ZNF263	1.630265251	0.414371042	1
ENSMMSG00000017344	0.313604483	0.90293122	1
ITIH2	0.286426688	0.916380601	1
KCNH3	1.867613264	0.537828061	1
ENSMMSG00000015083	0.441098489	0.829498488	1
PBX4	2.757436395	0.399464059	1
SLITRK1	-0.838363928	0.615045662	1
FAM43B	0.343403645	0.883881888	1
LHX6	-0.791062506	0.703175923	1
TRIM6	2.828947326	0.333475701	1
GPR156	2.746017723	0.202789153	1
IFIT3	0.465990958	0.844840818	1
ENSMMSG00000014611	-0.783245889	0.72070923	1
G23366	-0.754723283	0.749322335	1
G19643	5.624548898	0.098256111	1
DLGAP2	-0.80031301	0.793084682	1
LRRC3	1.683585438	0.529097586	1
PAX7	-0.856520361	0.698865128	1
TLCD5	-0.466351086	0.824522059	1
NTSR2	-0.037538202	0.988989888	1
DRAP1	3.12365523	0.235857239	1
PHKA2	5.393843288	0.175163903	1
RPGRIP1	4.32575333	0.122034914	1
ATP6V1FNB	0.201834063	0.932973579	1
PDE1C	-0.984288419	0.666678701	1
HES7	2.06153831	0.429914607	1
CPVL	-0.098409391	0.96541212	1
LGR5	0.345272107	0.900967816	1
CNTNAP4	0.613176192	0.797322164	1

ILDR2	-0.825259074	0.603082489	1
RAB44	0.208952597	0.915487033	1
LGMN	0.213797261	0.877217833	1
CATSPER1	0	1	1
DCX	1.372964005	0.489001124	1
ANKEF1	4.473643453	0.227539739	1
PLA2G12B	5.338131338	0.09226218	1
ENSMMSG00000004153	-0.397170598	0.848655947	1
GRAP2	-0.517470307	0.800653679	1
ARHGAP22	-0.593620843	0.803846418	1
CD3E	1.354449525	0.477318161	1
G17872	2.00094811	0.49727512	1
LOC518080	0.382178114	0.864861976	1
SBSPON	-0.08473136	0.971497738	1
PDE8A	5.878803133	0.093930566	1
CCK	3.912622589	0.249475083	1
ENSMMSG00000002093	0	1	1
PTX3	-1.122702633	0.626475963	1
ENSMMSG00000000549	4.948121723	0.124316344	1
PRR11	0	1	1
RSAD2	2.587080257	0.294036069	1
CFAP126	0.465302824	0.825982254	1
IKZF3	4.070894567	0.238512752	1
UGT1A1/UGT1A6	0	1	1
ENSMMSG00000009641	-0.231984562	0.882155415	1
ENSMMSG00000015077	5.230073317	0.15260663	1
LGALS1	0	1	1
CHSY3	-0.96804367	0.686859561	1
MYH15	0.69144202	0.795885218	1
FAM185A	0.257667238	0.89713408	1
ENSMMSG00000001372	5.440506872	0.109047198	1
MMEL1	3.672013841	0.311775347	1
REELD1	1.27143847	0.505088003	1
OAZ1	0.251389287	0.864830616	1
G1648	4.201171663	0.132420248	1
HOXA13	-0.550400904	0.836467437	1
LIPG	-0.662129114	0.736051672	1
G1185	0	1	1
TMEM130	0	1	1
CEACAM18	1.62423531	0.568002699	1
CRB1	-0.512092244	0.798534235	1
CHN2	2.700974091	0.306508481	1
RBM15B	-1.434722877	0.487803751	1
PALD1	-2.855216069	0.400932272	1
MMP24	2.93471566	0.286347526	1
RTN1/CCDC175	1.316660649	0.601492913	1
APOBEC3H	0.279634944	0.884931057	1
ENSMMSG00000000010	-0.991812154	0.668008968	1
C8H9ORF153	-0.318044666	0.870895687	1
MYCN	-3.829807894	0.151531841	1
ZNF711	0.43956225	0.830000269	1
FOXP3	1.491804472	0.543187686	1
AMHR2	4.634879999	0.08748573	1
TCF19	0.490046117	0.862402924	1
ADAMTS17	0.826305633	0.736742297	1
RSRP1	2.29670575	0.236026225	1
PCYT1B	-3.712700737	0.297215602	1
CNR1	4.049366607	0.120187812	1

PCLO	2.287520425	0.435737702	1
LOC107131722	-0.836944485	0.736330135	1
ENTPD8	3.987099963	0.186702955	1
PLEKHH1	-0.122149746	0.960987798	1
HOXA2	-0.784407351	0.63664543	1
ENSMMSG00000001919	-0.070791916	0.980413307	1
MEX3A	1.066431327	0.619824873	1
C9ORF78	0.818072171	0.628858192	1
KCNH8	0	1	1
TNFSF13B/LOC107131142	1.26129464	0.598075771	1
ENSMMSG00000015376	0.66805713	0.789373241	1
RAMP3	1.743962564	0.463173832	1
ADAMTS19	0.816017847	0.728495612	1
YBX1	0.364490724	0.900387779	1
RGS7	0.844167953	0.7528549	1
GPR18	1.480276111	0.474124056	1
TG	2.619941127	0.481964677	1
HCAR1	0	1	1
NLRP6	2.018023558	0.501964101	1
MYO16	0.270520791	0.937607	1
NADK	0.139455156	0.943982188	1
ATP2B4	-1.371514751	0.71661537	1
G5320	-0.900974205	0.732642452	1
MAGI2	4.198841076	0.216031093	1
ATP6V1E2	3.189623386	0.174150718	1
ENSBTAG00000019919	2.482076313	0.413324508	1
LOC618071	1.17056597	0.61209936	1
RPS14	0.048375461	0.973155563	1
PELI2	0.251745387	0.905041414	1
CLDN10	1.552087718	0.348673279	1
ENSMMSG00000002450	3.398206632	0.356895262	1
ASIC3	0	1	1
HOXB8	-1.574466207	0.412084818	1
GPC6	3.144404209	0.243072005	1
PPP1R36	-0.21509702	0.891540977	1
DIPK1C	4.551876752	0.121982606	1
CCL26	0.486530537	0.833282304	1
SCEL	0	1	1
CFAP161	4.586254137	0.099723587	1
SLC17A7	0	1	1
TNFSF15	5.669293112	0.118457644	1
HOXC5	0.859218905	0.583126344	1
HPDL	0.60066619	0.69115299	1
ENSMMSG00000015458	-0.683764683	0.663676916	1
ENSBTAG00000053113	0.152279187	0.947371968	1
SLA	0.321782746	0.890076646	1
DLG1	0.874225759	0.742845879	1
ENSBTAG00000051250	-0.80918587	0.674024309	1
UGT8	-0.615384868	0.801530317	1
NXPH3	4.288270731	0.160989902	1
RSPH10B	-0.554830771	0.774174649	1
KIF18A	3.307956799	0.3176799	1
IL25	0.663146474	0.767674828	1
BTBD8	0.527916024	0.82951224	1
MREG	2.262224599	0.427932976	1
G13773	-0.499339053	0.793308741	1
JAZF1	-0.357103034	0.894198054	1
CCR4	-0.939140379	0.64721998	1

NOS3	-1.262296811	0.539313007	1
ST8SIA1	0	1	1
RFLNA	0.309318365	0.873643023	1
ENSBTAG00000054693	0	1	1
ELAPOR2	-1.040804538	0.632756323	1
LOC532114	-0.127135977	0.936974384	1
DLGAP1	-0.198524738	0.914392353	1
ENSBTAG00000050395	3.219615422	0.269640471	1
EVC2	-0.227788033	0.904120164	1
LRRIQ1	2.923276396	0.450494599	1
NET1	-0.163943802	0.921537692	1
G28667	3.913359895	0.194278101	1
IKZF1	-0.125759282	0.959864359	1
RPL10A	-1.33279521	0.651105561	1
ERFL	4.648628716	0.24419256	1
ENSMMSG00000003017	2.221493458	0.343563901	1
PCP4L1	0.037261924	0.980547641	1
BBOX1	0	1	1
RPL37A-PS1	2.852229767	0.11089945	1
ABCA12	0	1	1
MYRIP	2.546847337	0.294401542	1
GIMAP7	-0.470185558	0.838107119	1
ENSMMSG00000012310	0.748818812	0.702129161	1
EREG	0	1	1
SIGLEC8	-0.735413264	0.674538114	1
SLC35F2	0.459324889	0.797266015	1
ANKRD61	1.120605579	0.625673435	1
MASTL	3.61425641	0.215789377	1
PFDN2	0	1	1
SPHKAP	0.859651792	0.747472614	1
METTL4	0.22083211	0.908183676	1
ARHGAP42	4.319691555	0.100001511	1
ENSMMSG00000012539	0.000222287	0.999932328	1
ENPP6	-3.987880535	0.238224499	1
XKRX	3.544104988	0.182184958	1
NEFH	4.231298557	0.16042397	1
G6451	-0.066527873	0.978064216	1
MAD2L2	1.04957019	0.490813942	1
G2957	-1.069021285	0.453862449	1
OPA3	3.039894081	0.161370649	1
TPRG1	2.699866231	0.254427314	1
MGC157368	0	1	1
ENSMMSG00000021273	-0.037983598	0.984075497	1
G7728	0.226803439	0.942303346	1
ARSH	0	1	1
STXBP5L	3.737409089	0.159864586	1
G2E3	1.206255742	0.544232721	1
ADGRG3	0.643605333	0.814064922	1
LOC784914	-0.579011461	0.75931992	1
CCBE1	0.18156461	0.938287645	1
ENSMMSG00000020335	1.180083384	0.602149892	1
IIST1H3B/H3C1/HIST1H2AIL	3.099442647	0.388182361	1
KCNE1	2.104842042	0.411920622	1
H2BC11	1.096319897	0.53374908	1
PPEF2	-0.957944146	0.530966481	1
CD38	-0.890980337	0.728493701	1
KLHDC1	0.149480175	0.950162595	1
ENSBTAG00000003408	2.954470106	0.158149834	1

ENSMMSG00000019664	-0.145331407	0.945774726	1
ADPRM	0.281719632	0.865314959	1
SLC25A53	-0.612377484	0.649316611	1
MPO	0	1	1
G3548	3.205936431	0.243397976	1
ENSMMSG00000023294	-0.145986024	0.92517609	1
UQCC2	-0.712988994	0.54898995	1
HEATR9	0	1	1
RUFY2	-0.792419146	0.585493243	1
HNF4A	3.070270077	0.375270946	1
ENSBTAG00000054988	0.899754623	0.6706812	1
GFRA3	-0.321310289	0.868021121	1
SELL	0.050462208	0.977483447	1
BLVRA	1.056131494	0.565082975	1
SLC5A10	2.766278728	0.364741149	1
LRRC55	0.924946837	0.729990164	1
TOGARAM2	3.861484318	0.197306039	1
SLC4A10	4.722714257	0.200529224	1
GAS2L3	4.101809479	0.184585975	1
SNX30	-1.099689095	0.65905283	1
YRDC	-0.045170215	0.977698872	1
ENSMMSG00000023293	4.341600719	0.11604229	1
CHIC1	0.939403264	0.629370834	1
PLA2G12A	1.134606875	0.597645136	1
G1370	5.227458729	0.122068493	1
G12012	1.449416138	0.61099571	1
CPNE6	0	1	1
NEIL3	4.0709054	0.130751958	1
ECRG4	-0.137873177	0.9527826	1
C13H20ORF96	0.876288655	0.613451912	1
PRDM7	3.053633689	0.280501901	1
ANKS6	-0.771476524	0.756458268	1
ECT2L	2.373496842	0.386565846	1
KCNQ5	-0.417254141	0.83674381	1
GFRA4	-0.180748941	0.92859363	1
SPATA6L	0.840125237	0.71460419	1
ENSBTAG00000026527	2.045518438	0.453906145	1
ENSMMSG00000006253	0.615657828	0.68553003	1
PSMG3	0.229131707	0.916522339	1
KRT73	-3.571913171	0.335290995	1
SUN3	-0.059203471	0.979934491	1
RPS15A	1.295630426	0.59811074	1
WNT16	4.366627758	0.098496346	1
ZNF132	0.75908821	0.71671137	1
SPRN	3.645313259	0.146630923	1
SCT	0	1	1
ENSMMSG00000010128	2.593306901	0.366416771	1
HDC	1.444966073	0.456514992	1
RNASE13	-0.302588347	0.835154137	1
ATP8A2	-0.618599368	0.764471533	1
ENSBTAG00000054505	-0.260757811	0.913504715	1
CEP170	-0.19318297	0.921932083	1
CYP26B1	2.132726095	0.30904421	1
1500009L16RIK	0.800558565	0.677807229	1
SLC5A9	0	1	1
STIL	1.914868306	0.476139664	1
ASMT	0.628240221	0.839203626	1
A4GNT	4.846506789	0.123997471	1

DLGAP3	-0.365043918	0.820428862	1
LYSB	0.273964333	0.907952159	1
ACRBP	-1.422066626	0.591645154	1
ZDHHC16	0.392691517	0.888472616	1
EEF1A1	-1.634300062	0.570748045	1
ASF1B	3.72386949	0.151255811	1
HTR1B	-1.921804983	0.555994767	1
ZNF365	1.173256489	0.599496606	1
ARF2	-0.012627176	0.993959249	1
FBXO48	0.449304462	0.771118888	1
IL21R	0.020474059	0.988675327	1
G38493	4.948450444	0.135017187	1
ENSMMSG00000017910	4.043006115	0.135426428	1
IFIT2	1.78455148	0.46162832	1
DNER	-3.168960271	0.167389846	1
KIF5C	-1.132668754	0.581036531	1
PRRT4	0.487718396	0.846211684	1
NEDD4L	-0.141831286	0.96324866	1
ICAM4	1.573808383	0.538672095	1
G27180	1.324470632	0.582533919	1
IGSF23	0.34066714	0.838646882	1
CTXN1/TIMM44	0	1	1
SFXN2	-0.28207256	0.863079049	1
PCSK2	-0.929562464	0.708997874	1
CACNA1B	-0.836361471	0.644827106	1
RNF183	0.154001525	0.948866702	1
IL16	4.300125766	0.229549826	1
UBL4A	0.024919415	0.985663537	1
PTGDR2	-1.013671039	0.555841436	1
COQ2	0.59690424	0.724794962	1
GRIN2A	3.430487213	0.330542099	1
REC8	0.664649858	0.754958291	1
ASAP1	-0.965744029	0.579822123	1
TMEM132C	1.007706607	0.657859995	1
TLR8	-0.441644445	0.819931157	1
IL12RB1	-0.055333597	0.974418218	1
EDA	-1.235457676	0.465972177	1
FECH	0.427101027	0.797789689	1
PABPC5	0.349681908	0.836579037	1
GNAI1	0.822507736	0.671173372	1
LRRC4B	-0.886029454	0.68336047	1
KIF20B	4.721527036	0.18821694	1
CBLN3	-0.21407336	0.891374256	1
ENSBTAG00000052124	0.428758085	0.829771108	1
ENSMMSG00000012408	-2.472094147	0.422189003	1
SNCG	0.297686187	0.836545319	1
EVC2	1.693472758	0.557267097	1
CHRNA5	1.037526444	0.644306772	1
CD300H	-0.115241536	0.953621383	1
TRPV3	3.777519984	0.194709087	1
GCNT1	0.569233328	0.799187694	1
ENSMMSG00000009324	1.381143389	0.496410312	1
TSSK1B	4.669011278	0.086099983	1
HOXA4	3.429971963	0.328403816	1
PLK5	0	1	1
PABPN1L	1.672313047	0.5368883	1
ATP10A	3.249808836	0.213090525	1
HNRNPA1	0.185376158	0.901838845	1

GATA6	-0.633651793	0.749465578	1
ENSMMSG00000021623	-0.711000143	0.75259526	1
MIOX	4.57709262	0.103996577	1
FCGR2A	0.971734274	0.723677234	1
MIS18A	2.689950376	0.326573119	1
ENSMMSG00000007851	-0.629313346	0.736697769	1
IL2RA	-0.591467798	0.694333706	1
USH1C	-1.759397772	0.497202782	1
ENSMMSG00000014313	0.210642823	0.914834669	1
TMEM216	0.221555892	0.919383363	1
NPAS3	-0.719247194	0.761275323	1
AJAP1	-0.907477982	0.573395915	1
GP5	0.693242315	0.72343407	1
ENSMMSG00000002257	0	1	1
G7305	-0.080581876	0.973956993	1
ARTN	2.078834113	0.23123408	1
PSD2	-0.295057742	0.911096554	1
ADAMTSL1	0.412666755	0.846427736	1
OPTC	0.299595587	0.895760808	1
TAX1BP3	-1.151660947	0.572099279	1
RPL13	-0.580318724	0.680170916	1
KNOP1	0.612306828	0.737031318	1
NOL4L	4.724220838	0.21813156	1
SLC13A3	1.677754487	0.365736046	1
RSPH14	5.135461554	0.099498244	1
MC4R	-3.701653285	0.221258834	1
CXCR2	3.910661877	0.151423161	1
MATN4	0.063844745	0.971441384	1
MICAL3	-1.555324248	0.604450485	1
CD300E	1.708400573	0.461283337	1
FUT7	-0.461073662	0.832567126	1
ENSMMSG00000008608	-0.170893697	0.924488646	1
ELOVL2	-0.191741362	0.931526624	1
MOV10L1	1.009552586	0.670606445	1
G17793	1.89652578	0.230269568	1
G11877	0	1	1
PGLYRP2	0	1	1
CELF5	2.658222449	0.440646918	1
ASPM	3.215892917	0.279027019	1
ZNF599	-0.59777418	0.788446024	1
ALAS2	1.604471164	0.520593751	1
CAMK1D	-0.832756319	0.740106085	1
DPP6	3.111323868	0.265136994	1
TTK	2.790848931	0.42847392	1
CSMD2	-1.057456723	0.500162562	1
INF2	1.28750362	0.448269134	1
G21994	2.056462416	0.586237311	1
TMC1	-2.015123541	0.451333346	1
SKA3	3.617590495	0.206422998	1
SYTL2	0.31342995	0.920962074	1
CA12	1.000377511	0.597657324	1
DIS3L2	0.350412584	0.881560518	1
DUOXA1	0	1	1
G37304	3.706559142	0.261894091	1
DIRAS1	0.459465592	0.849726654	1
G29877	1.108787955	0.64468536	1
G4682	3.678090699	0.20965125	1
CDHR3	-0.564586897	0.767083058	1

SKIDA1	0.120641433	0.951188965	1
RLBP1	0.282022223	0.899346154	1
NOXO1	-0.265535519	0.88921997	1
NYAP2	5.140605922	0.119532605	1
LY6E	1.199653426	0.578142237	1
EEF1A1	1.724616856	0.359224415	1
ENSBTAG00000003527	0.144988297	0.927326351	1
SERPINC1	1.823543942	0.408297347	1
HROB	0.957671839	0.572879154	1
ENSMMSG00000003903	-0.2410052	0.90317785	1
KL	0.977307091	0.675966245	1
TAL2	0.301259462	0.861796256	1
PRR16	0.507800989	0.782139532	1
HDAC1	-0.416413642	0.784599179	1
IL7R	0.161854969	0.946505797	1
G8477	0.083526256	0.972522171	1
SLC25A47	3.219509391	0.265243041	1
ASPA	-0.815983463	0.531915019	1
G10534	0	1	1
NUDT11	1.624665766	0.568897608	1
CCT8L2	0	1	1
FOXH1	2.203404413	0.347421428	1
FGF18	1.559155679	0.55456234	1
EIF5A2	0.078531364	0.968194346	1
ROBO3	-0.568599175	0.773919561	1
GDPD2	-0.046216154	0.983506103	1
CNTN3	1.513093056	0.445090406	1
TUBA3	1.276956626	0.565750985	1
KCNA3	1.717836943	0.350233454	1
GUCA2A	2.610744814	0.363952144	1
ENSMMSG00000013075	3.256660495	0.196584542	1
DKKL1	3.938951506	0.128040256	1
HNRNPA1	-0.469475137	0.711534432	1
ENSMMSG00000009949	0.679475981	0.672771608	1
G32628	2.065505513	0.437432585	1
IRF4	0.639707437	0.738890005	1
CSNK1G1	-0.382728526	0.833399466	1
GPM6A	0.0043941	0.99812996	1
IQCK	1.316645907	0.508939293	1
KCND2	4.301216543	0.23830722	1
PPM1E	5.640896261	0.147557873	1
ENSBTAG00000035171	0	1	1
ENSMMSG00000023657	0.603487283	0.772684956	1
RGS4	0.974706253	0.550020012	1
ALKBH8	0.04736574	0.97981579	1
MRPL2	1.450261892	0.367961588	1
HTRA3	-0.865617569	0.511782034	1
MXD3	1.324707472	0.476240011	1
PCDHA13	0.184136561	0.906254545	1
SLC16A12	1.584036383	0.423275937	1
G33275	-0.057917372	0.969554902	1
CHRNA3	0.062301554	0.974746296	1
ZNF2	0.7390609	0.712619223	1
ZNF181	1.394571347	0.37438795	1
SGO2	0.005473424	0.997245907	1
ALOX5AP	0.391442591	0.795717865	1
IRGC	0.259888642	0.882127353	1
DBX1	-0.231824506	0.870963284	1

ENSBTAG00000027760	-0.406420483	0.858463103	1
VCAM1	-0.744354619	0.620508165	1
TTC16	3.90822712	0.178179569	1
METTL24	-0.430985317	0.822539412	1
RALGPS2	1.14274161	0.646868248	1
FCGR3A	0.346466075	0.860963271	1
RGS18	2.071533273	0.297690217	1
LANCL3	0.372733602	0.842118028	1
LOC539818	1.730905132	0.416855124	1
SOX5	5.390788783	0.101961488	1
MROH8	1.111539937	0.57707575	1
CDKL2	0.132217855	0.94897024	1
LRR1	1.521605578	0.481347809	1
TLCD3B	-1.985736628	0.319081712	1
ENSBTAG00000040222	-0.49636188	0.838629737	1
ENSMMSG00000005389	3.232251131	0.240975855	1
CCL19	1.318412195	0.583564194	1
AMACR	-0.55426354	0.797489139	1
ISLR2	-0.985268922	0.5257394	1
SLITRK5	-0.942427273	0.508762561	1
TSSK2	1.410196335	0.426338662	1
BATF2	0.086355647	0.960997307	1
FAM124B	-0.374193851	0.891149052	1
ATP6V1G2	0.353036552	0.852372989	1
CCDC89	-0.734841184	0.697963295	1
SLC9B2	0.053223204	0.973315679	1
CXXC4	4.251025159	0.105910955	1
RNF19A	0.151554255	0.931725833	1
RNASE1	-0.200997354	0.910233191	1
PAQR9	0.062909063	0.97036585	1
PRPF39	0.078333219	0.966681581	1
G14133	4.439366004	0.127199621	1
NRCAM	2.18515803	0.376132508	1
ENSMMSG00000000115	0.193599704	0.913744572	1
FOXC1	0.722534871	0.546474198	1
G25403	1.451396136	0.351875152	1
FCER2	-0.728729358	0.616536129	1
NINL	-0.141042961	0.957703953	1
RSPO2	0	1	1
CYP2R1	3.172313372	0.180995383	1
SLC24A4	0	1	1
TRA2A	2.314246114	0.155156367	1
ATP6V1B2	-0.334302107	0.801774136	1
C20H5ORF49	-0.191092621	0.923510493	1
PLEK	0.589446243	0.767389196	1
LOC529823	2.820883233	0.189275803	1
ASTL	-0.798492148	0.580974608	1
SPAG4	1.271651117	0.55946869	1
XBP1	-0.04234288	0.977755087	1
MCM10	-0.54373799	0.843476319	1
KRT36	-3.992753123	0.362240586	1
TEX14	-0.72507878	0.769031926	1
SCG5	0.981573504	0.661932091	1
G22880	-0.700814438	0.653290349	1
CDH1	0	1	1
SLURP1	2.322860329	0.38321907	1
RNF24	2.342732532	0.307270482	1
GNGT2	-0.674418836	0.604026965	1

RNF227	3.93567792	0.133666034	1
RNF220	1.953319485	0.412823037	1
APBA2	0.024076699	0.987644849	1
CD80	-0.361095614	0.81870375	1
CHST11	-0.897269053	0.632926832	1
ANGPT2	-0.821839521	0.653626118	1
LRRC71	0.002510164	0.998527761	1
TCFL5	0.226654937	0.895259084	1
RPL37A-PS1	1.287840311	0.43514128	1
GAS2L2	0.511985374	0.707443327	1
G4327	-0.274048196	0.865155792	1
NAGS	-0.484237337	0.702366497	1
TBC1D2B	-1.003687687	0.563572222	1
LIPK	0	1	1
IHO1	0.477744845	0.80315972	1
SYDE2	-0.214881687	0.908703536	1
G30336	0	1	1
RND1	-1.034943964	0.573908223	1
LOC510613	0.095905439	0.956322552	1
ADCYAP1R1	0.021690527	0.990598986	1
ZBTB8A	-0.425996323	0.8139188	1
MYL6	0.913604962	0.621769101	1
LOC530973	1.065972496	0.612408663	1
G36145	0.547332331	0.849126927	1
TENT4A	0.919839186	0.63668144	1
CDC25C	0.614852837	0.788481398	1
NUP210	-0.602659475	0.705444804	1
ZCWPW2	-0.134753357	0.935094951	1
CREG2	0.435645715	0.779372537	1
NSG1	-0.725952061	0.626080251	1
ENSMMSG00000004299	0.333285829	0.863588539	1
GRIA3	1.373155467	0.520516243	1
G35601	0.816185262	0.658953715	1
HSP90AB1	1.245791328	0.443948665	1
EIF4EBP3	-0.487981109	0.743654248	1
ADM2	2.979894287	0.291182018	1
RPP25	0.580533256	0.810157464	1
MOG	1.059072274	0.575911675	1
ADAMTS6	0.61972011	0.699968002	1
CAMK1D	1.16257332	0.556817722	1
G28775	1.185501909	0.668466019	1
TLL1	-0.454097564	0.800980419	1
C23H6ORF141	1.271562911	0.425106501	1
G36142	1.014679073	0.767708483	1
G6571	-1.075280853	0.44284851	1
LOC101901911	-0.641653145	0.693582955	1
ENSMMSG00000005095	0.746900333	0.706542928	1
HOGA1	0.784322693	0.635352015	1
G20509	-0.792741728	0.612158158	1
ADPRM	0.690655628	0.676218287	1
SPNS3	2.350855355	0.307323612	1
IFITM10	-0.555693788	0.742623541	1
'HIST1H2BC/LOC100441051	0.72945554	0.754006082	1
HOXB5	0.101007974	0.968842888	1
SCN7A	0.446755722	0.830371684	1
DNAJC6	2.055693765	0.286765845	1
CNOT8	-0.231853632	0.886234897	1
ENSMMSG00000012941	1.86266674	0.353724934	1

UCK2	1.411106522	0.504820392	1
KRT9	0	1	1
LOC112449560	-0.175936422	0.938360567	1
RHOH	-0.628843133	0.738932327	1
ANAPC10	0.330514095	0.78370986	1
IRAG1	-1.995081832	0.46865399	1
DRC7	0	1	1
PEG10	-0.575513706	0.705943768	1
G25849	-0.066017187	0.954591329	1
NOVA1	-0.877779407	0.445523301	1
LOC526787	1.040055976	0.637056418	1
PAG8	-0.193454032	0.920990579	1
ENSMMSG00000020744	-0.357913013	0.794424256	1
TTC28	-0.496101737	0.723466441	1
HTRA4	-0.599558716	0.760751138	1
G26854	0.450299993	0.810883663	1
BAALC	-0.732641491	0.605296006	1
KCNN4	-1.018305083	0.631907507	1
TERB2	-0.157114977	0.920000237	1
SUSD5	1.5982134	0.342763818	1
KIF21B	-0.406780867	0.789237706	1
CABP7	-0.161505458	0.929951247	1
CACNA2D3	0.382949303	0.870998435	1
MR1	0.91660032	0.635190266	1
G34585	0.340329295	0.852082984	1
MTARC1	0.330028654	0.879482579	1
GPR68	0.325009086	0.836746395	1
ENSMMSG00000004941	-0.274413765	0.847639516	1
KCNK3	0.990627764	0.684367813	1
ENSBTAG00000053298	0.226513698	0.923101333	1
SCML2	-0.652343693	0.620542173	1
PMP2	2.052647644	0.36048754	1
EBI3	2.014334436	0.282157777	1
RPL7	0.108673161	0.941134194	1
BOLL	-1.046543275	0.541284811	1
CSPG4B	-0.550547405	0.759669573	1
LARP1B	-0.653214348	0.637801645	1
ARHGAP15	0.515222758	0.825741375	1
ENSMMSG00000021955	-0.329763162	0.825182079	1
NEFH	0	1	1
MYO1A	0.372243286	0.869851374	1
RPL7L1	-0.305075123	0.842093995	1
G5734	1.058152315	0.575986224	1
FBXO15	-0.100147952	0.964814051	1
LOC614914	0.853893912	0.619877682	1
CARMIL3	1.704142486	0.461404808	1
CXHXORF58	-0.990574368	0.464428168	1
KRT89	0	1	1
G24982	-1.810901221	0.619104572	1
BDNF	0.713066168	0.760308484	1
VWA5B2	2.237938064	0.363479152	1
SLC19A3	1.17104228	0.657993125	1
THUMPD2	0.578700018	0.777271321	1
RASGEF1C	0.261501658	0.908599044	1
ADORA2B	-0.814205644	0.629329644	1
GPR179	0.472440438	0.776019753	1
C6H4ORF19	0.470917067	0.80338542	1
AP3M2	-0.227304728	0.898993291	1

ARHGAP6	-0.910662701	0.435921156	1
BCAP29	-0.305282991	0.873802362	1
PADI3	2.695839465	0.22015615	1
NPTX1	-0.545241676	0.782152712	1
C2CD4D	3.226637041	0.192895425	1
WDR31	2.129761977	0.206643651	1
ZNF793	-0.073040342	0.972329713	1
MAML2	0.785873257	0.684436787	1
G595	-1.472700104	0.414470287	1
NPHS1	0	1	1
PBRM1	0.100827404	0.956973808	1
SPRED3	0.948738933	0.715386977	1
SP6	0.012286573	0.996023139	1
ASTN1	0	1	1
HOXB8	0.452725289	0.823328583	1
TM4SF19	-0.426354468	0.789030293	1
ZNF496	0.117024673	0.957135117	1
EFCC1	-0.031269368	0.981895654	1
STX11	-0.900511501	0.564020667	1
CHGB	-0.023259876	0.989867228	1
UXT	1.222396334	0.45981398	1
G33513	-0.916399128	0.713178871	1
GPC2	1.467755333	0.491278987	1
ENSBTAG00000040318	0	1	1
ENSBTAG00000053719	4.192446476	0.191207637	1
RT1-BB	3.344411067	0.218033477	1
RPL23A	-0.097165937	0.959323171	1
ENSMMSG00000020868	-0.472676237	0.838344224	1
ACTL7A	-0.754308514	0.641758268	1
BICC1	0.787952763	0.738058583	1
ENSMMSG00000006206	0.74750327	0.564469731	1
ZNF461	-0.349417949	0.840767983	1
PMEL	-0.08780767	0.95091769	1
CPNE5	-4.296198152	0.174260367	1
EOLA1	2.211817504	0.456172663	1
LOC530653	4.106243613	0.182084546	1
TTLL6	-0.157040147	0.925870832	1
CABP1	0.805187472	0.730983211	1
EFCAB8	-0.50379856	0.745165428	1
CD5	2.750776201	0.15643511	1
MPPED2	1.331247388	0.532214766	1
ABCB1	0.989878691	0.619418916	1
TEKT3	4.784615276	0.124437123	1
PIF1	-0.63272027	0.720576384	1
PGF	0.72837667	0.705507756	1
PIPOX	3.537808658	0.195719888	1
L3MBTL1	0	1	1
SAMD13	0.395579554	0.815712913	1
PPP1R14B	0.281506641	0.870375166	1
G12606	0.521561824	0.785829237	1
ABHD15	0.027600163	0.987012554	1
LOC536097	-0.516912459	0.725283028	1
TNRC6C	3.314975314	0.227817613	1
MIA	-0.072216545	0.957519211	1
LAX1	0.568508241	0.686765244	1
SLC28A2	-0.796372328	0.719601314	1
GLTPD2	1.137299408	0.460771342	1
ASMTL	0.612846454	0.744274625	1

SLC6A17	0.598125553	0.755506294	1
FBXO10	-0.437519493	0.755018738	1
BDA20	4.819499916	0.193013829	1
NRXN1	2.906335494	0.293476835	1
H1-1	3.443260263	0.208654761	1
HRH2	0.288650008	0.888027588	1
CEP128	0.005410545	0.99777634	1
ZNF813	0.976407024	0.595638937	1
MEFV	1.074743259	0.616619188	1
GSKIP	-0.162643626	0.912633471	1
SPATA18	0	1	1
ZC2HC1B	0.852542682	0.668587129	1
ZNF829	1.7151878	0.448346843	1
G5311	4.578329793	0.087565492	1
LOC509810	0.41064961	0.790956659	1
EPHA3	-0.45965332	0.761694708	1
OCM	0.605651511	0.637637262	1
IQSEC2	1.830911155	0.363252503	1
HS3ST5	-0.767619854	0.699708146	1
GGT7	5.395123276	0.085530876	1
CHRNA7	0.357478899	0.879259833	1
LOC510913	-0.144908767	0.910136755	1
LRRC66	0.774103499	0.77559202	1
PRSS12	0.787825353	0.66452054	1
ZGRF1	5.534568441	0.085462917	1
ITPKA	1.136358207	0.556987667	1
ENSMMSG00000016901	0.512396638	0.717171581	1
ALOX5	1.032518841	0.56253015	1
GARIN3	-0.568193339	0.696638985	1
EPHB1	-0.5703193	0.754878446	1
CYP46A1	-1.927924917	0.553114408	1
PTAFR	-0.084578177	0.965346498	1
PIG	0.367753174	0.888044965	1
ACTL7B	-1.329272894	0.342589681	1
MYO7B	1.128682685	0.594791523	1
KCNA2	0.68793855	0.799356977	1
LOC112441479	-0.183491588	0.949636527	1
NKAIN1	-0.51554703	0.69605296	1
RBPMS2	1.169364616	0.401772866	1
C19H17ORF49	0.704089485	0.694364344	1
ENSMMSG00000009887	1.802458598	0.498665007	1
KCNJ10	2.486403468	0.374010108	1
SNAI1	-1.166849032	0.507248367	1
GNAT1	0.727061332	0.719895515	1
DMTF1	-0.200881583	0.934708285	1
CACNA2D3	1.575827671	0.495523208	1
SERPINB6	2.318997615	0.394544243	1
CCNL1	-0.0900483	0.957938805	1
G5536	4.330133813	0.198065801	1
TMTC1	0.841932357	0.656509651	1
ADRA1A	-0.315691204	0.884753983	1
FBXO10	-0.434119052	0.799784352	1
PLA2G2F	3.815976187	0.225954302	1
G23385	0.633552249	0.777204289	1
COL9A2	2.071079459	0.454071691	1
G30756	1.083542863	0.481152324	1
GUCY1B1	-1.075698331	0.418453756	1
PDS5B	-0.79280639	0.582346204	1

SOX5	-1.045228637	0.5685125	1
HNRNPA1	-0.341439789	0.798495	1
CACNA2D3	1.728622198	0.417478863	1
GIPC2	-1.025813474	0.461158741	1
ERICH4	-0.734801304	0.761333145	1
CLDN15	1.124247177	0.52055718	1
NAT9	0.431774283	0.753967076	1
PTGIR	0.577652795	0.624819971	1
MKX	-0.846476557	0.710955113	1
ZNF548	1.087906219	0.580625447	1
TRAPPC12	-0.25540179	0.89123976	1
AGBL5	1.789131697	0.480040592	1
UBA1	0.193224035	0.917262585	1
ENSMMSG00000008143	3.154113135	0.193337176	1
MMP17	-0.126389217	0.938602954	1
FRMPD4	-0.876509442	0.605063852	1
G4528	0	1	1
LOC112441539/SEC14L6	0	1	1
RFX3	-0.285043481	0.900642692	1
ECHDC2	-0.915296493	0.584634734	1
ENSMMSG00000008023	2.359909516	0.21970278	1
RPL19	0.460148289	0.720350531	1
BEGAIN	-0.426761108	0.743288643	1
NIM1K	0.405679461	0.84024488	1
CYP21/CYP21A2	-0.189089238	0.911206934	1
AMPH	0.544724181	0.696749441	1
FBN2	-0.914181849	0.544680434	1
PBX3	-0.895715538	0.60592982	1
ZNF2	1.78429402	0.461789366	1
MSH2	0.027124039	0.988467787	1
HECTD4	-1.062674546	0.591720486	1
CHRNA2	0.205018966	0.876688866	1
ENPEP	-0.983729844	0.566001919	1
PTCHD4	-0.057991179	0.977188153	1
ENO2	2.589855951	0.232248221	1
THPO	0.467081939	0.737850716	1
WDR48	0.525766693	0.743718356	1
G35574	2.666526853	0.211231792	1
POU6F1	-0.460490983	0.841908665	1
GRIN2D	-0.724886352	0.664763778	1
NRP2	-0.701638001	0.701067655	1
TLL2	-0.985178992	0.438607374	1
HIPK4	0.801718332	0.57994351	1
TCF7L1	-0.265211377	0.885460929	1
ENSMMSG00000000696	-0.353781131	0.81084704	1
HS6ST2	-0.511105208	0.721119549	1
GARIN1B	1.233356265	0.450918693	1
G8102	-0.747211667	0.63026631	1
EEF1A1	1.140809795	0.570548485	1
CX3CR1	0.54471757	0.752612135	1
G2991	-0.124552085	0.940601858	1
NRIP2	-0.728983569	0.647298461	1
ENSBTAG00000050865	-0.532931231	0.728202325	1
FBXO41	1.670664246	0.396466865	1
ENSBTAG00000054370	-0.516486668	0.707925506	1
RHNO1	0.091735972	0.964598116	1
NPC1L1	0.226361328	0.856265977	1
TDRD9	0.551647211	0.741682196	1

CGRRF1	0.112376997	0.93841224	1
ELANE	0.357759494	0.829473748	1
ZSCAN31	0.369082787	0.789036325	1
TEAD1	-0.648427128	0.635949293	1
CTHRC1	0.06566981	0.97246958	1
G16027	0.712912407	0.635812591	1
SV2B	-0.90269193	0.543236188	1
ENSMMSG00000010230	1.541774296	0.304358907	1
BCL11B	3.596103856	0.279530775	1
LOC101905222	3.831901494	0.113444631	1
HCRTR1	-0.298724267	0.836551355	1
PLCXD3	3.94199979	0.140875837	1
LIX1	0.49931163	0.816688526	1
ASTN2	2.558689769	0.343010501	1
VANGL2	1.086315557	0.631600106	1
DBR1	-0.210833102	0.883750838	1
FSVMD	2.321516247	0.379635868	1
LCT	2.80723257	0.307700775	1
TMPRSS11E	0	1	1
PEG10	-0.706195778	0.788228719	1
LMX1B	0	1	1
MANEAL	0.10676574	0.946689766	1
ENSMMSG00000008310	-0.355513907	0.823948452	1
HOXA11	-0.786595905	0.6944762	1
DRAM1	-0.103293833	0.951255483	1
PLPPR1	-1.068103167	0.542350337	1
VSIG1	0.927169168	0.578929536	1
P2RX1	2.842887093	0.199802362	1
NLGN4X	4.144470057	0.159562311	1
SYT3	0.610961628	0.795414646	1
ACAN	1.540199646	0.525532448	1
ENSBTAG00000054041	0.112343629	0.943997283	1
TTYH2	2.302853982	0.368493823	1
IRAG2	0.121232203	0.949625128	1
BCL2A1	-0.857880465	0.65633584	1
ENSMMSG00000008183	0	1	1
GRIN2A	0	1	1
COLEC10	-1.022710036	0.535670457	1
IGF2BP2	0.255996463	0.917953162	1
KIRREL3	-0.637852398	0.643828657	1
DENND6A	0.363540082	0.828237599	1
MYBPHL	-0.079364078	0.958485962	1
UBAP1	-0.454605889	0.720520848	1
TAS1R3	-0.095552966	0.96078687	1
RPS11	0	1	1
PRR5L	-0.04074261	0.983694049	1
NGFR	0.606086404	0.708139269	1
ENSBTAG00000018009	3.032756711	0.136831985	1
DNAAF4	-0.7540278	0.672684443	1
G17650	3.438984079	0.202919572	1
DCLK2	0	1	1
KLRB1	-0.527479836	0.783669794	1
ENSMMSG00000014617	1.345923714	0.385398603	1
GRM3	-1.099417977	0.386251728	1
KIF14	-1.668044953	0.346278171	1
FAM131B	-0.97085772	0.547160163	1
ADAM22	-0.709014588	0.677317903	1
TRABD2B	-0.291048216	0.861070906	1

NEU2	0	1	1
LMX1B	0.904842414	0.702606733	1
TICRR	1.139536688	0.596634701	1
G12047	-0.245478635	0.856874455	1
IL6	2.852441935	0.278676815	1
ZBTB37	0.365404305	0.871204421	1
LY9	0.652009839	0.760611008	1
CDK2	0.244719453	0.877173641	1
CHODL	-0.086392988	0.961292123	1
FANK1	-0.855994166	0.729786631	1
MEIS2	2.668149961	0.155615018	1
SPATA17	1.455321161	0.633686516	1
ZFP2	0.163098197	0.897346008	1
MTUS2	0.377479152	0.852072234	1
PTPRO	1.204734831	0.597470831	1
G23537	0	1	1
DOK3	0.557701165	0.745050285	1
G20045	1.302192143	0.443711848	1
CYP7B1	-0.492608047	0.73144675	1
SCG3	1.128751832	0.399501894	1
GPR62	-0.537310409	0.705587497	1
G27694	0.511521206	0.820494224	1
GFRA2	0.258107633	0.878495296	1
ZNF567	0.019133901	0.990049699	1
LOC407171	-0.829762939	0.602881103	1
CDCA8	3.446315734	0.202063418	1
PPM1N	1.436564967	0.457243707	1
FHL5	-0.601821147	0.747477745	1
UCHL1	0.541654527	0.779337954	1
TDRD10	-0.304332441	0.842927085	1
ENSMMSG00000005417	0.726859191	0.670955713	1
ADAM23	-0.813174114	0.703355412	1
ILRUN	-0.557809833	0.645596705	1
G14678	-0.353454173	0.856627712	1
MYCL	-0.607276947	0.796325644	1
RPL10	2.39668426	0.16250455	1
G10402	2.696031663	0.31959864	1
G2588	2.195582172	0.236481899	1
GAREM2	0.368810805	0.831782128	1
GDNF	-0.650803783	0.748520819	1
BLK	1.084945717	0.494583326	1
PPP1R3E	1.550748375	0.327996162	1
TUBB3	2.954009094	0.262204947	1
RNF112	-1.486457316	0.521369356	1
ENSMMSG00000007631	1.672565191	0.398363824	1
SMOC1	-0.436933768	0.763182943	1
MAP1LC3C	-1.950980098	0.426893959	1
G9022	0.429835721	0.850591116	1
GRIFIN	0	1	1
KIAA0825	0.856997257	0.71650347	1
AVP	0	1	1
ENSMMSG00000023260	-0.550710257	0.738645942	1
GPR45	1.867645644	0.289852238	1
OLR1	0.444411221	0.807514511	1
EIF5	-0.596805669	0.678329824	1
DNM3	-0.789171763	0.676002374	1
SLC26A9	2.026182425	0.26313015	1
IL2RG	-0.201426613	0.878977563	1

ZNF713	0.406407702	0.82892309	1
POLE2	0.92655483	0.590755645	1
C21H14ORF132	0.727247409	0.726938496	1
ADAMTS12	2.374430752	0.22887971	1
SULT1C4	0.758955847	0.698148988	1
ZSCAN2	-0.620943288	0.684128108	1
CDCA7	1.36135358	0.482087047	1
PXDC1	-0.834611811	0.59629675	1
G15945	0.054370437	0.975052923	1
PGLYRP1	1.536738981	0.517678149	1
OIP5	2.917187396	0.261573972	1
ACVR1C	0.66887973	0.768384839	1
ADA2	0.794096393	0.676080605	1
G22171	1.420985972	0.429982058	1
SYNGR3	2.304273147	0.195826369	1
EEF1A1	1.516355198	0.344716544	1
ENSBTAG00000049278	-1.046709746	0.493144869	1
BGLAP	0.120496783	0.937376141	1
EPM2A	-1.250246691	0.424424906	1
SLAMF7	4.931597675	0.089446786	1
CDH2	-0.244843341	0.866069974	1
BOLL	-0.606102369	0.793912179	1
SLC26A7	0.171474166	0.915014078	1
ENSBTAG00000052097	-3.65400236	0.194063989	1
KIR2DL5A	-0.742076629	0.638263892	1
RNF152	0.060451311	0.964490401	1
BICD1	-0.902922245	0.584190718	1
MAL	-0.012471521	0.99505147	1
CENPK	-0.091208538	0.959891922	1
LMBR1	0.649826717	0.573927141	1
PHKA2	0.184668849	0.951072478	1
H3-5	1.642726168	0.256220973	1
LIN52	-0.719182817	0.566162738	1
EEF1A1	5.31313921	0.145016031	1
G15250	1.281653989	0.60853589	1
ENSMMSG00000024210	0.89291904	0.590306386	1
LOC520336	-0.783309496	0.685215368	1
ATAD5	1.547992704	0.427506034	1
ENSMMSG00000001823	1.686528572	0.384751801	1
ST6GAL2	-0.700955366	0.710761479	1
UBAP1L	-0.807060513	0.592078963	1
P2RY14	-0.985507867	0.508613672	1
EEF1A1	2.812820695	0.218094649	1
MAB21L2	-0.886187163	0.667410243	1
ENTR1	1.973213765	0.186903979	1
SLC38A5	4.052136329	0.108850037	1
KRT35	0	1	1
MORN3	0.644664183	0.726349486	1
PLCL1	-0.230551392	0.893265204	1
GINS2	0.543587574	0.790123505	1
TAL1	-0.827975035	0.523967239	1
LENEP	1.357665289	0.399537374	1
GPC6	-0.528334157	0.775411975	1
CCL11	2.156559756	0.317298491	1
ENSMMSG00000015602	1.566463564	0.187927853	1
PDIA2/ARHGDIG	4.409466896	0.086873045	1
KRT78	2.626510442	0.323354891	1
VCPKMT	-0.031466854	0.983291925	1

TBXA2R	-0.868458313	0.544810261	1
GAPDH	-0.101118364	0.955162432	1
ZFP30	0.405725969	0.794146213	1
TGM6	0	1	1
G20146	0.752817739	0.709231359	1
SNAP47	-0.208108108	0.890214091	1
ENSMMSG00000006205	-0.37875253	0.826369909	1
KIF19	3.156934457	0.250402897	1
ENSBTAG00000046728	4.836614072	0.159784245	1
GRIN2A	0	1	1
G29098	0.929811038	0.526076858	1
MSC	-1.060998611	0.538940752	1
SYT15	-0.060260473	0.963911113	1
G24980	-0.182787187	0.902135993	1
SPATA25	-0.455832254	0.774383307	1
NLRP12	0.774442833	0.700120863	1
TTC23	1.813983553	0.304382101	1
CRLF1	0.877477881	0.673060602	1
FAM162B	1.278429947	0.34725856	1
STRC	1.141069981	0.385653563	1
ENSMMSG00000008372	2.345507169	0.273188927	1
ORC6	0.783035194	0.639619009	1
XPNPEP2	-0.769368877	0.596971774	1
CLEC4G	0.586564332	0.655942937	1
ANK2	-0.919669758	0.548726193	1
PCDH19	-0.400865723	0.853310875	1
FBXO47	1.501451952	0.406642781	1
WNT8B	4.217973077	0.108368026	1
NCF2	2.551983703	0.261946393	1
G34224	-0.343568301	0.820623394	1
PABPC1L2A/PABPC1L2B	2.206815629	0.432914055	1
G26925	4.451792758	0.095789908	1
FAM209A	0.735151731	0.566177719	1
LOC537017	0.573367767	0.760246383	1
FAM81A	-0.489617657	0.751378168	1
ENSBTAG00000046007	0.747653642	0.719050243	1
LOC107131287	1.525491115	0.441987632	1
NCOA7	0	1	1
ALDOB	0.15621371	0.933807307	1
FAM83A	4.297698193	0.083790821	1
GPR37	2.710700538	0.248252842	1
DOC2G	-2.596601623	0.246577256	1
ENSBTAG00000024420	-0.309330744	0.830662711	1
EBF3	-0.523495465	0.754384961	1
G18456	1.058945727	0.557251585	1
LOC536229	-0.520843899	0.698247031	1
G29488	0.526319421	0.751861871	1
TRPC4	-0.486995982	0.717529835	1
ENSMMSG00000006134	0.807306399	0.760226761	1
NCR3	1.659134919	0.345102441	1
KCNQ3	2.419385553	0.256371828	1
APBA1	-0.023357365	0.991674591	1
LOC528919	1.04907934	0.481047187	1
SCIN	-0.406249662	0.860713006	1
ZNF596	0.202315662	0.89838603	1
MED12L	3.065077577	0.251451376	1
MACROD2	0	1	1
CHRND	-0.387812982	0.845193549	1

HHIP	-0.911344015	0.589865124	1
DNAJC24	0.233783763	0.883744644	1
CD19	1.558213024	0.350885762	1
OTUD7A	1.938417021	0.481296653	1
ZNF404	2.048173643	0.230050298	1
FYB2	0.33481155	0.82197512	1
ENSBTAG00000048772	1.659106667	0.274555783	1
TMEM198	2.357086546	0.175637135	1
LOC100335828	-0.39031033	0.79768883	1
MUSK	-0.586620164	0.710696513	1
WDCP	-0.356471417	0.809213128	1
COL4A4	-1.182156772	0.356843868	1
RASL11A	0.247627457	0.827941159	1
LOC511617	0.631419568	0.697948024	1
ZNF182	1.342629246	0.441783402	1
TRPC5	-0.209372044	0.931379416	1
GSDMC	0	1	1
CSF3	-1.477700263	0.423953555	1
CSPG5	0.239053297	0.867606713	1
ACKR1	-0.15026254	0.905304237	1
ZNF688	0.780697015	0.734418026	1
USP27X	1.191246458	0.483021608	1
IL36G	0	1	1
ENSMMSG00000019885	-1.800798903	0.487021824	1
TAFA5	-0.913288959	0.574209164	1
GLS2	0.760572898	0.679529698	1
MAP2	2.634342228	0.442276135	1
ENSMMSG00000010598	1.15412798	0.528549728	1
COL4A3	-0.699075862	0.56646397	1
LOC100849046	-0.199159816	0.915838644	1
CIITA	-0.270977238	0.921119786	1
G34090	1.409950089	0.473381625	1
CHD9	-1.03130001	0.565163208	1
LOC534578	-0.798483897	0.645073631	1
ENHO	0.110522614	0.942308018	1
ZFP82	1.292541088	0.464329397	1
LOC521656	0.20293219	0.865817144	1
POLN	0.393038114	0.790864759	1
PROSER2	2.898600523	0.101086722	1
GBX1	-0.644649702	0.692478722	1
TRPM6	2.363272568	0.35471766	1
HTR1D	0	1	1
MINAR1	1.918957944	0.533072734	1
ENSMMSG00000009554	1.870584898	0.398891179	1
PRSS33	0.412258561	0.831758074	1
NCKAP5	0.557465749	0.796205387	1
FOXA3	2.55135832	0.30561347	1
ADRA2A	2.574874546	0.220571887	1
BNIP2	0	1	1
LCORL	2.190503815	0.413617255	1
DDX25	-0.803930642	0.61060822	1
PDZD9	0.372114865	0.809045475	1
SPN	1.254228732	0.533016673	1
TMEM171	-0.654418829	0.734701377	1
ZNF169	-0.463863609	0.789955243	1
USB1	1.440633953	0.542785657	1
PLEKHD1	0.060758676	0.966764191	1
PLXNA4	-0.135787471	0.949583452	1

G5761	-0.363353343	0.834017636	1
CEP112	0.44481818	0.779805782	1
ASGR1	-0.539365724	0.82037284	1
DLX4	3.888377884	0.107154687	1
TCF21	0	1	1
DRC3	2.689664612	0.145449431	1
RAP2B	0.222480879	0.875456657	1
ENSBTAG00000031271	-0.525214242	0.746661875	1
ABCB1	1.268635331	0.512763332	1
ASGR2	0.848889813	0.671554152	1
HASPIN	1.282115947	0.374156399	1
ENSBTAG00000023064	1.119150875	0.722966135	1
KRT31	0	1	1
SLC11A2	3.617356841	0.108964398	1
LY6H	-0.070433738	0.966894951	1
CNDP1	0.696214113	0.774653989	1
SAG	0.591257118	0.748744486	1
HACD4	0.383147359	0.826816597	1
LURAP1	0.425617018	0.802830425	1
DAB1	1.120855783	0.617234919	1
ING2	-0.807084816	0.658062009	1
G28069	0.94717686	0.607608027	1
ENSMMSG00000001458	0.670303939	0.710384529	1
SOX12	0.707768748	0.643862744	1
KIAA2012	1.835036049	0.362215598	1
EPHB2	-0.146947692	0.947050295	1
MOV10L1	0.137729176	0.943329248	1
DCLK1	-0.136186827	0.95334398	1
HHEX	-1.047737057	0.439119539	1
RASGRF2	-0.837547806	0.66501926	1
GAN	0.977809926	0.594546972	1
G26100	1.391776871	0.474232171	1
PACRGL	0.459440354	0.777183005	1
PRIM2	-0.273622298	0.85646752	1
RAP1GAP2	-0.287073686	0.879410351	1
ADAMTS16	0.687579064	0.787643156	1
MFAP3	-0.34922169	0.826359654	1
PTPN5	1.231715841	0.592989656	1
METTL7A	1.655403312	0.427735061	1
DNAH10	0.466663697	0.777330419	1
BEST4	0.944296148	0.550028264	1
AOPEP	1.036573466	0.644011515	1
ANGPTL6	2.693008581	0.090100663	1
ENSBTAG00000004585	3.863915204	0.112853241	1
CCDC197	-2.101937767	0.402051328	1
CCDC158	0	1	1
ENSMMSG00000003058	0.519402141	0.696619612	1
CYP39A1	0.192781982	0.928515913	1
SYNGAP1	0.903955115	0.658598919	1
KRT85	0	1	1
NOVA1	-0.933115373	0.572077608	1
BDKRB2	-0.989685171	0.513747022	1
PAPPA2	3.510428906	0.27511789	1
ENSBTAG00000002545	-0.366772641	0.806722973	1
PPM1H	1.143378923	0.667948765	1
EPHA7	2.383768584	0.236502438	1
FAM193B	0.139129394	0.901899406	1
DUSP18	0.986759439	0.576599385	1

G6517	-1.364135759	0.558708195	1
CDKL1	-0.04494012	0.972810657	1
RIMKLA	-0.47014253	0.706615291	1
F12	2.398702107	0.210780836	1
LOC504773	2.00652826	0.31783499	1
CCL16	0.06440834	0.961764437	1
SOCS1	1.323094361	0.277538742	1
G13038	-1.821538814	0.472525059	1
LMO2	5.350382171	0.131078004	1
LRP8	-0.56145955	0.69048005	1
TTC26	0.784098052	0.691966677	1
SLC9A9	2.421888848	0.295037127	1
ENSMMSG00000005865	-0.739692643	0.639177251	1
IL27	-0.596989178	0.718722517	1
RSPO1	3.449789912	0.155389056	1
LCK	1.671946181	0.285724875	1
TPCN2	0.476294588	0.741141254	1
PNMA8A	-0.538339582	0.73977034	1
RABGGTB	-1.111660304	0.548403469	1
CFAP91	0.844803873	0.558535449	1
IL17B	1.627889444	0.407809936	1
RAD9B	1.497103751	0.368196555	1
CLDN19	-1.940421565	0.595792668	1
CEP152	1.183774751	0.48992446	1
TCF7	-0.087919656	0.96453052	1
ENSMMSG00000008344	2.623215002	0.380253658	1
ZNF677	1.910610292	0.281588357	1
RAD18	1.539400101	0.382661001	1
G16784	1.06108679	0.616457176	1
PCED1B	-0.170332318	0.898040793	1
LTB	0.362735358	0.80303379	1
ICAM5	2.713938332	0.106431618	1
FARS2	-0.124759537	0.94499033	1
GPR37L1	1.878265182	0.366080579	1
STMN3	0.108614301	0.965854845	1
ENSMMSG00000012749	0.074303025	0.974546017	1
ENSMMSG00000010948	-0.741649878	0.545403613	1
HAPLN2	1.888144883	0.31954073	1
ATL1	-0.093631319	0.938201787	1
SIGLEC11	2.690302039	0.24711885	1
SLC6A3	1.314355063	0.258914165	1
NPPC	0.648282798	0.709492881	1
GPR173	-0.411961522	0.697113947	1
SLC9A9	-0.39971485	0.7896466	1
SGIP1	1.560964003	0.535552301	1
CDK1	3.114238564	0.182041362	1
MPPED2	0.590672886	0.782017322	1
HAS2	-0.540743722	0.72430213	1
YPEL4	0.621107673	0.607577376	1
NLRP3	1.293232297	0.468494828	1
MRPL37	1.384485207	0.369163456	1
PBK	0.66925074	0.747712875	1
PLP1	-0.670904357	0.61702209	1
PAX8	2.838576864	0.126324224	1
HS3ST3A1	1.69344287	0.383590331	1
EMX2	-0.219963774	0.878146158	1
TUBB1	-0.9377196	0.503220837	1
FOXP1	0.861779392	0.509033693	1

IMPG2	-0.001678723	0.9992905	1
MEST	-0.708049963	0.604644155	1
SNCA	-0.297539999	0.838173549	1
G24814	1.385743492	0.55634527	1
WDR38	1.958597822	0.186879179	1
G29238	0.302214553	0.841972059	1
HAAO	1.921387867	0.354898736	1
RRAGB	1.162293459	0.50141715	1
IL11	0	1	1
SASH3	-0.165746637	0.917210867	1
LTB4R2	2.20765941	0.225521832	1
ADAMTS8	-0.036046852	0.985428846	1
FER1L6	0.415082277	0.7711615	1
ITGAE	3.300646056	0.127454705	1
TMEM37	1.53101238	0.255769872	1
ADAMTS17	1.13356948	0.693308671	1
MAML3	0.826182028	0.615527899	1
SLC25A40	0.112723792	0.938920799	1
MYRF	0.649120395	0.739003066	1
HS6ST2	0.28904632	0.862291879	1
GPRIN3	-4.196173874	0.103495854	1
TMEM119	2.583230865	0.192183326	1
GRIA3	-1.400472728	0.600717425	1
NFAM1	0.255843701	0.895604513	1
DSCAML1	0	1	1
LOXL2	-0.500505585	0.787500932	1
ENSBTAG00000031693	-0.21985837	0.891126151	1
IPMK	-0.564268639	0.726876995	1
CDKN3	1.290168204	0.457659458	1
GLIS3	0.414423667	0.848646942	1
PRDM9	3.786736941	0.160358607	1
BMPER	-0.497940198	0.678011894	1
CCNA1	0.596451767	0.562921866	1
CKS2	2.193427955	0.24513857	1
DPF3	-0.46868553	0.747927195	1
ENSMMSG00000000978	0.171986461	0.938427581	1
PDE1A	-0.576315017	0.674857245	1
UNC13D	-0.171801095	0.907294313	1
RPS20	2.873110687	0.092986013	1
ARL11	0.839745415	0.614460686	1
ACSM5	0.130885802	0.947077382	1
ENOPH1	0.40613699	0.798794575	1
ENSBTAG00000052788	-0.028379358	0.979594112	1
G6536	-0.403118868	0.72697062	1
FOXF1	-0.039520354	0.979128695	1
G984	0	1	1
ADGRF2	0	1	1
ZAP70	0.300837565	0.879791953	1
CHRDL2	-0.689848617	0.700362098	1
CNTLN	1.218363516	0.535023562	1
LOC112442678	-1.076074274	0.369423662	1
TMEM200B	0.401218799	0.800465301	1
G21788	-0.199553074	0.896973222	1
BTBD11	0.925463091	0.406224989	1
BAZ2B	0.334606711	0.879828791	1
GIMAP6	0.26589798	0.885833502	1
ENSMMSG00000023089	0.893281596	0.612299681	1
G26662	1.403853901	0.456007861	1

ENSMMSG00000011328	1.868219767	0.178770746	1
C18H19ORF84	0.312087635	0.866747248	1
GNAS	0.536971275	0.800841608	1
PRRT1	1.130707341	0.446120058	1
BLOC1S2	0.243116975	0.879201461	1
DEPTOR	-0.038078226	0.982093372	1
G23267	1.598754008	0.52782784	1
PHLDA2	4.198022018	0.098597052	1
PRRT1B	1.928861123	0.417656837	1
FAM83D	3.137878302	0.233138393	1
ZNF521	0.152094968	0.915498392	1
CDH7	-0.527000986	0.766081074	1
STK39	-0.466640535	0.705193092	1
G8030	0.922931687	0.477156132	1
AGPAT4	-0.823547456	0.563946547	1
NPY1R	0.15245997	0.932125119	1
PLA2G4F	0	1	1
ZFYVE28	-0.835108674	0.528800875	1
SH3GL2	1.169435009	0.517851334	1
PLEKHA5	1.596045226	0.420524787	1
ENSBTAG00000054046	0.223115179	0.868890765	1
RAB36	0.989303809	0.558284853	1
TH	2.662218894	0.139339134	1
TSSK1B	1.920143835	0.38825414	1
MYOCD	0.372323506	0.835600966	1
AK7	0.860169932	0.570084749	1
DBF4B	0.946294576	0.609447922	1
UBXN8	2.199648336	0.217359844	1
CASK	-0.25432782	0.883380695	1
CFAP92	3.456685881	0.181762161	1
KNL1	0.756206211	0.747861273	1
G7991	0.139326332	0.93168968	1
SGTB	-0.554703468	0.698559735	1
DIABLO	0.010188048	0.994756926	1
RNF113A	0.066620686	0.966871484	1
ENSMMSG00000014843	0.296999033	0.87368998	1
ZNF286A	2.112495913	0.42099366	1
JMJD7	0.975698715	0.53447363	1
XPO6	0.90274536	0.649467386	1
ENSBTAG00000005240	2.260027948	0.261276565	1
ZNF569	-1.113591339	0.359352637	1
ZNF665	1.551930972	0.376850106	1
SIRPA	0.241432055	0.878326461	1
ENSMMSG00000012722	-0.876221424	0.490535588	1
CDH6	0.517102749	0.797819656	1
CNKSR2	-0.151398331	0.94695859	1
FMN1	0.81695104	0.704071765	1
PRDM8	-0.348380846	0.809282414	1
GTPBP2	2.708666614	0.286848159	1
ANKLE1	-0.674391942	0.518665266	1
SPIRE2	0.098184718	0.934934717	1
AMIGO2	-0.30862358	0.820393215	1
G30126	-0.164987238	0.91680903	1
ANKRD53	0.672087726	0.614891755	1
SRGAP3	1.890801019	0.192668717	1
TOR4A	2.434256062	0.111502792	1
PLEKHB1	-0.972716489	0.408720826	1
PTHLH	-0.349114817	0.805643341	1

GDNF	0.467920209	0.86177066	1
CCDC74B	-0.444616708	0.714200148	1
MAD2L1BP	-0.732819594	0.613225751	1
ENSMMSG00000021461	1.211732544	0.563370293	1
CDC45	0.117987718	0.935595155	1
GPR158	0	1	1
SH2B3	2.52318266	0.418991995	1
ENSMMSG00000020641	-0.788847385	0.40797646	1
MGC157082	-0.037394714	0.974066854	1
G23414	-0.599086795	0.844125162	1
FGF3	0	1	1
CASS4	2.025185685	0.274298191	1
ABI2	-0.284265921	0.844451621	1
PRKCG	0.751421538	0.704107651	1
KLF14	-1.018541673	0.376706955	1
RUNX2	1.097605078	0.503519088	1
STING1	-0.144716441	0.93815807	1
PPEF1	1.286469656	0.527493803	1
RBM41	1.441979466	0.323236083	1
LRRC61	0.542532318	0.604789514	1
FOSL1	-0.768073949	0.734071317	1
LOC101905041	1.697240119	0.27937318	1
ENSBTAG00000045868	-0.135909603	0.930404833	1
ITGBL1	-0.32271227	0.840609355	1
ZNF319	0.200805643	0.905637247	1
TTC36	2.797963154	0.105032585	1
CIB3	0	1	1
BCAR3	0.884973727	0.704811147	1
SLC10A7	0.591211995	0.757494731	1
NRGN	-0.549651701	0.705059802	1
FAM89A	-0.929577437	0.502572411	1
PGA5	3.898787052	0.153402625	1
INHA	2.752942637	0.241111409	1
PDZD2	0.600194217	0.762442003	1
CEP76	-0.746348721	0.605601594	1
DNAH1	1.802476341	0.44027402	1
SHISAL2A	-3.302046922	0.236782855	1
MKS1	1.471615969	0.39302999	1
MAMLD1	0.89897572	0.709623798	1
ENSBTAG00000032873	0	1	1
KIAA0895	0.19667727	0.896318382	1
METTL15	-0.74103605	0.670760646	1
DMXL2	1.296932154	0.563552808	1
TRIM34	0.852488278	0.621441211	1
CD22	-0.665576954	0.730656846	1
G1594	-0.669610243	0.717398448	1
NTNG2	-0.560542082	0.716122439	1
LOC508933	1.526833908	0.245533722	1
SPI1	-0.971338227	0.506074629	1
KCNAB2	-0.41245033	0.772455525	1
SEMA3D	-0.043947314	0.970698422	1
NR5A2	-0.725004335	0.683470866	1
MED12L	1.962934654	0.40789068	1
SLC45A3	0.730920509	0.708555117	1
ENSMMSG00000015671	1.452397683	0.455772244	1
G18527	0.54014406	0.738468345	1
ADAT3	-0.106927524	0.935773424	1
ZNF280D	0.155574482	0.921204026	1

STX1A	-0.758585485	0.549485344	1
CCDC3	0.608614034	0.723559557	1
NRK	0.323920476	0.863619114	1
NTNG1	5.105194374	0.091557878	1
SRRM3	-1.813896979	0.567353825	1
G29695	1.281551041	0.506396146	1
RASSF2	0.022022041	0.989683793	1
SEZ6	-0.207257416	0.898640021	1
SYCE2	0.144122589	0.911060335	1
BDKRB2	-0.527329978	0.694275033	1
LOC100299180	0	1	1
NIPA1	1.54624611	0.377263583	1
PLEC	0.440744455	0.789260489	1
LINS1	2.628687465	0.099582862	1
TNFAIP8L3	0.551892035	0.619553392	1
MZB1	-0.437495955	0.73126706	1
ITGAD	-0.824526323	0.559679791	1
WSCD2	-0.978537725	0.379851785	1
EXOC6B	0.26219566	0.827864561	1
G26318	-0.518292338	0.740676496	1
CEBPE	3.319307811	0.160235449	1
LRRC17	0.115666174	0.934333879	1
MAML3	-0.265735467	0.824796542	1
TECTA	0.450376992	0.83368663	1
ENSBTAG00000053375	-0.000452955	0.999832192	1
HMGB3	0.89373958	0.58418441	1
G13658	1.28570747	0.608990608	1
SLIT2	0.483786635	0.827676755	1
COL11A2	-0.493524473	0.742495112	1
MRPL4	0.076789239	0.96237315	1
GALNT16	-0.736184317	0.588820787	1
RAD51B	0.118091526	0.930326521	1
CMKLR2	-0.647749916	0.57603405	1
TOPBP1	-0.554123458	0.715260848	1
P2RY6	1.453996	0.36641117	1
C19H17ORF100	-0.565181519	0.704803964	1
TMEM176B	0.02610893	0.982586138	1
SEMA4D	-0.950700107	0.504512387	1
NXT2	0.555290083	0.7102636	1
C25H16ORF89	0	1	1
UBE2QL1	0.44302377	0.807655985	1
IGF1R	0.559623656	0.72446605	1
GRAMD2A	-0.587380494	0.664272228	1
CHD9	0.517145585	0.719778566	1
LOC404103	-0.752870478	0.680420666	1
ZNF23	-0.480980761	0.701315576	1
RPS17	1.301808964	0.372740035	1
CENPA	1.139453887	0.708454271	1
C1ORF112	-0.63155578	0.655408615	1
ENSMMSG00000008683	0.505476902	0.68212751	1
LOC512867	1.746795106	0.200575969	1
SYBU	-0.69448527	0.68077332	1
ADORA2A	-0.619306079	0.631783941	1
CFAP100	1.652390835	0.431110182	1
SAXO2	3.348236694	0.210294944	1
C1QTNF6	-0.204448242	0.902451556	1
SRFBP1	0.404985051	0.78697017	1
IIST1H3B/H3C1/HIST1H2AIL	0.823759093	0.649877224	1

CHST7	-0.926732435	0.499412075	1
RUNX3	1.400330695	0.546514563	1
DDX4	2.312614261	0.28582523	1
MSH5	1.240090047	0.29170041	1
GASK1A	0.899911839	0.607230886	1
CEP112	1.071701378	0.550900246	1
A3GALT2	-0.444055512	0.746731477	1
FAM221B	0.424746924	0.745838446	1
MDM1	0	1	1
GAS2	-1.079527068	0.459226237	1
PCDHB4	-0.288120013	0.842665765	1
RAB20	0.62547297	0.675708404	1
SLC20A1	2.614338724	0.14219374	1
CDKL1	-0.855790901	0.626944623	1
G34319	-0.280511201	0.836682116	1
IRF8	-0.272071457	0.862940967	1
CCL24	-1.02110208	0.41760197	1
EN1	0.301136934	0.858889943	1
PDE10A	-0.498647863	0.749661819	1
G23764	-0.658354917	0.795788565	1
SHISA5	0.175520657	0.917486704	1
TESC	-0.007563611	0.995449083	1
ZNF385D	-0.203230018	0.88723406	1
ESCO2	0.084427478	0.963720888	1
G36461	-0.798982814	0.513152878	1
SPATA21	0.275315791	0.864802025	1
GRID1	1.510507896	0.38255485	1
VWA2	-0.186909277	0.935005203	1
EXD1	-0.026743879	0.985128245	1
SPEF2	0.832107348	0.491193117	1
GPR17	-0.412578356	0.824913535	1
TEX261	-2.851487806	0.201728299	1
TMEM26	-0.3389219	0.810885922	1
INTU	1.243166912	0.530892508	1
LOC788142	1.271932389	0.536793525	1
SLC9A4	4.436431538	0.116987961	1
G27815	0.69341247	0.687646792	1
GALNS	-0.100155465	0.924616194	1
ARHGAP11A	2.095220237	0.211624818	1
ROBO1	0	1	1
ATP5PD	-0.335565678	0.805400291	1
IRAK2	1.502280961	0.415147055	1
ATP8B3	2.471703245	0.282830535	1
TOMM40	-1.03380947	0.443536497	1
ENSMMSG00000021053	1.494441614	0.321850595	1
KSR1	0.258639088	0.92434381	1
SLC16A2	-0.194702242	0.896771921	1
ENSMMSG00000014391	3.58302852	0.121844672	1
ZNF541	0.388526935	0.740826013	1
DLC1	0.023773922	0.990151806	1
VEGFD	2.944635973	0.191652503	1
NFE2	0.707257975	0.637490706	1
KITLG	2.322780557	0.28894406	1
ENSBTAG00000010836	1.517390844	0.397893676	1
LIPT2	-0.463803923	0.758259054	1
DGKB	-0.954568179	0.742100325	1
CTNNA3	-0.079842112	0.951005598	1
BATF	0.749856711	0.701640972	1

GON4L	0.327766417	0.814663235	1
PROS1	0.024135958	0.982799706	1
INAFM2	-0.460041307	0.742157616	1
CACNA2D2	1.567276397	0.507593018	1
ZNF419	-0.606860613	0.657469941	1
ARHGAP9	0.226221134	0.869708554	1
KCNN2	2.413488372	0.292331097	1
ZNF621	0.516424391	0.752997961	1
PLA2G4E	-0.267234858	0.905094635	1
TP53TG5	0.301560315	0.857362231	1
KCNG2	-1.037516819	0.411434192	1
CCT6B	0.768203262	0.658794153	1
B4GALNT3	0	1	1
ANKRD65	1.415690461	0.50044987	1
PLEKHG4	0.647406018	0.663161281	1
ANKRD39	2.198682152	0.403711692	1
ABCB1	0.451192302	0.790425601	1
LOC509184	-0.67103215	0.634465798	1
RASL10B	-0.629690026	0.60515729	1
SHISAL1	-0.669905066	0.723373561	1
TWIST1	1.161835699	0.428856495	1
PTGER3	2.139408578	0.333112638	1
INSYN2A	1.394948614	0.548800307	1
RSPH1	3.017417021	0.226753626	1
LINS1	1.549043463	0.320373644	1
ADRA1D	-0.919180675	0.553826693	1
ATG9B	2.032016768	0.329537098	1
GNA12	0.596830924	0.683343213	1
CCDC146	-0.206606054	0.91231934	1
CCDC40	0.939621768	0.539911201	1
NOX4	0.189544159	0.901291091	1
ZFP90	-0.200531796	0.891560642	1
SLC25A19	-0.993618521	0.472794576	1
ENSMMSG00000019394	-0.430836458	0.821431643	1
AKAP6	-0.152619228	0.934913676	1
DIAPH2	1.317409395	0.565055235	1
ENSMMSG00000004083	-0.115100723	0.946952286	1
ENSMMSG00000013947	-0.379748018	0.796298473	1
ZNF879	0.371885131	0.805965106	1
C16H1ORF53	2.373914004	0.136123976	1
RPL3	0.057148908	0.958593232	1
VSTM2B	2.581910353	0.194023443	1
RAB3D	1.797395146	0.339307223	1
MAMDC2	-0.988626924	0.496863667	1
SOX7	-0.457956723	0.696504749	1
STIL	1.08137492	0.675774064	1
NHS	0.341654993	0.769400127	1
ABCG8	0	1	1
DMP1	5.024190379	0.127240882	1
SLC17A8	0.415533992	0.710946897	1
ENSMMSG00000017120	-0.289550381	0.828171365	1
LOC100848331	1.159521058	0.461020196	1
KRT2	2.923606266	0.344281726	1
KCNQ5	-0.31192151	0.845752893	1
G20057	-1.82363424	0.452441726	1
ENSBTAG00000003593	-0.500317385	0.736587926	1
ENSMMSG00000000605	2.949904206	0.224434931	1
RAB30	-0.526385309	0.781654515	1

ZFP69	-0.829707084	0.508692713	1
ZNF566	-0.589125508	0.655359108	1
OTOF	3.243702167	0.140648761	1
NR2F1	0.925990695	0.537525532	1
DENND5A	0.328475774	0.834246615	1
OIT3	0.183972112	0.879369089	1
LY6G5B	0.938801952	0.505698423	1
PYCARD	2.643862955	0.094142141	1
ZKSCAN7	-0.218193824	0.847240355	1
HGF	-0.236747056	0.872530532	1
ASTN1	-0.365216414	0.881047522	1
ENSMMSG00000013342	0	1	1
ENSBTAG00000053707	2.606553466	0.166394395	1
KIAA1614	0.384914305	0.76040203	1
LOC789996	1.571555193	0.480393192	1
HOXB4	-0.527138013	0.720295364	1
SLC11A1	-0.268026271	0.84974716	1
ADGRL3	1.55165227	0.349307153	1
ARID3B	0	1	1
ZNF835	-0.769560871	0.664981501	1
TMEM62	0.267104021	0.855187509	1
CENPM	-0.840728455	0.778765312	1
SPAG17	1.217750375	0.522265845	1
SCAMP5	-0.641673651	0.651607262	1
ENSMMSG00000000849	-0.200987953	0.896219866	1
LOC613282	2.628803589	0.335842036	1
APOB	0	1	1
MRAP2	1.716020812	0.424846508	1
FGB	1.295707816	0.518832818	1
ABCC8	2.055911857	0.197444374	1
GLIS3	1.250144823	0.588999578	1
IRAK1BP1	0.188940477	0.853620347	1
CXCR6	0.424219261	0.857278412	1
GP1BB	3.828551142	0.133060465	1
MMP27	3.162437923	0.182544382	1
IL1F10	0	1	1
TMEM241	0.741253076	0.677295811	1
CCDC68	-0.431855431	0.757297304	1
ENSMMSG00000012525	1.370877358	0.208418397	1
RASAL1	-0.0544306	0.97898167	1
LOC115945173	0.819741778	0.68148535	1
CCDC3	0.660777357	0.600615031	1
LIPG	-0.894496876	0.455827133	1
RPL10A	1.097814227	0.346756154	1
SORD	0.347087313	0.704982889	1
ELMOD1	2.272763227	0.403469541	1
BRCA2	2.309480641	0.305784814	1
CRTAC1	-0.636559883	0.567677563	1
ALX3	3.643662084	0.149648721	1
NCF4	0.370993366	0.780466192	1
MT1A	0.29228092	0.846554928	1
CCDC78	0.837009628	0.669352594	1
ZC4H2	-0.401956601	0.748077207	1
SLC36A4	1.063686802	0.572422583	1
CDCA2	2.422387171	0.209396849	1
PSRC1	1.062140532	0.495455991	1
RNF122	-0.875016896	0.46803475	1
TNFSF12	-0.936569279	0.456722977	1

G679	-1.074352978	0.557699765	1
ENSMMSG00000018785	1.004163868	0.521464821	1
C5AR2	-0.341351307	0.829651592	1
CABLES1	0	1	1
ELL3	0.052155444	0.963627495	1
LOC100852077	-1.017168602	0.456582044	1
ENSMMSG00000013786	1.273991216	0.445906347	1
CD52	0.022221606	0.985834395	1
INHBA	1.085329425	0.426672942	1
G33024	-0.252110787	0.874750099	1
NKG7	0.895445535	0.553538417	1
MAP2K5	-0.753083099	0.597099348	1
SMOX	0.740482921	0.600997002	1
QPRT	0.330430841	0.819028716	1
SEZ6L2	1.890975544	0.416433826	1
LRRC4	-0.32603908	0.863579548	1
ARSJ	0.19343012	0.853860645	1
TTC9C	-0.003560045	0.998265481	1
HHLA2	3.37770619	0.199293503	1
FOXP2	-0.191568817	0.928454334	1
CDH19	0.78557849	0.620877701	1
TSPAN6	-0.381676817	0.751895264	1
SYN3	-0.699068317	0.706140171	1
GAS2	-0.917716602	0.591598903	1
GPR137C	2.68024449	0.187312514	1
KLF12	0.074316988	0.967593715	1
G19720	2.601517429	0.280276663	1
DMTN	-0.630524366	0.632674231	1
APH1B	2.050051692	0.227153265	1
NSG2	0.423449865	0.81296559	1
CDHR4	0.559004998	0.685034765	1
CLEC4A	0.607200305	0.689465288	1
ENSBTAG00000017316	1.067181903	0.57021999	1
C5	1.018431189	0.726838426	1
HPGDS	0.818044921	0.548403143	1
TRPC1	-0.283866708	0.847164762	1
CWC25	0.651944305	0.593046103	1
G31237	0.861457574	0.607088222	1
FKBP1B	0	1	1
RERGL	-0.571678312	0.647605111	1
PPBP	0.779562104	0.666071299	1
NOVA2	-0.313395557	0.816201673	1
UNC13A	2.010639973	0.182859459	1
G29879	0	1	1
WHRN	2.336697378	0.286357798	1
ALDH1A3	-0.127314067	0.933965703	1
SIK2	-0.671708836	0.614354237	1
ATP8B1	2.623222088	0.183162791	1
LIMD2	-0.074530724	0.950264382	1
LOC100139670	0.689106135	0.684260352	1
ENSBTAG00000011671	1.185473271	0.603748588	1
PLAC8A	1.722158389	0.479186479	1
RALGAPA1	-0.436919984	0.744771619	1
GAS8	0.514349594	0.624465555	1
COL11A1	-0.142790301	0.938696975	1
S1PR3	-0.812413018	0.641432379	1
G13756	0.647590607	0.706537755	1
OTUB2	1.951571588	0.132301375	1

FOXD3	-1.002174322	0.400190747	1
LPXN	0.861874508	0.528583368	1
ITGB3BP	0.485776426	0.742480459	1
PDZRN4	0.326795402	0.826784293	1
ZNF35	0.505328648	0.755653229	1
CENPW	2.34892187	0.39600818	1
CADM3	-0.893065253	0.437324592	1
NEK11	-0.776373878	0.699758753	1
SPTSSA	0.73435853	0.702347675	1
INSC	-1.078279861	0.548710412	1
GOLGA7B	-0.821099704	0.552116386	1
SMAP2	-0.304714052	0.82991583	1
ENSMMSG00000013713	-0.534051489	0.667663918	1
ATP11C	-0.001863527	0.999035186	1
DUSP4	-0.777122586	0.68467911	1
STAR	2.244062588	0.186287731	1
DOK2	-0.611825667	0.668257902	1
ZBTB32	0.00314225	0.998026047	1
USH1C	-0.173891262	0.910368928	1
IL9R	-0.63929823	0.648716223	1
CFAP100	0.797480786	0.504713113	1
KRT81	0	1	1
C1H21ORF91	1.573007461	0.346800368	1
VPS37D	2.11228204	0.226517806	1
SPC25	0.302459837	0.809845275	1
LOC104968820	-0.153087265	0.913710703	1
BCHE	-0.548491816	0.702953899	1
SLC35B4	1.002313168	0.451168221	1
ENSMMSG00000019981	-0.342559021	0.813613146	1
SLC13A4	1.562054777	0.320036781	1
HNRNPLL	-0.841970394	0.646097309	1
PIP4K2B	0.011924017	0.993381634	1
EEF1A1	2.228984415	0.091263171	1
GGACT	-0.165851524	0.908876082	1
ENSMMSG00000003157	0.459730593	0.681007424	1
GNAI1	0.499859916	0.688995965	1
SLC32A1	3.204498101	0.170325041	1
CLEC16A	-0.072793632	0.968458417	1
UBE2E1	-0.653306149	0.80703954	1
HGD	2.434040436	0.16380726	1
CD101	1.265185862	0.560915262	1
ENSMMSG00000015959	-0.205937756	0.889200418	1
WDR31	0.895473443	0.642417445	1
CDYL2	-0.189499221	0.891880807	1
LYSMD4	1.23705567	0.451870968	1
ENPEP	-0.412687932	0.757575545	1
HAS3	0.384338595	0.805566611	1
YPEL1	-0.429712832	0.71182458	1
CDCA5	-1.813128682	0.579614762	1
ENSBTAG00000053322	-0.156142642	0.921997749	1
ARMH1	3.433054055	0.083702527	1
PCLAF	1.65910487	0.452447861	1
ANKRD50	-0.027864201	0.983688416	1
RIIAD1	0.20831441	0.890113937	1
LOC100336984	0.91538977	0.690072105	1
G3023	-0.511813989	0.760579052	1
C1RL	0.491326255	0.665004251	1
B4GALNT1	0.673708032	0.636480228	1

HHAT	3.644902625	0.147527276	1
SCNN1D	2.632975169	0.363204478	1
PSMD4	-5.546381591	0.184378495	1
ZNF132	0.553781812	0.688611295	1
CAPS2	-0.369607158	0.794556833	1
NPR3	-0.305717211	0.8637955	1
LOC512863	0.394307898	0.830559243	1
IRF8	-0.791344298	0.610541544	1
RANBP6	0.466644835	0.669650728	1
MYL10	0.495984372	0.790232288	1
SPTLC3	3.284326311	0.145132015	1
HEATR1	-0.260681357	0.857605264	1
ST18	0	1	1
NANOS1	-0.606863299	0.619817289	1
CENPE	5.259814157	0.133211875	1
UNC-30	0.701764843	0.708170451	1
ARHGEF33	-0.12379333	0.951494248	1
TNFAIP8L2	-0.742123459	0.626846664	1
G31406	-0.421129443	0.754701154	1
PFKP	-0.364548133	0.803893506	1
EIF4A1	0.310043009	0.833802903	1
CCL1	-0.455518627	0.733717178	1
GRO1	0.442023821	0.837817182	1
ENSBTAG00000038755	0.412516647	0.794287167	1
PRICKLE2	-0.061198372	0.973532314	1
APLNR	-0.905359592	0.457941657	1
ZNF385C	1.158437917	0.473904412	1
CXCL10	-0.299734075	0.84149552	1
PRKN	0.018533474	0.991343157	1
C25H7ORF61	0.301537646	0.806801763	1
PPIA	0.862466397	0.571417213	1
MECOM	-0.018600492	0.992395262	1
KIT	-0.595544488	0.691452439	1
RAB17	0.25759498	0.875429001	1
CNTLN	0.859702958	0.654784816	1
CYTH4	-0.058401739	0.967961861	1
LHFPL2	-0.389842828	0.829724723	1
ZDHHC4	2.171888359	0.28859664	1
GZMB	-2.853512563	0.283719423	1
TPPP2	1.014292684	0.501174033	1
MLLT3	-0.344679436	0.815662041	1
TMEM178A	0.11181322	0.938663587	1
LGALS4	1.675203991	0.192248319	1
ELP4	-0.362126228	0.803943841	1
RNF144A	-0.571808895	0.587400893	1
WDFY4	-0.175739823	0.873241374	1
FAM110B	-0.320116538	0.817601986	1
ZSCAN23	0.15030294	0.923253054	1
KRT33B	0	1	1
ZNF346	0.530968055	0.740853408	1
ENSMMSG00000011870	-1.100494745	0.353595654	1
ZNF697	-0.320010948	0.846111865	1
ADCY8	1.642476715	0.369519008	1
CNTN4	3.560569293	0.152470717	1
IQCA1L	-0.641345159	0.705498589	1
BOLA-DMB	1.156799302	0.447494435	1
LRCH2	0.923481781	0.669079991	1
SAA1	3.087526703	0.108356086	1

LRRC46	1.777496453	0.230594028	1
COPZ1	1.63788934	0.409664903	1
PHYHD1	1.22640276	0.41912575	1
TMEM35B	1.185037555	0.402660272	1
NUDT15	-0.418751179	0.773480475	1
G23865	0.570063769	0.605676599	1
WNT2	-0.289711674	0.870706418	1
SAMD14	0.331629357	0.779082856	1
IGSF10	1.974298174	0.291564795	1
SCN11A	-0.343994702	0.801267286	1
DCLK2	-0.266829253	0.857744584	1
VXN	0.620112471	0.708950315	1
DNM1	0.308906927	0.8809818	1
PRR3	0.547226314	0.712012899	1
CCDC86	1.058442845	0.596511611	1
SERPINB6	-0.934066799	0.449236789	1
HVCN1	0.290624693	0.805805986	1
IL34	-0.123150878	0.899246532	1
HS3ST1	0.933774751	0.462099285	1
MR1	1.157431589	0.451990096	1
ENSMMSG00000009678	0.698923384	0.596591359	1
G31761	0.236730816	0.813162275	1
PRDM15	0	1	1
LEF1	0.005475147	0.996820364	1
GRM4	-0.006496508	0.995979214	1
NOS2	4.144561258	0.141600752	1
LANCL1	-0.103352906	0.928133782	1
ZNF248	0.861039761	0.580174864	1
CRPPA	-0.41094871	0.758437898	1
EBF2	-1.035153498	0.329768445	1
BMERB1	1.114895116	0.58424311	1
GALNT14	3.000875716	0.108426638	1
NKD1	3.00457035	0.112340833	1
KIF2C	3.195171111	0.087961539	1
LOC782922	0.956919906	0.547438497	1
FUT4	1.022277034	0.509583313	1
PABIR2	1.676367324	0.413488167	1
JCHAIN	0.470353154	0.761170823	1
H2BC18	0.328984073	0.837292604	1
FOLH1B	1.278637775	0.5858045	1
ARX	-0.309362796	0.834620173	1
ALX4	3.027421394	0.212884351	1
LOC100297056	0.190477474	0.895988478	1
SLC4A9	1.899960297	0.107877845	1
CEP350	-0.758919712	0.738130367	1
ENSBTAG00000049560	1.472255938	0.511120013	1
IL3RA	2.469654631	0.143114341	1
SLCO4A1	0.363598956	0.854294393	1
UST	-0.368417333	0.78179885	1
DOC2B	1.058508809	0.482677852	1
SIGIRR	-0.258293293	0.895217004	1
NEK3	1.212868047	0.414092956	1
FGF6	-1.093787189	0.379640402	1
TNFAIP8	0.844147107	0.494321726	1
TMEM91	2.998390087	0.100872307	1
PIK3R5	-0.37221044	0.77845833	1
TMEM107	1.420453151	0.374544126	1
PPP4R4	-0.342269207	0.79271959	1

GSTP1	1.086317804	0.411921754	1
H1-5	4.311924277	0.158239036	1
GPR135	1.700732083	0.299064557	1
PTGER1	-0.170935054	0.882293143	1
CITED1	1.028758665	0.446994077	1
BTK	-0.185705615	0.905002558	1
STC1	1.54012295	0.394314349	1
C18H19ORF33	3.593876988	0.112689078	1
CEP112	1.881091023	0.298052271	1
INPP5F	-0.197805793	0.896402811	1
RASL12	0.887081977	0.592130963	1
KBTBD8	-0.403858597	0.783013717	1
ENSBTAG00000054538	-0.012972171	0.992270457	1
CAMKK1	-0.080088739	0.940944218	1
ENSBTAG00000053294	0.051025463	0.972448493	1
SLC35F3	0	1	1
EMILIN3	-0.488838765	0.767821692	1
U2AF2	1.295364353	0.432476954	1
ASTN2	2.935242788	0.270684742	1
CENPC	2.165779122	0.261962775	1
TMEM145	1.656458729	0.329068263	1
RNASE1	-0.735030611	0.602087968	1
TMEM98	-0.313421983	0.785340481	1
NRXN2	-0.123645273	0.934094366	1
LDLRAD4	-0.055883366	0.97464858	1
CPA3	0.880940005	0.493325191	1
PCDH18	1.05453529	0.481729149	1
FIBIN	0.222609021	0.886470143	1
THSD7A	0.093856089	0.957254305	1
ZNF304	1.358615241	0.347375091	1
LRRC38	0.078108179	0.960513081	1
PLEK2	0.636400759	0.636507383	1
DPYSL3	0.110324369	0.941239321	1
TMCC2	-0.1282446	0.932991761	1
BMP2	-0.825638315	0.465800603	1
ZNF407	-0.611308953	0.651966671	1
SV2A	3.947096421	0.120362708	1
SLC46A2	1.705720713	0.380491216	1
RELN	2.536391641	0.429174793	1
ZNF605	0.954773212	0.468300637	1
C1ORF53	0	1	1
RAB42	0.607922702	0.719988507	1
ENSMMSG00000017228	1.127066383	0.534257698	1
HPCAL4	-0.815690226	0.56546822	1
CYP2U1	0.022163905	0.989893655	1
TNRC6A	-0.675961863	0.621093984	1
CASP7	-0.697780168	0.59452217	1
LRRK1	1.466963781	0.434095623	1
PIK3CG	-0.659081893	0.632168859	1
NRM	-0.527258899	0.714988181	1
WC1/WC1-8	3.514776783	0.259711457	1
ACKR2	0.266833663	0.817908079	1
G11827	0.632523571	0.708689921	1
ENSMMSG00000017657	0.03778399	0.986700858	1
WDR77	0.26733566	0.857117603	1
KPNA5	0.329718431	0.830646737	1
PLIN5	-0.062398284	0.96690406	1
DNAI4	3.320516579	0.260470055	1

EIF3K	0.28048362	0.92851661	1
ENSBTAG00000047175	0.611032398	0.526787643	1
SLC12A3	0.740870671	0.606332232	1
PDE2A	-0.780742159	0.593941463	1
EBF3	-0.12161959	0.933792258	1
ENSMMSG00000019969	1.777626129	0.526895031	1
BUB1B	-0.261865194	0.902372359	1
FMN1	1.162162173	0.53177689	1
MSRA	0.783579641	0.653421913	1
ZNF350	0.656541565	0.655955042	1
MAP3K15	-0.802210075	0.506481766	1
SLC30A2	0.462816189	0.769955955	1
HOPX	1.337338274	0.410366359	1
DZIP1L	-0.01971952	0.989211321	1
TRPM3	-0.410078685	0.712644036	1
GAL3ST3	-0.590400401	0.617655995	1
KCNA5	-0.241144573	0.886343233	1
LOC616948	0.128067029	0.94791408	1
BCL2	3.372563707	0.169015981	1
SLIT3	-0.493288346	0.772406575	1
ENSMMSG00000021858	-0.045643321	0.965263598	1
NECAB3	2.022750021	0.126374026	1
ZSCAN30	0.279092836	0.84968922	1
SLC2A6	1.763877825	0.244927688	1
DICER1	0.220419718	0.920156108	1
CHAC1	-1.255412833	0.433090472	1
SEMA6D	-0.56218688	0.74517374	1
ENSMMSG00000017518	1.885811117	0.197889304	1
CFL1	1.454502613	0.30040712	1
RAB3IL1	1.722839145	0.426672017	1
BSN	0.00555499	0.997241652	1
ESRRB	0.294191473	0.856532704	1
SNPH	1.787126217	0.302575756	1
MAP10	-0.277996053	0.849381533	1
C8G	2.252790544	0.214910504	1
PTPRD	-0.135183641	0.931956011	1
NPFF	3.28799534	0.094369138	1
CREB5	-0.603912475	0.677429964	1
BLM	1.205894714	0.397095961	1
SLC12A8	1.84749553	0.251796487	1
ENSMMSG00000013388	-0.674748637	0.626833544	1
PRDM16	0.175112189	0.91127863	1
TBX18	-0.633882101	0.725978734	1
ENSMMSG00000023151	1.231637264	0.369695851	1
RAB3B	1.750815931	0.240895118	1
ATRNL1	0.301293259	0.872347678	1
DCLRE1B	0.474014299	0.774098398	1
HAS1	-0.630018918	0.581804329	1
G35633	-0.240326191	0.864798267	1
KCNIP3	1.361184013	0.454856425	1
LYRM7	-0.107304923	0.913519225	1
OXCT2	0	1	1
TMEM170B	-0.603283277	0.700142951	1
RUNDC3B	1.99354411	0.272059482	1
TMEM191C	2.618647094	0.290139481	1
CAMTA1	1.466397568	0.578122968	1
PHLDB1	-0.746971351	0.675991704	1
ZCCHC2	0.241259374	0.882075631	1

ALPK2	-0.610538023	0.699530245	1
SIRT3	0.323061436	0.839681623	1
UST	-0.20117457	0.893453071	1
ENSBTAG00000025023	1.639848019	0.233513326	1
IL15RA	0.068530568	0.961027256	1
TADA2A	-0.100247695	0.946004604	1
TMEFF2	-0.351390201	0.719925255	1
PRTFDC1	0.838426928	0.585640701	1
RGS16	-0.647423046	0.635991131	1
PGR	-1.221064417	0.358325125	1
PRRX2	-0.019836027	0.987764106	1
G24473	0.046696904	0.978887648	1
VOPP1	-0.363689213	0.79292959	1
OPHN1	1.582152933	0.383928298	1
HDAC9	0.425414542	0.872260578	1
ARL5B	0.964477973	0.617374571	1
ENSBTAG00000032047	0.026706706	0.985822551	1
ROR1	-0.465505829	0.740578079	1
HES2	-0.15737868	0.915064377	1
PODNL1	0.668414348	0.691991959	1
IL15	-1.130845064	0.311770954	1
LOC510536	0.317012837	0.825364862	1
ENSMMSG00000009673	-0.702496309	0.652160904	1
ZNF382	0.486027529	0.764186495	1
NAA16	0.750021683	0.73293607	1
NUBPL	0.094233974	0.924535252	1
CA5B	0.343449553	0.814726406	1
ARHGEF9	0.111909334	0.940772809	1
RBP2	0	1	1
GLCCI1	2.422632064	0.282267897	1
EML6	1.765012256	0.385214591	1
NXN	-0.699639397	0.640261275	1
CFAP100	1.435658836	0.412258379	1
BBS9	-0.996999861	0.454749386	1
DCLK2	4.710712813	0.114550291	1
ENSMMSG00000010165	1.155637084	0.562993467	1
PRTFDC1	-1.022302747	0.561571369	1
PUM1	-0.508702469	0.710223223	1
SMAD9	-0.371075249	0.786988496	1
DNMT3B	0.767919218	0.491772399	1
VWC2	0.383250381	0.817154089	1
TENM3	-0.124234993	0.922687772	1
OCA2	-0.657862515	0.700229687	1
PPP1R3D	-0.546267135	0.718391052	1
LEPR	-0.211249988	0.887325552	1
POLQ	0.193066585	0.904642901	1
MSANTD1	1.570151872	0.251724557	1
HESX1	0.767498939	0.599298063	1
STK32C	2.320353304	0.186220135	1
IDNK	0.474659456	0.696234865	1
SYT16	4.691283732	0.101599436	1
ATE1	-0.256188232	0.858562436	1
CGNL1	0.645950053	0.683822109	1
PRKAR1B	0.580116281	0.736197674	1
ENSMMSG00000014697	0.194234696	0.922132896	1
C2H2ORF69	-0.107415739	0.939462492	1
CST7	1.004513834	0.553559099	1
HSD17B7	-0.287793068	0.851796207	1

SGSM2	-0.773436032	0.68108633	1
KRBA1	-0.68270836	0.802180787	1
MMRN1	0.115593498	0.937442101	1
NDUFA4L2	0.312112785	0.806629601	1
G18888	-0.600571202	0.662477545	1
TUBG2	0.329913878	0.861027904	1
EBF3	-0.964795638	0.483288958	1
RAB26	1.994833841	0.292876302	1
PDE3A	2.916516655	0.204912994	1
RASSF5	-0.747747673	0.57919948	1
ENSMMSG00000003790	-0.560203803	0.658951586	1
RELN	0.365233965	0.842435576	1
CYTIP	-0.352650871	0.801524109	1
PGPEP1L	0.830014238	0.675154959	1
LAT	1.955213724	0.148526661	1
ZBTB3	-0.130440442	0.929676819	1
ENSMMSG00000001446	-0.204165384	0.880906421	1
DLG5	0.591463981	0.618529698	1
CCDC47	-0.256520707	0.784762143	1
ZNF565	0.179434771	0.904321128	1
MAPK14	-0.198981101	0.86465014	1
LMX1B	1.265478753	0.431431949	1
GP1BA	0.750574219	0.662983138	1
CKAP2L	1.561718563	0.330558457	1
SH3RF1	-0.421193324	0.79389308	1
PCDHGA12	0.788957962	0.572218881	1
G10070	2.678012897	0.146813669	1
IL12RB2	-0.738770355	0.664609935	1
ANXA13	0.134495447	0.904103968	1
FUNDC2	-0.731784937	0.441728809	1
RCOR2	0.338225439	0.771910382	1
BARD1	0.7844473	0.587502324	1
G25811	0.676382778	0.600020806	1
CAMKV	-0.586668489	0.729826526	1
SLC38A1	0.147749339	0.927675029	1
CIDEB	2.879725753	0.118980619	1
MMP16	0.876840744	0.4523563	1
ENSMMSG00000020441	0.920385052	0.624000721	1
NTN1	-0.319713021	0.838024449	1
SUGCT	1.718367143	0.30726867	1
METRN	1.502492579	0.349321259	1
ZNF614	-0.478386268	0.719763502	1
ALDH1A3	-0.408869119	0.782706456	1
ULK4	-0.584351804	0.656813398	1
RRP12	0.240056787	0.876144468	1
G15081	-0.600474346	0.674414932	1
GTDC1	0.798087908	0.605326753	1
MYL4	2.398989799	0.339744108	1
HRH1	-0.641296119	0.628422382	1
AVPR1A	-0.951264036	0.477652533	1
G37611	-0.075839624	0.953379761	1
TDRP	-0.071927127	0.958862786	1
ENSBTAG00000051419	-0.26125665	0.843398069	1
MAN1B1	0.384692302	0.781575267	1
PDE9A	1.098938254	0.530588068	1
ZNF570	-0.178499976	0.896993164	1
ETFBKMT	0.871024905	0.59399372	1
PRKD2	-0.080917914	0.946614314	1

KIF19	1.526946875	0.405906857	1
SKAP1	1.239387119	0.336147913	1
CALML4	0.313615934	0.786161071	1
BANF1	-0.383182638	0.676701973	1
LRRC25	1.379534848	0.456257749	1
LYST	1.068835686	0.641844606	1
EPOP	-0.739292625	0.68788367	1
CCDC102B	2.967430882	0.242681482	1
GDI1	1.023362353	0.467741761	1
WAS	0.636829278	0.578199998	1
FRAT1	0.117938249	0.941517459	1
NEURL1B	-0.161250315	0.883437516	1
AFG1L	-0.409499564	0.687247587	1
STXBP4	-0.694925748	0.632202856	1
GALNT17	-1.29376007	0.391916427	1
IGSF9B	-0.352072472	0.879746045	1
TNFAIP8L1	-0.211390585	0.856299797	1
G34752	0.467703116	0.752903749	1
SPATA6	2.419947685	0.184298441	1
G1234	1.060854517	0.504911352	1
AKAP3	2.097676274	0.279253047	1
FXYD4	0.416919639	0.772465871	1
NEK2	-0.756754232	0.626800632	1
GLI1	0.134829308	0.890265806	1
ADAMDEC1	1.820710194	0.249070925	1
ZCWPW1	0.584590597	0.557967066	1
C15H11ORF42	0.936434693	0.409581593	1
CPNE5	-1.129515662	0.506209402	1
ENSBTAG00000044010	-0.459509466	0.757212961	1
BCAS3	-0.441661123	0.689476834	1
ENSMMSG00000010759	2.195593182	0.394605528	1
KDM1A	-0.412371358	0.704484968	1
LOC513767	-0.240513693	0.865097923	1
MNS1	0.856837383	0.531732761	1
KCNMB4	0.08309239	0.956256265	1
SNF8	-0.747776973	0.453002246	1
CYBRD1	0.284224936	0.792740536	1
HAUS3	-0.521042549	0.632022429	1
SARM1	-0.346830188	0.795804287	1
ENSBTAG00000046663	2.232573958	0.21304005	1
CBARP	-0.141157571	0.901321647	1
TCFL5	-0.005973466	0.996832624	1
CCDC57	-0.103375069	0.936131722	1
USP11	0.222406573	0.878830004	1
NRIP3	0.477079081	0.695417648	1
CDK2AP1	-0.108561415	0.91770526	1
DAPK2	0.543225922	0.7471327	1
RIBC1	0.010970528	0.996125787	1
CILP2	0.710020905	0.640352058	1
CYB5R2	0.445275634	0.788879133	1
DGKE	0.909869181	0.649765708	1
KCNQ1	-0.49321669	0.696085476	1
DIAPH2	0.737397222	0.590514918	1
ENSMMSG00000003764	0.82523001	0.435710701	1
NPHP1	-0.028547052	0.978437409	1
RGS3	-0.985041111	0.431136767	1
MIS18BP1	0.244805773	0.882194741	1
UNC5A	0.336444528	0.850973503	1

MAP6D1	0.512719492	0.702461667	1
SP4	0.00389751	0.998330532	1
LCOR	-0.738184455	0.655455647	1
SATB2	3.117394228	0.173116654	1
BMP5	-0.351696436	0.836400876	1
SYT7	-0.781252164	0.599783993	1
BAHCC1	-0.417641387	0.838293755	1
H4C6/H4C1/H4C2/LOC5267[illegible]	0.705332142	0.621734744	1
RARB	-0.607791488	0.620574294	1
RERG	-0.700712838	0.603021872	1
MARCHF3	0.096090766	0.92331945	1
CHL1	1.280053153	0.334469566	1
RIMBP2	-0.298368111	0.851852192	1
SHCBP1	-0.065805186	0.975856514	1
CATSPER4	0.900380788	0.544222388	1
KIF24	3.973671625	0.096045342	1
FIBCD1	0.405025793	0.837991448	1
FCRL1	0.948797546	0.580580972	1
BLM	0.037729413	0.976785067	1
WWOX	0.662687454	0.690087	1
OTOF	0.619570094	0.795929171	1
ZNF800	0.228319315	0.869183864	1
FAM151A	0.07847215	0.957947688	1
ZGRF1	0.783653168	0.670669357	1
SCN8A	1.923430955	0.296300398	1
THEM6	0.479595006	0.748151434	1
TMEM255A	-1.086849369	0.407329548	1
FRAT2	-0.08247116	0.948886816	1
HJURP	4.141420185	0.099483263	1
TMEM243	-1.113741985	0.368100856	1
LOC104968820	-0.530207835	0.699059871	1
ENSBTAG00000049216	2.193848731	0.222853206	1
STAG3	0	1	1
FLRT1	-0.395075069	0.800877253	1
RPL12	1.278715968	0.400679378	1
G22668	2.688461462	0.131567288	1
VASH2	-0.647873303	0.668665089	1
TSPAN11	0.568460113	0.753193994	1
FCGR1A	-0.294208107	0.756974303	1
CDH23	-0.832511448	0.588847024	1
SLC17A9	0.462199343	0.801818044	1
THEMIS2	1.199897281	0.204189247	1
PIK3CD	-0.404052539	0.775652181	1
SYN3	-0.569866587	0.69686866	1
UCHL1	0.575154872	0.751294789	1
TSPAN5	0.517345277	0.654479014	1
IER5L	-0.061681739	0.959894617	1
MSX1	-0.780963679	0.545988244	1
IFNAR2	1.988402936	0.295404193	1
SEPTIN1	1.473202387	0.2151289	1
CCM2L	0.144231676	0.926459624	1
FAM221A	2.202557069	0.174945065	1
PADI6	-0.598379218	0.637260421	1
FAM217B	0.43155689	0.761603523	1
AGMAT	-1.178628492	0.405785316	1
TBX6	-0.248366827	0.809227431	1
OLFM1	-0.694262925	0.686412903	1
RPRD1A	-0.720798002	0.586447456	1

ERAS	0.810617801	0.541207288	1
CD53	0.036973409	0.981849631	1
FNDC4	1.170645728	0.499041923	1
ENSMMSG00000011480	1.340090827	0.496518306	1
FAM228B	2.140239485	0.182340974	1
TAF4	-1.082449612	0.728423114	1
FERMT1	0.852285968	0.583595029	1
MAT1A	0	1	1
CDK14	0.741007902	0.670388671	1
CCDC157	0.235265859	0.824788037	1
MUTYH	0.877563161	0.73253569	1
TMEM138	0.054806039	0.95990256	1
BANP	1.037579731	0.482502788	1
SETBP1	0.271901285	0.856390066	1
GPR27	1.742992777	0.343071367	1
DPP10	1.044635899	0.608433793	1
RPL13A	-0.138091167	0.899560898	1
FAM180B	-0.950533245	0.519637118	1
CLGN	0.548386637	0.77635512	1
ENSMMSG00000015023	1.136089792	0.489571547	1
SFRP5	0.710446696	0.570385279	1
SNX6	-0.177148046	0.911624137	1
EXOC3L4	0.672004199	0.705811989	1
GON4L	0.310046529	0.829668043	1
ABI2	3.685968287	0.377243449	1
TTR	2.106264808	0.281183979	1
LIMS1	0.142840508	0.924571619	1
CCNQ	-1.085617587	0.413302511	1
PON1	1.946326219	0.387020437	1
ENOX1	-0.667511452	0.72163033	1
MBLAC2	0.559245127	0.744115349	1
CD40	-0.55847022	0.628247417	1
CCER2	2.374180024	0.145710026	1
CCDC51	1.106330938	0.35210808	1
ST8SIA4	-0.693061568	0.694256891	1
LONRF1	-0.034965773	0.981968399	1
AMMECR1	1.11874751	0.575411436	1
PHF21B	0.66681282	0.617402708	1
G33509	0.622549799	0.630351909	1
TBX4	0.321097655	0.831829312	1
ANKS6	0.083793799	0.961486669	1
G2080	0.405834387	0.81874312	1
CCDC174	0.261109714	0.869074265	1
MYH9	2.007821029	0.247643417	1
TRMT9B	0.93225965	0.495703712	1
RASA2	-0.010406235	0.995599064	1
TSPAN2	0.178892337	0.905988673	1
FOXS1	-0.284701871	0.89963194	1
ZFPM2	-0.533459628	0.700287052	1
NOX5	-0.375423491	0.846949183	1
ARHGAP19	0.11144463	0.9528474	1
HMGCS2	0	1	1
AK4	-0.510075097	0.701646181	1
P2RY4	-0.176717093	0.892154066	1
KCNK5	4.360251335	0.087814958	1
XKR8	0.446495974	0.775703555	1
TTC22	2.373209049	0.308225162	1
NT5E	0.536639962	0.562994652	1

GPATCH2	-0.278053098	0.851274001	1
HHIPL1	0.306181035	0.818610723	1
ADAM12	-0.298795562	0.852677682	1
LCORL	0.256659117	0.853315168	1
PLA2G4D	0	1	1
LOC534578	-0.54742028	0.695618496	1
G11490	-0.081348926	0.932731672	1
RPL19	0.232608088	0.861075078	1
ZC3H8	-0.863885298	0.504906233	1
EVI5	0.005168764	0.997754136	1
TRIM62	0.471797724	0.676430707	1
ABHD2	1.428268476	0.472148443	1
RSF1	4.290837527	0.09782691	1
LIN52	0.3336077	0.862628642	1
WNT2B	-1.344825048	0.328589051	1
C3ORF18	0.524979398	0.756358371	1
FZD2	0.135277556	0.908997423	1
ENSMMSG00000015887	-0.474447402	0.71454092	1
VDR	1.218835514	0.483376536	1
ZNF473	0.462101195	0.767986914	1
DLG2	0.486264863	0.803215228	1
KIF26B	0.185768213	0.922409196	1
C9H6ORF118	0.046487144	0.977994838	1
NR1I3	0.100008123	0.935342935	1
MAMSTR	-1.164320099	0.416777844	1
LOC531557	-0.469769259	0.707997371	1
TET1	-0.433926525	0.756866756	1
ENSMMSG00000017641	1.579996615	0.379631052	1
SCGB1C1	2.127520491	0.107406478	1
SGPP1	0.277464512	0.837467674	1
ENSMMSG00000014574	1.211645609	0.461377594	1
MTMR3	-1.024693939	0.432259641	1
AQP11	2.067855968	0.261495141	1
NMI	1.121824798	0.430904649	1
MAOB	-0.178344627	0.908645844	1
ZEB1	-0.852196911	0.33928371	1
LSM11	-0.725664922	0.548710341	1
CABYR	0.322282824	0.833987221	1
ENSBTAG00000051415	0.568344275	0.744927273	1
ENSMMSG00000002474	1.390484227	0.304858818	1
WDFY4	-0.33443642	0.857935164	1
NBEA	0.675693848	0.802922215	1
ZNF428	1.640515872	0.219559022	1
SOX7	-0.200737951	0.877216995	1
SOX6	-1.061208938	0.608301999	1
PTGIS	-0.892188689	0.489921595	1
B4GALNT2	0.143638355	0.905312613	1
SLC4A4	-1.07554465	0.428470254	1
DNAL1	-0.363163635	0.787792751	1
CUBN	-0.632719593	0.738045818	1
CCDC51	0.377587099	0.775689983	1
EXTL2	-0.572709421	0.598306886	1
PIK3R6	0.947675092	0.602988441	1
CDKN2AIP	0.271788738	0.835876699	1
CLEC12A	-0.117431586	0.927097092	1
G14500	0.928877709	0.527010648	1
CHAF1B	-4.296749321	0.165053448	1
MSR1	0.037570609	0.98414358	1

ENSMMSG00000002742	1.856186787	0.442050796	1
PTBP1	0.665880076	0.698715487	1
RANBP17	1.44522455	0.412118247	1
GLCCI1	0.330532684	0.83445322	1
SUSD3	1.390938441	0.418064317	1
FRMPD3	1.558427283	0.461005915	1
PENK	0.045932223	0.977594997	1
LAYN	-1.542606136	0.471561934	1
ENSMMSG00000010685	1.093505982	0.512823152	1
PDYN	1.631663385	0.293746482	1
B3GALT1	-0.094213113	0.950785761	1
JAZF1	-0.531216358	0.584964899	1
C8H8ORF74	-0.670727701	0.867585007	1
NGF	0.316843628	0.784692567	1
EEF1A1	1.310175773	0.368195386	1
CPLANE1	1.605739568	0.544101978	1
MRO	0.531663564	0.759030237	1
DEPDC7	1.594240527	0.397723814	1
ITGB3	-0.269436002	0.854553481	1
CTF1	0.587810834	0.683408058	1
IQSEC2	-0.672655928	0.629174963	1
RASGRF2	-0.91211982	0.417926902	1
SCOC	-0.688709035	0.614313763	1
ENSMMSG00000021833	0.471157517	0.744716663	1
CMPK2	0.758348738	0.663280284	1
PRKN	-0.399504544	0.814882292	1
BBS10	1.04562918	0.517241978	1
MCPH1	0.729752833	0.592293705	1
AP1S2	-0.403246861	0.770959936	1
C4BPA	2.352149765	0.267465084	1
STON1	0.040609303	0.969807578	1
TOM1L2	-0.575485936	0.722765159	1
HDAC9	0.847266858	0.625844664	1
OPRD1	0	1	1
SLA2	0.844421385	0.558643121	1
ZNF180	1.477240132	0.371087719	1
PRIM2	0.089942822	0.949134662	1
JAM2	0.101623559	0.946048589	1
CYBRD1	0.585089381	0.669292757	1
RBM11	0	1	1
G25561	0	1	1
TOR2A	1.404950846	0.550597666	1
HOXA6	-0.229909319	0.866898895	1
C1QTNF2	0.547535272	0.678445879	1
FAM126A	-1.009538366	0.400407104	1
G26503	0.002681701	0.998669572	1
KCTD21	0.12603185	0.933971291	1
RIMS3	0.711335116	0.717410111	1
USP49	1.753122518	0.519760107	1
GAB3	-0.762921678	0.548733962	1
NHLRC1	0.652817848	0.619511553	1
G6468	0.314443358	0.822491757	1
SYT11	0.985380782	0.716890053	1
NOVA1	2.994086654	0.137014023	1
NTN1	-0.720813669	0.629848976	1
DNAH1	0.078863171	0.951463269	1
RPH3AL	-0.450161954	0.750872729	1
GRAP	-0.687204905	0.620266229	1

ENSMMSG00000006725	0.680739692	0.599559461	1
CD1E	0.15136119	0.94419964	1
FHIP2A	-0.711682976	0.611410449	1
HEPHL1	1.441810991	0.269570371	1
LOC528802	1.036413004	0.534229647	1
SLC25A27	0.349497901	0.803176878	1
VTI1A	0.293236275	0.769236447	1
CENPQ	0.195186518	0.899581223	1
FGD4	0.7287561	0.672674623	1
ENSMMSG00000000204	1.318478639	0.433825342	1
CTBP2	-0.683300349	0.54694408	1
EBF4	1.317487749	0.428580466	1
CSNK2A2	0.746126179	0.57111355	1
ITGA4	-0.303729045	0.823679212	1
G35603	1.683728253	0.462694235	1
ENSBTAG00000052226	-0.889080826	0.313951742	1
KBTBD13	-0.993011996	0.426186616	1
SELPLG	-0.675664973	0.60808382	1
IL17D	1.909682624	0.198381994	1
KCTD19	1.095994494	0.514002615	1
LYPD3	3.032462839	0.188075694	1
NAPSA	1.21128133	0.331171888	1
APOA1	0.031825643	0.983033219	1
RASL11B	-0.441921447	0.757906135	1
ABCG1	0.017739857	0.992403453	1
PELI2	-0.539982432	0.781924668	1
MDGA1	-0.144684524	0.908825448	1
ZNF582	0.803324768	0.554605468	1
PIWIL3	2.377127276	0.316493788	1
ACOT8	1.319260647	0.303332769	1
G27655	0.144495497	0.921853075	1
TAGLN3	-0.320515483	0.738219869	1
C3H1ORF226	-0.128634808	0.925864776	1
DISC1	0.067865295	0.94603316	1
MYO3A	0.42792167	0.712860609	1
GNAQ	-0.478019309	0.727478826	1
VWCE	1.614449167	0.197523758	1
G24434	0	1	1
ENSMMSG00000009844	0.200773617	0.922660246	1
CCDC28A	-0.30618075	0.813096873	1
RIPOR1	-0.157865135	0.916361303	1
CD300LG	-0.070608767	0.958333202	1
ESR1	-0.617995834	0.662613206	1
TTC30B	1.298262361	0.41129491	1
ARHGAP36	-0.776353668	0.563299155	1
SLK	0	1	1
KIAA1958	-0.041973593	0.967301057	1
AMER1	0.744494484	0.655412439	1
MTM1	0.078212234	0.962031765	1
GRK6	-0.641576056	0.584788593	1
GNPTG	0.419288682	0.773183399	1
PRDM2	-0.585454781	0.744606376	1
NAT1	0.102267809	0.919471026	1
GALNT10	0.557054377	0.737917634	1
BCAS3	0.24453644	0.878747438	1
BORA	0.972723499	0.416322982	1
NTMT2	-1.506765644	0.297681404	1
SAMSN1	-0.083493649	0.953846524	1

PAG1	0.99365031	0.59442267	1
TMEM132C	1.430794921	0.316123429	1
TTPAL	1.11686368	0.481530434	1
FAM205C	0.941611142	0.463644803	1
PECR	0.069760851	0.966985134	1
GDPD5	-0.944543078	0.481989106	1
G2174	1.353932684	0.289255684	1
TIRAP	0.176722834	0.921597307	1
PCNX4	0.506296496	0.711283403	1
AKAP8	1.173742328	0.424683312	1
RBPMS	-1.422560218	0.523534398	1
STXBP6	-0.584264118	0.590170188	1
TMEM116	1.116963844	0.461980232	1
B3GNT6	0	1	1
SLC10A7	0.960366272	0.455645687	1
C14ORF28	0.562511425	0.64277935	1
ENSBTAG00000046939	2.224311039	0.183383555	1
BTBD19	0.573901376	0.642818831	1
HDAC9	-0.466189835	0.848532232	1
PITPNM3	2.510870696	0.239391206	1
SULT4A1	2.181279538	0.361744067	1
CCDC153	0	1	1
CEP41	1.302663709	0.179895838	1
G23919	4.087583317	0.109912445	1
MAP3K20	0.267076657	0.840775294	1
NDNF	-0.783377167	0.586667098	1
METTL21E	-1.151106671	0.291548993	1
PLA2G2C	4.21762714	0.085218404	1
ZFYVE9	-0.938308885	0.394404589	1
ROPN1L	2.898386807	0.225306195	1
KCNT1	-1.030022558	0.467895428	1
GNAT2	0.681475146	0.551755501	1
G38630	1.404674296	0.346584423	1
N4BP2L1	-0.118655857	0.90040943	1
ETV1	1.538844361	0.404230581	1
UBE2I	-0.31486885	0.790559689	1
FER	1.120688363	0.431324181	1
STARD3NL	-0.660253561	0.635829684	1
TTLL11	0.652069904	0.604229214	1
RNF138	1.321822633	0.286910164	1
PAQR7	-2.853837783	0.301644636	1
BRCA1	1.058705654	0.624706778	1
ZNF283	1.372459039	0.333370132	1
KRT24	0.403631818	0.852993592	1
AP1S2	0.100034144	0.943880046	1
GALNT18	-0.37824084	0.714146599	1
ZNF383	0.091708124	0.946699987	1
KIF18B	0.276729444	0.816495191	1
GSTM1	-1.012744724	0.398012571	1
DTL	0.74450539	0.705055677	1
TEAD3	0.590628757	0.748428183	1
DRC3	-0.639562837	0.659338951	1
RASGRF1	2.501991243	0.241484828	1
DRP2	-0.609586142	0.652820928	1
LOC100296105	0	1	1
MAML3	0.351372237	0.7096803	1
PARVG	-0.10641553	0.925562261	1
KLRG2	1.80254548	0.262313833	1

TMEM100	-1.173147685	0.418826233	1
PAPSS2	0.515205511	0.642681032	1
GJB5	1.454979831	0.532001788	1
PM20D1	-0.566961076	0.670526735	1
CDH4	-0.570748434	0.682492168	1
ABCB1	0.154440855	0.921029593	1
SLC35D2	-0.579217005	0.686497457	1
GINS3	-0.464008209	0.80367869	1
GULP1	-0.690901956	0.60931128	1
CCDC85C	0.996761989	0.615513307	1
LCMT2	0.983794963	0.467292966	1
PIH1D2	1.048980578	0.459984325	1
DESI2	0.446489642	0.705224806	1
APOBEC3H	1.797579057	0.159643857	1
KIAA1328	1.832016785	0.379205307	1
MDM1	1.164529893	0.506383234	1
CCNJ	1.581552423	0.329425453	1
KCNAB1	0.284823876	0.868552669	1
PROCR	-0.764989389	0.564487761	1
GREM1	2.193693014	0.270143072	1
STOX2	4.11827491	0.109980934	1
ALDH1L1	-0.364763091	0.824873788	1
G36303	-0.323280604	0.820559971	1
LTC4S	2.729753802	0.159583195	1
ACAD10	-0.170960299	0.901855638	1
FAM13C	-0.417393869	0.81927691	1
ZC2HC1C	0.39233311	0.745747078	1
PPARG	0.954594701	0.552948411	1
ZNF200	1.006382515	0.537409946	1
CAPN1	0.598098317	0.674880754	1
RRP12	-0.523964256	0.717587161	1
LTBP1	-0.432855032	0.806237071	1
ZNF568	1.112603697	0.659709525	1
PTK6	2.446165827	0.087559926	1
HEY2	0.311031861	0.843817697	1
KCNC3	0.369598912	0.796382667	1
HMBOX1	1.809704657	0.335952067	1
GPER1	0.210332882	0.880118747	1
PFDN6	1.251056551	0.357165992	1
OMP	0.80961456	0.557405236	1
ZMYM1	0.105618685	0.945071566	1
JAK3	0.978316319	0.502912973	1
TEC	1.05918382	0.523204006	1
SLC38A8	0	1	1
ABHD16B	0.290945539	0.831425841	1
ZNF184	0.177129767	0.872786281	1
HCN4	0.402109609	0.867312809	1
RPL23A	1.162707082	0.396115935	1
POU3F3A	-1.814801686	0.553762851	1
CYP1B1	-0.485741123	0.751228268	1
ALKBH8	-0.466401616	0.72351789	1
CLCF1	-0.194772122	0.8937907	1
FZD8	-0.538041913	0.700856882	1
USP45	-0.62021918	0.644908295	1
NCK2	-0.412434919	0.799182531	1
ENSMMSG00000021626	0	1	1
PDE9A	0.85127511	0.698517139	1
APAF1	1.844970891	0.363041556	1

SYT1	-0.011092644	0.995282565	1
FGFR1OP2	-0.282772702	0.853391112	1
SOAT2	0.056709868	0.969369735	1
HERC6	-0.690594278	0.706814344	1
MAP4K1	1.449241722	0.223024186	1
SYNJ1	2.744010537	0.510749614	1
SYTL3	0.184038159	0.896592586	1
NSD3	2.537528958	0.272702559	1
SHLD1	1.756985842	0.419290646	1
CGREF1	-0.653436181	0.602156176	1
ZKSCAN5	-0.2765344	0.827547862	1
VEPH1	2.176065671	0.335885235	1
ENSBTAG00000050322	-0.132868284	0.910750482	1
RAD54L	2.937146707	0.137274552	1
PRDM5	0.942045153	0.535723793	1
NRCAM	6.85477811	0.089959633	1
GZMM	-0.481829493	0.700785867	1
SHOX2	-0.929995892	0.530195677	1
SMRP1	-0.568460058	0.705464459	1
TRUB1	0.533792302	0.700339572	1
ZFAND4	1.468282945	0.358835743	1
LRRC29	1.039545741	0.443413283	1
CDR2L	-0.63437122	0.61548466	1
SERPIND1	1.088791804	0.53852735	1
STRADA	1.822712512	0.402149085	1
NUS1	0.194909265	0.891712021	1
BIN2	0.265413717	0.818355521	1
MMP16	-0.046572843	0.9725662	1
C19H17ORF113	-0.788938889	0.637640949	1
C1QTNF7	0.489103681	0.702817438	1
VPS13A	1.265393092	0.456180516	1
NDC80	2.594778293	0.235935574	1
ST8SIA2	0.545847243	0.742183993	1
AFG1L	-0.406663238	0.78049767	1
RHBDL3	-0.348927287	0.81434894	1
THUMPD2	1.298090026	0.447413473	1
ELMO1	-0.411835464	0.760153991	1
LARP1B	1.118080246	0.576874556	1
HERC1	-0.232097941	0.916989872	1
ENSBTAG00000045892	0.393966213	0.798666357	1
CCR1	-0.610588022	0.657933845	1
HOXA5	-0.608813745	0.652039923	1
PABPC4L	0.020430487	0.984699907	1
SLC3A1	1.427745685	0.328343619	1
G34946	1.637018855	0.295662355	1
XRCC3	1.113752401	0.509554183	1
NR2F1	0.109766703	0.940164503	1
FBXL22	0.346221901	0.838275845	1
ATOH8	-0.575847474	0.717528848	1
NLRX1	0.208973529	0.853208413	1
ZDHHC15	3.885524059	0.112479007	1
ZNF689	-0.351850136	0.820082349	1
SASH1	0.238244347	0.87653013	1
PAQR8	1.791430976	0.292174092	1
ANGPT2	0.323909502	0.836486079	1
POF1B	3.225124136	0.263554521	1
SEMA4G	0.192843832	0.899708478	1
OTOA	0.257659566	0.883932836	1

RADIL	-0.26868463	0.859421224	1
C18H16ORF74	1.469288201	0.181920151	1
ZNF26	-0.19570599	0.895128986	1
UBE3D	-0.614423163	0.669145146	1
TMEM170A	0.870322884	0.543685035	1
RPL4	2.236631727	0.137599435	1
PCDHGA11	-0.06344507	0.965049962	1
ASB7	0.02105328	0.988363191	1
SEMA5B	-0.957925238	0.451951575	1
ELN	1.36425719	0.462327329	1
AOAH	0.53775993	0.747384805	1
HKDC1	1.014569325	0.411217415	1
REELD1	1.277122052	0.433567217	1
CPZ	4.172338633	0.158435466	1
TMOD2	-0.481968246	0.731116542	1
LOC509006	0.802585051	0.503098419	1
BRSK1	0.19694897	0.908416976	1
HIP1R	1.080620949	0.628134962	1
CHD5	4.090015987	0.093830923	1
DNASE1L2	1.494025189	0.241748127	1
RALGAPA1	-1.051698161	0.460151926	1
ENSMMSG00000021783	-0.091100846	0.952406412	1
DNAH3	1.225792414	0.363219121	1
IKZF2	2.004893144	0.242995363	1
ZKSCAN2	-0.265045028	0.854259699	1
CEP135	0.868669885	0.583900423	1
MFSD6L	2.157248965	0.220192317	1
SAMD1	0.279564045	0.83599207	1
ITPRID2	-0.260463291	0.853370326	1
TAS1R1	-0.89585874	0.465497618	1
REEP2	-0.748744238	0.596562411	1
TWIST2	1.119113684	0.38149772	1
MAST4	1.440411608	0.398979039	1
ENSMMSG00000013153	0.628697718	0.699758919	1
HELLS	1.779271849	0.338739304	1
SERF1A	-0.016717248	0.988522591	1
METTL25	0.070710127	0.955460192	1
AIRE	0	1	1
TOMT	2.13161604	0.267847953	1
HENMT1	0.937235134	0.562641037	1
CHRD	0.151206906	0.917072475	1
DNPH1	0.626809965	0.623428343	1
TMEM47	0.57639878	0.673017352	1
RUBCNL	0.092031118	0.947690263	1
RBPMS2	-0.63517587	0.616114455	1
G3747	0.512282645	0.787725906	1
IGF2R	0.329354658	0.831428823	1
FAM171A2	0.278613601	0.839039821	1
ENSMMSG00000002661	1.108697858	0.449017834	1
AFF2	1.798990126	0.370081512	1
SIM1	0.347251125	0.89529278	1
ECT2	0.907197702	0.4873776	1
KIF21B	0.466303402	0.69613826	1
PTGS2	-1.112201858	0.543094699	1
ZAR1L	0.64662255	0.692523567	1
NOS2	-2.755347484	0.290944219	1
COL6A5	0	1	1
STUM	-1.924793995	0.5429033	1

PHC2	-0.950675205	0.516300597	1
DNAL4	0.37857801	0.685771913	1
ZNF25	0.944759989	0.56634498	1
NAAA	0.119767092	0.906836534	1
TAGAP	-0.706385478	0.590781655	1
CRYM	1.318047588	0.433834624	1
IGLON5	0.935899295	0.610410483	1
SLC24A1	1.299598255	0.380133994	1
SCLT1	-0.626358764	0.637872779	1
CCN5	1.007938974	0.48482204	1
LOC523461	-0.102620564	0.937860993	1
G37914	-0.210607558	0.884752273	1
SPAG1	-0.071205754	0.934756563	1
CRIM1	0.453034839	0.756596394	1
ENSBTAG00000002675	0	1	1
IL20RB	1.103344406	0.382225675	1
MAP3K8	0.175295301	0.89938179	1
SAMD10	0.36900459	0.793065652	1
BAMBI	1.700291457	0.407539877	1
ZFHX4	-0.628296486	0.697604704	1
SLC4A8	-0.958580625	0.449279012	1
NOS1AP	-0.217903152	0.868154652	1
TEX30	-0.255080215	0.834619592	1
GJC1	-0.633833849	0.633257494	1
ZDHHC21	2.542198908	0.114391419	1
NPL	2.19645144	0.379383077	1
RALGPS1	0.394279537	0.846434738	1
GTPBP8	-0.889923289	0.640106324	1
DAAM2	1.09648157	0.680692746	1
G8614	0.622619257	0.734859952	1
VSTM4	-0.385454822	0.766550341	1
RNF44	1.79602985	0.310866477	1
TMEM80	0.706927707	0.562715717	1
RFXAP	-0.314560155	0.823951	1
LOC540707	0.472757588	0.832869509	1
TMTC4	1.906086048	0.271534026	1
LOC789175	0	1	1
PRKCE	-0.54191177	0.722288666	1
SUPT4H1	0.351089651	0.808199813	1
LOC505052	-5.375375922	0.197723164	1
CNBD2	-0.305315628	0.78011619	1
ARL15	1.006486159	0.507003567	1
GDPD1/SMG8	-0.104538838	0.93562672	1
B9D2	-0.631643249	0.568193006	1
ENSMMSG00000014604	0.025627686	0.98098871	1
ARHGAP22	-0.880429347	0.364405233	1
VASH1	-0.040103081	0.975894171	1
BOLA-DMA	0.347475229	0.804374579	1
APC	-0.361831234	0.776721614	1
EXOC6	1.775691851	0.303760156	1
KIAA1755	0.642489405	0.664088623	1
MSTN	-1.216677297	0.389687034	1
BEND3	0.719623963	0.614674343	1
STMN2	-0.758727681	0.646307244	1
CFTR	3.997958408	0.151382084	1
RAD51	1.066019792	0.402397695	1
PTAR1	-0.148873985	0.91504211	1
PIK3R3	0.423536071	0.783935428	1

CRIP3	0.089796481	0.948189915	1
ROBO2	2.637938098	0.407966788	1
LAYN	0.782546974	0.582450744	1
SPI1	0.847243455	0.712760676	1
ATP2A2	0.898082049	0.521744907	1
SYN2	-0.47353527	0.600668444	1
FAM184A	-1.823358408	0.454951489	1
TASOR	0.622169962	0.687575485	1
MAP3K7CL	-1.308898032	0.319292259	1
POU6F1	-1.052283243	0.37542148	1
LOC784451	-0.973207666	0.479066903	1
CEP70	1.039718384	0.42020655	1
BBS1	-0.778512388	0.717862678	1
ENSMMSG00000024133	-0.836681512	0.513059206	1
DCAF10	0.417036818	0.749082613	1
RGS19	-0.246029569	0.842670933	1
HS2ST1	0.423877529	0.775350113	1
ROBO2	0	1	1
TAF3	-0.717005998	0.434102305	1
CNKSR2	0.439383188	0.788367287	1
SIRT6	0.767774036	0.638913507	1
ZNF311	1.61381535	0.266494969	1
HIGD1B	0.743520529	0.520096883	1
ENSMMSG00000009708	1.105033538	0.390991946	1
TTC21A	1.445520517	0.14913949	1
RTN4RL1	-0.314124118	0.814147433	1
TMEM81	-0.298454925	0.768953598	1
NACAD	2.746830417	0.143263332	1
MTMR7	-0.922808725	0.438751831	1
GLIPR1	0.288494639	0.813783823	1
MAGI1	0	1	1
COQ2	-0.120169912	0.927722051	1
G6855	-0.155876912	0.870988166	1
PEAK3	0	1	1
SERPINI1	0.197267609	0.858293465	1
C19/H2BC13/H2BC17/H2BC	1.624026533	0.3242186	1
PRDM12	-1.823183262	0.456567792	1
ENSBTAG00000009664	0.796458756	0.688176918	1
CPNE9	-0.170839355	0.87166936	1
EBF1	3.180690366	0.174555124	1
P4HA3	-0.907290399	0.461925647	1
MELK	3.480043613	0.145378915	1
G22602	0.912637494	0.486322133	1
XRRA1	0.190511176	0.828811517	1
CEP170	4.063795744	0.111735096	1
B3GALT6	0.038665466	0.977484389	1
PJA1	-0.936256289	0.367339491	1
ABCA4	1.813573441	0.175802655	1
PHF24	-0.034855414	0.980639657	1
ZNF283	1.478209855	0.232178611	1
OR10AH1	0	1	1
HOXB6	0.50938234	0.692315871	1
MAX	-0.960341858	0.413187548	1
INTS6L	-0.065536459	0.96541188	1
STK39	-0.481536825	0.648651718	1
ZNF154	0.205711481	0.891247424	1
GNG2	-0.530644445	0.631903005	1
WDFY3	0.205283835	0.898958952	1

ZSCAN12	-0.342961036	0.808285914	1
SLC13A5	0	1	1
TP53I11	-0.626953459	0.587795797	1
NTF3	1.564083395	0.274763116	1
CDK5R1	1.010492633	0.465559181	1
E2F3	-0.932481094	0.48003672	1
ZMYM4	0.328643853	0.832758496	1
ST7	2.083679003	0.268223605	1
SMOC1	-0.939441383	0.490589875	1
INKA2	-0.89439431	0.399403672	1
LYPD6	-0.139985364	0.931700358	1
COIL	-0.456768351	0.684038855	1
NFKBID	0.828400269	0.42905289	1
CAMKMT	0.197405024	0.904745089	1
SPATC1	2.188142029	0.375886756	1
ATP5MG	-0.68606446	0.544792148	1
NRP1	-0.793331899	0.534806409	1
ARMCX1	-0.443597682	0.752068859	1
KCNQ4	-0.607942866	0.666676858	1
ACHE	0.582639825	0.739084973	1
C19H17ORF98	0	1	1
HPSE2	1.515341263	0.485309642	1
ENSMMSG00000018694	0.396353375	0.77198599	1
SHC4	2.295299828	0.187231019	1
PLA2G4A	0.215588757	0.841683006	1
CTSL	-0.388281239	0.788264578	1
CA5B	0.464752348	0.749616993	1
NCALD	-0.641963817	0.655326047	1
ADRA2B	-0.903036471	0.499484722	1
PNP	1.0007131	0.453746057	1
S1PR2	-0.444801897	0.761396634	1
RPL12	1.039774316	0.472631034	1
DZIP1L	2.514109736	0.158823763	1
ENSBTAG00000015176	0.050358828	0.955635638	1
B3GNT9	-0.077707856	0.957366961	1
ZNF557	1.183332135	0.456508545	1
ENSMMSG00000018124	0.511105908	0.671262704	1
ENDOU	1.158834617	0.374418853	1
PBRM1	-4.561051199	0.274666512	1
CCDC112	1.986029859	0.244513209	1
MYOM1	-0.44590307	0.783963146	1
ENSMMSG00000012378	1.222581971	0.568023556	1
GNA14	0.352228783	0.762673233	1
AJM1	0.462043945	0.776842898	1
LOC510860	0.000340407	0.999775135	1
SLC19A2	0.673311587	0.676246316	1
LRRC34	1.674866595	0.336268419	1
SMYD3	0.134238094	0.922782738	1
FBXL2	1.586719953	0.444358259	1
PBX3	0.649226818	0.572051636	1
SNRPE	0.006002716	0.995725155	1
ZNF449/LOC112441465	0.670223026	0.696166605	1
ANLN	0.104393204	0.929590796	1
SYNGR1	0.235260631	0.88672506	1
CCDC85C	0.68722752	0.619075479	1
EBF2	-0.876917156	0.485757579	1
G2E3	0.618330489	0.57580921	1
SH3BP2	-0.772755188	0.607465999	1

RASAL2	2.513643944	0.282663983	1
MPV17L	2.978610604	0.167928671	1
SNX24	-1.003536791	0.439879827	1
ENSMMSG00000010271	-0.257407924	0.896537035	1
RCC2	0.966071299	0.356596052	1
ENSMMSG00000020225	0	1	1
RGS11	0.911352188	0.46809207	1
BOLA-DRB3	0.766664649	0.547022426	1
G24748	1.142091625	0.377276948	1
SERPINB6	0.111331723	0.939871258	1
VSIG4	-0.9697452	0.667262572	1
SFRP4	-0.725806186	0.574715052	1
ACAP2	0.724654054	0.625947886	1
ZNF613	-0.415051677	0.73458847	1
DAB2IP	-1.528082174	0.565721456	1
RPL12	0.965432731	0.488471272	1
DOCK3	0.777963737	0.46131956	1
DLG2	0.899784896	0.723678495	1
ACSM5	0.105119188	0.965740715	1
ENSMMSG00000019874	0	1	1
ENSBTAG00000053131	0.331225931	0.827649877	1
PLPP6	0.943272078	0.436163456	1
TLE4	-1.831590269	0.660316024	1
FAM185A	-0.391412241	0.778530867	1
ENSBTAG00000031246	1.234724299	0.306276535	1
LYRM2	-0.702675108	0.6153201	1
OGFRL1	0.695977164	0.60403694	1
HORMAD1	0.739672436	0.619469597	1
KIF4A	1.909688418	0.182500315	1
GNG12	0.809370461	0.512292796	1
XPA	-0.70832888	0.48968156	1
TSPAN33	-0.453728769	0.768804859	1
G28646	0.071835991	0.942198191	1
TRAF5	0.053363491	0.97106836	1
CEP63	-0.070599664	0.959605277	1
PCSK1N	2.155418415	0.222380083	1
CASP4	0.341625999	0.750646956	1
MXI1	-0.382214653	0.789911085	1
FAM124A	-0.129556959	0.940910251	1
SMG8/GDPD1	-0.262610989	0.846398531	1
NRDC	0	1	1
G10535	0.419450232	0.823365917	1
HMBOX1	0.453249319	0.708037327	1
DUSP14	-0.380423673	0.690121001	1
RALB	-0.487190587	0.666525296	1
G12422	0.23894943	0.856289412	1
XYLT1	-0.228987286	0.867293367	1
G36634	-0.81024294	0.545484153	1
NAV3	-0.28590701	0.832051832	1
CNIH3	-2.849681732	0.155492335	1
CENPU	0.076637892	0.963166677	1
ATRX	-0.065365168	0.972015645	1
TARS3	1.363691091	0.456864087	1
CYP3A4	-0.733827909	0.860330933	1
PRDM5	3.733938179	0.146250379	1
GRIK3	0.569169917	0.721336307	1
DOCK2	-0.073568878	0.959968433	1
KITLG	-0.453216874	0.802411485	1

ADCYAP1	-1.890221565	0.438026948	1
GCK	0.41860365	0.823572167	1
FAM167A	2.34527956	0.300137324	1
SLC44A1	-0.488547028	0.705469843	1
PROSER3	-2.227869394	0.392286145	1
NUSAP1	1.422529274	0.444689843	1
CDK11B	-0.726212181	0.508857923	1
NPAS2	1.52657325	0.195854916	1
POLR3G	2.715130914	0.085215062	1
IGSF9B	-0.338240682	0.806838468	1
MAP4K5	-0.461613517	0.849954115	1
TIA1	1.335049867	0.561407254	1
SLC25A19	-0.30285118	0.832792769	1
RAP2A	0.674514964	0.545509202	1
SNX9	-0.018505483	0.989127312	1
CHEK1	1.395945059	0.457999569	1
APC2	1.321467794	0.403883015	1
ARL6	0.384840249	0.797707866	1
SPTY2D1	0.193050923	0.889807973	1
BRIP1	-0.667014789	0.709164956	1
CHAF1B	1.121032253	0.327622319	1
APAF1	2.337603669	0.177411852	1
NIPAL4	0.982145344	0.457539628	1
CD200	-0.304578353	0.830493621	1
G24500	0.004919458	0.996544096	1
UBE2V1	0.020140928	0.99097067	1
IRAK3	0.019854502	0.989650521	1
NALF2	-0.421564261	0.748077081	1
CPE	1.363056523	0.280026618	1
MTCP1	0.956210122	0.428805038	1
FASTKD3	0.22219212	0.881841678	1
GPX7	-0.845286296	0.446597608	1
CYRIA	-0.050476084	0.951555108	1
CRYBB1	0	1	1
TRERF1	0.660379679	0.521105237	1
MID1	-0.009872372	0.994308671	1
ZWINT	-0.033189538	0.980094439	1
CENPI	-0.225574933	0.872457298	1
OSBP2	-0.512943332	0.645691046	1
SOX17	-1.122798529	0.262296291	1
RNFT2	0.918665043	0.591387616	1
SEMA6B	-0.797425071	0.530787815	1
PPARD	-0.330853677	0.828734077	1
DCXR	-0.483191925	0.804954201	1
GK	1.504787418	0.463169659	1
RNF112	3.812658057	0.156586364	1
PRKAR1B	0.736788808	0.668006319	1
RNF212B	3.522263857	0.082151527	1
EPB41L3	4.341962471	0.112958191	1
RABGAP1L	-0.726053184	0.583315434	1
EHD3	-0.191982763	0.887791797	1
CASP8AP2	0.636507107	0.679397807	1
RAPGEF5	-0.347519388	0.772458497	1
RANBP17	2.246096227	0.267634179	1
PDE1B	1.871311803	0.216669222	1
CUSTOS	-0.307845069	0.720653959	1
RFX7	0.19492481	0.898112968	1
HABP4	0.835147857	0.529010991	1

SFXN1	0.024053001	0.986639973	1
PABPC1L	0.834515885	0.486136959	1
CHN2	-0.376320011	0.681971011	1
ENSMMSG00000001877	1.130773873	0.308598983	1
PLCB2	0.899438827	0.626734686	1
CPEB3	1.444813885	0.550077304	1
G20776	-0.291916748	0.826685273	1
PCBP3	-0.403241957	0.754934432	1
ERBB4	3.299830628	0.164833114	1
BBS1	-0.648101792	0.73962653	1
NTN5	1.385779372	0.276586825	1
TTPAL	1.67363627	0.304239138	1
G12629	-0.761431958	0.601940289	1
PATL2	0.887881868	0.547816822	1
KCNJ3	-0.76056714	0.64315701	1
ZNF283	1.990623631	0.127933864	1
CUTC	-0.765521732	0.52814197	1
GSDME	-0.009107134	0.99499715	1
IFT22	-0.472583182	0.753778993	1
ARPC5L	-0.721964228	0.488340975	1
ENSMMSG00000000433	-0.095348595	0.92332708	1
ABCC4	-0.139608633	0.921307507	1
PHACTR1	0.384333328	0.814042869	1
ENSBTAG00000055293	1.862562665	0.17687041	1
PTPRCAP	1.473351844	0.190708104	1
HOXC8	0.474772727	0.658619503	1
FZD8	-0.216407047	0.890796092	1
MTHFD2L	1.127133611	0.494667181	1
GPM6B	0.173710969	0.902889648	1
SMIM10	-1.92526559	0.544436014	1
MORN2	0.050289314	0.960519754	1
NLRX1	0.149276928	0.91970125	1
RTP5	0	1	1
TMEM168	1.022389756	0.519711448	1
KCNN1	0.438109822	0.735282372	1
HSD11B2	0.151503813	0.908671637	1
COL8A1	0.273007348	0.865177974	1
LINGO3	0.938777081	0.656169586	1
LAMA1	0	1	1
ADAMTSL1	2.058507678	0.309637659	1
DNAJC18	-0.976343534	0.33501146	1
GNG7	0.634809249	0.700853848	1
PLSCR2	-0.904163671	0.513122523	1
DIS3L	-0.565358711	0.680420424	1
PHACTR2	3.165411935	0.142918433	1
KIAA0895L	0.046349238	0.974805983	1
ADAMTS4	-1.119931767	0.478922859	1
CPA6	1.312096769	0.575101187	1
LMBR1	0.486857142	0.735358201	1
G31177	4.571143057	0.08681771	1
G26552	-0.335211548	0.732094114	1
ENSMMSG00000022208	0	1	1
TRIL	0.440709534	0.71550713	1
PDE3A	-0.348196549	0.664233117	1
HPSE2	2.559833823	0.294989574	1
NTN3	0.928408801	0.420115265	1
MZT2	1.108436736	0.373677021	1
PDE1B	3.05757326	0.091525504	1

PPP1R9A	-0.633809404	0.706043021	1
NOPCHAP1	0.075779933	0.957527535	1
WFIKKN2	-0.105618812	0.947623029	1
MEIS1	2.634733982	0.190744184	1
SPSB1	-0.787641317	0.375073592	1
COL11A2	-0.460237262	0.716642459	1
RPS13	0.830111238	0.613717206	1
NUP42	0.279431997	0.845584182	1
RANBP3L	3.734403336	0.127057443	1
VEGFC	-0.035479149	0.978364457	1
TTYH1	-0.509690592	0.655870196	1
ITSN1	-0.624401128	0.657721713	1
MINDY4	0.259808886	0.800730708	1
APBB1IP	1.300005776	0.496024978	1
MGC134040	-0.218585407	0.871415147	1
CPSF4	0.527239532	0.739355281	1
NPAS1	2.098688887	0.217611465	1
CCHCR1	3.539227484	0.099632631	1
CSTB	2.607903341	0.519762136	1
ESPL1	1.092056395	0.575491918	1
ENSBTAG00000033169	2.60549452	0.244838489	1
ROCK1	-0.217937677	0.880474523	1
EEF1AKMT3	0.698170469	0.62769058	1
RDH12	-2.954882611	0.237142932	1
ALKBH2	-0.84936751	0.525207317	1
ADAT2	1.069145303	0.513787281	1
ENSMMSG00000006412	-0.008886904	0.994749731	1
KLHL28	-0.265385086	0.797799487	1
ITGA11	-0.57459106	0.659243283	1
KCTD13	-0.312601539	0.819687037	1
ZNF527	0.317264235	0.834563076	1
ZNF644	0.392736601	0.803432201	1
MBP	-1.106297567	0.365088815	1
B4GALT1	0.358637817	0.804532497	1
ROR2	0.229024614	0.854188551	1
ERCC8	1.331883383	0.296506395	1
A4GALT	-0.495052058	0.593663569	1
NR2C1	0.255804881	0.867988676	1
LOC101903385	0.369792449	0.670173826	1
FNDC11	2.673644851	0.138367813	1
TBXAS1	0.343187693	0.761345988	1
DOK7	-0.248399932	0.88578526	1
TDRD9	-1.95873895	0.666360463	1
SRPX2	0.143579241	0.910567443	1
PCYOX1L	2.084373818	0.14587904	1
TMEM42	0.573409756	0.49073761	1
PRSS8	1.269701063	0.238727668	1
ZNF787	-0.119419899	0.917445361	1
MOB3C	-0.673080177	0.520347938	1
DNAJC27	0.398778083	0.817047691	1
SOD3	-1.066407844	0.458100272	1
PHETA2	-0.293290045	0.843358833	1
CENPJ	0.921964598	0.521199487	1
CCDC174	-0.358411627	0.784774453	1
MAP2	0.471111296	0.753265534	1
EXOC3L2	0.967474851	0.340937275	1
ASB7	0.085730164	0.950779305	1
TTC23	0.299918569	0.85051603	1

TMEM135	0.358008796	0.810021406	1
TMEM82	-0.826238853	0.448347054	1
KAZN	-1.827110906	0.422982551	1
MEIS3	-0.451879691	0.749101616	1
NECTIN3	-0.894699647	0.41933994	1
MYO1G	0.314940389	0.802256661	1
UNC80	1.782098811	0.408621428	1
C17H5ORF52	1.302775379	0.30531343	1
ENSBTAG00000019266	-0.930226842	0.606058475	1
RBM24	-0.093718781	0.956066607	1
ARHGAP4	-0.302482469	0.774192663	1
CCDC141	-0.174450288	0.922468653	1
DENND11	0.263302118	0.865886399	1
ENSMMSG00000003690	1.076608495	0.450383755	1
SLC38A6	1.994709569	0.226768933	1
CDK6	0.324088698	0.753033548	1
FAM241B	0.99953996	0.488497756	1
CXCL3	0.103486196	0.923828409	1
TMEM176A	1.201154206	0.341287035	1
PHACTR1	-0.076788771	0.960812958	1
NRARP	-0.462552222	0.697778575	1
LRRC49	-1.270393517	0.362017092	1
KCNE2	-0.043929073	0.975423311	1
ENSBTAG00000009290	0.712436828	0.6707725	1
RLIM	0.549414554	0.771152083	1
RCN1	0.403370193	0.791283223	1
KNSTRN	-0.213614427	0.877889706	1
SCAPER	0.401968184	0.79516564	1
ALG6	0.316183684	0.831805233	1
KLK8	0	1	1
FBXO10	0.05340276	0.972497518	1
EVC2	0.531211369	0.679424305	1
SHC2	1.320289561	0.374581416	1
SLCO5A1	-0.116319976	0.93061504	1
ENSMMSG00000009617	-0.604160161	0.574855619	1
IK	-0.040902015	0.971329048	1
SOX8	-0.055183352	0.975072555	1
PPARGC1A	-0.796055237	0.524325009	1
RBL1	0.590772302	0.745951756	1
NEMP2	0.037003193	0.98047329	1
ZNF287	1.039615661	0.540066456	1
G5289	0.179147137	0.877124591	1
TMA7	0.119166801	0.924045668	1
G20610	1.121957702	0.618146837	1
DUSP22	-0.027876258	0.986425781	1
IQGAP3	1.160177644	0.511853324	1
ZNF436	0.368418307	0.797434557	1
CRHR2	0.019901594	0.990115631	1
KATNBL1	0.532756874	0.697000988	1
WDR76	0.293492532	0.784702849	1
G34614	0.021708101	0.991190904	1
CCDC136	0.463041524	0.703307253	1
PCDH10	1.598568551	0.382536413	1
CH25H	-0.435557562	0.681232054	1
RT1-BB	0.326133534	0.813025579	1
ZBTB9	0.836819248	0.566929333	1
LOC519132	0	1	1
ARAP2	0.880200138	0.403747629	1

HCK	-0.058123105	0.957220478	1
NUMBL	0.086193003	0.925715253	1
ARMH4	0.896014185	0.699629041	1
PHC1	0.458089525	0.849280674	1
MST1	1.26088876	0.251410103	1
STK17B	0.66073395	0.555696973	1
WASF3	-0.417824388	0.827644033	1
SNTG2	-0.86705861	0.700802969	1
ENSMMSG00000002762	0.942133746	0.566209	1
GPAT2	-0.968473964	0.456293116	1
LRRC3B	1.709317512	0.327494953	1
DDC	-1.224187246	0.248782645	1
ICAM1	-0.204143689	0.884220035	1
PLCH1	2.514719428	0.094969519	1
C5H5ORF22	0.328942803	0.83022439	1
SEMA6D	-0.12568338	0.932636655	1
CPB2	1.048357445	0.519707646	1
IFFO1	0.053815665	0.971377247	1
RPS2	2.835545763	0.097337057	1
G5121	0.861166147	0.582425527	1
DIPK2A	0.53171297	0.72184347	1
G352	1.821462264	0.237580553	1
CMKLR1	-0.726443393	0.584057716	1
SEMA3G	-1.115054718	0.568476896	1
URI1	1.230591809	0.452607284	1
NTAQ1	1.30129465	0.467244269	1
ENSMMSG00000023131	1.625205733	0.267441141	1
HOXB2	-0.123351553	0.90791628	1
MACROH2A2	1.568852093	0.267951579	1
TMEM268	0.247689053	0.866174641	1
MGC137036	2.333957753	0.167215547	1
PLK1	0.204067404	0.887687068	1
SPIRE2	0.868658583	0.6517888	1
TEX9	0.418084868	0.783144714	1
WTIP	-0.744616753	0.588487438	1
UBP1	-0.49005961	0.653605622	1
IMMT	0	1	1
SLC9A7	0	1	1
ZNF805	-0.003870536	0.997804749	1
RPH3AL	-1.038785484	0.430992788	1
SHANK3	-0.000982547	0.999502066	1
CFAP73	1.288545679	0.255179164	1
EXOC3L1	0.474516742	0.697951641	1
FBXL7	-0.476821251	0.735109026	1
MTFR1	0.624400596	0.660148681	1
MGC137055	-0.794849858	0.3769077	1
MLLT3	0.514206823	0.703047481	1
FGFBP1	0	1	1
RGS6	-0.265395335	0.812339424	1
RPL7A	0.709766207	0.460805658	1
RPL12	0.323377207	0.830207193	1
TGFB3	-0.856666023	0.497636772	1
ZNF346	1.023404685	0.524333698	1
ATP7B	0.048918897	0.970386373	1
PLA1A/ADPRH	0.293435592	0.780393372	1
C17H4ORF46	0.12755234	0.932839124	1
KCTD11	0.553113889	0.600615888	1
LOC768255	0.08791442	0.955790591	1

AMN1	-0.431157458	0.74322913	1
RGS10	0.382390764	0.782929452	1
RPS3	0.896878096	0.509847725	1
PARS2	0.910018693	0.571182109	1
CDKN2D	-0.778752179	0.517418256	1
RIBC1	1.950068112	0.355605794	1
ADGRL2	-0.372807596	0.749260602	1
MYO7A	-0.221944993	0.873133101	1
LOC618737	-0.514983287	0.646691207	1
PODXL2	2.760836236	0.251762783	1
ATP11B	2.474752171	0.12963671	1
RGS1	0.455235106	0.767698199	1
SLC16A14	0.750351633	0.611900848	1
KHDC3L	0.794347077	0.70222736	1
ANKRD6	-0.580308623	0.669530086	1
HOXB3	0.497610663	0.671007811	1
G28771	0.705126224	0.628781964	1
CALHM2	-0.271024684	0.768523405	1
ENSMMSG00000000149	0.064495251	0.965675991	1
RESF1	-0.27036787	0.781373841	1
SINHCAF	1.486811659	0.362252735	1
TLR2	0.931226334	0.429972158	1
BANK1	0	1	1
HNRNPA3	1.090026302	0.453479255	1
ENSMMSG00000023265	-1.074280357	0.402552659	1
CYGB	-1.08438089	0.420049473	1
FTSJ1	0.798970614	0.611439761	1
PUS7L	-0.084096321	0.952829721	1
XRCC3	-0.132033193	0.897762116	1
IZUMO4	2.490655439	0.094245621	1
PALM2	0.476214897	0.803548669	1
SFT2D3	1.331197066	0.30567992	1
G29157	0.958403273	0.584629517	1
CELA3B	-1.812884121	0.583638113	1
PIKFYVE	0.808057082	0.732262114	1
CPNE8	0.551990965	0.616699262	1
SLC35E3	0.134970859	0.899737151	1
COL22A1	-0.80209178	0.643122788	1
DNAH12	-0.054775243	0.978127419	1
RAPGEF4	0.317797788	0.840683046	1
COLQ	0.491225794	0.767473741	1
TCP11L1	0.189997143	0.858677435	1
NR6A1	1.407899747	0.399726725	1
PXDC1	-0.583122335	0.582112371	1
C7H5ORF63	-0.207874632	0.88495334	1
POLE3	-0.391389104	0.785907656	1
LOC513659	1.048749692	0.549022383	1
ENSMMSG00000010183	0	1	1
UCHL1	0.425521313	0.793940326	1
RNF39	0.840230456	0.595900848	1
CC2D2A	1.095234501	0.52826725	1
DOCK11	-0.774070502	0.585887366	1
CCDC61	2.09934468	0.247547854	1
ABRAXAS1	0.240165245	0.87857039	1
ZNF292	1.080142499	0.512454995	1
G9674	1.696562678	0.305019798	1
SLC6A13	-0.921823886	0.474051554	1
PRSS53	1.529464952	0.285327992	1

CYB561D1	0.959662812	0.486383182	1
ZNF197	0.568283067	0.663354688	1
DCSTAMP	3.972839	0.113484018	1
ZC3H13	0.247704721	0.952131452	1
PBLD	-0.728042253	0.601892571	1
PARD6A	0.039362389	0.978380088	1
GORAB	1.556530399	0.25118455	1
SLC25A16	0.072207866	0.956184851	1
SIX4	-0.63272913	0.658009518	1
ZNF3	0.499103599	0.721595341	1
ENSMMSG00000017909	-0.044035385	0.971626063	1
SCIN	-0.703009575	0.628281929	1
ENSMMSG00000003952	0.253636982	0.871028224	1
RORA	0.249905679	0.826331316	1
G10647	1.015034707	0.426518737	1
SYN2	0.750979048	0.557787182	1
BRWD3	0.531195128	0.730298787	1
LOC100847773	0.761146548	0.502695988	1
PARM1	-0.572117081	0.599659831	1
KDM1A	0.041628605	0.976775841	1
ENSMMSG00000010175	1.230492959	0.432617736	1
FAM219A	-0.513298859	0.693586691	1
ZNF865	0.328017667	0.777013665	1
G24942	-0.302596659	0.825385201	1
CEACAM16	1.889759712	0.35808777	1
G13482	-0.81709566	0.714881828	1
PDE1C	0.051172143	0.964018317	1
ZNF93	0.111816928	0.924907149	1
KLHL6	0.159227838	0.93306958	1
LOC512684	-0.296408687	0.826528897	1
EBF1	0.613925001	0.700526374	1
SYCP3	0.5657866	0.619700314	1
KIF19	0.912888397	0.500563168	1
CACNB2	1.315451812	0.355212639	1
ENSMMSG00000015463	1.954887148	0.131905984	1
CLSPN	0.857472865	0.591955829	1
MEF2A	-0.61798753	0.692764878	1
CEP78	-0.202379465	0.885770224	1
ENSBTAG00000031532	-1.13476956	0.446145353	1
ENSMMSG00000013268	0.390978248	0.809040902	1
ARHGAP28	0.426422543	0.7148885	1
PTGES	0.062623186	0.974813993	1
ENKUR	0.645775401	0.509350745	1
TNFSF10	0.020730591	0.9893923	1
PRKACB	-0.031009469	0.982820508	1
RPL23A	0.592349947	0.749874982	1
FLT4	0.466378006	0.61505043	1
SLC43A1	1.354815068	0.452384866	1
LOC784541	0.891762947	0.544699853	1
TLX2	1.252656824	0.468599571	1
PEX11A	0.530006985	0.614790319	1
H2BU1	0.70815358	0.651177696	1
C3H1ORF109	0.740467632	0.539231717	1
ENSMMSG00000006182	0.312772687	0.843389099	1
MFAP3L	0.349509807	0.787626591	1
MTUS1	0.560274263	0.706014104	1
VKORC1L1	-0.345854016	0.807380842	1
YY1	-0.360144666	0.70744086	1

SMAGP	1.130117073	0.440515087	1
G14425	1.983020476	0.13790421	1
SHB	1.611629021	0.239174603	1
DTWD1	-0.012398472	0.991182119	1
ENSMMSG00000000466	0.825065083	0.481438078	1
RBL1	1.301070072	0.38566039	1
BIRC6	1.157684848	0.617165241	1
ANP32B	0.42117292	0.775395931	1
PCGF3	0.101508646	0.941678339	1
ACTR5	0.129578372	0.926987851	1
SHROOM1	0.106184607	0.930052571	1
FAM20A	0.601507748	0.637311433	1
CHST2	-0.648650222	0.554515527	1
PARVA	0.289635393	0.842333009	1
IL10RA	-0.494076133	0.73441963	1
DYNC2LI1	0.343984051	0.797053806	1
C8H8ORF58	2.000416572	0.132999319	1
SKP2	0.085128631	0.955480178	1
QTRT2	1.247954018	0.362545962	1
SMIM4	-0.110002253	0.911843649	1
ZACN	1.176629044	0.286473936	1
RPS8	1.71740279	0.246414574	1
ATP6V0E2	0.573468414	0.737662223	1
TMEFF1	1.785479624	0.268913211	1
KLHL15	0.243769266	0.871407636	1
GIN1	-0.342882978	0.794011004	1
SLC50A1	0.19995413	0.857369246	1
KAT6B	-0.032095813	0.988873467	1
POC1A	-1.879348588	0.40695765	1
KCNB1	0.041130499	0.978556582	1
RFX2	-0.34062734	0.839152999	1
OLA-N/JSP.1/LOC10012591	-0.634884477	0.670897142	1
SOX6	-0.927654922	0.562527442	1
IKBIP	-0.206178464	0.8768558	1
ITPRIPL2	0.250024506	0.867675261	1
SLC6A1	0.994307563	0.569147046	1
GNA15	1.270314603	0.304530285	1
ZFP57	0.070084311	0.96323318	1
TMEM204	-0.162530992	0.850145099	1
GNPDA2	0.128507616	0.907064291	1
TSPYL5	-0.811603566	0.486910076	1
RBM41	-0.031597626	0.977989348	1
G3107	-0.215761447	0.877550586	1
LOC100137897	0.762975147	0.613853511	1
IFITM1	0.201098653	0.846153823	1
PURG	0.8084742	0.616700096	1
MMP28	-0.137036652	0.891334948	1
FBLN7	0.133178564	0.934303797	1
SLC25A43	-0.216663549	0.881778365	1
FGFR3	0.299078503	0.84728113	1
MEF2A	-0.860819704	0.536869069	1
LMLN	1.490402122	0.390498167	1
G26671	-0.547912953	0.725155981	1
CCL21	0.881075715	0.455388083	1
TSACC	-0.671215862	0.643464154	1
PPARD	-0.197644492	0.894372655	1
NPTXR	0.168721436	0.907002403	1
SOX4	1.624010761	0.220081031	1

DNAJC18	-0.167122602	0.890117529	1
ATP8B4	-0.374937228	0.81550653	1
ENSBTAG00000050172	-0.280876605	0.83134832	1
POU6F1	-0.889169551	0.418589265	1
SNTB2	0.388282697	0.813316701	1
NHSL2	-0.129800425	0.911542705	1
BMP7	0.238182846	0.866573554	1
NKRF	0.027034181	0.980978686	1
CYB5RL	-0.319170573	0.815901579	1
DCLRE1A	-0.026823725	0.985557233	1
MBTD1	0.663224204	0.710232138	1
EFHB	-0.286293235	0.862862333	1
CD164L2	0	1	1
CCDC158	1.249684783	0.52855003	1
WNK4	-1.313291194	0.55639932	1
FADS1	-0.443173859	0.757692653	1
LVRN	-0.093910169	0.948453678	1
EFNB2	-0.158425346	0.914544941	1
CEP152	2.044201248	0.086826412	1
FMNL1	-0.405656587	0.859543738	1
RNASE6	-0.088447577	0.941841513	1
SHE	-0.244528113	0.874041736	1
ZBTB34	-0.515683262	0.736869637	1
TENT5B	1.520520041	0.300279844	1
PSD3	0.584066166	0.75463137	1
MAPK11	-0.179815594	0.836150107	1
ERG28	0	1	1
TNFAIP6	-0.916882817	0.518822184	1
MRPL50	-0.562568646	0.655858239	1
CCDC117	0.677849763	0.621303927	1
GEMIN6	-0.243083035	0.810614509	1
SLC35D1	0.342289731	0.780053658	1
TTC13	1.197883922	0.604871017	1
LBH	-0.407477673	0.762966718	1
FAM102B	-0.588539716	0.684562847	1
WDR54	1.395165695	0.270292501	1
AGT	-0.906275221	0.608806784	1
SLC7A6	-0.816613882	0.531097707	1
GSTO1	2.121402586	0.126273495	1
BCL2L11	2.39131103	0.103114588	1
PLXNC1	-0.322510834	0.813301069	1
PLD1	0.692895466	0.652280092	1
CD83	1.116678837	0.493660533	1
ST3GAL1	-0.394726929	0.778255939	1
CSDC2	3.010265166	0.175833343	1
RASD2	-0.916379134	0.36389262	1
CCDC86	-0.339069958	0.723279075	1
ZNF618	6.369690209	0.148789344	1
GIPC3	0.436914834	0.663079804	1
MBOAT1	-0.439965232	0.756092782	1
ABHD17B	-0.455255019	0.728649782	1
PLSCR4	1.049699264	0.593309708	1
ARHGAP20	-0.214998549	0.886416366	1
MAB21L1	-0.743565708	0.585592118	1
WFDC3	1.178899717	0.427185829	1
PGBD1	-0.166182674	0.902477967	1
RUNDC3A	2.198890949	0.254053613	1
BRD2	-0.081171214	0.946254761	1

PIGA	0.981032474	0.465465328	1
CDSN	0	1	1
G20058	-0.862415981	0.405146155	1
CDK18	2.308272968	0.140238187	1
PSMB9	1.77812181	0.153702261	1
UMAD1	-0.691513963	0.606394964	1
C1H3ORF70	0.557083216	0.641879222	1
CORO7	-0.586986775	0.675908181	1
DMRT2	-1.300031309	0.307186522	1
RWDD1	1.116241464	0.50861037	1
GRAMD1B	2.32259617	0.362954855	1
KIF3C	0.761012539	0.61263664	1
NTRK2	0.94199158	0.6566615	1
ADAMTS3	1.614880477	0.288236524	1
TCAM1	0.388474936	0.683512924	1
C3H1ORF216	-0.271443964	0.863119193	1
HOXA3	-0.758073953	0.593578416	1
NCOA5	-0.718067357	0.688621729	1
HDAC8	-0.093840639	0.949029715	1
NAIF1	-0.18748618	0.890692981	1
LOC526769	-0.151245111	0.88832489	1
RGN	1.130481906	0.400012337	1
VSIG4	0.837932709	0.59219449	1
FAM167B	1.723937184	0.451459027	1
RAD51D	0.6125622	0.559048426	1
FLT3LG	0.783756466	0.528524188	1
SLC16A7	0.658342431	0.699543728	1
VAV1	0.482049571	0.637110317	1
ZNF672	-0.912086146	0.495467798	1
GUCA1B	-1.814082577	0.564487657	1
CNNM2	0.507987405	0.759263716	1
LDB2	-0.7716544	0.564150857	1
ARSK	0.552611161	0.699486028	1
CHST3	-0.627892447	0.658188418	1
DIPK1B	-0.270304055	0.759359157	1
GDF11	-0.112841756	0.929010709	1
VEGFD	-0.445537228	0.750692411	1
FZD5	0.844002556	0.627777519	1
ZNF704	-1.219346576	0.508657049	1
NOD2	1.231926734	0.421815478	1
G37665	-0.381368733	0.751545776	1
CFP	0.877464	0.47506053	1
REM2	-0.144635664	0.88751608	1
TFR2	1.687703181	0.095055981	1
BMF	1.436745827	0.343257082	1
MORN1	0.056542283	0.953845203	1
ENSBTAG00000038916	0.378140552	0.715872894	1
CD302	0.373580317	0.812097396	1
USP35	0.461217664	0.721268943	1
OLFML2B	0.190795203	0.913503873	1
EGFL8	1.100507341	0.254596752	1
ENSMMSG00000000115	1.102273424	0.411986943	1
GATA2	-0.868920313	0.463216673	1
FOXO6	-1.269071515	0.309844743	1
KIF23	1.569271834	0.247505674	1
NMRK1	1.437407027	0.263352362	1
C3H1ORF56	0.75309138	0.468111723	1
MLKL	-0.01046254	0.990817725	1

TM4SF18	1.297882115	0.25558946	1
CPLANE2	1.521610789	0.291767149	1
KLHL13	-0.137290058	0.927145028	1
ITGA2	0.730556352	0.679444296	1
CD48	-0.369093156	0.81942176	1
ABCC2	0.791950665	0.587561844	1
AGO3	0.144627035	0.898520599	1
AGO3	1.260010617	0.484046292	1
IRAK4	0.511349912	0.616169585	1
MYO9A	0.959825479	0.573909582	1
RAB3IL1	-0.968257334	0.451874705	1
CDKL5	0.817901422	0.723687243	1
CDH24	1.219191527	0.438803959	1
ENSMMSG00000001565	0.012262398	0.990584601	1
KCNH6	-0.4369672	0.674763051	1
SYTL2	-0.577391912	0.654590757	1
BCL10	-0.099536808	0.916978289	1
FOLR3	1.08975269	0.64775414	1
MAN2A1	0.579502426	0.616760452	1
GSAP	-0.077124256	0.957437256	1
CSPP1	0.156792879	0.915806319	1
RRAS	0.173007805	0.883717771	1
DDC	-0.417753072	0.803414209	1
LDAH	2.081924185	0.422578265	1
LRRC4C	0.338892579	0.764670204	1
MT3	0.153093976	0.892018041	1
ENSBTAG00000013334	0.540076468	0.677503915	1
ISOC1	0.658017277	0.626499196	1
G30219	0.986761884	0.542111075	1
PPP1R14C	-0.696944876	0.580723781	1
LOC781710	1.581303277	0.222423849	1
ADAMTS20	0.282981093	0.858982996	1
MGAT5	0.125747739	0.905518986	1
ENSMMSG00000007393	1.792524066	0.315935078	1
AOC3	1.612438	0.494966958	1
ZSWIM9	-0.697462672	0.444211916	1
PCSK5	-0.014668674	0.989144251	1
SHB	-0.4913892	0.673476017	1
SYT11	-0.253668249	0.86888933	1
AOC3	-0.913447474	0.497510901	1
IL16	-1.476648109	0.464132244	1
CYBRD1	0.091480485	0.948285751	1
IFT81	0.521723002	0.750418394	1
KIF20A	1.2723755	0.518351755	1
DUSP19	0.219636287	0.821923296	1
ARHGEF9	1.180186679	0.321350532	1
CMTM7	0.777164594	0.591805266	1
ENSMMSG00000023325	0.602275276	0.580054364	1
RMI2	1.058525115	0.48421758	1
MAP3K9	1.455066354	0.266721466	1
ENSMMSG00000020014	0.228736838	0.872013006	1
CHST14	0.956224739	0.399706258	1
IQCD	0.093123238	0.938964309	1
CFAP45	3.425945559	0.090123957	1
SLC4A11	1.753687032	0.137258187	1
ZNF317	0.85410681	0.540449661	1
KIAA1109	1.492792274	0.451184256	1
TTPA	2.424373592	0.10913732	1

ACOT11	0.097285918	0.953084758	1
PPM1H	1.490535694	0.331683296	1
ATF6	0.767557675	0.479858434	1
TCOF1	2.268169663	0.586245498	1
ENSMMSG00000023998	-0.183081268	0.860641708	1
ENSBTAG00000031242	0.625455104	0.526316967	1
CDK20	0.031319108	0.983879559	1
TMEM145	-0.89145328	0.464963701	1
SMOX	0.606552342	0.61254222	1
NLRP2	-2.849380736	0.149333933	1
NTRK3	0.28086837	0.904579425	1
LRRC8C	-0.767769672	0.473322595	1
RPL12	0.64310563	0.653715381	1
LRIG2	4.018262836	0.113624551	1
SYN1	-0.442493284	0.644016416	1
ZNF674	0.445392106	0.695861832	1
CRYM	1.872630054	0.232181379	1
BHMT	-1.898624453	0.462505831	1
RAB19	2.626958191	0.186022998	1
FHOD3	-0.412764657	0.776222918	1
ARL4D	1.748332668	0.145042518	1
PDSS1	-0.908760854	0.484437009	1
NAP1L4	-0.665173473	0.689139349	1
SPOCK2	0.471125558	0.639290862	1
CMTM3	-0.472535343	0.745247892	1
ACSL6	0.508605651	0.769123948	1
SMYD5	0.975659851	0.571629096	1
WNT5A	0.47967879	0.595743221	1
GEN1	1.372897763	0.344449687	1
HCFC2	-0.494262748	0.73419459	1
BACH2	-0.619147299	0.640277607	1
PGBD5	0.697491079	0.672301202	1
TBRG1	-0.378962668	0.787943191	1
USF3	0.154474847	0.884666564	1
HECTD2	-0.83737464	0.537174986	1
ADAMTSL3	-1.054888313	0.329534668	1
FRS3	0.049542482	0.966638969	1
LAT2	0.122988446	0.931165168	1
SDK1	0.936793983	0.454541561	1
SNCAIP	-1.055593881	0.405225805	1
REM1	-0.618501745	0.576770651	1
CPS1	2.274624719	0.245160444	1
VSIG8	3.11464806	0.093156946	1
RNLS	-0.385247416	0.685539358	1
FBN3	-0.302950965	0.860869988	1
INVS	0.121813708	0.912523009	1
ZSCAN20	0.635020901	0.611253519	1
KCTD7	0.311071511	0.845321199	1
ITGA8	-0.100802054	0.913479878	1
CMSS1	1.520637384	0.294204103	1
PPIL6	1.843195791	0.294884739	1
ENSBTAG00000052265	2.025432915	0.235157259	1
ENSMMSG00000002250	0.681159355	0.544078791	1
ZNF655	-0.214430023	0.895714232	1
ZNF516	0.269649832	0.804380784	1
PHLDB1	-0.815239971	0.538513348	1
FAM110D	0.183319234	0.872730752	1
HES6	1.162690111	0.463635182	1

ODR4	0.384222118	0.713625747	1
PHLDB1	-0.858396355	0.480572339	1
PRKAR2B	-0.095019373	0.947259958	1
F8A1	0.228295326	0.857546436	1
LOC615706	-0.118701319	0.915236314	1
CTSW	-0.039386206	0.97410614	1
SERHL2	-0.029198672	0.977568112	1
IRAG1	0	1	1
PHF6	0.947795669	0.429985858	1
GOLT1B	0.452244789	0.757007826	1
DND1	0.180606753	0.870879619	1
SLC25A5	0	1	1
TMPRSS4	4.445305939	0.098976887	1
DAR1	0.912983931	0.515474872	1
RPS6KL1	0.264925339	0.847002855	1
C18H16ORF46	0.809721998	0.486412749	1
SEMA5A	0.403210605	0.779312312	1
RNF130	-0.328582822	0.816143519	1
EFHD1	-0.718678553	0.606201118	1
ENSMMSG00000010726	-0.369889018	0.681331705	1
PCSK5	-0.284531905	0.845830666	1
ASB9	0.203870219	0.898640351	1
MMP20	0	1	1
CCDC88A	0.247394266	0.897553371	1
ARHGEF10	-1.065503459	0.634220081	1
ASTN1	0	1	1
F2	0.89941577	0.471801676	1
RBP4	-0.740292356	0.509062201	1
SMOC2	0.271588281	0.776996379	1
NEMP1	0.452017463	0.748681491	1
FSTL3	-0.154773125	0.904804005	1
ABI2	1.306830291	0.346988635	1
MTHFSD	1.289926004	0.335170281	1
RTN2	-1.131330507	0.381082025	1
KIAA1958	-0.314311439	0.816546201	1
EPC1	-0.351280893	0.742528407	1
FGF10	0.876477563	0.561561314	1
EXD2	-0.837323384	0.540314167	1
LSMEM2	-0.177251692	0.893754695	1
COL4A5	1.840874997	0.261109117	1
COL8A2	2.637709067	0.148025988	1
RGS9	0.419550993	0.742216189	1
ANGPT1	-0.688528301	0.597143288	1
USP54	-0.040638726	0.985936938	1
SMYD3	0.660881035	0.540357865	1
ZC2HC1A	-0.026744738	0.985359245	1
DPYSL3	0.584111962	0.739438269	1
JAKMIP1	0	1	1
MGARP	-0.566661812	0.659195981	1
OTULINL	0.599997921	0.70559538	1
ALDOA	-0.361556446	0.70358336	1
ATP11C	0.138815888	0.927414805	1
CSN2	0	1	1
RARRES1	0.803624261	0.500522238	1
PXK	-0.217114903	0.836041713	1
ENSBTAG00000049866	0.674840079	0.653527641	1
IFT80	-0.388912344	0.788082297	1
SEL1L3	-0.534004452	0.687514999	1

SLC30A4	-0.471172481	0.6517801	1
RPGR	-0.107447363	0.937287774	1
NXPE4	3.069430318	0.107810774	1
DSEL	1.570036973	0.274675341	1
PDE4D	-0.20447584	0.910083231	1
MESD	1.181926835	0.438476984	1
BRD1	-0.720231393	0.450132398	1
MXI1	-0.44209804	0.772982736	1
G17204	1.806966133	0.224381431	1
AKAP7	-0.426452063	0.782992966	1
ITGAL	2.448560889	0.242555469	1
LTF	1.514047735	0.380770925	1
CPNE8	0.620372209	0.670160155	1
ZNF133	0.248513456	0.854717812	1
KLHL42	0.413628438	0.655490282	1
ZNF467	2.512346187	0.227846991	1
COTL1	-0.184849627	0.902657985	1
LOC507756	-0.555608033	0.604246852	1
SAP30	0.328148099	0.781226627	1
EDN1	0.626649201	0.688184621	1
PRR19	0	1	1
BST1	0.027798295	0.985048881	1
ENSMMSG00000024298	0.205227858	0.889310672	1
PABIR2	0.786744511	0.486687362	1
CLIC2	-0.79931334	0.470683673	1
PLK4	0.800538708	0.511755315	1
SELP	0.515326309	0.728409644	1
ZNF331	0.32350227	0.833061832	1
MYD88	-0.50835002	0.717238558	1
PIK3C2G	2.957608267	0.16379312	1
FANCF	0.109581162	0.920577031	1
SLC5A8	0	1	1
RASGEF1A	0.345470604	0.704478523	1
RAB29	0.463091323	0.761110167	1
TSTD3	0.278830566	0.836750944	1
CA9	2.036261118	0.104431107	1
CYB5D1	0.300120121	0.834000747	1
CPT1C	0.850872713	0.472149212	1
ZBTB46	-0.867748907	0.489096157	1
GLCE	1.1678794	0.384873857	1
CENPO	1.22852014	0.359485982	1
HOXA10	-0.015832878	0.990709144	1
ZNF207	-0.114703749	0.931583034	1
NIN	0	1	1
RAB6B	0.539881052	0.712620305	1
ENSMMSG00000014728	-0.158391799	0.881126853	1
PTPN13	6.225861516	0.150733589	1
MT1A	-0.798568705	0.565610885	1
RNLS	-0.547026244	0.696735695	1
ATP10D	-0.292891328	0.765820765	1
ZBTB2	0.09209849	0.947791506	1
MPHOSPH6	0.598914518	0.575983649	1
LCP2	-0.383141995	0.800245555	1
CPEB1	2.905060252	0.110751484	1
DOCK2	0.340575026	0.813799771	1
ESRRB	0.320307217	0.84135458	1
TUSC1	-0.419995802	0.794510048	1
ZNF775	-0.259993425	0.833052017	1

ENSMMSG00000014148	-0.326775109	0.794047305	1
UBE2T	-0.099627505	0.90963103	1
EPHA4	0.825946735	0.455873713	1
ENSBTAG00000026626	0	1	1
CD37	0.356063157	0.760139707	1
ITIH4	0.709797348	0.769224669	1
ENSMMSG00000009292	0.07886053	0.933756352	1
MBD5	0.117196544	0.925010346	1
TMEM186	0.285580998	0.853245801	1
FZD3	1.691151949	0.383140733	1
G9024	-0.109758799	0.93755713	1
ENSMMSG00000019952	0.647109177	0.559610949	1
PTCH2	-0.430125436	0.674017764	1
HSF2BP	-0.426047781	0.750352352	1
MSI2	-0.277474279	0.845984372	1
NTRK1	0	1	1
PCNX4	0.384678694	0.816420884	1
CREB1	2.095501672	0.25515903	1
LOC100124497	0.17122427	0.914388678	1
KCNQ5	0.153271429	0.92136556	1
FAM50A	-0.978428339	0.326049693	1
BEND5	2.093632837	0.158215013	1
TCF7L2	0.907383531	0.569057084	1
MYZAP	-0.457280434	0.664052939	1
ANGPTL7	-0.259065814	0.846744404	1
GORAB	1.272730568	0.451094021	1
LRRC75A	1.262989063	0.307772295	1
HSPA12A	-0.13430269	0.942365357	1
ANKRD44	-0.752821307	0.580394963	1
IFI16	-0.537517199	0.635114225	1
HOXD9	-0.91516184	0.406325494	1
ZNF688	-0.30178173	0.801432828	1
SLC19A1	1.198747276	0.227057683	1
PCDHGA8	1.952935787	0.297636052	1
CLP1	-0.365716304	0.808127095	1
GCH1	0.252866583	0.837397014	1
G35670	1.289460414	0.358398197	1
HIF3A	-0.132259189	0.946075064	1
ASAP2	-0.160358305	0.909416377	1
KIF5A	0.741389884	0.640767079	1
SLC39A10	0.840930656	0.610053986	1
MYF5	-0.065283747	0.965144246	1
FOXD4	-1.82438458	0.445671231	1
BICC1	0.063570149	0.973046695	1
FBXO17	0.347709804	0.812862876	1
TMEM220	0.90239504	0.387364774	1
ENSMMSG00000014485	1.234616125	0.468979882	1
PDGFD	0.74373267	0.607693124	1
PRKN	0.045712656	0.977724798	1
KCNIP2	-0.749344246	0.627336732	1
DCTD	1.8217693	0.289475365	1
HIC2	0.405332471	0.722678901	1
CKS1B	-0.5433935	0.617221238	1
ASTE1	-0.377291232	0.739314534	1
DPP4	0.440116514	0.764769435	1
RORC	1.768527916	0.228829966	1
SDCBP2	1.363385531	0.535363545	1
B4GALT6	0.742233446	0.603226861	1

FICD	-0.077178251	0.957961074	1
NCOA2	0.397056122	0.784432258	1
ATG4C	1.549028004	0.369723379	1
CTH	1.122925553	0.467307068	1
SYT12	0.224149741	0.880165831	1
SCAI	0.27973904	0.816593255	1
CFAP44	0.442347905	0.807984196	1
SLC1A3	0.419809186	0.796166139	1
NRROS	-0.769040904	0.475759723	1
PLCB1	0.720041767	0.703139381	1
CASD1	-0.270854074	0.85222728	1
CD55	-0.300993937	0.75940572	1
PEG3	0.873843709	0.463303321	1
YIPF1	-0.213199455	0.872032084	1
METTL5	0.362038142	0.754339286	1
OVGP1	-0.092198549	0.951711242	1
MMS22L	1.169446333	0.414982047	1
ARSK	0.383648271	0.788550834	1
NAA40	-0.58858637	0.579550801	1
ENSBTAG00000019931	0.439105347	0.837164774	1
BIRC3	-0.379363916	0.677327898	1
HMCN1	-0.488991674	0.735628698	1
CCDC88B	1.861557546	0.127980511	1
SDHAF3	0.801503289	0.515522264	1
SEMA3E	3.397089464	0.164215899	1
ENSBTAG00000008836	-0.526785062	0.714093338	1
IL4I1	2.017861344	0.310225162	1
IMMP2L	0.107368881	0.942983171	1
LOC100848700	0	1	1
TBCK	0.009254314	0.995128289	1
PDZD3	0.973367572	0.379245918	1
PLXNA4	-0.135984298	0.935699983	1
MORN2	-0.60065838	0.674833208	1
ERCC6L2	0.400673154	0.781257633	1
ROBO1	0.785069395	0.52015024	1
RPS20	2.463367741	0.123803416	1
ENSMMSG00000024193	0.711755323	0.594648821	1
LOC616254	-0.880930802	0.322224442	1
GLRX2	0.647204293	0.492566238	1
DUSP5	0.878214522	0.389636533	1
CCDC15	-0.052612503	0.972973766	1
IFNAR2	-0.612308734	0.667403522	1
WNT4	0.732634638	0.679625324	1
PSMC3IP	-0.394628261	0.683138611	1
CLDN17	0	1	1
ENSMMSG00000015650	-0.239949387	0.860613835	1
ANKRD44	-0.347908045	0.72771671	1
DNASE1L3	0.272909255	0.79316891	1
IKZF4	-0.834302871	0.537272521	1
TUBA3	0.213848504	0.91298385	1
PLK1	0.430408381	0.654141415	1
FBXO36	3.662323248	0.121704651	1
FAM172A	1.426779298	0.393301665	1
ESPN	1.490178306	0.306405131	1
MEOX1	0.334136931	0.833630602	1
GPR161	1.223165122	0.227672905	1
MRAS	-0.285195833	0.801228403	1
GATC	-1.008111996	0.439803702	1

C11H9ORF50	-1.310755338	0.515813046	1
KIRREL1	-0.436821266	0.753981992	1
DZANK1	1.432665263	0.246835988	1
ENSBTAG00000013871	-2.850237985	0.168040776	1
BCAS4	1.399400659	0.364620252	1
C18H19ORF81	0	1	1
ZCCHC9	-0.580397976	0.617843074	1
C22H3ORF14	0.194181757	0.849538475	1
SLC26A1	2.005042359	0.089944155	1
RASSF8	-0.478328667	0.675222342	1
MED9	-0.411363466	0.78261318	1
ENSBTAG00000049815	3.105568977	0.11632356	1
NUP210L	1.48874005	0.389300007	1
ABCG4	0.054462021	0.970237205	1
MGC134040	0.165732254	0.906583635	1
HAUS2	0.641301298	0.691486617	1
DNAAF4	0.035342837	0.981668729	1
BASP1	1.296610785	0.352846374	1
MMP9	-0.328517373	0.845787655	1
HYKK	1.233692433	0.379664012	1
G23488	0.111578203	0.920408357	1
TMEM59	0.460418006	0.671864813	1
ZNF397	0.353463972	0.821202678	1
AURKA	-0.467722067	0.733774507	1
VSTM5	3.008050071	0.153603985	1
WNT9A	-0.620247627	0.686407307	1
ARV1	2.200593561	0.196437702	1
RTKN	0.342470618	0.756212867	1
NKD2	2.240590141	0.093641607	1
CLIP4	-0.51838792	0.753125154	1
SYBU	0.081650679	0.954522841	1
ZNF618	1.355390434	0.277624167	1
RAD51AP1	-0.483979849	0.739194722	1
SPTBN4	-0.332304799	0.753013337	1
ATF2	0.221066239	0.880562974	1
GET1	-0.765761189	0.581411766	1
DENND5B	-0.839082352	0.48214379	1
ENSBTAG00000040603	0.152471465	0.892500519	1
CSF2RB	0.061477481	0.951229477	1
SMAD6	0.2367955	0.796265437	1
COPG2	0.615073879	0.693689024	1
TBX1	-0.579335651	0.694557555	1
LMTK3	0.81846095	0.51127912	1
H2AW	0.983546541	0.56543583	1
ZCCHC4	-0.08821837	0.952985194	1
SLC25A41	0.813429008	0.476877885	1
FGR	-0.636862809	0.663791879	1
ABI3	-0.747748448	0.462531978	1
LOX	-0.720690285	0.582810745	1
COX4I2	0.671228846	0.53537892	1
C16ORF72	-0.474436555	0.672048352	1
INTS9	0.199464351	0.894430175	1
COMP	-1.337210586	0.340520013	1
ENSBTAG00000052906	0.341943485	0.805763306	1
ST6GALNAC2	1.738079054	0.213150332	1
ERICH2	0	1	1
SFRP1	1.153967749	0.392071191	1
NUDT7	0.246223462	0.863375644	1

HDAC8	-0.474778773	0.582582928	1
OLFM2	2.56207887	0.196596621	1
PPP1R18	-0.65103821	0.642872388	1
ENSMMSG00000017998	-0.253310703	0.799637029	1
G13899	0.842914113	0.613790022	1
RNF122	-1.284903079	0.315301752	1
ENTPD1	0.816195965	0.408539509	1
BIRC5	2.888276459	0.296162781	1
PDP2	1.061382188	0.526456003	1
TSPEAR	-0.388346045	0.796303453	1
PDZK1	1.963641422	0.187929414	1
SLC16A10	-0.722045069	0.551265452	1
GK	-0.411596159	0.821152881	1
GLI2	0.272193612	0.842490161	1
CCL2	0.216660219	0.892808968	1
ANG/RNASE4	1.356984998	0.25154124	1
DACT1	-0.176949403	0.913069806	1
PRDM5	-0.258643559	0.849822483	1
FBXL13	0	1	1
DOC2G	-0.285304814	0.84688799	1
ZMYND12	0.005893719	0.996605184	1
ZNF710	0.08538377	0.938345834	1
RNF6	-0.245430669	0.803906272	1
BANP	-0.402052795	0.705632109	1
ETNK2	2.851406827	0.098563944	1
LOC512464	2.360932134	0.26939326	1
AQP4	-0.262355946	0.856649031	1
FAM219A	-0.290868164	0.836665193	1
TCF12	0.180349392	0.907112046	1
GMFB	0.951279541	0.555952558	1
ZDHHC14	1.318987555	0.356088219	1
FAM172A	1.274940406	0.517921435	1
ERG	-0.87381526	0.535884115	1
ZNF774	-0.152554384	0.912973945	1
CLEC1A	-0.562755645	0.578617624	1
POLG2	0.826388449	0.496197177	1
LONRF1	-0.116588711	0.935801909	1
SNRPD3	0	1	1
HEATR4	2.234018108	0.095871316	1
BORCS5	2.074983624	0.151670561	1
NHEJ1	-0.308206472	0.816524342	1
ATP2B3	2.989871974	0.204761697	1
BEND7	-0.718313125	0.595649558	1
RELL2	1.601959056	0.182634262	1
KCNH2	-0.34731482	0.839906893	1
MAD1L1	1.02253436	0.39926358	1
CCDC14	-0.136560296	0.886421505	1
FAAP24	1.979575484	0.139328885	1
TULP2	-0.032100366	0.973574606	1
TNFRSF6B	1.503295281	0.205878555	1
SHROOM2	-1.370617273	0.732663927	1
ENSMMSG00000021023	0.798134083	0.488900593	1
ESYT2	0.778025186	0.62021454	1
PRIMPOL	1.370780772	0.295338649	1
ENSMMSG00000019083	1.423306762	0.307839388	1
OPA3	-0.390287445	0.807794641	1
SSX2IP	-0.124327059	0.929575064	1
SMURF1	-0.066103574	0.964636346	1

ZNF554	0.010074287	0.991905517	1
UPF3A	0.360590651	0.763422682	1
MRE11	1.734930509	0.274438714	1
RASGRP4	0.018273177	0.986300499	1
CACNA1A	-0.031357376	0.975135748	1
METTL21A	-0.091098812	0.954101562	1
TNFSF13	0.235512747	0.769258384	1
SYK	1.401722306	0.206742805	1
WIPF1	-0.768058738	0.581654821	1
NAPB	0.318171871	0.781714828	1
NSUN3	-0.321448621	0.827065253	1
CDCA7L	1.687070602	0.193013265	1
LOC101906939	1.104691323	0.374073047	1
FGFR4	-0.177201595	0.845521156	1
HMMR	-0.401058946	0.795160191	1
ENSMMSG00000018226	-0.307028048	0.775565681	1
ARHGAP26	0.29501966	0.839436242	1
PWWP2A	1.046758846	0.401146986	1
ZNF274	1.647628619	0.208477168	1
FUT8	-0.240056225	0.867955488	1
ADGRG6	0	1	1
LOC785216	1.284283478	0.228006654	1
ASB12	0.171070054	0.907699228	1
GMFG	1.099707322	0.403665021	1
ZNF384	-0.442827623	0.7626348	1
ENSBTAG00000036222	0.738135341	0.639784629	1
TRIM47	1.41770259	0.379230994	1
SLC38A4	-0.016539138	0.991285669	1
EXOC4	-0.383577777	0.804002825	1
PIMREG	0	1	1
ZNF385A	1.050853562	0.448042293	1
788175/LOC782367/LOC51	-0.593139959	0.551212986	1
RASA2	0.877777122	0.594035157	1
TMEM170A	0.329084671	0.837657177	1
G20142	1.081604084	0.384942717	1
ADGRV1	0.558023907	0.684240753	1
FER1L6	-0.345486761	0.65670552	1
ENSMMSG00000019685	0.646667539	0.690265496	1
PACRGL	2.364572264	0.283537759	1
INPP4A	-0.106133095	0.93630475	1
KCNK6	1.720526233	0.366687906	1
METTL8	-0.13861053	0.91850539	1
LOC507581	-0.387646179	0.777109854	1
SPEF1	-0.961475184	0.321771958	1
CXCL12	-0.125261933	0.944063919	1
USPL1	0.090747713	0.953641254	1
MOK	1.604007272	0.20790977	1
ARHGEF39	0.5884578	0.592278455	1
SNAPC5	-0.00017684	0.99989697	1
LOC100296324	1.042130999	0.451816487	1
CDC14B	1.26888855	0.419047224	1
ENSMMSG00000009755	2.8890203	0.496180807	1
NCOA6	0.314557702	0.900448527	1
ZNF286A	0.291860686	0.805831717	1
ADAMTS15	-0.508933525	0.720100792	1
LOC787904	0.286214226	0.841591532	1
DDHD2	-1.396044422	0.480473962	1
MFRP	-0.587253931	0.577703194	1

TRIM66	1.219095865	0.287826408	1
RABL6	-0.076149885	0.946020627	1
RBM33	-0.634160776	0.722536337	1
TEFM	0.132595408	0.915559659	1
RIC3	1.522120514	0.282429837	1
EFNA2	1.444204143	0.187033222	1
OGG1	0.1375314	0.894864272	1
SRMS	1.116358676	0.371485909	1
RASGRF2	-0.640198241	0.739048557	1
MESP2	0	1	1
VIPR2	0.06645257	0.968051472	1
CNNM2	-0.151278781	0.924524597	1
STT3B	0.699207095	0.491825363	1
ADGRF4	0	1	1
SOX13	0.350379192	0.804271147	1
SPDL1	-0.49726171	0.722803211	1
TMEM234	-0.470650968	0.737248979	1
PMS1	-0.836588139	0.40014485	1
SLC30A1	0.254615759	0.839995311	1
CNOT4	0.000565368	0.999725261	1
MROH2A	0	1	1
FCGR2B	1.779695154	0.2271194	1
BRSK2	1.244554129	0.576338834	1
ENSMMSG00000012653	-0.710514574	0.577759529	1
RNASEH2B	0.144925284	0.889335536	1
DACT3	-0.10258706	0.92314501	1
NEK11	0.358673881	0.783705331	1
E2F7	-0.502026556	0.825051876	1
RASGEF1B	-0.226591801	0.816853932	1
TXNDC16	0.625910427	0.593948717	1
FBXO45	-0.621332157	0.676626786	1
PLAUR	0.482254683	0.703750327	1
AREL1	0.229916778	0.880311975	1
TICAM2	1.130639389	0.505612871	1
CCDC102A	0.236621858	0.785772566	1
PTN	0.934285592	0.378134188	1
CCDC167	0.088377781	0.925860917	1
GRAMD1B	-0.206386047	0.873273282	1
STX2	-0.613150863	0.642341264	1
DUOX1	2.200852551	0.193774628	1
TROAP	1.367452741	0.380067968	1
ENSMMSG00000011451	-0.372177921	0.760753217	1
GEMIN7	0.138458964	0.86118226	1
ANKRD34A	-0.265639788	0.813088402	1
SLC35B3	0.468360087	0.739231905	1
GDI2	0.129811906	0.892795558	1
NECAB2/OSGIN1	-0.999972647	0.4541449	1
PRAF2	-0.448654967	0.61418343	1
ENSMMSG00000021344	1.325870695	0.375275785	1
VMO1	1.980634355	0.164681976	1
DIPK2A	1.15090325	0.46262513	1
TMEM59L	2.199509872	0.258156217	1
ZNF366	-0.773309418	0.557351109	1
GLIS2	-0.591513711	0.493479158	1
DEF8	0.780827996	0.650536545	1
PYM1	0.841917382	0.484105923	1
RHOJ	-0.572889059	0.68026503	1
APBB1	-0.509344178	0.700269592	1

BAK1	0.168066607	0.877863292	1
HECW2	-0.04206573	0.97640425	1
ZNF10	0.130498924	0.910432232	1
TCF7L2	0.011388463	0.993906081	1
PGAP2	0.360577561	0.816435498	1
GABRE	0.059632538	0.942716061	1
OXLD1	-0.017429392	0.986855635	1
ATP1B2	-0.570919455	0.577105763	1
MARCKSL1	0.314980733	0.769355182	1
PRXL2A	1.674682408	0.238488036	1
LYVE1	-0.789838514	0.555224075	1
ENSMMSG00000023136	3.074431122	0.093702529	1
ADGRA1	0.35932381	0.81624246	1
SETD9	-0.213356824	0.888459984	1
ENSMMSG00000001649	-1.164494785	0.488871577	1
G36592	0.842877988	0.586487007	1
C2H2ORF76	1.4504871	0.228023344	1
FAM126B	2.39382647	0.226432029	1
UTP23	-0.602464334	0.544604748	1
TSSK4	0.515127232	0.638782234	1
GNB4	-0.566418742	0.683013564	1
HPCAL1	-0.491686686	0.744025417	1
CPXM2	-0.204303426	0.875840647	1
S100Z	0	1	1
BLOC1S3	1.664317099	0.255112169	1
INKA1	1.509395901	0.232392103	1
OLFML1	-0.289571955	0.771697414	1
ENSMMSG00000024456	0.976933559	0.371236253	1
NCKAP1L	-0.09032097	0.927369636	1
PROC	-0.126773231	0.899005799	1
INPP4A	-0.699251399	0.655978611	1
ANKRD26	0.182301442	0.866330101	1
G23255	1.8650983	0.094909462	1
UBTD2	0.809437195	0.442813264	1
DCAF10	-0.481796085	0.630765311	1
PLEKHH2	0.778287152	0.529759572	1
SOX9	0.451804603	0.709940749	1
SLC7A7	-0.45732465	0.603180887	1
SEC1	0.306234771	0.828519867	1
TMEM67	0.414218645	0.76662527	1
RAB33A	-2.853965294	0.309090918	1
TAF5	0.27105334	0.799057146	1
TBCCD1	-0.050092106	0.971486645	1
GNB5	-0.400014151	0.782019162	1
NPHP4	0.618190887	0.507489333	1
RPL7	1.062625895	0.349703718	1
MCUB	1.314132562	0.303583446	1
LOC525426	2.251965774	0.117612046	1
KIRREL1	1.173440831	0.525292153	1
PHF8	0.720829427	0.723770671	1
LYPD2	-3.858500371	0.368727355	1
MARK1	0.479321084	0.764960486	1
ZXDB	1.046146974	0.456018417	1
SERPINB9	-0.83106282	0.514283279	1
TBPL1	-0.218696468	0.881362197	1
PLXDC2	0.915295451	0.514569902	1
ZNF280D	-0.215729126	0.889059001	1
KCNS3	0.188826414	0.900976464	1

PLEKHA6	0.80015602	0.715857493	1
TCF3	0.548266489	0.63376193	1
RELN	0.257886626	0.853770935	1
LRRC58	0.080853857	0.940147934	1
CEP44	-0.13355717	0.895013229	1
MARK1	0.304477532	0.879553652	1
DEXI	-0.135056597	0.899664037	1
RPL11	-0.112988001	0.908954813	1
MYPOP	-0.626182287	0.525092927	1
STC2	1.774322504	0.089067791	1
CCDC150	0.062078739	0.966231704	1
ADORA1	-0.087638148	0.946856502	1
ENSMMSG00000012404	0.815304145	0.565869031	1
GTSE1	0.314719076	0.71515032	1
MINDY3	0.830477193	0.561744733	1
TUBE1	1.33779708	0.430678017	1
FUT10	0.345945747	0.826448131	1
DHODH	0.144669869	0.910443889	1
CBLL1	0.109038029	0.946214403	1
G10141	0.318302444	0.827676132	1
ENSMMSG00000008245	-1.040144631	0.453004026	1
SUV39H1	1.111600817	0.500560362	1
LURAP1L	0.783011307	0.614170797	1
FANCC	-0.416602233	0.717083167	1
TUBA1C	1.348118504	0.323926624	1
TCHH	4.002207502	0.140176012	1
MXRA7	-0.805489738	0.554933858	1
ZDHHC8	-0.432469205	0.783543967	1
WWOX	2.45442539	0.081983136	1
ENSMMSG00000017172	0.229201075	0.80913488	1
SEC22C	0.15242189	0.92142121	1
ZNF746	-0.405489582	0.832359444	1
DMAC2L	-0.822353389	0.370071271	1
CORO2B	-0.828228639	0.549316327	1
ZNF2	0.039059029	0.977998895	1
FBXW9	1.098491121	0.304816633	1
CHST6	2.11317566	0.225349854	1
SLCO3A1	-0.663522707	0.526437556	1
LAMA3	0.072256025	0.953061937	1
JAK2	0.803061682	0.628732633	1
MAD2L1	0.311011202	0.829889365	1
TAF9B	-0.237586106	0.866381544	1
LYN	-0.911994733	0.514764293	1
RFC5	-0.239412318	0.772100177	1
FEZ1	0.160591605	0.881681465	1
MEF2A	0.241400682	0.870334797	1
ENSMMSG00000014637	0.410255668	0.815157736	1
ZNF18	0.982168103	0.477916522	1
TNFRSF25	0.161509398	0.867783046	1
INPP5D	0.514059194	0.716857323	1
FBXO24	0.653700752	0.564194757	1
CCDC77	1.05101704	0.425058375	1
API5	-0.134511802	0.92020856	1
KIAA2026	-0.115261358	0.920491869	1
APH1B	0.91596415	0.553271104	1
ECE2	1.02719249	0.299308802	1
ATP1A3	0	1	1
GRIN3B	1.074536988	0.404361319	1

SOCS3	-1.012956636	0.464540549	1
TMEM150A	-0.287976933	0.855288386	1
ZNF227	1.3769281	0.422235866	1
CLUAP1	-0.18455496	0.917146434	1
TMCC1	0.56121819	0.72829555	1
CARD19	-0.792539972	0.424361571	1
RNF111	-0.123998882	0.90084335	1
IL16	-0.775506914	0.602751011	1
ENSMMSG00000017844	-0.670158072	0.556852079	1
AOPEP	1.249664535	0.339503242	1
SERGEF	0.599751728	0.608866651	1
PLEKHB2	-0.764823701	0.491243855	1
MLXIPL	-0.232064511	0.826586799	1
ENSMMSG00000009630	-0.847129314	0.710103983	1
CNRIP1	-0.763080741	0.583855575	1
AOC1	-1.16558279	0.236108158	1
IL10	0	1	1
BRME1	0.840279211	0.356753823	1
F5	-0.465351677	0.755933637	1
CRIM1	0.662536778	0.580756327	1
NPNT	-0.210231122	0.866824091	1
CBLN4	-0.42768191	0.767218957	1
DSN1	0.366657666	0.796253453	1
VWA3B	1.567271334	0.375178391	1
ME3	1.2930523	0.414946677	1
ISCA2	-0.290386512	0.84393057	1
SMOC2	2.065167738	0.125659346	1
SLC16A11	2.699767174	0.133312181	1
ENSBTAG00000053991	0.933516794	0.826257582	1
MSL2	-0.359344537	0.749149816	1
CHSY1	0.000440522	0.99973942	1
EVI5L	1.311185826	0.436978788	1
TTYH2	0.015104965	0.989193443	1
AEN	0.47346099	0.742440333	1
HPCA	0	1	1
PTBP2	-0.264060686	0.861743995	1
NCAPG2	0.400041248	0.724463674	1
ADAMTS12	0.255648941	0.865968458	1
TLNRD1	-0.412705595	0.783811713	1
LZTS1	-0.501215016	0.732841487	1
GSTM1	-0.94854461	0.497577647	1
FAM117B	1.379067865	0.348282143	1
CNEP1R1	-0.653068688	0.506239378	1
GPR153	-0.615745016	0.678501028	1
TXNL4B	0.936661422	0.527942809	1
SEC14L2	-0.251772835	0.800467062	1
LRRC8D	0.706725534	0.646809369	1
MTMR14	-0.583194964	0.678797085	1
GRIK5	1.114572988	0.389511306	1
RP2	2.028264488	0.101075048	1
DTD2	0.553794564	0.639867411	1
UBASH3B	1.723567127	0.246258913	1
TARBP1	0.133434872	0.929165565	1
LHFPL6	-0.491004288	0.617064977	1
ZMAT3	-0.038974935	0.979408225	1
PLGRKT	0.56598437	0.638112504	1
LFNG	0.037800512	0.978866845	1
NLRX1	-0.100270805	0.94346061	1

CHSY1	-0.008963333	0.99474606	1
NMI	-0.314895682	0.835015859	1
ANAPC10	0.306222047	0.845405694	1
PLEKHA2	-0.391481776	0.715875685	1
CHTF18	1.324275794	0.257566625	1
GAPDH	-1.021049576	0.330898519	1
ALDH1A2	-0.591058835	0.598417226	1
EXOG	0.300913487	0.771976761	1
ZNF451	-0.780853099	0.55984051	1
RBKS	0.403152228	0.684998008	1
CSNK1G3	0.585374594	0.716183828	1
PSTPIP1	0.856405188	0.511693643	1
SERPINA12	0	1	1
ERG	-0.839872592	0.533201653	1
SNAP91	4.106484475	0.094778033	1
ZFP64	0.298162918	0.82084484	1
STK26	0.200430195	0.895346701	1
USP18	1.096061298	0.44878205	1
SCD5	0.342781664	0.813227385	1
SLC2A13	2.966804374	0.083554919	1
PRSS56	0.659379577	0.659978812	1
SLC66A2	-0.559730614	0.666735017	1
ENSBTAG00000051647	0.526538247	0.641785774	1
SLC10A1	0.265551216	0.829336732	1
PRXL2C	-0.153410759	0.912404384	1
THSD4	0.152577583	0.93106485	1
GALNT10	-0.362954443	0.729244893	1
CMTM8	1.174543886	0.373398301	1
EPHA10	0.385994713	0.819646677	1
TMED8	-0.086676832	0.954319271	1
GABPB2	0.544325473	0.746725898	1
ATM	2.810647561	0.179166227	1
E2F8	2.122440146	0.284669853	1
SCFD2	0.066924496	0.949593164	1
BAG4	0.13748835	0.919708329	1
SORBS2	1.21870203	0.48003398	1
TEPP	-1.88450582	0.421627185	1
SPINDOC	-0.322847604	0.752430771	1
RGS14	-0.725610305	0.648104479	1
ADAM11	2.466441869	0.090433412	1
FRS2	0.568885926	0.724525537	1
NMNAT2	-0.665104757	0.614282807	1
RFLNA	0.093965239	0.948313537	1
G24157	0.778296776	0.662751187	1
SRC	0.230365008	0.832606136	1
TMC7	0.821531768	0.61328639	1
RLF	0.445744701	0.771172844	1
ARL13B	0.447509499	0.714325037	1
G27637	-0.200137094	0.896573351	1
CHRNA1	-0.68403447	0.598751936	1
ENSMMSG00000021770	-0.146469304	0.889381505	1
PLCL1	0.108948131	0.944952018	1
G37761	-1.071099197	0.315161846	1
ZEB1	-0.23337471	0.885716404	1
C7H19ORF44	0.639032671	0.705010119	1
ICE2	-0.107564849	0.94240987	1
LOC520336	1.195154495	0.379493454	1
SS18L2	-0.741126181	0.434372961	1

TNFSF12	-0.225218461	0.841400355	1
ZMIZ2	-0.337625744	0.727212128	1
SMAP2	-0.442927259	0.763181483	1
VWA5A	-0.170407504	0.906224692	1
EPS15L1	0.854505615	0.535829027	1
ENSMMSG00000024257	0.383120955	0.830299606	1
SAYSD1	1.943767091	0.087356966	1
KLHL23	-1.064099465	0.44134252	1
HNRNPM	-0.464428192	0.622763266	1
BRI3BP	1.101868509	0.334569127	1
TSSK4	-1.016568337	0.646163954	1
ENSBTAG00000051168	0.555939923	0.731920815	1
GPR4	-0.096910104	0.927667105	1
FREM1	0.54964866	0.793400416	1
SUV39H2	0.762051162	0.426645052	1
ZBTB12	0.216693504	0.886836172	1
FCHO1	1.381379157	0.241333093	1
KIF3C	0.22596345	0.884846246	1
PHC1	0.507503805	0.754379291	1
PTPDC1	-0.510520781	0.721242113	1
NCAM1	-0.043376864	0.974588013	1
WNT11	-0.906252138	0.527890976	1
AKAP11	2.37989441	0.197258524	1
LIMS2	-0.74128701	0.628079534	1
PARP14	-0.327866637	0.754094748	1
PLLP	1.658070271	0.289336199	1
G256	0.314021346	0.796918782	1
AP5S1	1.302728075	0.328806958	1
WSCD1	-0.729140211	0.577357858	1
SLC5A2	0.611544566	0.574772872	1
PI4K2B	0.604723365	0.679610341	1
BNC2	0.036631984	0.979845948	1
RGS20	0	1	1
GALNT12	-0.169842295	0.874196427	1
CHST10	-0.606686463	0.576178814	1
ZNF174	0.398001423	0.698929361	1
TRMT5	0.191007946	0.902737225	1
PCDH17	-0.656313867	0.652047273	1
CCDC50	-0.366779673	0.855099917	1
SWSAP1	-0.004286913	0.997722719	1
SNX27	-0.201558185	0.889764541	1
SULT1A1	-0.834391334	0.530976324	1
ENSMMSG00000016395	0.835796911	0.569943512	1
ISCA2	-0.453536434	0.738580951	1
SIAH1	-0.356487372	0.806168004	1
FAM114A1	-0.477981803	0.739353456	1
SLC22A23	0.04603303	0.972612802	1
FBLN5	-0.940810285	0.502398501	1
CCSAP	0.4661931	0.779067961	1
LOXL4	1.381602603	0.313287531	1
LOC107131254	-1.807647909	0.691404321	1
RAB11FIP2	-0.328994264	0.82080718	1
CHRD	1.910822141	0.123848327	1
RNF139	0.303891318	0.78696165	1
MAP3K20	0.030435371	0.983286724	1
SLIT2	0.232173175	0.869639063	1
PARP6	-0.602349833	0.521724041	1
BLOC1S6	0.049700555	0.973916202	1

RILPL2	-0.769753078	0.446427584	1
SOX6	-1.211250849	0.315445144	1
KIAA0408	0.209043317	0.8957667	1
RAP2B	-0.278175976	0.792612824	1
SMC1A	-0.186844679	0.900994366	1
TBC1D5	-0.110465435	0.937412305	1
TNRC18	0.259595752	0.91359685	1
BRCC3	0.118380078	0.919191667	1
KRT75	2.185441721	0.370010662	1
ZDHHC23	-0.168118208	0.874135011	1
C17H12ORF43	0.605305351	0.608719513	1
ZBTB41	0.626213485	0.582200038	1
TTC9	-1.012595679	0.388540993	1
ABCG2	0.134124666	0.903143918	1
KCTD12	-0.274557061	0.803440491	1
TMEM135	0.753834161	0.523829705	1
LYRM9	-0.032168768	0.983085693	1
CDK2AP1	-0.616081452	0.507430458	1
RNF168	-0.612786259	0.67566507	1
TFB1M	-0.231773589	0.851659958	1
DHDDS	1.967930185	0.27862568	1
PPIA	1.111540054	0.373811707	1
CDIN1	1.044763598	0.542116493	1
MFAP3	-0.362398433	0.797472232	1
PPP1R14C	-0.988602257	0.42754218	1
RAB27A	1.304210395	0.242350681	1
CDK3	-0.109113632	0.94225015	1
SLC36A1	0.270212353	0.852769708	1
OAF	-0.60011932	0.6604474	1
LOC101903064	2.489613916	0.08551845	1
RCBTB1	0.199864338	0.889067358	1
SHANK3	-0.489025473	0.748747506	1
HNMT	-0.469287716	0.730114049	1
ZSWIM4	0.591145157	0.615823179	1
KCNAB3	-1.827284672	0.421609007	1
ATP23	0.565883861	0.602848733	1
CXCR4	-0.577418682	0.688285108	1
TPST1	-0.395687173	0.771146528	1
RASGRP3	-0.573786539	0.680800329	1
SCARA3	-0.349963643	0.803637844	1
PTH1R	0.734582176	0.508448578	1
NUP133	0.820644653	0.614857459	1
HOXA7	0.114976937	0.919797386	1
KRT27	4.137962666	0.349991246	1
ACBD6	-0.485595027	0.664618093	1
EEFSEC	-0.604627623	0.495289515	1
TSGA10	1.612078422	0.090829733	1
G6255	0.478943567	0.641809373	1
CEMIP	2.881018702	0.107821299	1
TMEM18	-0.0087289	0.991250494	1
ELN	2.3390335	0.200450833	1
FAM13C	-0.778282826	0.590511466	1
PPM1H	1.302659397	0.368582308	1
SIGLEC10	0.021879967	0.983972667	1
PPIP5K2	0.988555668	0.65820748	1
METTL27	0.901697953	0.42374787	1
TMEM125	0	1	1
TMTC2	1.206161501	0.323149536	1

MB21D2	0.459344714	0.791226884	1
GTF2A1	0.525512394	0.676491224	1
SLC8A1	1.556069196	0.177624685	1
KIAA1217	-0.191923264	0.890546987	1
RIN2	0.329448634	0.866126584	1
TGFB2	-0.305104148	0.828864368	1
MAML2	-0.120481301	0.90109921	1
DIPK2B	-0.730291698	0.4343539	1
ZNF641	-0.605574828	0.678338161	1
IRF5	-0.135179874	0.902304806	1
DTX3L	-0.384511863	0.802597951	1
MTLN	-0.247760684	0.820546594	1
UIMC1	0.054370206	0.95794794	1
RAB32	0.85685718	0.34907562	1
ENSMMSG00000009643	-0.454154698	0.772130599	1
FBXL12	0.507448012	0.705505776	1
ZNF33B	0.882094451	0.483037124	1
CEP19	-0.683282828	0.475144853	1
HEY1	-0.292922932	0.845252517	1
PDGFC	1.335939275	0.362065137	1
RPAP2	-0.133929649	0.929637906	1
SELENOT	-0.001804081	0.99899641	1
TMC8	1.383993127	0.178721118	1
RPS23	0.438622536	0.718429302	1
OXSM	-0.724708901	0.472479674	1
COL21A1	1.426175486	0.435462858	1
BRD3OS	0.110985923	0.912957261	1
COX6B2	-1.883225722	0.417985491	1
ARHGAP30	0.380618228	0.714003904	1
CFAP251	0.648881917	0.663151525	1
MIC1	-0.272174644	0.84979263	1
ZNF496	-0.356685115	0.755239315	1
TTC32	0.338978631	0.68045455	1
CES5A	3.849430297	0.113361182	1
KLHL18	0.295976378	0.851677722	1
C15ORF62	0.516957	0.574184983	1
INIP	0.275314396	0.807819449	1
MTX3	0.554666636	0.699416489	1
MED18	-0.398312043	0.789067264	1
F2R	-0.422724374	0.765042526	1
SPTBN1	0.26047008	0.811919715	1
PLEKHM3	-0.27879279	0.790947244	1
CACNA1D	2.346412109	0.217064443	1
EME1	0.559841044	0.705103263	1
FOXN4	0	1	1
GON7	0.996509274	0.471927152	1
WDR70	-0.142491887	0.877570209	1
SHISA5	-0.101653638	0.96404001	1
MARVELD1	0.68414803	0.530174261	1
CCDC92B	0	1	1
HSD11B1	-0.182858701	0.898456572	1
CLDN23	0.759933456	0.742791083	1
DTX1	0.551691528	0.529533829	1
RRM2	0.418196769	0.842331396	1
SIM2	0.136094396	0.954216497	1
APBB2	-0.397445441	0.77802127	1
ANO9	2.274704898	0.12860744	1
BRCC3	-0.627384593	0.649559286	1

POU2F1	1.061729041	0.373526574	1
PUDP	-0.279694528	0.79511675	1
KDELR3	0.011202387	0.990802455	1
OOEP	2.288144518	0.183435413	1
TMEM140	-0.586852789	0.686914219	1
MAP3K12	0.082340945	0.941723651	1
ADGRG6	0.976023417	0.479506118	1
NEPRO	-0.465267609	0.646357432	1
CENPT	2.651702638	0.149503686	1
KIF16B	0.724378989	0.599758541	1
TMEM17	0.778732355	0.526434702	1
FBXL18	1.074291099	0.480389525	1
OSBPL10	0.353350076	0.804174083	1
KANSL1L	1.196714144	0.486742629	1
KIF3C	0.692617108	0.608117118	1
TMEM86A	1.165136438	0.295378294	1
TRIM21	0.738285482	0.596672095	1
G17213	-0.111468348	0.934363756	1
SDR42E1	2.377942808	0.154691041	1
NFATC1	-0.092477306	0.945501938	1
CYP26B1	0.510762091	0.701846913	1
UNC5B	0.291305467	0.784452148	1
G4961	0.361612161	0.807813504	1
C5AR1	-0.409118618	0.781343814	1
CALCRL	-0.509244437	0.708253593	1
ELP6	0.671123634	0.692759459	1
CNTN2	-0.108041149	0.906228805	1
PARP12	-0.355543071	0.793453798	1
SKA2	0.331591813	0.77986063	1
RXYLT1	0.286384396	0.838279249	1
PPP1R16B	-0.912455589	0.490696303	1
UBA7	1.051176028	0.411066212	1
C20H5ORF22	0.055208138	0.972379267	1
ELMO1	-0.424356086	0.753593295	1
DRG1	1.22035386	0.38728638	1
BTC	-0.858124837	0.459211436	1
DCHS2	1.886247416	0.180175239	1
CBX6	0.343612816	0.76132445	1
PIP4P2	-0.856592217	0.534866889	1
C18H19ORF12	0.164598911	0.893444917	1
GRIP1	2.093453836	0.359805597	1
NCF2	0.481072217	0.805535737	1
SUDS3	0.15444752	0.905151008	1
ZNF606	0.561511108	0.705865323	1
CNOT6L	-0.145764967	0.941632693	1
ZNF19	-0.118642029	0.906965742	1
FAM189A1	2.156972703	0.238477326	1
TUFT1	2.555187407	0.136348294	1
SH3GL1	0.336852796	0.755627611	1
MYCBP	0.735235611	0.501064988	1
KRBA2	0.820999808	0.416173114	1
IFFO2	0.31864775	0.708844908	1
PHLDB2	0.429223602	0.663516375	1
FAM104A	0.071607167	0.940489393	1
SSH3	1.423988733	0.360179278	1
ENSMMSG00000004355	1.593967557	0.522260798	1
RPS26	-1.829119513	0.407781756	1
ANKRD9	-0.988220393	0.474066221	1

KLF7	-0.529277058	0.717665446	1
RABL2A	0.094284024	0.947487947	1
CELF6	1.425271872	0.361408354	1
DIPK1A	-0.914975288	0.33801529	1
CDK2	-0.260582981	0.863892707	1
SETDB2	1.27011181	0.445081625	1
RSRC1	-0.562243245	0.496859047	1
SORCS2	-0.034894412	0.986452716	1
VWA3A	-0.053076728	0.971518473	1
ZFTA	0.049029477	0.972777654	1
HOXC6	-0.583754539	0.688751449	1
TLE1	-1.051904145	0.45481492	1
CNOT6L	1.309043649	0.445033485	1
SFT2D2	0.755727431	0.495736972	1
ZBTB45	0.701335735	0.523654111	1
CARD10	0.359006418	0.807717848	1
RAB4A	-0.558005088	0.600904205	1
METTL5	1.106594286	0.438560045	1
KLF16	0.76161051	0.586569078	1
LCOR	-0.666486959	0.471059279	1
PFKFB1	-0.714536036	0.456700058	1
DMD	-0.238045233	0.897232368	1
SPATA20	0.713420548	0.510580563	1
RFC3	-0.325623398	0.705421109	1
FADS2	-0.820692828	0.397296189	1
PHF10	1.670541693	0.171348038	1
EME2	1.015775887	0.343980503	1
AGAP2	-0.288447295	0.733241175	1
N6AMT1	-0.2420253	0.871837959	1
FBXO25	0.102826218	0.939849312	1
SIGLEC1	0.906229781	0.438721877	1
B4GALT1	-0.586380276	0.807265596	1
TMEM104	1.020162993	0.55343937	1
FOXRED2	1.311380073	0.350791429	1
MSANTD4	1.027684421	0.515636983	1
SLC22A16	-1.035536103	0.330466058	1
CNTN1	1.379239452	0.339619668	1
MXD1	0.790323003	0.531299919	1
ZXDB	0.849982937	0.531266922	1
SMAD7	-0.292064953	0.773879069	1
NEDD4	-0.518160246	0.70258564	1
LOC618542	0.427802962	0.706061158	1
S100B	0.972527858	0.54325464	1
GLYCTK	2.04733612	0.177295604	1
TTC39C	1.232458372	0.351338732	1
RNASEL	-0.386989064	0.722476607	1
SLX4IP	0.205484683	0.88669754	1
PRKX	0.146073056	0.883130768	1
RBBP9	0.230636568	0.823874405	1
FER	0.724812394	0.505190465	1
FLYWCH2	-0.458722616	0.678226434	1
BCL2L2	0.377319391	0.712470961	1
ACYP1	0.289979741	0.786866678	1
ITIH4	1.122888064	0.445312808	1
VPS8	1.143465927	0.349164155	1
ATP6V1E1	0.698236394	0.533984402	1
DPYD	-0.449703417	0.738180795	1
SHB	-0.166447084	0.88633149	1

PLD4	1.802019135	0.135021591	1
IL31RA	0	1	1
EGR2	0.202302613	0.883521471	1
STAMBP	-0.21575879	0.886109186	1
NANP	0.257363277	0.787259589	1
TRIM32	-0.207403213	0.892268304	1
TMEFF1	2.644390291	0.190720002	1
ZWILCH	0.064632677	0.967053629	1
TPM3	1.256744887	0.259248771	1
LOC507443	-0.051028958	0.960597306	1
FAM120A	-0.189765383	0.903079211	1
PAPLN	0	1	1
TYROBP	-0.14343853	0.910007772	1
WWOX	1.432486442	0.156440692	1
SULF1	-1.024126485	0.44544974	1
LY75	2.808301942	0.141680288	1
FEZ2	0.089433744	0.953857909	1
RABEPK	1.166569891	0.378150105	1
CNOT4	-0.57803115	0.689289915	1
KCNMB1	1.291499369	0.284652285	1
CPM	0.382740417	0.730977139	1
RPP14	-0.490624615	0.588704457	1
EVI5	1.398417968	0.365205147	1
EPB41L3	0.282573773	0.853278697	1
ATPSCKMT	-0.34693076	0.753168639	1
DCLK3	-0.795477214	0.648763856	1
PPARG	0.495879554	0.674491022	1
PRKCE	-0.645810338	0.680657411	1
ANKRD49	-0.213823748	0.880745349	1
BOLA-DQA2	0.344201274	0.72339393	1
BLA-DQB	0.168876528	0.86058861	1
PGK1	-0.903455456	0.490170488	1
TECRL	-0.104085624	0.947699993	1
NFXL1	2.55372927	0.218595333	1
B3GNTL1	0.619732198	0.503817826	1
SAFB	0.19012881	0.86080055	1
P2RY6	-1.828689203	0.410946765	1
ZNF668	-0.191621017	0.848165755	1
DAB2IP	0.43899349	0.845236068	1
SLC39A9	0.49327699	0.733792437	1
HOXA4	-0.541476392	0.584490955	1
IL33	-1.057669795	0.383286735	1
ITGA2B	1.028737855	0.420036678	1
CRPPA	-0.491304747	0.741629259	1
CHRAC1	-0.129547124	0.896784805	1
ADAT1	-0.278447708	0.836452307	1
SLC25A38	-0.366995062	0.792357429	1
SLC34A3	2.549053405	0.284763792	1
ZNF783	1.667476903	0.333439075	1
AMFR	-0.125688776	0.92898982	1
CHAD	-0.676290922	0.671443438	1
SH2B2	1.867454817	0.455115538	1
GEM	-0.383489278	0.805001139	1
NGEF	4.214599491	0.093616634	1
NICN1	0.713450698	0.549701267	1
CSKMT	-1.133297012	0.414358345	1
FOXJ1	-1.82557331	0.435442579	1
LGALS9	0.514176693	0.584695871	1

ZFHX2	0.040603471	0.962977815	1
MSN	-0.358980521	0.805716666	1
NCMAP	2.035223419	0.297889832	1
REST	-0.244969955	0.875051165	1
GLI3	0.774827262	0.472001522	1
MTMR11	-0.604516112	0.542371182	1
DACH1	-0.41188141	0.692224483	1
AATK	0.570729598	0.701463269	1
AP4S1	-0.834270461	0.562962844	1
CABLES1	0.199254513	0.821115668	1
SBF2	-0.135787132	0.889168466	1
BRF2	-0.520107679	0.646199673	1
PIP4K2A	-0.496686521	0.717464205	1
CENPX	0.996263216	0.329148691	1
ARHGAP26	-0.075677217	0.939787581	1
CHI3L1	1.778038538	0.163461119	1
EFNA4	1.246010595	0.331324345	1
VIT	-0.856847257	0.539546712	1
TEKT1	-0.377959388	0.729103948	1
MAPK14	0.30198394	0.76704079	1
SLC18B1	-0.827542972	0.537310044	1
SPRY4	-0.466532264	0.610063061	1
ZNF445	-0.357868359	0.808125362	1
ENSMMSG00000023211	1.561586417	0.358829563	1
G13771	3.584201271	0.120491625	1
STX17	0.35632964	0.684827908	1
NSUN6	0.678811993	0.590806881	1
PLD1	1.217099214	0.299393593	1
PCSK6	-0.041474475	0.972125006	1
BTG2	-0.888459638	0.397702645	1
MFSD2A	0.613585917	0.79342826	1
FOXN3	-0.068961554	0.95599281	1
PLEKHG6	2.252974112	0.130779532	1
METTL5	0.550026626	0.624140709	1
DCUN1D2	-1.236765933	0.290423118	1
ATP6V1C2	0.435377815	0.719824644	1
COX18	0.75596156	0.54547503	1
NCAPH	0.094464518	0.914041502	1
KHK	-0.312877014	0.833528581	1
FAM229A	1.820979763	0.165123468	1
QPCT	0.937237287	0.434684165	1
TCTA	0.679375869	0.425947204	1
ALOX15	-0.675177585	0.582293105	1
CA4	0.219060217	0.84337147	1
WDHD1	0.592476165	0.656676989	1
CEP76	-0.656411401	0.658454134	1
SLC31A2	-0.189711629	0.901399141	1
PRORP	-0.180796452	0.810267774	1
TRIB3	2.953889986	0.199652408	1
RFWD3	0.796067314	0.586468596	1
GPATCH2	1.137028675	0.370921251	1
TRIM52	-0.522365049	0.702329369	1
PAQR3	-0.25075404	0.868129634	1
NSL1	0.230728842	0.883685082	1
C19H17ORF80	0.950241642	0.336379787	1
KASH5	0.842315614	0.276274296	1
BBS9	-0.76313607	0.453924627	1
NPFFR1	-0.064084908	0.974575245	1

RCCD1	0.118502117	0.923309583	1
ANKRD13C	0.115970872	0.895564932	1
NPR3	0.118996009	0.945294497	1
PRELID3A	0.3104992	0.806802128	1
XXYLT1	0.573476041	0.691881879	1
BRF1	-0.430767258	0.710268407	1
HSD17B7	0.902554908	0.357086887	1
RBM26	0.715156344	0.659632304	1
SHF	-0.339606218	0.688767651	1
GPR180	2.45152588	0.13217578	1
WDR20	-0.227742259	0.872521436	1
PDE3B	-0.58901318	0.67794366	1
ROM1	0.457536471	0.704823625	1
ZDHHC2	1.06716013	0.431713909	1
RWDD2A	1.732848291	0.322702316	1
PTPN6	0.977160371	0.559445524	1
TMEM167B	0.422275153	0.784789966	1
CEP97	-0.348109767	0.806406189	1
KIF1A	1.319827613	0.446684667	1
CARD11	0.424233256	0.619121778	1
PARD3B	0.777318079	0.61778487	1
TGIF2	-0.479447767	0.663946766	1
CHRDL1	1.200707965	0.622031381	1
FIGN	2.131782191	0.202304358	1
ITGB7	1.805949752	0.117771968	1
CDC25B	0.107642886	0.917218547	1
RAB31	-0.767569	0.537079436	1
MTRES1	-0.7573968	0.466649122	1
PEX10	-0.360530784	0.765564418	1
AKR1E2	1.330847498	0.239025317	1
FMNL1	-0.167579733	0.906469546	1
HACD3	0.348791208	0.730407045	1
ATP10B	1.251350561	0.502599865	1
TRIP13	0.1493562	0.885067078	1
CIP2A	0.439188695	0.722235083	1
ADAMTS14	2.006516256	0.248706844	1
IFNW1	0	1	1
ZBTB2	0.952788798	0.568619388	1
ACP7	3.626696315	0.183664449	1
TSEN15	-0.071461408	0.961675492	1
DCAF1	-0.121987684	0.887359614	1
SRGAP2	0.136365406	0.954214224	1
EDA2R	-0.065623249	0.960348718	1
SOS2	-0.025830133	0.988053286	1
TRAM2	-0.264337476	0.796604094	1
CD3D	-1.88193502	0.414309171	1
DCK	0.914491486	0.543480208	1
DEGS1	0.399774215	0.716712898	1
SLC45A4	1.43806715	0.272811177	1
SFMBT2	0.369145092	0.850910368	1
HDGFL3	0.422898186	0.797062805	1
MC1R	-1.823591254	0.452753641	1
G6305	-0.100065509	0.94988154	1
BET1	0.238117324	0.789388337	1
DKK2	0.556646706	0.679800904	1
HSD3B7	-0.608044136	0.544848517	1
SYTL4	-0.181435791	0.850555238	1
CCDC170	-0.499557912	0.728046168	1

ENSBTAG00000051176	-0.489492392	0.762960265	1
TAF13	-0.04956406	0.972152506	1
MAGED4	1.651944506	0.188119664	1
CARS1	1.700052114	0.151791186	1
ARMC2	-0.898813437	0.509801172	1
ZNF649	-0.159592355	0.880369831	1
TCF7L1	-0.66892615	0.628452956	1
ZSWIM7	1.221977381	0.4891752	1
MRGBP	-0.019755078	0.985239877	1
ARMH4	2.140997168	0.305290551	1
ATP10A	-0.057351556	0.964586127	1
ZSCAN26	-0.231186474	0.783954577	1
DNAAF2	0.670528887	0.594809378	1
KCNC1	-0.931599253	0.366441216	1
KLK1	1.290314475	0.40117571	1
L3HYPDH	0.19892239	0.895407126	1
DPYD	-0.048441053	0.973947794	1
ABI2	-0.399237645	0.790358292	1
CDH16	2.412869689	0.222943224	1
MEDAG	-0.936056636	0.255189471	1
CD58	-0.138551316	0.888241492	1
COL27A1	1.575297989	0.1668936	1
DHFR	0.102132094	0.931501846	1
PLEKHA6	1.465822885	0.451816811	1
G9382	1.48165048	0.274847337	1
ENSMMSG00000006577	1.562257107	0.208484108	1
C16H1ORF112	-0.525691453	0.599488011	1
SMIM8	-0.756408426	0.419389948	1
MFSD13A	-0.756065324	0.450735782	1
P2RX4	-0.273079086	0.849018341	1
PPP1R26	1.685389155	0.251011051	1
ULK4	0.06218244	0.948690336	1
ITIH3	-0.31026602	0.773421705	1
EEF1A1	1.687688266	0.122902622	1
EEF1D	1.7197363	0.215146604	1
DLGAP4	-0.806096545	0.33932471	1
NDE1	-0.521658794	0.573880295	1
BMP6	-1.054548648	0.275832118	1
CAMTA1	-0.008242537	0.995974358	1
NIP7	0.07257429	0.960313997	1
CSNK1G3	-0.182210012	0.90318503	1
ODAD1	-0.104842846	0.926422838	1
LSM5	0.038840565	0.971125709	1
SORBS3	-0.256299818	0.908703159	1
TP53I3	2.000476372	0.088012361	1
ANGPTL1	-0.57510144	0.704680517	1
DKK3	0.374450346	0.808511904	1
LRRC32	-0.095563193	0.923390099	1
G22917	-0.118478422	0.940736456	1
ERAP1	1.495636998	0.307912035	1
FAM170A	0	1	1
BMT2	0.265637228	0.859051494	1
ENSBTAG00000045912	-0.040325579	0.979386385	1
SBK1	-0.230306485	0.867124518	1
KCND3	0	1	1
CIT	0.838206723	0.449606613	1
LIMS1	-0.285718326	0.876770592	1
PCGF5	-0.06178193	0.966461357	1

EVA1B	1.300859774	0.306911046	1
LIN9	-0.42344469	0.773874398	1
TRMT10A	1.891914064	0.173422558	1
IKZF5	-0.146173021	0.927267041	1
SLC26A10	0.107610584	0.937321563	1
SBK2	-0.84417759	0.65012101	1
TGM5	0	1	1
RPGRIP1L	0.930960749	0.397572475	1
AK8	0.307449055	0.874242199	1
BRMS1L	-0.427024422	0.780153354	1
PDE7B	0.762400596	0.655489389	1
ZNF263	0.297317877	0.761261969	1
TBX2	-0.176240751	0.867288321	1
TXNL4A	-0.690809396	0.391739409	1
SPRED1	0.072392909	0.940585661	1
ABITRAM	-0.329022061	0.756213831	1
C16H1ORF74	0.899541851	0.501974229	1
USP53	-0.163149582	0.868631582	1
TMEM59L	1.391828876	0.380067414	1
MED20	0.393430658	0.798205142	1
OSR2	-0.659126547	0.567516066	1
RAP2C	1.188828547	0.379465742	1
SH3RF3	-0.884971989	0.565513275	1
RGS3	-0.672095212	0.380147028	1
ARID3B	-0.834180582	0.546022193	1
IL1B	-1.27886274	0.222398665	1
DBH	3.635379322	0.134808254	1
TMEM154	2.188914997	0.176514724	1
FBXL18	-0.322221576	0.873226637	1
PKHD1L1	0.997218758	0.266807866	1
KDM4C	0.842042019	0.416416283	1
SUDS3	0.093493062	0.934845514	1
MISP	2.238486614	0.131107576	1
ADAMTS7	-0.315633451	0.793167134	1
TRMT61B	-0.170818065	0.877110424	1
G14658	0	1	1
STK35	0.729394193	0.481563006	1
CDH23	-0.816059301	0.343445459	1
FNDC5	-1.067973614	0.387443009	1
ELOVL5	-0.756885364	0.435083657	1
SUGP1	1.264759527	0.619387321	1
FBXO2	1.291703584	0.238970324	1
LY96	1.770904188	0.138155899	1
CUTC	-0.7004295	0.536526105	1
CCNE1	0.06147858	0.96711674	1
VGLL3	-0.427310564	0.729849672	1
ELK4	1.278503405	0.456030155	1
NONO	0.276889232	0.755258659	1
LEPROT	0.232207185	0.82651858	1
FBXO33	0.381296628	0.640889109	1
ENTPD7	0.051384917	0.971402959	1
SPAG8	2.410756492	0.089427974	1
TTC30A	1.403097984	0.369076974	1
MTFR1	1.926972686	0.271109853	1
KIF20B	-0.100751936	0.922383047	1
NIN	1.594840913	0.156602994	1
SLC39A3	0.298158722	0.759548969	1
IDUA	1.298367283	0.192200986	1

PROX1	0.419118894	0.834198601	1
STRADA	-0.210633888	0.908872227	1
SCUBE2	-0.537847762	0.731597852	1
CDHR5	0	1	1
NCEH1	0.843216344	0.631369791	1
SANBR	0.09408119	0.93884905	1
CDK11B	-0.358448906	0.796607053	1
ENPP1	-0.638260938	0.665542899	1
RALGAPA1	0.232442476	0.825502992	1
ABCG1	-0.633069572	0.547423295	1
HELQ	1.057443245	0.382849289	1
RAB6A	-0.14099209	0.929454675	1
PRDM10	0.252051757	0.872883642	1
CPSF4	-0.497923644	0.669133001	1
COL4A6	0.584485809	0.583363734	1
DYNC1I1	-0.863040424	0.505467595	1
GUCY1A1	-0.87975764	0.505533062	1
IGFALS	1.685837826	0.1964133	1
IER5L	2.271142677	0.108019365	1
PCNT	-0.6298235	0.692270717	1
SLC35A2	0.208173849	0.84051818	1
ZRSR2	1.191214846	0.337595172	1
OPHN1	2.354670079	0.087245745	1
UNC5CL	-0.219496398	0.905389003	1
EDNRB	-0.450804626	0.740039693	1
PLAU	0.247205238	0.87803518	1
AURKC	0.653241824	0.544353335	1
NAT9	0.293305734	0.816471515	1
ERO1B	0.67447726	0.636486397	1
THAP9	-0.548724429	0.542277106	1
LYSMD1	-0.564078677	0.593083876	1
APBB2	-0.6847981	0.623684482	1
MAPRE1	-0.199758062	0.841787987	1
MIA3	1.252759002	0.449304073	1
CASD1	-0.201155994	0.894164136	1
AGXT2	0	1	1
USP49	0.67612527	0.565956445	1
NR4A3	-1.23274917	0.351598279	1
RHOU	0.338028706	0.735673048	1
RARG	-0.536588718	0.692420867	1
BMX	0.724550827	0.490308173	1
TMPRSS9	0.730023137	0.660109338	1
ST6GALNAC6	-0.538936545	0.726183182	1
ZNF274	0.184564635	0.857358247	1
TTBK2	-0.677383786	0.630305914	1
CFAP157	1.629344807	0.22317509	1
SPON1	0.318781278	0.760367624	1
DIAPH3	1.449114811	0.445262339	1
CHGA	1.10520191	0.525365656	1
DHX58	0.214819363	0.861601039	1
NXPE3	0.566225491	0.727487929	1
UBAC2	0.117541922	0.92837063	1
MRC1	0.212144736	0.889085412	1
COL4A5	0.805256449	0.572761035	1
SERPINB6	0.1559407	0.90038589	1
RIN2	1.380409305	0.463606224	1
COLGALT2	0.25182526	0.850396899	1
HEYL	-0.908179369	0.330353077	1

GRIN2C	0	1	1
PSD	1.978391388	0.128132495	1
CDK8	2.386479024	0.157523203	1
S100PBP	0.57777959	0.587509353	1
MPEG1	0.618618821	0.68963999	1
ADGRV1	0	1	1
NBEA	0.204893544	0.89631254	1
ANXA9	1.749898969	0.258675859	1
PPCDC	-0.333237077	0.745883163	1
MAPK8IP3	2.70106002	0.094873066	1
BMP4	0.381027961	0.746879566	1
RCAN2	0.749676067	0.683542728	1
CEP72	0.99736327	0.515505516	1
ECHDC2	-1.606955823	0.296765706	1
SLC13A3	1.586928333	0.334134037	1
C7H19ORF71	0.003317816	0.997308536	1
SLC27A5	0.648570628	0.499450436	1
GINS4	-0.28359019	0.844092914	1
BEX4	-0.425545137	0.679864209	1
ORC3	0.432612876	0.803860465	1
CREBL2	0.383035701	0.803667287	1
CATSPERG	-1.876340959	0.398452843	1
ZFP69	-0.30255848	0.766544982	1
MCM9	1.84087661	0.269808891	1
NATD1	-0.55473625	0.708427955	1
UBXN6	0.536870461	0.684288479	1
PHF10	-0.057819536	0.969790112	1
ERCC8	0.012354814	0.991203174	1
ICAM3	-0.406487571	0.648643585	1
UBE2V1	-0.071443661	0.971096326	1
ALG6	0.781997267	0.486382607	1
PPFIBP1	-0.941829087	0.461299892	1
AKNA	0.183162426	0.821619463	1
OBI1	0.735489509	0.656680718	1
FAM76B	1.225909045	0.321964657	1
APTX	0.274195843	0.855481336	1
DENND5B	-0.327395007	0.839573004	1
XPR1	-0.173095827	0.907315095	1
TEK	0.054380627	0.9729636	1
TPX2	-0.178636553	0.925613032	1
NBEA	-0.101416222	0.95038444	1
PGAP2	0.118298961	0.889436344	1
ALDH1L2	-0.214343669	0.8869444	1
INTS6L	0.083740404	0.953261999	1
CDC20	2.830762357	0.110987825	1
MARCHF8	2.069277272	0.215149251	1
USP1	0.40943901	0.789700061	1
CMC4	1.164075248	0.379374614	1
LDLRAD4	-0.650271831	0.632498104	1
COIL	0.326585678	0.83229185	1
ENSBTAG00000004921	-1.051268873	0.264428488	1
IFFO1	-0.095267675	0.945146653	1
DPYSL5	0	1	1
TSSK3	1.095188066	0.368481605	1
TBC1D22A	0.302418928	0.82691616	1
NFAT5	2.381737391	0.179797678	1
SDHAF1	-0.151903015	0.887219102	1
TRPC2	1.002238783	0.388495645	1

ARHGAP25	-0.2172722	0.848203564	1
TRIM38	-0.176235056	0.866835085	1
IGF1R	0.970748702	0.567472967	1
LOC512486	0.992830774	0.409202874	1
DCTPP1	0.382918594	0.691565278	1
IGHMBP2	-0.378997631	0.791771698	1
MSI2	0.048773615	0.974278493	1
ANKRD16	-0.104905371	0.908629266	1
ESRRG	0.665471433	0.742515172	1
WDFY3	0.617425304	0.796175732	1
PPT2	-0.352891886	0.813270911	1
TMEM8B	0.575546751	0.663385119	1
MGLL	0.305758819	0.863167176	1
PRIM2	-0.236764545	0.82461719	1
POPDC3	-0.481481336	0.666288175	1
SERINC4	0.358639514	0.736386681	1
MYEF2	-0.770554816	0.664588349	1
ECM1	-0.846305889	0.747420021	1
WARS2	1.432918586	0.390053544	1
OLFML2B	-0.565044365	0.788617057	1
CFAP251	1.399286461	0.479364563	1
ZBTB49	-0.368790781	0.792635329	1
GARIN5A	-0.792722687	0.556583018	1
CLMP	0.773204399	0.610720177	1
CLDN12	0.523526698	0.680674878	1
ACTR5	0.511342939	0.666253077	1
MMP19	-0.878461859	0.55116917	1
ATXN7L3	1.648317625	0.232383377	1
FUT11	0.0582277	0.951592155	1
ENSMMSG00000000495	0.382363922	0.754556206	1
MOB3A	0.346316146	0.812715232	1
DPH6	1.52848248	0.331273653	1
HSD11B1L	1.068016693	0.201181951	1
HARBI1	0.325771553	0.72666735	1
FAM180A	-2.849305937	0.147882476	1
SUCO	3.563793241	0.159114876	1
FJX1	0.042662604	0.960230492	1
SLMAP	3.036816148	0.122325685	1
THAP1	-0.333640006	0.710911268	1
DNAAF8	-0.753860867	0.328719328	1
CIDEC	2.886473531	0.094723236	1
EPDR1	-0.25177442	0.840706784	1
WNK1	0.213089292	0.899321589	1
NCS1	1.376341266	0.249496315	1
FBXL8	0.220144861	0.853543883	1
C11H2ORF68	1.01658498	0.448714869	1
CCDC85A	-0.938738913	0.483072136	1
DFFB	0.069315587	0.961075985	1
ENSBTAG00000021531	-0.314778137	0.82313513	1
ELP4	-0.211778618	0.831402163	1
RPUSD1	0.129379381	0.936117273	1
RAD54B	1.024753406	0.410459153	1
PUS10	0.238832294	0.823105562	1
TMEM25	-0.415825781	0.728769977	1
ENSBTAG00000001773	-0.698742536	0.360255702	1
EBF3	-1.684082988	0.308706736	1
MBTPS2	0.721579974	0.575365156	1
ARHGAP44	-0.977339237	0.468111494	1

BRCC3	-0.362718863	0.719458289	1
PSTK	-0.435056558	0.661226355	1
ELF2	0.099741165	0.950214347	1
GAS7	-0.077081927	0.943204652	1
IGF1	2.380500544	0.102461927	1
RASGRP1	-0.619417825	0.658175841	1
ENSBTAG00000026408	0.356212483	0.777807802	1
GNAO1	1.979080327	0.206050365	1
TYW3	-0.209705961	0.892432586	1
C14H8ORF76	0.333250814	0.782411296	1
ZNRF2	0.679294966	0.429482923	1
ARHGEF4	2.381085108	0.104873064	1
YRDC	1.411560077	0.291561776	1
F13A1	-0.664616965	0.638653607	1
SERPINE1	-0.460863211	0.733301438	1
WARS2	1.581183456	0.240422471	1
GGCT	-0.383032274	0.706150975	1
GABRR1	2.549744886	0.218798307	1
HSD17B6	0	1	1
AGAP1	-0.641220364	0.665245749	1
PLSCR4	-0.010655468	0.994021123	1
CAMKK2	0.708681552	0.737274624	1
WDR59	0.13218841	0.92782716	1
TEAD1	-0.855937319	0.629775753	1
FAM199X	0.169187135	0.916310331	1
G18172	0.820296567	0.347882375	1
ENTPD5	0.015717082	0.990798365	1
CDC42EP2	-0.339790081	0.767002817	1
SLC5A1	5.361545966	0.19087585	1
ARHGEF3	-0.465384246	0.631158094	1
SNRPE	0.3217104	0.811628104	1
UFSP1	-0.156006271	0.874742834	1
LOC512464	1.159110047	0.491814376	1
ATXN7L1	-0.044219131	0.95633842	1
FAM149A	1.483257635	0.342833021	1
WNT3	0	1	1
SLC39A2	1.178843645	0.400296581	1
RALGPS1	-0.049153069	0.972537971	1
TRIB2	-0.832430276	0.553528703	1
RGS3	0.589057653	0.736196023	1
TRIM56	0.145916473	0.924460571	1
TAB3	-0.518774731	0.707191831	1
CBY1	-0.015398426	0.989258254	1
MAFF	-0.930209198	0.387032564	1
GFOD2	-0.00372238	0.997600107	1
CBX8	1.028818944	0.296801693	1
CDCA3	3.239101055	0.130440355	1
NFKBIE	-0.17850158	0.887691539	1
DSE	0.240607086	0.870374379	1
G36620	2.089659219	0.255275981	1
OSGEPL1	0.430831112	0.756687889	1
WDR4	0.524383697	0.562996109	1
EFHC1	-0.153940381	0.886145887	1
HPN	0.548464228	0.594100054	1
MAGI3	0.403024901	0.833336083	1
FAM91A1	1.129356361	0.439427245	1
KNOP1	-0.001205918	0.999020892	1
DLG4	0.111002591	0.910977564	1

MYB	1.56362009	0.360814437	1
EFNB1	-0.184492399	0.897264763	1
ENSMMSG00000023877	-0.30453005	0.83167848	1
DCLK1	1.029638852	0.602956287	1
ENSBTAG00000052058	-0.16566225	0.88131955	1
FAM168A	0.090099552	0.953486023	1
NSD3	0.120884298	0.934483147	1
HUNK	-0.644705526	0.661830329	1
TCEANC2	0.966061286	0.506497565	1
STX6	0.849613083	0.447426852	1
RYBP	1.154276219	0.347895735	1
GJA5	-0.355288944	0.705606862	1
DMAC2L	-0.716665804	0.526671849	1
PRDM1	0.871931837	0.418969796	1
FGL1	-0.116897577	0.927601786	1
ZNF235	1.372928736	0.430901089	1
RAB2B	0.667071977	0.535396143	1
ZBTB7C	0.17445932	0.88681329	1
ICAM1	-0.483714857	0.6166169	1
ENSMMSG00000015771	0.826501507	0.595618666	1
ACER3	0.640650223	0.582226205	1
KIAA0586	0.254804284	0.837720832	1
IGF2	-0.124807023	0.887701378	1
HIC1	-0.584974043	0.444875281	1
C8H9ORF72	-0.397968869	0.65642419	1
B4GALT1	1.307575702	0.590520956	1
ATE1	-0.282478375	0.732518672	1
MRPL1	-0.63825971	0.530978572	1
FAM219B	0.430670221	0.658256335	1
NUF2	0.803050879	0.418588951	1
RBM27	0.364244139	0.822594763	1
CBL	-0.051742973	0.974603177	1
SEC16B	0	1	1
KLHL7	-0.290127981	0.835218013	1
ANKRD13B	-0.894934386	0.291667684	1
SNAI2	-0.409206506	0.703262359	1
TMEM92	-1.80787908	0.685528273	1
KLHL26	-0.434253768	0.777466373	1
COX19	-0.480706457	0.592830789	1
GLYR1	1.217144823	0.433803406	1
ZNF507	-0.251499617	0.855311027	1
TMEM68	0.875641003	0.569680053	1
TBC1D8B	0.687154578	0.654557066	1
VRK1	-0.060475093	0.951013518	1
KLHL18	0.753463434	0.712622078	1
DOCK11	-0.694805138	0.639241215	1
ZBED2	0	1	1
GPR146	-0.721965479	0.541585701	1
HDAC9	-1.013171877	0.66937358	1
L1CAM	0.347349688	0.818871762	1
CCDC171	-0.282078867	0.845863074	1
CCNE2	0.356984073	0.830242377	1
ZMYND15	1.724105305	0.439203709	1
AGBL1	-1.30881162	0.482744834	1
ABCD4	-0.204962133	0.858713152	1
TRIM25	-0.577271364	0.678447909	1
NTN4	-0.917833477	0.308299442	1
MELTF	0.026874874	0.982099219	1

WDR25	0.446835584	0.696487652	1
PACC1	-0.477892245	0.562959737	1
REST	0.709284581	0.454458038	1
PCDHGA9	1.412013087	0.345212734	1
DNM1	0.4181456	0.716282813	1
ZNF148	-0.035858771	0.97998242	1
CHST15	0.120948322	0.929206021	1
SMIM19	-0.409546928	0.659944718	1
ARHGAP23	3.945871419	0.34427189	1
ZDHHC24	1.058580054	0.287938767	1
APOL3	2.242530578	0.111586304	1
TTC9C	0.569591328	0.728752012	1
SMYD5	1.248017748	0.379375922	1
PCBD1	0.906914787	0.526255879	1
COG8	-0.243157594	0.879507291	1
CRKL	-0.708027647	0.522258091	1
MAPK8	0.143719645	0.925850465	1
FRMD3	-0.645915475	0.671712762	1
PBDC1	-0.335887394	0.686834225	1
PML	1.601635075	0.220429671	1
ENSMMSG00000022035	0.771284725	0.57218918	1
ATG4A	-0.245352135	0.864569702	1
NOL4L	0.296560429	0.838938423	1
RPAIN	0.042159867	0.965082968	1
SLCO2B1	0.090779144	0.960739358	1
MOSPD1	0.696198133	0.618707843	1
GJA4	-0.907116123	0.496858152	1
G12418	0.426031006	0.807058172	1
SELENOI	1.082660898	0.299711591	1
SLC15A3	1.047882496	0.339881976	1
GK	-0.382214855	0.707515747	1
FAM136A	-0.078526201	0.955719625	1
C25H7ORF50	0.520271259	0.549652532	1
FBXL15	0.75344894	0.501758047	1
VPS13A	0.481999262	0.810798233	1
RBM48	0.67193983	0.59025633	1
TRIM47	0.404351805	0.81012886	1
LCLAT1	1.23958413	0.467616039	1
TYW5	0.707227559	0.67225528	1
CCDC103	-1.828396299	0.413114632	1
FOXP1	-0.603809798	0.622695095	1
ESM1	1.995124266	0.23305003	1
G6352	0.016511775	0.986933632	1
KIF15	2.193077968	0.223254987	1
HLX	0.754298427	0.4977914	1
NSMCE2	0.360954698	0.735795177	1
LMAN2L	2.154446781	0.302760076	1
MAD1L1	-0.055021983	0.955931647	1
METTL7B	-0.462939411	0.719909604	1
TNFRSF19	0.424977199	0.774854605	1
TERF2	0.775445818	0.639185703	1
ALS2	0.400820252	0.822315873	1
SEMA3G	-0.398974415	0.77377584	1
ZNF654	-0.31107759	0.751279255	1
MGMT	2.181386783	0.086432384	1
NUDT16	0.113169933	0.916005994	1
KNTC1	-0.551443645	0.673175225	1
VTI1A	-0.381077121	0.789816775	1

TGM3	0	1	1
TNFAIP3	-0.153964465	0.84835273	1
MORN4	-0.35588752	0.805710348	1
ZSCAN25	-0.01118504	0.992434127	1
WDR37	0.321871222	0.766744881	1
ELF2	0.346556462	0.838859634	1
SPRYD4	-0.294167193	0.724629601	1
PFKP	-0.389263283	0.720814943	1
NCOA7	0.584973658	0.678996204	1
EDC3	0.146533542	0.882946335	1
HOXC9	-0.861486965	0.362458871	1
DCP2	0.445235612	0.670167803	1
RASAL2	-0.364221092	0.752295488	1
LPAR1	-0.825453933	0.421460978	1
LRRC28	-0.212331805	0.899216231	1
CEP164	0.948978171	0.40221904	1
SDK2	0.521734072	0.726974671	1
TECPR1	-0.683306628	0.421209673	1
KIF11	3.795650228	0.088371087	1
CDNF	-0.568907092	0.699542691	1
TBC1D32	0.57243648	0.710190058	1
PHF14	0.818628898	0.536718409	1
NUDT13	0.839195435	0.620547005	1
VPS37C	-0.100726818	0.936094296	1
YEATS4	0.290467339	0.801955769	1
PRR22	1.489803635	0.221531098	1
NRBF2	1.082729098	0.409218105	1
RGL1	-0.525110276	0.612221415	1
CCDC138	-0.128421469	0.901662599	1
TRMT44	0.428293208	0.829591682	1
PTRH1	1.780434727	0.204760471	1
SUDS3	0.743126765	0.548970325	1
ABCA6	0.691009466	0.769611396	1
ENSBTAG00000030322	3.590631741	0.113417867	1
NFATC2	-0.233670459	0.877669007	1
PPIC	-0.510957331	0.606970308	1
ENSMMSG00000014745	0.994489085	0.495044459	1
LRRC73	3.56941318	0.113348023	1
ZFYVE21	-0.402838984	0.694946187	1
SH3RF1	-0.554906176	0.684738649	1
ZNF12	-0.078982575	0.959880174	1
KBTBD2	-0.189806187	0.904428718	1
TMEM88	-0.515517758	0.628323884	1
NPR2	-0.776701709	0.668499121	1
CHD1L	0.463455471	0.765074051	1
GLI4	0.622581086	0.574316471	1
RCAN3	0.562499375	0.652175154	1
BANP	-0.143452133	0.9233192	1
ZSWIM6	-0.703134142	0.462077218	1
AP5M1	0.4504475	0.66423323	1
HYAL1	-0.670057148	0.568465268	1
DPM2	1.600871662	0.14455851	1
TPMT	-0.448400692	0.607858441	1
CX3CL1	0.754910536	0.462564526	1
JDP2	-0.002613895	0.997392357	1
ENSMMSG00000010403	1.049275635	0.453295806	1
GAL3ST2	-1.825358126	0.43727267	1
PRKRA	0.219536852	0.895081449	1

FOXR1	0.271734686	0.826289103	1
CREB1	0.047993742	0.980180639	1
SYNE1	0.065181087	0.968567742	1
FLVCR1	-0.026752212	0.985225545	1
AMMECR1L	0.237101988	0.864606972	1
TAC1	0	1	1
SIRT4	0.435827351	0.80110953	1
COA5	-0.429835263	0.711380602	1
WFDC5	0	1	1
TSPAN18	-0.90454225	0.531100265	1
CCDC134	1.459075109	0.27296685	1
MEF2B	1.968455572	0.149675413	1
MMACHC	0.766526992	0.486529332	1
LOC112445144	-0.36608373	0.709904449	1
ARL10	2.352404563	0.160368775	1
PAQR4	0.126775054	0.893458021	1
SLCO2B1	-0.824805372	0.725194721	1
SAMD8	-0.589720922	0.692681991	1
MCRIP2	0.422839516	0.736590048	1
LIG4	0.517611299	0.678227996	1
MCF2L	2.10853816	0.13205721	1
KIAA1109	1.221203027	0.37690803	1
PSMD10	0.376023673	0.695039378	1
APOA1	1.790021588	0.20556426	1
REEP1	-0.952023583	0.432592409	1
ZNF75A	0.05718231	0.968460162	1
CFAP57	0	1	1
SRCAP	0.150806832	0.887151841	1
EYA3	0.248064381	0.879479776	1
ENSMMSG00000008586	0.709155074	0.678366096	1
KIF22	0.919192866	0.480860995	1
KIFC1	1.487966078	0.259589987	1
SLC4A3	1.193571554	0.376473679	1
CD177	0	1	1
INSIG2	0.211765443	0.885325171	1
APBB3	0.123089534	0.895058509	1
SOCS7	-0.504993919	0.624318574	1
IRF1	-0.706957282	0.608288991	1
SLC7A10	-1.120633955	0.353513241	1
HSPB11	0.50142636	0.668488887	1
PUS1	-0.677170958	0.63448134	1
SNX10	1.495447414	0.271775061	1
LRRC14	-0.160967653	0.843894806	1
TRPV5	0	1	1
TMEM185B	-0.570057874	0.678871746	1
ADA	-0.79681787	0.359065169	1
ENSMMSG00000006456	0.033999539	0.971568119	1
PDSS2	0.381858732	0.811423244	1
CEP128	-0.360244243	0.748860019	1
DOK1	-0.145661058	0.891572047	1
DHRS12	-0.113146492	0.92345799	1
DKK3	0.865102872	0.513716322	1
ZNF628	0.582464483	0.623524024	1
NACA	-0.753862219	0.588983708	1
MINDY2	0.290215971	0.844476181	1
ARID2	0.050942283	0.974091381	1
CFAP97	1.168843434	0.492387898	1
GTPBP8	-0.653335075	0.663976917	1

ZBTB39	0.153716755	0.866309828	1
CCL14	0.260161805	0.848351426	1
METTL16	0.099123059	0.948280643	1
EBF1	-0.388697906	0.804370855	1
LPAR3	-0.523138838	0.579558856	1
PLBD2	0.029396021	0.982272319	1
LYRM1	0.07406136	0.961126655	1
WDR44	-0.413823162	0.779990899	1
ARMCX3	0.69798022	0.563484723	1
NRAP	-0.051707031	0.97744429	1
MEGF6	1.761278885	0.177626076	1
SCPEP1	-0.253914405	0.823067466	1
ZNF165	-0.322339217	0.747301711	1
GRB10	-0.791084789	0.591702782	1
HABP4	0.186008211	0.907158592	1
NRP2	-0.685018661	0.634457245	1
FEM1B	-0.759027796	0.573149078	1
MAFB	1.427294222	0.409578662	1
SMCO1	-0.906371935	0.495741359	1
KLF13	-0.159807771	0.917704643	1
CHST15	0.346592549	0.700129798	1
RDM1	0	1	1
SEPSECS	1.410919554	0.404414215	1
BMPR1B	-0.020559501	0.990043385	1
LAG3	-1.807873791	0.685633208	1
OCIAD2	0.357160094	0.833423094	1
TOR2A	2.037131915	0.136783682	1
TMEM128	1.257236659	0.340507771	1
PLD2	0.17557856	0.898869017	1
EMCN	-0.769006404	0.596501961	1
DCUN1D4	1.516198852	0.354911535	1
SAC3D1	0.229704142	0.838829258	1
ZNF217	0.303609987	0.817660409	1
CITED2	1.984209853	0.123751853	1
PLAT	-1.087498678	0.449989496	1
MMP11	0.352216556	0.781997285	1
COL21A1	1.189518716	0.50252972	1
PTPRG	-0.639197395	0.741793548	1
PDE6D	-0.375651185	0.795065361	1
MASP1	-0.03418741	0.984735859	1
PRKG1	0.322399156	0.846612968	1
PLXNA3	0.281725018	0.774541096	1
DVL2	2.26594363	0.232540623	1
PTGS1	-0.01928907	0.984381626	1
RACGAP1	-0.76359893	0.493029378	1
E2F1	1.806331163	0.08671017	1
DOP1A	0.815108823	0.68675209	1
MARS2	-0.064407665	0.965229219	1
ALG10	0.403795594	0.793623972	1
PEAR1	-0.063530509	0.965367603	1
PDCD7	0.046976545	0.963564749	1
TIMM10B	0.709610999	0.495774974	1
CRADD	-0.397397394	0.742046356	1
PTPN12	-0.066098246	0.964407012	1
CEP85L	-0.599027201	0.749128733	1
FAM126B	0.909518792	0.608870851	1
IFT140	1.194409304	0.403267843	1
KIF26A	-0.985233461	0.521904069	1

TIA1	1.704822353	0.31346221	1
TYMS	0.602495227	0.58609864	1
SLC2A12	-0.70148376	0.411849832	1
UGGT2	-0.442391259	0.686567395	1
EGR3	-0.645458201	0.541248261	1
KLKB1	-0.136554162	0.928538389	1
ZKSCAN8	1.255732314	0.433172215	1
TRIM37	0.616099135	0.516249241	1
MSANTD3	-1.089652273	0.32290667	1
ENSMMSG00000015182	0.551361718	0.722201686	1
LOC789384	0.400900902	0.721119244	1
AGBL5	1.462596017	0.300025544	1
AIF1	0.402023024	0.750720018	1
SSX2IP	-0.756587641	0.584597898	1
ZBTB43	0.88999266	0.527883357	1
TP73	1.526874172	0.507532482	1
DZIP1	0.023065921	0.981169963	1
MTAP	0.617769863	0.702925257	1
EPB41L3	0.640253787	0.673490021	1
FUT8	0.016717727	0.990639113	1
PLOD2	-0.448982247	0.757846351	1
PSMD10	2.17175236	0.194216685	1
GCNT2	0.497236161	0.745637878	1
ADAP2	0.271822363	0.84858577	1
GFAP	-2.849539604	0.152585048	1
G18209	1.018213565	0.467788843	1
TBC1D30	1.016351293	0.399488968	1
GAREM1	2.194143008	0.164404391	1
CCL8	-2.850027372	0.163137791	1
NYAP1	2.311012544	0.120133406	1
CCDC120	1.777834202	0.388908123	1
PHF19	0.289050468	0.799396429	1
INPP4B	-0.560986637	0.523114461	1
ITPRIPL1	-0.399418537	0.71122059	1
/H2AC13/H2AC16/H2AC17/	2.584240727	0.161919016	1
PALD1	-0.285651356	0.85282141	1
C15H11ORF49	2.250404415	0.109318881	1
HFE	0.856620019	0.444726694	1
CD68	0.154799449	0.892680258	1
PRPS2	1.050327846	0.520098707	1
TAPBPL	2.65091336	0.087503067	1
WASF3	1.37091018	0.404926932	1
ARHGAP42	0.622172156	0.683016434	1
PTGER4	-0.765798808	0.466214776	1
G10414	0	1	1
TBC1D25	-0.619098837	0.538981763	1
LYRM4	0.499164439	0.601871908	1
NCOA7	0.536536646	0.714757761	1
ITGAM	-0.45146614	0.770444081	1
KIF7	0.310324125	0.731594568	1
FIGNL1	1.082278967	0.340606118	1
ITGB2	-0.051545617	0.963509593	1
FBXO34	1.026684768	0.471938256	1
C10H15ORF61	-0.391268273	0.728218561	1
CDC123	-0.376829984	0.849065369	1
TIMM9	0.403077642	0.732847091	1
ALKBH1	-0.621235531	0.663005654	1
MYT1	2.620973611	0.298771387	1

STOML1	1.60753715	0.236051303	1
STOM	-0.333069375	0.739793009	1
RPL26	-0.253435423	0.778173617	1
IFT43	0.840052342	0.496088348	1
DCDC2B	1.233257428	0.353731841	1
SLC6A6	0.683631961	0.622886328	1
ZKSCAN5	-0.379391068	0.81136685	1
MYO1F	0.07140915	0.935647503	1
BIVM	-0.071872199	0.962527798	1
SOX13	-0.496660167	0.616763632	1
HLF	-0.191610549	0.904733735	1
PAMR1	-0.218003346	0.884729421	1
POSTN	-0.032714856	0.984865724	1
P2RY1	-1.137508991	0.302620078	1
MTHFD2	-0.052633631	0.967993525	1
NXT1	1.126998407	0.377305127	1
SLC39A14	-0.873652922	0.559089723	1
ALG13	-0.622713932	0.662385813	1
CPSF7	1.524000094	0.280949562	1
PUSL1	1.461002648	0.243517092	1
CABLES2	-0.173157277	0.908195383	1
G8426	0.121867936	0.939490257	1
G18055	0.074080082	0.935111217	1
CFH	-0.066530228	0.965392436	1
TSHZ3	0.200954254	0.795559221	1
UBE3D	-0.654452469	0.561369801	1
GAL3ST4	0.801969293	0.488069722	1
MAN1A1	-0.251909355	0.80292613	1
GOSR1	-0.351562652	0.807278441	1
RAB33B	0.524110852	0.750832555	1
ADCK1	-0.27659718	0.790774151	1
ENSMMSG00000011324	1.71953891	0.232282481	1
MPZ	-0.732695582	0.62059955	1
SKIL	-0.656806169	0.642598826	1
G33485	1.605687157	0.146703962	1
IFITM5	0	1	1
BIN3	1.147886121	0.411004914	1
MIPOL1	-0.044561903	0.975840426	1
ADAMTS5	-0.388668887	0.784363202	1
MAFK	-0.549704941	0.669437614	1
C11H2ORF42	-0.06987659	0.962326281	1
IRAG1	-0.855817461	0.639213777	1
TATDN3	-0.310662146	0.830503556	1
BBS7	0.377061917	0.71274895	1
TRDMT1	0.020632017	0.985319267	1
PRKD1	-0.397838437	0.773262008	1
CHST12	-0.216816383	0.780933744	1
SFXN3	0.492387813	0.596821843	1
FUZ	-0.773245019	0.38819432	1
PTER	-0.381290541	0.799721907	1
PLPPR3	0.049694859	0.962020331	1
ZBTB14	0.005218869	0.99697258	1
XPO4	0.191378777	0.897244141	1
E2F5	0.025504539	0.980579433	1
TMEM86B	1.300286674	0.237750851	1
IPP	-0.591098767	0.575982289	1
ATXN7L1	0.329725842	0.745931478	1
POC5	-0.152005761	0.854236145	1

RET	-0.970811432	0.324693199	1
TMEM64	-0.140275867	0.879003635	1
SPTY2D1	0.134416677	0.92903717	1
IGF2BP2	-0.36825291	0.835323331	1
GNAZ	-0.841245744	0.548524368	1
PDE5A	-0.734630581	0.574763415	1
DNAJC30	0.313429532	0.732031279	1
BORCS6	-0.336944193	0.8022319	1
FAM181B	-2.849913516	0.160585347	1
TSNARE1	0.566243073	0.791265766	1
MGST2	0.342524821	0.838087155	1
CRTC2	-0.067453023	0.935768169	1
PID1	0.989404838	0.474623191	1
HOXD3	0.317786008	0.829027516	1
BCL3	-0.178990795	0.876100258	1
GALNT18	-0.554889309	0.58260077	1
CETN2	-0.846106911	0.490095507	1
BRSK1	0.595714078	0.568332494	1
EFCAB2	-0.942141811	0.298273127	1
PRKCE	-0.601348763	0.706268006	1
EVC	0.121217689	0.934059712	1
TERF1	-0.879700931	0.522934794	1
ENSMMSG00000005228	0.99406612	0.692786168	1
POMT2	-0.093393509	0.935048127	1
CRYGS	0.049327761	0.959420804	1
RPUSD3	-0.576462974	0.537838485	1
PLCG2	0.517438074	0.578708024	1
TRIAP1	-0.216463191	0.807206267	1
PABIR1	-0.609573864	0.551210314	1
G33742	0.582103465	0.672199014	1
EFCAB11	-0.147570895	0.919525189	1
FEN1	-0.000928485	0.99935743	1
FBXO11	-0.490303109	0.742296909	1
CLXN	2.905301556	0.090898938	1
MYO18A	0.872237756	0.49221897	1
DOCK10	-0.219497845	0.876499929	1
ACTR3B	-1.053529013	0.463634443	1
ZRANB3	-0.134580637	0.925595876	1
SLC4A4	-0.450369617	0.740608092	1
KRAS	-0.301583434	0.764075769	1
DCUN1D3	0.189597681	0.828383322	1
FOXN2	0.557553657	0.749394084	1
DUSP15	0.74525677	0.5938417	1
SMUG1	0.059649549	0.957725716	1
ASB13	-0.55912344	0.701017312	1
MSI2	-0.217337774	0.882993564	1
ADCY7	-0.528037741	0.56296724	1
SGSM2	-0.297585169	0.776595921	1
YIPF1	0.991444004	0.404328047	1
FGD1	0.858827068	0.505168485	1
NPAT	0.793164882	0.543909623	1
TBC1D22A	0.034781699	0.968486893	1
G33895	1.049504925	0.432696952	1
HBEGF	0.312818347	0.858233485	1
NTAQ1	0.819226255	0.427949981	1
FGFR1OP2	0.120124222	0.932952228	1
C7H19ORF25	1.475775147	0.312073502	1
TMEM203	-0.223281783	0.828101239	1

PLAC9	0.527125353	0.654192307	1
SP2	0.007654106	0.996246388	1
FAM13B	2.972822047	0.264878095	1
SPNS2	-0.801142476	0.460421073	1
TRIM9	3.138044966	0.127480935	1
FRMD6	1.27388701	0.509941839	1
CINP	0.197891104	0.814180685	1
G18528	0.722678487	0.615522684	1
RPUSD2	0.609675203	0.521291628	1
ZDHHC16	-0.456503285	0.763736877	1
TIMP4	-0.954885329	0.494808054	1
G36132	-0.363182509	0.799610954	1
OLFML2A	-0.543372912	0.727232211	1
IL17A	0.074550158	0.959792219	1
MMS19	-0.051787257	0.968651744	1
FNDC3A	3.18399508	0.184595499	1
CDADC1	0.517534937	0.692524292	1
PCDHGB5	0.507794257	0.67574	1
PRG4	-0.727321775	0.621341304	1
TIMELESS	-0.312373831	0.759047043	1
HGH1	0.134991325	0.886731523	1
GAREM1	0.329240073	0.93707355	1
CCDC117	-0.386377095	0.700381751	1
TTC39B	0.998939337	0.31062289	1
MED13	-0.672986154	0.641357918	1
TNFRSF1B	-0.224736781	0.804296371	1
MPP2	2.980257867	0.10975493	1
ITPRIP	0.005459446	0.997109054	1
SEC22A	1.491271882	0.249699004	1
GRIN2C	-1.827375849	0.420869878	1
ERCC6L2	-0.065312523	0.944648384	1
ARHGEF10	0.126398362	0.916208065	1
RELB	-0.179006048	0.859826588	1
AKIRIN1	-0.321098446	0.823308187	1
ENSMMSG00000006119	0.317223038	0.788736597	1
SPATA13	0.714183804	0.653602334	1
PRSS55	0	1	1
ENSMMSG00000020634	4.454059224	0.109677962	1
SIRPA	-0.694078593	0.645485911	1
C3H1ORF52	-0.535183732	0.569308924	1
IFT57	1.059208602	0.440339735	1
SH2D3A	1.44791099	0.26474474	1
PEX12	1.399822936	0.305113406	1
C14H8ORF34	0	1	1
ANKRD33B	-0.289410031	0.861631282	1
ENSMMSG00000013018	1.291434142	0.367758304	1
VAMP4	0.132972056	0.879803285	1
FREM1	1.017011731	0.585871112	1
MEOX2	-0.87058828	0.500710731	1
DGKH	0.563505238	0.567064393	1
GDAP1	0.053254434	0.973511638	1
GLB1L	0.962394277	0.39965887	1
SLC38A9	0.241643355	0.807023855	1
ZCCHC10	0.445447432	0.666293421	1
AFAP1L2	-0.793553927	0.334713838	1
TATDN3	1.037027525	0.517545362	1
ENSBTAG00000047133	0.537798201	0.592370304	1
RFXANK	1.167450552	0.37703138	1

CORO7	-0.223429226	0.825159009	1
R3HCC1L	-0.55235845	0.680344232	1
ENSMMSG00000009616	-0.380332294	0.795819064	1
CDKN1C	0.232602217	0.82712979	1
GTPBP10	0.732006531	0.640562864	1
ENSMMSG00000022941	1.567233538	0.283125534	1
EXTL1	0.501288683	0.764753415	1
TMEM192	0.96450267	0.53491459	1
ARHGAP23	-0.654959654	0.614068302	1
ENSMMSG00000005917	-0.346341862	0.81765911	1
C1H3ORF38	0.026417076	0.981789777	1
SYT2	0	1	1
CARS2	-0.267988182	0.868557546	1
SYDE1	-0.547771924	0.586244551	1
MDM4	0.441715577	0.696626805	1
ADAMDEC1	1.143061682	0.583114532	1
SH2D3C	-0.7148873	0.349052408	1
RTL6	-0.016228426	0.990832759	1
GALNT15	-0.114640405	0.942454904	1
PHC3	1.133675666	0.416101701	1
NFYA	0.838506773	0.593206326	1
GFPT2	-0.904707191	0.328551687	1
GAR1	0.292641612	0.784142851	1
WDR62	1.861713907	0.124840321	1
MRGPRF	0.698787069	0.625787771	1
TPPP	0.033923207	0.975376417	1
LMOD1	-0.526660872	0.746018636	1
IGFBP3	0.798385134	0.431983391	1
TTLL5	1.723118888	0.47206469	1
GPR157	-0.788840558	0.582606489	1
FAM184A	-0.402124993	0.714160522	1
G1069	-0.394267713	0.778016714	1
MAST3	0	1	1
FAM184B	0.72743232	0.66022276	1
ZFAND2A	1.027031296	0.46994464	1
SASS6	1.154907686	0.509961562	1
MITD1	1.19737744	0.364187773	1
SOX18	-0.662747452	0.473059133	1
ELMOD2	1.374041407	0.377152682	1
NMU	0	1	1
COA7	-0.856041967	0.452156879	1
NREP	-0.910945706	0.406480265	1
KMT5C	1.371729694	0.264743119	1
TFDP2	-0.754122133	0.595411581	1
TBCEL	2.061197076	0.224891673	1
ISL2	0	1	1
RAD1	-0.064480088	0.953421333	1
METTL6	0.190840241	0.865936119	1
CNKSR3	-0.222909477	0.770189576	1
ATAT1	-0.104580446	0.911554934	1
DAGLA	-0.720058968	0.529870638	1
INPP5F	0.459885324	0.71852509	1
KLHL11	0.520686846	0.526812223	1
TIMM29	-0.12043527	0.925708494	1
COA4	-0.463688503	0.677841034	1
CNTNAP1	0.554767923	0.549511859	1
NR3C2	0.383081832	0.825631313	1
ENSMMSG00000006232	1.069740934	0.505260103	1

FAM114A1	0.079497946	0.962159263	1
G17697	-0.290617142	0.792877052	1
ZSWIM1	0.890580367	0.532435648	1
KDM8	-0.713928311	0.615952773	1
RADIL	0.08661807	0.941792682	1
SLPI	0.839524827	0.686957294	1
ADARB1	-0.2133942	0.885989175	1
LOC112445031	-1.128778809	0.312150699	1
ENSBTAG00000049478	-0.637126672	0.65705453	1
NHLRC3	0.604313286	0.688082669	1
CYBC1	0.327499116	0.766235714	1
FHIP1B	-0.118958768	0.935327516	1
FGD5	-0.643472145	0.660975596	1
ITGA10	1.170881733	0.256626924	1
MAD2L1BP	-0.580655754	0.614181458	1
TEAD1	-1.280077641	0.445977012	1
G3214	0.048641015	0.962752838	1
CCDC91	0.023507175	0.979737836	1
G21294	-0.455767225	0.769437984	1
INCENP	-0.410358871	0.77549146	1
KCTD18	0.125408512	0.939250028	1
CFLAR	1.490258354	0.362087834	1
NAB1	0.06834881	0.966187944	1
GRIP2	-0.443854222	0.788797975	1
RNF6	-0.331455848	0.817265427	1
VEGFA	0.714981963	0.522954711	1
GNAQ	-0.316164828	0.758310207	1
TMEM104	0.273813636	0.858449769	1
SLC23A2	0.385106325	0.692884219	1
LOC505479	2.362774738	0.231564874	1
METTL17	0.282914078	0.855315867	1
MANBA	1.058004309	0.530687489	1
MAGEL2	0	1	1
TRAPPC9	0.602717755	0.626318417	1
TPST2	-0.103860984	0.907866666	1
LAMA2	-0.529341562	0.668126233	1
YPEL2	0.703411698	0.602124314	1
HK3	0.424354644	0.701535693	1
MSANTD2	-0.13038813	0.896420652	1
DLL3	0	1	1
PLEKHA8	0.191101446	0.85015356	1
ZNF746	0.742455954	0.498198996	1
MCM8	1.171748423	0.364067805	1
NEDD9	-0.281339768	0.797423593	1
SRPX	1.01150554	0.330959772	1
ARPIN	-0.063602289	0.95559535	1
PHACTR2	-0.665370413	0.64592222	1
TTLL10	0	1	1
DLC1	-0.582662912	0.531433742	1
MFSD8	1.087439869	0.496997547	1
FZD9	-0.35172323	0.78625529	1
COPZ2	1.890974283	0.237103111	1
CDKAL1	-0.64607976	0.637701911	1
FBLIM1	-0.354789363	0.805266215	1
FRMD4A	-0.546210917	0.586098532	1
RORA	0.204313895	0.839007271	1
NLRX1	-0.114192533	0.916674039	1
CLHC1	0.66132394	0.692441941	1

CHN1	-0.886547552	0.453942669	1
POLR1H	0.184883574	0.866229065	1
LYST	0.222815521	0.887110907	1
RBM12	0.064677298	0.944157483	1
ADGRD1	-0.328082389	0.831099828	1
ADGRB2	1.856513756	0.146429318	1
C1ORF122	0.856639515	0.454601741	1
SGCD	0.061236689	0.962851097	1
TAMALIN	-0.463799713	0.765793253	1
GLS	1.140525891	0.515472978	1
ELF4	-0.335817288	0.713808509	1
ENSMMSG00000001934	0.57721884	0.607463058	1
GTF2F2	-0.407755193	0.77593918	1
SH2B3	-0.494392845	0.615729701	1
MYXGL	0.238030235	0.868927372	1
PHLDA1	1.067498114	0.336467902	1
HAUS1	-0.473053735	0.541557446	1
NYNRIN	0.388196113	0.64353188	1
NFASC	-0.944520595	0.398822342	1
G4300	0.16201026	0.864248651	1
G22764	-1.915739328	0.514221644	1
TENT5A	0.247003562	0.886651162	1
GSTCD	1.3592942	0.206751061	1
CYBB	0.761953292	0.50012427	1
ANKIB1	0.063724188	0.968587833	1
CPPED1	0.389787943	0.708660615	1
SUPT7L	-0.363575489	0.701470714	1
MTRFR	-0.176198105	0.869541736	1
ENSMMSG00000009595	0.003255833	0.99762074	1
FILIP1L	-1.040670209	0.263187652	1
KANSL2	0.133140977	0.9340065	1
RPS3A	0.854012403	0.458281426	1
ZNF367	1.330973392	0.166330734	1
POLM	-0.519854326	0.616361838	1
AK5	-1.439106616	0.42882227	1
UGCG	0.642283097	0.526611995	1
VMA21	0.428682991	0.776190164	1
CHAF1A	-0.576067788	0.780097625	1
SLC6A4	0.468735703	0.639374368	1
MBD4	6.94E-05	0.99994143	1
DIXDC1	0.253136953	0.801778654	1
MPP4	0.508870681	0.739998475	1
MED30	-0.191323704	0.853537893	1
HSF2	0.579345525	0.744016794	1
ENSMMSG00000014667	1.623088927	0.333077599	1
ATAD2B	-0.085348743	0.954069299	1
KANSL1L	0.864835802	0.602786993	1
MEGF10	0.654299873	0.637666285	1
ZNF205	-0.410804463	0.693947235	1
MCMBP	0.247147669	0.90008845	1
EYA1	-0.96948839	0.510144956	1
MS4A8	-0.506350521	0.605895366	1
TBP	-0.518055325	0.716412081	1
GIT2	-0.467413322	0.743179118	1
SDCCAG8	-0.418396322	0.583651159	1
LDHD	-0.196814947	0.934238188	1
NEK3	0.520213981	0.644916463	1
PPP1R1B	2.083625163	0.082741905	1

VSIG10/WSB2	0.187039016	0.891665669	1
HIVEP3	1.53027996	0.300335221	1
DPH3	-0.042540666	0.962632159	1
KANK4	1.438082695	0.096031636	1
LMBR1	0.369118114	0.680932873	1
PHF5A	-0.001464927	0.998894428	1
FGD5	-0.809140426	0.552591352	1
AZIN2	-2.850377428	0.17145939	1
TMEM199	0.291349787	0.797147702	1
C4H7ORF57	1.054210646	0.305651116	1
GALNT17	-1.226793772	0.318836744	1
PCM1	-0.769334058	0.631177464	1
MOB1B	0.198776216	0.853727759	1
CTSO	0.976191503	0.48844513	1
PGAP6	0.449323928	0.733304548	1
SENP1	0.259740938	0.815049287	1
THG1L	1.217361603	0.358979911	1
PPM1J	-0.84547543	0.459700518	1
RSPH9	1.266101945	0.272577421	1
RPS6KA6	0.061127316	0.959514533	1
CTNNBIP1	1.248110521	0.248465614	1
GRK3	-0.153257043	0.892870885	1
UROC1	0.104455248	0.931861475	1
TNFAIP2	-0.810868795	0.585142579	1
SAV1	-0.349133257	0.814940909	1
LRRK1	0.959606516	0.390323817	1
ARHGAP5	-0.099489684	0.948038006	1
AHSA2	0.412870695	0.656092219	1
CCDC137	1.097724042	0.386533238	1
AGBL2	0.683074861	0.631070811	1
LPCAT4	0.979920091	0.434456315	1
HMCN1	-0.090463576	0.970162853	1
ENSMMSG00000010458	-0.124358741	0.932596957	1
ENSBTAG00000038116	1.082792301	0.261412404	1
GID4	-0.448553564	0.755185542	1
ADAP1	-0.155388785	0.866449153	1
VIRMA	1.071434631	0.397809311	1
DNAJC13	0.038337344	0.971806023	1
MICAL1	-0.469802662	0.778831844	1
TRPM8	0	1	1
SDC3	0.128516251	0.899525399	1
FKBP10	0.103049663	0.91233805	1
MSH3	-0.263777517	0.866617346	1
EDNRA	1.010243269	0.479004049	1
SNIP1	1.196258124	0.487680223	1
FAM193B	0.01276315	0.99453675	1
GPIHBP1	0.557150823	0.649216592	1
RAI2	0.91534608	0.619524987	1
PACSIN1	0.741157623	0.587411541	1
SUPT3H	1.123932123	0.389712226	1
ANKRD54	0.26431082	0.816012738	1
CSGALNACT2	-0.116111387	0.939487037	1
MAOA	-0.238621091	0.877298057	1
AFAP1L1	-0.994658612	0.24957295	1
CCNB1	1.200087636	0.26228357	1
SIK1	-0.395843623	0.79149037	1
FBXL2	0.980066615	0.655771552	1
CEP112	0.030502624	0.982796566	1

TRPC6	1.636465005	0.28715098	1
MRPS30	-0.554548743	0.532368906	1
SUMO3	-0.551003938	0.616083551	1
RBM18	0.588906044	0.6680876	1
CDHR2	0	1	1
GSDMD	1.925600117	0.138967492	1
ARID3A	0.418023033	0.724549731	1
DYNLT2B	0.983063485	0.33912314	1
NRF1	-0.451511426	0.680509017	1
ENSMMSG00000006468	-0.681312706	0.479542757	1
ADCY3	0.029413837	0.974685101	1
PELI3	-1.09216965	0.377033892	1
ENSMMSG00000019259	1.802355355	0.174416479	1
METTL9	1.143366172	0.285646294	1
PRCP	-0.729426413	0.604910203	1
MCM9	1.414730687	0.263951473	1
TGFBR2	0.088658364	0.954918208	1
MYBL2	1.160388711	0.128975413	1
MBTD1	0.898794993	0.669342546	1
SHLD2	0.530730994	0.611976866	1
KLHL29	-1.050541751	0.414907859	1
MYEF2	0.720130804	0.593789115	1
TFB1M	-0.640523184	0.660689406	1
LOC787554	1.348815603	0.275971727	1
RMDN2	1.423409903	0.288845309	1
USP33	0.873108652	0.553739889	1
MUTYH	0.946641037	0.369134683	1
SLC23A2	0.753268622	0.620140794	1
CEP57	1.312573617	0.432886888	1
TERF1	1.312264717	0.315670319	1
NME6	-0.056309593	0.956487079	1
SERAC1	0.438696497	0.779831496	1
TUBB4A	-0.95304267	0.472319772	1
MLH1	1.38356892	0.279704376	1
G37748	0.536881928	0.711909269	1
GRAMD1C	1.205529567	0.239111328	1
CRCP	0.131583289	0.924647179	1
LGI4	0.19356467	0.867016612	1
ADK	0.091983051	0.937492357	1
DYRK3	-0.441853724	0.660524914	1
LOC616903	0.917140382	0.467185973	1
EEF1A1	0.818021343	0.360508045	1
ORC5	0.703710105	0.653390688	1
SLF1	0.415147503	0.777905787	1
ZNF629	-0.007390794	0.995979169	1
SELENOS	0.212057782	0.8476766	1
POLR2K	-0.293129036	0.759550429	1
TMTC2	0.978442559	0.503784937	1
OVCA2	2.567077289	0.261527466	1
EFR3B	3.134916325	0.109377485	1
C4BPA	3.372501091	0.13756701	1
PRPF18	0.96997671	0.594163827	1
CCN3	-0.359352121	0.798486058	1
CD44	2.089619045	0.163559374	1
LSM10	-0.608166746	0.565094612	1
SWT1	0.93968989	0.56802286	1
TPX2	-1.095073029	0.348058341	1
G34590	1.352801454	0.358651281	1

TBC1D19	0.342018107	0.769794977	1
KLHDC8B	-0.867287659	0.274715587	1
ATXN3	1.706373767	0.185799742	1
WBP1	1.437552358	0.389593812	1
CHCHD4	-0.234230377	0.809515171	1
TGFBR3	0.841292344	0.680700456	1
TNIP2	0.359841644	0.717351126	1
ADGRF5	-0.658174772	0.720543764	1
CCNQ	-0.217289457	0.851546582	1
PDIA5	1.274190464	0.266018905	1
VMAC	0.922139863	0.494891536	1
ERICH1	0.369463595	0.741668376	1
ANO10	0.678812367	0.652702926	1
ZMYM3	0.024102184	0.990794628	1
SMPD5	2.205652168	0.272689388	1
UBXN2B	-0.669436763	0.650425438	1
RNF111	0.482536286	0.757445577	1
ZFPM1	0.614714302	0.566905747	1
GCFC2	0.494981187	0.760649358	1
UNC93A	-0.617679574	0.554352329	1
SLC8A3	-0.912095572	0.481121977	1
STARD8	-0.90595974	0.329185747	1
ADGRL1	0.04005871	0.969135306	1
MVB12B	-0.311608881	0.752340696	1
H2BC18	0.466025614	0.681390267	1
ENKD1	0.954240217	0.442341255	1
FYN	-0.672993492	0.476616571	1
PSME4	-0.657108984	0.68575508	1
RAN	-0.323000059	0.722054324	1
MOB3B	0.487431902	0.733472207	1
NKAPD1	-0.241420314	0.802089026	1
CRACD	0.136681014	0.925466426	1
GADD45B	-0.092616076	0.946780474	1
IRX6	0	1	1
BOC	0.025850485	0.989973075	1
SGK3	0.316372694	0.83467827	1
ISYNA1	0.704386591	0.523617441	1
CEP162	0.477682038	0.627065357	1
SH2B2	0.002515167	0.997995852	1
MPLKIP	-0.139410737	0.900391584	1
IPPK	-0.399327394	0.624963067	1
GDPGP1	0.506690734	0.54795617	1
FZD6	-0.126299138	0.882428797	1
GRK6	1.477785638	0.29726121	1
NEIL1	0.222023772	0.831632133	1
BAZ2B	1.07361463	0.450733055	1
MX1	0.121704944	0.926128027	1
JAM3	-0.303881496	0.774229826	1
BBX	1.079231514	0.577208967	1
RBM3	-0.296931638	0.768065004	1
CLDN5	-0.689569515	0.453643189	1
C21H15ORF40	0.602516531	0.577094482	1
KCTD17	1.965542142	0.295305598	1
LIN54	0.616708285	0.70571939	1
NAF1	0.915848205	0.474141344	1
EYA1	-0.176205864	0.922596396	1
OSBPL6	-0.713558594	0.648384651	1
CDKAL1	-0.398418445	0.706101255	1

TATDN2	0.846978032	0.606941269	1
SLC8A1	1.745257578	0.109363439	1
AARSD1	-0.243602916	0.875462076	1
ARMC9	0.255709935	0.810251441	1
PLXDC1	-0.710081833	0.641712166	1
PCDH7	1.015451065	0.401198615	1
FSCN1	-0.773953039	0.431604614	1
S100A13	1.332107551	0.32260225	1
EXD2	-0.696965597	0.632908213	1
NAT14	0.593295959	0.660599842	1
CSRNP2	-0.044294095	0.969480651	1
TCF12	1.202411858	0.424973481	1
MRPL48	-0.485191225	0.57217701	1
UNG/ACACB	0.070037348	0.963936223	1
LMAN2L	-0.905131255	0.508909953	1
RBMS1	-0.359984733	0.807708549	1
SAV1	-0.37924352	0.797027974	1
VANGL1	1.024233791	0.393154048	1
KDM7A	-0.046900323	0.968905936	1
SOX6	-1.336391513	0.438950581	1
CCDC51	0.726829035	0.565735987	1
WDR91	0.700564547	0.663648848	1
PGBD2	1.311849098	0.34218348	1
BORCS8	0.036643086	0.963989099	1
SYT7	-0.730223305	0.586677636	1
CTNS	0.36418661	0.726101049	1
ZBTB21	0.102670626	0.929742427	1
TUBG2	0.510989153	0.650691346	1
CEP43	-0.525223225	0.594889655	1
GXYLT1	1.589501527	0.21260969	1
SLC39A6	-0.242687876	0.819118156	1
HSD17B12	1.619242905	0.131476292	1
MYCBP2	1.036082093	0.421954075	1
GIGYF2	-0.245893508	0.808524045	1
PARP10	1.311332166	0.201467023	1
PLEKHO2	-0.870767106	0.541158775	1
KRT25	0	1	1
ZFHX4	-0.583926905	0.693555003	1
MAGI3	0.614762851	0.714323139	1
RIDA	-0.343619382	0.720617217	1
JADE3	1.29927252	0.439384339	1
SMC4	1.271227208	0.320105908	1
CDR2	-0.313837797	0.82962647	1
IGSF3	0.385677115	0.693125728	1
MLLT10	-0.442544453	0.756425671	1
SLC2A11	-1.063726545	0.421292133	1
ZBTB24	1.112226855	0.378265935	1
CCDC65	-2.849946386	0.161335039	1
ZCCHC3	0.947235502	0.492761932	1
MTMR12	1.215163925	0.229625536	1
ZNF281	-0.525035613	0.720145932	1
ACOT11	-0.033673779	0.984128806	1
LRFN1	1.078830394	0.521259527	1
BCL2L12	0.669851052	0.596384975	1
NR2C2AP	0.249731218	0.839549598	1
FXYD6	0.156114078	0.862566011	1
IFT27	0.996579432	0.513728889	1
DTYMK	1.935070755	0.135709043	1

DTNA	-0.689783158	0.698043868	1
HECA	-0.006493545	0.996703082	1
ATR	0.421235023	0.700765558	1
PLEKHG2	-0.051188338	0.956821462	1
FLI1	-0.788522023	0.39681212	1
LOC100848815	1.208192741	0.436968756	1
KATNAL1	-0.078445776	0.938654132	1
CEP70	0.707161166	0.535013799	1
SLC66A2	-0.553223958	0.624950476	1
GNPNAT1	0.182563754	0.869468012	1
TDRKH	1.744415189	0.249265838	1
MSH2	-0.368228031	0.690750019	1
CHERP	0.862881725	0.529688163	1
ENSMMSG00000005317	0.12884613	0.932261217	1
USP34	1.0264511	0.447512647	1
PMEPA1	0.461200932	0.655026071	1
MTUS1	-0.721364184	0.506044024	1
DCBLD2	0.451946313	0.727496202	1
ENSMMSG00000012926	0.439925697	0.70163689	1
RSKR	1.159309627	0.370200533	1
CDK19	0.565292759	0.732054443	1
ENSBTAG00000052466	0.17561585	0.869503624	1
NME7	-0.29474937	0.767668191	1
MED23	-1.687339812	0.400469669	1
RFC4	-0.086694642	0.937062767	1
NBL1	-0.892623663	0.32167952	1
C1QTNF1	-0.671235736	0.515571383	1
BCAR3	0.433189827	0.720267953	1
DHDH	-0.833377361	0.494804195	1
ENSBTAG00000048620	0.048880575	0.965017209	1
FDX1	-0.160912558	0.856445882	1
TRIM33	0.977180305	0.59422846	1
RRP8	0.262471388	0.813827335	1
HAGHL	1.235039111	0.413107542	1
ARL4C	1.622192024	0.101821189	1
POLD4	0.073115598	0.937337186	1
ATG16L2	0.852240509	0.388731023	1
RORC	1.500135418	0.277955037	1
UQCC3	-0.320415684	0.746398963	1
ATXN7L3	0.487163487	0.74375934	1
THSD1	-0.014693896	0.988721672	1
AACS	0.229751773	0.833064026	1
NAA15	-0.65007469	0.627429719	1
TMEM50B	1.566405908	0.13521275	1
ABL2	0.022646315	0.983344335	1
PDXP	0.296727496	0.834160493	1
CFAP410	-0.07707497	0.937717071	1
SPTBN1	2.207230201	0.226404239	1
LPP	0.345401809	0.815245609	1
AMOTL2	-0.080757651	0.956108415	1
MDFIC	1.155632866	0.44657623	1
ART5	-0.434481394	0.711605592	1
CCDC191	0.682811598	0.587646742	1
GALNT17	-1.204282144	0.302483725	1
BRWD3	0.83273279	0.468267505	1
ZNF451	2.329082942	0.277333711	1
CHM	-0.455403511	0.750303281	1
IL27RA	-0.241327404	0.805201922	1

NIPA2	-0.584261173	0.567234727	1
ZNF608	-0.14204272	0.888474196	1
OMA1	-0.190127392	0.853625786	1
TBC1D5	0.073017096	0.960124666	1
LARP1B	0.529707098	0.753538168	1
NAGLU	0.198772058	0.834430103	1
TFAP4	0.767611618	0.529570772	1
TNFRSF11B	-1.165894056	0.377105474	1
TGM7	0	1	1
GATC	-0.93886835	0.49142332	1
PLCL2	-1.266248581	0.314758607	1
SOAT1	1.649978285	0.193877298	1
RABGAP1L	-0.5703692	0.705881543	1
FIZ1	0.850325259	0.548255724	1
SMURF1	2.126050975	0.227570057	1
DMXL1	0.016832827	0.991455531	1
TRIM6	0	1	1
MTHFSD	-0.754489374	0.616966273	1
SEC24A	0.069081212	0.940991938	1
SLC35F6	-0.137817624	0.885266699	1
DUS4L/BCAP29	0.10810088	0.939152685	1
PIK3AP1	0.371864391	0.793285456	1
RETSAT	-0.580953261	0.541138649	1
GATAD1	-0.049521966	0.953727984	1
CASP2	0.464031199	0.742880027	1
PMM2	1.136453196	0.444891474	1
MDN1	1.39426223	0.365730301	1
KCNJ8	-0.550190202	0.626040993	1
CEBPG	0.088898447	0.937386673	1
OSBPL5	1.085944363	0.253822705	1
MTURN	1.926566605	0.243454247	1
COMMD8	-0.553164149	0.57010722	1
MRPL36	0.276148691	0.804839797	1
PIP4P1	0.593993846	0.513352312	1
OSGIN2	-0.292119144	0.720724978	1
TUSC2	0.054552734	0.953368552	1
ART4	1.530195594	0.515148188	1
UBXN7	1.085333556	0.341535146	1
NLRC5	-0.881853189	0.540772773	1
C14H8ORF33	-0.60161973	0.686922151	1
LOXL3	-0.117408065	0.914694029	1
SIMC1	-0.141011735	0.929657054	1
SHROOM4	-0.58357414	0.69102282	1
ZBTB44	-0.212248767	0.888826677	1
GTF2F2	0.116895376	0.910546777	1
PITX1	0.387807247	0.727320872	1
ABHD8	0.220652444	0.850286872	1
AEN	1.150258348	0.484711298	1
MERTK	-0.532414469	0.713550041	1
DVL2	0.403421835	0.786261026	1
DNTTIP1	0.630980158	0.719670704	1
ASXL2	1.542067031	0.241687571	1
PLAU	-0.546932421	0.71411328	1
DENND6A	1.022408387	0.414188376	1
CENPC	1.444156995	0.270409784	1
OPA1	2.042414238	0.313137701	1
DNAL1	0	1	1
ZEB1	-0.829167196	0.680496417	1

ADAM15	1.10270029	0.587987774	1
THOC6	1.945505238	0.158028817	1
URI1	1.450651838	0.264532338	1
CREM	-0.213526428	0.837145285	1
FDX2	1.200447677	0.301364452	1
TXNDC5	0.995975962	0.503031054	1
ENC1	0.362398659	0.734051179	1
POFUT1	0.535765728	0.627209375	1
PINX1	0.497547574	0.67453952	1
EHD4	1.602274993	0.343402791	1
STX16	-0.066108898	0.966982257	1
SENP5	0.468436515	0.772837081	1
RSBN1L	0.575367094	0.614425895	1
RWDD1	-0.053512663	0.962259495	1
ALPK2	-0.184885147	0.910288975	1
GPR108	0.847918015	0.553152522	1
MTBP	-0.247356335	0.784305628	1
RBMS1	-0.56711735	0.704403727	1
TCF4	-0.841038123	0.628015194	1
STRADA	1.42957034	0.386038183	1
SEMA4F	0.262519907	0.832765834	1
EIF2AK1	-0.661351566	0.636090494	1
ZDHHC12	0.57575508	0.601663342	1
SLC6A14	0	1	1
HOXA4	0.840392016	0.450806525	1
POLR3H	0.761772134	0.440501527	1
CAPRIN2	-0.506614243	0.723797876	1
ORC3	-0.261465425	0.858122753	1
DUSP13	-0.712140176	0.582816396	1
CMTM6	0.416260181	0.705136328	1
IMPDH1	-0.468962562	0.743830099	1
CEACAM1	-0.784475462	0.580055941	1
ERMARD	0.17191815	0.930412225	1
SRGAP1	1.578787207	0.280213594	1
AP2A1	-0.772973012	0.452153895	1
LOC512672	-0.188772904	0.901589572	1
APOM	1.311187579	0.359092646	1
PSORS1C2	0	1	1
PEX2	0.454860999	0.672820313	1
TPGS2	-0.19282067	0.899672616	1
CETN4	0	1	1
GUCY1B1	-0.766567054	0.616023985	1
PRDM15	-0.052043508	0.973196425	1
NINJ1	-0.414881515	0.693831235	1
G24673	0.078425736	0.940149993	1
CD320	-0.063150487	0.966169298	1
APOOL	0.200058809	0.869886554	1
ASAP2	0.088943379	0.936408841	1
ZNF638	0.901765105	0.592313022	1
PIGM	0.612072762	0.621792404	1
RGL3	2.785035498	0.087400465	1
MEAK7	1.644221844	0.328822167	1
FAM118B	-0.05219664	0.961921767	1
GIMAP8	-0.310851799	0.730814575	1
MAGOHB	1.090117777	0.252115266	1
ESR1	-0.776798714	0.575524762	1
YAE1	-0.776821984	0.595266587	1
UEVLD	-0.014077946	0.992484147	1

ETAA1	0.997838634	0.410961473	1
ENSMMSG00000022600	0.845694791	0.610361709	1
LOC618409	0.834338738	0.56616313	1
OVCA2	0.065908305	0.948114096	1
SLC9A3R2	-0.678493747	0.661731747	1
ABLIM3	-0.83810289	0.621311565	1
ZNF470	2.140394249	0.138030894	1
RAPH1	-0.273673523	0.760881942	1
IGFLR1	1.147729542	0.430393521	1
CASP6	0.439754033	0.617941444	1
MYO19	-0.545364608	0.572202582	1
TUBD1	0.311773159	0.837072193	1
SLC38A3	0.090778586	0.960769506	1
CMC1	-0.292909261	0.804144737	1
MRTFB	-0.447388092	0.764720147	1
RAB32	0	1	1
ZNF575	1.272379412	0.332439859	1
CERCAM	-0.592542735	0.619534255	1
ZDHHC7	-0.198019097	0.88475029	1
LIG1	1.002491249	0.582270628	1
SH3TC2	-0.224281998	0.817245896	1
GOLM1	-0.903983384	0.371497877	1
PPP1R3F	-0.886538074	0.541918317	1
ZNF583	-0.185189582	0.833523774	1
WWTR1	0.693005652	0.510809615	1
UEVLD	-0.423755692	0.665133222	1
YEATS2	0.846195251	0.606701872	1
TRAF3	0.637329452	0.545931901	1
TRAPPC2	0.411582631	0.691772246	1
TMEM41A	0.803693654	0.41341997	1
G32555	-0.170347107	0.905319378	1
CBFA2T2	-0.020692175	0.989693525	1
PGM2L1	-1.055085777	0.412357316	1
LOC107131494	1.182414161	0.312894236	1
TMTC3	0.581879607	0.708246217	1
TP63	-0.641079578	0.647373716	1
ARHGAP12	0.775098646	0.568713066	1
ZMYND19	0.559470579	0.63831538	1
LMO2	0.141338417	0.903791873	1
DRAM2	0.279517889	0.850103382	1
C23H6ORF47	0.472148569	0.721515556	1
STX10	0.135887017	0.908943784	1
TGFB1	0.820511126	0.515596723	1
MAZ	-0.702060884	0.631897445	1
CDCP2	0	1	1
BNIP1	0.536933624	0.640908677	1
USP37	0.183295618	0.910833279	1
STK3	-0.481133918	0.624557773	1
RNF169	1.228648508	0.345800725	1
RCE1	0.689500335	0.771392953	1
ABHD2	1.045006669	0.428971947	1
FAAP20	0.668004486	0.575124216	1
MND1	1.096732062	0.310370237	1
TRIM35	0.153988257	0.919939428	1
PGAP3	2.196821722	0.096728009	1
EEF1AKMT1	0.99382928	0.485959464	1
SLX9	-0.208637834	0.85678198	1
ARNTL	0.54726937	0.76045937	1

MMP7	0	1	1
TMEM263	-0.144908237	0.850077615	1
ACP1	0.627215302	0.542059046	1
F8	-0.712520727	0.62606647	1
CREBRF	0.12975128	0.935969019	1
RPL37A	1.069981706	0.435544172	1
ZBTB20	0.995269725	0.494184475	1
CDC27	0.833335757	0.621904182	1
RTN4IP1	-0.464310505	0.64247479	1
ADGRA1	2.191565164	0.38397146	1
POSTN	0.626544174	0.769943158	1
KCNMA1	1.758385142	0.298598364	1
EXOSC1	-0.479980022	0.621796245	1
ACTR6	0.373329943	0.715793318	1
TXNDC12	1.300743098	0.229899517	1
TMX1	1.8600078	0.186568796	1
CIBAR1	1.965994197	0.140542691	1
BCLAF3	0.852055011	0.400149893	1
MECOM	0.162813454	0.875066351	1
JMJD8	0.453636705	0.587831058	1
PIBF1	0.38778884	0.746543026	1
CARMIL2	0.797293822	0.402498086	1
AFMID	2.508570532	0.082785402	1
LIMS1	0.907616953	0.442861932	1
FBXL14	0.270734377	0.809562126	1
C13H20ORF27	-0.948344927	0.247055105	1
PLK3	0.259637241	0.824527097	1
CLN6	0.918499974	0.3628858	1
METTL17	0.607089428	0.628228892	1
ZBTB5	0.045751798	0.967085003	1
GFRA1	0.03888871	0.979532379	1
GAN	1.399753216	0.17675967	1
PCDH12	-0.639019798	0.532576204	1
SERGEF	0.546230702	0.623841274	1
TASP1	0.819910482	0.607801223	1
ARL16	0.375825576	0.790248521	1
FAM43A	-0.352072062	0.674201646	1
UBE2W	1.058561342	0.511959885	1
GRAMD2B	1.671765099	0.209458895	1
ZNF830	-0.395486651	0.699714826	1
EVA1C	0.965023228	0.323784548	1
FRMD6	-0.437242185	0.831477315	1
ILKAP	0.891972887	0.574635549	1
FUNDC1	-0.327999788	0.77531291	1
CFAP418	0.569679705	0.626754483	1
SLU7	0.660967166	0.689540856	1
CUX2	7.141582296	0.104103472	1
PTRHD1	1.039442068	0.383168874	1
MRPL52	0.096037413	0.937896378	1
HPS6	0.630790811	0.522047606	1
CDC7	-0.126146704	0.931397837	1
ADGRL4	-0.826760832	0.55732737	1
CACNA1G	0.970542559	0.320849373	1
ATP2B4	-0.249356353	0.900208848	1
ALG8	-0.253173786	0.80238659	1
TCTN3	-0.267587073	0.798441465	1
GSR	-0.057466346	0.969435339	1
ZFX	0.02096685	0.985640453	1

DTX4	0.094362079	0.929198377	1
FCHSD1	0.888231861	0.493964874	1
SESN2	2.19435902	0.142634447	1
TOMM34	-0.012380495	0.9907252	1
ZDHHC18	0.505225157	0.706441609	1
VNN1	0.965751589	0.507780103	1
SLC26A10	0.281283628	0.946173166	1
MSRB2	0.520683898	0.707843801	1
ENSMMSG00000022039	0	1	1
KIF2A	1.619008053	0.310259594	1
FNTB	-0.462652382	0.613858157	1
UPF3A	-0.009909185	0.992314871	1
RIOX2	-0.127867348	0.891306534	1
DUSP13	-0.375364374	0.816303213	1
KICS2	1.164636312	0.487675206	1
TRAPPC5	0.957455331	0.342833272	1
MANBAL	1.249809323	0.466535675	1
RTL5	-0.321052198	0.731951532	1
XRCC4	0.899100411	0.563780562	1
RFTN2	-0.562960959	0.533810745	1
NTRK2	2.070734482	0.185583657	1
PNMA1	-0.188220102	0.898013396	1
PXK	-0.247802379	0.797098629	1
C29H11ORF98	-0.569330674	0.594184679	1
PTK2B	-0.11993762	0.920758952	1
UCK2	1.082612095	0.439592868	1
SMO	1.579585222	0.251410526	1
NHSL1	-0.783330569	0.696165646	1
PLEKHA7	1.203225474	0.278292935	1
IGF2	0.025256627	0.986008058	1
RHBDD1	0.280989758	0.719329	1
ASB4	-0.278251348	0.853163038	1
FKTN	0.379278022	0.705143799	1
ENSMMSG00000013535	0.515482472	0.632473455	1
PAQR6	-0.439216008	0.665520993	1
MSS51	1.658316548	0.24979972	1
MAP2K5	-0.452144296	0.683597663	1
KIN	0.79112125	0.527685266	1
PHKA1	-1.297607124	0.269779925	1
AOX2	3.610504166	0.082118461	1
DYNLT1	0.025819735	0.982792923	1
ZNF70	-0.244292229	0.845552812	1
TRIM65	0.780139012	0.54901144	1
FTSJ1	0.644271603	0.633684895	1
PTPRT	1.948432521	0.379485492	1
TK2	0.434159251	0.662283951	1
CADM1	1.784824238	0.238788591	1
ZBTB10	-0.410649342	0.793062485	1
ENSMMSG00000004106	0.046284971	0.976948647	1
ZMIZ1	-0.477265833	0.753468374	1
MIDN	-0.389791009	0.789192292	1
STK4	0.532916558	0.559276627	1
PTPRU	0.028865407	0.980825515	1
LOC508666	-0.245816447	0.798025315	1
RAB7B	0.260849883	0.819960193	1
BTBD9	-0.228662383	0.824806109	1
GYG2	3.213348769	0.088434552	1
CD320	1.024132635	0.415117184	1

LOC515578	0.466063981	0.689927808	1
MTRF1	-0.6763961	0.640603859	1
RWDD2B	1.321281952	0.308990284	1
CKAP4	-0.165123091	0.857508514	1
MOSPD2	0.469068705	0.647845365	1
MALL	1.806079602	0.127345486	1
CCDC97	-0.10244266	0.929393455	1
SF3A3	0.752713138	0.66642565	1
C3H1ORF50	-0.239748684	0.78011402	1
SRRD	-0.192473708	0.857699839	1
SEC14L1	-0.33660272	0.764821889	1
PPP1R15B	-0.09047435	0.931486573	1
495184/LOC101732638/LO(	1.452276601	0.246491091	1
DAAM2	-0.616305547	0.591473172	1
NCBP2AS2	-0.292835863	0.766794654	1
RASSF1	-0.472044621	0.619582388	1
SQOR	0.360056002	0.770395946	1
BCL7B	-0.532744637	0.5907076	1
C3/PCDHGB4/PCDHGA8/PC	0.595997954	0.711738227	1
SLC38A7	0.225679163	0.845481074	1
ENSMMSG00000017630	0.724493224	0.50060063	1
SDHD	-0.679573288	0.641580347	1
GALNT16	-0.707199002	0.612405358	1
C1H3ORF52	0	1	1
PHLPP2	1.444771064	0.39056055	1
WFDC1	-0.881660877	0.414599214	1
FREM2	-0.073936937	0.961939638	1
SHMT1	0.659609129	0.621378249	1
GPR39	1.961060551	0.22577857	1
KCTD1	1.474951141	0.15999089	1
RCOR1	0.446589429	0.654506307	1
PPP1R14D	2.838999469	0.101434723	1
RTTN	-0.132399208	0.892806432	1
KCNJ2	-0.273045915	0.856528436	1
ACTRT3	-0.866649135	0.388568249	1
LDAH	1.201677452	0.343945578	1
PTS	-0.261065965	0.771751286	1
FAM118B	-0.477283192	0.84214007	1
MED11	0.936631266	0.433732128	1
MAPRE3	-0.030529092	0.984830909	1
MYO9A	0.851557876	0.529008328	1
SNX16	0.47963134	0.771196222	1
CRIPT	-0.597150084	0.523788824	1
WDR5	-0.825506264	0.589442127	1
PRKCZ	0.517416517	0.636306263	1
SEPTIN12	0.06615068	0.956826969	1
TRMT11	-0.284972645	0.774534924	1
SCAF11	0.527443476	0.749199275	1
NOM1	-0.911381476	0.667281172	1
NT5M	0.097718313	0.936401489	1
TPK1	-1.105719025	0.437613307	1
OSR1	2.413379537	0.090557157	1
ASPH	0.155869098	0.915095802	1
RRAS2	0.027768921	0.985824511	1
PPIG	0.705930314	0.66333708	1
POC1B	1.214197579	0.472164187	1
TBKBP1	-0.748289269	0.497831404	1
NCCRP1	0	1	1

KASH5	0.150746532	0.928510609	1
LOXL2	-0.69709861	0.542879117	1
PSD3	0.271290979	0.792911306	1
TMEM161B	0.298906563	0.8503348	1
COPG2	0.81104228	0.476620696	1
EPS15L1	0.576593838	0.656544085	1
ARVCF	-0.378153579	0.784830523	1
UGDH	-0.745018658	0.61287305	1
DIAPH2	0.567982574	0.73634307	1
CASP9	1.574489606	0.277872536	1
PRDM11	0.839161194	0.538148349	1
RECK	0.231861404	0.828733298	1
MTMR9	0.57370806	0.565879591	1
RASSF4	-1.235758974	0.401840392	1
KCND1	-0.566453276	0.595171235	1
GTPBP10	-0.134479091	0.927678295	1
ATP2A3	-0.298161687	0.736205507	1
BTBD3	-0.547735181	0.600178699	1
MYLK3	0.033863139	0.984600508	1
COL6A6	-1.028822711	0.591722906	1
PIGP	-0.0745709	0.943024835	1
CELF1	0.437784357	0.754458914	1
GLIPR2	-0.084933608	0.937121668	1
RPAP2	0.01577545	0.988701349	1
POGLUT2	0.59333466	0.614236464	1
GTF3C4	0.293702282	0.795211859	1
RECQL	0.227096372	0.876239103	1
MYRIP	0.893863257	0.56040894	1
MGC127133	-0.841548121	0.501050942	1
EXT1	0.050297303	0.958295521	1
BTNL9	-0.679768722	0.624653117	1
ID1	0.505477813	0.678877596	1
WDPCP	1.555016633	0.140372561	1
RFC5	-0.184438679	0.89782485	1
NDC1	-0.448266675	0.636221445	1
STK3	-0.205555566	0.890962788	1
RAB29	1.463867303	0.331230223	1
TRIM45	-0.242136923	0.821258105	1
MANSC1	0.353532012	0.705608696	1
TOR3A	0.287010216	0.811745173	1
FBXO7	0.693417206	0.593408714	1
RNF223	0.022385401	0.978232939	1
NUP37	-0.899499821	0.34971662	1
AKT3	-0.691613278	0.505217144	1
STMN1	-0.039348035	0.972207797	1
DPY19L1	0.388402683	0.78731963	1
SDHD	-0.851326631	0.385227951	1
RPE	0.365987556	0.713590428	1
MYSM1	0.381252745	0.800160065	1
G22367	-0.127866072	0.933449361	1
FMR1	0.356682483	0.708933916	1
EARS2	0.407568031	0.720171509	1
DUSP29	-0.885717182	0.464153612	1
ABCA2	0.768300339	0.390366864	1
MAP4K5	0.249347742	0.837311411	1
SLC8A2	0.002904364	0.997747195	1
PLAGL2	0.420022507	0.772990516	1
HSPA14	-0.171215241	0.907221147	1

CISD2	0.211671511	0.833721169	1
ELL2	1.089042258	0.265285685	1
RAPGEF6	1.389668218	0.394913573	1
ACSM1	3.636958284	0.115966022	1
THBS3	-0.365951692	0.641543711	1
SNRPD2	0.204360014	0.84081688	1
HTRA3	-0.920190174	0.36029292	1
TMEM150B	-2.849282797	0.147465679	1
NIN	0.011418719	0.99239118	1
LMO7	-0.30922524	0.874373182	1
KIF16B	0.838478048	0.576396721	1
NUDT6	1.132247515	0.507385026	1
SLC7A4	1.391029245	0.245141425	1
TES	0.24477153	0.805909821	1
ISM1	0.499235414	0.616050447	1
DHX33	0.334429351	0.76119844	1
HAUS6	-0.181732098	0.859774866	1
DNAI2	1.629716695	0.331079066	1
MARCHF2	0.588639058	0.56113513	1
SRXN1	-0.643367282	0.577439952	1
GTF2IRD1	1.009567314	0.481920449	1
UPRT	-0.017116475	0.990465287	1
YTHDC2	-0.037267317	0.979915325	1
DESI1	1.175092292	0.291859194	1
PLEKHA1	-0.812265446	0.585228675	1
POLR1F	1.294069346	0.262914734	1
NMNAT3	-0.521185906	0.718790878	1
IPO11	-0.044464402	0.962197201	1
NIPSNAP1	0.282151167	0.782809163	1
ENSBTAG00000008857	0	1	1
CNOT8	0.18193942	0.864912821	1
MTF2	0.12109357	0.914034061	1
SETBP1	0.422700318	0.804619689	1
PGPEP1	0.754288891	0.635508662	1
PARD3B	0.459277916	0.63349223	1
IMMP1L	0.358101912	0.706844352	1
BPI	0.503422635	0.657371969	1
SNX11	-0.142726139	0.900416341	1
C18H19ORF48	0.026817149	0.980018712	1
FTCDNL1	0.633258556	0.473414218	1
CCNH	-0.272845193	0.787483158	1
PDCD6	0.56720141	0.653401682	1
PIEZO2	-0.373464311	0.733927939	1
PBXIP1	-0.045645886	0.97277498	1
LOC101906131/IER3IP1	-0.011764812	0.991188637	1
MTMR9	0.13441516	0.935780531	1
TMLHE	-0.2964166	0.732787174	1
PPFIBP1	0.350532437	0.885436576	1
TGFBR1	-0.175905773	0.907058001	1
CCDC86	-0.043575732	0.958993277	1
RPUSD4	-0.504294028	0.616216218	1
FAR1	-0.343367127	0.825079361	1
IRAK2	0.149072374	0.869065239	1
HNRNPDL	-0.630273521	0.659686632	1
CCDC61	0.717792194	0.560632925	1
CORO1A	0.889812075	0.435226593	1
MAMSTR	-0.505625785	0.593717019	1
PPWD1	-0.003419037	0.998285281	1

CHRNB1/FGF11	0.981487702	0.378887294	1
CHRDL1	-0.061211072	0.960128432	1
TOR1B	0.312199366	0.830573747	1
ZFYVE19	0.970884753	0.389975379	1
LANCL1	0.262879574	0.811499107	1
WWC3	-0.025084695	0.982484385	1
TBC1D4	-0.612955718	0.668319264	1
LRP12	0.893886464	0.617699343	1
TBC1D4	-0.750703925	0.640053462	1
NUAK1	-0.758900567	0.499079309	1
CXCL14	0.819221814	0.478724945	1
ZNF362	-0.009152178	0.992709017	1
SYNRG	0.827468423	0.58959761	1
NT5C2	1.403422636	0.349975375	1
SGCE	-0.584148604	0.694781628	1
GASK1B	-0.051843893	0.972658781	1
CAPN6	0.659977774	0.676264731	1
SPRTN	0.772724266	0.523926538	1
OTULIN	0.737380475	0.567645762	1
RPS4	0.932568235	0.422999927	1
XIAP	0.555705634	0.735608282	1
B3GAT1	0.837106591	0.618346362	1
STT3B	2.091117831	0.1741216	1
DOK4	0.358852843	0.804760783	1
CLP1	-0.065194584	0.951285017	1
CASTOR1	-0.011009681	0.990908835	1
CPED1	-0.1918619	0.900472213	1
SPIN1	0.522984842	0.673885803	1
INTS9	-0.06495285	0.946357753	1
ENSMMSG00000008461	2.031827073	0.130987684	1
TTC7B	0.029325711	0.985287822	1
GDAP2	0.485450178	0.74328226	1
THEM4	1.199868512	0.419656509	1
XPA	0.738155095	0.548858126	1
INSYN1	-0.786002584	0.531678528	1
KIF1B	-1.04542076	0.658236774	1
H3F3B/H3-3B/H3-3A	0.137407907	0.847092216	1
FAM118A	-0.039145361	0.981004544	1
PEX11G	1.55004629	0.121967551	1
SPCS3	-0.131034042	0.877467976	1
ZC3H13	0.318552408	0.799728645	1
KIAA1143	0.037464346	0.967124653	1
ANTXR2	-0.322519553	0.795599576	1
NAB1	-0.025552259	0.980148156	1
ZDHHC9	0.154132159	0.875642497	1
FXN	-0.577606325	0.521978965	1
PRKAA1	-0.027748779	0.976737627	1
SLC29A3	0.690061615	0.629642105	1
CASP8	1.260266785	0.212239113	1
ZFP28	-0.135189697	0.933505678	1
NAGPA	0.20628551	0.864614165	1
SLC41A3	-0.380268493	0.676061067	1
CACHD1	-0.796242279	0.468607419	1
EXOSC3	0.053825769	0.953691951	1
PPM1D	0.009050396	0.995257589	1
C22H3ORF62	0.042545371	0.964388463	1
CIB2	-1.129532026	0.373050573	1
RHOF	-0.093182304	0.926722703	1

C1QTNF9	-0.931221557	0.345572841	1
PRR5	1.550482233	0.272364255	1
EYA2	2.460208562	0.190319691	1
LOC613444	-0.319582492	0.778708135	1
ILRUN	-0.681626022	0.649729877	1
PCBP2	1.524177422	0.274061011	1
KANSL1	-0.669104088	0.731849343	1
DEDD2	-0.079550848	0.950022942	1
TRAF1	-0.61644889	0.53430326	1
DOCK4	-0.652170389	0.736019382	1
KBTBD7	0.508443139	0.700361563	1
BOK	1.020351669	0.329041128	1
RBM33	-0.289620801	0.856032041	1
CD209	-0.472572633	0.631059067	1
CDADC1	-0.039943714	0.97078792	1
MED26	-0.147065084	0.926174644	1
CRISPLD2	-0.742179422	0.480506832	1
COMMD2	0.163143276	0.860203204	1
FAM161B	-0.727077672	0.609652523	1
CAMK2G	-0.105310259	0.933551529	1
SOCS6	-0.793786472	0.604145223	1
G10276	1.817802089	0.094521426	1
TSNAX	-0.065155504	0.967535364	1
PBX1	1.261517557	0.378738484	1
NRP1	-0.493383038	0.623930869	1
YJU2	1.278969655	0.308903354	1
PPP3CC	-0.866787148	0.646213224	1
DCAF4	0.264575771	0.865573657	1
MDC1	1.257203097	0.353983751	1
PCBP2	-0.547667156	0.477400483	1
GK5	1.013967606	0.404073075	1
M-SAA3.2	0	1	1
PIP4P1	1.055206037	0.545201984	1
RIN3	-0.78679405	0.289253621	1
SLC25A24	-0.090097514	0.931532057	1
LRRK1	1.071987557	0.320267249	1
PKNOX1	-0.4382703	0.769537452	1
ADM	-0.901240573	0.284971291	1
TMEM177	1.197524602	0.37707792	1
MCTS1	0.377175944	0.753564415	1
ADGRE5	0.288589479	0.769464888	1
C18H19ORF54	-0.203747951	0.849270641	1
THAP12	0.052822514	0.963154002	1
ARPC5L	-0.235441993	0.786101435	1
CHIC2	-0.094839276	0.902722293	1
IBA57	-0.324031767	0.766314027	1
PDGFB	-0.837097609	0.552386952	1
ZNF667	0.751797092	0.619660673	1
HSDL1	0.716483924	0.611447679	1
AAK1	-0.974113754	0.279049942	1
SHFL	0.739200087	0.530572609	1
ODR4	-0.242190965	0.8099252	1
CLCN5	1.46572056	0.155636994	1
ENSBTAG00000011178	2.072158807	0.088202422	1
G16407	-0.776366174	0.619101655	1
DCAF17	0.495340655	0.761548556	1
SNRNP35	0.43592756	0.708341447	1
DESI2	-0.041182736	0.969210737	1

PLEKHG5	-0.728197109	0.358904426	1
TBC1D5	0.897258147	0.393516689	1
SNX29	0.216498698	0.892951333	1
KIAA0586	0.289373411	0.76790707	1
RFX7	0.274185825	0.834981302	1
CREM	-0.615656166	0.57158741	1
PLA2G6	-0.174566753	0.85364457	1
DOLPP1	0.561062211	0.70443148	1
TBC1D2	1.055299637	0.333229557	1
MCF2L	-0.508258034	0.582388596	1
ASDURF	-0.676989424	0.647076341	1
RABIF	-0.588505127	0.684819754	1
ZNF503	0.157578044	0.87571103	1
ZNF740	-0.063009549	0.942667811	1
RUNX1T1	-0.933763787	0.523529153	1
TTLL5	0.859396856	0.625086187	1
GAL	-1.815627457	0.541898894	1
YWHAZ	0.211154846	0.883983399	1
VCAN	-0.722217615	0.62877959	1
DPF3	-0.496656749	0.61517691	1
SELENON	-0.117847422	0.935785932	1
HOXA9	-0.373861437	0.697281986	1
SNUPN	-0.499033572	0.748245594	1
CFD	1.080688813	0.315264315	1
KIF9	0.742437331	0.508970706	1
NRADD	1.531219319	0.228883574	1
CEP170	-0.588487745	0.560034108	1
FAM151B	0.44374215	0.682779799	1
SPATA2L	1.596884679	0.153997045	1
ZNF521	-0.151588001	0.915526551	1
C2CD5	0.816346503	0.492809123	1
AOC3	-0.744993313	0.619925841	1
CASK	-0.512217368	0.728067571	1
FKRP	-0.199894281	0.861245272	1
TIMMDC1	-0.386907106	0.645932033	1
ENSMMSG00000022345	0.355369048	0.773538421	1
CCNT2	1.255867993	0.462329303	1
NOCT	-0.902271494	0.281821606	1
GADD45A	-0.800123774	0.434211136	1
LOC101905951/CMC2	-0.626895162	0.565005545	1
STXBP1	1.29027231	0.321567464	1
IFI27L2	1.702754236	0.184979516	1
TNIK	0.269578179	0.854822534	1
G1352	-0.509044508	0.757125565	1
TCEA2	-0.617164832	0.520764222	1
CTPS1	-0.277382633	0.776773238	1
SOS2	-0.480192646	0.69904821	1
RXRG	-0.149883944	0.918003631	1
ZNF394	1.229722977	0.24460073	1
PUM2	1.223359839	0.481127452	1
HIVEP2	-1.038878604	0.468312259	1
KDM4C	0.606264689	0.663673978	1
PEAK1	-0.61762096	0.664488271	1
NDUFAF4	-0.426491959	0.647326094	1
NUDT2	1.005980866	0.397473276	1
POLE4	1.678732534	0.192932491	1
TENM3	-0.094731406	0.950288461	1
DST	-0.125445363	0.944029039	1

ENSMMSG00000003429	-0.082221552	0.956213783	1
IPO11	-0.157553719	0.915932395	1
PDCD2L	0.296349723	0.82868927	1
JAML	2.392481209	0.091985361	1
ABHD10	-0.150546377	0.875212653	1
SLC44A3	2.654083704	0.104619545	1
UBIAD1	-0.439713229	0.647911533	1
NUP62	-0.40993149	0.676774102	1
CNTFR	-1.029159399	0.499603047	1
NFATC4	0.29995809	0.764834712	1
MAP3K2	0.764225264	0.401841607	1
GFOD1	-0.832216576	0.586230396	1
USP3	0.028948783	0.976662078	1
DENND1B	-0.088335269	0.960548417	1
CHEK2	0.875221412	0.402277489	1
DNAH17	0.868200724	0.527646987	1
EXOSC8	0.902186476	0.478265056	1
SYNGR1	-1.104129725	0.2834945	1
PPM1F	-0.368315157	0.708116057	1
SLC16A13	0.095004809	0.928977328	1
ZNF215	3.179245235	0.162601936	1
ARHGAP6	-0.47311162	0.768642471	1
CTDSP1	0.410995521	0.813714098	1
BCAS3	-0.50199373	0.727130545	1
PDK3	0.783515866	0.475579656	1
KCNN3	-1.059414648	0.268140041	1
PNKD	0.337631679	0.821408601	1
POLD3	1.238567994	0.445611979	1
RFT1	1.563199034	0.187176315	1
NPHP3	0.615501447	0.508639352	1
FAM163B	0	1	1
CA2	-1.223229795	0.267262345	1
GPATCH11	0.344395967	0.777920301	1
LRFN3	1.38018304	0.318945241	1
USP40	0.455795984	0.852447753	1
OSBPL1A	-0.678359643	0.650720194	1
WRAP73	-0.0975936	0.919064028	1
REL	1.027408386	0.294128777	1
FCHSD2	0.143974592	0.921170555	1
PDZD11	0.086952864	0.914870227	1
RAB8B	0.622382917	0.549690749	1
CDC26	0.377264434	0.73604319	1
GAB2	0.199829773	0.894159132	1
ZNF438	0.916760773	0.589848362	1
LTN1	-0.438252075	0.767421064	1
CNIH1	0.960174447	0.420641641	1
GPR155	-0.576662786	0.708848737	1
STAT3	-0.010167664	0.991807073	1
P2RY2	-0.103533493	0.94579533	1
SEMA6D	-4.011834577	0.33548179	1
RNF34	-0.2404255	0.881042335	1
IFT140	-0.160187107	0.889814544	1
ZNF266	0.157603301	0.887251309	1
DSG1	3.418758239	0.116593844	1
SOX15	1.063724919	0.393747838	1
MYO19	3.782960746	0.387814868	1
ENSMMSG00000016879	1.200661369	0.450630355	1
GBP5	1.661831815	0.251848746	1

NUDT5	0.846733867	0.422534587	1
KCTD6	0.120303804	0.925102596	1
ECHDC2	-1.239851775	0.273223601	1
IL36RN	0	1	1
SUGT1	-0.907043474	0.546528896	1
B9D1	1.034644062	0.356693097	1
ENSMMSG00000021481	-0.018535067	0.990570877	1
BCOR	-0.383248357	0.869507105	1
RNF19A	-0.457071951	0.754886075	1
LRRC57	0.25901628	0.784692631	1
SELENOS	-0.358101149	0.630694587	1
TSPAN15	0.401133542	0.754185359	1
FAM210A	-0.245492405	0.879081298	1
CCDC8	0.21392679	0.891934302	1
CCDC92	-0.50633325	0.640437376	1
LOC112442305	0.529828337	0.717215371	1
ZDHHC1	0.446606311	0.659298963	1
DCP1B	-0.072777921	0.939195303	1
HYAL3	-0.382575025	0.713716213	1
COMMD10	-0.228226926	0.878600265	1
TMEM242	-0.59650582	0.525670846	1
CWC25	0.476686079	0.623359906	1
TOP2A	2.437023786	0.110071609	1
DHX35	0.617436743	0.603063239	1
SCN3B	0.292141099	0.861406166	1
DOCK4	-0.545066353	0.531928417	1
GEMIN5	0.782395418	0.622004446	1
FAM234B	0.795988649	0.440940288	1
STAMBPL1	-0.930346743	0.439701517	1
SLC1A4	0.220832011	0.890160392	1
FBXO28	0.607620088	0.625287054	1
MIER3	0.035384674	0.981622803	1
ADPGK	-0.135784667	0.900932189	1
ARRB1	0.872653355	0.567987671	1
PLD2	0.386936395	0.685506232	1
FANCM	0.716056235	0.690132564	1
INO80D	0.366594939	0.707533776	1
BICC1	0.755377617	0.667387633	1
KCTD13	0.185291652	0.900689994	1
SLC25A14	-1.116321717	0.449230928	1
SCFD1	0.218663067	0.842394935	1
SPATA7	-0.154159583	0.890666886	1
PCDHGA12	1.200863648	0.440569905	1
G31086	1.196033689	0.2891044	1
RIMOC1	0.530707041	0.625863103	1
RPL7	0.576005425	0.599658693	1
G6PC3	0.053021416	0.955511244	1
TXLNG	0.846644542	0.621154406	1
PGP	-0.777808679	0.463564214	1
SYNE3	-0.26645595	0.797577234	1
LDLRAD3	1.610348672	0.393660856	1
G9007	-0.03027151	0.975323536	1
PIGG	1.173468067	0.371566166	1
VASN	0.502390289	0.644586588	1
TRAF3IP2	1.232805554	0.217395819	1
ADCY5	-0.182336606	0.907202503	1
ABHD17C	1.241954864	0.392743238	1
FSD1L	-0.092969408	0.95385424	1

RELT	-0.753947202	0.492726645	1
CSGALNACT1	1.199308379	0.234796533	1
SLC25A23	-0.317425846	0.840839885	1
TBC1D12	0.247508574	0.83046466	1
CEP63	0.593805759	0.601543722	1
APOLD1	-1.032116125	0.457380118	1
TOMM40	-0.72413631	0.479801863	1
RXRB	-0.661170819	0.53932185	1
TAF4B	-0.122345983	0.902730135	1
H2AC20	1.568911504	0.205529823	1
CHST1	0.247014999	0.876777077	1
SCO1	0.52317465	0.650662955	1
RAB35	0.5670041	0.599883864	1
C25H16ORF72	-0.717904145	0.474476315	1
ZNF407	-0.398898888	0.688689803	1
EML1	0.317919992	0.896265091	1
MYNN	-0.403473041	0.787271885	1
SCAMP4	0.063179908	0.948853132	1
COA8	0.988393689	0.413936336	1
UBE2T	0.219834217	0.812537023	1
ENSMMSG00000001016	-1.807638321	0.691405874	1
RBM26	0.7938753	0.639585402	1
VEZT	2.851147079	0.159218649	1
PPCS	1.67056209	0.253071136	1
SPATA5L1	0.002819982	0.998211972	1
ERI1	0.33374491	0.771589622	1
MPV17L2	0.414288034	0.707998768	1
CNN2	-0.307608271	0.772091827	1
MFSD9	1.759080596	0.193826638	1
CADM4	0.103328375	0.947659284	1
AFAP1	0.572326762	0.577403827	1
G24706	0.323871318	0.768783325	1
CARF	1.841479467	0.287648104	1
IRGQ	0.174426369	0.894016194	1
PHKA2	0.274403281	0.782051212	1
TTF2	1.655290465	0.192012189	1
JRK	0.047816208	0.966724639	1
IL17RD	0.937622642	0.431283704	1
WNK4	1.433452674	0.228136404	1
WWC3	1.159869622	0.633308079	1
LOC100336971	0.104293751	0.914597854	1
VTCN1	4.258150254	0.084232146	1
UPK3B	1.529165702	0.187237053	1
G8967	0.427287983	0.705574997	1
SOBP	-0.852857431	0.548435569	1
DUSP28	-0.669472099	0.544284739	1
ENSMMSG00000017998	0.605104029	0.554409841	1
CA11	1.511810371	0.24365098	1
MFSD11	-0.073867006	0.961830943	1
LRRC51	0.73133064	0.390356598	1
APCDD1	0.663770911	0.536109568	1
HYAL2	0.462179999	0.736692148	1
ATP13A2	0.39364417	0.744071488	1
SUPT20H	2.718619272	0.193541114	1
AUH	-0.325201964	0.827909114	1
DNTTIP1	1.662092749	0.379460027	1
KCND3	0.646379589	0.640088647	1
NMT2	1.454393803	0.279652939	1

IMPACT	-0.382926687	0.802165055	1
STX3	1.388618143	0.267731549	1
PPRC1	-0.249229674	0.786013508	1
ENSMMSG00000010580	0.891210536	0.490267308	1
LYST	0.687219143	0.669493321	1
TLCD3A	0.683811538	0.488205987	1
SGF29	-0.12228315	0.897632565	1
SS18L1	-0.314227716	0.777979709	1
PELI1	0.296425494	0.852684598	1
STOX2	-0.395481995	0.715137325	1
CACNA1C	0.636897303	0.552953829	1
MAP3K1	0.422144659	0.777655911	1
PXDN	-1.017881768	0.260831369	1
TNS1	-0.726438115	0.703520793	1
RMI2	-1.825515804	0.435903936	1
LIG3	-0.650892717	0.665654581	1
ARVCF	0.561170036	0.745072389	1
TRMT61A	0.596826394	0.579827763	1
HMGA1	0.04086601	0.979606615	1
SLC25A35	1.11756027	0.416649136	1
FMNL2	0.754251625	0.439586552	1
EMSY	-0.193030894	0.898967452	1
UTRN	0.978383009	0.354887768	1
TMEM63A	1.75120982	0.399031592	1
ENOX2	-0.63642053	0.547745722	1
H2BC15	0.626112416	0.606510439	1
POLR3F	-0.149233717	0.916991231	1
RFK	0.272992254	0.849264829	1
CCDC154	1.529040653	0.139572164	1
ITPA	0.424953106	0.721837589	1
RNF215	-0.189163034	0.852587049	1
TACO1	-0.620185353	0.556464834	1
ABLIM3	0.782262508	0.693998706	1
ENSMMSG00000020310	1.801514401	0.194510835	1
VPS37B	0.374726539	0.750526091	1
SH3PXD2B	0.782021735	0.442640195	1
TEF	0.377264957	0.710389015	1
TRIM4	1.032691373	0.488579239	1
CCNDBP1	-0.085692701	0.91360255	1
RAVER2	1.017818081	0.506392534	1
EEF1E1	-0.974002716	0.281031534	1
SEPTIN6	-0.130420502	0.928816544	1
CLN5	1.1275872	0.428218856	1
SLC29A2	-0.176666275	0.863859467	1
TRNT1	0.072318529	0.948023514	1
STAM2	0.508059616	0.557439909	1
COL4A1	-0.697833466	0.413165983	1
LOC787600	0	1	1
ZFAT	-0.231254526	0.834389004	1
DOCK9	-0.343040408	0.8400789	1
PMS2	0.969209286	0.454266711	1
POLR1G	0.822441949	0.459393381	1
KIFBP	0.540536254	0.734557186	1
GEMIN8	0.162083538	0.850287884	1
CLEC14A	-0.951232213	0.48354872	1
PLEKHB2	-0.782986391	0.587732349	1
TMEM187	0.79159205	0.519079622	1
MINPP1	1.403827529	0.334116999	1

PCDHGA5	1.245170979	0.368026021	1
PIDD1	0.602410417	0.544037074	1
MSTO1	0.103998524	0.913863341	1
VCPIP1	0.447711698	0.767395307	1
FAM189B	-0.155026099	0.874330257	1
PDZD4	3.665966725	0.088343918	1
LOC112444288	-0.269112739	0.777548251	1
ARMC1	0.309760747	0.83780544	1
POL	0.086365338	0.951217225	1
BBX	0.189932849	0.862868827	1
CYYR1	-0.653120826	0.489572576	1
FBF1	0.542618766	0.661467437	1
ZNHIT3	0.071565444	0.936169954	1
KIAA0100	-0.201231425	0.870842751	1
ENSMMSG00000009728	0.385035886	0.794608095	1
TTC37	-0.575686401	0.700070119	1
RSBN1	-0.416601297	0.672999598	1
ERO1A	0.200773963	0.824625613	1
KAT2A	2.053810303	0.13792628	1
NEK7	-0.502566494	0.736015287	1
CAPN5	0.848051121	0.343483069	1
HES4	0.488738117	0.650911655	1
OAS2	0.947977241	0.474948046	1
NEK8	1.719969398	0.132104437	1
YIPF6	0.299993582	0.738113156	1
SHPRH	-0.176872639	0.844838942	1
PRMT6	0.256295992	0.826506975	1
PHF20L1	0.843681337	0.729187612	1
SHBG	1.350502677	0.232300088	1
NIBAN3	1.162571345	0.224776925	1
SIPA1L1	0.335310126	0.844834518	1
MRTFA	-1.1517484	0.345801339	1
EXOC7	5.109698749	0.24741545	1
ECSCR	-0.654518228	0.511692114	1
TTC8	0.077458209	0.959666884	1
GSR	-0.178528721	0.910989038	1
DEPTOR	-0.157217631	0.90706412	1
MEIS1	1.423079221	0.163397062	1
MNT	-0.032454632	0.976025318	1
JAM2	-0.605901826	0.755634583	1
G32014	0.702001221	0.595446619	1
BVES	-0.85690169	0.513242875	1
SPATA2	-0.604327127	0.604416619	1
ENSMMSG00000024369	0.244493194	0.812212061	1
ENSMMSG00000021450	-0.418991871	0.661426744	1
NLN	0.685536441	0.496129131	1
MAP7D1	-0.954674638	0.381779396	1
TAF1D	0.635364885	0.525867206	1
TINF2	0.874681711	0.507524979	1
SAMD11	-0.810220591	0.456178445	1
AZIN1	1.379793107	0.354813642	1
DFFA	0.133531397	0.889836403	1
PREB	-0.654585696	0.660932124	1
CDON	-0.374994101	0.688764006	1
PRICKLE1	1.436909702	0.288131689	1
CPTP	0.293254301	0.77453278	1
NUDCD1	0.05916161	0.943829835	1
COQ8B	1.034723896	0.407550826	1

UTP15	-0.118476489	0.900282675	1
DPT	0.41488672	0.725022499	1
CWH43	0	1	1
TLK1	-0.822884924	0.569764506	1
GPR137B	1.120905604	0.340879933	1
INCA1	-1.093796413	0.2298977	1
FNDC1	-1.086768797	0.500628503	1
FGFRL1	0.04839923	0.976434224	1
RNASEH2B	-0.842450404	0.554766426	1
FADS3	-1.172993954	0.260241732	1
ORC2	0.51567719	0.637558133	1
DPH5	-0.116131933	0.911220144	1
URI1	1.001543053	0.442346593	1
PAEP	0	1	1
C5H12ORF4	1.559216356	0.217373816	1
ABCA6	-0.282205984	0.843640989	1
ORAI3	-0.019994213	0.986555126	1
MZT2B	2.087973906	0.089598437	1
CAV2	-0.827392954	0.436599617	1
ZNF862	0.705524612	0.543497218	1
ACVRL1	-0.368867391	0.663218975	1
RARG	3.042544123	0.417375227	1
MPDZ	0.283325507	0.884635078	1
LMBRD2	0.979681114	0.575353882	1
PPP1R11	-0.255426231	0.793119631	1
TNIK	-0.705338708	0.753853583	1
PDE12	-0.0887367	0.928859549	1
MYO9B	1.055840083	0.691339109	1
SLX4	0.090911156	0.924342658	1
HAUS7	1.685876322	0.141576235	1
URM1	-0.207471974	0.885046737	1
P2RX5	-0.180047813	0.908762234	1
ETV6	0.820280892	0.368535001	1
L2HGDH	-0.758711751	0.469427814	1
SLIT3	1.309089153	0.2269943	1
ENSMMSG00000024247	0.068791991	0.965285571	1
ETFRF1	-0.393453327	0.697503016	1
SNRNP70	1.445723606	0.285395749	1
PROSER1	0.788732911	0.502677828	1
CPOX	-0.189006574	0.853643128	1
SPG11	0.464470986	0.791885497	1
GMEB2	-0.40051475	0.804984255	1
DNAJC25	0.076579034	0.936378054	1
MICU3	0.402309917	0.800517817	1
YWHAQ	1.947899653	0.121786156	1
FOSL2	-0.002942475	0.997946429	1
G3097	0.285070273	0.71415444	1
TREX2	1.365569012	0.327327687	1
SLC24A3	1.29268823	0.360726612	1
SAP30L	0.171841135	0.886305646	1
SENP7	0.287507102	0.856301614	1
TMEM106B	1.169275173	0.493245944	1
MPPE1	0.184869447	0.87217758	1
DSB	0	1	1
ENSMMSG00000016081	-0.687175772	0.657985516	1
MPHOSPH9	-0.127928646	0.896434073	1
NDUFAF1	-0.682055423	0.520196431	1
MX2	1.416572427	0.336597582	1

INPP1	0.816244249	0.428163484	1
TIMM22	0.017733163	0.981891469	1
METTL1	0.898725558	0.400947847	1
COPS7A	-0.361155196	0.803156541	1
ZNF768	-0.586978703	0.707126042	1
TBC1D23	-0.538353211	0.707000473	1
COL14A1	-0.224882927	0.813192672	1
SLC28A3	0	1	1
CYP26C1	1.345310671	0.300137927	1
SPATS2	-0.48257363	0.618290096	1
ADIRF	1.059899058	0.391608955	1
FAM214B	-0.013460536	0.993206	1
PYCR1	0.679136718	0.552584797	1
DGUOK	0.178016771	0.860709183	1
USP48	-0.087794181	0.951898709	1
UBTD1	-0.589073329	0.624596935	1
SHQ1	0.045460891	0.948765458	1
OSBPL10	0.211825408	0.888345459	1
FAM174B	1.509484625	0.263442454	1
RXRG	-0.110614518	0.941293918	1
EPB41L4A	1.024896454	0.29422576	1
ENSMMSG00000000414	-0.11827255	0.914441703	1
CENPV	0.228020016	0.84671972	1
SULT1A1	0.577292753	0.661856243	1
TTC9	-1.160194827	0.360879665	1
DGKD	0.020718681	0.989461674	1
ZNF74	-0.552756787	0.509859093	1
CBX7	-0.621046691	0.607572494	1
TBL1X	1.227084911	0.490310412	1
TAFAZZIN	-0.370290165	0.707064324	1
SH3BGRL	0.692906268	0.646992852	1
THUMPD1	0.90965089	0.577678667	1
G1610	2.053694094	0.102128881	1
B4GALT4	1.162110394	0.477405862	1
PURB	-0.464572843	0.622199528	1
WDR59	-1.992306809	0.630066683	1
APBB1	-0.073012628	0.954004634	1
EMC1	0.273056704	0.775678154	1
ENSBTAG00000048670	0.828011858	0.498985124	1
IFT74	0.859428531	0.599953787	1
SCFD2	0.444403797	0.772879391	1
NKAP	0.615205126	0.703846476	1
FBXL20	0.57787733	0.576312878	1
DUT	0.840141064	0.41836807	1
KANSL2	0.636208035	0.698962563	1
QSER1	-1.038835319	0.478724059	1
TMED1	0.348289392	0.785258938	1
FAM98C	0.225949541	0.839744966	1
ENSBTAG00000013305	0	1	1
EPN2	-0.703965223	0.638200683	1
RMND1	0.899922789	0.397292231	1
ENSMMSG00000002971	2.38884844	0.098023449	1
TCERG1	1.037624451	0.625167544	1
NUP43	0.407930058	0.722618	1
ENSMMSG00000006349	0	1	1
EGFLAM	-0.837314024	0.541166332	1
MON1B	0.72367398	0.546440354	1
NPRL3	-0.208670582	0.852919873	1

ARHGEF15	-0.623922632	0.410845583	1
MAPKBP1	0.368010084	0.665847816	1
TRNAU1AP	0.405224228	0.717507774	1
NUS1	0.173424205	0.858647607	1
ALOX12	0.95194342	0.348482776	1
CDK14	1.693656243	0.172361828	1
TMEM14A	1.282463671	0.174663262	1
RBPMS	0.10428348	0.924287198	1
RHOA	0.443180068	0.77372343	1
YARS2	-0.372636933	0.688794103	1
RUSF1	0.354598058	0.718596373	1
TBC1D7	1.24664455	0.304423018	1
KCTD9	0.236613876	0.879869501	1
ZBED4	-0.25711564	0.822530006	1
COL13A1	1.719746942	0.363613933	1
ADAM19	-0.845831077	0.533160208	1
POMGNT2	-0.217602868	0.849122713	1
ZFAND1	0.304789438	0.793507128	1
MKKS	0.061273128	0.950513525	1
TRMT13	1.035036098	0.53643348	1
CBLB	0.566161061	0.617106973	1
MRPS31	-0.286250448	0.826555542	1
NT5DC1	1.363572766	0.34609257	1
HPRT1	-0.288696461	0.845444362	1
GCC1	-0.190199209	0.86135013	1
G12595	-0.589308562	0.696237913	1
PSMG2	-0.573935861	0.523773791	1
DPYSL5	0	1	1
TLE4	-0.536803863	0.678913843	1
PPFIBP2	-0.806750197	0.393746284	1
STARD13	-0.613966295	0.700909494	1
PDE6A	2.753802084	0.101736715	1
LNX1	-0.48374476	0.627488698	1
B4GALT5	1.058382096	0.542446544	1
SIAH2	0.626516558	0.465179326	1
NEDD4L	-0.510328792	0.742459099	1
BAHD1	0.631885026	0.711104057	1
TSHZ2	-0.057735055	0.945337587	1
IFI35	1.908695503	0.148315772	1
FRMD8	0.464046509	0.76074366	1
GLS	0.721545381	0.59044742	1
NTHL1	0.682193747	0.48117042	1
RICTOR	0.990887064	0.463885158	1
CCDC115/LOC614882	0.267447741	0.792288351	1
TESK2	0.495503749	0.748997031	1
PTPRK	0.856969749	0.837055363	1
ZBTB48	0.975503278	0.341036748	1
RPRD1A	0.578533249	0.613468639	1
GALT	1.046979854	0.397769599	1
SMG7	0.681867652	0.651649992	1
L2HGDH	-0.695511862	0.64925197	1
SIX2	-1.028871446	0.522676584	1
ENSMMSG00000011269	-0.38882292	0.804750741	1
NOP16	0.055767177	0.946911748	1
TCERG1	0.007527834	0.995119367	1
MEIS2	0.939551644	0.502394596	1
P3H4	1.586708612	0.200652994	1
ENSMMSG00000008909	1.086659741	0.42765324	1

FAM189B	-0.394636941	0.793362258	1
GAPDHS	1.527556418	0.234519282	1
TMX1	1.465888648	0.198977191	1
RAI14	0.896671868	0.628177511	1
GSTM1	-0.911559198	0.355185519	1
TDP1	0.247684436	0.795956403	1
RRS1	0.166978783	0.877550268	1
COL5A1	0.562340684	0.614816152	1
RASGRP3	-3.732141544	0.370238952	1
ACVR2B	-0.006545327	0.996295599	1
LEPROTL1	0.501613861	0.649269501	1
MYO1D	-0.273726935	0.74482727	1
ZNRF3	1.867383209	0.170735807	1
TM2D3	0.013582715	0.989071437	1
FOXN3	-0.430722828	0.722091374	1
SRGAP2	-0.426726871	0.674411774	1
CFAP276	0.413021564	0.704617231	1
INTS6	0.187877888	0.855430473	1
TRIM24	0.464531028	0.737630456	1
BCL6B	-1.048356533	0.275016842	1
PDZD8	-0.076622202	0.941197156	1
OGT	-0.222662833	0.891227102	1
KHDRBS3	-0.139626347	0.888102602	1
NEDD4	-0.418010017	0.784191994	1
DLL1	-0.236740588	0.778468108	1
OSTM1	0.407066993	0.635279817	1
USHBP1	-0.215842257	0.81139116	1
ZNF444	-0.017666894	0.98726904	1
LRRC14B	-0.900395563	0.553314661	1
RNF214	1.753934982	0.316843492	1
TARBP1	0.045173123	0.961834403	1
VGLL4	0.643364037	0.566690768	1
RPS6KA1	1.286286802	0.205529565	1
P3H2	0.928898887	0.367233244	1
WFDC2	-0.126971979	0.922137933	1
GOSR1	0.020640072	0.989524082	1
GPC3	0.443525453	0.680786728	1
TGFB2	0.002150845	0.998926391	1
PKDCC	-0.803115847	0.401421506	1
KCTD9	0.255758289	0.770122619	1
GLMN	1.127560493	0.374387185	1
NF1	0.690358527	0.48691268	1
ZCCHC24	-0.896232588	0.559851072	1
MAFG	0.174782003	0.892526013	1
GRIP2	-0.217748177	0.885722235	1
P4HA2	-0.721020441	0.401695402	1
CRTC1	0.944133921	0.372666699	1
COA1	-0.748624728	0.416871401	1
ALG14	1.619121444	0.123609914	1
CNOT9	-0.328313079	0.729613701	1
CHAF1A	0.256528513	0.812993365	1
MTFP1	-1.029863317	0.267520903	1
PLXDC2	0.52091762	0.541880193	1
TATDN1	0.139292717	0.867841779	1
IL17REL	3.822896758	0.10412754	1
RRP1B	1.350704195	0.277311964	1
BRINP2	-1.88003802	0.408926721	1
MIEF2	-0.205776476	0.851430308	1

TBC1D2B	-0.690992703	0.656849529	1
PTCD2	-0.198464236	0.899923774	1
SRPK2	0.246031189	0.805468937	1
STIM2	0.038719834	0.978340321	1
MIDN	0.13302206	0.887132068	1
AAR2	1.327038139	0.305876184	1
PTCH1	0.219254094	0.848232048	1
DERPC/CHTF8	0.40713255	0.676348166	1
SMYD4	0.403445908	0.806368421	1
CCDC32	0.122156938	0.918601144	1
MPDZ	-0.521870285	0.728975492	1
KIAA1109	0.890634221	0.574859549	1
FBLN2	-0.454330081	0.707103751	1
GTF2H5	0.101282614	0.909961927	1
TUBGCP2	-1.829130055	0.407686856	1
SMIM12	-0.659355633	0.553532796	1
DOCK5	1.563737207	0.314578737	1
ZNF202	1.857332281	0.191755781	1
HDAC4	0.425850686	0.750284792	1
TXLNG	0.86879839	0.604590283	1
SCAF4	0.16645989	0.850402278	1
SNUPN	-0.380093848	0.763485922	1
VAMP3	-0.460532985	0.610902425	1
EPHA2	0.006205977	0.994980092	1
HSPBAP1	-0.377919224	0.809112897	1
TAF5L	-0.66211623	0.538607	1
ARMC5	0.603173319	0.559809981	1
TXNDC12	0.125243769	0.894843017	1
FBXO25	-0.015505512	0.991800115	1
PANK2	0.984532971	0.437015728	1
CLUAP1	1.594617577	0.35865611	1
DEF6	1.426792957	0.181419322	1
PHF8	0.333015285	0.730746856	1
GPC4	0.901160135	0.523339797	1
XYLB	0.88033964	0.380287212	1
INVS	0.881944294	0.420365807	1
MCC	0.239489539	0.840365258	1
CTTNBP2NL	-0.087046275	0.920744062	1
TSTD2	1.039983716	0.545611758	1
HEATR5B	1.704322727	0.315053316	1
LPCAT1	0.985113495	0.376390212	1
KTN1	0.279842642	0.873306414	1
CISD3	0.06274437	0.948676983	1
JOSD1	-0.46321075	0.7593696	1
SLC35A5	1.238370057	0.36420379	1
SLCO3A1	-0.875601383	0.539161306	1
SET	0.18485474	0.906162772	1
STEEP1	-0.015275671	0.983817682	1
LYPD5	0	1	1
DNAAF9	-0.099307766	0.919131996	1
KIF27	0.655860982	0.671186733	1
PPARGC1A	-0.519742348	0.732723678	1
CACNA1H	0.600879396	0.579985576	1
TRAPPC13	0.573886479	0.766828962	1
SLC39A11	2.208913225	0.131793914	1
STYX	0.076937991	0.936294265	1
PRRX1	0.451275435	0.799543031	1
ENSBTAG00000007093	2.132029445	0.0896973	1

LIMS2	-0.441563119	0.717476973	1
SCRN2	0.768891151	0.540655027	1
IL18BP	1.433187081	0.116821261	1
EPG5	-0.002738595	0.998725776	1
SLC17A1	0	1	1
SCNN1G	0	1	1
FAT4	-0.190799797	0.897797662	1
VAMP7	-0.084410095	0.956091292	1
INPP5E	-0.422352569	0.706739723	1
LOC107131134	0.467607429	0.566049718	1
CDC45	0.983889427	0.403393125	1
PCGF1	1.09416092	0.348969464	1
CTIF	0.179638397	0.903654147	1
SEMA6A	-0.748713818	0.487726477	1
PURA	-0.342003169	0.835972691	1
ARHGEF12	0.941484709	0.59125706	1
SLC16A5	-0.664279061	0.512179679	1
MED13	-0.068263653	0.95292665	1
MAPKBP1	0.338294084	0.76206544	1
DNAAF10	0.288824752	0.807976526	1
DCTN5	0.144781944	0.904546214	1
TXNDC17	-0.225610887	0.834328431	1
MED13	-0.587032863	0.697146902	1
LOC513508	0.273904933	0.805864422	1
FAXC	0.0848191	0.939762382	1
GRK5	-0.591922698	0.520986923	1
PML	1.401603428	0.335754046	1
NUP210	-0.197460336	0.834218676	1
FBP1	0.737605611	0.478662152	1
CEP43	-0.239353731	0.901799895	1
TM2D1	-0.344034067	0.820191892	1
PHOSPHO1	-0.988030727	0.432061288	1
RABL3	0.063798024	0.966661186	1
RPTOR	-0.158949357	0.87719514	1
ARHGDIB	-0.520871743	0.598286793	1
C2	-0.526079138	0.605694504	1
PHYKPL	-0.234799445	0.797775662	1
BICRAL	-0.645870002	0.67871281	1
USP54	0.18551559	0.882812965	1
AMT	0.039095097	0.975377668	1
PDLIM1	0.913482604	0.527692832	1
RILP	-1.04826173	0.473302359	1
B4GALT7	-0.043550526	0.979658856	1
G33533	1.803404073	0.217461263	1
TEX10	0.941676919	0.459197155	1
PRKCH	-0.438522555	0.655859874	1
ENSBTAG00000040559	1.345499547	0.175771156	1
ATG12	0.047438476	0.965897812	1
KLF1	0.192981401	0.795030827	1
NUMB	3.25860721	0.083624765	1
ARHGAP24	0.308482376	0.859513421	1
METTL16	-0.30559781	0.750721754	1
E	0.010681627	0.990400678	1
BUD13	0.672616493	0.544311315	1
LIN54	-0.268264263	0.887395368	1
SMPDL3A	-0.645772704	0.661356888	1
RPS23	1.739611999	0.2114704	1
MGAT2	0.662851342	0.573037162	1

SLC37A4	-1.120046767	0.453622171	1
KIAA1549	1.154225215	0.345632617	1
PUS7	0.197286234	0.904000948	1
CCHCR1	1.522854998	0.170403977	1
VGLL2	-0.128488058	0.938146564	1
SLC25A15	0.065379836	0.953129914	1
POGLUT1	0.545780813	0.544109866	1
ATG14	0.586654419	0.722930751	1
TMEM230	0.247937731	0.868365489	1
TMEM209	1.38533116	0.396723421	1
FIG4	0.324425306	0.756169402	1
INPP4A	-0.102726799	0.932948471	1
ZNF277	-0.393332168	0.619410482	1
C16H1ORF115	0.507183841	0.617785633	1
CEBPA	1.193394786	0.316867529	1
CELF2	-0.670425693	0.524314558	1
SMC2	0.854528655	0.566474658	1
NT5C3B	0.424661204	0.66130408	1
ERAP2	0.46016739	0.714908251	1
RBM4	-0.019260612	0.979613374	1
MAP3K7	-0.56953847	0.714331636	1
CYLD	0.688766805	0.62776652	1
MTFMT	-0.290084963	0.770439582	1
CLCC1	0.197839096	0.900986251	1
HACD1	-1.026459969	0.469983056	1
N4BP3	0.89619521	0.465723278	1
ASS1	-0.416828449	0.695365958	1
ZNF526	-0.180287138	0.838695412	1
ITPK1	0.086276196	0.940104728	1
MED31	-0.322939782	0.726319205	1
SLC37A1	0.881879092	0.395389553	1
BTBD7	-0.203427845	0.811698277	1
GALNT7	0.334305278	0.831794526	1
SIRT1	1.14761435	0.350968957	1
DUSP11	0.4995704	0.680394278	1
TGDS	2.315429555	0.098826875	1
TWSG1	0.282872432	0.775729633	1
NR3C2	-0.262280713	0.869695771	1
SKAP2	-0.289756749	0.792682682	1
PPIL1	0.509032142	0.623299411	1
KLF11	0.268529115	0.866149335	1
MRTO4	-0.47280408	0.563353432	1
DEPDC5	-0.209636139	0.903314629	1
BAHD1	1.106365033	0.414591721	1
PSMD9	0.189435906	0.847172219	1
FXYD5	0.747418684	0.564643909	1
PRICKLE3	0.5399889	0.614415326	1
STK19	0.226256734	0.87442404	1
TM2D2	-0.477899354	0.64366634	1
SEMA3B	1.615131799	0.09817723	1
PTK2	0.499039374	0.676697111	1
SMAD3	-0.883467878	0.417270208	1
TENT2	0.470129974	0.755971409	1
ENSBTAG00000030608	1.627704923	0.212703991	1
BRAF	0.483563214	0.70373056	1
RDH13	-0.255820555	0.785554086	1
LRP11	-0.106573788	0.908035495	1
ELF2	-0.256763584	0.8665915	1

RABGAP1L	1.174801682	0.448617601	1
CABIN1	-0.480718471	0.817065348	1
PLEKHG1	-0.540597943	0.614824764	1
EXOC6B	0.492726999	0.677051841	1
SLC35A4/LOC112447523	-0.367865266	0.805185976	1
PHC1	0.441857412	0.690896643	1
EIF1AD	1.765300393	0.128046084	1
FAM120C	0.939985467	0.475699174	1
JKAMP	0.977094721	0.404178422	1
LRFN4	0.635567501	0.568667673	1
FZD1	0.789710473	0.446159185	1
PDE4B	-0.943320026	0.529559614	1
RNF44	0.255062271	0.800239409	1
SLC12A9	0.827202408	0.413215779	1
HDHD5	-0.363180129	0.721497941	1
PAPOLG	0.472359612	0.684938229	1
PRMT9	1.155679592	0.359911174	1
MRPS14	-0.180462946	0.862377736	1
TMEM150C	0.821906696	0.454748964	1
B4GAT1	-0.301881854	0.795070071	1
BAX	0.858986622	0.427122916	1
BLVRA	1.639209685	0.150063832	1
SP2	-0.176947819	0.913814224	1
PKIG	0.721868653	0.626794066	1
SLC30A7	1.516974936	0.184837141	1
ARL8A	-0.036560933	0.974117491	1
IKBKG	0.745662215	0.649293694	1
BAHCC1	-0.195268116	0.83565807	1
C15H11ORF71	1.887315708	0.168095441	1
MAP4K5	-0.16778884	0.863601685	1
CITED4	1.855638975	0.161351417	1
MEX3C	1.186761414	0.429382223	1
CYB561D2	0.975084216	0.315372819	1
AFF3	4.952680314	0.262369731	1
CDC27	0.523382921	0.757815504	1
ENSMMSG00000008728	2.570543082	0.25557087	1
MICALL1	0.125254015	0.91072592	1
PIP5K1C	-0.324048892	0.765102778	1
STK38L	0.75895935	0.564333257	1
LOC112445119/TCEANC	0.026470654	0.975769586	1
TFPT	0.791930022	0.526578321	1
G35990	0.369461761	0.678376551	1
PAM	-0.099111291	0.949698839	1
TP63	2.78345598	0.254936807	1
SUSD6	0.651066094	0.559268549	1
CRTC2	-0.222600048	0.803419851	1
CPT1A	-0.690115255	0.631058295	1
MORC4	1.641756033	0.331174214	1
DDX52	-0.167512078	0.879893748	1
DR1	-0.261205672	0.863011059	1
ATPAF1	-1.164834439	0.410162677	1
MAGIX	1.126387681	0.327354953	1
DLL4	-1.097709837	0.275017723	1
CLCC1	-0.066611756	0.947030501	1
VSIR	-0.066697623	0.932700239	1
GNA13	0.024402736	0.981659079	1
PMP22	-0.792105366	0.579122492	1
SH3TC1	1.127463582	0.331526289	1

CCDC127	-1.041007752	0.471405591	1
MAP3K14	-0.508044574	0.663196719	1
HELZ	-0.549400041	0.803764761	1
CHRNE	1.879223455	0.288012729	1
POP1	0.877478204	0.383185084	1
THOC3	-0.386695091	0.622360132	1
G30177	0	1	1
PYCR3	0.199950895	0.858445808	1
SLC25A26	-0.800845538	0.386786701	1
BET1L	0.720174035	0.497838571	1
AKT1	-0.259404747	0.810408871	1
RUFY2	0.029018319	0.982947096	1
STN1	0.628808758	0.630627654	1
KRT222	0	1	1
RRM2B	-0.759393787	0.598262925	1
FBXO22	-0.1372181	0.891669893	1
ODF2L	0.822945706	0.448038894	1
ABI3BP	-0.60743174	0.69475035	1
TEAD2	0.374933953	0.771810794	1
INSR	0.22457052	0.903186306	1
CEP162	0.640510597	0.684919666	1
PDS5B	-0.463959519	0.763988478	1
OAS1Y	1.610839128	0.306057918	1
MIIP	1.149532557	0.395703504	1
CYP11A1	-0.672746605	0.497071124	1
C16H1ORF174	-0.505517294	0.596526	1
RNF41	0.579843405	0.724961809	1
POGZ	0.567271758	0.77784726	1
AUH	0.54736168	0.749719884	1
CLOCK	-0.230820212	0.816860053	1
UBN2	-0.095980407	0.923188303	1
G5790	-0.232200113	0.795100867	1
INTS14	0.802673953	0.453737176	1
METTL14	1.429003908	0.279562853	1
CDC23	-0.19652051	0.898538645	1
CLCN6	0.239816873	0.831253591	1
IMPA2	1.8891662	0.199213676	1
STXBP5	-0.497669134	0.624280631	1
BRMS1	0.082547268	0.930818106	1
NCOA3	0.038962562	0.972382243	1
ZNF853	-0.318862794	0.849559972	1
CDK12	-0.072438394	0.941964711	1
ZNF131	0.03152198	0.982178646	1
C9H6ORF120	-0.059511036	0.956191912	1
POP5	-0.328078474	0.717250465	1
G15450	-0.480500058	0.59939545	1
SLC44A1	-0.139339579	0.891372773	1
GPSM1	0.955153908	0.402041856	1
STKLD1	0.936525308	0.391747385	1
NKIRAS2	-0.417635581	0.712829565	1
MLH3	-0.138478373	0.883878003	1
LOC515551	0.386510638	0.801581298	1
TCP11L2	0.469439886	0.717666666	1
TBC1D10C	-0.439683709	0.671267752	1
UFM1	0.419735064	0.595537115	1
MME	0.472392623	0.6850948	1
UBE2L6	-0.447187695	0.741042158	1
PPARD	-0.309731548	0.751164038	1

IFIH1	1.49229184	0.280998314	1
DDX20	-0.321230456	0.825047583	1
PTGFRN	0.647037539	0.564362798	1
SPATA13	0	1	1
SENP2	-0.429162317	0.778474235	1
NDN	0.065404912	0.961627422	1
MARCKS	-0.328362548	0.765787582	1
RSPH3	0.970618132	0.242265779	1
MAPT	-1.737643078	0.429815113	1
NPRL3	0.041516702	0.971244276	1
TP53INP1	1.059776901	0.567866582	1
SAE1	-0.297356913	0.844405418	1
PDCD11	0.201122567	0.85649525	1
COQ10B	0.315040144	0.771593229	1
GIPC1	1.193266697	0.33441565	1
ODAD3	0	1	1
WDR70	0.639620555	0.599438458	1
PIM1	-1.040264984	0.349909823	1
ENSMMSG00000009952	1.590691625	0.252796289	1
AHR	0.429705176	0.66953285	1
MIX23	-0.336005788	0.773375924	1
TEAD1	0.232598796	0.875727287	1
AAAS	0.969079259	0.420253114	1
DTNA	-0.340294759	0.857282406	1
FBXO21	-0.189509183	0.834164228	1
SGMS1	-0.558221196	0.589351675	1
BSCL2	1.182602771	0.31858461	1
FBXO8	0.327569995	0.787753514	1
PLSCR3	1.498914348	0.240189319	1
MMS19	1.240257826	0.49036696	1
PRELP	-0.180341772	0.856759197	1
CELSR3	1.494769406	0.330454319	1
SNRPB2	0.601421708	0.62754072	1
CBX2	1.025112831	0.374478694	1
PLEKHG3	-1.184978014	0.776355158	1
LIN7B	-0.195748843	0.853603535	1
DDR2	-0.365460534	0.706158126	1
JMJD8	0.774961015	0.510586172	1
ENSMMSG00000006731	-0.014368714	0.989924888	1
ASB6	-0.080436359	0.939974756	1
STEAP3	0.023269267	0.987863695	1
THBS2	-0.691654533	0.512828804	1
PDCD2	0.414527001	0.649228101	1
TPCN2	0.713227589	0.440684921	1
TMEM110	2.348812506	0.090200642	1
DIMT1	0.090685697	0.931134594	1
NADK2	-0.182587482	0.818738117	1
ING3	0.508611972	0.638982645	1
RPP40	0.850750216	0.352280545	1
GATA3	2.239303426	0.246758084	1
UBOX5	-0.399303753	0.740618795	1
TRMT6	-0.548793976	0.569600882	1
DNAJC1	0.400542764	0.679771147	1
HMGXB4	0.3993429	0.699428057	1
UPF3B	-0.428500414	0.835185142	1
SNX15	-0.232741095	0.820465922	1
DENND2A	-0.213714925	0.819994107	1
PGAP4	0.215528763	0.886396925	1

SPOUT1	0.096152363	0.901977155	1
FAP	-0.35586646	0.724896109	1
NUP50	1.081437381	0.310783238	1
ENSMMSG00000022020	-0.237465835	0.794268126	1
ODAD2	3.836415439	0.10686383	1
UBAC2	0.455616477	0.745195673	1
ADPGK	2.273546963	0.11579955	1
ARL5A	-0.146568814	0.88241145	1
ICE1	1.388907171	0.152727861	1
SLC2A3	-0.955450612	0.507304753	1
RFX1	-0.060927008	0.958321703	1
PSMB10	1.527801912	0.199206858	1
BMP1	-0.191201737	0.817572162	1
GOLIM4	1.644271091	0.308351185	1
TRAPPC2L	1.814701047	0.12092116	1
GGACT	0.597960899	0.597097109	1
G8469	0.784990139	0.515416863	1
VSTM2A	-1.807641631	0.691405338	1
STARD9	0.032611845	0.977833732	1
RGD1307554	-0.23194027	0.851163559	1
ECHDC1	0.132588372	0.922043624	1
RBMS2	-0.559238201	0.552080944	1
NCK2	-0.501485877	0.598108164	1
PKMYT1	1.584668692	0.12710251	1
TRIQK	0	1	1
AHSA2	1.094028863	0.779369786	1
GTF2F1	-0.224368028	0.839391362	1
WDR45B	1.349368635	0.266485454	1
ZNF579	0.97146902	0.409375874	1
COL4A3	-0.35751507	0.721757668	1
TMEM101	-0.312758741	0.774768493	1
POLR2D	-0.119672159	0.882579153	1
COPZ2	1.515261226	0.21341164	1
MIOS	0.842614834	0.525781661	1
COASY	-0.714906813	0.608790908	1
SAP25	1.267897246	0.31246087	1
NAA30	-0.436927663	0.698933022	1
ODF2	0.248339497	0.868009868	1
RREB1	0.233451837	0.955310419	1
FNBP1	1.707092839	0.248378279	1
DCP1A	0.000393041	0.999696175	1
SLC25A12	-1.092319124	0.438211411	1
PLA2G15	0.682010432	0.698234712	1
KIAA0232	-0.114873299	0.934729802	1
SYNJ2BP	-0.940704029	0.505336974	1
PRPF39	-0.025606043	0.986681027	1
GTF2H3	0.130922078	0.911431356	1
HEXIM2	0.68577541	0.407703227	1
ARL6IP6	0.571356509	0.601050151	1
FBLN1	0.350796557	0.681396649	1
RUBCN	1.238268601	0.348344842	1
GAS1	-0.320280584	0.838413663	1
TAF5L	-0.666799502	0.399237007	1
UNC119B	1.086316004	0.477883432	1
CCDC59	-0.243465163	0.821157287	1
ENSMMSG00000016969	0.496039695	0.709408561	1
FAM171A1	1.034987183	0.494133694	1
FANCI	0.894211367	0.396899675	1

SUCO	0.327301676	0.834373974	1
SMARCD1	0.851623189	0.510819097	1
CD14	0.376019358	0.7169216	1
B4GALT7	0.336917321	0.81584608	1
ELK3	-0.371745551	0.802006752	1
LOC534155	1.136025422	0.288043281	1
AGK	-0.06063841	0.945640205	1
CHTOP	-0.225856901	0.877196034	1
PXMP2	1.002428578	0.453162111	1
WIPF3	-0.944294083	0.383815675	1
ISG20	-1.875095431	0.394952596	1
MARCHF9	1.454525386	0.282279767	1
GTF2E1	0.31825162	0.847183929	1
NAA16	0.305190499	0.749128932	1
PTPRT	6.113875547	0.147139711	1
ASL	1.626095732	0.168632228	1
SNX12	-0.029674381	0.974536158	1
BCL9	0.807494879	0.538504356	1
ZSCAN29	0.45382027	0.648873577	1
HDHD2	-0.501075728	0.57958017	1
CLIP2	0.059668812	0.952889634	1
RFLNB	-0.487368635	0.607504066	1
DMXL1	-0.59003423	0.703798494	1
RGCC	-0.919565941	0.41810198	1
YIPF7	-0.422166259	0.755640203	1
DNAAF11	-1.875646515	0.396500129	1
TCEAL1	0.717246032	0.609237367	1
GMDS	1.793568042	0.096104003	1
TMEM225B	0.422539854	0.699168487	1
DNASE1L1	-1.11392537	0.789114265	1
CGRRF1	-0.605452507	0.48802151	1
BORCS7	0.559339749	0.656043344	1
PLIN5	-0.031740781	0.983417638	1
GUCY2C	1.541281798	0.243876132	1
DYNC2I2	1.090871131	0.259068821	1
LRRC30	-1.271641021	0.419703786	1
ITGA9	0.746347041	0.662647806	1
GRAMD2B	1.733664216	0.193219437	1
CETN3	1.008645737	0.400335807	1
GLT8D2	0.094363832	0.917179273	1
CARD9	2.17229433	0.123002686	1
GOSR2	0.558360466	0.552527498	1
MRM2	-0.068466964	0.939960369	1
SYT9	3.05049856	0.133921518	1
RELL1	0.827518659	0.546427861	1
SLC9A5	0.645236049	0.631786243	1
FOXP1	0.365654882	0.824114895	1
C2CD2	0.782223812	0.480687275	1
CCNL2	1.344961942	0.231528315	1
TWF1	1.271115979	0.388233451	1
LOC785873	0.641785261	0.452329722	1
CDH11	-0.326600101	0.732111779	1
METTL13	-0.206854501	0.828443498	1
PKN3	-0.209798201	0.820150032	1
STK17A	-0.455957883	0.762380535	1
RNF26	0.741920862	0.62473975	1
L3MBTL3	-0.661295406	0.476039073	1
ATP1B3	0.168593364	0.839066958	1

PLEKHA3	-0.353107873	0.803920617	1
MGAT4A	1.394061923	0.292523209	1
PARP4	0.406237047	0.715516096	1
KATNIP	0.128070472	0.933807699	1
DAPK2	-0.471751147	0.758744936	1
MOCS1	0.631606697	0.578890289	1
IFNGR1	0.117753272	0.942058518	1
GFER	0.288353877	0.772498284	1
C8H9ORF64	0.532744802	0.734867317	1
UNK	0.805035274	0.534929547	1
SLC9A3R2	-0.43054586	0.624861316	1
SEPTIN5	-0.093072042	0.926671414	1
RNF141	0.538767395	0.625879527	1
ZNF524	0.283922456	0.78175264	1
TMEM39A	0.739022176	0.516819512	1
CD109	0.329389066	0.673961673	1
TREX1	0.499117817	0.666220288	1
PDRG1	-0.050811527	0.96149181	1
ARL3	-0.524593107	0.627900828	1
ARMC6	-0.048053809	0.966892679	1
LTO1	-0.087073855	0.92702086	1
TTC5	0.555586008	0.679877334	1
LHPP	-0.778239099	0.474329085	1
RTF2	0.234489791	0.84560202	1
METAP1D	-0.710372384	0.418102565	1
LMBR1L	-0.641805389	0.42227356	1
ZNF326	0.430601618	0.710810885	1
NOD1	0.590444609	0.660033847	1
SPINK7	0	1	1
ADO	-0.426763419	0.730590497	1
GMIP	0.432606431	0.678442292	1
ACKR3	-0.886173525	0.561978429	1
TSEN2	0.010432726	0.992095755	1
SPRED2	0.268547022	0.80323205	1
CIC	0.698550766	0.764489576	1
PRIM1	1.282773067	0.307824242	1
FAM174A	-0.852084178	0.324462085	1
AK6	-0.280399532	0.78384598	1
SLC25A38	0.686252413	0.58685875	1
SCARA5	-0.605879851	0.55002926	1
SLC9A1	1.059158305	0.436915897	1
CTSS	0.177128105	0.869068673	1
TCF12	-0.073641909	0.972142547	1
AKAP11	0.003505006	0.997239923	1
HECTD4	-0.1410144	0.928379997	1
MZF1	1.333738194	0.202486495	1
RAP1B	-0.048867137	0.965304602	1
NKTR	1.566387039	0.233866318	1
DCPS	0.485606401	0.659712265	1
RAPGEF2	0.581424095	0.88901528	1
MRPS35	0.322193375	0.801739902	1
ERCC4	-0.09884166	0.931694742	1
PDE2A	-0.737671294	0.596980176	1
CSF1	0.054583509	0.957209371	1
LYNX1	1.701774765	0.251231747	1
ICOSLG	1.214343683	0.283187209	1
ANKRD13C	-0.036665074	0.968803696	1
NUDT17	0.496200487	0.673786221	1

ENSBTAG00000012019	-0.392017505	0.772146398	1
PROCA1	0.731930767	0.434734677	1
RBMX2	1.637162264	0.208863312	1
TMEM41B	0.20370962	0.828379676	1
ZC3H12A	-0.085010642	0.94151834	1
FANCL	-0.240232114	0.877748733	1
SRP9	-0.554879859	0.71009083	1
CD276	-0.553092916	0.585785515	1
KASH5	2.307855826	0.259796362	1
UBE2N	-0.305840345	0.759852235	1
PARP16	-0.436592492	0.719973324	1
DALRD3	2.002582631	0.102281218	1
PPP2R3C	-0.444879072	0.607644828	1
OSBPL6	-1.024696288	0.610372083	1
WDR37	-0.181190091	0.844585217	1
TOMM22	-0.238032152	0.809106343	1
HHIPL2	0.627520946	0.547176368	1
BOC	-0.403815981	0.623581475	1
PGGT1B	-0.169353808	0.87734589	1
ATG16L1	-0.586783296	0.675073975	1
RIN2	0.355291621	0.8120981	1
REPS1	-0.333398723	0.828785074	1
E2F6	-0.413584074	0.644930337	1
SLC39A11	1.674997731	0.288818392	1
FAM98B	0.442505592	0.716419535	1
RC3H1	-0.80061827	0.505411718	1
TRMU	0.600601961	0.582201844	1
MOV10	0.680274568	0.546177789	1
KIAA1671	0.567681064	0.602193209	1
WDR77	-0.572929307	0.695946096	1
GSE1	-0.693770267	0.752012069	1
RCL1	0.977467588	0.456571287	1
MARVELD2	1.133699436	0.410695693	1
ENSMMSG00000002705	-0.240153314	0.879633385	1
TRADD	-0.145658338	0.893846202	1
IL17RA	1.338241102	0.229722723	1
KHDC4	-0.337768668	0.825205789	1
RAB11FIP5	0.556334702	0.68068001	1
H2AX	0.966099707	0.378681151	1
SCARF1	-0.87277166	0.296233667	1
SLC25A32	-0.633146179	0.662558786	1
ABRAXAS2	-0.225733601	0.827613888	1
DYNC2H1	0.492407363	0.626122214	1
PRKAA2	-0.832277755	0.41326254	1
SMC4	0.349883666	0.76059335	1
ZFAND3	-0.918900308	0.279940825	1
AKIP1	-0.128045867	0.898509124	1
ZC3H12C	-0.829857111	0.574002679	1
POLR2H	0.272280735	0.77470598	1
INTS2	0.428856527	0.707635527	1
ZC3H6	1.229027275	0.481348276	1
PPM1M	0.1426201	0.88877803	1
DENND1A	0.496538995	0.769586869	1
RAB6A	-0.382322948	0.814307235	1
CSTF1	0.69632772	0.547248632	1
NFKB1	0.234374129	0.879862179	1
PIK3IP1	1.567589812	0.334291835	1
GPN3	-0.309648944	0.743415091	1

PODN	0.210568229	0.861005606	1
ENSMMSG00000010991	1.417713574	0.355929403	1
SORBS3	0.030193667	0.985038341	1
SLC9A6	-0.015769897	0.988350192	1
CD59	-1.026046231	0.474079026	1
PRUNE2	0.750625907	0.681606594	1
RND3	0.344485685	0.775028458	1
LOC107131749	-0.684530975	0.632483314	1
ENSMMSG00000009860	-0.402149401	0.780255042	1
USB1	0.524355141	0.694928271	1
NFIB	0.510677754	0.751824372	1
FAM76A	-0.622591023	0.686339887	1
AKTIP	0.336310602	0.740000679	1
ATG5	0.205359018	0.833116057	1
CNPY4	-0.172400901	0.825384472	1
MED6	0.766010279	0.51320688	1
CA14	-1.223578138	0.395467887	1
LBX1	-0.130110407	0.916862816	1
PLCB4	-0.885216514	0.540480424	1
ALG1	0.561122341	0.606733585	1
UBLCP1	-0.405093808	0.706620716	1
RPP38	1.006293581	0.426040818	1
APTX	2.806010626	0.125203708	1
TMTC1	0.117361277	0.936991297	1
STS	-1.880523218	0.410313962	1
SOGA1	0.346550471	0.796224318	1
METTL2A	0.379101794	0.805698969	1
PLPBP	0.124012568	0.900130981	1
LOC107132045	-0.64939524	0.422265493	1
AZIN2	-2.849039103	0.142820438	1
LRRC42	0.338124129	0.777507006	1
RFC2	0.058441537	0.941906654	1
TTC7A	1.475290146	0.19400249	1
PRDM4	-0.594461116	0.680079355	1
PTPRT	1.413434326	0.589606717	1
PLAGL1	0.876812077	0.616900173	1
GMDS	1.444851572	0.205284215	1
AVPI1	0.347152532	0.718700009	1
PRPF38B	0.236713146	0.798081782	1
RGMA	-0.604443326	0.588611549	1
FBXL17	-0.257021158	0.876953761	1
KDM6B	0.233131167	0.830841154	1
SLC29A4	0	1	1
EOGT	0.844940663	0.610900017	1
PI4K2B	0.884288844	0.354672458	1
PDCD5	-0.401763106	0.719766648	1
ABCB9	0.641693519	0.679673653	1
PHLDB2	-1.131949898	0.256692242	1
USE1	0.933235832	0.524238894	1
VKORC1	0.26964602	0.784770565	1
ACAP3	-0.023043265	0.98068459	1
AGBL1	-0.657821642	0.7193175	1
CCNT1	0.009017616	0.993224862	1
ZCCHC7	1.291319869	0.312475926	1
TAF3	-0.406521541	0.724576481	1
C3/PCDHGA8/PCDHGB4/PC	1.46621629	0.409911732	1
ENSMMSG00000020366	0.659632802	0.476399503	1
LOC784417	-1.829822693	0.402743611	1

NSMCE4A	0.726963833	0.493185144	1
NUDT12	0.182293029	0.898968473	1
RFFL	0.497086898	0.764569593	1
RASD1	-1.112323442	0.470473428	1
MAP1B	0.328615063	0.752899216	1
EPN2	-0.407420917	0.767800436	1
MPDZ	-0.790838938	0.431001217	1
ZNF653	0.501811434	0.636778955	1
TAF7	-0.185006894	0.846768601	1
GTF3A	0.279291068	0.765544006	1
FZD4	-0.641549189	0.691786101	1
MORC4	2.546231034	0.141168946	1
PASK	1.019106467	0.287948906	1
RBFA	-0.365119362	0.749198865	1
SOS2	-0.490415651	0.641228994	1
CRTC1	-0.088390177	0.95450577	1
ADAL	0.525709155	0.747982553	1
ALOX12B	0	1	1
ARHGAP17	0.598162454	0.663863409	1
TJP2	1.882780685	0.368382889	1
LOXL1	-0.627696755	0.532142054	1
MAP1S	-0.067598953	0.950713643	1
FASTKD1	0.062551786	0.95543666	1
FARS2	-0.562135511	0.533026044	1
TTC3	0.365374374	0.811851774	1
SLC35A3	2.970707751	0.087519654	1
TCF4	-0.404596814	0.675128389	1
ING5	1.120989635	0.519763587	1
WWC2	-0.053841734	0.949520257	1
RDH10	0.731927579	0.498085723	1
CLMN	1.311022783	0.257890404	1
HAUS4	0.890180049	0.507036416	1
CDT1	1.579656545	0.135896757	1
TIGAR	-0.252887672	0.792459578	1
ESCO1	-0.262453141	0.807819728	1
ZNF75D	0.140151577	0.925712364	1
UHRF1	1.627610557	0.395629358	1
ATAD2	1.134944966	0.269396562	1
ACBD6	-0.037293147	0.973907643	1
SYNGR4	-1.873305605	0.389907368	1
POLH	-0.119679586	0.904145592	1
ITPKC	-0.214264236	0.840160788	1
USP28	-0.155513359	0.942339581	1
SPIDR	0.449242367	0.629690804	1
RNF145	0.323792109	0.845569006	1
CYRIB	-0.572487723	0.71849642	1
AGO2	-0.644803782	0.357075382	1
HIRIP3	0.771439418	0.495538947	1
ERC1	3.606805472	0.153888752	1
UGGT1	1.415432088	0.565578831	1
RRP7	-0.167631045	0.862922971	1
TAF8	0.089758096	0.958653151	1
ZNF316	0.201777869	0.835332565	1
SPOCK1	1.772105856	0.236502817	1
WDR12	0.084812145	0.931509362	1
HUS1	1.580606911	0.2222611	1
GRPEL2	-0.580236276	0.555737737	1
ZFAND3	-0.783842413	0.454053326	1

DMAC1	0.041224533	0.972639563	1
KIAA0930	-0.514406872	0.612172513	1
MET	1.096264469	0.42370066	1
ATP9B	2.221893417	0.200433044	1
RBBP8	0.657813567	0.541450009	1
AGFG1	-0.382293431	0.799917664	1
PREX2	-0.735861364	0.341434545	1
ATP2B2	-1.056550955	0.561892394	1
TCTN1	-0.051116295	0.958259031	1
GABPB1	0.119343353	0.936684038	1
G26537	-0.146656234	0.917091991	1
SCRN3	-0.147348519	0.927798386	1
ACAP1	0.87014508	0.403088383	1
ENSMMSG00000023320	0	1	1
TADA2A	-0.21431325	0.829498016	1
TSTD1	2.188120273	0.113107998	1
CDK5RAP2	0.134952109	0.916448951	1
KIFC3	1.260045959	0.53541982	1
KIF2A	0.238086647	0.832319863	1
FEZ2	1.025992916	0.521382027	1
EPN2	-0.543413916	0.708964236	1
TTC13	-0.214280006	0.792430181	1
CYB5A	0.30862435	0.797257333	1
ZDHHC17	0.602960392	0.724712585	1
SMNDC1	-0.218408398	0.827027337	1
MED14	-0.394579473	0.79960748	1
MARCHF5	-0.342003582	0.705916228	1
LTA4H	0.458289767	0.666751756	1
BCAS3	-0.726150246	0.425612995	1
AAGAB	0.075402386	0.930366562	1
RNF220	-0.607986278	0.573549122	1
HNF1A	0	1	1
TMEM221	0	1	1
HNRNPM	-0.143982243	0.881050604	1
PRPF38A	-0.041169885	0.9596012	1
FNBP1L	-0.432947787	0.776792196	1
FAM133B	0.932935445	0.412117145	1
NTRK3	1.693103518	0.093196327	1
TRMT12	-0.174486913	0.877455208	1
RPL9P8/RPL9P7/RPL9/RPL9P	-0.476967477	0.669543489	1
ENSBTAG00000050725	0	1	1
EMP3	1.229259691	0.314321088	1
MPZL1	1.38974211	0.419206402	1
RIC8B	-0.486761596	0.638622413	1
C28H1ORF198	0.909138431	0.518263084	1
ARRDC4	2.014749667	0.100490925	1
ZNF280D	-0.101809725	0.949356452	1
TADA2B	0.04790802	0.967228125	1
POMK	-0.461355134	0.700811587	1
WDR73	-0.42952172	0.637958355	1
HPGD	1.762892087	0.285628723	1
TBCK	0.303992008	0.851299337	1
FAM178B	-2.850450723	0.173249958	1
LRCH1	-0.065122022	0.946212419	1
VPS13C	0.148893661	0.94106792	1
MPZL2	2.450306306	0.186776106	1
CCDC149	-0.555381625	0.73281746	1
GOLGA7	-0.006050737	0.996940017	1

RPP21	0.195399385	0.865269871	1
TIE1	-0.597473535	0.499680679	1
ZBTB8OS	0.326951256	0.716877311	1
C5H12ORF29	-0.343620746	0.716676664	1
DMTF1	-0.23875512	0.810689752	1
EXOC8	-0.198823073	0.851382018	1
PPP3CC	0.366046099	0.81238673	1
PAK2	0.786473838	0.53735042	1
ARHGAP12	0.695783712	0.571497585	1
IDI1	0.117171243	0.90235853	1
MRM3	-0.428754445	0.679337904	1
PALLD	-0.855520362	0.545736506	1
CRY1	-0.107042971	0.944114774	1
LRATD2	2.345089425	0.165315067	1
CCDC13	-0.504614378	0.733464913	1
MUS81	-0.311837	0.719800935	1
CEPT1	0.546072728	0.720091383	1
EML2	1.43397206	0.394343389	1
ATXN1	-0.843880384	0.3995519	1
FAM102A	1.204262139	0.261518932	1
BOLA1	0.145041226	0.884914168	1
PRRG2	1.64592216	0.187733676	1
PAPLN	2.263029758	0.099315709	1
GNPDA1	0.394274099	0.784536836	1
FRZB	-0.120061401	0.94274582	1
ADAMTS9	-0.714987323	0.46612794	1
CYB561A3	0.044675507	0.967697947	1
TENM3	-0.302164408	0.783064733	1
TSPAN6	-0.078381925	0.916756418	1
PPP1R14A	1.033495681	0.369073848	1
ZNF385B	2.586930845	0.152146332	1
TEK	-0.566277343	0.587888251	1
STARD4	0.300296673	0.798807482	1
TMCC3	1.82149676	0.177468377	1
CDS2	1.232193601	0.463938377	1
COMMD4	0.689663119	0.491381295	1
SORD	0.858521356	0.401913264	1
CDK12	1.118870756	0.48413323	1
ENSMMSG00000021079	-0.085078433	0.939050219	1
TRMO	0.656595617	0.647263822	1
ENSMMSG00000008854	0	1	1
ACP5	1.726691382	0.146023516	1
REEP1	-2.194825373	0.240643789	1
MYLIP	-0.248463775	0.869945301	1
MSX2	-1.82860309	0.411560387	1
SLC25A13	1.596348613	0.334251523	1
SLC66A1	0.200125416	0.838112999	1
GTF2A2	0.334996985	0.7635713	1
ADAMTS2	-0.108095449	0.919871287	1
NFYB	-0.695232717	0.458269733	1
CYP27C1	1.740265214	0.269766364	1
ADGRA2	-0.132350226	0.882496499	1
SPATA5	0.326221919	0.827640752	1
ZDHHC13	0.777134505	0.605268117	1
SPECC1	0.388078977	0.765266753	1
HMCN1	-2.685025398	0.519130309	1
CPNE2	0.118759827	0.894100804	1
CLCA1	0	1	1

HPF1	0.297637784	0.776053578	1
THRB	-0.80938653	0.480643477	1
PROS1	-0.109383243	0.915511333	1
GSTM3	0.490037661	0.650849718	1
LZIC	0.030942097	0.975022058	1
CNOT6	0.245723897	0.807245287	1
RTN4R	0	1	1
LRRC28	-0.904337215	0.364313127	1
PARP11	0.933286607	0.309190025	1
EZH2	0.511089869	0.627752384	1
DDX59	-0.462194618	0.636968103	1
ASB3	-0.490183139	0.736904823	1
GPATCH4	0.130816201	0.875904558	1
CDK17	-0.231301859	0.875749996	1
ORMDL2	1.1444168	0.242382816	1
SLC2A4RG	1.557674912	0.1534754	1
UPF2	-0.579592535	0.554275835	1
RYR3	2.515759718	0.284493663	1
SEC31B	0.571721608	0.723263023	1
BRSK1	3.750367353	0.118391733	1
HSPA2	0.931009135	0.506990517	1
NCOA3	-0.150126317	0.941430795	1
COLEC12	-0.616766764	0.529893404	1
PEX13	0.271456016	0.816986397	1
LMNB2	0.060255065	0.944235825	1
ZMPSTE24	1.354791753	0.316769804	1
STARD5	1.161694391	0.186764572	1
CEBPB	-0.802604003	0.429699391	1
BNIP2	-0.736769434	0.621415703	1
RPS6KC1	-0.337309473	0.824257063	1
MMD	0.887817371	0.58986867	1
ELL2	1.50852973	0.148184251	1
CBX3	1.288072547	0.293961449	1
SRR	0.079869629	0.937661219	1
MOCS3	0.236598435	0.845208155	1
ABLIM3	-0.387753443	0.74800009	1
EMILIN2	-0.39448326	0.68664248	1
ANGEL1	-0.258484232	0.833830025	1
UROS	-0.519752656	0.575283316	1
AKR7A2	0.488149241	0.621585526	1
TRPT1	-0.222032516	0.824436567	1
WDR20	-0.169563854	0.912852072	1
ASMTL	0.034593371	0.970123784	1
RAB11FIP5	0.791762257	0.655260655	1
SLC16A9	1.914975671	0.216481556	1
ST3GAL2	0.171927993	0.917144705	1
CHRNA9	0	1	1
DARS2	0.788262778	0.6182804	1
UCP2	0.567834617	0.594078694	1
LARP6	-0.852881086	0.343487055	1
ZNF383	-1.880436066	0.410057664	1
ATXN1L	0.590723811	0.661311303	1
MYO9B	2.487635267	0.31875456	1
C18H19ORF47	0.434604422	0.755417139	1
BIRC2	-0.560613468	0.560095691	1
PTPN13	1.260895884	0.208212627	1
AOC2	1.213078649	0.204052916	1
TENT4B	-0.657339967	0.672034235	1

EAF1	0.13394562	0.910186184	1
POLR3C	1.068146526	0.394881135	1
PLEKHJ1	0.114407893	0.90501461	1
SLIT3	0.605015169	0.68570124	1
SDC2	-0.306550562	0.775982074	1
LRWD1	-0.339205395	0.768994914	1
ALKBH7	0.842326616	0.506296255	1
ENSMMSG00000002048	-0.04028623	0.967053523	1
ARID4A	0.283066026	0.775720964	1
USP11	0.577904583	0.569958745	1
SLC39A5	1.566527361	0.17524232	1
ZNF581	1.374315536	0.189587706	1
PARD3	0.531891327	0.636478102	1
DHDDS	0.705299988	0.517052743	1
DHRS7	0.629930706	0.631602652	1
GABBR1	-0.058640009	0.956384972	1
TTL	-0.810003729	0.391038344	1
WDR35	0.666654772	0.571508893	1
CHCHD5	-0.218165185	0.831116373	1
RNGTT	0.316838219	0.844060919	1
TOE1	0.015506689	0.988464961	1
VPS9D1	0.545548447	0.599396544	1
EYA3	0.442917205	0.794209453	1
MLH1	0.701390911	0.679271295	1
CLIP4	0.056925749	0.967040739	1
POLE4	0.821474559	0.461454498	1
TMEM185A	0.134634293	0.91063817	1
LDB1	0.689359723	0.549400615	1
SCAMP1	-0.650693682	0.505361212	1
RSRP1	0.450223949	0.605320457	1
DBR1	0.27159076	0.780085952	1
CHCHD7	1.153473064	0.343216926	1
IFT52	0.076205131	0.921611802	1
CPXM1	0.817490004	0.590407067	1
RHPN1	0.064502836	0.952927669	1
AKIRIN2	-0.366957505	0.759838157	1
TBC1D22B	0.290755478	0.742883231	1
INO80E	-0.150452487	0.863030287	1
PRADC1	-0.593422531	0.55761323	1
TMEM164	0.766851623	0.427021359	1
ELAC1	-0.521942823	0.555816434	1
THNSL2	-0.59304847	0.699233665	1
SLC35C1	1.014739476	0.513854104	1
GEMIN4	0.099944529	0.911526406	1
PIK3C2B	-0.358587158	0.780920728	1
CCDC18	1.560297227	0.375327315	1
AIDA	-0.005234578	0.995792282	1
IFTAP	0.417963022	0.704703914	1
RWDD3	-0.764509077	0.4240111	1
ATXN2	-0.648250719	0.544850632	1
ACOT2	1.446759932	0.26450196	1
FBXL19	0.793937399	0.488248945	1
RNF4	-0.182470006	0.904891699	1
SSH1	-0.036843922	0.973824464	1
G20800	0.58695526	0.781991491	1
CCNC	1.987749306	0.257197092	1
RAD51C	-0.012381919	0.993781545	1
UBE2E1	-0.38182467	0.809189416	1

SGSH	0.409691634	0.701387684	1
TLR4	-0.700880004	0.5194311	1
LNX2	1.30981222	0.349515965	1
ZNF318	0.402926546	0.749968112	1
POLE	0.893457937	0.446861868	1
NR4A2	-1.009207319	0.47511894	1
RFTN1	-1.09573564	0.419163361	1
CDIPT	0.482394511	0.557772915	1
ATE1	-0.343982167	0.824976092	1
FRMD4B	1.337451268	0.329856434	1
NCOR2	0.579740624	0.731293915	1
GANC	-0.483227873	0.75620767	1
PALM	-0.772496762	0.401234546	1
SLC29A1	0.902953008	0.506345378	1
RAB4B	-0.00854952	0.993348872	1
TPRKB	-0.018029409	0.985867229	1
GNRHR2	0.904564101	0.52860648	1
TEF	0.138696203	0.937514882	1
EYA4	-0.967787417	0.497284546	1
TANK	0.701629365	0.548019167	1
PPARGC1B	-0.860358785	0.48618913	1
PAXIP1	-1.135769503	0.785139561	1
LOC616574/PCTP	1.241027029	0.363984772	1
C24H18ORF25	-0.57662623	0.482496785	1
TSNARE1	1.199815234	0.371778467	1
SCARB1	-0.118563848	0.884626393	1
PTTG1IP	-0.73366204	0.377318909	1
VWA1	0.713061717	0.536428973	1
RNF2	0.252531733	0.826359574	1
ZUP1	0.808560208	0.513883127	1
MED19	0.64172141	0.512146197	1
CRLS1	-0.214348463	0.846063781	1
EIF2AK2	0.780776433	0.480774646	1
MID2	0.246225603	0.846056857	1
C1QA	-0.449064916	0.650790054	1
ENSMMSG00000019092	-0.423142707	0.613345262	1
ITGBL1	1.455174276	0.227255317	1
CENPN	-0.535990249	0.719190101	1
TMEM179B	1.416802634	0.256213978	1
RBM26	0.009751151	0.992875434	1
XRCC1	1.391862981	0.247604772	1
IFT122	0.683168701	0.472687859	1
WASL	0.337985924	0.772298118	1
ATF4	-0.721867012	0.381756886	1
HEATR5A	-0.69077681	0.712202739	1
RAMAC	0.633925735	0.61326118	1
FAIM	0.828328917	0.443872328	1
MADD	-0.750625297	0.484564537	1
AJUBA	-0.288623521	0.764301096	1
MRPS12	-0.945217736	0.36239989	1
ETNK1	0.757788271	0.384273308	1
ARHGAP1	-0.075145844	0.953131269	1
PCYT1A	-0.277871042	0.770392145	1
ACVR2A	0.056150319	0.960438835	1
MLIP	-1.822827747	0.48715486	1
CPEB4	0.049752097	0.974584488	1
TCHP	0.371464635	0.691982876	1
MARK1	-0.053328441	0.973154119	1

ATPAF2	-0.263013577	0.746223472	1
RBM28	0.218048545	0.826653601	1
LOC615278	-0.192378594	0.83007621	1
NUDT18	1.126852638	0.330713729	1
CTPS2	0.738398538	0.566593528	1
PITPNM2	1.675322288	0.687591508	1
SIGMAR1	0.887351942	0.423029428	1
CRLF3	0.2111268	0.850973239	1
LATS1	-0.141226223	0.897601444	1
TRIM68	-0.590892603	0.710535328	1
TMEM266	-0.467680218	0.721049956	1
INSIG1	1.999266923	0.116074364	1
LGR4	0.452335919	0.70736985	1
PARG	-0.339902163	0.814431933	1
KLHL2	0.052610339	0.962787625	1
TMIGD2	0	1	1
SLC5A6	-0.360215214	0.6774604	1
WBP1L	0.063384942	0.968294976	1
MAP3K1	0.545909693	0.606110855	1
C29H11ORF54	-0.031879477	0.976136245	1
BLVRA	1.317928609	0.404499998	1
ZDHHC8	-0.036228046	0.972821541	1
TMEM63A	-0.434555308	0.771706938	1
SPICE1	1.306353223	0.261729121	1
CDC42SE1	0.833783441	0.582221993	1
ERF	-0.089012385	0.926341408	1
CCNDBP1	-0.446264415	0.766068724	1
RBM26	2.783231958	0.102733813	1
SDHAF2	-0.423881614	0.68605395	1
LIMK1	-0.945706738	0.516706823	1
JMY	0.080902986	0.95391689	1
SLC20A1	2.676231627	0.097494798	1
ENSBTAG00000011470	-1.829645818	0.403995527	1
PHTF1	-0.66401127	0.361191691	1
FRG1	0.463237201	0.654620785	1
FAM13A	-0.171653255	0.913688297	1
ITFG2	1.122823907	0.369772907	1
LOC100848100	0	1	1
ZXDC	-0.039004097	0.965855396	1
SLC16A6	-0.951689965	0.425034701	1
TFDP2	0.002691777	0.998073469	1
TRAF3IP3	-1.042987978	0.292545829	1
MTA3	0.309409049	0.841527881	1
COX16	-0.689548968	0.513978638	1
ABTB2	-0.865274079	0.568090229	1
IL10RB	-0.309659745	0.734287521	1
TIPIN	1.270163775	0.341678422	1
PPIL4	0.452730173	0.686671571	1
TNS3	-0.055532089	0.971740559	1
RHOG	-0.0357554	0.969309055	1
COMMD5	1.330848835	0.257505177	1
G20171	0	1	1
CDK5	-0.151616213	0.860630761	1
FHIP2A	-0.544235884	0.636968973	1
FBXO46	1.585486845	0.224733019	1
CDK8	0.64387091	0.694825285	1
NSMCE3	-0.092204299	0.93282538	1
MRPL30	0.705961264	0.558373582	1

TOR1AIP2	0.433958934	0.689735818	1
CDKN1A	-0.83430297	0.474556866	1
SFRP2	1.938621384	0.241373393	1
CHKA	1.137587913	0.630582563	1
CCNDBP1	-0.401784061	0.792518703	1
PSPC1	0.175462309	0.871144146	1
LARP4	-0.365643158	0.811272185	1
RSAD1	-0.071908284	0.954103729	1
MGME1	0.504687787	0.641569675	1
PCOLCE2	-0.984221065	0.532600461	1
MTPN	0.409082956	0.707938177	1
NHLRC2	-0.160936057	0.887352761	1
GABPB1	0.420574303	0.623598056	1
NUBP2	0.271412031	0.83030014	1
LRCH3	0.432623245	0.799306002	1
ISLR	-0.448736675	0.645616175	1
SLC44A2	-0.559556929	0.613667723	1
POPDC2	-1.208372769	0.328061024	1
MRPL35	-0.627414885	0.520460941	1
BYSL	-0.083462767	0.931983847	1
PTPRG	-0.451759233	0.686188404	1
LDAF1	0.670549757	0.634190144	1
TMEM9B	-0.423897877	0.674056836	1
ZNHIT2	1.481325931	0.22679029	1
RGS2	1.363721295	0.319740155	1
GPATCH2L	0.547220834	0.688906341	1
MAGEF1	0.024331285	0.980049148	1
RNF144B	-0.397611395	0.801818254	1
KIF3A	0.515125893	0.574938891	1
KATNB1	0.823734076	0.487838377	1
NOL9	0.004550619	0.996497983	1
NFATC2IP	-0.196824308	0.872610754	1
KLHL20	-0.069724553	0.945027833	1
ATF6	0.636273846	0.505467952	1
MECR	0.456758991	0.648269696	1
DPH1	-0.195393741	0.896577634	1
GOLGB1	0.642395468	0.501007673	1
CAMTA1	0.013319103	0.989862578	1
CSPP1	0.370310661	0.741638407	1
RNFT1	-0.121643502	0.941467577	1
ENSMMSG00000005531	-0.148265235	0.932036138	1
TMEM131L	2.532620894	0.328984129	1
ARID5A	-0.202376869	0.879720202	1
MRRF	-0.371847104	0.702932249	1
EPM2A	-1.180121554	0.404212575	1
CDK13	0.590351926	0.719497219	1
BOLA-DYA	0	1	1
NCOA5	0.111020522	0.939274312	1
GLA	0.931510372	0.351100372	1
DGAT2/UVRAG	1.149781192	0.426180557	1
IRF2	0.254367987	0.812010294	1
DYM	-0.299841003	0.767336587	1
C1D	1.17431401	0.319125931	1
ENSBTAG00000052790	1.598796257	0.236481008	1
PI4K2A	0.312332922	0.792406531	1
CDC6	0.159555175	0.873918579	1
TMEM126B	-0.274947192	0.736033025	1
ZNF512	0.755727287	0.659637079	1

DDO	-0.345511347	0.803253909	1
EEF1AKMT2	-0.48138451	0.635337329	1
SPOPL	-0.304246975	0.772996832	1
TERF2IP	0.00492794	0.99607992	1
ADAMTSL5	-0.149904235	0.885357572	1
HGS	-0.016435497	0.986411074	1
ARRB1	1.738345109	0.106859159	1
MFSD12	-0.018981434	0.982999502	1
ADGRE5	-0.47758669	0.760682128	1
MAP3K13	2.366390983	0.127798937	1
C2CD2L	0.021604195	0.981026496	1
FUBP1	0.490909047	0.715583132	1
MARK3	-1.001409209	0.283854601	1
GPRC5B	-0.560553514	0.521984998	1
USP43	-0.317414694	0.838892965	1
LRCH3	-1.222023671	0.769201387	1
SRM	-0.033006126	0.974205904	1
TGFB1I1	-0.393125221	0.629022457	1
SSPN	-0.943223665	0.531306749	1
MRI1	1.662769903	0.225802259	1
IFT20	0.70851047	0.4657463	1
CFAP20DC	1.840004575	0.084326854	1
DHRS7C	-0.477885184	0.787035469	1
TDG	0.469508799	0.766175878	1
FHIP1B	1.183482718	0.336039212	1
PPH22	0.815959671	0.558249401	1
ARID4B	0.546044957	0.658774974	1
MACROH2A1	-0.097817685	0.949366063	1
ITIH5	-0.506488865	0.539626502	1
RPS4Y1	-0.467001716	0.641034565	1
SETD1A	0.163243268	0.88663125	1
PPM1L	0.557215066	0.666220901	1
PFN2	0.718585402	0.684959633	1
RAB34	0.584259605	0.484852105	1
PHF23	-0.372063595	0.649456436	1
COL5A2	-0.091099272	0.931398438	1
ASB10	-0.544091171	0.641537453	1
NEMF	0.714104898	0.651125161	1
SLC35A1	-0.22784786	0.799943807	1
KLHL25	-0.304342021	0.793650594	1
PAAF1	0.511460762	0.729214035	1
CISH	0.630079584	0.645073002	1
AGPAT1	0.19481655	0.828922086	1
LRIG2	0.259157572	0.808636554	1
SURF2	-0.332768957	0.733183558	1
YJU2B	1.438796994	0.319628155	1
MTO1	0.280318732	0.81689207	1
SRD5A3	0.520489229	0.512508357	1
CASP14	0	1	1
AP1S1	0.481992092	0.611636633	1
BEX2	-0.08471115	0.93295942	1
TSPAN13	-0.176408807	0.906845795	1
NUP54	-0.413409905	0.674515585	1
TPCN1	0.296613815	0.789456656	1
RALGDS	-0.292054311	0.846021254	1
FASTKD2	-0.352457545	0.73211971	1
DCXR	-0.792370696	0.563753237	1
SSBP4	0.044080344	0.963247744	1

TMEM143	-0.579966288	0.664070387	1
MBIP	0.253772929	0.821461592	1
CXCL12	0.14381435	0.89480321	1
G38069	-0.127897138	0.931269467	1
TEKT4	-1.826194196	0.430256035	1
CCDC82	0.159762249	0.873710633	1
SRI	0.374050751	0.70969057	1
NOL12	0.238558966	0.792041872	1
HMGN5	2.644404177	0.161950857	1
ACBD4	1.655810375	0.221762352	1
KLHDC4	0.395583286	0.684962432	1
NAALADL2	1.849496492	0.35353986	1
XYLT2	1.025906327	0.38150753	1
ALYREF	0.105811874	0.925990979	1
ZNF821	1.402078955	0.319092599	1
LUZP1	0.538572165	0.57557923	1
NME3	0.137448577	0.885287868	1
PIR	-0.801950861	0.421924563	1
TXNDC9	0.745814274	0.516126186	1
SLC25A29	0.190124073	0.901476362	1
SSBP2	0.690617644	0.700905793	1
PHF20L1	0.821203067	0.493911145	1
ZNF593	0.32096391	0.785155857	1
LAPTM5	-0.117052902	0.908467984	1
BPNT2	-0.463873664	0.615606971	1
ASAP1	-0.780238612	0.336550541	1
PRKAB1	-0.457887676	0.754068358	1
EIF1B	-0.766065083	0.3734838	1
RAB22A	-0.444365682	0.640291446	1
PEBP4	-0.993704732	0.475756618	1
YPEL5	1.113085228	0.421885941	1
LLGL1	-0.355578788	0.740457983	1
V-MPL	0	1	1
FMOD	-0.880935394	0.592023084	1
QDPR	0	1	1
COMTD1	0.630578158	0.603417679	1
LOC505600/UTP14A	0.049067323	0.957872287	1
C19H17ORF75	-0.334853862	0.833636109	1
TOGARAM1	1.202749606	0.346039261	1
TSPAN9	0.107690459	0.948652471	1
EAPP	0.155876318	0.85920877	1
PFKP	0.74518146	0.510903605	1
TSPAN13	-0.166032485	0.91123898	1
DIS3	0.632487274	0.588472981	1
YIPF4	-0.12942751	0.907176732	1
CBX1	-0.32280159	0.840310666	1
DNAJC17	0.615846021	0.494023741	1
TFAM	-0.141045269	0.882281802	1
RAB28	-0.93470023	0.530303058	1
SLC39A13	-0.638827136	0.565336266	1
CCNG2	1.029893406	0.545431358	1
APMAP	2.640471816	0.135777589	1
AP4E1	-0.606517714	0.593737585	1
TET2	0.730611891	0.511652373	1
GEMIN2	0.207627826	0.87698069	1
TMEM258	0.337074934	0.764586805	1
MAFA	-1.146374566	0.247539375	1
GEMIN5	0.327334074	0.800652732	1

KIAA0753	-0.016657651	0.98340978	1
ACOXL	0	1	1
COL23A1	0.34392901	0.777416382	1
LRIG3	0.63266569	0.559370935	1
NQO2	0.683029189	0.569532343	1
KLF10	-0.418624786	0.686321007	1
POLL	-0.545567118	0.579246424	1
SPPL3	-0.030119578	0.97570148	1
BSDC1	-0.26612784	0.900213639	1
CDK7	1.074868144	0.397959932	1
CHMP6	-0.47898288	0.675275926	1
RBM24	-0.475182124	0.694988251	1
RNF185	-0.078928499	0.936731022	1
MRPL47	-0.323423742	0.750287495	1
RPIA	-0.086727675	0.957627701	1
TMEM106C	0.113212065	0.910859095	1
HNRNPA0	0.530886849	0.653285858	1
SH3KBP1	0.279948193	0.836065344	1
TMEM245	-0.146007815	0.934660875	1
HMG20A	0.37818019	0.764099086	1
POGK	0.93490148	0.425479361	1
VPS26C	0.638467903	0.573697115	1
INTS13	-0.173821688	0.863464988	1
STK36	0.110075591	0.914041688	1
ETS1	-0.688039082	0.452746518	1
STX7	0.075346288	0.960753492	1
U2AF1L4	-0.314902257	0.719488671	1
SPAG5	0.66511988	0.512624096	1
PPP2R5E	-0.315383852	0.755310032	1
ARIH1	-0.676240255	0.478494125	1
PORCN	0.764013979	0.582755389	1
IGSF8	0.854473993	0.419943095	1
MTERF4	-0.166474631	0.857621543	1
DENND2C	1.349088562	0.395940742	1
TMEM38B	-0.520030606	0.640940796	1
IL4R	-0.54344603	0.586460222	1
CDC42EP4	0.632634841	0.596089151	1
NCAPD3	0.966185855	0.567458255	1
SCAPER	0.350637561	0.769955787	1
SCCPDH	0.83619997	0.531141901	1
DUS2	0.8316266	0.547131557	1
POP4	-0.254694209	0.742403408	1
RBM27	-0.11142798	0.907839245	1
ELOC	0.059695116	0.95867234	1
NPAS2	1.044173335	0.338375923	1
SCNM1	1.054847412	0.443902023	1
LBR	0.40862795	0.660574342	1
ASB12	-1.107645196	0.262183573	1
RPL10A	0.654524714	0.56991007	1
ENSMMSG00000001912	1.937062075	0.093481283	1
CCDC9B	-0.038735929	0.978050343	1
APLF	-0.310771955	0.740552338	1
SELENOT	0.310560828	0.775681308	1
CRTC3	-0.193047922	0.82121617	1
ZNF705A	0.71519551	0.694003336	1
UPF3B	0.518929167	0.706791771	1
KCNB1	0.446658679	0.796238438	1
METTL25B	0.040923945	0.969967194	1

SNRPF	0.627416779	0.614819801	1
GTF2H4	1.046672973	0.393677261	1
SEMA4B	0.034929175	0.974707165	1
SETD4	0.226982727	0.803731842	1
KIF3B	0.498871252	0.545514531	1
SLC8A3	-0.982814985	0.486042444	1
TMEM233	-1.088630375	0.464306337	1
RIOX1	-0.194826534	0.821113916	1
TRIM13	-0.685223836	0.516547821	1
NFKB2	-0.065738038	0.954395681	1
PLCG1	0.052026876	0.951741052	1
RPP30	-0.350166254	0.684272607	1
TMEM127	1.817416924	0.174795745	1
BCORL1	-0.346389593	0.708770561	1
CIAPIN1	-0.659446619	0.492884286	1
HSPBP1	-0.233998304	0.815846754	1
EIF2AK1	0.007503495	0.993209855	1
GREB1	0.692035293	0.759068198	1
TRIM37	0.56212734	0.506294134	1
PCED1A	0.664341048	0.414670971	1
SORBS2	0.588970613	0.730132299	1
ARF2	0.063992048	0.952637034	1
ESAM	-0.960507842	0.236834279	1
MALT1	1.345336641	0.444737267	1
NR2F2	-0.203673734	0.896614848	1
ENSBTAG00000045547	0.07032354	0.924373383	1
RAB28	0.168022751	0.867201543	1
CARD10	-0.538658133	0.553312427	1
SCO2	0.748645247	0.413130978	1
PLCB4	0.168128108	0.946684961	1
MEAF6	1.132053309	0.403014764	1
TYW1	0.473105561	0.70393306	1
RPA3	-0.796154215	0.352203486	1
MSH3	0.097314268	0.934244451	1
ENPP2	0.059485821	0.94646247	1
ATP13A3	0.090877637	0.952938054	1
TSPOAP1	2.533654762	0.162788821	1
AP4M1	0.718379626	0.455659053	1
PGM5	-0.486120509	0.601299859	1
TNFRSF4	2.260551319	0.110595433	1
RIOK1	-0.16487347	0.859304304	1
CEP68	0.164021475	0.901549866	1
PITPNM2	1.102713463	0.788322035	1
WDR47	0.240827914	0.877160233	1
ZMYM6	0.36705556	0.669265722	1
PSME3IP1	-0.141329642	0.886480392	1
POLA2	0.840273275	0.488312921	1
FAM13B	0.906823858	0.475447385	1
FOXJ3	-0.365484854	0.810872693	1
TOM1L1	2.042266928	0.235560214	1
CDC40	0.379518411	0.750074067	1
KCTD5	1.268993021	0.132321563	1
FAM168B	-0.40302236	0.692444188	1
NR3C1	-0.65648903	0.675002022	1
ATP5F1B	-0.627281757	0.458634205	1
SLC25A23	1.821358212	0.241855675	1
MACF1	0.683802493	0.566406643	1
TRAPPC3	1.267086851	0.352557303	1

ANKIB1	0.426272382	0.728112709	1
MED17	1.548659025	0.238045457	1
MYADML2	-0.930326153	0.424251595	1
NID2	-0.782289003	0.611915463	1
THADA	0.305099129	0.741980248	1
LRRC36	1.102578664	0.419770711	1
ANXA8L1	1.151940124	0.308805019	1
NEURL2	0.25991894	0.862414708	1
KRT6A	0	1	1
SLC9A3R2	-0.836369543	0.56943527	1
ENSMMSG00000009323	1.128917273	0.424959114	1
ORAI1	-0.763814541	0.402861766	1
CDH15	0.848019932	0.618984207	1
THOC7	0.897206823	0.488860273	1
LEMD3	0.045205634	0.976522652	1
HPF1	0.303438123	0.79261339	1
NPR1	-1.001216642	0.305378028	1
SEMA4D	-0.878453961	0.394843195	1
AASDH	0.625519008	0.611584325	1
TUT4	0.543309103	0.76076475	1
RIPK2	0.47078263	0.651753269	1
TAB1	-0.085846763	0.941018452	1
ADAP2	-0.205403907	0.832870807	1
ITPRID1	3.869187509	0.107118074	1
GABBR2	1.754285411	0.246341256	1
NIPAL3	0.164202454	0.888199128	1
G14976	-0.057329646	0.944657168	1
CLDND1	-0.04989672	0.961718255	1
COMMD9	-0.008315172	0.993107508	1
ITM2C	-0.015628147	0.988384158	1
BRD1	0	1	1
PGRMC2	0.293216237	0.843040428	1
DSC1	0	1	1
LIPA	0.723909649	0.583419903	1
CCDC50	1.313273346	0.220598961	1
DTX2	-0.670811511	0.643878617	1
PRKAB2	-0.247421927	0.874025121	1
JAK2	0.827022559	0.516941369	1
GPNMB	-0.131185974	0.893982693	1
PLEKHF1	-0.098806956	0.949602462	1
SLC35E1	-0.223132224	0.887896413	1
ST3GAL3	-0.300153797	0.807337076	1
NAPEPLD	-0.692550504	0.573995177	1
LRRC28	-0.459901361	0.608841257	1
EXOSC5	0.559060328	0.510459045	1
ENSMMSG00000013516	-0.197139044	0.894671498	1
GMEB1	0.643722438	0.47771752	1
ABLIM2	-0.264161876	0.876590571	1
SHPRH	-0.10390751	0.911086287	1
TPD52	0.434370958	0.78314346	1
ASRGL1	-1.02675658	0.204730161	1
GJA1	0.299324137	0.721741205	1
MKS1	1.699871968	0.220069735	1
ZNF395	-0.189778043	0.857242502	1
PUS1	0.639804151	0.605586031	1
PPP3CC	0.029102843	0.987594376	1
ADAMTS10	0.180058439	0.843026618	1
ARRDC3	0.245947057	0.871019289	1

RARS2	0.578890644	0.647434375	1
PDZRN3	-0.951367077	0.531172973	1
CDKN2AIPNL	-0.472080408	0.628589799	1
G36602	0.098728908	0.952372498	1
ABHD18	0.199587105	0.9058245	1
HGFAC	1.202820695	0.325016497	1
MIEF1	-0.339563706	0.732789875	1
CLCN3	0.726120714	0.709046689	1
LRRC27	1.079040444	0.343276955	1
POT1	0.721397018	0.66506178	1
CNTROB	1.073353435	0.406051212	1
SNRPA1	0.70042488	0.507674484	1
SLC22A23	-0.013237103	0.991204781	1
GPANK1	0.114398962	0.897827621	1
RAB43	1.389670667	0.370851257	1
CYP2B6	0.609872761	0.641443043	1
CDH13	0.202535761	0.880868269	1
TMEM51	1.392480174	0.273548175	1
SMARCAD1	1.384741586	0.267462552	1
ICMT	1.296544659	0.415195597	1
EXOC4	-0.530756446	0.718973946	1
EPC1	-0.235486341	0.83307348	1
POP7	-0.287385062	0.740264439	1
TLE3	3.021805942	0.179508548	1
NEU3	0.154719048	0.928710028	1
SSBP1	-0.252394358	0.819622408	1
WWTR1	0.110524913	0.942507333	1
CERS1	0.092214438	0.933879073	1
EXOC4	-0.427697607	0.781488317	1
PEX11B	0.774073717	0.654995379	1
TTI2	-0.525230278	0.5158206	1
PCID2	1.096063551	0.396973733	1
HDDC3	0.859148168	0.522220841	1
ALG2	0.424367824	0.780304394	1
CWC22	-0.183784704	0.849547563	1
TJAP1	0.705875379	0.594090377	1
C24H18ORF21	-0.184154622	0.846935166	1
AMIGO1	-0.385615839	0.763409727	1
DMD	-0.012204314	0.993577306	1
CPAMD8	0.745709989	0.453656831	1
NACC2	-0.103001299	0.911196108	1
KHNYN	-0.000986088	0.999317423	1
PPP4R3A	-0.265241559	0.791981443	1
CREBZF	0.014990274	0.985216487	1
LZTFL1	0.448864397	0.6709369	1
RABL2B	0.750946931	0.443553164	1
LSMEM1	-0.759596964	0.513697202	1
RNF217	-1.019109329	0.308615686	1
PIKFYVE	1.438480671	0.308297297	1
BAP18	1.203593062	0.2932286	1
HEPH	0.590111211	0.587560749	1
ENSMMSG00000001012	-0.892721535	0.37982924	1
OSGIN1/NECAB2	-0.77048462	0.458088978	1
RBFOX2	0.986316464	0.568027193	1
CALY	0	1	1
CCL25	0	1	1
ENSMMSG00000013514	0	1	1
PLCH2	3.470517928	0.098122167	1

FN3KRP	1.583544155	0.25334908	1
ROCK2	-0.143360662	0.911484503	1
NAB2	-0.51502335	0.53623783	1
ZZZ3	0.307710305	0.84298227	1
ARF6	0.138813393	0.869533799	1
LYAR	0.266107239	0.799444088	1
PACS1	0.226853088	0.85027537	1
THY1	-0.877678948	0.398000367	1
ERC1	0.075128099	0.947687282	1
P4HA2	-0.830818536	0.576321213	1
AKAP4	0.607299163	0.510526371	1
LSM6	-0.319728366	0.7461418	1
CGGBP1	-0.330050478	0.703697159	1
CAMK2D	0.077169276	0.95141242	1
AP4B1	0.740613867	0.425787245	1
APOO	-1.019021592	0.296391189	1
ZNF750	2.360929995	0.101344365	1
UNC50	1.186888058	0.354496108	1
TRAPPC14	0.278096112	0.76095593	1
DDIT4L	-0.917294578	0.512014041	1
CDIP1	-0.639908639	0.466182749	1
QRSL1	-0.370450903	0.733311507	1
ZMYM4	0.702858166	0.557190902	1
PRUNE1	-0.586185954	0.570830701	1
COX11	-0.370451272	0.710197096	1
PTPN9	0.060027481	0.949196128	1
CTTNBP2	0.816181872	0.589376837	1
MIGA1	0.131039654	0.935697798	1
MMRN2	-0.748305894	0.331405065	1
POLE3	-0.294755404	0.850499922	1
GCHFR	0.655204585	0.455205593	1
NT5DC2	0.497855332	0.584950477	1
WNK1	-0.869635725	0.584095224	1
OSBPL1A	0.164613935	0.918432582	1
CIAO2A	-0.306468912	0.838984154	1
GPRC5D	2.057542638	0.134863636	1
ZNF276	-0.072703328	0.940397198	1
UBE2A	-0.313191468	0.735350452	1
ZC3HAV1	0.335013729	0.831779554	1
AMOT	-0.079714452	0.962083875	1
MORC3	0.708820327	0.686021109	1
CLEC16A	-0.090944447	0.924422984	1
PPIF	-1.041655536	0.343012367	1
POLR1C	0.083635899	0.922801185	1
ARF6	0.173853038	0.838695209	1
ZDHHC6	0.15062561	0.854154872	1
SNRPE	-1.912117913	0.503024336	1
CBR4	0.178266638	0.910280639	1
P4HA1	1.348639764	0.468695657	1
RPP25L	-0.189407012	0.881443458	1
PIN4	-0.029521306	0.977463894	1
RHBDD2	0.560919925	0.579540692	1
ATF7	0.155249868	0.875536088	1
LIN37	0.15668701	0.898944145	1
PPFIA4	-0.054250744	0.957771489	1
ZFYVE9	-1.009828378	0.383322883	1
BBIP1	0.252609857	0.784577494	1
FAM3A	1.107451905	0.371024461	1

HCCS	-0.89153131	0.313464018	1
NFIA	-0.002731443	0.99766521	1
HINFP	-0.261758964	0.782455591	1
LTBP2	2.270193541	0.088102272	1
ZDHHC20	0.789954345	0.446257611	1
POC1A	1.899128844	0.086664565	1
FMR1	1.13478055	0.372830483	1
FRA10AC1	1.567927959	0.251683587	1
GALC	2.375373631	0.166979588	1
PLXNB1	0.256871856	0.870684216	1
PHF10	1.005168879	0.368624856	1
ENSMMSG00000019915	2.283599097	0.121051877	1
PAN3	0.515439077	0.655329896	1
ERCC2	0.024507289	0.978419688	1
TAP1	-0.463416519	0.688161704	1
PIK3C2A	0.617805323	0.538168281	1
PLA2G3	0	1	1
SRPK2	0.940901891	0.331520702	1
TCF3	0.326615653	0.78613276	1
IER3	1.533750018	0.296003513	1
CHD8	0.540026859	0.831914982	1
DUT	-0.184054374	0.829798529	1
FIP1L1	0.450899241	0.695968718	1
PRX	0.70433497	0.612827861	1
PHACTR4	0.434012092	0.702137104	1
WAC	-0.431512205	0.681550141	1
DSTYK	-0.384808237	0.69290881	1
ANO5	-0.895014706	0.47559774	1
TRIM33	0.29483601	0.740981118	1
THUMPD3	0.410933953	0.798338127	1
TIPARP	0.996042609	0.418273076	1
GGTA1	-0.557692586	0.575440773	1
EVL	1.047556791	0.364745149	1
FBXL17	-0.090013212	0.956677113	1
LENG1	0.932056466	0.399602648	1
PISD/PRR14L	0.149410773	0.918867261	1
WRAP53	0.796801184	0.437573858	1
FAM98A	-0.369402361	0.812193088	1
CBR1	-1.072968971	0.296394851	1
C23H6ORF136	-0.536319062	0.583960754	1
LTBP1	0.766378544	0.66021109	1
NT5DC3	0.189349588	0.904288213	1
RUNDC1	0.478736441	0.618224626	1
AAMDC	-0.984853952	0.368589949	1
SMG9	0.324141469	0.729923278	1
AMOTL2	-0.504595802	0.606248956	1
MRPL57	0.797125334	0.50760224	1
DCAF15	0.152725142	0.856943241	1
PUM1	-0.113149511	0.956538206	1
AGFG1	0.717630226	0.63575354	1
SMARCAL1	-0.148661909	0.8938534	1
DPCD	-0.397691156	0.698999527	1
ZCCHC14	-0.621383412	0.567624035	1
PRKAG3	-1.23000459	0.3265272	1
ATXN7	0.398348712	0.719817867	1
ANKRD46	0.353518228	0.843602461	1
YIF1B	0.192522637	0.873344273	1
SEC14L3	0	1	1

ANAPC1	2.106996992	0.611524024	1
NOS3	0.079261496	0.941464571	1
THBD	0.41471372	0.696248897	1
EYA4	-1.43745488	0.348061229	1
KBTBD4	-0.253122623	0.868987708	1
ADRB2	0.24705641	0.891419991	1
TFPI2	-0.856153639	0.572371931	1
TAMM41	-0.361138743	0.688321697	1
PUM2	0.045716011	0.977312453	1
AMBRA1	-0.155353366	0.928076013	1
PEX6	1.766904216	0.20176097	1
MYH13	-1.328761258	0.279900805	1
EXD3	1.397981732	0.275160423	1
FMNL3	-0.651510259	0.358255491	1
SEPTIN11	-0.300335686	0.755178181	1
GMNN	0.808382011	0.545462598	1
DNAJC5	0.250510972	0.818069129	1
DTD1	0.870867529	0.530789067	1
AXIN2	1.329972254	0.330682363	1
SLC26A6	-0.307790027	0.703711568	1
RBPJ	-0.212705945	0.837938879	1
MMAB	0.057543032	0.952407251	1
NUBP1	-0.031518897	0.967501116	1
C28H1ORF131	-0.488065099	0.600499937	1
HDAC7	-0.143393208	0.946957149	1
ZC3H18	-0.430587373	0.796312019	1
LOC100297097	1.049449411	0.418063277	1
USP31	-0.240590835	0.814435476	1
G4313	-0.555713078	0.692340849	1
TSPAN12	1.156534872	0.405395378	1
ECM2	-0.248585539	0.876796071	1
TVP23B	0.232896035	0.874657687	1
RBM25	0.969650132	0.457936224	1
CUX1	-0.199045895	0.883919552	1
TMEM201	-0.232206441	0.837471549	1
CD99L2	0.280541834	0.87389557	1
TMEM208	0.594488989	0.539507853	1
TBC1D23	0.155494158	0.901451359	1
CNNM4	0.697982412	0.643925207	1
TMEM141	1.452496237	0.223406086	1
RAB40C	0.078465941	0.94452071	1
RELCH	-0.012857763	0.99362757	1
NDEL1	0.220344769	0.902060516	1
TSR2	-0.695374485	0.440034359	1
AHI1	-0.103238594	0.892757642	1
DMAC2	-0.238932124	0.837763499	1
SLC12A6	0.744541614	0.582780705	1
IFT88	1.31541249	0.277546049	1
MTCL1	-0.265624354	0.860181262	1
TMEM223	-0.457601061	0.624997363	1
MRPL44	-0.56610109	0.543498597	1
EMILIN1	0.216138036	0.838257206	1
GM2A	0.497867999	0.606958918	1
GUCD1	0.204036961	0.876933498	1
IKBKG	0.421792132	0.667635312	1
JMJD4	1.056322751	0.331307721	1
HEMK1	0.596229205	0.576841865	1
SHOC2	-0.433178907	0.774811642	1

FAHD1	-0.80862194	0.372756578	1
EXOSC2	0.077100327	0.939187099	1
TRAPPC6B	0.187165584	0.879124338	1
GSTA2	1.520773315	0.112995858	1
MAP3K7	2.906929264	0.104629147	1
USP42	-0.201457119	0.896057038	1
ANGPTL2	-0.271423429	0.786659561	1
SCD	1.664581717	0.357173431	1
PNO1	0.358523119	0.828071753	1
G30765	0.589709322	0.665918449	1
OXR1	-0.280891459	0.856130483	1
TAB2	-0.745628577	0.619010604	1
PKNOX2	0.206190843	0.881318136	1
PIANP	-0.35292523	0.724880457	1
LOC100300759	0	1	1
CXXC5	-0.729828698	0.484263418	1
F3	1.254047448	0.252459534	1
TNIK	0.002706883	0.998774101	1
SLC52A2	0.675219795	0.598193572	1
LDLRAP1	0.041634652	0.966627349	1
G31995	0	1	1
PPIP5K2	1.700736403	0.201986952	1
PPFIBP2	-0.306398757	0.799022982	1
RALB	-0.113122697	0.912875985	1
SNRPB	-0.269004726	0.792547736	1
CPLANE1	0.269379775	0.782284813	1
CLK2	0.290166714	0.852459618	1
VPS33B	0.131326659	0.90038308	1
SMARCA4	1.036631594	0.470044701	1
PAFAH1B3	1.09118976	0.335453213	1
NDUFAF6	-0.832956278	0.572255051	1
FAM222B	-0.500863719	0.7487595	1
ENSMMSG00000002945	1.117425091	0.330202116	1
ENSMMSG00000016118	-0.184225368	0.850480386	1
TOMM40L	-0.621737168	0.504991579	1
KCNQ5	-4.46110509	0.284133625	1
ZMYND8	1.48493886	0.370456411	1
TFCP2	0.31481048	0.849976222	1
ACSBG1	3.313295395	0.088429173	1
SC5D	1.266911451	0.429969782	1
PANX2	-0.03015693	0.975213475	1
GSTT4	0	1	1
DAPK1	0.763178393	0.621553731	1
SPATS2L	0.376760103	0.764473401	1
MTMR14	-0.466656177	0.675803184	1
FCF1	0.277020012	0.800957757	1
KIFC2	0.675410016	0.466789537	1
ARV1	0.020220049	0.983669078	1
MCMBP	-0.11266998	0.918114937	1
SNAPIN	-0.444068831	0.675064583	1
AMPD2	2.008570365	0.121086717	1
NLE1	0.214232936	0.828572231	1
BID	0.499217615	0.640420258	1
FOXJ3	0.788446066	0.644482907	1
MARK4	0.914868252	0.483060055	1
EPM2AIP1	0.947504995	0.448684991	1
MTF1	-0.063502962	0.948417341	1
CHD1L	0.412580584	0.699153218	1

SLC9A2	-0.731076794	0.667241224	1
FAM204A	1.74151567	0.184353749	1
PDZD7	-1.88634039	0.426900922	1
MRE11	1.126741278	0.468004542	1
PITPNM2	0.243011923	0.828061293	1
TTLL7	-1.001011218	0.442373306	1
G8300	-0.019204656	0.984915798	1
PHAF1	0.645733502	0.70500277	1
CYB5D2	1.518571045	0.385418955	1
ZDHHC14	0.140747677	0.94719724	1
SS18	0.601173784	0.636567238	1
COPS7B	0.94113878	0.501754824	1
YLPM1	-0.15224972	0.87960945	1
HEXD	0.31631537	0.768020736	1
RXRB	-0.490739567	0.7481113	1
PPP3CB	0.587486512	0.788662929	1
RPRD1B	-0.146413309	0.878144972	1
HTATSF1	0.532718753	0.755653852	1
MTRF1L	0.170653565	0.842022183	1
DUSP12	-0.038855622	0.968950906	1
PAFAH1B2	0.076758593	0.962876971	1
CFB	-0.589608438	0.611377889	1
HDAC4	-0.765437211	0.527251973	1
PAN2	0.228528866	0.84308455	1
SEC14L5	0.906870018	0.429891516	1
SLC16A3	-1.225780817	0.387174953	1
SNAPC3	-0.355781634	0.711600601	1
REPIN1	-0.521074158	0.642599721	1
RRP9	-0.318713155	0.752980784	1
LOC511229	0.985672982	0.516424013	1
MRPL46	-0.388739744	0.68057158	1
SELENOF	0.511181623	0.626848479	1
NECAP2	0.179811917	0.84230452	1
EGFL7	-0.033123425	0.973059597	1
NARS2	-0.117507659	0.938847031	1
SAMHD1	-0.208947983	0.893073794	1
FAM120B	0.553206403	0.584251266	1
P3H1	0.367981148	0.672216899	1
MEX3D	-0.155073766	0.893080597	1
MAF	-1.123308494	0.479075532	1
S1PR1	-0.913782708	0.541530187	1
ABCB4	-1.337833456	0.249928276	1
NBEAL1	1.100672514	0.321834163	1
CDV3	-0.293238361	0.849446432	1
SIK2	-0.401647661	0.710715914	1
CYP27A1	-0.096265863	0.936592601	1
EXT1	0.098592933	0.920898678	1
HS3ST6	-0.217764779	0.827718637	1
ZBTB17	-0.24643595	0.803616776	1
NKTR	0.858314485	0.429100392	1
SLIRP	0.367284816	0.758114541	1
ENSBTAG00000026836	-0.603300272	0.610099964	1
TSPAN14	0.422349535	0.793397619	1
UFSP2	-0.286684285	0.718116282	1
REPS1	0.251459267	0.828511538	1
ATP6V1G1	0.121679715	0.902463688	1
EPB41L1	1.543913686	0.288376514	1
MAP3K10	-0.36396353	0.730676203	1

MTARC2	0.175810408	0.855925534	1
OCRL	-0.416467095	0.768453478	1
VPS54	0.049224581	0.963433879	1
CCSER2	-0.169764691	0.915736499	1
C1QC	0.061874809	0.948995073	1
P2RX6	-0.680735942	0.573450093	1
PTPRS	2.465231842	0.192116273	1
IFT46	0.151841322	0.880009607	1
OXR1	0.189906716	0.878163464	1
PMS1	-0.466757437	0.766719973	1
OXSR1	0.104337505	0.93630529	1
MED27	0.096533719	0.921560758	1
MRPS17	0.686138501	0.51488973	1
CEP295	1.278138415	0.365673141	1
ERLIN1	1.439348668	0.225631473	1
SLC43A2	-0.943189359	0.310578822	1
MRPS36	-1.068677469	0.204099164	1
CDYL	1.842300472	0.170886345	1
SASH1	-0.156200785	0.851539178	1
SCN4B	-0.611457014	0.689116226	1
PDE7A	0.046265187	0.979512842	1
TTC19	-0.229982771	0.859703388	1
ADRB1	0	1	1
TLE1	-0.990917019	0.343744773	1
CUEDC2	-1.060156485	0.567304675	1
GATD1	-0.496646289	0.647070179	1
EIF2B1	-0.119899155	0.882587515	1
TNS1	-0.754877573	0.562446918	1
FTSJ1	0.115330599	0.943737967	1
RARRES2	0.938471563	0.464358083	1
MCCC2	0.639905967	0.701872351	1
VRK2	0.448721652	0.768520235	1
SYNRG	-0.431149384	0.694679161	1
HS6ST1	-0.88916195	0.575171907	1
TNFAIP1	0.070022658	0.965442351	1
LRRC45	1.114470236	0.253793134	1
HEATR3	0.76938532	0.656600102	1
PLA2R1	1.410146763	0.232748568	1
ALG5	0.712563048	0.420382012	1
TIAM2	0.827267087	0.571116151	1
CHMP2B	0.472626696	0.611395397	1
ZYG11B	-0.763550609	0.385233952	1
FBXO38	0.603691658	0.726088937	1
MYO3B	0	1	1
ADD3	-0.731245052	0.630119718	1
TTYH3	0.928269712	0.310235825	1
TELO2	0.386847682	0.728199798	1
GJB4	0	1	1
STAB1	1.34089877	0.331554542	1
NFKB1	0.799241308	0.56794302	1
YAP1	0.211145414	0.891458941	1
ATRX	0.928745695	0.466220243	1
CYB5R4	0.427690689	0.773296281	1
ACSF3	1.633885091	0.202321467	1
ID3	-0.771775664	0.343333142	1
DOLK	0.613660528	0.537550057	1
HSF2	0.742010195	0.607074423	1
CAVIN3	-0.006002988	0.995837196	1

MBP	-0.481851631	0.685524912	1
DDX39A	-0.567002899	0.545024396	1
LOC788425	0.919299514	0.447531954	1
NUDT9	-0.348250029	0.823330453	1
CENPU	1.10676379	0.433037628	1
KLHL5	0.380678893	0.731453699	1
ATF2	-0.01530715	0.992338424	1
C25H7ORF26	-0.548542466	0.548725326	1
CARMIL1	0.640406648	0.547008127	1
JPT2	0.286996764	0.736890149	1
ABHD6	0.961316545	0.521509646	1
EMC8	-0.296753561	0.780803953	1
HACE1	0.495735565	0.754805026	1
THAP4	-0.545377199	0.643978767	1
PMVK	0.123491633	0.916125858	1
SHD	-1.830017301	0.401352985	1
PPM1L	0.926114301	0.484192542	1
ATRN	0.325842107	0.679545212	1
CCDC90B	-0.110196471	0.916665886	1
HRAS	0.103704792	0.930573376	1
MSMP	-0.413002847	0.658265657	1
KAZN	1.44983245	0.36933868	1
AVIL	-0.445580171	0.61045734	1
ATG9A	-0.89054415	0.558605109	1
ALKBH4	-0.091588919	0.937563775	1
GATAD2B	0.361491204	0.744348004	1
SLC25A46	-0.56027371	0.717748711	1
MAPK14	-0.507598792	0.546364557	1
LIPE	-0.488022828	0.618431472	1
DDX51	-0.527343842	0.573175414	1
ATP6V0B	0.184149234	0.871508677	1
COBL	1.746631984	0.130604235	1
GPN1	-0.186262592	0.823428636	1
HSCB	-0.098641536	0.920797523	1
STK38	-0.49086817	0.611262846	1
C1QB	-0.113343561	0.910663052	1
DET1	-0.771272081	0.423187157	1
EFEMP1	-0.428459876	0.671606138	1
MFSD3	0.948682036	0.471176517	1
PPIP5K1	0.135280069	0.907127988	1
MKNK1	-0.845833033	0.403980269	1
CCDC66	0.302047255	0.778056009	1
SLC1A5	0.277221072	0.764736267	1
MAB21L3	-1.891325403	0.44125225	1
EIF2AK3	0.655389283	0.479225713	1
SEC24A	0.447947513	0.780463595	1
ARPP21	-0.837600506	0.616694222	1
TWNK	-0.163477375	0.886148044	1
TSHZ1	0.182110319	0.895531302	1
C2CD3	0.123239217	0.935889495	1
ZMAT5	-0.403122696	0.708428791	1
BEST3	0.102135021	0.950472264	1
MPP7	0.610335768	0.694147435	1
STX18	-0.21130339	0.841562152	1
LOC614522	0.620804198	0.672113017	1
RNF38	-0.1536078	0.887289886	1
ENSMMSG00000006911	0	1	1
ZNF576	0.856254728	0.434058663	1

NMNAT1	-0.327929547	0.722751488	1
MRPS18C	-0.554680141	0.575410862	1
PPP1R35	1.154097882	0.241514284	1
SLC25A36	1.003878725	0.485179834	1
TAF4	0.323457364	0.754333595	1
RGMB	-0.542318276	0.614181627	1
VIPAS39	0.594433376	0.628724524	1
ANK3	0.900324341	0.613119602	1
KAT7	0.340203935	0.793526013	1
GPR137	0.117348101	0.905674507	1
ZNF423	-0.619071525	0.693986516	1
WRN	1.14003357	0.322331026	1
PIAS1	0.156792133	0.873392711	1
HTRA2	-0.089460439	0.935837278	1
TGS1	0.658326528	0.601498801	1
CACNG7	0.099040337	0.951702562	1
FRRS1	1.594640829	0.289931691	1
WDR83	0.357194744	0.733378204	1
WDFY2	0.460663369	0.66698985	1
UBE2E3	-0.700167797	0.470118159	1
NEO1	-0.777098157	0.595592845	1
MRPL42	-0.119138263	0.911924798	1
SUZ12	1.101112106	0.384749074	1
TNS2	0.806927207	0.358396256	1
SF3B5	1.95760317	0.122703529	1
ZCCHC8	-0.322932211	0.737498212	1
SDE2	-0.175033047	0.862705823	1
RDH11	0.718131566	0.417740535	1
ATXN7L2	0.542675234	0.65435538	1
DNAJC9	0.254225877	0.822067902	1
TP53	0.209391493	0.839559206	1
CCDC91	-0.283919754	0.778134323	1
DUSP13	-0.713192182	0.602018137	1
CSPG4	0.856624924	0.442311894	1
PLA1A/ADPRH	-0.60333426	0.611171089	1
C24H18ORF32	0.155208719	0.870564379	1
LOC101904614	-0.734104101	0.480128383	1
TMEM65	-0.384460825	0.702321524	1
SLC25A44	-0.189498759	0.857451317	1
FBXO21	-0.60461148	0.682094643	1
FAF2	0.433624406	0.663954994	1
METRNL	1.832625975	0.091544852	1
BRB	-0.02280091	0.982880683	1
LDHD	0.101561849	0.916418751	1
ARSB	0.485550788	0.712150131	1
AIFM2	-0.258154143	0.788174823	1
ANGEL2	0.158339029	0.873555013	1
LRP4	0.981328548	0.452908159	1
FOSB	-1.049104008	0.393792585	1
ATP13A2	0.643044345	0.57944393	1
PDPK1	-0.085708875	0.928794291	1
RALA	0.324587701	0.737734306	1
SEL1L3	0.119157438	0.900692194	1
LSM1	-0.589577619	0.524549636	1
PALS2	-0.989428315	0.622590985	1
SNURF	0.197321855	0.874902261	1
FOXP4	0.090908485	0.953815346	1
ANKMY2	-0.011382804	0.989677731	1

KANK2	-0.075657751	0.941883049	1
UHRF2	0.42463563	0.640523828	1
ODAD4	-0.122863164	0.893074588	1
GGH	1.258383531	0.17984964	1
KLHL17	0.805320647	0.372258496	1
ZMYND19	-0.894880481	0.406057449	1
LTN1	-0.341755064	0.826767172	1
HOXC10	-1.01472679	0.464829109	1
SSBP3	0.12462053	0.937092902	1
SLC35C2	0.914038994	0.418296248	1
KDM6A	-0.103002906	0.908470279	1
EIF4EBP2	-0.496576081	0.663197598	1
ZMYM3	0.861420211	0.497069599	1
ANAPC11	0.131499809	0.907329639	1
SLC2A8	0.120723658	0.898236267	1
EFNA2	1.27120146	0.293998855	1
DISP1	-0.860064916	0.331806311	1
EDRF1	-0.085964674	0.921234683	1
HR	0.134479566	0.935481111	1
G5312	-0.030557529	0.970234655	1
FLRT2	-0.569931458	0.576728178	1
HSPA1L	-0.345980449	0.69655019	1
DNAJC15	0.593958033	0.641112497	1
POLR2J	-0.192893026	0.824878958	1
SEC14L1	0.031653936	0.978522152	1
ACTL6A	0.087489815	0.931960619	1
LSP1	0.816493249	0.533997243	1
MAP4K4	-0.111646396	0.946319556	1
NAV2	1.429762013	0.320500176	1
CCDC43	-0.207713172	0.849641657	1
NUP35	0.475825272	0.751529486	1
KHDC4	-0.000843135	0.999559203	1
ATP8B2	-0.365267101	0.663430127	1
NXPH4	-1.914175844	0.509381924	1
LOC615697	1.478136269	0.239662762	1
ASB14	-1.136941111	0.325525472	1
ASB1	-0.206451177	0.881200949	1
SIRT3	0.556583745	0.66223934	1
CYP20A1	1.036570627	0.361261339	1
SELENON	0.033149655	0.975316024	1
ELL	0.05173536	0.956922251	1
EPHB4	-0.226941211	0.77510121	1
CSTB	1.660512481	0.157496051	1
DLX3	0	1	1
PPARA	0.481099879	0.730036153	1
TMA16	0.587855808	0.589193361	1
CARD19	1.565356601	0.183198603	1
TMEM134	0.492862681	0.643761087	1
DDAH2	0.665565538	0.558606632	1
PODXL	-0.689020458	0.41539853	1
RALGDS	0.018189472	0.991056777	1
BTBD9	-0.65318206	0.667897994	1
USP30	0.678915162	0.580359398	1
HNRNPLL	0.008318224	0.993052227	1
GLT8D1	0.031364764	0.977177965	1
DPYSL4	-2.849368248	0.149191815	1
MRPL4	-0.429995504	0.647626297	1
TANGO6	-0.385380487	0.681259148	1

FGF21	0	1	1
CACUL1	0.471756573	0.665414136	1
TMCO6	0.000825372	0.999146638	1
EEFSEC	0.490219363	0.694257738	1
AHCTF1	-0.213286375	0.839326324	1
MAP2K4	-0.336700918	0.838108228	1
YBEY	0.014201222	0.988206939	1
STAM	-0.190989508	0.850085743	1
ARHGEF25	-0.583081979	0.442544146	1
CHKA	0.337648172	0.766613951	1
CYB5B	0.697562039	0.559881488	1
RANGRF	-0.102740153	0.925846199	1
RAMP1	-1.100034896	0.383980832	1
EPC2	0.017830295	0.987367914	1
KCTD10	-0.157203759	0.872870834	1
ZBTB11	-0.066225565	0.966648883	1
ASXL2	0.363990994	0.820371411	1
COL27A1	0.838573995	0.400955753	1
CSTF2	0.823369087	0.461722648	1
RITA1	-0.525088758	0.514033655	1
MON2	1.403854832	0.42707335	1
TTLL1	0.989513497	0.320875915	1
ITGB1BP2	0.13821279	0.923031365	1
ITGA1	-0.474965568	0.617403931	1
MYO5A	0.016381109	0.988241485	1
DENND1B	0.070942204	0.944889181	1
POLD2	0.140649357	0.886163323	1
GSS	1.646362077	0.168071788	1
SZRD1	-0.411827055	0.720200002	1
LZTS2	0.056098913	0.960846645	1
ZDHHC3	2.183003479	0.261505341	1
RNF125	1.394089684	0.370499365	1
PITPNC1	0.882590832	0.350207632	1
SLBP	0.418540485	0.783759454	1
YDJC	1.630682753	0.165014782	1
STAMBP	-0.325325662	0.727709914	1
EGLN3	-0.179491634	0.888730981	1
FHDC1	2.384641385	0.094879722	1
TAF11	-0.407590059	0.649709461	1
TBC1D8	0.154285102	0.902794388	1
PDGFRA	-0.382319949	0.696820003	1
RRP15	-0.197031004	0.825636627	1
GLE1	0.131043463	0.891376779	1
WDR18	0.265815376	0.786479206	1
LOC615514/GSTM2	-0.829801148	0.384185399	1
INTS6	0.269299688	0.808233219	1
HMGB2	1.156812992	0.383375856	1
EPG5	0.600123181	0.663584293	1
DPY19L4	1.185607299	0.339018024	1
ELP3	0.50214285	0.675648257	1
MCAT	-0.726870659	0.469110406	1
BLCAP	-0.833074259	0.402363784	1
C29H11ORF24	0.270729227	0.773577457	1
GUSB	1.590336679	0.158636088	1
ADGRL2	-0.358735865	0.703028286	1
NUDCD2	-0.026509322	0.975524783	1
YWHAQ	1.164535893	0.466133204	1
MYLK4	-0.939609849	0.579527335	1

OSBPL9	0.177588784	0.916762244	1
CDC42BPA	-0.296967503	0.772086471	1
MPHOSPH10	0.449114633	0.712519698	1
BGN	0.100966884	0.948655457	1
TSC22D3	-0.778465174	0.61051737	1
ENOSF1	0.046121411	0.96464693	1
HMBS	-0.317966061	0.73286143	1
TCIM	-0.971600726	0.351262903	1
DGKZ	1.281893949	0.488572481	1
ENSMMSG00000002030	-1.829863042	0.40245108	1
COQ5	0.063577008	0.953157058	1
SPIRE2	0.3846658	0.709247641	1
ABCD1	-0.657871092	0.478577007	1
TMEM184C	0.046709147	0.975659878	1
CWF19L1	0.282271094	0.79703183	1
DENND1A	-0.470603052	0.704731742	1
OAT	-0.457184484	0.580465253	1
SGCG	-0.249593671	0.870632284	1
DHX57	-0.180536103	0.854870338	1
AKAP12	-0.412672572	0.679357132	1
HPS5	-0.14644911	0.875776859	1
LRSAM1	0.660329467	0.584643836	1
RBFOX2	0.283956897	0.808339854	1
KRR1	-0.062909162	0.953406491	1
PLXNA2	0.266813664	0.785177613	1
MSRA	1.649750161	0.268423343	1
UBR7	0.513497262	0.622198264	1
TIGD5	-0.612331429	0.629434472	1
NECAP1	1.244870869	0.487132478	1
SLC25A37	-0.380851131	0.696030453	1
SPHK1	1.952845852	0.118422096	1
COPZ1	-0.341741966	0.698545159	1
ZC3H4	-0.155575792	0.885654169	1
DDX58	0.292071791	0.866374476	1
SEC11A	0.866053563	0.60915259	1
NDUFAF5	-0.272973344	0.796817613	1
ZNF408	0.081979749	0.948459041	1
SMIM29	1.346189732	0.293755533	1
SERTAD1	0.392511805	0.724528455	1
MISP3	1.201500106	0.419035818	1
CRYBG3	-0.347698946	0.744343462	1
SEC31A	-0.912841773	0.53596004	1
FHL2	2.540321588	0.091791905	1
BDP1	0.359812115	0.769054676	1
TMC6	1.871131261	0.182306141	1
GPRC5C	-0.612663593	0.61452435	1
SLC48A1	-0.775066646	0.402752876	1
HNRNPH2	0.282437441	0.858136558	1
YIPF5	-0.089122443	0.936440726	1
GUCA1A	0	1	1
AVL9	1.327837633	0.29738	1
ACOT9	0.73092635	0.592023933	1
PRKAG2	0.41226743	0.74928142	1
EVI5L	-0.624004657	0.535766901	1
ENSMMSG00000006577	1.329869465	0.202916004	1
PCGF2	-0.340760957	0.661842381	1
ANAPC13	0.567698435	0.61648596	1
MRS2	-0.486589506	0.608564201	1

CITED2	1.184828633	0.335014224	1
KANSL3	-0.334311774	0.767081636	1
ATAD1	-0.669440217	0.675244692	1
CAB39L	1.789560229	0.221119449	1
AP1AR	-0.523178709	0.639276229	1
TDO2	1.112950194	0.65723543	1
CIR1	0.457102878	0.652795013	1
UHMK1	0.090103896	0.928403759	1
FUT2	2.461807299	0.212796475	1
ERMP1	1.358541594	0.206545709	1
CAPN10	0.782188171	0.463900618	1
PVR	0.550083213	0.696065732	1
PPIE	0.065615557	0.945526391	1
NOA1A	0.459583413	0.561063184	1
CTC1	0.275858537	0.813742736	1
NRAS	-0.36860762	0.653524074	1
MTG2	-0.509645753	0.740841348	1
CEP83	0.160693726	0.892761054	1
GSTZ1	-0.324534752	0.754436727	1
SMURF2	-0.364103236	0.714959498	1
ARHGAP18	-0.379247294	0.70053803	1
MIER1	0.750415766	0.535536815	1
DENND4A	0.173314277	0.856516038	1
PEAR1	-0.494990219	0.601802735	1
TMEM183	1.791308329	0.34776019	1
DNLZ	1.622978318	0.241275528	1
REPS2	2.336269509	0.296644555	1
DEPP1	-0.646670066	0.604003372	1
RAB9A	0.329764244	0.717553037	1
NUDT16L1	1.853793743	0.178759604	1
TP53INP2	-0.233735467	0.814550824	1
PSEN1	-0.021064826	0.983754338	1
LANCL2	0.6500786	0.580039022	1
AP2A2	0.363050926	0.839440681	1
RIF1	0.389702043	0.741826669	1
MNAT1	-0.727426384	0.391408873	1
WASHC4	-0.018773048	0.984170697	1
SMIM14	0.822160464	0.525470362	1
FRAS1	0.363627012	0.802121085	1
CEP295	0.040653041	0.985972688	1
PTPN14	-0.37056949	0.73367726	1
PIGK	0.472998125	0.763524621	1
NBN	-0.489684988	0.753613511	1
NKIRAS1	-1.016448375	0.357747867	1
TSN	-0.236527426	0.807356751	1
MIER2	1.800754169	0.155953833	1
SLC35F5	0.320762012	0.773759392	1
NOP9	0.227747677	0.825852962	1
GBGT1	1.9606153	0.1072964	1
FAM177A1	1.714074445	0.154525877	1
PUS3	0.572305862	0.604032569	1
USP9X	0.17857412	0.883963126	1
PDCD10	0.85763977	0.462483937	1
CLN8	0.530830291	0.641957397	1
SLC27A3	1.781430862	0.118535262	1
ZEB2	-0.772890954	0.400336609	1
TTC33	-0.587112008	0.519306642	1
PPFIA3	1.153442426	0.199798513	1

DCUN1D5	-0.503193213	0.602106947	1
ZNF771	-0.588631419	0.581099322	1
DDX55	0.126693903	0.865956918	1
PISD/PRR14L	-0.054533064	0.959868876	1
STK16	0.318840482	0.738363745	1
ENSMMSG00000004786	0.277770572	0.895476937	1
TMEM256	0.351748727	0.736011515	1
DUSP8	0.479074174	0.731401407	1
MZT2	1.990123339	0.135384645	1
ARHGEF26	1.732927922	0.091883312	1
NSUN4	-0.376534615	0.68501051	1
CBX5	0.499640869	0.673547776	1
PRDX4	0.488385438	0.642754484	1
RARA	-0.487079276	0.652515088	1
RPS19BP1	0.917467705	0.317088566	1
LYPLA2	-0.241446291	0.817838199	1
BMS1	0.093705025	0.935094435	1
ENOPH1	0.057701458	0.953619371	1
RFXANK	1.1499033	0.264580224	1
PPIG	1.032292505	0.402247293	1
TMEM87B	1.707383397	0.128508191	1
KCTD20	-0.175399287	0.911836823	1
NFIL3	0.679519668	0.626223136	1
AR	0.807193111	0.600829208	1
MCM2	1.641832619	0.199604725	1
LETMD1	-0.596295931	0.698813563	1
SLC41A1	-0.343068519	0.822785758	1
YTHDC2	0.045428388	0.959827178	1
DUSP23	-0.688014905	0.517925679	1
MED16	0.376574529	0.713257301	1
CPEB2	0.122411716	0.917875612	1
PLEKHA5	1.243655431	0.33595491	1
SLF2	0.279289554	0.821036952	1
DNAJA3	-0.559557776	0.707200771	1
UBA6	0.219213052	0.817539842	1
COPS7A	-0.75742487	0.539983237	1
ARHGEF11	-1.043407228	0.50511339	1
AP1M1	0.059615737	0.955951984	1
CROT	-0.421958153	0.56265501	1
COA6	-0.623847247	0.548152348	1
OXNAD1	-0.337965213	0.736132378	1
PXDN	-0.612099309	0.505974907	1
TBC1D31	0.091307651	0.931388666	1
TMEM132A	2.584457908	0.171895695	1
RPS6KA4	-0.166734657	0.867277201	1
ELOF1	0.047107671	0.963739403	1
TMED5	0.73942355	0.400576202	1
MRPS21	0.398082926	0.702113136	1
ARAP1	-0.220875085	0.851964204	1
BAD	0.528913903	0.626412722	1
MTX1	0.904512954	0.440957555	1
ZNF652	0.626202426	0.679073136	1
BCOR	0.798665508	0.6615884	1
ABCA9	-0.067175134	0.943482773	1
RDH14	0.339429161	0.811106927	1
PTPMT1	-0.148216535	0.862927644	1
EXOSC4	1.113077628	0.338905566	1
XK	1.313296756	0.47395445	1

GTF3C3	1.088086868	0.428957552	1
DCTN3	0.130678589	0.887052485	1
ENDOD1	2.375854099	0.083886708	1
KANK3	-0.218981625	0.845193622	1
CSRNP1	-0.871867184	0.403653688	1
CTDSPL	1.512085861	0.214939087	1
ITGA5	-0.892594933	0.349532087	1
PNPT1	-0.192610577	0.808146281	1
ENSMMSG00000014419	0.310864197	0.810506079	1
GTPBP2	-0.625937501	0.598799865	1
NIFK	-0.00623858	0.995397665	1
GRIA1	0	1	1
MED23	0.164865368	0.89629459	1
ARRDC1	1.309765404	0.233725365	1
CYLD	0.636355967	0.642222408	1
ENTPD2	1.233088515	0.307064493	1
PAK1	0.515554056	0.74560967	1
SCUBE2	1.094551245	0.375517454	1
LMNB1	1.404437063	0.167067961	1
DDX10	0.207708531	0.840509261	1
UCKL1	-1.304764832	0.361811672	1
MGRN1	-0.168515584	0.912015853	1
TMX3	0.977163324	0.434133369	1
C9	0	1	1
RNF8	-0.47338766	0.605787071	1
SNX18	0.026286713	0.981165167	1
MFSD2B	-0.588564264	0.704450067	1
PIGU	0.717907588	0.536567401	1
RPA2	-0.176011927	0.872173786	1
SH2D3A	0	1	1
KLHL12	-0.385018936	0.709586446	1
SLC16A3	-1.771944718	0.356447637	1
C2CD5	0.259635797	0.86811273	1
AQP1	1.378334856	0.325714613	1
RP9	0.585925981	0.584702621	1
FBLN5	0.564352975	0.734398719	1
TMPO	-0.074685393	0.938361615	1
TAF12	0.050779162	0.964841961	1
PPT2	-0.424200818	0.639734818	1
MAK16	0.193736052	0.845319155	1
DENND3	-0.239055813	0.828660442	1
CIART	-0.521201574	0.590180894	1
INSR	2.357779236	0.571396484	1
KIAA1191	-0.388430028	0.820141753	1
CNST	-0.594162181	0.527059946	1
CDAN1	-0.252859928	0.814449123	1
SLC7A6OS	0.11020196	0.921147344	1
TAOK1	0.18499307	0.878827115	1
ANKRD13D	1.005753329	0.407362426	1
AFF3	1.994962032	0.172094556	1
PAPSS1	0.550834628	0.574878212	1
NDUFA2	-0.474986371	0.610757276	1
ZNF574	-0.204799888	0.852033079	1
GSTM1	-0.440385477	0.704617521	1
ADHFE1	0.705396111	0.501264651	1
TACSTD2	1.442277716	0.178340501	1
MCM3	0.565611018	0.632109406	1
BCNT2	0.109798505	0.922376022	1

ARRDC2	-0.794259723	0.412659786	1
ENSMMSG00000020476	-0.972353964	0.418236901	1
LCN2	-1.873792086	0.391282601	1
USP38	-0.366858057	0.668554869	1
CLYBL	1.093811832	0.378822297	1
JAG2	-1.239766175	0.542113915	1
KANSL3	0.231337478	0.832567276	1
GRK4	0.111461874	0.906493448	1
LRRFIP2	0.661458863	0.702527779	1
MYLK	1.572752552	0.332781186	1
YTHDF1	-0.338704982	0.706039443	1
TRIM27	0.054422097	0.954880216	1
FMC1	-0.397873936	0.705095495	1
CEP192	0.637072598	0.608832743	1
MAPKAPK3	1.053402629	0.452033729	1
USP33	0.284282276	0.799280354	1
HERC4	-0.246479098	0.876701365	1
RNASEH2C	0.130263486	0.906253852	1
GCSH	1.526468145	0.152040876	1
GID8	0.25636223	0.798738208	1
ENSMMSG00000010532	0.613260594	0.661150499	1
ARSG	-0.064698067	0.952859811	1
AKAP13	2.401426484	0.185547981	1
ATXN2	-0.025686538	0.982324604	1
G32876	0.539574468	0.577543285	1
RAB13	0.619863573	0.519380204	1
SPOP	-0.466159635	0.60321269	1
LRRC24	0.731772973	0.534448856	1
PPM1B	0.50761893	0.707326012	1
TMX4	0.454578913	0.634854905	1
LNPEP	0.350068364	0.840916495	1
IRF7	2.278457608	0.100302837	1
SPPL2B	0.284287255	0.730884827	1
NUFIP1	-0.632428338	0.505933833	1
SIK3	-0.724927369	0.598633417	1
TBC1D13	1.473860442	0.350239285	1
TRMT10B	0.998592557	0.356195011	1
NCOA1	0.621153636	0.718905676	1
TBK1	0.073192275	0.946127798	1
MRPS11	-0.595425561	0.493968537	1
G4318	-0.329856821	0.810768273	1
ZDHHC7	-0.045486685	0.97332155	1
NACC1	0.193184396	0.830170877	1
CTU2	1.394959495	0.302087068	1
SPON2	1.558039818	0.199317104	1
MAT2B	0.787495863	0.551809126	1
AKAP17A	-0.168740698	0.880661617	1
MICAL1	0.932904251	0.317194396	1
ADNP2	0.212844802	0.834216655	1
G33790	0	1	1
KCTD2	0.220984205	0.835937785	1
ART1	-0.526287775	0.725785163	1
POLR1D	1.278583917	0.173683234	1
RBM3	0.350012691	0.749808266	1
SENP3	0.210990752	0.78295344	1
MAP2K6	0.22209178	0.875968023	1
TTI1	-0.215674089	0.840917446	1
MRPS6	1.987240964	0.163412596	1

NEK6	-0.30590892	0.842233649	1
KLHL30	-1.013764411	0.380316998	1
ACVR1	0.515381113	0.761031077	1
CNIH4	-0.532959787	0.600129498	1
OSGEP	0.177595594	0.835904043	1
ANP32E	0.920268582	0.440010355	1
LUM	0.855000344	0.540885749	1
SBF2	0.425910221	0.691343494	1
DPY19L3	1.388938302	0.239358797	1
CD247	-0.955871403	0.523774349	1
PRKCD	1.149372741	0.273781287	1
ACVR1B	0.335923135	0.844167577	1
TENT5C	0.396211385	0.808947977	1
EML5	0.829939507	0.540715125	1
KLHL24	0.191294896	0.889341458	1
GOLPH3L	1.478301236	0.245967179	1
PANK3	1.68954071	0.338602391	1
LIMD1	-0.505732847	0.603940427	1
MEMO1	0.080800249	0.946631541	1
SCPEP1	-0.2788843	0.796485602	1
FBXW4	-0.559227858	0.635562512	1
URB2	-0.448848916	0.627045291	1
TMOD3	0.304082733	0.756215574	1
ELMOD3	-0.83893234	0.42201629	1
SEMA7A	-1.043109761	0.291710113	1
ARHGEF17	-0.365398979	0.73319729	1
IMP3	0.050015725	0.952551153	1
RNF114	1.103528339	0.412625736	1
ZCCHC24	-0.909843111	0.572116763	1
PHTF2	-0.650328678	0.652244698	1
ST3GAL5	1.537863838	0.122623049	1
CDKN1B	-0.309179889	0.822832083	1
EXOSC7	0.147332482	0.841756449	1
LOC530599	0.385655372	0.686241632	1
PHIP	1.302878048	0.321213313	1
RPS6KA5	-0.583152517	0.714967488	1
PRRC1	0.508727261	0.60080689	1
CACNG6	-0.574300705	0.698586062	1
ATP2B4	0.202057334	0.860881735	1
GPSM2	1.552231069	0.251931271	1
RRAGC	0.25237358	0.798702321	1
RAB6A	-0.385578493	0.681553359	1
OSBPL11	0.342034678	0.844772363	1
AKTIP	-0.324182512	0.705847089	1
MIEN1	0.651602918	0.541647521	1
PHF14	0.64882217	0.609630738	1
SFT2D1	0.273091888	0.771710836	1
HYPK	0.225081498	0.830749321	1
DECR2	-0.292827903	0.791610674	1
TFG	-0.258080649	0.781863757	1
CALU	-0.27624668	0.776346037	1
TMEM160	1.269705708	0.288571348	1
RIPOR3	-0.018843678	0.985023625	1
ACTR1B	-0.261051437	0.809317416	1
AMH	-0.017984263	0.982178479	1
WFS1	-0.519038491	0.676898397	1
DCXR	0.949755484	0.469877823	1
LOC788915	-0.554145933	0.643082875	1

DNAJC16	-0.249282461	0.874633627	1
TBCD	0.797775355	0.449846704	1
POLD1	1.040790198	0.34639377	1
CHMP1A	-0.052642054	0.961468567	1
ZNF143	-0.636069027	0.52645792	1
SH3PXD2A	-0.042593451	0.971653253	1
NFRKB	-0.099496475	0.92466471	1
P4HA1	1.718270501	0.343410388	1
SLMAP	1.299721823	0.42131738	1
RNMT	0.966814871	0.462704742	1
DDHD2	0.264480473	0.816216166	1
TASOR	0.602425004	0.612087781	1
PI4KB	0.609363385	0.67300783	1
MAP3K5	1.98772717	0.150229714	1
RSF1	0.024001272	0.981863765	1
TECPR2	0.246762302	0.881235684	1
MSH6	1.013262307	0.446211335	1
DMD	-0.063201608	0.963396389	1
MBNL1	-0.040434197	0.980676026	1
STAT5A	-0.543283686	0.632471867	1
CEL	1.135809048	0.363626824	1
RNF25	-0.010526159	0.991584197	1
IP6K3	-0.754187864	0.528576378	1
BTBD10	-0.731355247	0.481853451	1
SLC16A1	-0.716882462	0.52214493	1
COX10	-0.886000864	0.294623506	1
GAT	0	1	1
CRACDL	0.805067207	0.486626551	1
NDUFV3	1.683389842	0.187820767	1
SLC46A3	1.047661066	0.495950508	1
LMF1	0.879798573	0.298398078	1
PRMT3	-0.195714445	0.830859583	1
HSPA12B	-0.94063985	0.220347057	1
TUBB6	0.150440851	0.902389103	1
LATS2	0.5881049	0.667010219	1
MLLT10	-0.30466562	0.84550951	1
BOLA-DRA	0.444421907	0.666404342	1
LARP7	0.010549268	0.992213798	1
QPCTL	1.736232355	0.119436462	1
RMDN3	0.830585585	0.535410446	1
RO60	0.639529067	0.569713705	1
ARF5	-0.194622265	0.867361791	1
TUSC3	1.425862679	0.217954787	1
CTDSP1	-0.359200731	0.749988149	1
ZW10	0.024578172	0.98106876	1
RRAS	-0.641713256	0.553099722	1
FNBP4	-0.219723625	0.82719822	1
NDUFA5	-0.839553642	0.361097378	1
KDM2A	-0.344078141	0.82904839	1
FAM149B1	0.527619635	0.653060134	1
UNKL	-0.269221315	0.810262124	1
SFXN5	1.169489765	0.244788379	1
AKT1S1	-0.54488593	0.601991649	1
CTDSPL2	0.053766112	0.960557963	1
LRP3	-0.938517136	0.338735419	1
RNF7	-0.958814058	0.291427544	1
INTS5	0.034707694	0.977265796	1
GLRX5	-0.197002993	0.83838347	1

RBM15	-0.178585948	0.882815476	1
L3MBTL2	0.878209729	0.408232498	1
LTBR	0.287444021	0.751731676	1
JOSD2	0.171265275	0.878658569	1
MYDGF	1.223954581	0.23641161	1
PHAX	1.26572258	0.237157743	1
GPATCH3	-0.199195012	0.792815839	1
DMD	-0.328984813	0.832125524	1
RAPGEF2	-0.437974446	0.867040217	1
TTF1	1.316103973	0.304266271	1
CILK1	0.212830381	0.797189268	1
CHCHD1	0.41962482	0.710696496	1
TSPAN31	-0.159741742	0.848048551	1
FAHD2A	-0.252406597	0.823261761	1
STOM	-0.350123001	0.818853574	1
DPAGT1	0.84617432	0.439193035	1
SEPHS1	0.116239993	0.907395438	1
SNX21	-0.096875111	0.925729492	1
DNAJB4	-0.032618412	0.979901683	1
SERPINF2	0.324624235	0.880976033	1
KLHDC10	-0.329614091	0.718586993	1
CD93	-1.028829331	0.272076927	1
BMPR1A	0.976002681	0.456563194	1
MTSS1	0.110876666	0.901294827	1
MFHAS1	0.96292081	0.310662588	1
TDRD7	-0.020384274	0.981286431	1
POMC	0.138808681	0.894639282	1
MALSU1	-0.13279523	0.903454883	1
MAD2L2	-0.339137912	0.721442192	1
ZNF518B	0.762101992	0.50965716	1
SYTL1	1.514445251	0.138791125	1
MCM6	0.696135443	0.532750011	1
DOT1L	0.330166129	0.729777679	1
TBL1X	1.314163335	0.34886455	1
BTG1	-0.835905916	0.297001156	1
ENY2	-0.479078705	0.634490183	1
ZFAND2B	0.134490482	0.879227138	1
ACSF2	1.876344363	0.139542135	1
INSR	0.173126968	0.918551348	1
HMGN3	0.298670068	0.783158485	1
HES1	0.024126747	0.980703692	1
CSF1R	0.005035661	0.995858473	1
MRPL20	-0.212185065	0.817852347	1
DCBLD1	1.148086247	0.392136939	1
PTPRT	0	1	1
LOC112447087	-1.092585437	0.508101102	1
NUDT21	0.183554287	0.862011488	1
DDIT3	-0.328400735	0.729993846	1
ARL2BP	-0.240924836	0.829533848	1
BICDL1	0	1	1
C7H19ORF53	-0.122215076	0.909490186	1
BLZF1	0.123997278	0.906965051	1
TGIF1	1.740298987	0.277116535	1
MAPK7	0.225466174	0.832745251	1
PARN	0.273647926	0.796535112	1
NDUFS3	-0.230279365	0.838322451	1
PEF1	0.544442398	0.535056601	1
CC2D1A	0.582186473	0.578582618	1

ZFP64	-0.411911444	0.673096904	1
SMIM7	1.558964225	0.156607534	1
H1-12	1.299588083	0.369891279	1
ISOC1	0.661354239	0.484685747	1
RUSC1	0.341546285	0.706084404	1
MYG1	0.241129642	0.824849338	1
DTNA	-1.102670429	0.188177064	1
AP3S2	-0.534405759	0.602534568	1
TYRO3	1.75043973	0.113842934	1
R3HDM1	-0.319934674	0.842503671	1
SNX6	0.219326587	0.849444123	1
IL6R	-0.885017712	0.393977748	1
TNRC18	0.235240423	0.807789624	1
CAPN15	0.183143584	0.857790751	1
FBXL4	-0.785698863	0.413422453	1
EPC1	0.121169066	0.941316516	1
YEATS2	0.670618062	0.581295664	1
RAD17	-0.194614992	0.89757215	1
TIPRL	-0.226676028	0.803732366	1
AMBRA1	1.068854604	0.244065842	1
ISG20L2	-0.371856968	0.725400771	1
SEPHS2	-0.633102715	0.495043859	1
ANK3	0.439176497	0.860064747	1
TRAF3IP1	0.845006251	0.384416508	1
OTUD3	-0.056455971	0.954791057	1
CREG1	0.91296754	0.386622448	1
E2F4	-0.611885452	0.524660453	1
TRIB1	-0.53862318	0.632643246	1
PLBD1	0.8295397	0.458508991	1
ARFGAP3	0.198135492	0.898051261	1
RSPH6A	-0.395097101	0.678584303	1
CDH13	0.201024739	0.884942148	1
CROCC	-0.062044869	0.949696882	1
IFITM3	0.734727585	0.52612624	1
MED10	0.556277339	0.590789428	1
FOXO1	-0.740077876	0.534414302	1
GHDC	-0.03758821	0.97264315	1
EXOC5	-0.393118493	0.719795798	1
ATN1	0.091416357	0.923285908	1
HSD17B11	1.274720865	0.418573139	1
ST3GAL6	-0.030131342	0.978871445	1
LOC515333	-0.538963141	0.577444245	1
UBALD2	-0.265158025	0.83176296	1
MRPL19	-0.255791841	0.77359386	1
CDC37L1	0.049549167	0.963065581	1
ALG12	0.576727601	0.546150797	1
TXNRD3	0.700568068	0.534743076	1
FRK	2.112397221	0.222905782	1
PRSS23	-1.199733496	0.158739907	1
CPED1	0.110191668	0.929861411	1
METTL9	0.999628201	0.3069658	1
NOL6	0.565234951	0.606998704	1
TMEM184B	1.604349885	0.369161426	1
CAVIN2	-0.678854688	0.669323717	1
SRSF4	1.677404244	0.152736228	1
ZKSCAN1	0.695758746	0.510334271	1
IMPA1	0.364678104	0.746875932	1
HARS2	-0.52503126	0.734679727	1

NAA38	0.393414726	0.706672926	1
QSOX1	0.628866324	0.47488105	1
YTHDF3	-0.232995848	0.782848323	1
DCAF12	0.504485048	0.604518838	1
LIN7C	0.67530436	0.539047097	1
PCBD2	0.715426931	0.510065947	1
SGSM1	1.549605572	0.321757596	1
ANAPC5	0.401678464	0.691185704	1
VPS33B	0.704963908	0.518672134	1
IGFBP4	-0.236272622	0.82324007	1
TBX10	1.017827496	0.253690011	1
DAP	1.166921399	0.251761525	1
ZNRF1	-0.706800367	0.537555465	1
MRPL51	-0.469110665	0.619289263	1
MAML1	-0.079919683	0.937012266	1
UTP4	0.176538289	0.850485553	1
POLR3K	-0.878941201	0.337279503	1
PHLDB1	-0.849437081	0.604999955	1
RASL10A	2.532923186	0.116782553	1
LIAS	-0.656960414	0.514444948	1
ZCCHC2	0.238699847	0.813202799	1
NLGN2	0.9278502	0.354268423	1
PIGB	1.146885504	0.329687326	1
MED1	0.010699626	0.992622537	1
LIPH	0	1	1
CHMP4A	0.886856794	0.408456521	1
ESF1	0.583449652	0.633722719	1
ARAP3	-0.659570927	0.459460667	1
PPP1R21	1.036068591	0.456801243	1
TTC28	-0.626660924	0.533677869	1
PGM3	1.435266963	0.274325234	1
LUC7L	1.995260191	0.185239952	1
DUSP6	-0.228504968	0.826448376	1
PLTP	1.332138108	0.184877842	1
UVRAG/DGAT2	-0.002280844	0.99845078	1
G3BP2	-0.018159975	0.98776611	1
MPP1	-0.242974526	0.810463153	1
ABI1	0.081506808	0.95779336	1
RIPOR2	-0.034520046	0.984482158	1
CUL4B	0.551871454	0.514689546	1
FBXL6	1.399375889	0.223717891	1
POLA1	-0.289148517	0.779168882	1
PLOD3	-0.115374405	0.889423152	1
LOC529036	3.959731876	0.105635965	1
MAP4K2	0.82115409	0.400316865	1
SLAIN2	0.21880871	0.856421276	1
MGAT1	-0.390059937	0.810899792	1
SIVA1	1.196085034	0.285748789	1
CIAO1	0.469074739	0.69483866	1
UBQLN1	0.204066996	0.896309729	1
HCFC2	-0.234368227	0.811910428	1
SCAND1	0.40687143	0.658690935	1
TSC2	0.618204489	0.582436409	1
CCDC22	-0.454718442	0.691652714	1
ENTR1	-0.460033759	0.661536923	1
B3GLCT	0.877038773	0.348571339	1
RAVER1	0.129035722	0.897054526	1
USF2	-0.619537132	0.513150536	1

TMEM143	-0.885809787	0.262336082	1
MAPK9	-0.286031786	0.857683942	1
PDLIM4	1.534583383	0.109660419	1
SIRT7	0.662063338	0.538336611	1
AIFM2	-0.305574711	0.842839318	1
USP6NL	1.553334634	0.11776504	1
PIK3CA	-0.025399374	0.987250832	1
LRRN2	0.306067476	0.748896353	1
ENTPD6	0.709582324	0.59507322	1
MCEE	-0.204662388	0.823042871	1
OAZ2	-0.116758498	0.924910199	1
INTS7	0.683115915	0.473121625	1
NENF	0.213710527	0.860009689	1
EML3	-0.057939246	0.974648672	1
ARID1A	-0.076259768	0.962037399	1
WDR61	-0.042192476	0.958069875	1
U2SURP	1.677018313	0.338660393	1
DUSP16	1.39262691	0.311350874	1
TTC14	0.627238041	0.598577599	1
DHX34	1.678288252	0.175509605	1
LSM3	-0.126693739	0.903509337	1
CALR	0	1	1
LMO4	-0.001510041	0.998882119	1
ENSMMSG00000005999	-0.32540636	0.722035405	1
DBNL	0.424146853	0.63301552	1
PICK1	0.265079106	0.790664898	1
YAP1	1.15292656	0.422177703	1
FBXO44	0.778694604	0.51734774	1
SYS1	0.104476416	0.92902291	1
LOC112445177	0.618828724	0.640038415	1
GUF1	0.052458217	0.956334798	1
SYAP1	0.617590733	0.52680519	1
NCDN	-0.74200713	0.501195873	1
PIGL	0.970339968	0.310532178	1
SMARCA1	0.12345059	0.942371271	1
DHRS11	-0.327078387	0.718707436	1
MARK2	-0.212335333	0.797713118	1
MGA	0.655412301	0.704093115	1
ALMS1	1.776025713	0.185399393	1
TTC27	-0.098610869	0.914918995	1
UBE2D4	-0.437055913	0.781456754	1
SEC16B	2.673228765	0.083107479	1
TRAPPC1	0.033952721	0.972533046	1
SERINC5	0.659047379	0.538124843	1
CTSH	0.06821336	0.944709162	1
ENSMMSG00000009431	-0.142895361	0.913417362	1
ETS2	-0.787819235	0.456078509	1
PRR14	0.467331149	0.642962468	1
CHP1	-0.170238591	0.832131853	1
HPS4	0.92814136	0.507121115	1
AXL	-0.272327713	0.751575264	1
COX20	0.341658536	0.772889061	1
TRMT1	1.3086378	0.322902264	1
PARP4	0.27829572	0.776689865	1
SARS2	1.158666313	0.349309183	1
MCRIP2	-0.851825076	0.365113836	1
RTF2	0.119524964	0.884356486	1
UBE2D1	-0.228761725	0.890579924	1

SLC49A3	0.852789559	0.340832252	1
KMT2E	-0.02243186	0.983604849	1
GPR89A	1.042259217	0.418914121	1
CAMK2N2	0	1	1
HMGXB3	-0.150860858	0.922428241	1
SLC35A4	-0.497252353	0.605969517	1
CCS	-0.126276074	0.917689743	1
SRP19	0.110238218	0.915158022	1
IBTK	0.516366459	0.679159427	1
RAE1	0.710815164	0.574694917	1
ZNF524	0.52957605	0.675116464	1
SHKBP1	0.692331459	0.540886078	1
GPALPP1	1.053592076	0.434739287	1
NCKAP5L	-0.06510816	0.941563486	1
MAPK8IP1	0.116868488	0.909890785	1
PREB	-0.682538586	0.538456778	1
DNASE1L1	-0.23222932	0.878604385	1
RCOR3	-0.409335991	0.599551387	1
CCND3	0.429334197	0.690041629	1
PTCD1	0.016266325	0.990103068	1
NUP160	-0.205337196	0.896136951	1
INTS4	0.183271119	0.843943944	1
RCHY1	-0.223213166	0.886118385	1
NXN	-0.628052928	0.496614269	1
ZNF777	0.254708277	0.85068074	1
NTMT1	1.035255909	0.426656208	1
MAN1A2	0.142193571	0.891129127	1
MXD4	1.089553995	0.368841497	1
MAST3	1.3344398	0.205511078	1
TIMP1	1.949234099	0.131444359	1
LOC100299281	1.090878717	0.296576073	1
EXOC2	1.840211379	0.13023728	1
BIRC6	0.771153579	0.502406158	1
COQ2	-0.292628252	0.853326259	1
POLR1A	0.967896597	0.444082127	1
REEP3	0.712888484	0.465181549	1
FAM20B	1.253137223	0.374919306	1
ITGB6	0.243952573	0.862510807	1
DOP1A	0.400457573	0.692938035	1
GTPBP3	-0.016885052	0.987500297	1
ATP13A3	0.566234302	0.637348697	1
TOP3B	0.022693059	0.985801658	1
COL6A2	0.332615344	0.75505496	1
SPRYD7	-0.779629434	0.423612822	1
FRMD3	-1.065668308	0.463211579	1
PSME3IP1	0.224052314	0.815498176	1
URB1	0.119451025	0.896481395	1
USP21	-0.127801197	0.895374483	1
EIF4E2	0.373812761	0.706972882	1
OTUD6B	-0.557202101	0.57586037	1
ATXN2	-0.370962999	0.809762892	1
NECTIN2	1.554390129	0.275614096	1
PIGX	1.171520692	0.332909898	1
TCIRG1	0.479681577	0.678005993	1
CWC27	0.155778056	0.883278644	1
BAG2	0.398191383	0.756870248	1
ARHGAP17	-0.724164181	0.736006943	1
ARHGAP45	1.373804158	0.17389902	1

RAB23	-0.143093329	0.927340001	1
LRRC20	-0.881716595	0.579318209	1
AADAT	1.719688438	0.226384716	1
TMEM9	0.435442771	0.659471941	1
PDGFA	0.110343483	0.91522157	1
SNRK	0.224392851	0.85170251	1
RAB3IP	0.181845732	0.859553698	1
KLF6	-0.674919996	0.544734797	1
PQBP1	0.281745919	0.810086455	1
PCK2	1.944347086	0.085242688	1
NR2C2	-0.363583242	0.720388156	1
POGLUT3	0.564720297	0.560325819	1
DTX3	1.669507862	0.233839913	1
TMEM19	0.400668757	0.787727367	1
PRKAG1	0.587817792	0.655914143	1
ORC4	0.240782447	0.843333404	1
DUS1L	0.056162483	0.951843203	1
PIAS4	0.206081195	0.78708931	1
SIK1	-0.663620906	0.455943162	1
POLR2I	0.644475337	0.558867281	1
CRYBA4	-2.849451224	0.150917276	1
GRTP1	1.315186204	0.129933892	1
IBTK	0.473154381	0.697000544	1
ZNF330	0.979337663	0.463196552	1
HLTF	1.029117079	0.41142189	1
NUMB	0.334468432	0.778372414	1
CNOT7	0.722847759	0.554206117	1
ARRB2	0.322178656	0.711411583	1
TERF2	1.144695513	0.406270611	1
CBFA2T3	-0.435633575	0.704943134	1
TGFBRAP1	0.212281768	0.848591511	1
RHOBTB1	-0.885321471	0.602524269	1
ZNF839	0.84570875	0.509606797	1
KLF3	1.096229045	0.334326299	1
STRBP	-0.021301839	0.989164314	1
TIMM21	-0.400138575	0.664634131	1
VGLL2	-0.422404527	0.780635594	1
HS1BP3	0.985667629	0.349048468	1
ATG101	-0.594877434	0.507076798	1
ZNF236	-0.130538069	0.87554723	1
ANKRD13A	0.096984005	0.93350616	1
PITPNB	0.4797364	0.750305865	1
UGGT1	0.669739349	0.550222028	1
PGAM5	-0.411582858	0.670838498	1
SPG11	0.954582389	0.579159312	1
DYSF	0.224228519	0.899825371	1
ACSF2	-0.379587241	0.750395018	1
TBC1D10A	0.915990147	0.368687371	1
TMEM181	0.274517843	0.79139817	1
COBL	-0.927255881	0.431139037	1
ALDH16A1	0.997710655	0.33615366	1
BRAP	-0.608162382	0.518820213	1
ZNF326	0.839077223	0.521191982	1
MYH14	-0.508332378	0.720991763	1
BRD1	-0.532812119	0.71508412	1
ARHGAP10	-0.070678836	0.949970866	1
MXRA8	-0.404935083	0.649954737	1
DBN1/PDLIM7	0.730601021	0.503257992	1

CLPB	0.283208099	0.746480061	1
DMAP1	0.022390717	0.984829957	1
MED4	-0.469393751	0.604470772	1
PRPSAP2	0.772618673	0.435645157	1
RBBP6	0.302896906	0.777962909	1
SDF2	0.299599246	0.6987629	1
CMIP	0.502829173	0.628367829	1
ENSMMSG00000022774	-0.170180971	0.877100289	1
GNG11	0.749644428	0.530505073	1
SPP1	0.359829861	0.824706684	1
SLC10A3	0.632741622	0.501328778	1
ABLIM2	-0.002044268	0.99895564	1
EBAG9	-0.448030862	0.675291745	1
COQ7	0.619922571	0.574077624	1
PHLDB1	-0.536287251	0.753037953	1
PPP2R5B	-0.613138809	0.518173206	1
DHRSX	0.952650802	0.282851284	1
FKBP1A	-0.376199631	0.689494949	1
TUT1	1.336172421	0.256889951	1
GDF1	1.281148503	0.318377728	1
PPIP5K1	-0.019258692	0.991268496	1
TRAFD1	0.18373917	0.863145179	1
METTL22	-0.09121778	0.931371615	1
ERMARD	-0.062802196	0.948996457	1
RAB8A	1.110533702	0.146037889	1
PARVA	0.252696169	0.868812132	1
YIPF2	0.13642355	0.905961501	1
CECR2	-0.749130408	0.506301173	1
ANK1	-0.959082326	0.412035408	1
GFPT2	-0.819353271	0.325908538	1
PHF13	-0.225397869	0.814145053	1
MALT1	-0.202653356	0.844791124	1
TLE3	1.893156263	0.143487053	1
TFEB	-0.915576257	0.376605204	1
KCNH8	0	1	1
TPRA1	1.467931514	0.271076795	1
IGFBP6	1.029196677	0.399341509	1
PTP4A1	-0.329090365	0.82580221	1
MRPL22	-0.018516427	0.986643905	1
COL26A1	-1.829075492	0.408069505	1
TMEM190	0	1	1
DNAJC12	1.36638945	0.11158191	1
SEMA3B	1.124167276	0.282826846	1
ARHGEF11	-0.771794231	0.597317112	1
CDC42EP3	0.602034108	0.72991594	1
MTX2	0.09009303	0.953545058	1
DGKA	0.766376917	0.572463874	1
SGMS2	1.72026497	0.134417341	1
CHERP	0.168202316	0.913297537	1
CIPC	-0.773797466	0.428990229	1
NUP58	0.362316929	0.778168982	1
TEPSIN	0.63081035	0.433884135	1
PRAG1	0.662166688	0.580581332	1
ASAP3	0.51232435	0.605264726	1
RGS5	-0.847209261	0.546832906	1
BRAT1	0.63386939	0.431118756	1
ZFYVE27	0.468092312	0.714196249	1
TFAP2B	2.606366471	0.255798996	1

NRDE2	0.574374003	0.668530338	1
DAB2	-0.374699558	0.681536884	1
APEX2	-0.630235828	0.485738468	1
MTFR1L	-0.554802394	0.514073687	1
SMC3	0.633307935	0.619959477	1
GSTO2	-1.874287148	0.392671124	1
TRUB2	0.313039174	0.815034366	1
LACTB	-0.535181755	0.557586112	1
SAAL1	-0.404207449	0.798711458	1
PAICS	-0.013974062	0.989119001	1
WLS	-0.331805072	0.768126378	1
DCAF7	-0.24008953	0.813964663	1
ODF2	0.389898807	0.706884248	1
DAGLB	0.277007395	0.830087076	1
XPO5	0.428949873	0.650581294	1
ZGPAT	1.238204915	0.333087523	1
SOS1	0.137268291	0.935624008	1
ITGB1BP1	1.557506496	0.254889496	1
TRIM55	-0.371846528	0.820939973	1
NFS1	-0.42365568	0.679710284	1
PALM2	-0.666136712	0.499285334	1
SCYL3	0.063626872	0.947162484	1
COLCA2	3.124485523	0.096151105	1
SLC15A4	-0.453185781	0.632502256	1
UBE2D2	-0.245742969	0.800124016	1
CHUK	-0.088002656	0.956573803	1
EIF4E3	-0.651861473	0.687590232	1
DIP2B	1.238648595	0.340375455	1
LSM12	-0.333127443	0.684377913	1
OTUD1	-1.255270326	0.265071993	1
DNMBP	1.563254274	0.258974526	1
CHFR	0.185541415	0.838880694	1
BMPR2	-0.062013511	0.955802892	1
ACSS2	0.366327222	0.86500649	1
SEMA3A	1.79211686	0.226969889	1
DMD	-0.146699933	0.930892271	1
RHOBTB2	-0.960436284	0.376155517	1
HPS3	0.284243557	0.848660707	1
ATP6V0A1	0.236648528	0.816770012	1
DBNL	0.62539437	0.533100367	1
PPP2R5C	1.196288069	0.5051379	1
A1BG	1.593720846	0.162453924	1
ALG9	1.760970325	0.125569999	1
KALRN	-0.82820727	0.58012757	1
ACTR3	0.229728932	0.801865673	1
ABCB8	0.833466072	0.448060071	1
CDPF1	-0.04422576	0.966375895	1
NEURL1	-0.91268168	0.539253701	1
ZNF598	0.23974645	0.815630119	1
LARS2	-0.43658948	0.654766497	1
ANGPT4	1.574707844	0.242508783	1
SH3BP5L	-0.199989417	0.844326744	1
CD164L2	0	1	1
NGDN	0.033688117	0.970536437	1
PIK3C2B	1.791454363	0.196679507	1
DIS3L2	-0.247338993	0.819583925	1
CRAMP1	0.308182521	0.795553653	1
MECP2	-0.208136104	0.849481495	1

RBM33	0.075469202	0.943684085	1
CCDC12	1.316484653	0.362110307	1
MAX	-0.41646428	0.652231162	1
MRPS9	0.044627914	0.962129804	1
RANBP10	-0.522555737	0.530173274	1
ANKRA2	1.220148199	0.363194537	1
EIPR1	0.346485719	0.758854339	1
POLR3E	-0.255889254	0.806596176	1
AHCYL2	0.472147468	0.783505403	1
MVB12A	-0.162583167	0.887235633	1
WNT3A	0	1	1
IGFBP7	-0.508034658	0.629151104	1
SNAP23	-0.452693905	0.77643106	1
COX15	-0.217871442	0.820593085	1
TMEM219	0.46578336	0.662631723	1
AOPEP	0.206550672	0.880959419	1
SNED1	0.298088609	0.793332387	1
UNC45A	-0.993576667	0.373856964	1
HDAC3	-0.457729011	0.530041911	1
NDUFAF7	-0.431324166	0.653734314	1
ZC3H18	1.125569402	0.398427328	1
SEC61A2	-0.619074149	0.486470788	1
SLC25A42	0.730407189	0.691981255	1
NPRL2/TMEM115	-0.245364877	0.791530258	1
SMYD1	-1.136996905	0.276885285	1
RIC1	-0.591443906	0.561499838	1
UBE2G2	0.875440843	0.49608585	1
KIF13A	1.832435055	0.222155438	1
MYOF	6.19303233	0.160438051	1
PPP4R2	-0.070445819	0.94268643	1
TSEN54	0.187152953	0.850746427	1
RB1	0.845899783	0.515131874	1
SLK	0.033913676	0.977019345	1
PPM1G	-0.27870706	0.75978955	1
ATG7	1.21982268	0.367386094	1
BHLHE41	-0.634470713	0.593351245	1
PLRG1	-0.587122584	0.551886798	1
EFHD2	1.403003127	0.248252348	1
TFE3	-0.084997368	0.937460819	1
WDR74	0.933528699	0.473659353	1
NELFE	0.710886167	0.528788195	1
SLC35B2	-0.017981394	0.984486642	1
ENSBTAG00000006937	0.074580704	0.953602128	1
CNOT10	0.004684379	0.995956011	1
CACTIN	-0.333900829	0.745900504	1
MYOD1	-1.134154057	0.436475454	1
PMM1	1.389246505	0.252579493	1
NEDD1	0.356600726	0.837522436	1
HOMER3	-0.500117996	0.597227791	1
N4BP2L2	-0.558848786	0.571219255	1
PPP6C	-0.261909995	0.806485338	1
INSRR	0	1	1
MVK	0.094072824	0.915889815	1
FAM210B	1.220617593	0.216030576	1
TIMM17A	-0.614591344	0.545392931	1
PIGN	0.61763341	0.565326768	1
SNX4	0.708760385	0.549799711	1
SLC30A9	0.156584433	0.864572546	1

MFSD1	-0.035821804	0.972016498	1
MRPS26	0.491800512	0.59245949	1
AIMP2	0.15089069	0.902366837	1
GTF2H1	-0.204215623	0.847582174	1
COMMD1	0.629210528	0.604479943	1
RETREG2	0.451444005	0.719220699	1
RFNG	-0.548896758	0.578343964	1
MBNL1	-0.684886111	0.518907215	1
AARS2	-0.648288236	0.506667052	1
MRC2	0.864053852	0.510375472	1
PPFIBP2	2.831683312	0.12750425	1
CLIC4	0.384077594	0.734505162	1
SDAD1	0.949661949	0.409909577	1
ITPKC	-1.829266702	0.406680906	1
MCU	0.381478419	0.788605242	1
NLK	0.611373469	0.68002777	1
NOC4L	-0.746176775	0.401542858	1
HAT1	0.46387825	0.687304186	1
KCNA7	-0.970153659	0.416818345	1
FANCA	0.004135758	0.996228272	1
APH1A	0.157780149	0.874875305	1
AGPAT5	-0.110647047	0.912615859	1
RWDD4	-0.46080913	0.664777921	1
PPTC7	-0.801724975	0.625451423	1
DEAF1	-0.184977256	0.836621716	1
FAM117A	1.210843849	0.469309425	1
NDUFAF8	0.327442154	0.777173877	1
G31115	0.834066699	0.326452478	1
DMPK	0.229497959	0.896580259	1
PKN2	-0.145501843	0.887487142	1
RRP1	0.050773053	0.957255301	1
GALNT2	-0.419529115	0.715763172	1
LEMD2	-0.044215594	0.963657931	1
RTL8C/RTL8B/RTL8A	1.233238252	0.364551487	1
WDR19	0.181085958	0.836287254	1
ZMYND10	0.038378442	0.968560564	1
CACNB3	0.244962301	0.820968956	1
PLEKHM1	0.152582641	0.894915039	1
RNF14	-0.614383244	0.702854074	1
ADCK2	0.504670516	0.670583482	1
G3BP2	-0.439464397	0.783476933	1
TAF2	1.225223721	0.424433572	1
EML2	1.415066574	0.159446751	1
LOC507055	0.434323918	0.773208892	1
TRIT1	1.177834205	0.390029106	1
SLC39A1	0.650195345	0.535592738	1
AFTPH	-0.266315794	0.796798482	1
STX4	-0.30914186	0.76271159	1
WDFY1	0.056588803	0.954841584	1
TLK2	0.215045353	0.852804862	1
ADCY4	-0.427127733	0.660692924	1
PKD2	-0.020259225	0.990009804	1
TMEM97	1.681327925	0.123885999	1
PRKD2	1.113578471	0.367873278	1
PDE4D	-0.62047022	0.669754704	1
UBE2D3	-0.677243004	0.599471052	1
PLCG1	-0.229958853	0.882903029	1
CNKSR1	0.563352417	0.690941879	1

ELP1	-0.628318123	0.710047856	1
RAD52	-0.880175785	0.382638611	1
NF1	0.210093686	0.860812581	1
GPHN	0.915119094	0.506563017	1
SESTD1	-0.06477412	0.967954076	1
CCNYL1	-0.222382111	0.840338033	1
CNNM3	1.148672191	0.362413781	1
TIAL1	-0.46325572	0.681380443	1
HERC3	0.619609019	0.66522461	1
CAPN1	0.421357463	0.752706339	1
SLC25A33	0.451218322	0.710157604	1
TBL2	0.146122401	0.888155781	1
TUBGCP5	0.029139099	0.985228167	1
LOC518768	-0.18516776	0.859666838	1
ALG3	0.950894504	0.357723035	1
RCN1	0.697139008	0.590218192	1
RIOK2	0.888308158	0.487511621	1
YLPM1	-0.011709952	0.991321187	1
VPS18	-0.222245862	0.840162805	1
SON	-0.52035705	0.557255273	1
CAMK2D	-0.599870027	0.667585658	1
KIF16B	1.818448718	0.119559367	1
KCNJ11	-0.744339414	0.605754235	1
ARHGAP39	0.680286101	0.50877448	1
ATG4A	-0.777331146	0.616846037	1
LGALS3BP	0.298586867	0.737150301	1
CAPZB	-1.022983206	0.50533256	1
CRK	0.231656089	0.811434391	1
RC3H2	0.183922255	0.836764539	1
UNC45A	1.633195961	0.11301549	1
TGM2	0.509798007	0.722900171	1
UBE2Q2	0.324598769	0.79606018	1
ARID1B	0.074693579	0.957792872	1
TNFRSF1A	-0.064589463	0.94587164	1
ZNF692	0.13512172	0.887274196	1
LRRC8A	-0.353650648	0.673871153	1
CEFIP	-0.446611634	0.753920795	1
MED21	-0.326927392	0.742716288	1
REPS1	0.505037597	0.752950892	1
ACAP3	2.810618669	0.256416487	1
G8616	0.770494283	0.444974787	1
ZMIZ1	0.133335293	0.899112115	1
ZCRB1	-0.705339305	0.448831914	1
EP400	0.781251522	0.766502628	1
CDK4	0.258891255	0.778121321	1
FOXK1	0.03963418	0.971483301	1
PPP2R5C	0.003818401	0.997057845	1
PSMF1	0.369621185	0.82559301	1
ACD	1.217033527	0.250546288	1
LIFR	-0.601250221	0.474304084	1
DUSP13	-0.876624734	0.465160434	1
VAMP2	-0.225699111	0.844601904	1
HSPB3	-0.268930537	0.854614692	1
SNAI3	-0.165708853	0.891997248	1
ANKS3	0.10848904	0.902802426	1
MYC	-0.578792251	0.589802496	1
SRRM1	0.209424864	0.897223534	1
MCRIP1/LOC101908069	1.265898368	0.264216604	1

AFDN	-0.002742699	0.998036333	1
TAF1C	0.344457045	0.76549954	1
FNBP4	-0.357557644	0.722863193	1
MYB	0	1	1
MRPL33/LOC101904449	-0.405352257	0.702534683	1
SPNS1	0.184012225	0.857867655	1
MAPK8IP3	0.669253689	0.611969005	1
ATP1B4	-0.070190677	0.957434562	1
ABT1	0.227063188	0.860145519	1
EXTL3	0.054986195	0.960987369	1
NT5C3A	-0.68080252	0.458852243	1
SMCR8	0.106626094	0.919122011	1
VSNL1	0	1	1
ENSMMSG00000017998	0.844640478	0.547784108	1
MITF	-0.842102968	0.578428629	1
CLIP3	0.316320688	0.767284717	1
GPD1L	-0.369747578	0.826115973	1
SIX5	1.099486374	0.234632685	1
GTPBP6	-0.061877476	0.953921341	1
MAP3K3	0.710799366	0.645363239	1
TRIP11	1.09474584	0.529554851	1
ISY1	-0.065742617	0.950624765	1
RANBP3	-0.434074974	0.792849737	1
MTHFR	0.52826115	0.640545179	1
SMARCB1	1.992081041	0.168021148	1
PLEKHH3	0.353972615	0.765652652	1
STRIP2	-0.821456545	0.370440294	1
NDRG1	0.802637784	0.434356752	1
EI24	-0.212990063	0.836787544	1
KLHL8	-0.126773862	0.897998685	1
USE1	-0.145186683	0.888930331	1
JADE2	1.003742623	0.569120716	1
ACAP2	0.713047311	0.462924998	1
TSPAN4	-0.097889009	0.925947478	1
FGD6	0.370904485	0.811417569	1
PKD2L1	-0.706650932	0.463358697	1
ASH2L	0.265113574	0.814342408	1
DROSHA	0.962005582	0.468665583	1
ST7	1.523788155	0.257920865	1
G5654	-0.609157667	0.575970472	1
SMAP1	-0.030953372	0.974531491	1
PHRF1	0.644866291	0.807580478	1
CASZ1	-2.527042259	0.286049226	1
COLGALT1	-0.30306242	0.754854161	1
DOK5	-1.050182248	0.257734283	1
MADCAM1	0.611527021	0.616958472	1
PAM16	-0.855969783	0.412798113	1
COG2	0.776299168	0.525037164	1
SLC9A8	0.43499846	0.799304678	1
SLC13A1	0	1	1
ARAF	-0.739904132	0.63011579	1
AFF4	0.785256652	0.460187277	1
KLF4	-0.927983832	0.346508178	1
MAPRE1	-0.146343933	0.875355204	1
PPP2R5D	-0.022969706	0.979482611	1
STIJA	-0.831916507	0.392135242	1
PRKCI	1.419137541	0.171565489	1
CFH	-0.353752836	0.677960133	1

YTHDC1	0.886124931	0.470256537	1
CHRNE	-0.266869615	0.751563672	1
PPP2R1B	0.695593393	0.531333988	1
CD248	0.023021653	0.983989141	1
CCN4	-2.849313825	0.148181843	1
HERC4	0.552743285	0.649151985	1
NCLN	0.672421945	0.450629172	1
B4/PCDHGA8/PCDHGC3/PC	1.092768986	0.421921604	1
CIAO3	-0.187955264	0.849925905	1
TNRC6A	-0.460028361	0.590332437	1
CELSR2	-0.228667163	0.785428658	1
WDR41	-0.047552407	0.963368297	1
ASB15	-0.62031977	0.550530015	1
CD74	0.563858411	0.565848668	1
ACP2	1.241836254	0.204710081	1
CCDC186	0.147705569	0.89859303	1
RBFOX1	-0.728451251	0.615831608	1
SNX25	1.60296568	0.186290643	1
FOXJ2	-0.290678258	0.795899011	1
MYOC	-0.88373575	0.588356815	1
TSPYL1	0.003716769	0.997046553	1
FAM193A	-0.136523385	0.934302498	1
TP53RK	0.871965773	0.426543387	1
SLC4A7	0.959792226	0.486449595	1
COQ4	-0.196947708	0.878284295	1
C29H11ORF68	0.28728802	0.830888419	1
BCS1L	-0.509718769	0.584544727	1
AKAP10	0.483362379	0.750074096	1
LPGAT1	0.763960186	0.504025318	1
PHC2	-0.505600751	0.737118863	1
USF1	-0.512040454	0.632963266	1
ALKBH3	-0.901097658	0.315979891	1
ZFPL1	0.827316137	0.402888112	1
CSK	-0.070199699	0.942034084	1
DDX49	-0.095966593	0.921563572	1
FAM189A2	-0.395153846	0.713900099	1
ZNF511	1.371019501	0.225825174	1
RASA3	-0.173540643	0.860338917	1
CEL	-0.301654591	0.740217147	1
LRATD1	1.965304261	0.086243663	1
JMJD1C	-0.341554976	0.744874439	1
VAMP8	2.162336014	0.107624306	1
KLC4	0.543054661	0.653323876	1
ESD	0.722636228	0.54085535	1
TTC7B	0.195124033	0.907322047	1
COPG2	0.962549004	0.459014776	1
UBE2K	0.079417981	0.934230802	1
CORO1C	0.032197044	0.965234156	1
SSBP3	0.594522596	0.534394922	1
PDGFA	1.557599539	0.322952662	1
RPL7	1.718271769	0.143760248	1
TFDP1	-0.209114221	0.839444268	1
HIPK2	-0.054785629	0.972825982	1
FLOT2	0.728506875	0.475317291	1
PDLIM5	1.249113868	0.290426648	1
TM4SF1	-0.129729454	0.891334197	1
PPAN	0.31549593	0.75981441	1
ARHGEF2	-0.680134402	0.427619145	1

RRP36	-0.009054156	0.990898445	1
DHX38	0.737425935	0.549155553	1
CGNL1	0.80937052	0.412892488	1
LASP1	0.295424702	0.774569071	1
DHX16	-0.441121067	0.700364785	1
ZMYND8	1.053749205	0.405828401	1
RNF220	-0.269536961	0.824572669	1
AREL1	0.251181729	0.816879291	1
HEATR5B	1.717966183	0.331984144	1
ZNHIT1	0.564198697	0.62323823	1
PYROXD1	0.113629583	0.944985755	1
NPR2	0.106049567	0.907757103	1
OLFM1	-0.677717799	0.622480497	1
ST7L	0.421989038	0.741492641	1
STON2	0.580096059	0.685196684	1
NFATC3	-0.642436091	0.689248847	1
NFKBIB	-0.266396975	0.816732471	1
LOC532875	1.037009459	0.364557824	1
R3HDM4	0.216311978	0.851433129	1
CHMP1B	-0.155808241	0.855900875	1
MRPS31	-0.826034412	0.396857707	1
RSL24D1	0.189825939	0.863163842	1
MRPL54	1.271611592	0.27972578	1
MACROH2A1	1.098156173	0.286714484	1
CARM1	0.482472858	0.793628871	1
TTLL3	0.975659231	0.364720403	1
AHNAK	-0.531159416	0.667136398	1
LSM8	-0.773737812	0.411793715	1
ZNF687	0.187101948	0.854642273	1
OGFR	1.869714603	0.177646536	1
CHRM3	2.798728617	0.15811968	1
DENND10	-0.059853142	0.952452309	1
ERCC6	-0.251008376	0.800073761	1
MON2	0.925380761	0.500166606	1
ELOVL1	1.173993668	0.396784356	1
PSMC6	-0.394061873	0.670747534	1
ABHD12	0.589701484	0.498504037	1
HMG20B	0.480231826	0.586796208	1
USP15	0.222375405	0.85099519	1
CCDC107	1.4073125	0.212811142	1
ERAP1	1.613902239	0.208501948	1
SRBD1	1.939444682	0.189754852	1
HDAC11	-0.071492306	0.956605364	1
NCBP3	1.075453916	0.413496354	1
TIMM13	-0.59922224	0.442053904	1
BACE2	1.346293749	0.225227874	1
ITGA7	-0.030068956	0.979475746	1
MLIP	-0.496331808	0.777261113	1
NFKB2	-0.471117182	0.661605452	1
ARFRP1	-0.277137461	0.753725781	1
ARHGAP31	-0.247538932	0.806636242	1
RNF150	-0.463749839	0.789378065	1
SRA1	1.18883251	0.376573853	1
PGS1	-0.13883399	0.891252593	1
NOP10	-0.025823608	0.979833657	1
MED29	0.130619713	0.904931298	1
PPRC1	0.46072762	0.63551589	1
NGLY1	-0.282003603	0.800495673	1

PTEN	0.499952518	0.665825978	1
HSPA13	1.376052412	0.391401383	1
HMGA1	0.439539819	0.700868765	1
RAP1A	-0.297659446	0.849635042	1
ESS2	-0.185197511	0.870447283	1
SAFB	0.47696507	0.635910416	1
ITSN1	-0.624760514	0.462422702	1
DDX56	-0.058661089	0.948435902	1
ATG9B	1.788992048	0.087957546	1
AHNAK2	1.950142542	0.142700733	1
PEX14	8.67E-05	0.999929999	1
DMKN	0	1	1
RAP1GDS1	0.517220182	0.610472095	1
TSPAN17	-0.521531118	0.534497565	1
LCMT1	-0.372136796	0.661187558	1
MRPS28	0.151256631	0.887072476	1
NFKBIL1	-0.504722092	0.643781647	1
VPS13A	0.517409646	0.663647457	1
CELF6	0	1	1
RBM22	-0.311072987	0.769048164	1
PPHLN1	-0.312368582	0.736667474	1
CUEDC2	-0.387879561	0.689984405	1
TGFBR2	0.05692121	0.957384291	1
S100A5	0	1	1
FGGY	0.212476753	0.822285791	1
KDSR	0.52106572	0.552078213	1
MADD	-0.146198805	0.897906798	1
ENSMMSG00000013001	1.053462994	0.378851617	1
JADE2	0.08249423	0.950043523	1
QDPR	0.806125095	0.530376829	1
PPME1	0.270328542	0.792028632	1
APBB2	-1.078588709	0.332239954	1
ITPKB	-0.209363618	0.838981786	1
ACYP2	-0.993455616	0.528343783	1
NUAK2	1.714484116	0.260551978	1
NIT2	-0.372031948	0.604220438	1
CEP290	0.980054289	0.430099999	1
UMPS	-0.034898864	0.971262632	1
SYNPR	0	1	1
SAP30BP	-0.082466184	0.916267755	1
JCAD	-0.751195345	0.463469599	1
ENSMMSG00000012008	-0.161249656	0.94073304	1
CERK	1.359083528	0.196675984	1
RAC1	1.521917023	0.20984699	1
SYNC	0.070058218	0.959078475	1
RBM15B	0.611098394	0.551755286	1
TRMT1	0.382026302	0.835349813	1
SDCBP	-0.8060418	0.425505666	1
ABHD5	0.747786245	0.451968274	1
KAT2A	0.317868874	0.83974104	1
COMT	0.381611143	0.818379216	1
ZBTB47	0.268188285	0.843148202	1
ATAD3A	-0.266840172	0.764171301	1
ENSMMSG00000002469	-0.788500674	0.382141683	1
SMC3	0.914346791	0.460691722	1
TDRD3	0.673081594	0.586817677	1
TYK2	0.275338066	0.786221037	1
LIG1	1.46153482	0.197689907	1

SCYL2	0.415048025	0.80279984	1
KDM1B	-0.429683624	0.782161321	1
MUL1	-0.811307138	0.425776938	1
AGA	1.113117462	0.298073251	1
GRB10	-0.2372285	0.844330678	1
TET3	0.854038065	0.533070914	1
MACROH2A1	-0.557714115	0.715511394	1
LRRFIP1	0.280305678	0.869741776	1
APPL2	-0.420288768	0.679514726	1
UBA5	-0.051482126	0.956092163	1
NFATC1	-0.804507248	0.507814051	1
ARFGAP1	0.047997104	0.965271893	1
ERI3	-0.604811635	0.417658174	1
RNF187	-0.128589267	0.904953599	1
NAV1	-0.42565261	0.593200655	1
LDLR	1.570063135	0.209438059	1
TXNL4A	-0.145036913	0.886298959	1
CHRNA10	-0.977466215	0.51350484	1
CEP95	0.235896436	0.759552357	1
NAA35	-0.046950072	0.96661212	1
BSCL2	0.444379686	0.671997483	1
TMED7	0.807710088	0.608060458	1
ATF7IP	0.680034559	0.516375068	1
SGK1	-1.08759335	0.491316154	1
AGFG2	1.904069742	0.179779783	1
CTBP2	-0.624244888	0.559444577	1
MID1IP1	0.138935041	0.9204454	1
MMP14	-0.164120979	0.863010161	1
LTV1	0.210688971	0.845226809	1
DTX2	0.656002115	0.61436496	1
TNPO1	-0.05704914	0.970644252	1
SH3GL1	0.827478452	0.444796234	1
HAX1	-0.519660371	0.475937739	1
PLXNA1	0.312546362	0.756896737	1
TSC2	0.704739139	0.733687451	1
FBXW2	0.320495542	0.692684391	1
MYADM	-0.675395963	0.480606283	1
TADA3	0.057814746	0.950585339	1
SACM1L	0.475172899	0.653249011	1
PPFIA1	1.195539023	0.345759576	1
RAI1	-0.197582313	0.824055468	1
PRPF4	0.065348383	0.967927295	1
TBRG1	-0.351807153	0.707063624	1
FANCD2	-0.359758168	0.72436392	1
RALGAPA1	0.050842806	0.981130637	1
PSMG1	0.491167269	0.540306263	1
SLC66A2	2.004013511	0.380086622	1
TRAK1	-0.338203916	0.825456372	1
SEPTIN8	0.679803329	0.44233823	1
CES2	0.739039952	0.708252177	1
ACP1	-0.271663536	0.751063271	1
TSC22D2	-0.387794422	0.740083269	1
ABCC10	0.868074687	0.469869171	1
GCC2	1.923912705	0.139374731	1
POLR2A	0.004383381	0.99688562	1
PNKD	-0.252196417	0.827147102	1
CELF1	-0.506145961	0.757730132	1
NOL10	0.093476898	0.952597876	1

RCN2	0.500996431	0.687257074	1
SFMBT1	0.925361388	0.432839039	1
MPDU1	0.968467011	0.269316331	1
TEN1	0.531204822	0.631261506	1
FBXO42	-0.320987997	0.754327909	1
PANK1	1.448180756	0.288650666	1
TMOD1	-0.826784315	0.496493849	1
PDK1	1.158004854	0.342634902	1
ACOT2	0	1	1
CD36	-0.850094938	0.585298801	1
ZBTB4	-0.264916969	0.823228941	1
SEPTIN4	1.568537703	0.209696654	1
CPNE3	0.714755168	0.554963174	1
MYO9B	5.112882758	0.220124498	1
KDM3A	-0.471421137	0.667816406	1
EMC6	-0.362160197	0.71410018	1
GPKOW	-0.358525461	0.703655689	1
HEG1	-0.420929644	0.612763719	1
SMC5	0.832094781	0.636740146	1
PRMT2	0.143651449	0.906254329	1
GPATCH1	0.395381511	0.697719601	1
RBBP5	-0.076133132	0.943957554	1
TNRC6B	-0.378348546	0.667602363	1
OPA1	-0.781425919	0.625473067	1
LOC509506	0	1	1
HMGCL	0.356264643	0.728006104	1
SPCS2	0.671948005	0.48738392	1
RNF123	-0.330775101	0.74580754	1
DYNC2I1	1.274136108	0.272841372	1
USP12	0.28838026	0.780490376	1
GNA12	0.811525169	0.549709051	1
TUBGCP4	0.079276033	0.944928593	1
RHBDD3	1.721610034	0.132880403	1
OFD1	1.529667585	0.269521567	1
HOOK3	0.09460132	0.907144145	1
GTF2B	-0.296358976	0.704019305	1
PEX26	-0.213783999	0.856414671	1
SLC11A2	1.072969852	0.455559626	1
MTIF3	0.404572181	0.705944896	1
TUT4	-0.696747575	0.458953304	1
ING4	0.736520638	0.676161925	1
MOB4	0.221508579	0.804491289	1
DNMT3A	-0.423046213	0.618473865	1
NDUFC1	-0.759520451	0.453087281	1
UBE2J2	-0.228722107	0.799113792	1
C7H1ORF35	0.905207062	0.379540326	1
ATXN2L	0.437543152	0.842521242	1
DEF8	0.381116928	0.7552756	1
KLHL34	0.094287439	0.949971927	1
FAM89B	-0.135824829	0.896799012	1
BPNT1	0.546458578	0.594981092	1
BFAR	0.454036922	0.711701041	1
SIX1	-0.366617597	0.766520288	1
SUMF1	0.786071797	0.604316211	1
ZNF24	-0.15718256	0.874479607	1
CCNL1	-0.257441458	0.786846087	1
EIF2B4	-0.365004069	0.811845993	1
SLC17A5	2.411072697	0.147439666	1

KAT7	1.45E-05	0.999992856	1
GRAMD4	0.411952005	0.700772184	1
TRIM11	0.910308555	0.383617909	1
SALSA	0.216430285	0.824482706	1
FAN1	-0.160183495	0.871424545	1
CPSF6	0.313187407	0.854297494	1
ZC3H3	-0.129567089	0.909314517	1
POLR1E	0.364327215	0.764781952	1
NUP85	-0.229364019	0.801783523	1
HIKESHI	-0.714527116	0.414342359	1
GOLGA5	0.71045509	0.522946405	1
VPS13D	-0.166612443	0.86060695	1
PHKG2	1.019708751	0.286192301	1
AASS	-0.822166888	0.227470556	1
DDX47	0.07552758	0.930797549	1
ZFP36L2	0.244696304	0.824586993	1
CAV3	-0.14129576	0.921440884	1
SNAP29	0.54990941	0.598287585	1
FANCE	-0.319121261	0.699524276	1
KRT1	-1.415632864	0.726294536	1
SLC7A2	0.839982234	0.571841	1
SEMA3F	1.339678863	0.159565177	1
FAM193A	-0.128138482	0.898650931	1
LIMCH1	1.319268973	0.436918121	1
LAMA2	-0.359292464	0.722161018	1
AGO1	-0.197320132	0.841821981	1
PDLIM5	1.493466515	0.444338298	1
SHMT2	-0.468547352	0.548102143	1
MRPS18B	-0.155181978	0.865214018	1
BCAR1	-0.29414983	0.778798567	1
WDR91	0.984264292	0.461553983	1
VPS39	-0.181398917	0.829153717	1
TFB2M	-0.679009911	0.665811202	1
KDM5A	-0.152056561	0.880893637	1
ANTXR1	-0.217062114	0.846312152	1
WASHC3	-0.425085904	0.595401282	1
PATZ1	1.397057895	0.317594945	1
GADL1	-1.21301647	0.394723559	1
PSME2	0.295393888	0.76609572	1
PAOX	0.540546167	0.592701067	1
KCMF1	-0.756504644	0.446805037	1
OVOL3	0	1	1
G37022	0.550960875	0.607859133	1
WNK1	-1.110661784	0.27647913	1
SETD1A	-0.204650836	0.856727467	1
ADSS2	0.473538711	0.709496734	1
ANG/RNASE4	0.122331286	0.910859777	1
NCOR2	0.136344448	0.937694047	1
MRPS22	-0.405127166	0.695262927	1
DAD1	-0.217295365	0.80455149	1
GTPBP1	-0.173910344	0.871528366	1
CHPF2	1.674012259	0.190626192	1
LOC112446383	-0.275366269	0.763062645	1
PPIP5K2	3.0677021	0.229386784	1
KPNA2	0.164165044	0.862408417	1
IL6ST	0.12118141	0.903449513	1
PAX3	0	1	1
CNTRL	1.068637897	0.324004823	1

SCARB2	0.008383979	0.995901874	1
LZTS3	-0.418202028	0.73242665	1
SMAD1	-0.618686609	0.54777949	1
FBXW7	0.331837997	0.787042038	1
EVL	1.197026938	0.33905693	1
RNF121	0.124060468	0.890153779	1
CCDC85B	1.712179837	0.132773346	1
FHAD1	-1.879169201	0.406473272	1
TYSND1	0.693065023	0.552761277	1
RSU1	-0.436254037	0.783601881	1
TNKS	-0.342916621	0.735022725	1
FAHD2	-0.400778082	0.708047278	1
MARCHF7	0.154882017	0.892139375	1
NTPCR	-0.53401904	0.57053661	1
PIP5K1A	-0.030979418	0.977501271	1
MTG1	-0.077827906	0.938777588	1
KRIT1	0.828658477	0.528400342	1
FUBP3	0.054036869	0.959910208	1
DDX18	0.428552732	0.695460784	1
TRIM50	0.112394168	0.901562155	1
PUF60	-0.511054654	0.757232929	1
MICALL1	-0.303387609	0.819899835	1
SPR	0.276315578	0.781316725	1
NOS1	-1.987775746	0.621570392	1
SHARPIN	0.822947425	0.446752158	1
DHX40	-0.19690796	0.863904024	1
RNF170	-0.148467432	0.895263442	1
STAT2	0.539873713	0.729147223	1
BOLA2B	1.838012943	0.108739812	1
CHMP7	-0.461153833	0.627824281	1
FNIP2	0.572320282	0.681034519	1
ASCC1	-0.287124081	0.865532945	1
SRPRB	-0.005766293	0.99482376	1
PUM3	1.436470243	0.285018321	1
NUP107	0.338737736	0.775845089	1
LOC521224	0.299573838	0.821016619	1
PLK2	-0.661001022	0.509275865	1
VTA1	-0.337438218	0.753168471	1
DDB2	0.977096995	0.3860504	1
SERPINB1	0.953788703	0.274325836	1
PRKD3	0.508149612	0.559916913	1
UBE2F	-0.14369427	0.884760325	1
GTF2H2	0.446530474	0.648719356	1
ARL2	-0.598222988	0.53412507	1
MYO1E	-0.348537718	0.718543545	1
EEPD1	-0.528242788	0.689545814	1
CHURC1	-0.297921843	0.699910575	1
ZFYVE16	0.444774904	0.712541538	1
ARIH1	-0.718997328	0.451406491	1
CYTH1	0.803996565	0.649718834	1
ANO5	-0.885974369	0.578530673	1
GABPA	0.106700705	0.899788221	1
GNA11	-0.250679388	0.808608899	1
PNPO	0.928370336	0.402409459	1
POLR2M	-0.389131037	0.718877356	1
ASB8	-0.622483659	0.620274681	1
ARID5B	0.782584948	0.572830487	1
DNAJB14	1.030271722	0.525291081	1

DBN1/PDLIM7	-0.502001592	0.506951837	1
CDK5RAP1	-0.728421792	0.462129367	1
CCDC85C	-1.82486223	0.441439353	1
TRA2A	-0.0456276	0.962962744	1
TCN1	0	1	1
AP5Z1	0.491662008	0.709613451	1
CALU	-0.07706291	0.944721824	1
ENSMMSG00000022738	1.446205589	0.136113254	1
ENDOG	-0.29848363	0.801186464	1
ROBO4	-0.659681326	0.498628285	1
NOL8	0.444287134	0.699872538	1
ENSMMSG00000009950	-0.319305562	0.723389729	1
ZADH2	-0.189316422	0.86488499	1
C7	-0.409292026	0.80290739	1
PSIP1	-0.164154897	0.920307398	1
PDXK	0.126834891	0.899669064	1
PPM1K	1.850331404	0.304598149	1
PIK3C3	0.288111475	0.773483164	1
FOXRED1	-0.153386085	0.877069318	1
LIG3	0.843841564	0.465259665	1
HNRNPK	1.965567676	0.442981229	1
SUPT20H	0.658278549	0.610340006	1
MAP3K4	0.683505104	0.609693026	1
NOB1	0.233973895	0.804078075	1
RSRC2	-0.231500198	0.812180022	1
RPL10	0.893579022	0.48663186	1
PEX7	0.545236453	0.56659773	1
KMT2E	-0.071441295	0.944259999	1
LAMTOR1	-0.384675535	0.713522448	1
INTS12	-0.310037748	0.762804283	1
S100A16	0.118317782	0.891913452	1
POGZ	-0.474924397	0.757764253	1
TSC2	0.459068696	0.750887848	1
ARAP1	0.242879453	0.843261444	1
FTO	0.954520681	0.447570643	1
HIPK2	-0.1719048	0.856573171	1
WHAMMP3	-0.405457945	0.646583325	1
METTL23	-0.571408915	0.561694025	1
PRNP	-0.407688638	0.702329267	1
BDH2	0.713109536	0.505470545	1
TSEN34	0.899734046	0.421526349	1
WDR36	-0.11287205	0.918521215	1
CREB3	0.017197382	0.986144645	1
FAM32A	0.016976623	0.986341694	1
COG5	-0.033003566	0.972726258	1
ATIC	0.896124977	0.483400193	1
AGAP1	-0.386192505	0.740008378	1
GKAP1	-0.893292169	0.325028804	1
ITGA6	0.307948213	0.843906733	1
AMD1	0.714030338	0.688886346	1
FNDC3A	-0.074566061	0.943991963	1
BMP2K	-0.18906679	0.848345385	1
SORBS1	0.109554303	0.946835484	1
ATL3	1.033671185	0.250658516	1
NEO1	0.148465555	0.883215817	1
ANAPC16	-0.189895824	0.909930412	1
SLX1A	1.553534635	0.185780664	1
TMEM33	0.110888778	0.917469551	1

RAMP2	-0.407745586	0.667261102	1
DBP	1.613400442	0.19238605	1
TRIM41	0.655519963	0.605993675	1
HMGN2	0.634480346	0.608897855	1
MRTFB	-0.037697571	0.971422993	1
EML1	-0.853755238	0.422268633	1
VASP	-0.388240123	0.702609397	1
YTHDF2	-0.087950365	0.9553882	1
BUB1	2.905732893	0.113250062	1
DAP3	-0.352383964	0.664856426	1
POLRMT	-0.1678801	0.875599556	1
RPF1	0.80043144	0.498116583	1
SPRING1	-0.076425848	0.94639587	1
RBM23	-0.004884022	0.996599154	1
PPP2R3B	-0.910210044	0.383347612	1
AMY2B	-0.362118321	0.784964626	1
MAGI1	0.353061435	0.732401796	1
DELE1	-0.197148787	0.868618255	1
NIT1	0.082794732	0.938327038	1
GAB1	0.135506457	0.922564952	1
OPA1	0.475814943	0.697551842	1
NMD3	1.053561443	0.404651328	1
PBRM1	1.460720702	0.526699982	1
MOB2	-0.161374336	0.880987783	1
SRPK1	0.419682688	0.714338694	1
NAT10	0.028239726	0.980928383	1
PAN2	1.572895633	0.54426211	1
PNPLA4	1.288315905	0.32067823	1
LDB1	-0.59152395	0.538518051	1
CEBPD	0.906011655	0.421756078	1
ELN	0.175463193	0.866395284	1
PRRC2B	0.979464167	0.318477016	1
FOXO4	-0.743041304	0.657262862	1
DIP2A	0.223410226	0.842502396	1
JAGN1	-0.107373701	0.920159068	1
TRIM16	0.670082138	0.531598852	1
RNF5	-0.685191558	0.482993565	1
THOC1	0.770275356	0.434140317	1
JADE1	-0.222509178	0.850149641	1
TAX1BP3	0.191767619	0.823061337	1
HPS1	1.667047228	0.170425464	1
PET100	0.840344536	0.485548822	1
ABR	-0.193851923	0.848958066	1
GTF2E2	-0.039946852	0.966393309	1
NCOR1	0.96461492	0.59844885	1
SEMA4C	-0.255578544	0.798617721	1
LRRC40	-0.056876114	0.97172405	1
MTRR	0.182751371	0.891582725	1
NCOR2	0.897477056	0.428321194	1
NCOA2	0.865517243	0.390013456	1
COMMD7	0.152814956	0.888179526	1
DDA1	-0.294533543	0.760512933	1
CPSF7	0.415423145	0.798680799	1
LGALSL	0.245260345	0.851697552	1
H2AZ2	-0.077003809	0.939361804	1
C5H12ORF57	0.869208662	0.411436784	1
ENSMMSG00000008145	0.149338131	0.924636374	1
ANKRD27	0.752651424	0.57962666	1

DPYSL3	0.599238135	0.749007939	1
CTNNBL1	-0.248158842	0.759795746	1
FSTL1	-0.475601613	0.634266606	1
ENSBTAG00000037552	1.135236079	0.491000106	1
PAK2	0.93947901	0.466494546	1
ERRFI1	-0.327858745	0.75048632	1
MED14	0.672131203	0.600107782	1
TBC1D20	0.244464746	0.795447627	1
SNRNP40	0.058685301	0.959821939	1
SPICE1	1.99376245	0.136670192	1
EHMT1	-0.259423391	0.870537572	1
G3130	1.047778709	0.422327483	1
UBE2H	0.7244228	0.508273893	1
OSBPL9	0.461236167	0.644585854	1
AEBP2	-0.023644945	0.988453455	1
TBCC	-0.01956949	0.987256249	1
NME4	0.581297844	0.689833173	1
BTRC	0.373652706	0.765001254	1
PTK2	-0.56267139	0.708603432	1
DXO	0.577119586	0.596582283	1
DNAJB12	-0.185082139	0.847293272	1
DAB2IP	-0.675312976	0.667736136	1
ARMC10	0.928016859	0.295223735	1
TANC2	1.297303646	0.268537433	1
KIDINS220	-0.624910259	0.721013238	1
UBR5	-0.904345519	0.584945045	1
PRPF39	0.860068348	0.475789985	1
CPNE1	0.53931823	0.6219359	1
GUCD1	0.151652085	0.92212959	1
PTPN13	1.145223613	0.470667719	1
AKAP6	0.070786923	0.968049352	1
HDAC7	0.01179692	0.989915679	1
LONRF3	0.852579964	0.473705553	1
EPS15L1	-0.597873032	0.50514176	1
FAM214A	0.390136294	0.74053932	1
PPP1R3B	-0.333847158	0.83227435	1
PAIP2	-0.514215661	0.563313693	1
MAP3K11	-0.03811804	0.969838624	1
SEC31A	-0.592453002	0.550387544	1
PWP2	0.05187388	0.955492673	1
ZNF622	0.03171847	0.973694823	1
NCBP2	0.412110911	0.628974943	1
LSM7	0.458277446	0.688934042	1
ARPP21	-0.73747726	0.525866801	1
KRI1	-0.041985309	0.962295848	1
G6PD	1.478819138	0.169263569	1
MLLT11	0.235798745	0.863847985	1
DYNLT3	1.263820671	0.441562452	1
PEX1	1.354368905	0.29704336	1
MMAA	0.310595157	0.751078846	1
TEAD3	0.824572052	0.547746075	1
DKK1	2.931410026	0.187116054	1
ITGA6	-0.288446833	0.785655573	1
PTBP3	1.102800366	0.227098081	1
IFT172	0.526959909	0.577728839	1
LOC509283	0.753228149	0.570354189	1
FCSK	1.672456765	0.22615615	1
GOLGA1	-0.138255101	0.886383541	1

SCMH1	0.046136489	0.977786788	1
MCAM	-0.799876796	0.407938384	1
WBP1	0.635321978	0.47141082	1
PALS1	0.096494845	0.951743498	1
SON	1.979906912	0.126648188	1
WDR7	2.931912692	0.228281261	1
ZNF786	0.411961427	0.725930846	1
SRP72	0.200080091	0.84338646	1
CD99	-0.088379009	0.92973838	1
NSMAF	-0.611790511	0.543390064	1
LRRK2	-0.193448322	0.843315431	1
ARPC4	0.643192009	0.373085981	1
MRPL43	-0.843214855	0.35109116	1
GNAI3	1.082155763	0.245184387	1
SAFB	1.095455398	0.346867584	1
CTNNAL1	-0.008894524	0.995808268	1
PLEKHG3	-0.341926525	0.704491086	1
FURIN	-0.348050526	0.759739356	1
MRPS27	-0.384869014	0.696151062	1
MAPT	0.682893843	0.678729645	1
GPD2	-0.961416425	0.44673852	1
DPM3	0.560799313	0.56557781	1
PTPRJ	1.671580304	0.301412064	1
STRN	1.79300631	0.178615669	1
U2AF2	0.098080453	0.952576509	1
KCNJ14	0.312601201	0.74668734	1
RCSD1	-0.924478127	0.559019768	1
PCCA	-0.9276599	0.291025876	1
MRPL34	-0.064539206	0.952603251	1
BRD9	-0.476889606	0.655114597	1
SHANK3	-0.168429624	0.876776795	1
TMEM54	3.40766879	0.120096151	1
SALL2	0.029269284	0.97750991	1
EPB41L2	-0.677358488	0.654198675	1
NUP93	0.020945393	0.980152222	1
SESN1	-0.81075967	0.576951098	1
EFNA1	0.294796783	0.794188426	1
PRPS1	-0.441097677	0.644210426	1
ENPP4	1.068486548	0.484145915	1
RBM23	0.146824178	0.885852038	1
CES4A	1.344614969	0.125966778	1
GOLPH3	-0.181161249	0.845557419	1
AS3MT	1.324082372	0.269205598	1
USP11	1.367045533	0.282070353	1
SLAIN2	0.448555345	0.804077503	1
GAPVD1	1.303800763	0.460002351	1
ADD1	-0.326123547	0.813037473	1
RECQL5	1.037777091	0.394872696	1
NFX1	0.36909187	0.662637303	1
GSK3B	0.975332213	0.419111287	1
G19895	-0.060023164	0.956219905	1
CERT1	-1.120070043	0.286267499	1
BABAM1	-0.377599683	0.70253609	1
KANSL1	-0.03290882	0.978546279	1
NDFIP2	-0.656232336	0.678500499	1
SEC31A	1.498493414	0.39588437	1
TARBP2	1.770452223	0.147069921	1
CARNMT1	-0.758200905	0.456823235	1

CAMK2D	-0.53240997	0.749220082	1
PPOX	0.386266959	0.702933721	1
SLC33A1	2.051671707	0.115188824	1
BUD31	0.850890255	0.417823244	1
DHTKD1	-0.553197735	0.744416597	1
MPHOSPH8	0.058053508	0.956821543	1
ADAR	0.587217608	0.727699009	1
ARHGEF40	-0.123866018	0.895529502	1
OLFML3	0.043308959	0.962185728	1
MTR	1.444484077	0.429198529	1
TAOK3	1.349806699	0.306307992	1
KIZ	-0.385722726	0.699710287	1
RNF43	1.511108232	0.317881919	1
TPM3	0.971408567	0.499997851	1
MCM4	0.262682116	0.777237062	1
RAB5IF	-0.493107166	0.484442429	1
PDP1	-0.778520246	0.614460059	1
HARS1	0.378627342	0.670170665	1
KLF2	-0.954884435	0.301314741	1
HAUS5	0.380421623	0.67207192	1
BCL7C	2.33806491	0.093433724	1
SCLY	0.079649496	0.939513846	1
ULK3	0.37484695	0.691999866	1
MAPK3	-0.579543562	0.506168895	1
PAIP2B	-0.654657991	0.54133616	1
PBXIP1	0.243084749	0.834058106	1
TMEM129	0.422516044	0.689400902	1
BANF1	0.319627492	0.728141324	1
CCIN	0	1	1
FAM78A	-1.148006267	0.271485224	1
PPP1R12B	0.149297338	0.932723184	1
SEC22B	0.581958066	0.711527435	1
ATM	2.007437793	0.104420559	1
SNX17	-0.185527588	0.861436622	1
DYRK1A	0.336930391	0.796384872	1
PLVAP	1.392952051	0.229444956	1
TSFM	-0.146308638	0.876343736	1
XPNPEP1	-0.367812527	0.660894461	1
STRN4	0.43897731	0.784055389	1
UBE2Z	0.419226452	0.701191166	1
DYRK1A	0.407255859	0.815910112	1
TFG	0.397022382	0.740491039	1
SNRPN	0.655372237	0.59988811	1
NCOA6	-0.14512148	0.874980154	1
SND1	0.556098635	0.720156978	1
PLP2	-0.212859301	0.818542935	1
ARL1	0.056672411	0.956849785	1
SFXN4	-0.759258696	0.410862546	1
NOTCH4	-0.635061289	0.444766018	1
HELZ	0.543308257	0.771020354	1
RPRD2	0.28483706	0.759117571	1
ASXL1	0.281510871	0.761448636	1
TOR1AIP1	-0.361835794	0.828558973	1
AGPS	0.399691635	0.804871854	1
TSPYL4	1.884310379	0.172417477	1
DUSP7	-0.571244825	0.571922704	1
EFCAB14	0.221090805	0.861704879	1
EIF4ENIF1	0.78827939	0.567897559	1

GIT2	0.059522294	0.950944938	1
ABHD11	0.350136335	0.723513028	1
WDR48	1.783445261	0.183231766	1
ME2	0.326344107	0.799694567	1
UBAP1	-0.396947918	0.641987821	1
TRIM8	-0.573284938	0.599824857	1
PM20D2	-0.882409406	0.590833254	1
APOD	-0.896201502	0.442705958	1
TNNT3	-1.224129646	0.434691774	1
GATB	0.52764899	0.699677669	1
SLC66A3	1.973733815	0.181294042	1
FAM50A	0.14725965	0.841972495	1
PTPRK	0.912788746	0.338548869	1
DUSP10	0.426258005	0.755016603	1
BRPF3	-0.463081901	0.672140827	1
ZRANB2	0.826560566	0.513736836	1
MFF	-0.468196813	0.640096966	1
CCDC96	-2.848759374	0.137821403	1
CCN2	-0.616182969	0.563930248	1
POFUT2	0.17503509	0.870298932	1
JTB	-0.258735355	0.794593296	1
CPSF3	0.231401471	0.844766255	1
STK10	0.756610408	0.497086445	1
MCRS1	0.830881286	0.546412002	1
LGALS8	0.330150164	0.71151843	1
USP39	-0.157438064	0.879962065	1
YY1	-0.498207792	0.74966949	1
STRIP1	0.128889178	0.913704341	1
TRAK2	-0.572320643	0.599550948	1
BCAS2	-0.118712231	0.895114816	1
MAP3K21	1.448975806	0.285966442	1
COQ6	0.057883057	0.951487669	1
WDR75	0.180192065	0.872416183	1
ATP8A1	-0.017092858	0.989829178	1
DGCR8	0.112333018	0.876777511	1
AZIN1	0.63195427	0.703483088	1
BREH1	-1.871735075	0.385495567	1
TGFBI	-0.493969975	0.515360503	1
ANKRD35	0.358103758	0.711186807	1
ST6GALNAC4	-0.600058173	0.577223046	1
TEDC2/CCNF	0.476670295	0.614412495	1
TCERG1	0.181791602	0.914956083	1
EPS8	-0.475440419	0.761329809	1
TMEM130	-1.828496337	0.412338803	1
DNAH2	1.334338773	0.217980588	1
SLC27A1	0.058997718	0.963801864	1
SKI	-0.351501632	0.745608536	1
IQCE	0.802914768	0.337691313	1
SMRP1	-0.947248197	0.571278015	1
TSSC4	2.175304402	0.13519845	1
APRT	0.866582534	0.425315129	1
HEATR5A	0.093920321	0.933429054	1
GPBP1	-0.414016658	0.799918253	1
OSTC	0.620504547	0.498181331	1
CREB3L2	0.786368412	0.487149491	1
CD34	-0.824096434	0.589163537	1
DNAJB11	1.00900745	0.337465969	1
VIPR1	2.127643436	0.2357126	1

AAMP	-0.172595808	0.861809837	1
ITCH	-0.018290907	0.991411014	1
MIB2	-0.646157643	0.606851782	1
VEZT	0.058596943	0.971172818	1
CCDC25	-0.130627115	0.877565535	1
DHX37	0.251774564	0.770869351	1
EED	-0.022797005	0.980728504	1
BTF3L4	0.605829293	0.580217655	1
SETD1B	0.144477433	0.885626778	1
NFE2L2	-0.715141343	0.35851593	1
PSMB2	-0.638987938	0.476861775	1
MEN1	0.487200055	0.670339367	1
CNPY2	0.765220467	0.416217228	1
AGTPBP1	-1.027754092	0.343862683	1
DENR	-0.337883212	0.746708336	1
HIGD2A	-0.060689767	0.953385905	1
RETREG3	0.529142337	0.626278344	1
DIP2C	0.330311093	0.799327229	1
MAPK6	-0.597134817	0.601854345	1
FECH	-0.321044658	0.762059416	1
YKT6	-0.004312906	0.995956514	1
CHRNB1/FGF11	-0.535840441	0.672064061	1
LAMTOR5	-0.378424126	0.664780173	1
DNAJB5	-0.327449605	0.848910819	1
MLLT1	0.160192976	0.887058655	1
VPS72	-0.360075949	0.690151611	1
AXIN1	-0.704864458	0.666553578	1
SRRM2	0.04639565	0.965301928	1
FBXW11	-0.441128255	0.684904094	1
PARL	0.251047347	0.823912687	1
TRIOBP	-0.215213904	0.828538138	1
ENSMMSG00000011485	0.61582373	0.604774528	1
AXIN1	-0.711904217	0.532693867	1
TIMM17B	-0.686943576	0.470208825	1
GPAT3	0.250755841	0.887406062	1
PIGQ	-0.009449379	0.991830513	1
CAMK2G	-0.661941973	0.673711216	1
MIA2	0.111112277	0.927202511	1
NOS1	-1.151653441	0.467450471	1
SARNP	0.198260577	0.860747679	1
TCEA1	0.102950482	0.913853786	1
BTAF1	-0.128912988	0.89487828	1
FITM1	-0.993459686	0.279040984	1
STX8	0.14256848	0.890306023	1
MTMR2	0.436363896	0.70381574	1
SAMD4B	-0.131404211	0.901966938	1
RIC8A	-0.167546411	0.8775596	1
ACE	-0.707440839	0.503644039	1
ANKFY1	0.163779645	0.859359144	1
GAPVD1	0.61295494	0.636402653	1
PUM1	0.666478955	0.641211997	1
RBM45	-0.189803907	0.853168273	1
ZNF646	0.178653145	0.878905218	1
RRN3	0.735819077	0.557557967	1
RNF115	0.750694561	0.592047619	1
DTNBP1	0.971348384	0.49968724	1
COL5A3	-0.432432026	0.656594218	1
RNF126	0.457096357	0.684564075	1

ELK1	-0.08124916	0.932410179	1
TRAF2	0.354873773	0.749996387	1
BACE1	-0.528732856	0.539882687	1
TMEM175	0.44431162	0.678832048	1
CARS1	0.008008381	0.993615664	1
ZRANB1	-0.876515453	0.364064634	1
CUX1	-0.539553144	0.697418789	1
RAPSN	-0.666426547	0.516709143	1
ITPR2	1.936242967	0.132489441	1
WDR46	1.973913726	0.129734812	1
TRIM7	-0.466925214	0.708615591	1
CDC73	-0.30763737	0.842814277	1
CSNK1D	-0.468853639	0.648024126	1
NTAN1	-0.375077945	0.709419195	1
ANKRD28	-0.595109615	0.447989457	1
TMEM132A	1.277827472	0.272277592	1
IRF2BP1	-0.589746809	0.556311384	1
KAT14	-0.587292237	0.530681529	1
LIMCH1	-0.886280346	0.538021296	1
ATG4B	-0.309208102	0.797111286	1
HDDC2	-0.29133094	0.758889399	1
PELO	-0.194491623	0.78865016	1
PFDN2	-0.275266332	0.763640117	1
ATP7A	0.927173811	0.451317685	1
HINT3	-0.11328989	0.911491574	1
NAGK	0.864280072	0.392117092	1
TBCA	0.573738734	0.550724514	1
CSNK1D	-0.584185177	0.546309589	1
ARL8B	-0.505607507	0.595953695	1
FZD7	-0.526338552	0.654473068	1
ELAVL1	-0.102444158	0.911693433	1
DOCK1	0.402028418	0.691255073	1
B3GAT3	-0.318773538	0.785158972	1
WDR7	-0.222421175	0.831834838	1
JARID2	0.256082944	0.80931532	1
OSTF1	0.251335304	0.782569512	1
MRPS10	-0.030075016	0.977368701	1
USP32	-0.555139761	0.760139326	1
ATG4D	-0.040165531	0.969785536	1
REX1BD	0.128328575	0.907026203	1
DDX19A	-0.159896	0.875729771	1
ERAL1	0.890822143	0.517900256	1
TTLL4	-0.186325316	0.854505626	1
BCL2L12	0.813181513	0.55590312	1
SP1	-0.177037707	0.869341792	1
OARD1	-0.371534794	0.680373813	1
CCZ1	-0.526430126	0.577239442	1
MTIF2	1.038171539	0.455605484	1
TFAP2B	3.214791555	0.181816634	1
IGHMBP2	-0.126338624	0.908548689	1
PEDS1	0.132859227	0.913072446	1
DAPK3	-0.71458081	0.409157949	1
F11R	0.326523043	0.772856156	1
PGAM1	0.153144133	0.890036308	1
PITHD1	-0.550394833	0.734655616	1
SLC22A17	1.483764698	0.138514971	1
SAR1A	-0.01431569	0.986844241	1
CTSC	0.974007631	0.543371342	1

VAV2	1.922751979	0.201427579	1
MRPL17	-0.401547855	0.671221755	1
ARHGAP27	1.068871358	0.235462726	1
SUPT20H	1.717593629	0.338700883	1
ENSMMSG00000004991	1.290207052	0.314349628	1
ATF1	-0.126754314	0.901521794	1
CSTF2T	0.683989953	0.672077155	1
NFKBIZ	0.521008467	0.624149906	1
YWHAQ	1.239704915	0.293529522	1
FKBP8	-0.393956574	0.695257335	1
TMEM14C	-0.443704593	0.659618111	1
JAG1	-0.057136543	0.972426021	1
CTDP1	0.221249943	0.852783293	1
QSOX2	0.302066581	0.804918471	1
RUFY1	0.304858726	0.74123775	1
JAG2	0.080047388	0.928097565	1
RAPGEF3	0.330132129	0.755049155	1
PEX16	0.603653726	0.489192388	1
FBRSL1	0.159306392	0.880726581	1
ZNF598	0.496702057	0.697374073	1
GAB1	0.99877215	0.635506543	1
SNRPB	1.186881962	0.346834956	1
SLC12A7	0.546520806	0.635347882	1
PNLDC1	-0.71916956	0.443105001	1
TIMM10	-0.342446949	0.733599064	1
P3H3	1.249301274	0.257687249	1
GORASP1	0.244787212	0.823608936	1
POLR2A	-0.081908996	0.932481787	1
D2HGDH	-0.638275148	0.545750909	1
PNPLA8	-0.801252891	0.419929326	1
VPS4B	0.337607383	0.739416265	1
PRKCA	-0.231329141	0.834417754	1
PUM1	-0.341503436	0.83214413	1
AZI2	-0.44926049	0.776523826	1
ANO10	0.935354365	0.371771809	1
WDR5	0.411426156	0.652207947	1
USP19	-0.310299911	0.748142573	1
FAM20C	-0.580922285	0.628784335	1
EIF2S1	-0.149781459	0.857572371	1
LOC509283	0.532804461	0.706078153	1
ABTB1	0.534649035	0.619598396	1
LOC617406	1.940937107	0.210530703	1
MTHFS	-0.512335027	0.650673559	1
MYH3	-0.369378605	0.773028242	1
CFDP1	0.469849598	0.591464223	1
DPM1	0.628480501	0.577944648	1
LOC101905242/ANAPC15	0.229627324	0.838792361	1
INF2	0.696459068	0.591312143	1
KMT2A	-0.197807518	0.844390001	1
SF3B6	0.078175312	0.942361742	1
DERA	0.590351043	0.622066612	1
RBM19	0.398709437	0.693944826	1
PREPL	1.109649486	0.536453028	1
FIBP	0.540038929	0.623666271	1
MFAP4	0.929898031	0.483943543	1
OTX1	3.899845571	0.092973936	1
ACTR3B	-0.966301711	0.28547994	1
PIH1D1	0.705459543	0.539555379	1

PTDSS1	-0.153459296	0.879587678	1
DYNLRB1	-0.099168378	0.924138136	1
ENGASE	1.122106909	0.381981786	1
EHMT1	0.50727758	0.606121561	1
PPFIA1	0.503545188	0.806988783	1
CSAD	0.598281128	0.528996895	1
PSPH	1.520399161	0.097250594	1
FYCO1	-0.691517143	0.495214409	1
BABAM2	-0.091834664	0.927299206	1
POLB	0.344792364	0.776313088	1
C3H1ORF43	-0.390908045	0.678206303	1
TMEM30A	0.077209512	0.942999303	1
PCMTD1	-0.730125479	0.545032322	1
ENSMMSG00000000219	1.013611876	0.490568828	1
LRRC47	0.538084545	0.681955809	1
NUCB2	0.889206111	0.366450935	1
BCAP29/DUS4L	-0.933912202	0.282437741	1
FAM162A	-0.688595649	0.465602501	1
ZPR1	0.383102242	0.723318926	1
JMJD6	-0.374137509	0.692636769	1
ARID1B	-0.37035509	0.88162446	1
REXO1	-0.191234352	0.858890102	1
STAG1	0.587856625	0.651481287	1
LRP6	-0.248509142	0.762949739	1
TMED10	0.451892437	0.694320925	1
CEP131	1.965103434	0.108644677	1
ENSMMSG00000006749	-0.134761059	0.903332055	1
TRAF6	-0.634562698	0.493017373	1
PBX2	-0.132535199	0.912115513	1
ARMH3	-0.581414709	0.53643077	1
SERPING1	-0.110998814	0.909194439	1
ING1	-0.140040157	0.890508239	1
GMPR2	0.569868855	0.52334822	1
IGDCC4	-0.166862192	0.928265788	1
FRYL	-0.459500327	0.624265509	1
PRKCQ	-0.242820596	0.847388732	1
RPS6KA2	0.271696147	0.826245874	1
PEA15	-0.578378466	0.557588849	1
ADPRS	0.116727418	0.916615175	1
ZBED1	-0.607967225	0.615341635	1
SEC23IP	0.903352707	0.473898221	1
TSPAN7	0.267934759	0.879961811	1
PHF21A	0.15359729	0.881253134	1
EDEM3	0.310566199	0.773213975	1
DKC1	-0.145798386	0.887161119	1
TRIP4	1.000038863	0.3877588	1
UBXN4	-0.053403337	0.960343448	1
UCHL5	0.305610211	0.856305986	1
LARGE1	0.221022762	0.879589651	1
RAI14	0.236181235	0.888266294	1
APLP1	1.644805284	0.243840981	1
CNOT11	0.185309453	0.848478499	1
NCOR1	-0.222419286	0.895188508	1
ZHX3	-0.617901302	0.570306308	1
IL1R1	0.697260353	0.415498719	1
VPS36	-0.581283024	0.582770219	1
WIZ	0.030453853	0.976410262	1
ELMO2	0.187395265	0.907252817	1

STK33	0	1	1
WDR11	0.973217735	0.565265893	1
TBX3	0.814917714	0.374921907	1
TTC1	-0.758102295	0.409308635	1
CTBP1	-0.435191925	0.714956374	1
IWS1	-0.646924132	0.666143615	1
ENSA	0.484929589	0.656475709	1
GTF2I	1.050566019	0.548154634	1
PARD3	-0.527219126	0.624169705	1
MRPS25	0.101174951	0.928347086	1
AIP	-0.6051776	0.575958394	1
BCL2L1	-0.047462321	0.951857647	1
IPO8	-0.126061648	0.898150493	1
PDCL3	-0.643599807	0.512969287	1
ZFC3H1	0.796333114	0.518704235	1
TPPP3	0.228595754	0.869097737	1
FUOM	1.34410792	0.227885938	1
DOHH	-0.300374404	0.788951743	1
PCNA	-0.227862605	0.810231588	1
ATG13	1.818514432	0.200682212	1
IRF3	0.517784855	0.691146629	1
ANKH	-0.74867514	0.483660711	1
DRG1	0.022919999	0.976693866	1
PPT1	0.15174875	0.885222474	1
ENSMMSG00000018081	-2.853523546	0.285129237	1
DONSON	-0.581789751	0.516070606	1
TMEM120B	-0.753714874	0.537998712	1
FAM13A	1.290180838	0.320516856	1
SNRPD1	1.614330124	0.19360995	1
PTPN1	-0.144776567	0.852004861	1
PWP1	0.166903092	0.87034887	1
PFKFB2	0.5050797	0.715562057	1
TEX261	-0.176710048	0.835537862	1
PITPNA	-0.463531672	0.598561695	1
ANKHD1	1.370400574	0.225607339	1
PI4KB	0.407173968	0.84885884	1
KRT80	2.935300462	0.18804778	1
DOCK7	0.24689815	0.783468681	1
CAP1	0.749881077	0.639706197	1
WDR83OS	0.006295787	0.99417904	1
NSF	0.510496873	0.528143847	1
FLCN	-0.658450122	0.59897974	1
PARP2	0.191449819	0.856792235	1
HIRA	-0.081568404	0.919890386	1
CLASP2	-0.975733	0.629085005	1
SENP2	-0.315310482	0.843840641	1
SNRPC	-0.618071745	0.478696884	1
TINAGL1	0.1185127	0.907547317	1
COG6	0.356820279	0.723296917	1
CCNY	-0.311148906	0.774598281	1
KLF15	0.714535624	0.688902363	1
NIBAN1	0.329520428	0.763050642	1
XRN1	0.310229552	0.79829743	1
FIP1L1	0.019784476	0.985562726	1
DST	0.800847585	0.657441814	1
DYRK2	-0.252833644	0.802556021	1
RABL6	0.557377967	0.574802311	1
WDR24	1.056184115	0.297402485	1

NAE1	0.169703606	0.876858183	1
ELAC2	-0.440885362	0.63168246	1
APIP	-0.619534086	0.497562811	1
PRDM2	-0.173466326	0.860787796	1
ISOC2	0.354790895	0.747192549	1
FAM53C	0.236674989	0.865041068	1
BAG5	0.047590602	0.956036838	1
TMEM50A	0.280145461	0.79787106	1
NR1D2	-0.585418729	0.60779745	1
RMDN1	-0.712061798	0.472026794	1
PIK3CB	0.539901135	0.724359988	1
LOC618733/TAP2	-0.338885455	0.785535583	1
SNAPC4	0.023615544	0.988749304	1
SNTB1	-1.155520866	0.386374911	1
REEP5	-0.519953469	0.565207228	1
FITM2	1.347573406	0.394598631	1
SAP130	0.478007724	0.692825726	1
HOMER1	-0.940367879	0.346016458	1
DCTN6	-0.28171728	0.791268702	1
SNRNP25	0.594162542	0.606334132	1
PPDPFL	0.141756534	0.933023572	1
RINT1	0.660264464	0.583563078	1
HIF1A	0.103674087	0.894046106	1
AP3M1	0.840797124	0.478453026	1
DPYSL2	0.650559586	0.634657261	1
PRMT7	-0.083381955	0.931176223	1
KLC2	-0.533459944	0.608610048	1
SWAP70	0.233578993	0.822154523	1
BRI3	-0.249518425	0.801370478	1
GTF2I	-0.251075288	0.875127018	1
B4GALT3	0.732981302	0.444770583	1
MAP1A	-0.046966731	0.991002389	1
MEGF9	2.144109931	0.117505276	1
ULK2	0.051240218	0.958818664	1
GAK	0.667328186	0.512107246	1
SIK3	0.086229491	0.931332097	1
POMT1	-0.16606636	0.867801092	1
WDR55	-0.347095463	0.730519126	1
APPL1	-0.401464399	0.700405927	1
TMED4	0.195627419	0.842867535	1
DUS3L	-0.050059575	0.962806039	1
GPAM	-0.251449732	0.879957821	1
GTF2IRD2	1.117746608	0.364321779	1
ZNF219	-0.199664779	0.854572916	1
FAM114A2	-0.833085849	0.283574905	1
CDH5	-0.833013718	0.576839871	1
PNPLA7	0.997250516	0.417441742	1
TBL1XR1	0.832155677	0.497108474	1
SNRPA	0.55874871	0.603838021	1
RBIS	0.271244128	0.81338941	1
NASP	1.189347884	0.339172582	1
ABCA1	0.763170663	0.569162448	1
DNAJC14	0.463987037	0.648238086	1
LAMTOR2	0.217637129	0.823153594	1
SLC25A25	-1.035930875	0.34219691	1
CLK2	0.321877227	0.737699016	1
KIAA1671	1.492173814	0.290146084	1
GARRE1	-0.625838245	0.518400593	1

OTUD4	-0.546640227	0.514303336	1
SMPDL3B	-0.111760303	0.916708459	1
PPA2	0.328827173	0.771544408	1
TADA1	-0.085094054	0.92968295	1
E4F1	0.350416965	0.782216584	1
RWDD1	-0.84488205	0.59209542	1
TMCO4	0.474507845	0.69705078	1
EMD	-0.272851236	0.777345914	1
SNX33	0.729834273	0.517926588	1
VPS50	0.297657599	0.800963326	1
TMF1	-0.007681701	0.994461982	1
PLEKHA6	0.030708298	0.994116982	1
NFKBIA	-0.196533977	0.849831887	1
RELA	-0.202518118	0.844278941	1
NDUFAF3	-0.257229129	0.78468659	1
PNCK	-1.829716799	0.403472173	1
EBNA1BP2	0.162002802	0.878363888	1
DIDO1	-0.147605664	0.871038109	1
TMOD1	-0.843474189	0.499121254	1
THAP4	-0.250793529	0.831108204	1
CBWD1	0.155492205	0.868009547	1
PPP1R14B	-0.782357264	0.451726705	1
SLC25A28	0.000831899	0.999430625	1
PTPRM	-0.578781405	0.725115071	1
GDE1	-0.111757622	0.917487517	1
PLPP1	0.605497703	0.643029411	1
ADCY2	-0.712427065	0.62748251	1
PLA2G16	-0.450777518	0.650246412	1
SH3BGR	-0.205411711	0.882869955	1
ARHGAP29	-0.427891477	0.654895127	1
RHPN2	1.934410509	0.185806836	1
SLC40A1	0.494865523	0.690700533	1
CSNK1E	-0.14425954	0.867850918	1
UHRF1BP1	-0.591124197	0.618105538	1
RCBTB2	-0.169078573	0.828044533	1
FABP9	3.657870748	0.409538354	1
ENSMMSG00000001933	-0.117370989	0.923763466	1
RPAP3	0.038677785	0.968480601	1
BMS1	0.183662976	0.876391191	1
MTMR6	-0.407682072	0.711938889	1
SOWAHB	0	1	1
RIPK1	0.155042101	0.880766485	1
GLB1	0.193507017	0.830362174	1
PAFAH2	1.36337439	0.266823782	1
PBRM1	2.908709842	0.485370805	1
BRD9	0.084839735	0.925477959	1
CYTH3	-0.861666083	0.487426838	1
ZC3H10	0.401660347	0.703276149	1
G7298	-1.829233529	0.406916135	1
TTC21B	-0.183089488	0.849439465	1
PFDN5	1.026917132	0.394306687	1
FNIP1	0.812307867	0.557408505	1
MRPL11	1.037531851	0.425171812	1
AHNAK	-0.650687705	0.584808853	1
EIF6	1.515216412	0.227908381	1
QTRT1	0.419081002	0.682661616	1
SDK1	0.511129531	0.612267024	1
FAM3C	1.206082822	0.323492451	1

ABL1	-0.037497066	0.972230378	1
LYPLA1	0.453425726	0.657762006	1
DCAF13	1.015658284	0.43007856	1
DPH2	0.935510825	0.436242911	1
EIF4G3	-0.790331837	0.487195085	1
PDGFRB	-0.559174686	0.511945415	1
ZFYVE1	0.233810813	0.757957587	1
FAM53B	-0.58627266	0.59610205	1
NOL10	0.018313456	0.986742288	1
SLC49A4	-0.404101263	0.801891323	1
PWWP3A	-0.019502391	0.985410518	1
G10229	1.327399895	0.304073552	1
KCNC4	-1.161199558	0.306346984	1
PRPF18	0.907954827	0.514417693	1
GGCX	1.405142593	0.220723656	1
NEMF	0.936958517	0.434033501	1
KDELR1	0.095047316	0.922185332	1
ABCA7	1.706619504	0.128363743	1
SEL1L	0.511156589	0.574129018	1
OGFOD1	-0.025975782	0.9786151	1
MLYCD	-0.531506567	0.629591151	1
DDX19A	-0.469008451	0.631938245	1
PLCD3	1.12384057	0.328807196	1
PLPP3	0.051429529	0.959021598	1
LPL	-0.334097225	0.81890828	1
CNBP	-0.461519587	0.637851013	1
DNAAF5	0.065144153	0.945089332	1
CAV1	-0.906729998	0.564387598	1
PLXNA2	0.371763503	0.726214644	1
MT2A	0.669997248	0.495223785	1
METTL26	0.68184822	0.487319686	1
SBSN	1.349445601	0.765658115	1
RAB21	0.466407344	0.715071103	1
SPATS2L	0.337765684	0.850987988	1
PDE4C	-1.004237115	0.396554842	1
RNF220	-0.686318241	0.676135382	1
ANAPC4	1.143127989	0.399800178	1
VPS37A	1.003913847	0.456344655	1
RNF216	-0.470071562	0.629464	1
ANKLE2	0.880483249	0.487156984	1
PSTPIP2	-0.921190661	0.559277618	1
MIPEP	-0.193121534	0.840864459	1
KLHL36	-0.079429704	0.944437465	1
VPS53	0.413931614	0.67551146	1
KAT5	1.384133325	0.302640393	1
SH3BGRL3	1.25599782	0.243284501	1
MTMR1	-0.49093294	0.744999154	1
PPP4R1	-0.440283064	0.69794908	1
IL13RA1	0.937113295	0.502637994	1
ENSMMSG00000018851	0.756801181	0.773684852	1
STAT3	-0.055913401	0.954551932	1
TOR1AIP1	0.473343131	0.672500324	1
BRPF1	0.088916416	0.930366984	1
OGN	-0.225876202	0.853386255	1
UBE2R2	-0.441760093	0.674278233	1
GTF2F1	0.898837048	0.465958118	1
GRSF1	-0.334865278	0.742706076	1
PI16	0.536297574	0.637543453	1

ATP6V0B	-0.166462111	0.86058292	1
KAT6B	-0.078582429	0.939907172	1
RABEP2	2.092456354	0.133510268	1
'HIST1H2BC/LOC100441051	0.662002077	0.588199467	1
MRPL58	-0.816437031	0.377688158	1
DNAJB2	-0.416986754	0.71240864	1
TLE2	1.626033003	0.363106734	1
UQCC1	-0.672383995	0.347699505	1
MFAP1	0.293002788	0.788685499	1
CLASRP	0.688331085	0.505773986	1
TNFRSF18	-1.875411644	0.395855049	1
ACSS2	0.900112442	0.505711062	1
DIP2C	-1.011439455	0.32818472	1
PDCD4	0.99570978	0.392890873	1
LEO1	0.041387412	0.965443265	1
IREB2	-0.211586769	0.819044987	1
MAEA	-0.103287807	0.906342081	1
NDST2	0.323647671	0.73129581	1
APPBP2	-0.503284754	0.636010821	1
RANBP3	0.084839132	0.935369969	1
POLR1B	-0.396809241	0.711261162	1
NDUFA1	-0.93575999	0.34793494	1
ITGB4	1.35258183	0.186909978	1
PAGR1	-0.212660037	0.831972859	1
ERLIN2	1.969706335	0.154190228	1
BEND6	0	1	1
NQO1	0.598066241	0.703987965	1
TULP3	-0.504597999	0.655717413	1
DCAKD	1.337898261	0.225701103	1
TMEM43	0.262520024	0.799036201	1
ARMC8	-0.214711097	0.858679655	1
DPP3	0.245248289	0.757796256	1
MRPL52	0.475654423	0.689035666	1
LNPK	0.673582332	0.617551456	1
U2AF1	0.2207004	0.835395909	1
SEC31B	-0.580129301	0.711386735	1
MED24	0.555107468	0.625528704	1
MCFD2	-0.6968344	0.488107041	1
XRCC5	-0.19739823	0.847921225	1
H1-10	0.744857267	0.510169163	1
STAC2	3.268249095	0.096218348	1
HSPA14	-0.144657112	0.891275789	1
KCNJ12	-1.165277309	0.428195655	1
RHOT1	-0.431262622	0.697146862	1
ECHDC3	1.456014512	0.234055921	1
SIPA1L2	0.44474914	0.685209435	1
PLBD2	0.297106171	0.818463456	1
MRPS23	-0.266685292	0.780412648	1
DGKQ	0.995558271	0.296549311	1
MDP1	-0.465761543	0.616533392	1
CHD6	0.168047872	0.860204907	1
SUGP1	0.457689529	0.670903671	1
MAP2K7	-0.11085417	0.913617376	1
CUEDC1	-0.115853109	0.932968079	1
NAPG	-0.01737891	0.986602116	1
KMT5B	-0.274723331	0.781396669	1
ALDH5A1	1.415998478	0.272350647	1
CCNK	-0.148314215	0.891298776	1

ELF1	0.366144959	0.710990409	1
ZSWIM8	-0.028788935	0.98585922	1
CEMIP2	-0.375740413	0.693381458	1
COL18A1	2.479921779	0.161180438	1
KIAA1109	0.35279303	0.753354072	1
CRBN	-0.380238655	0.724401801	1
DMKN	-5.94998428	0.170585623	1
SPAST	0.976670766	0.446326554	1
SORBS3	0.030434762	0.978702598	1
IRAK1	-0.594811187	0.582320578	1
GPBP1L1	-0.067406855	0.975989139	1
DPH7	1.444752364	0.214859049	1
STAU2	0.389878686	0.822207874	1
NT5C2	0.741292908	0.688138632	1
SERPINF2	-0.182842085	0.856670356	1
KANK1	-1.132260582	0.634867701	1
SCAF4	-0.206291156	0.896832995	1
CCDC124	-0.083841029	0.939594846	1
FAM83G	1.824820533	0.11533739	1
WDR13	0.287796787	0.787445882	1
EGLN2	-0.442542271	0.68739194	1
MAPKAPK5	-0.21356809	0.822299904	1
FAT1	0.057155424	0.954358067	1
ERCC1	0.23016288	0.823251403	1
EXOSC9	-0.254923327	0.797531221	1
RTL8C/RTL8B/RTL8A	1.229026355	0.364652871	1
WDR45	-0.743754835	0.470568691	1
EXOC2	-0.653394022	0.673353486	1
KAT14	-0.055278825	0.972814186	1
MANF	1.268030254	0.223403831	1
SCAMP2	0.737273594	0.486941874	1
ITGAV	0.316447468	0.743782953	1
STXBP3	1.142233103	0.391282028	1
PTDSS2	-0.480438218	0.618787122	1
ENSBTAG00000006019	-2.848438002	0.132295379	1
RIMKLB	2.290394869	0.136724323	1
RNF214	-0.345835053	0.64617144	1
SLMAP	0.945559105	0.472782402	1
MXRA5	-0.120415121	0.91051938	1
EMC9	-0.090778149	0.931075569	1
MYH14	-0.310243654	0.728622172	1
PIP4K2C	1.125356695	0.185690351	1
COX7A2L	-0.137854148	0.903323612	1
N4BP1	-0.800763545	0.41149521	1
SNX13	0.153596577	0.897441159	1
YTHDC1	0.476791419	0.683212454	1
REV3L	-0.188753395	0.843824587	1
TMEM131L	0.671469494	0.617878263	1
HGSNAT	0	1	1
HDAC2	-0.464047081	0.621810878	1
ITGA3	1.13326358	0.230928684	1
DYNC1LI1	-0.069923988	0.94568898	1
MKLN1	-0.138868545	0.889942985	1
TIMP3	-0.633844745	0.524767954	1
APEX1	-0.239408109	0.80399013	1
CCDC88C	-0.675394477	0.672949021	1
TRANK1	1.091620628	0.543647898	1
FOXO3	-0.414545221	0.746777569	1

CEP350	0.820823943	0.494706273	1
UBQLN4	-0.070732147	0.946508392	1
AMACR	0.160368714	0.853789387	1
NEU1	1.089483112	0.28260157	1
NOTCH3	-0.067195301	0.942742198	1
GALNT1	-0.327920924	0.773692638	1
LITAF	1.183877381	0.4315308	1
PCNX1	-0.572184215	0.49837961	1
FAAH	1.976175304	0.109269294	1
R3HDM2	0.568936464	0.73109905	1
TJP2	1.73120035	0.200713688	1
PHLDB3	0.462577588	0.693277077	1
WDR3	-0.338141561	0.721704861	1
ADAM9	-0.613360547	0.686614144	1
UTP11	0.971132846	0.463280922	1
REV1	-0.65055765	0.489329449	1
MFF	-0.583041054	0.500921911	1
DMXL1	1.035448041	0.575806957	1
SPAG9	-0.648000926	0.41835227	1
OSBPL7	0.337208004	0.796621638	1
RPS6KB1	-0.291855051	0.77611843	1
ESYT1	-0.108393883	0.898345622	1
SREK1	1.643290603	0.170032179	1
RANBP1	1.369970104	0.241622534	1
GBA	1.15325524	0.209785929	1
RHBDF1	1.39599756	0.32070025	1
RTN4RL2	0	1	1
PTPN4	1.821643806	0.186580483	1
THOC5	0.178385064	0.875332017	1
FBLN2	-0.408942305	0.674367199	1
HIPK1	0.410138044	0.740498963	1
MEPCE	-0.249316291	0.781463669	1
VPS25	-0.157383342	0.85203567	1
EDEM1	0.914273703	0.326798295	1
FBXO11	-0.182944589	0.873425523	1
LMOD1	-0.343015362	0.771379662	1
ACSS1	0.395263704	0.78424451	1
BBS4	-0.365235711	0.730402672	1
XPO6	0.873886673	0.389766481	1
PAK3	2.122260905	0.132941203	1
NUDT19	0.315378879	0.768454935	1
MRPL28	0.179566311	0.86884231	1
PLS3	-0.211705307	0.850478467	1
BANF1	-0.442936812	0.667446185	1
PKP4	-0.368530551	0.68429271	1
MRPL39	-0.445928156	0.615757984	1
MMS19	-0.746163509	0.656045304	1
ROMO1	0.062815177	0.954091879	1
ZNF76	-0.581760134	0.6122587	1
SMAD2	-0.493092376	0.553744352	1
NFYC	0.24233061	0.8321695	1
TMEM165	1.098356991	0.332368972	1
WNT10A	0	1	1
VPS29	0.151478804	0.883173433	1
REXO4	-0.510845701	0.64704886	1
AP5B1	0.355398695	0.750683403	1
RPS14	2.527245628	0.086541056	1
SMCHD1	0.786809398	0.657525276	1

NUP188	0.27547092	0.761270242	1
CFDP2	0.071934014	0.949292894	1
UTP18	-0.242454307	0.817318543	1
GFM2	-0.765621821	0.627950964	1
LOC615514/GSTM2	-1.059103498	0.504034555	1
XRCC6	0.781400724	0.514186208	1
RUVBL2	0.615119458	0.553310034	1
IL11RA	-0.752877441	0.496773833	1
MCM5	-0.221354698	0.836254574	1
ACSL3	0.913766145	0.424092118	1
SBNO1	-0.117057622	0.898480723	1
TLK1	1.188469934	0.319319021	1
LRRC59	0.449551248	0.641623384	1
UBR1	0.520442289	0.66147961	1
ATF3	-0.758002473	0.465606412	1
PTPA	-0.425385998	0.589322357	1
MACO1	0.797610224	0.523662718	1
NOC3L	-0.051860404	0.958770176	1
ADAMTS1	-0.814301914	0.455465967	1
ANKRD52	0.627483973	0.659034851	1
WAC	-0.225168272	0.841905293	1
RMC1	0.24527038	0.762702559	1
GPATCH8	0.711335019	0.601965175	1
LAPTM4B	-0.325297881	0.759563324	1
SMG6	-0.308386353	0.75127196	1
CCDC142	-0.372554557	0.621215446	1
MAP4K3	-0.078235087	0.963066	1
FBXO38	1.131141411	0.4267785	1
TCAF1	0.78448674	0.450344814	1
GRWD1	-0.469443414	0.670516103	1
VBP1	-0.422335791	0.670937563	1
CTSZ	-0.073941062	0.94506607	1
DPF2	-0.038891437	0.960219177	1
TBCE	0.083075715	0.94034099	1
HLCS	0.102194828	0.910502215	1
RBSN	0.153573675	0.88708684	1
CDC16	-0.196975124	0.835082284	1
DLG1	-0.031253331	0.975908981	1
PEX5	-0.288273165	0.856115586	1
ACAD9	0.360311832	0.677033637	1
TENT4A	0.616409434	0.596422968	1
GPR39	3.893843084	0.100588811	1
CEP120	-0.68513799	0.656419496	1
MRPL49	-0.590832356	0.558032654	1
PRPF40B	1.044303656	0.427710365	1
TKFC	1.098100943	0.170155506	1
NABP2	0.722114686	0.568882134	1
TUT7	-0.211642735	0.832462208	1
POLR1D	-0.198102142	0.816317208	1
B3GALNT2	0.527926124	0.684602742	1
ASB16	-0.721097442	0.515854624	1
NUDT3	-0.507195749	0.600275055	1
SUMO1	-0.454505518	0.65161407	1
DHX36	0.421752977	0.725540191	1
INPP5K	-0.016991089	0.982985891	1
MARK2	0.775125868	0.499615613	1
RPL23A	0.694877727	0.625020521	1
SMARCB1	2.195242614	0.099832831	1

DDR1	1.294197259	0.341585319	1
POLR3B	0.619255928	0.726815222	1
ANO8	-0.225958806	0.821020042	1
MBNL2	-0.637513806	0.572254889	1
ZFHX3	-0.190580836	0.841329696	1
PIM3	0.269238752	0.795716815	1
KRTCAP2	-0.066126051	0.942473449	1
MRPL3	-0.265404931	0.764801837	1
LOC530929	2.410801947	0.090595059	1
YIF1A	-0.325425723	0.733485416	1
RABGEF1	0.420803467	0.652275671	1
SLC12A2	-0.846974476	0.453866613	1
THOC2	0.415148614	0.718451557	1
NFIB	1.024132525	0.630894202	1
SRSF9	0.805711761	0.377104549	1
DRG2	-0.471170672	0.60362923	1
TMED9	0.112199524	0.895923148	1
CSTF3	0.708338052	0.517352874	1
ANKRD34C	1.933395642	0.101534393	1
U2SURP	0.337943564	0.774002011	1
EIF2D	0.715200779	0.588749564	1
PLA2G12A	0.907116624	0.355559094	1
GFUS	0.242059582	0.805654741	1
CAPN1	-0.020530376	0.986952598	1
GINM1	0.450816962	0.64840308	1
GIT1	-0.563498194	0.604162552	1
IFRD1	0.053654468	0.961051183	1
COPS2	-0.381715914	0.817433772	1
FTSJ3	0.240278467	0.758626631	1
ELP2	0.81569493	0.523229067	1
PCMT1	-0.758054866	0.319949917	1
MATN2	1.071607198	0.25137989	1
CLASP1	-0.45065379	0.70022064	1
PPP1R13L	0.107912097	0.920364717	1
DVL3	0.592944282	0.578644699	1
MPND	0.442367171	0.657536635	1
LAMC3	1.07453257	0.365314382	1
MFSD14A	0.707154325	0.651237948	1
FUCA2	0.938463672	0.357127599	1
FAM234A	-0.282087882	0.764853794	1
WDFY3	0.002495573	0.998175262	1
RNPC3	1.093393005	0.399742855	1
TOP3A	0.076670557	0.953086344	1
MRPL13	-0.190280704	0.854881839	1
RBM8A	0.106795173	0.916832521	1
DOCK1	-0.057377728	0.950233784	1
DOT1L	0.617171242	0.627290071	1
POLDIP3	0.300541386	0.757597726	1
NHP2	0.1367352	0.878115781	1
PIP4K2B	-0.552591876	0.616245453	1
ZHX2	-0.756137636	0.423998345	1
PIAS2	-0.555017741	0.567286319	1
DALRD3	0.906008849	0.328535689	1
DHX8	0.712280832	0.575901844	1
GALK2	1.793767152	0.117776596	1
PHLDA3	2.347313681	0.105739672	1
EFEMP2	-0.185034234	0.84603414	1
ACTN1	0.827429348	0.434709246	1

MPI	0.056388746	0.966475431	1
TMED2	-0.880085369	0.382938455	1
RPLP0	-0.323510902	0.742997058	1
MRPL21	-0.614006537	0.602012003	1
GPBP1	0.541210358	0.610849819	1
MED15	-0.113316587	0.918205158	1
HTRA1	-0.638822021	0.502329615	1
PIK3R2	1.173644111	0.233218524	1
CD2BP2	-0.185327027	0.845283616	1
MRPL16	-0.217858276	0.835628383	1
TP53BP1	0.728517671	0.501678684	1
PPP4R3A	-0.341314086	0.82882049	1
USF2	-0.724614723	0.513440305	1
MYL12B	2.07094785	0.135373828	1
WDR81	0.148559757	0.896717378	1
SETDB1	0.281350403	0.764036896	1
GAB2	0.387319259	0.830328899	1
SNF8	-0.221503291	0.826507502	1
GNE	1.292210002	0.284305436	1
TMEM260	1.351968892	0.280992332	1
COL5A1	0.282584914	0.761722932	1
GCDH	-0.385574652	0.724818938	1
NRBP2	1.168815108	0.24770021	1
XBP1	0.300517228	0.784598234	1
INPP5A	-0.458887798	0.716355229	1
PABPC4	-0.99344242	0.519389599	1
RPN2	-0.891738496	0.482312371	1
ANKRD12	0.717799741	0.67408031	1
BCKDK	-0.774247801	0.407216291	1
AUTS2	0.918912464	0.522970062	1
CCDC93	-0.375585129	0.720302625	1
TPD52L2	0.095305504	0.930808193	1
PTPN2	-0.08189334	0.931474532	1
MAN1C1	1.601349025	0.131949262	1
GTF2I	-0.037957263	0.963798328	1
TOX4	0.762884474	0.519206219	1
ERGIC2	0.072672153	0.937036097	1
SEC23A	-0.547137496	0.574255916	1
SETD5	-0.116832326	0.944735208	1
ARHGAP10	0.677433905	0.576210917	1
ALS2	-0.011815307	0.994111769	1
SYF2	0.09170021	0.930815037	1
KLHL21	-0.440693769	0.802447227	1
HEATR6	-0.195496273	0.825061037	1
ATG2B	-0.491722829	0.633109813	1
SCAF8	0.315225608	0.80298887	1
MAP3K6	1.240426721	0.214565747	1
HIBCH	-0.322136845	0.736925235	1
ADAM17	0.542291464	0.549616526	1
NELFA	0.218222233	0.821746451	1
DDX21	-0.244808269	0.796993183	1
DNAJC19	-0.884123429	0.361961428	1
BPHL	0.179214724	0.846589405	1
NR2F6	0.374480923	0.717510984	1
EIF4ENIF1	0.794210285	0.658958977	1
ANKS1A	-0.140059323	0.895696578	1
TSC1	-0.375929544	0.755929945	1
ZFAND6	-0.855907144	0.311457023	1

IKBKB	0.527529628	0.701655056	1
ADAM15	0.308501472	0.785333901	1
MLST8	-0.732174919	0.466312853	1
MAP7D1	-0.5686146	0.728430104	1
ZMAT2	-0.077060814	0.93442305	1
EIF4E	-0.35244553	0.753375261	1
TULP4	-0.363178109	0.758703565	1
SMPD4	0.270234214	0.791358432	1
CHD1	0.179099433	0.861585505	1
ANAPC1	0.407306525	0.667951058	1
CCDC80	-0.163106181	0.901896532	1
MOB1A	-0.396615872	0.674671252	1
LSG1	-0.146868399	0.871747405	1
CSNK2A2	2.236729142	0.114442933	1
GRINA	0.501510474	0.561770016	1
DUSP3	-0.770698868	0.461788114	1
CSNK1G2	-0.359713685	0.689867133	1
MRPL23	0.551674262	0.643343035	1
TRPM7	0.881438074	0.484463085	1
TCF20	1.029049926	0.317697358	1
LSM14B	0.398503372	0.775724493	1
MAN1B1	0.90144212	0.372840431	1
SYNJ2	1.926043882	0.170039898	1
SURF6	-0.455992388	0.670292503	1
AAMP	-0.377537412	0.821234921	1
SLC25A13	1.733347883	0.372123549	1
TMEM161A	-0.307396208	0.742684864	1
ACAD11	0.51532551	0.67318126	1
SMYD1	-0.124003903	0.940111054	1
SFI1	1.444799508	0.119872062	1
BRD7	0.522475076	0.639187001	1
ENSMMSG00000006522	0.813182242	0.355531992	1
NAA60	0.311670366	0.779155742	1
KMT2D	0.223432616	0.862334321	1
SYPL1	-0.361067395	0.64771619	1
MAST1	-1.830781858	0.396054472	1
PDPR	-0.685071342	0.678037897	1
G33898	1.309572699	0.138654719	1
STRADB	-0.938385352	0.540497084	1
MAPT	-0.210628765	0.86300354	1
BTBD6	-0.552612746	0.614967064	1
ILRUN	-0.900021	0.347996954	1
KREMEN1	-0.270953857	0.812902571	1
USP16	0.680673893	0.567537531	1
CES1P1	2.230225073	0.252737488	1
COPS8	-0.848417096	0.326401678	1
TBC1D14	1.078380456	0.284161989	1
SELENBP1	0.766170109	0.482313025	1
ATP2B1	-0.726346838	0.688405641	1
TP53BP2	0.640747483	0.618817846	1
GABARAPL2	-0.386716043	0.680765401	1
TBCB	-0.358276328	0.735108888	1
RTCB	-0.425267962	0.654805316	1
FAM193B	0.100918725	0.920055845	1
ADAM10	0.373927383	0.76730325	1
GSE1	0.404243635	0.677013147	1
HDAC6	0.320141074	0.717762471	1
SGCB	0.210063121	0.905017953	1

RTEL1	1.511898648	0.203368762	1
ENAH	0.909969016	0.516968234	1
TMEM11	-0.606470959	0.560502433	1
GALNT11	-0.069065418	0.9391684	1
IDS	1.167039486	0.52059189	1
NAA15	-0.469853622	0.63412669	1
C23H6ORF89	0.739145773	0.430881491	1
MFSD14B	1.685718101	0.124789061	1
PPP1R2	-0.405383462	0.719529547	1
EPB41	0.441784431	0.631462006	1
PELP1	1.427759891	0.168679044	1
HDAC1	0.339241673	0.759196693	1
CDK10	1.262085837	0.26197444	1
MVD	0.000535833	0.999664464	1
IFNGR2	0.174341668	0.844318057	1
KLHL38	-0.757260215	0.616412001	1
MRPL45	0.241121566	0.837377358	1
STK40	-0.528113308	0.619063868	1
KMT5A	-0.091260325	0.932432151	1
KATNA1	-0.067954774	0.946611832	1
C1S	0.02254179	0.981784876	1
FCGBP	0.289885738	0.77526466	1
RPL22L1	1.406836924	0.24320585	1
TSR1	-0.231450924	0.824015699	1
LMBRD1	0.066558903	0.946447438	1
ABHD17A	-0.492376976	0.592992767	1
CRYZ	0.014157719	0.990924708	1
CDC42EP1	-0.039268825	0.971769424	1
BAP1	-0.518203335	0.610621601	1
SLC38A2	-0.224985998	0.826542647	1
KSR1	-0.559752242	0.726578307	1
KIDINS220	0.859056565	0.618807876	1
MAP2K1	-0.563363831	0.626384743	1
SENP6	-0.499083734	0.760270522	1
GOLM2	0.033509532	0.983574322	1
MDC1	0.232444027	0.824686075	1
UROD	-0.263586039	0.813424698	1
TM9SF1	0.417669327	0.66579642	1
SEMA3C	1.273955611	0.489007854	1
POLR2F	-0.248852473	0.820235091	1
SART3	0.998284224	0.470045991	1
ATF5	-0.177027501	0.8492376	1
HCFC1R1	1.065774566	0.420421239	1
UQCC2	-0.329269712	0.738955769	1
GANAB	-0.31992847	0.664580238	1
PDCD6	1.02652168	0.265634621	1
MTSS2	-0.303558728	0.792267709	1
PIN1	-0.728821137	0.326153664	1
MOGS	1.082883554	0.250369317	1
FBXO3	-0.196309725	0.905937707	1
TRIM24	1.430455652	0.420003419	1
SF3B4	0.635925508	0.597515204	1
CMBL	-0.152807	0.924880685	1
SNX8	1.207647646	0.220833512	1
UBL3	-0.098905508	0.921459279	1
FXR2	0.825596529	0.657041259	1
VPS26B	-0.162697356	0.86512979	1
TMEM183A	0.784519492	0.477939571	1

ICE1	0.053219275	0.964630109	1
UBALD1	-0.36479134	0.675609721	1
PHF20	1.593027956	0.232376384	1
RHOB	-0.535788074	0.646968611	1
ATP6V1H	0.342562713	0.677769336	1
TCOF1	0.031201923	0.979005662	1
RFC1	0.620388738	0.596324276	1
LAMTOR4	0.511075642	0.592167471	1
ENSMMSG00000005582	0.338854676	0.76893308	1
FBRS	0.620099835	0.502069245	1
BOD1L1	0.886187499	0.431431678	1
ATPAF1	-0.8494767	0.385747851	1
GARS1	-0.19962176	0.815809109	1
CDC123	-0.055424917	0.953598168	1
SNX14	-0.189157389	0.823984753	1
EXOSC10	-0.235292156	0.826523626	1
SCHIP1	0.253337162	0.858767748	1
LRRFIP1	0.884270333	0.57375138	1
CAD	0.530029369	0.557774425	1
RAB11FIP3	0.187385732	0.887825286	1
BPTF	-0.292454237	0.792801414	1
AP2B1	0.45312165	0.778674114	1
LACTB2	0.369029558	0.82056406	1
DECR1	0.723636904	0.559484793	1
SAR1B	-0.717455901	0.652037633	1
NDST1	0.886698685	0.455647986	1
UBXN4	0.040327775	0.969260367	1
CILP	-0.159039569	0.899984089	1
OSBPL6	2.946788219	0.224390181	1
PSENEN	-0.274033722	0.786299946	1
ACTC1	0.333190309	0.844799209	1
DNPEP	0.079971603	0.942719009	1
FKBP9	0.077664972	0.913693057	1
RAB5A	-0.379852885	0.704836528	1
TMEM214	-0.061899241	0.950962485	1
NOSIP	1.512706177	0.286794981	1
BCLAF1	0.493293381	0.682069841	1
NUP155	-0.301293547	0.764024582	1
AATF	-0.080090096	0.932650183	1
NCOR2	0.443771217	0.807851116	1
LOC782101	-0.482320942	0.662615193	1
FLYWCH1	-0.066577827	0.954266308	1
FMO3	1.06933801	0.264159074	1
AMOT	-0.824946285	0.605362862	1
IMPDH2	0.066165606	0.966889535	1
SPAG9	0.62914562	0.721818863	1
GTF3C1	0.468171122	0.642901246	1
DEDD	-0.661636563	0.509553134	1
CEP250	0.845694676	0.294322141	1
H2BC21	-0.066204782	0.966645421	1
SETD5	1.002567216	0.591696702	1
R3HCC1	0.364841677	0.787421304	1
CHD4	-0.052358827	0.955881609	1
TARDBP	0.143954272	0.896339725	1
ZC3H13	0.659103241	0.591007159	1
ANKRD10	-0.984204446	0.329261185	1
RAB18	-0.080564247	0.94351662	1
PEPD	0.012723098	0.989928414	1

NCOA1	1.353049129	0.309603041	1
HIVEP2	-0.640449161	0.54094535	1
PEX5	0.061953175	0.954625269	1
HNRNPC	0.336622715	0.841898454	1
PATJ	1.460092777	0.262094336	1
CLPTM1L	1.230630379	0.332099641	1
FOCAD	0.160795041	0.862108183	1
TXN	1.200582681	0.262764238	1
SPSB3	0.037192755	0.969310792	1
KANK1	-0.794897091	0.340720588	1
SLC30A5	0.862624851	0.439510511	1
TFIP11	0.99749365	0.462819157	1
UFL1	0.168588834	0.88171983	1
MRPL40	0.287126476	0.799407797	1
CES1	2.003598971	0.398991521	1
RCC2	1.646541028	0.162448718	1
MRPS18A	-0.364647515	0.723424774	1
RAB11FIP4	3.493502748	0.137234043	1
TTC38	1.443994949	0.286349311	1
AASDHPPT	-0.025292159	0.981589635	1
WDR26	1.184290173	0.52654857	1
ELP1	-0.474458947	0.671190428	1
CXXC1	0.999691769	0.433339973	1
ELP5	-0.281947305	0.720006575	1
DOCK6	-0.071877972	0.945470445	1
GPR107	0.66626026	0.6969852	1
USP36	0.982079987	0.293419913	1
MINK1	-0.360390886	0.664641774	1
TSC22D4	0.55671199	0.618779031	1
UBR5	-0.287378135	0.909222275	1
DOP1B	0.287912046	0.787854785	1
COP1	-0.33304101	0.72166286	1
AXL	-1.443118498	0.728597127	1
KDM2B	0.147879277	0.901868711	1
TM6SF1	0.082539191	0.963039263	1
MAP1LC3A	0.135952493	0.910450327	1
TRMT112	0.028293089	0.97782843	1
OSBPL1A	-0.535113557	0.604402996	1
TRAPPC10	0.090179142	0.936597206	1
KLHL22	0.158225822	0.883206861	1
RNF113A	0.08240409	0.940944856	1
ZMYND11	-0.669979736	0.674611425	1
MCCC1	0.100666066	0.917603842	1
ZNFX1	0.283268431	0.800325449	1
MTMR3	-0.661163023	0.64058	1
MIDEAS	0.958072123	0.429810563	1
BRF1	-0.251789919	0.79394752	1
BCR	0.620573567	0.563371256	1
FXR2	-0.445603188	0.714109503	1
USP15	-0.423781267	0.669005796	1
TRAPPC2L	0.872210464	0.459665769	1
UXS1	-0.706235264	0.461264755	1
ACAA1	0.503698012	0.69874907	1
GBE1	0.669623244	0.71184229	1
DNAJB6	-0.500105269	0.505908159	1
ABCB7	-0.613500193	0.509221115	1
CNOT2	0.020827899	0.9901432	1
SEH1L	-0.397229374	0.804566223	1

FNDC3B	0.839715566	0.504346262	1
SLC2A4	-0.35758706	0.817273424	1
AGGF1	0.459151187	0.694162059	1
NUP98	-0.186947996	0.864969656	1
PYGB	0.797453886	0.411867118	1
ATP6V1A	1.09566367	0.313097133	1
MRPL9	-0.470975013	0.704997677	1
TCAIM	-0.033892356	0.984517142	1
PDZD2	0.67875046	0.62127154	1
PI4KA	0.057728257	0.96428038	1
CAMK2A	-0.996694425	0.506383952	1
ZER1	-0.594675312	0.583001973	1
ABCA5	0.102345119	0.925898679	1
INO80	-0.454664832	0.61848195	1
DENND4C	0.367509708	0.69711016	1
GPBP1L1	0.352077845	0.720455566	1
RTN3	0.833013674	0.41395455	1
C16H1ORF21	0.278561215	0.823005852	1
SEC61G	0.595583424	0.564610996	1
NAA25	0.01834512	0.984715868	1
PADI2	-1.424854687	0.365465946	1
CUTA	0.345253122	0.744395783	1
INPP5J	-2.848787325	0.138328385	1
ARGLU1	-0.128090804	0.886247497	1
CTDSP2	-0.437958638	0.703161521	1
FARP1	0.526690631	0.544635008	1
OGFOD2	0.799709338	0.466729756	1
PTPN3	1.45334253	0.293569145	1
MINDY1	-0.170946047	0.863026634	1
ATP11A	0.900907148	0.491792565	1
RMND5B	-0.003771903	0.997217819	1
IWS1	0.739587077	0.536498231	1
PANK4	-0.746391595	0.470564907	1
MRPS5	-0.651474977	0.496816004	1
ACBD3	-0.1003563	0.913668671	1
ATP9B	0.581116545	0.560258537	1
H2AC6	1.592700754	0.205446544	1
ASCC3	0.94148677	0.45788335	1
SMC6	-0.222988331	0.825478162	1
HID1	2.041844421	0.097370442	1
MAPKAP1	-0.591882054	0.530121012	1
NUDT22	0.412543692	0.694994863	1
MEF2C	-0.030522937	0.986032158	1
PPM1A	-0.646663749	0.677688384	1
FN1	-0.336396299	0.715894841	1
GRAMD1A	0.625250084	0.590959913	1
SIDT2	0.212753372	0.826518988	1
RPS6KB2	0.639971459	0.593521964	1
KPNA6	-0.345190927	0.710184911	1
PYURF/PIGY	-0.640059571	0.500109979	1
TARBP1	0.66491564	0.552448886	1
ZNF207	-0.409191165	0.691798847	1
CLCN3	0.189910585	0.860264193	1
KTN1	-0.729474649	0.463885769	1
VPS8	0.843129957	0.523252456	1
TUBA1D	0.246359195	0.851634906	1
ERBIN	1.003708274	0.43982524	1
DNM2	0.147573521	0.88164314	1

PPP1R12C	0.888560608	0.525243578	1
IPO4	-0.261690373	0.786689489	1
TAF6L	0.248422259	0.799138068	1
SNRPD2	0.650606415	0.580213119	1
TIAL1	-0.579319868	0.723305472	1
FABP5	1.095429717	0.258397145	1
SH2B1	-0.523188702	0.570769053	1
LIMK2	1.204115837	0.515064594	1
IGFBP5	0.365589533	0.738630212	1
LAGE3	0.415338096	0.748328436	1
FUBP1	0.460579066	0.691994544	1
SYNJ1	-0.39235262	0.691061403	1
CHPF	1.135754329	0.329771207	1
SLC25A10	-0.456363464	0.63233882	1
KYAT3	0.141446128	0.887302299	1
KAT8	-0.215794959	0.815915863	1
PFDN5	1.301700344	0.236389378	1
CASZ1	0.537725738	0.610851409	1
ACOX1	0.261022274	0.827295312	1
ALDH9A1	1.54897354	0.275220279	1
PPP5C	-0.652414269	0.509860098	1
RRP12	-0.648710849	0.356637569	1
NCAPH2	0.693205629	0.526865861	1
PNN	0.514671898	0.637655853	1
HTT	0.416668946	0.768632737	1
GNB1L	0.76499319	0.559495506	1
PEX3	1.487423076	0.1882418	1
DDX27	-0.154709701	0.874221264	1
IRX1	3.634475001	0.119512309	1
TXNDC15	0.11356935	0.917151518	1
ATP6AP2	1.663806123	0.091510425	1
SLC25A20	-0.501330298	0.759890642	1
TGFBR3	1.033142042	0.312863987	1
NDRG3	0.999270758	0.336094987	1
AP3S1	0.808683667	0.433214675	1
PPP1R8	0.187915154	0.845305794	1
ARFGAP2	-0.324048615	0.786163168	1
ZBTB38	1.149310649	0.380528377	1
EPS15	1.016825704	0.531901076	1
WDR43	0.466728734	0.676122066	1
PCNP	-0.227765322	0.814490879	1
TRIM2	2.319828257	0.10282549	1
BICD2	-0.176619017	0.878465359	1
SGTA	-0.55438854	0.574528591	1
SRF	-0.787928306	0.303786814	1
MRPL27	0.362211533	0.719683781	1
GNL3	-0.365964291	0.735961597	1
FBXW8	-0.263547662	0.759716065	1
MTUS1	-0.635157993	0.550987749	1
ZC3H7A	0.306105892	0.790799657	1
PLCE1	1.776202962	0.161845919	1
CACNB1	-0.532296909	0.710695101	1
CFAP36	-0.288543483	0.757487412	1
RNPS1	-0.714345138	0.553325155	1
ZC3H7B	-0.153464237	0.880158368	1
OTUB1	0.271068789	0.789932992	1
PRCC	-0.345482833	0.724229383	1
GNL1	0.893576765	0.316022228	1

EXOC1	0.201387706	0.81885512	1
TTC37	-0.489947254	0.619513683	1
RANGAP1	0.160394994	0.870269913	1
PRPF4B	-0.057591637	0.955882395	1
MINK1	1.762994313	0.175859048	1
DERL2	1.129367647	0.345788126	1
SIPA1L3	0.869320858	0.421569901	1
C15H11ORF58	-0.441384955	0.52793421	1
STARD3	0.714581274	0.451697282	1
VPS45	0.040154156	0.965537173	1
RBM17	-0.582897593	0.501237377	1
MSL3	-0.225881397	0.815951883	1
TMED2	0.578756472	0.652352386	1
KIFAP3	-0.380628056	0.696583826	1
ENSMMSG00000014290	0.14398268	0.874946856	1
HMGCR	1.384063283	0.105181609	1
RPS27L/LOC101903478	0.044334671	0.96560694	1
MBD6	0.328442259	0.755296184	1
CD82	-0.515413068	0.670494447	1
CZIB	0.890549721	0.48709991	1
PPP6R3	-0.552963465	0.745360407	1
AP1M2	0	1	1
SF3A3	-0.282999749	0.791853175	1
PLXNB3	0.395280903	0.773801581	1
GMPS	0.9070346	0.457738368	1
CTDNEP1	-0.640053298	0.385611733	1
MTUS1	-0.84506999	0.601395814	1
EID1	0.038953077	0.968443894	1
VCL	0.106415653	0.925354362	1
PITPNM1	0.022531648	0.98151956	1
EP400	0.042260677	0.966124405	1
GCLM	0.813033465	0.474546497	1
DMD	-0.305160146	0.783422572	1
AKAP8	-0.320182203	0.734462971	1
USP11	-0.058186976	0.972493958	1
UBFD1	-0.517388298	0.577551093	1
MED12	1.025396415	0.394246385	1
PHF12	-0.178715375	0.801473147	1
VPS52	0.670202531	0.50810493	1
ZMIZ2	-0.378805508	0.676645693	1
PERM1	-0.673688296	0.618777788	1
KTN1	-0.977186844	0.537374857	1
SPECC1L	0.046865672	0.962029061	1
SPART	-0.466135283	0.6327255	1
AOX2	2.988912664	0.083391581	1
ACTR8	0.56300434	0.685099877	1
TRAPPC4	0.127560386	0.889817347	1
AADAT	0	1	1
TMEM182	-0.245928017	0.887039007	1
RAB14	0.194262935	0.833289486	1
MBD3	-1.055904619	0.306180705	1
MARK3	-0.816575104	0.493221848	1
PFKFB1	-0.226130144	0.902196833	1
PSMB6	0.375806516	0.709988642	1
ANAPC7	0.697518973	0.555864008	1
SH3GLB2	-0.064185227	0.95021162	1
CNN3	-0.609235974	0.533210898	1
GRHPR	-0.770318424	0.476401189	1

AKAP9	1.340022828	0.313911463	1
LUC7L	0.229184798	0.898311103	1
BCO2	1.064055791	0.400482475	1
MAP2K3	-0.34598062	0.7120318	1
PLPP7	-1.180271195	0.36312338	1
NUDT1	0.304861425	0.776551139	1
UCHL3	1.30957484	0.362231564	1
CENPB	-0.185813637	0.864060201	1
PDCD6IP	-0.659955546	0.42230372	1
MRPL15	-0.360707274	0.661572149	1
SEPTIN10	1.568472832	0.201771593	1
RGP1	-0.393016401	0.710535927	1
RAN	-0.399889354	0.667339759	1
RAB5C	-0.065532281	0.951704617	1
DYNC1I2	0.632667965	0.72054406	1
SERPINH1	-0.879596633	0.386650748	1
TFRC	0.456371649	0.732452837	1
NR1D1	0.239541879	0.835259363	1
VAPB	-0.313116428	0.728550448	1
DERL1	0.488428373	0.634194482	1
SLC4A1AP	0.58948981	0.626411796	1
ARHGAP32	1.63429928	0.118286307	1
HEXIM1	-0.682304829	0.45245422	1
PDCD11	-0.147580407	0.925646486	1
RABGAP1	0.433011815	0.706029708	1
LAS1L	0.468839845	0.66346725	1
MYOZ3	-1.263285358	0.426003691	1
TNRC6C	-0.287686182	0.799475286	1
RPS12	2.134701277	0.115338495	1
UBE2S	0.847707474	0.437541758	1
ADCY6	0.172481162	0.874521929	1
MRPL38	-0.369775691	0.724842869	1
ITGB5	-0.00474168	0.996383639	1
STAT6	0.655828625	0.535697834	1
PPP1R13B	-0.819741756	0.598428006	1
UCK1	0.892837383	0.323629643	1
CPSF2	0.804818589	0.538281669	1
ITPR3	1.012405525	0.278525732	1
METAP2	-0.226993175	0.806399587	1
HEXA	0.725944703	0.513135476	1
COL14A1	-0.145309927	0.883782682	1
TUBB2A	0.7608078	0.517899308	1
SECISBP2	0.057227696	0.958547553	1
BRD7	0.02339326	0.981511158	1
LMAN1	0.199634204	0.814091796	1
DHX29	-0.021857693	0.983701	1
ARHGEF6	-0.078746299	0.943346652	1
PHLPP1	-0.611679581	0.562512866	1
TBL3	0.532317443	0.630032278	1
DCUN1D1	-0.666201636	0.556060916	1
TOMM6	0.221250389	0.812580496	1
MICU1	-0.047539405	0.977704633	1
LOC516108	1.325021786	0.222899201	1
AQR	0.047248798	0.964772781	1
ACOX1	0.356438207	0.794444605	1
SSH3	1.390321378	0.315114798	1
MBD1/CFAP53	-0.120285936	0.897989619	1
CYP4B1	1.569928192	0.182859422	1

MAN1B1	0.801364442	0.456576568	1
G6578	0.893639246	0.450398048	1
SCARB2	0.030011855	0.974840174	1
SH3BP5	-0.968311481	0.386266845	1
DNAJC5B	0	1	1
TUBG1	-0.065643604	0.956383728	1
SPPL2A	-0.130385247	0.94008754	1
PFDN6	0.39724833	0.70273652	1
AHCYL1	0.138868147	0.896792353	1
ECI1	-0.321573171	0.758776914	1
RPA1	0.653002848	0.566349861	1
LPCAT3	-0.368284044	0.637746219	1
TXN2	-0.094116439	0.899476594	1
UBE3C	-0.468080565	0.784543809	1
PSME3	-0.084330063	0.926411175	1
CASKIN2	-0.465534546	0.61960715	1
ART3	-0.922357874	0.541830499	1
TRAPPC11	-0.362114608	0.71836255	1
STK24	-0.308839906	0.737929462	1
PBX1	1.330320094	0.281609738	1
PNKP	1.681364617	0.183085684	1
VARS2	1.237422865	0.375366456	1
SUPV3L1	0.380469121	0.689835219	1
PLXND1	-0.430674861	0.659792	1
CACYBP	0.098988231	0.919165127	1
DVL1	-0.371382191	0.735671256	1
NOLC1	-0.034661993	0.96733844	1
BUB3	0.035967613	0.97357088	1
EEA1	0.768017211	0.535370486	1
NAA10	1.062713302	0.38111803	1
OSBPL8	0.669588459	0.463749172	1
NARF	0.210238484	0.878125557	1
ARHGEF18	0.969672108	0.467649973	1
MAP7D1	-1.066040078	0.503514741	1
BOP1	0.946831452	0.444255416	1
LAMC1	-0.442358714	0.661815513	1
PREP	-0.678430056	0.457404437	1
NR1H2	-0.160300447	0.874120712	1
MKRN2	0.445575467	0.750655762	1
PSMG4	1.31074694	0.278078125	1
HEATR1	0.053298707	0.950032235	1
FUS	-0.445694976	0.782775428	1
S100A10	0.856697998	0.4521727	1
CMTR1	-0.388640334	0.744611795	1
THOP1	0.172765692	0.862856246	1
RALGAPB	-0.152763493	0.871695022	1
SLC23A3	0.740848213	0.498835667	1
CBS	2.000038571	0.167320905	1
NADSYN1	1.597744499	0.237679136	1
FGFR3	3.642324348	0.188241106	1
DYNLL2	0.583026939	0.610089377	1
TAF1	-0.601535812	0.730777353	1
GGNBP2	0.847773219	0.51002508	1
ENSBTAG00000024889	-0.191038586	0.853368235	1
GRB2	-0.082224032	0.932193097	1
SSB	-0.003923512	0.99688388	1
ANP32A	0.064836962	0.955416445	1
UBA2	-0.006168546	0.99456848	1

CAMK2B	-0.431544622	0.779006232	1
TASOR2	-0.078843037	0.932376991	1
SMAD4	-0.520389945	0.745753042	1
SATB1	0.464478087	0.709625469	1
PDE4A	-0.231741129	0.892282493	1
ROCK1	0.207129285	0.844576924	1
PFDN1	0.022683727	0.983923551	1
COL5A2	-0.3509975	0.722743418	1
ILF3	-0.027476504	0.977495943	1
DNAJB6	-0.48005326	0.578666644	1
MOCS2	-0.427000651	0.671050855	1
PAPOLA	0.08591557	0.930101952	1
POLR3A	0.485805956	0.662556623	1
TRAPPC9	0.578366214	0.742559563	1
SETD6	0.555406112	0.454530947	1
AK2	-0.329006972	0.722945973	1
WASF2	0.576241977	0.55612002	1
DNAJC13	0.166174876	0.88385007	1
IGBP1	-0.656113719	0.445831898	1
HDGFL2	0.131708861	0.901824962	1
MAU2	0.297782572	0.802017979	1
USP40	-0.261100831	0.803079107	1
PCSK7	-0.098500473	0.921872759	1
ADGRA3	-0.333755131	0.773480301	1
SEC24D	0.815272499	0.413346187	1
EGFR	0.400834281	0.659688771	1
PALLD	0.30025537	0.830366358	1
CAST	1.104477847	0.351637407	1
HNRNPK	0.358074514	0.838380231	1
BNIP3	-0.72654407	0.493148809	1
ADCK5	1.04868846	0.317780839	1
MGRN1	0.260556688	0.78204511	1
PREX1	1.265934321	0.41742186	1
VPS28	0.687859924	0.52050489	1
KCND1	0.327324776	0.786352806	1
WEE1	0.412903674	0.805375786	1
PRXL2B	1.279197437	0.309475594	1
NOP58	0.186213574	0.865357204	1
MED12	-0.119152193	0.942979554	1
PLOD1	0.41086911	0.641296428	1
LMO7	0.189210494	0.868621532	1
TMC4	1.42853276	0.133431784	1
ANXA4	0.362995323	0.726014484	1
LAMB1	-0.251754111	0.808624368	1
ARFGAP2	0.91003465	0.663688353	1
PHKB	0.968466553	0.498350394	1
FILIP1	-0.180670913	0.878073191	1
TRAF4	1.170892584	0.248904419	1
SMC1A	0.050102894	0.961769497	1
PTPRS	-0.082608271	0.936267916	1
ATN1	-0.325645984	0.737896219	1
NOTCH1	-0.321627031	0.73503924	1
PCYT2	0.916590372	0.433452873	1
SRSF3	-0.372465355	0.700887701	1
ITPR2	1.694446333	0.165871495	1
NPC1	0.008007766	0.994134201	1
CYHR1	-0.566604536	0.522120171	1
EIF2A	-0.347377843	0.740151859	1

IRF2BPL	-0.253787037	0.821960405	1
CFAP70	-0.25657093	0.783443112	1
GARS1	1.153739489	0.311338776	1
ARHGEF10L	1.269843974	0.362332295	1
RAB24	0.981108499	0.375524113	1
ALDH18A1	0.834771072	0.532584468	1
RNF149	1.311662449	0.108041542	1
RUFY3	-0.493621486	0.548676334	1
CAND1	1.042500561	0.374268723	1
SMARCE1	0.919504779	0.406090695	1
ZNF142	0.247141788	0.856218807	1
GGA3	-0.181687728	0.856136196	1
TOPBP1	-0.733382534	0.4508386	1
PAXBP1	0.289404605	0.778995407	1
LMF2	0.82763921	0.342629397	1
STRN3	-0.280047429	0.816058245	1
TANC1	0.506794953	0.581953601	1
SEC13	-0.461053576	0.62078082	1
CAMSAP3	1.672209667	0.124178965	1
LOC107131209	0.232283765	0.835705682	1
VIRMA	0.954005649	0.428987303	1
RIPOR1	-0.389604699	0.7107543	1
BMI1	-0.549038347	0.53720787	1
WNT10B	1.556962455	0.159226086	1
KDELR2	0.651581954	0.510645222	1
ZDHHC4	-0.194631217	0.847464636	1
PCIF1	0.566072167	0.645058607	1
CLASP2	1.061620439	0.500085231	1
NRP1	-0.793985575	0.317397937	1
LLGL2	1.292260999	0.243060488	1
CDK9	-0.273454151	0.76243329	1
PIGZ	0.164924848	0.870247061	1
GNL2	0.83927014	0.460529851	1
NPDC1	0.52020639	0.622163831	1
TRIM44	-0.800454141	0.441315531	1
RELCH	1.997254991	0.253451954	1
DLGAP4	-0.135148413	0.907433915	1
UPF2	0.567357188	0.601329237	1
AP1G2	0.858090378	0.377685766	1
DENND6B	0.864671836	0.386103441	1
UBTF	-0.486650411	0.759209144	1
AP1G1	0.154219349	0.894658206	1
MKRN1	-0.373247888	0.733390356	1
EPHB3	1.546792744	0.129452246	1
CRISPLD1	-2.848535101	0.133911412	1
SETX	-0.059479322	0.94503155	1
AARSD1	0.670152545	0.540391059	1
SFSWAP	-0.284822315	0.755083377	1
BRWD1	0.291628021	0.800617678	1
PROB1	-0.530865703	0.664617062	1
XPO4	1.368955719	0.340129183	1
ENSMMSG00000015362	0	1	1
ERCC5	0.857861323	0.491915075	1
TXNRD1	-0.484622941	0.65082544	1
MPRIP	-0.006803097	0.994874405	1
CACNB1	-0.625765637	0.671157862	1
OGFOD3	0.60906821	0.477093804	1
G18923	-0.072837925	0.954884884	1

EXT2	0.73022201	0.478060504	1
PTMS	1.052214072	0.311890781	1
MRPS7	-0.307469716	0.729438923	1
TAPBP	0.713436263	0.497476727	1
DAZAP2	0.450173167	0.694525802	1
CLIP1	-0.315837195	0.866736752	1
ACP6	1.240768702	0.327063753	1
CTCF	-0.414691297	0.667956564	1
PDAP1	-0.283384896	0.709026702	1
DICER1	-0.447426446	0.644300619	1
CCDC106	0.665728536	0.515864654	1
ARF4	0.69722506	0.49986207	1
AHCY	-0.366454366	0.747586627	1
UBP1	-0.423329639	0.655198805	1
ILF3	-0.359509366	0.837647803	1
MAPK12	-0.55560986	0.615713316	1
NUP205	0.547097607	0.638614354	1
GMCL1	0.06248622	0.951435938	1
FKBP8	-0.269344396	0.817649016	1
SUGP2	0.734474965	0.580347343	1
ACAD10	-0.153459311	0.883959749	1
PRPF31	0.250571886	0.817127122	1
MRPS34	0.131864901	0.896766273	1
NUTF2	-0.587811997	0.55190851	1
GRPEL1	-0.032712746	0.972790853	1
NOL11	0.531898007	0.620722376	1
NSD2	0.169825232	0.823422274	1
RNF40	0.112163611	0.876925662	1
LPIN3	1.236835621	0.19546627	1
TMX2	0.032369013	0.971213234	1
HOXD8	-0.014067808	0.990623396	1
UBTF	0.627187633	0.592868381	1
IMPDH2	0.095302171	0.947235004	1
SMU1	-0.284242635	0.753181628	1
PIEZO1	0.323662723	0.762574018	1
RASA1	0.597755218	0.540878651	1
VTI1B	-0.419234993	0.600337488	1
NDUFV3	-1.144630314	0.181902336	1
ENSMMSG00000009469	-0.860092333	0.326422427	1
CERS2	0.475179392	0.572808607	1
ACBD5	-0.573278608	0.616095438	1
CLNS1A	0.545278156	0.726615112	1
SH3D19	0.698021998	0.539603435	1
ENSBTAG00000047319	0.457456791	0.762322983	1
RHOQ	-0.972774473	0.280090129	1
DGAT1	0.694490523	0.562489567	1
GSTT1	1.029463432	0.305059237	1
FLOT2	-0.726437949	0.648268488	1
C1QBP	-0.464319019	0.615205189	1
MAGED1	1.797988265	0.15891357	1
PRPF40A	0.26525532	0.817663429	1
ARHGEF1	0.323057919	0.733232723	1
TAPT1	0.092495266	0.942217727	1
CTNND1	0.244832195	0.891899743	1
HERC2	-0.226764652	0.810959903	1
NHLH2	0	1	1
RHOC	0.183431137	0.841768225	1
ZYX	-0.519215636	0.565012745	1

PTPN18	-0.359875709	0.694161372	1
CC2D1A	0.260708433	0.828747875	1
ATP2B1	0.474872088	0.758624784	1
KIDINS220	1.501901428	0.526109372	1
CUL2	-0.477947444	0.646711786	1
UBXN6	-0.335749696	0.760862793	1
FBLN1	1.225709011	0.259666381	1
CNPY3	1.26789932	0.264038017	1
SERBP1	-0.511443036	0.457996405	1
TANGO2	-0.379757017	0.756951416	1
NELFB	-0.18685068	0.852284496	1
SH3BGRL2	2.306989112	0.112468938	1
PGM2	1.39590256	0.153522678	1
MRPL41	0.894092335	0.441610907	1
UBA3	-0.280463968	0.787305287	1
MARS1	0.257712166	0.766668376	1
CWF19L2	1.019059288	0.435863104	1
YARS1	0.401450818	0.662686713	1
LRRCC1	1.206846561	0.305854916	1
SRSF1	0.182075939	0.913298887	1
FZR1	0.561767162	0.571936744	1
ABCC5	-0.646044473	0.551698763	1
PABPC4	-0.812450466	0.365660753	1
PPP1R7	1.670148218	0.197981434	1
ADD1	0.197989798	0.902701985	1
UCHL5	0.093755428	0.920334891	1
POLR2L	1.138957843	0.324091548	1
ARHGEF10L	-1.087555489	0.300666034	1
DNMT1	0.919761946	0.411491843	1
FLAD1	0.089489145	0.922366627	1
BBS2	2.058089357	0.149436927	1
FBXO9	-0.217046742	0.82638899	1
RPS8	0.379567872	0.686750233	1
YME1L1	-0.254317225	0.882991255	1
TRMT2A	-0.26911686	0.804145303	1
NDUFB4	-0.521228816	0.623860797	1
RNF181	0.55720646	0.590504423	1
HINT2	1.19737408	0.371435148	1
POLR2C	-0.446338268	0.560385382	1
ASPH	-0.769056839	0.459332707	1
ZBTB18	-0.917264394	0.431915319	1
RPL7L1	-0.459919756	0.603193598	1
NCBP1	-0.066406648	0.945024107	1
HEXB	1.48007846	0.185335595	1
PSMG3	0.776187571	0.56079587	1
KIAA0100	-0.207832375	0.819640234	1
CLEC3B	0.656097331	0.576779694	1
FBXL5	-0.645782845	0.691706359	1
TRAPPC12	0.596328567	0.583416614	1
AUTS2	0.533489285	0.841755288	1
TXLNA	2.182480029	0.10866758	1
PPA1	-0.931908221	0.317809475	1
SIN3A	1.007774854	0.432410194	1
PTPRB	-0.782913438	0.324335858	1
RABGEF1	0.304468416	0.758337266	1
NAGA	1.380406613	0.15316198	1
DHRS7B	0.438266884	0.657172926	1
TNC	2.113369122	0.188946948	1

MRPL24	-0.052109118	0.96181975	1
FAM107A	2.052577678	0.168796245	1
DYM	-0.535667064	0.743730228	1
KDM3B	0.529044282	0.686043773	1
GYPC	-1.240674621	0.209997855	1
MBD2	-0.235863602	0.800373594	1
PCMTD2	0.486569745	0.707417189	1
HTT	0	1	1
LOC781741/BAG1	0.766785099	0.573634114	1
SRSF4	0.312030873	0.848684201	1
TOR1A	-0.023079902	0.972862892	1
NEDD4L	0.328554523	0.795820608	1
RIOK3	0.985749253	0.434163793	1
PCYOX1	0.130996151	0.901550894	1
FHIP2B	-0.369467962	0.750995976	1
INPP5B	1.162166171	0.296050989	1
TACC2	-0.6856248	0.665509102	1
GAA	-0.094249376	0.920006226	1
COL4A2	-0.644983541	0.404621463	1
ZBTB40	0.091224977	0.939424459	1
IER2	0.106638494	0.911352503	1
ANK2	0.656616865	0.874744774	1
CAPN7	-0.450583777	0.783959573	1
DMKN	0	1	1
CISD1	-0.856572104	0.362847741	1
GLO1	-0.805722219	0.40147961	1
HMGB1	-0.153819221	0.879920916	1
SNTA1	-0.704924861	0.550688077	1
COG4	-0.21495799	0.810920672	1
IRF2BP2	-0.724217507	0.426942044	1
UBE2J1	-0.428087988	0.686292657	1
GIGYF1	1.054855204	0.453565069	1
SIRT5	-0.518008884	0.612459292	1
MAST4	-0.04571264	0.96512806	1
FLT1	-0.399373243	0.645051508	1
GUK1	0.449934171	0.678500568	1
ABHD4	-0.256645001	0.877773717	1
NEK4	-0.378918073	0.714085259	1
ATP6V0E1	0.577052805	0.588080658	1
GART	-0.02158886	0.982374037	1
PIGT	0.339211762	0.722723113	1
STK25	-0.296031824	0.728903464	1
SPRYD3	0.743072217	0.491144597	1
VSIG10/WSB2	-0.736203207	0.539849738	1
ERH	-0.519085891	0.613356679	1
BCCIP	-0.425612276	0.60794132	1
FHOD3	0.249495781	0.857488595	1
SMS	-0.351134583	0.732625443	1
ZC3HC1	0.447660714	0.742872908	1
NDUFA7	0.241497241	0.838639353	1
NF2	-0.669120527	0.406552181	1
AK3	0.779265988	0.569228531	1
DHPS	-0.628101521	0.549383784	1
SVEP1	-0.09867649	0.918700203	1
USP48	0.677679963	0.571684911	1
DPP9	-0.671159959	0.426908	1
SCN1B	-0.794217994	0.604431578	1
VAC14	0.066990565	0.951449018	1

CLPP	0.308056508	0.778550801	1
GHR	-0.969388066	0.527059542	1
RUSC2	0.591864942	0.662554689	1
RANBP9	-0.55530084	0.585108359	1
C23H6ORF62	0.361009102	0.782396989	1
CLN3	0.826665734	0.332835936	1
VPS11	0.146749196	0.911291549	1
HR	0.138084529	0.908177628	1
RAB1A	0.271245768	0.776651365	1
RSL1D1	-0.005985048	0.99578601	1
DBT	2.33063291	0.186918512	1
TMEM238	0	1	1
HMCN2	-0.035254576	0.977872908	1
ATP6V0A1	0.378236212	0.81738274	1
STK11IP	1.108313307	0.374133369	1
SMPD1	0.456456216	0.628415273	1
KY	-0.999433355	0.569313887	1
MRPL14	1.143360032	0.423260902	1
ZNF335	0.055032783	0.958070002	1
SNX9	-0.334906609	0.782282797	1
NSD3	0.513845938	0.611797794	1
XPC	0.447521426	0.665557307	1
ESYT2	0.268284565	0.740880632	1
ABCD3	0.321006087	0.751070167	1
PHF3	0.184641276	0.878537182	1
SEPTIN2	0.220730197	0.821852079	1
DCAF1	-0.160571902	0.876074643	1
OBSL1	1.764515759	0.191977351	1
ZNF341	1.827174224	0.117664717	1
ATG3	-0.117279548	0.895948891	1
XPO7	0.00378222	0.997141596	1
FARSA	-0.432993608	0.617266538	1
SBNO2	0.194810936	0.858660777	1
SNW1	-0.089195552	0.937186086	1
RBM14	0.095609091	0.927207671	1
RNASET2	1.323695642	0.180223889	1
ATP6V0A2	0.690795763	0.492508129	1
ABHD16A	-0.135443531	0.881560277	1
TBCD	1.000677122	0.413650101	1
DST	1.056239776	0.384912877	1
SPTLC1	1.138031245	0.303967052	1
CLIC5	-0.251494546	0.87981749	1
FYTTD1	0.634500548	0.639136975	1
PTPRA	0.502167409	0.600106176	1
VPS13B	0.471397655	0.676127927	1
EIF4A3	-0.095352932	0.924039993	1
DGCR6L	0.271309918	0.812103491	1
TAOK2	-0.09845999	0.921448195	1
SEC31A	0.823377719	0.414439782	1
TOB2	-0.209560831	0.839617941	1
RNF11	0.02426493	0.984675501	1
PIGO	0.144142768	0.876871166	1
PPP2R5A	-0.518408903	0.602610481	1
UTP20	0.3052173	0.726702337	1
ITSN2	0.359926472	0.636003214	1
PPIL2	0.279593091	0.791599896	1
EZH1	0.428576405	0.758638944	1
HIVEP1	-0.405341734	0.749418869	1

SNX14	2.10269009	0.234025662	1
UBL7	-0.045613658	0.966205201	1
COX7A2	1.458254553	0.147762769	1
SMARCD2	0.755970342	0.349767372	1
MAPRE3	-0.838525443	0.396650012	1
SUOX	-0.342683492	0.83534288	1
DBH	0	1	1
SRSF2	-0.077309271	0.936413535	1
ABCC1	-0.27974186	0.782342612	1
ENSMMSG00000000337	0.235882294	0.833906364	1
ECD	0.922156698	0.494777061	1
RXRA	-0.772330518	0.479509909	1
MRPL55	0.035875972	0.97495526	1
OGDHL	-1.828505416	0.412255403	1
KIAA0232	-0.746785042	0.544957513	1
RNF167	0.678199397	0.496441579	1
MYO10	-0.903819899	0.42612506	1
ZC3H15	-0.147783631	0.880907616	1
RBM20	-1.014654027	0.37963782	1
UTP25	0.044183212	0.9672946	1
KDM2A	0.525857741	0.634659665	1
NAA20	-0.57124393	0.543104066	1
TMEM59	1.081418362	0.388689549	1
MESD	1.447139684	0.253885063	1
MTREX	0.257000408	0.808241294	1
ACTR2	0.457179119	0.635825048	1
WASHC1	-0.204694363	0.863114415	1
METAP1	-0.520062156	0.59552848	1
UBE2L3	-0.090185849	0.930473286	1
DHRS4	0.286269627	0.827081107	1
NEK1	0.276530494	0.802927078	1
SGPL1	1.688505822	0.099344894	1
DAXX	-0.270710565	0.786346984	1
SPG21	1.643801712	0.168066852	1
CCM2	-0.489904019	0.565564327	1
SNX2	-0.281389611	0.769344655	1
USP28	-0.081158847	0.950890435	1
SEC16A	1.415101659	0.317539065	1
TPM4	0.217828875	0.818073333	1
NCOR2	0.796294284	0.457842873	1
CRKL	-0.560471503	0.602071634	1
HIP1	0.586910887	0.627878122	1
SMYD2	-1.001155676	0.231455558	1
EGR1	-0.855363972	0.382445026	1
DDX50	0.340353972	0.715831915	1
DAZAP1	-0.01312178	0.98956194	1
HNRNPM	-0.388130082	0.670684654	1
TMCO1	0.821113888	0.397510518	1
ATP6V1C1	-0.261704425	0.775430114	1
PCYT2	0.237568148	0.889202984	1
KAT6A	-0.402793971	0.659649439	1
PCM1	-0.210999064	0.837969969	1
CHD7	-0.325831899	0.718696638	1
ASB11	-1.016481958	0.343620685	1
VARS1	0.380184404	0.819640993	1
ZDHHC3	1.031009848	0.21524272	1
NPEPL1	-0.096707481	0.940013715	1
SLMAP	0.160282313	0.912442417	1

MTOR	-0.226081306	0.826195684	1
WRNIP1	0.259064435	0.845269426	1
DYNC1LI2	0.860795207	0.518906596	1
SLC43A1	1.517031647	0.214425284	1
ARPC1A	1.540450567	0.255628487	1
DSG1	0	1	1
CHORDC1	-0.455067015	0.671933849	1
CPT2	-0.018282639	0.986210088	1
ADGRG1	0.30616887	0.766780584	1
ABLIM1	0.357603546	0.804875281	1
USP2	-0.814025547	0.396591402	1
EMC4	-0.763894347	0.354208898	1
ENSMMSG00000020369	-0.655774652	0.603160999	1
PSEN2	1.381667376	0.410721185	1
SORT1	1.190227951	0.363255922	1
MAPRE2	-0.739599508	0.518736582	1
TLE2	0.859583893	0.443256564	1
MTMR4	0.546861709	0.661418652	1
MAVS	-0.320714094	0.748063138	1
COL4A2	-0.482559496	0.563167444	1
G21918	0.954036196	0.398225915	1
EIF3J	0.555759061	0.638706517	1
ENO1	-0.233308176	0.877870529	1
PVALB	-4.32712138	0.29880664	1
DHRS3	-0.623977097	0.526019973	1
RAB3GAP1	0.037954078	0.971562641	1
PRMT5	-0.055877309	0.958856579	1
EEF2K	-0.678009882	0.493515267	1
FXR2	-0.901082407	0.578897041	1
WDR82	-0.115551244	0.915592365	1
PPP2R3A	0.304989431	0.830107126	1
FOXK2	-0.146287597	0.900383171	1
STK11	-0.658558056	0.561004397	1
ANO6	-0.483611306	0.671394839	1
POLR2E	-0.151544095	0.884355397	1
MAGED2	1.777276002	0.192192183	1
SLC20A1	0.395010809	0.714421588	1
HK1	-0.081798772	0.935287346	1
PPP3R1	-0.33528929	0.847449997	1
RAC1	0.466106602	0.63593794	1
AGPAT3	-0.348943402	0.777220391	1
EMP2	0.695060605	0.520901473	1
NOL3	-0.422733265	0.747248501	1
PAPOLA	-0.275909841	0.758646884	1
LAMA4	-0.467901217	0.620768891	1
CHTOP	0.488748316	0.713532163	1
NDUFS4	-0.533766791	0.594155981	1
GNAI2	-0.058962804	0.953466156	1
PTPN11	0.027898415	0.980721852	1
NDFIP1	0.843432958	0.517180564	1
AMPD3	0.521294585	0.774881133	1
URGCP	-0.803100493	0.524596245	1
DIS3L	-0.548750577	0.583167037	1
WASHC5	0.571520432	0.559281054	1
MFAP5	-0.742341477	0.461210634	1
SIL1	1.213482213	0.235076446	1
NVL	0.248538279	0.806579229	1
GRIPAP1	-0.209669635	0.8358047	1

TIMM50	0.052943273	0.952979427	1
MICU1	0.398644946	0.825620318	1
ATP13A1	0.486715618	0.656815378	1
WDR33	-0.180365329	0.818150238	1
NABP1	1.747873092	0.117342736	1
CALCOCO1	0.213483377	0.853036473	1
SDR39U1	0.091284378	0.941252639	1
PSME1	1.44728679	0.254550775	1
TM9SF4	0.605049132	0.491032167	1
TJP1	0.359017491	0.725649558	1
JPH2	-0.533759288	0.747536538	1
WIPI1	0.193872862	0.885722841	1
UBE2M	-0.636354472	0.438015471	1
ARSA	1.453877606	0.092677314	1
BAZ2B	0.753838366	0.834869086	1
NID1	-0.653424301	0.51957467	1
ME1	1.899278237	0.131588072	1
ZMYND11	-0.311767779	0.747722411	1
KCTD3	-0.492802228	0.638222981	1
PICALM	-0.108571263	0.947208074	1
SYVN1	0.793112924	0.418213863	1
MED25	0.85169804	0.42793223	1
CCPG1	0.145840714	0.879922697	1
NUP153	-0.312158016	0.779535575	1
SEMA6C	-0.956676748	0.426843306	1
ACADS	-0.560156975	0.57811493	1
NDRG2	1.598687943	0.201414267	1
PPM1B	0.347581167	0.846929019	1
NDUFA6	-0.669040724	0.52853813	1
COQ10A	-1.196735	0.421040261	1
PPP6R3	-0.504742733	0.608800374	1
PHPT1	0.235062841	0.805021887	1
AKT2	-0.260514087	0.799994739	1
LRCH4	-0.133813992	0.902363743	1
CHMP5	-0.001531688	0.998709344	1
TMEM39B	-0.151478295	0.87290669	1
SH3YL1	0.331938401	0.736257924	1
PATL1	0.054079916	0.958727829	1
TRMT1L	0.109250554	0.929201639	1
COG8	1.176828913	0.38031118	1
GADD45GIP1	1.496175875	0.294989589	1
KMT2C	0.172556564	0.874164041	1
PGP	-0.334109767	0.76484958	1
PSMD14	-0.301121177	0.725454968	1
H2AC18	1.467396919	0.189685893	1
NDUFA11	-0.681309975	0.485809276	1
TTC4	0.126911803	0.914181316	1
RNASE4	0	1	1
PPP1R10	1.180808471	0.340733224	1
CNOT1	-0.104738532	0.951320964	1
MLF2	-0.334509317	0.734696832	1
MPST	-0.019745721	0.985948313	1
SNX19	-0.556313208	0.558925229	1
SDC4	1.929079499	0.15250723	1
GOLGA4	-1.057488392	0.313920058	1
PSKH1	-0.823636129	0.617344446	1
SLC25A39	-0.098059621	0.920902263	1
ABCE1	-0.435580437	0.644545348	1

TGM1	1.171714725	0.382904963	1
CNN1	1.329116118	0.128364582	1
ENTPD4	0.515408833	0.574678926	1
SUGT1	-0.486409856	0.647963734	1
SNX3	-0.670232904	0.475080977	1
SND1	0.675376789	0.5243356	1
NOP2	-0.029609916	0.973854325	1
PCDH20	0	1	1
FGFR1	0.264344474	0.72175431	1
ACTR10	-0.086223552	0.926345538	1
NR2C1	-0.733967578	0.445882607	1
TPT1	-0.665925769	0.535099921	1
GTPBP4	-0.150886417	0.879149962	1
LINGO1	-1.311963282	0.248452541	1
CHKB	0.313191702	0.748654898	1
USP14	0.187570138	0.873733807	1
EWSR1	-0.257014439	0.873854123	1
POLR1A	0.000968427	0.999080274	1
MARF1	-0.270754685	0.81942363	1
PSMC6	-0.277835168	0.821418616	1
PPP1R3A	-0.976762877	0.477514075	1
IAH1	0.545083506	0.539973077	1
ANXA11	-0.293042249	0.766816424	1
RHOT2	0.571485566	0.510145067	1
RBCK1	0.370131886	0.782133845	1
EHMT2	0.206690704	0.827809735	1
PAF1	-0.161937085	0.833996024	1
SULF2	0.342390648	0.771327858	1
ENSMMSG00000003352	0.672670382	0.583832426	1
ADIPOR1	0.599219353	0.635806665	1
AHDC1	-0.458142392	0.666601429	1
TST	0.92909233	0.35461441	1
MYO6	1.517268434	0.115487972	1
DGKZ	0.108199488	0.953184349	1
ANKZF1	0.15330976	0.871719133	1
RABGAP1	-0.281652968	0.780965155	1
ANKHD1	0.289730837	0.898165112	1
PES1	0.042385721	0.969012728	1
ANXA11	-0.350419762	0.759301276	1
PUF60	0.451709898	0.741077912	1
TM7SF3	1.546537207	0.183597112	1
ECE1	-0.652625709	0.498674175	1
TBC1D15	0.243040318	0.811677166	1
DYNLL1	-0.351479624	0.76901685	1
BAIAP2	0.882334886	0.3824148	1
DDX6	0.358088892	0.744566995	1
WDR1	0.413975494	0.821166499	1
KPNA1	-0.600128068	0.493207252	1
PRRC2C	0.317996546	0.779734039	1
NANS	1.164929545	0.255758398	1
RASSF3	0.179360941	0.869882131	1
ENSMMSG00000023622	1.401162486	0.11742298	1
RPS16	1.432607419	0.23306919	1
TMBIM1	-0.238061155	0.81115877	1
LMTK2	-0.378041038	0.748055465	1
CRIM1	0.201707341	0.906375974	1
SERINC2	0.111182756	0.933708752	1
DVL1	-1.004961326	0.505811457	1

QRICH1	-0.256146468	0.803071904	1
DNM1L	0.358572202	0.745735141	1
DENND2B	0.314153414	0.787651658	1
AP2A1	0.603656883	0.647885689	1
TMCC1	-0.46024957	0.654982261	1
EWSR1	0.306325662	0.81016948	1
ATOX1	1.895693593	0.098420252	1
PTOV1	1.861677854	0.176656286	1
M6PR	-0.043047292	0.978334351	1
SMG7	-0.485291456	0.614963683	1
DYRK1B	-0.758303399	0.49325028	1
LAMA2	-0.48534602	0.600984783	1
SCAMP3	-0.604500469	0.512124062	1
PRR12	0.782786428	0.480047729	1
CIZ1	-0.487531151	0.529448493	1
OLA1	-0.045290978	0.966177643	1
NEK9	-0.661938743	0.43166522	1
FCGRT	0.166230109	0.844181897	1
SLC38A10	0.602375623	0.540890744	1
ILF2	0.476415674	0.701055136	1
CIRBP	1.001577755	0.450499417	1
TPRG1L	0.058452051	0.964372982	1
TUBGCP3	-0.090018516	0.938480203	1
UFC1	0.052286605	0.955703841	1
PLCD4	-0.960671672	0.516159559	1
SNRPD3	0.593448228	0.580602819	1
BTBD2	0.556171362	0.689848256	1
PPP4R3B	0.609272774	0.593412375	1
TESK1	-0.31832616	0.751485337	1
TMBIM4	1.150683745	0.361524857	1
COPS6	-0.12167555	0.897355652	1
DBNDD2	-0.669803753	0.611137405	1
MTCH1	0.845585989	0.527967618	1
DNAJC2	-0.513389502	0.595653512	1
UBE2D3	0.432795189	0.692969015	1
PLIN3	1.741664333	0.194487076	1
UBE2Q1	-0.280013712	0.77900762	1
UBN1	-0.554463081	0.548381178	1
ETNPPL	0.265705536	0.830861545	1
PTPRM	-0.612121231	0.534814735	1
MAN2C1	1.078282642	0.391300916	1
CCDC9	0.879241313	0.529152812	1
PPFIA4	2.957546835	0.28886721	1
GPT	1.471960689	0.255881815	1
MAST2	0.293208049	0.835969244	1
UBQLN2	0.373067928	0.83314856	1
KTN1	-0.097456767	0.935923916	1
MSRB3	-0.109700406	0.919893388	1
RABL6	0.062398542	0.951769486	1
SLC6A8	-0.584611929	0.597286297	1
RNF130	-0.37653741	0.673594191	1
WWP2	0.719190242	0.350236337	1
ATP6V1B2	-0.187974443	0.836000136	1
CRYL1	1.035424344	0.294660253	1
EXOC7	1.268802625	0.341197392	1
GSK3A	-0.391676547	0.630343554	1
PPP1R3C	-1.106739274	0.496169105	1
NSMCE1	1.154434323	0.385910654	1

UBA1	-0.151395349	0.906039324	1
ATG9A	-0.851914573	0.434447397	1
VPS33A	0.130442454	0.894076801	1
FGFR1	0.199094855	0.857624876	1
DLG5	1.249824858	0.145436738	1
RAD50	0.243773023	0.808505776	1
DOCK8	1.386077241	0.375804741	1
BHLHE40	-0.384187396	0.813181956	1
BBLN	1.985684078	0.096647212	1
CTXN1/TIMM44	-0.870140987	0.335626608	1
SLC26A11	1.541610867	0.118196609	1
PSMA4	-0.264173854	0.775136729	1
FSD2	-0.611863134	0.618766883	1
RBM39	-0.107080536	0.947831748	1
ISCA1	-0.961969163	0.320842036	1
TOLLIP	0.409062822	0.740387648	1
KDR	-0.3510443	0.720159669	1
DDX42	1.116938969	0.418960041	1
DCTN4	0.079369355	0.963920061	1
GPS1	-0.455430468	0.622329667	1
POLR3D	0.35050778	0.795843668	1
MFGE8	-0.780914669	0.375240256	1
USP32	1.407559781	0.358781772	1
TNS2	-0.141541512	0.906366495	1
SNU13	0.132551291	0.907174515	1
USP10	-0.350370518	0.735626712	1
ACTR1A	-0.319346363	0.69758922	1
CARS2	1.216418959	0.358524579	1
FAF1	-0.626985986	0.524445406	1
PHF2	0.132452752	0.895986391	1
SMARCC2	0.093739408	0.933167072	1
C4BPB	0	1	1
ORMDL3	0.735436149	0.651581782	1
MRPL37	-0.371364178	0.695771464	1
RPAP1	-0.480444106	0.653843697	1
ERLEC1	0.004212765	0.996033204	1
DOCK6	0.989550277	0.593411497	1
SRP54	0.237902005	0.836526072	1
FNTA	-0.603021238	0.542018115	1
MCM7	0.252450937	0.813992974	1
MGAT4B	-0.108727988	0.902938594	1
CTSV	0.007035919	0.994250441	1
PDLIM1	0.696272319	0.624720726	1
TMEM91	-1.826628679	0.426730743	1
PCOLCE	-0.114607091	0.907782671	1
CAPZA1	-0.108239851	0.917916899	1
MEF2C	-0.170687444	0.914104312	1
PSMD5	0.173629241	0.878739989	1
EDC4	-0.121740033	0.919094393	1
RAD23A	-0.53609098	0.472128701	1
PJA2	0.114766506	0.868101131	1
SSR3	0.5919978	0.592148772	1
MVP	0.219124603	0.821621723	1
VEGFA	-0.700089882	0.501886362	1
ILKAP	1.45432147	0.159973729	1
TSPAN3	0.555857094	0.698997305	1
SLC29A1	0.778571881	0.554187664	1
EPB41	1.205227708	0.466257734	1

EFL1	-0.377981845	0.693023333	1
BUD23	0.246179278	0.771193314	1
SELENOO	1.056294382	0.398253743	1
SLC35B1	-0.134881297	0.875745109	1
TBC1D10B	-0.832600181	0.455359911	1
NRTN	0	1	1
KBTBD12	-0.804374867	0.438265277	1
KLHDC2	-0.52091094	0.647196654	1
ACSL4	-1.075927237	0.246972778	1
RUVBL1	-0.273661357	0.767596306	1
USP34	-0.539327448	0.742751759	1
C1R	1.009820711	0.383024254	1
SH3RF2	0.444205388	0.734323443	1
SLC25A19	-0.324404298	0.740375285	1
DHX15	-0.384068453	0.694225792	1
OSBPL2	0.266255392	0.775326599	1
TACC1	1.857584119	0.153636733	1
WAPL	0.11754641	0.915438153	1
PACSIN2	0.329268798	0.746657348	1
PPP1R12B	-0.280532379	0.879668626	1
HSF1	-0.395518031	0.721311434	1
GABARAPL1	1.407569958	0.32775195	1
HHATL	-0.228118287	0.877515944	1
CHPT1	-0.497396403	0.564571778	1
BZW1	0.815032279	0.61250865	1
PRPSAP1	1.009865589	0.284266965	1
TXNRD2	0.176933985	0.874373717	1
ARHGEF7	-0.138345891	0.879209212	1
GON4L	-0.146493538	0.857289306	1
ACO1	-0.501461422	0.53253484	1
ZFR	0.15922002	0.898126747	1
ELOA	-0.183880461	0.85272245	1
PRPF19	-0.116007047	0.906580502	1
COQ3	-0.888614864	0.312289868	1
EIF2B5	-0.453394015	0.574641484	1
GPC1	-0.255910636	0.847320631	1
NAA50	-0.027768467	0.980048705	1
PITX2	1.813553854	0.192695068	1
INTS10	0.25732354	0.799410542	1
GNS	0.952271765	0.344194725	1
ENSMMSG00000005501	0.248012079	0.803191817	1
KPNA3	-0.829675857	0.597611437	1
LMO7	-0.958337145	0.60762879	1
EMG1	-0.116915936	0.899785246	1
ARPC3	0.949422304	0.394152475	1
BRD3	-0.015472652	0.985064142	1
MLEC	1.518734231	0.130832308	1
FAM21A	-0.258981434	0.799717238	1
CRNKL1	0.118297747	0.901769794	1
ADNP	-0.253541268	0.798949735	1
LRRC41	-0.016472668	0.987044107	1
EMC7	-0.042681374	0.960443178	1
DNAJC11	-0.718124641	0.479834855	1
H3-3A	0.342169433	0.625002249	1
RTCA	0.944721138	0.429103754	1
MIB1	0.611132165	0.568376224	1
CNOT3	0.887806001	0.310376365	1
ATRAID	0.142806781	0.869936432	1

ABCC9	-0.133675533	0.937534539	1
AGPAT2	-0.292730031	0.768157684	1
TBX15	-0.862975489	0.392839599	1
RRAGA	-0.290359663	0.786873681	1
NONO	0.269883458	0.774568769	1
EIF2S3	-0.370192931	0.664789741	1
CCN1	-1.044502017	0.261734467	1
ALDH4A1	-0.080492174	0.947557245	1
KIF12/AMBP	0	1	1
CTSF	-0.01091162	0.991670937	1
L3MBTL1	0.31232792	0.796655245	1
PITRM1	-0.335245297	0.704308574	1
ATG2A	0.084830105	0.933233955	1
RING1	-0.012489412	0.990110276	1
AIMP1	0.529633894	0.676910961	1
PABPN1	0.708258977	0.477035131	1
TACC1	0.87449797	0.396510631	1
ATL2	-0.513955004	0.618832743	1
MAGT1	0.827492634	0.458577355	1
PGD	0.468059835	0.599171826	1
ARNT	0.177092124	0.850577356	1
RBM6	0.652009383	0.571208066	1
AP2S1	0.494287788	0.658631685	1
WDR1	1.01625552	0.443846347	1
NPC2	0.631304041	0.554188769	1
COX6A1	1.618623627	0.107614465	1
DARS1	-0.516382369	0.615345301	1
MRPS33	-0.502118103	0.629375364	1
R3HDM2	-0.411005073	0.80365051	1
MTDH	-0.12648542	0.899107108	1
PRXL2A	1.831274126	0.213689574	1
SRP14	-0.630692045	0.435607616	1
R3HDM2	0.405320942	0.822546054	1
TNC	1.434384883	0.400941093	1
ATRIP	1.26427632	0.2385182	1
TUBGCP6	0.723183619	0.484545805	1
MN1	-1.010821706	0.392388355	1
FRY	0.086761178	0.940577691	1
HK2	-0.916779954	0.420736602	1
PRR7	-2.848473395	0.132913137	1
CAVIN4	-0.78582265	0.640345639	1
HID1	1.306727766	0.395192151	1
CAB39	-0.347377792	0.687885696	1
TAF6	0.558666759	0.562432398	1
WDR26	-0.423403405	0.653917581	1
UCP3	-1.420187957	0.280191382	1
RBBP4	0.868979966	0.462029715	1
TARS2	0.072007796	0.939930799	1
ENSMMSG00000017087	-0.427195012	0.681319545	1
NIPBL	0.176023554	0.878470072	1
SSU72	-0.110965116	0.907705093	1
PC	1.292815536	0.177741092	1
PSMA5	-0.309683108	0.724740724	1
AP3B1	0.015701404	0.98812673	1
CLIC1	1.237480814	0.220767554	1
TIMP2	0.542065792	0.648688414	1
HTATIP2	0.190918358	0.836312017	1
RCC1L	0.309473042	0.798068857	1

HIBADH	-0.605073871	0.515267178	1
NOC2L	0.346332649	0.73079216	1
COASY	0.657631117	0.507157227	1
AP2B1	-0.152795692	0.878784978	1
CORO1B	1.369253863	0.197336199	1
STOML2	-0.201453818	0.84007045	1
CLK4	0.912427966	0.486351503	1
YIPF3	0.390819111	0.687448422	1
EHHADH	1.325557971	0.420861645	1
ANKRD11	-0.380024701	0.866795711	1
ARNTL	0.029206648	0.989591638	1
SSH2	0.001124009	0.999272908	1
SCAP	0.217086363	0.92670214	1
FXYD1	-0.270124867	0.835907826	1
OSBP	-0.38459201	0.711141923	1
KIF19	1.536004858	0.207001491	1
NEK10	0	1	1
SEC24C	-0.207942348	0.825675357	1
ERCC3	-0.207384615	0.862379261	1
RAD54L2	1.179607457	0.295835066	1
NUP98	-0.242975612	0.832797076	1
CACNA2D1	0.155935571	0.929142137	1
IDH3B	0.356115861	0.688207051	1
SLC4A2	0.934769163	0.34659711	1
IDH3A	-1.074302156	0.278680729	1
TINCR	0	1	1
BOLA	-0.491655663	0.608107258	1
GNB2	0.231771484	0.805125012	1
GAS6	0.751178579	0.443618514	1
PICALM	0.093392597	0.936230453	1
YWHAB	0.453594326	0.63958283	1
HAGH	-0.61683631	0.472191574	1
SKIV2L	0.262105581	0.816949398	1
GET4	-0.572991556	0.738250645	1
ANKRD22	0	1	1
ATP11B	1.061447134	0.282774329	1
RNF20	0.020142696	0.984761787	1
HGS	0.110802717	0.922047473	1
PPID	-0.286189359	0.70500262	1
ENG	-0.623222973	0.36471081	1
HM13	1.016016487	0.218967845	1
ILVBL	-0.187748941	0.849299122	1
TBRG4	-0.598619388	0.573720704	1
SSR4	1.506328659	0.204656535	1
ZMYM2	0.27047868	0.815183737	1
SEC24B	-0.163341083	0.875678993	1
TTLL12	0.024111314	0.975628395	1
STAG2	-0.026246989	0.981429534	1
SACS	0.377664581	0.841635892	1
PEX19	0.077230437	0.945703509	1
MIB2	0.257994904	0.836913792	1
LOC515042	-0.076222224	0.937217134	1
SLC37A3	2.222500905	0.11640026	1
FBH1	-0.109057345	0.918522747	1
POLG	-0.481913896	0.656284154	1
CLIP1	-0.653717973	0.638543092	1
COG3	0.75126041	0.481542726	1
STAT3	-0.134655724	0.88690496	1

RBM34	-0.349449989	0.722200325	1
VPS16	0.46936134	0.600699171	1
ITPRID2	-0.288404074	0.822826015	1
LZTR1	0.464696339	0.577899831	1
GDPD3	-0.365339351	0.721202717	1
SERINC3	1.132846144	0.365072716	1
HSD17B10	-0.308953149	0.751965709	1
DPP8	0.178861587	0.889673316	1
PPP1R12A	0.29734132	0.803199651	1
FERMT2	-0.476248221	0.620924812	1
PDE8A	-0.833129523	0.618098274	1
CLPTM1L	0.063732683	0.949662025	1
MAP1LC3B	-0.703211876	0.515895911	1
NDOR1	1.326902398	0.232292302	1
EMC2	0.565217597	0.7463719	1
SELENOW	-0.603575372	0.58430095	1
HACL1	1.332266044	0.311726044	1
SON	-0.195281247	0.838615599	1
ROCK2	-0.162794535	0.87294574	1
COL1A1	0.073692287	0.952275301	1
ANXA7	-0.278212463	0.760874123	1
TBC1D9B	-0.37975919	0.708250076	1
AP2A2	-0.189606478	0.859944412	1
UBE3A	-0.295176603	0.773207484	1
SLCO1A2	0	1	1
IST1	0.856460688	0.503697051	1
XPNPEP3	-0.165650127	0.869578784	1
LRRFIP2	-0.313313542	0.796533439	1
DDIT4	-0.274170369	0.818126549	1
PRELID1	1.467248184	0.092482433	1
MCOLN1	0.268881649	0.793721479	1
SH3GLB1	-0.794319327	0.373686364	1
NCKIPSD	0.615572587	0.549149669	1
UBE3C	-0.437964573	0.68482682	1
PCDH1	0.901846878	0.407909138	1
SNX5	0.741223223	0.483427796	1
TMEM63B	1.368747311	0.311458555	1
NAXE	0.439478165	0.635190118	1
NRG1	0	1	1
TNPO3	-0.506830816	0.471837971	1
PABPC4	0.179516381	0.888579974	1
CD36	-0.767516852	0.482297266	1
PTCD3	0.743643163	0.577336355	1
HBP1	0.784478684	0.528475917	1
LRBA	0.754119703	0.500639858	1
GLYR1	-0.528925006	0.51558857	1
GGA1	0.495231256	0.710071545	1
RARS1	-0.226446997	0.827666408	1
GOLGA3	-0.048032657	0.964907338	1
RABAC1	0.270726278	0.809635192	1
CSNK2A1	-0.490374077	0.608049789	1
ALS2CL	0.461939586	0.639015833	1
MYCBP2	0.987121595	0.425919797	1
NDUFB6	-0.672117548	0.511748111	1
DDX41	0.112196197	0.923852869	1
CD2AP	1.611069596	0.119177697	1
GTF3C2	0.210580632	0.822522947	1
CEBPZ	0.291662579	0.785289979	1

CCDC180	4.36173712	0.084898225	1
MEA1	-0.009964955	0.990045427	1
CTTN	0.036999242	0.960867853	1
NRBP1	-0.487247675	0.563741794	1
HNRNPM	-0.18906053	0.855409124	1
GNPTG	0.816091897	0.430598879	1
SURF4	0.267921084	0.796451148	1
PSMA1	-0.175144316	0.847383187	1
PTGES2	-0.696104909	0.488829365	1
ULK1	-0.80846888	0.419528035	1
PHF1	0.668145419	0.611844947	1
DDX54	-0.296835326	0.780078057	1
HMGCS1	1.159730635	0.239810593	1
GSTK1	0.607043336	0.629729659	1
MLIP	-1.531723726	0.361245079	1
OTUD7B	-0.075859081	0.955676721	1
NOP56	0.79594318	0.482857582	1
ARHGEF28	1.860103227	0.203085359	1
EHD2	-0.752449631	0.428654827	1
RAB10	-0.145697649	0.873331781	1
UAP1L1	0.749071972	0.536134659	1
THRA	-0.67144911	0.546502468	1
ADSL	-0.77215766	0.406639175	1
ARFGEF3	2.365922669	0.258199171	1
NMRAL1	0.85791614	0.369731305	1
GIGYF2	-0.156551139	0.876157446	1
TMCO3	-0.152654093	0.886617499	1
BCAM	0.177918172	0.863783117	1
ETV4	3.754475192	0.135408482	1
CLCN1	-0.945065336	0.520859164	1
NSMF	1.094024851	0.347377486	1
MADD	0.771774944	0.641876801	1
NR1H3	-0.350385272	0.732567865	1
SMARCC2	-0.286500434	0.863060041	1
EMP1	2.046243834	0.104162035	1
LAMP2	1.492273558	0.201095342	1
TRAF7	1.282403841	0.350321823	1
DHRS1	1.07766766	0.155246477	1
TRRAP	0.436167693	0.725511694	1
AOX1	0.312393514	0.739000711	1
TRRAP	1.292711312	0.488826669	1
SYNGR2	-0.387326135	0.714383846	1
ABLIM1	-1.098919333	0.535685059	1
HUWE1	-0.550130676	0.747239245	1
CARM1	-0.697040507	0.427848148	1
MACROD1	0.934840947	0.502322822	1
LRPAP1	0.398304004	0.70923876	1
VPS4A	-0.569635865	0.630721728	1
CUX1	0.186711707	0.917139225	1
ANKRD42	0.17593564	0.875668488	1
CDK11B	-0.287969567	0.76384468	1
ANGPTL4	-0.284902136	0.751276258	1
C6H4ORF54	-0.354083643	0.838566335	1
ESRRA	-0.756666515	0.519490414	1
RTF1	0.07504398	0.940802129	1
IGF1R	1.960851707	0.117273312	1
ZDHHC5	0.318202516	0.752445818	1
TBC1D9B	1.47462771	0.333189537	1

THBS1	-0.454459314	0.600707397	1
KYAT1	0.902634168	0.518755472	1
NUDCD3	-0.38807654	0.701045482	1
COL16A1	1.56159939	0.092859164	1
PPP1R37	0.442484807	0.721871615	1
RALBP1	-0.35571161	0.682279718	1
SAMD4A	0.257747269	0.851369221	1
BIRC6	0.481952182	0.692252439	1
DNAJC8	0.753069981	0.540197883	1
INTS11	1.482551203	0.240088681	1
PPP2CA	1.224724665	0.388672347	1
NET1	0.32078785	0.773176337	1
PECAM1	-0.906636856	0.329144179	1
IDUA	1.430724145	0.176475615	1
GRK2	-0.608669017	0.550212608	1
RTRAF	-0.335281227	0.722509527	1
CCDC6	0.315416761	0.793126508	1
KIF13A	-0.687237683	0.475305095	1
LRRC15	-1.878226398	0.403810862	1
ZFP36L1	0.011137985	0.991184677	1
RASIP1	0.459463787	0.710619505	1
G9826	0	1	1
NDUFC2	-0.42874076	0.655146125	1
PRELID3B	0.270971442	0.75035187	1
HDGF	-0.644819916	0.496698633	1
RNF31	-0.360727129	0.76133398	1
BAZ2A	0.314809019	0.732190833	1
CAPZA2	-0.388659293	0.750880297	1
NSD1	-0.276019429	0.798136053	1
SCAF1	0.368408401	0.701360517	1
APBA3	0.470632935	0.659703852	1
HSF4	0.609706571	0.646740021	1
MICU2	-0.012876871	0.987829393	1
LOC783202	0.544336148	0.474196675	1
ACAT2	0.605965667	0.560911891	1
SLC31A1	2.058221194	0.183545628	1
SMARCA2	-0.215305729	0.824262062	1
CNDP2	1.17958651	0.220008501	1
GLRX3	-0.317033338	0.703731104	1
CXCL16	1.342989543	0.125396478	1
LOC112445002	0.836129059	0.519430889	1
CASC3	-0.162770924	0.839084766	1
TIMM23	-0.916865804	0.327293355	1
RBM4	-0.294278869	0.7507438	1
LRRC2	-0.210792602	0.901251811	1
SLC44A2	-0.21182807	0.898856647	1
PIGS	0.887593971	0.364469803	1
LAMC2	3.637999526	0.141999231	1
HNRNPC	0.831093615	0.465377467	1
SGSM3	1.027455523	0.382629915	1
ITFG1	0.794224066	0.533379966	1
CAPZB	0.317111082	0.706862285	1
COL3A1	-0.273328683	0.82544175	1
TOP1	-0.018418051	0.985890589	1
PRKACA	0.372052717	0.753891699	1
ALAD	1.972636716	0.152802593	1
TAF2	-0.080054624	0.935219169	1
KIF1B	0.783148717	0.673320766	1

CIC	0.693476632	0.597360126	1
CTNND1	1.758068462	0.120162016	1
EMC1	0.267065557	0.836808963	1
RYR1	-0.85419278	0.53319912	1
DCHS1	-0.001520224	0.998630321	1
JUNB	-0.629217665	0.474205627	1
TNKS2	0.692384895	0.600971046	1
SRRM1	0.881708103	0.4821171	1
IARS1	-0.377582376	0.705489213	1
UBAP2L	-0.640667196	0.397270363	1
PRKDC	0.17746494	0.861706581	1
GLG1	0.96441421	0.463754111	1
NBAS	-0.177473858	0.836668945	1
SLC25A34	-1.102688559	0.50502687	1
CDO1	2.476107023	0.126543572	1
RALGAPA2	2.266901098	0.211105907	1
TBC1D8	2.065324395	0.572872939	1
TRAM1	1.07025686	0.266905449	1
GLUD1	-0.278739836	0.868861229	1
RBBP7	0.200372558	0.87060036	1
FBXO31	-0.814869343	0.498143971	1
MAPK1	-0.331372786	0.739115485	1
PCBP4	0.89121053	0.528176336	1
ATG13	-2.779205047	0.14720323	1
TRIM28	1.060211147	0.352370521	1
FAM78B	-1.828969589	0.408814601	1
H3F3B/H3-3B/H3-3A	-0.307234863	0.693205722	1
TNIP1	-0.295816477	0.818698022	1
LAMA3	1.077079241	0.273200668	1
MSS51	-0.850043759	0.346997596	1
PPP6R2	-0.375561023	0.714077847	1
ASPSCR1	0.737601054	0.531979341	1
EFTUD2	0.174064079	0.869551514	1
UBAP2L	0.637200526	0.582512041	1
MFSD10	0.624814789	0.56675481	1
TIMM8B	-0.768591146	0.447099879	1
BRD8	0.350540822	0.760970546	1
FHIP1A	0.830136132	0.510330611	1
WARS1	0.320780387	0.753894781	1
TAF1	-0.059744688	0.955912542	1
NEURL4	-0.494803367	0.614502284	1
USP8	0.922067851	0.375491308	1
PMPCB	-0.444366492	0.561134943	1
SMYD2	-1.113563338	0.212119986	1
SAP18	-0.110059475	0.916290533	1
UACA	-0.562108582	0.604122581	1
CLIP1	0.468363633	0.836865691	1
KDM4A	0.395691158	0.745503269	1
ARCN1	0.084729381	0.93386448	1
MAP1A	-0.292703039	0.767751072	1
ATP6V1D	0.928145725	0.277901	1
LGMN	0.282558901	0.820920372	1
VRK3	-0.296450278	0.775713364	1
ZC3H14	-0.718811273	0.351544717	1
SFRS18	0.624583888	0.597405179	1
SSR2	0.799559041	0.460639817	1
SGCA	-0.567917218	0.625908681	1
CAPN2	-0.089448423	0.930224954	1

SAFB2	-0.255336098	0.776985383	1
THRAP3	0.89177278	0.455391472	1
ADAMTSL4	1.147265923	0.399513823	1
SEZ6L2	0	1	1
NUP214	0.866719966	0.389465938	1
SPAG7	-0.712483162	0.443598021	1
TOM1	0.219918111	0.867934557	1
TMEM126A	-0.443858703	0.610767139	1
MMP2	-0.227385877	0.798679203	1
GEMIN7	-1.827889924	0.416876574	1
NDUFB2	-0.327595926	0.779642988	1
VPS13C	-0.45906419	0.784524943	1
SF3B1	0.266372494	0.834103639	1
CLK3	0.691376335	0.517973567	1
PDXDC1	1.727742644	0.186492505	1
MST1R	1.064608966	0.341443926	1
TRAPPC8	-0.041275006	0.962042084	1
TRIM63	-0.374923395	0.780799413	1
SURF1	1.594758088	0.1839282	1
ZMYND8	1.148414345	0.324005711	1
RMND5A	-0.588683095	0.727802362	1
PAK4	0.973812099	0.405878331	1
DNAJC10	1.18862704	0.363099001	1
ZNF638	0.891129845	0.469479927	1
GANAB	0.85837521	0.597359765	1
YWHAE	-0.625303118	0.597817048	1
NUP88	1.32502724	0.260059583	1
PIP	0	1	1
NDUFS6	-0.594759371	0.592778876	1
HIP1R	0.953419469	0.428447853	1
PHKG1	-1.057206203	0.311132992	1
CLPX	0.590659607	0.646125325	1
NDUFB11	-0.580119956	0.578782738	1
A2M	1.439129993	0.235799856	1
CDK5RAP3	0.483907784	0.630812561	1
SEM1	-0.180744349	0.868611872	1
DAAM1	1.353190093	0.103629331	1
PTK2	0.463597918	0.711011094	1
MED13L	-0.487878869	0.605997714	1
DNAJC21	-0.464286802	0.639040314	1
SNX1	-0.502179566	0.569029712	1
SRSF11	0.735518012	0.479408608	1
PNRC1	-0.670722602	0.494778246	1
NCSTN	-0.00955269	0.990855008	1
WIPI2	0.638577513	0.457816851	1
MRPS15	-0.432471031	0.670954757	1
ABCF3	-0.641362109	0.448149256	1
DEK	-0.412662579	0.679646037	1
ZFYVE26	-0.333913923	0.781892964	1
NIBAN2	1.148157387	0.298744698	1
CHID1	1.365223106	0.269405448	1
RREB1	-0.23131458	0.85170411	1
TMEM120A	1.011310135	0.495655586	1
FUNDC2	-0.93508743	0.302403144	1
HIF1AN	-0.81311274	0.349743412	1
ASH1L	0.535867661	0.681232787	1
POMGNT1	0.252796011	0.835763881	1
LUC7L3	0.854289441	0.467467759	1

TBC1D9	1.882713916	0.091812877	1
ADPRHL1	-0.277684126	0.790613675	1
LRP5	0.68084409	0.56341954	1
LARP4B	0.682948141	0.617695868	1
ANKRD40	0.00446311	0.99701108	1
FKBP15	0.404878931	0.647147149	1
ATP6V1F	-0.03015442	0.973504644	1
MTHFD1	0.030938563	0.976683398	1
PSEN2	1.373914641	0.285155547	1
CNBP	-0.423508117	0.743632929	1
ASCC2	1.066073076	0.427670574	1
DHX30	1.026787958	0.296594701	1
COPE	0.64163987	0.56682565	1
INTS8	0.913561338	0.475204083	1
CAND2	-0.738411109	0.56979645	1
CRELD1	1.436540684	0.197731743	1
ARL6IP5	0.708660318	0.567713805	1
ARFGEF1	1.122220235	0.363734711	1
PFKFB3	-1.299743938	0.295972926	1
CTR9	-0.157974972	0.843138916	1
DGCR2	-0.030275953	0.97739958	1
RHEB	-0.44219992	0.630569215	1
CACNG1	-0.522568199	0.684729083	1
PHTF2	-2.008457108	0.279988014	1
TRIM72	-0.494962912	0.743634869	1
IVD	0.89576097	0.494875825	1
SLC12A4	1.002627546	0.465218039	1
TAF15	0.692607452	0.593407102	1
REXO2	0.304228101	0.775244931	1
CHMP2A	0.602507181	0.551243516	1
CC2D1B	-0.022654077	0.984201525	1
HP1BP3	0.802177445	0.526387062	1
TARS1	-0.096999338	0.912542218	1
CEP104	-0.38596172	0.717043199	1
RYK	-0.265644622	0.755168692	1
IRS1	-0.851848099	0.406593606	1
COPS5	-0.517498873	0.570825157	1
CLCN4	-0.259049792	0.833111139	1
ENSBTAG00000054994	0	1	1
RAB3GAP2	-0.082350791	0.92294097	1
PLCB3	0.519896544	0.606184078	1
CDC5L	-0.259129251	0.780038387	1
MBTPS1	0.395133583	0.713513055	1
VARS1	0.308020368	0.788482957	1
CREBBP	-0.290119925	0.754669696	1
HECTD3	-0.444571076	0.682943986	1
DNAJC7	-0.543898584	0.560252793	1
SYPL2	-0.484315105	0.734207792	1
ARAF	-0.621350203	0.516869939	1
OPLAH	0.01116518	0.991513238	1
TNRC18	0.808837702	0.408499777	1
ANKRD1	0.218419565	0.884067871	1
ATXN10	0.958913521	0.482603713	1
TXNDC11	1.15873697	0.203830567	1
PARD3	-0.13164721	0.974789545	1
PON3	1.070401353	0.329573973	1
LOC516355	0.333920177	0.758146117	1
PRKAR2A	0.148270448	0.902792986	1

LRIG1	-0.723575774	0.358634519	1
ABAT	2.069480207	0.243613901	1
ZBTB7B	0.241348055	0.838461545	1
COL15A1	-0.789015754	0.411431402	1
STARD7	0.444613857	0.760043751	1
TMSB4X	0.1768921	0.879120822	1
PNPLA6	0.431730207	0.700930876	1
SMDT1	0.035235675	0.971673178	1
TSG101	-0.488870266	0.632916549	1
GORASP2	-0.001383005	0.998890695	1
UBE3B	-0.413597586	0.668455718	1
ZNF532	-0.685094751	0.558426005	1
DENND5A	-0.350010226	0.669127294	1
SLC5A1	0	1	1
COL6A3	-0.68784903	0.510600327	1
SZT2	2.147226058	0.112872423	1
SPCS1	-0.180231493	0.856660203	1
SMARCA4	0.129916666	0.891931035	1
MAP3K20	0.116155249	0.94659281	1
SLC25A30	-0.856979651	0.459213453	1
CLIP1	-0.019784141	0.986925241	1
EML3	0.895691728	0.498862069	1
DDX46	-0.120828862	0.901124603	1
GNPAT	0.008224007	0.99425755	1
GAMT	0.288443486	0.756143935	1
ILK	0.208408192	0.880236731	1
ARHGDIA	0.275022296	0.757968981	1
PDS5A	0.398253683	0.740756502	1
ADIPOR2	-0.876949133	0.355637042	1
COG1	0.016866315	0.988238097	1
SEC63	0.485222567	0.638211413	1
TSC22D3	-0.007226327	0.995012465	1
CYSTM1	-0.365173439	0.620268059	1
MRPS2	-0.502377336	0.621019021	1
LOC515736	0.323232704	0.775148585	1
SRP68	-0.473766254	0.567775444	1
ETF1	-0.580774393	0.721161296	1
HERPUD2	0.436545641	0.707340786	1
CRABP1	2.472099208	0.275792365	1
DCAF5	-0.440840481	0.570775498	1
CAP1	0.502211461	0.612621366	1
KMT2C	0.081505478	0.964600627	1
NDUFB5	-0.814885705	0.424187875	1
MAP3K4	0.468954533	0.727361369	1
UBE2O	-0.250119699	0.831498793	1
PPP3CB	-0.884055293	0.583641719	1
WBP11	-0.443047668	0.655285686	1
ATP1B1	0.107506085	0.939129817	1
PACS2	-0.678661792	0.47858945	1
G3BP1	-0.025726144	0.978946132	1
KMT2B	0.064318996	0.950410577	1
TENT2	-0.709946783	0.560070451	1
PSMB3	-0.136576341	0.888466374	1
VPS35	0.208809384	0.842029163	1
G978	-5.162746519	0.235528924	1
SNX7	1.74813346	0.182939925	1
ANP32B	0.072874115	0.946831752	1
INTS3	0.140628454	0.890652357	1

RER1	0.634931191	0.410579421	1
GOLGB1	1.149954173	0.334431317	1
HJV	-0.988137742	0.300535797	1
ISL1	0	1	1
RDX	-0.431612836	0.684809796	1
STAU1	0.022000055	0.985739653	1
MICOS13	0.015612253	0.98817424	1
GDI1	0.52394875	0.569848356	1
CEP85	0.153720508	0.933364724	1
STRAP	-0.627945806	0.472399765	1
MORC2	0.152294899	0.869992909	1
GET3	-0.423981324	0.687825044	1
CRY2	0.632121046	0.634626104	1
GATD3	-0.426313413	0.668580179	1
ANKRD11	0.640640238	0.586110812	1
PLIN2	1.82556296	0.198112308	1
BLMH	-0.317704177	0.769785733	1
TUBA8	-0.241444663	0.808219877	1
COL1A2	-0.18904788	0.865920089	1
LOC782545	0	1	1
MMP15	1.458701124	0.15302311	1
PSMD12	-0.008251347	0.993370358	1
RAB12	-0.986236755	0.337608976	1
MACROD1	-0.447374751	0.69685915	1
ZNF609	0.490515201	0.684424178	1
SLC25A5	1.570578214	0.082533999	1
BNIP3	-0.891677167	0.343499565	1
NELFCD	-0.20225959	0.851456346	1
EPAS1	-0.303172999	0.739647739	1
SERTAD3	0.751439818	0.360451059	1
TLE5	1.014058129	0.399605705	1
ARFGEF2	-0.317216267	0.727258146	1
TMEM87A	1.333404713	0.242614125	1
RBMX	0.159075788	0.875789052	1
SF3A1	-0.144008362	0.842969674	1
CLASP1	-2.247891258	0.326043344	1
DDRGK1	0.701359676	0.445038328	1
DRAP1	-0.3565331	0.755133883	1
CWC15	-0.516863141	0.591983118	1
EFR3A	0.7401856	0.538485579	1
AMOTL1	-0.464082084	0.62507437	1
HADH	0.16286134	0.876148937	1
SH3BGR	-0.548640647	0.587678017	1
CLUH	-0.663444694	0.525985784	1
STRN4	-0.978175232	0.281793708	1
TNPO2	0.31200227	0.770414354	1
DMPK	0.435188864	0.750501683	1
KHSRP	-0.16613234	0.871487938	1
SPG7	0.111418355	0.913270956	1
HERC1	-0.544098102	0.576527597	1
LIPM	0	1	1
APEH	0.966163975	0.463614272	1
ZNF512B	-0.370585476	0.737067226	1
XPO1	0.881176748	0.420195883	1
C4H7ORF25	-0.352009355	0.673948689	1
EMC3	0.3538014	0.751818691	1
AEBP1	1.365312199	0.229399962	1
CAMTA2	-0.672441194	0.512203295	1

LARS1	0.202809193	0.857776727	1
ANKRD17	0.462910765	0.70133691	1
CD44	0.85758697	0.341357895	1
SBDS	0.502951282	0.720021164	1
VMP1	1.739445497	0.110303742	1
LRRFIP1	0.013922377	0.993726631	1
AGAP3	-0.314042472	0.785266189	1
UNC13B	0.618530333	0.539336366	1
SEC23B	0.893393854	0.307377074	1
OCIAD1	-0.628768782	0.53068989	1
LIMA1	0.88804072	0.310053117	1
GNB1	0.388352394	0.671099776	1
PRPF6	0.017973155	0.982719775	1
TXNDC5	1.718565252	0.107150806	1
ERP44	1.068797771	0.162146655	1
CCNL2	-0.047729857	0.960980242	1
RAB11A	0.534187522	0.566869026	1
RBM39	0.124724158	0.863186859	1
PLCL2	-1.209683854	0.423000336	1
SYNE2	1.344650949	0.251293855	1
ALDH6A1	-0.129581222	0.923646307	1
KIF13A	-1.430373648	0.691483349	1
VEGFB	0.309981619	0.794509103	1
TOMM70	-0.204018897	0.854659098	1
UBR2	-0.609743553	0.559337321	1
FBXO40	-0.592927463	0.524373007	1
SSR1	0.428673917	0.563000554	1
FBXW5	-0.400160705	0.713769415	1
HCFC1	-0.322999205	0.702641762	1
NAMPT	-0.362962497	0.715025028	1
SREBF2	1.234332909	0.37362679	1
DNAJB1	-0.436456819	0.655586379	1
PRMT1	0.825061596	0.546509378	1
XAB2	0.847203617	0.361491769	1
DDX24	0.248333933	0.825222177	1
RASL10A	0	1	1
ENSMMSG00000022404	0	1	1
ACSS3	0.993669705	0.401387742	1
COQ9	-0.877361208	0.434630988	1
TLN2	-0.303360815	0.819506048	1
ADH5	0.318588502	0.784559776	1
AFF1	1.485782738	0.217444758	1
DDX23	-0.553704476	0.561456173	1
ZBTB16	0.425409694	0.752877895	1
MYLK	0.723289605	0.476821167	1
PMPCA	-0.51191677	0.609800751	1
PSMA3	-0.429231178	0.631089799	1
CYB5R3	-0.0237929	0.977362211	1
SMARCA2	1.606833068	0.38341825	1
OGT	-0.2935782	0.714005892	1
TBC1D17	-0.006891257	0.995583457	1
HMGN1	0.998825131	0.290134872	1
AP1B1	0.511767011	0.607425394	1
ECSIT	-0.896656318	0.420063903	1
HNRNPR	-0.042090103	0.969987683	1
MYH14	0.207633442	0.864978834	1
NAPA	0.128246578	0.888367729	1
BECN1	-0.368984968	0.700329093	1

CSE1L	-0.443429495	0.638970985	1
TPP2	-0.087613248	0.924869393	1
STAT1	-0.082436128	0.951188117	1
MTCH2	-0.286148644	0.730297609	1
YME1L1	0.289580359	0.826100021	1
SETD2	-0.204370522	0.810841593	1
GAS2L1	1.352238128	0.173196843	1
SAMM50	-0.458573912	0.608791063	1
NMT1	0.062075074	0.943100221	1
KIAA0319L	-0.88913179	0.395136644	1
RBM38	-1.128913897	0.297817004	1
RERE	-0.444277669	0.67246393	1
CLINT1	0.277427507	0.800203547	1
NAP1L4	-0.491931407	0.663343644	1
SART1	0.298550478	0.781231949	1
PSMC4	-0.480369591	0.598944839	1
SEPTIN7	0.163262674	0.881526099	1
PLAA	-0.362003095	0.722147935	1
NXF1	0.472019422	0.575387795	1
GNL3L	-0.0990393	0.918006422	1
CABIN1	1.479481533	0.267271896	1
IPO9	-0.122899028	0.893508926	1
ABCA2	0.706746171	0.45835089	1
NBEAL2	0.913754956	0.406342341	1
COG7	0.280700434	0.766504821	1
GOLGA3	0.992446092	0.566801393	1
TMEM59	1.187184911	0.16335958	1
ADRM1	-0.137118829	0.896298582	1
MICAL2	-1.210544165	0.313828696	1
TTC17	-0.209926411	0.821222615	1
DHX32	-0.641254984	0.50510123	1
FUCA1	1.445143433	0.178152079	1
KIAA1217	2.15140275	0.092474704	1
G993	-4.512330809	0.301357702	1
GBA2	-0.774356754	0.461633578	1
MAT2A	0.617746573	0.603264704	1
TRIM28	0.685609762	0.714503807	1
AKAP1	-0.246129848	0.849606381	1
SEC61A1	0.63930838	0.518739049	1
MTMR10	-0.011654743	0.990955545	1
TSR3	0.790908398	0.549515568	1
VPS41	0.417901481	0.706951012	1
CSRP1	0.131837147	0.898426031	1
BCL9L	0.500988508	0.642151908	1
TNK2	-0.050364108	0.963421723	1
SORT1	1.002248207	0.475903108	1
UBE4B	-0.053172447	0.949789479	1
SULT2B1	-3.708169266	0.39872454	1
TRIP10	-0.500642668	0.68605339	1
ZFAND5	-0.19691624	0.85788811	1
PLXNB1	1.706216614	0.182067674	1
FIS1	0.689052364	0.632530463	1
SIN3B	0.356934352	0.790300675	1
UTP14A	0.33669498	0.78704461	1
EPS15	0.427346629	0.663687185	1
ELOB	-0.057991105	0.959447229	1
PRDX5	0.130940626	0.892422671	1
FUS	-0.369322847	0.680042493	1

MGLL	1.274968599	0.612139167	1
RAD23A	-0.617988961	0.504465731	1
MMUT	-0.189551051	0.862511641	1
MYH10	1.615990348	0.269906	1
WBP2	-0.150747065	0.897837707	1
CAST	0.161653701	0.863652118	1
SQSTM1	-0.904175438	0.373333315	1
PIK3R1	-0.824472786	0.455774098	1
COPB1	0.626662956	0.517304766	1
KDM5B	0.513355492	0.59833775	1
CLSTN1	-0.33911312	0.69538992	1
EXOC3	-0.360325043	0.745917374	1
TRPM4	0.892938003	0.439056312	1
COL17A1	2.984801079	0.157164848	1
PXN	-0.556965381	0.564542776	1
COPS4	-0.63165716	0.510689856	1
NUDC	0.028627993	0.975863579	1
CDC37	-0.639537399	0.403754373	1
MEF2D	-0.927580912	0.296514606	1
RABEP1	-0.441448152	0.681958579	1
SLC20A2	0.537201496	0.701233898	1
ANTKMT	1.361368345	0.27293153	1
KLHL31	-1.024105183	0.302890197	1
ARHGAP21	0.320105157	0.755028687	1
U2AF2	0.117443627	0.900725897	1
CHD9	0.424479707	0.728369859	1
SERBP1	-0.423964731	0.644573722	1
DNAJA3	-0.536319669	0.58715164	1
CLTB	1.104054526	0.396570622	1
NDUFB7	-0.34535898	0.751697019	1
RAB7A	-0.161995973	0.859435573	1
VPS51	0.529379081	0.643365363	1
STT3A	0.132966888	0.861825224	1
LSM4	-0.418797448	0.67720936	1
UBA1	2.036534418	0.176018204	1
RBM42	0.281146568	0.806048945	1
SETD3	0.299086535	0.801326089	1
PARP1	-0.249822797	0.801642355	1
CCAR1	0.259596249	0.800734996	1
CALM3	-0.189956344	0.839353661	1
ABCB6	-0.320143977	0.730570952	1
CMAS	0.93396479	0.307289159	1
NAXD	0.978936191	0.443554127	1
UPF1	-0.026234375	0.980665092	1
MTA1	0.078226795	0.928173662	1
DIAPH1	1.224956812	0.388203952	1
CIC	-1.826471417	0.427977939	1
DDOST	0.395179199	0.619660619	1
EMC10	1.256494495	0.40749376	1
GLOD4	0.628561581	0.452642847	1
LSM14A	-0.196114626	0.854851463	1
SPARCL1	-0.874672281	0.370803299	1
EIF3I	-0.670930486	0.414276364	1
RAB1B	-0.200469029	0.849734862	1
FN1	-0.98300185	0.554173712	1
NUB1	-0.356768209	0.607682545	1
BZW2	-0.145366468	0.901079111	1
MGP	0.732579734	0.509721861	1

CHD2	0.057229385	0.943001501	1
GYG1	-1.07473321	0.16163182	1
ABCF2	-0.698906034	0.468952865	1
SIRT2	-0.771443479	0.488320857	1
PIK3R4	-0.421825896	0.654442777	1
MAST2	0.92059214	0.545079689	1
GADD45G	0.484463225	0.657027721	1
COX8B	-1.263066862	0.431991532	1
MRFAP1L1	-0.659862469	0.487547268	1
ENSMMSG00000000501	0.015521562	0.988713697	1
EWSR1	1.554503947	0.171195812	1
PAX9	0	1	1
DHRS7C	-0.77527501	0.52104819	1
ERGIC3	0.939781444	0.309341888	1
PSMD8	-0.220820044	0.819945887	1
SLC17A3	0	1	1
IDE	0.205394533	0.851285482	1
NPEPPS	-0.243507323	0.783102402	1
ATCAY	-0.740887266	0.528020516	1
RALY	-0.221497496	0.820140046	1
MLLT6	-0.498973675	0.601532436	1
NDUFA8	-0.830683216	0.475150726	1
PRRC2B	0.168884122	0.924185159	1
MPRIP	2.496364944	0.232310682	1
SRSF5	1.108525771	0.386950624	1
AKAP8L	0.975039663	0.310083714	1
MIGA2	1.157296906	0.303639118	1
ANK3	0.243887408	0.836381929	1
G22603	0.246722583	0.826502544	1
ITPR1	0.794520631	0.495392784	1
TJP1	-0.526849328	0.554860437	1
CHCHD3	-0.97707937	0.336685304	1
MYBBP1A	-0.100234978	0.891811983	1
RAB2A	-0.339027277	0.732125174	1
XPOT	0.11526371	0.915937372	1
PCNX3	0.31108758	0.77555998	1
OGA	-0.202334454	0.830487339	1
RNASEK	0.905511072	0.375178048	1
EIF3F	0.898210791	0.49733784	1
TMEM94	-0.288401862	0.813438814	1
PAFAH1B1	-0.249903347	0.750431052	1
ZNF592	0.304464025	0.843921503	1
PTBP1	0.337458702	0.728430992	1
EIF4G3	-0.77404943	0.408358222	1
RRM1	-0.250781714	0.80568	1
PPP3CA	-0.108861265	0.930112168	1
BCL2L13	-0.832811749	0.412032786	1
CUL9	0.919335056	0.409930802	1
CUL1	-0.62363818	0.514592867	1
UBL5	0.378236645	0.715429633	1
VLDLR	-0.678678542	0.560819357	1
HNRNPH1	0.395116962	0.83139828	1
SERBP1	-0.534900248	0.591015447	1
DOCK9	0.524561433	0.57634561	1
YWHAG	-0.746330946	0.42002095	1
GBF1	0.908847413	0.468166793	1
HOOK2	2.377392079	0.097798864	1
MBD3	-0.486182476	0.781047778	1

MYH8	-1.248626621	0.492201061	1
BAZ1A	0.577160869	0.662119647	1
DCAF6	-1.007261441	0.536582735	1
QKI	0.359898474	0.798662096	1
BAZ1B	-0.085259524	0.936965852	1
FKBP3	0.613436433	0.652636357	1
UAP1	-0.202279756	0.833231687	1
UBE4A	-0.319669531	0.75776026	1
SMTN	0.610611833	0.657272713	1
KIF13B	1.115753579	0.330376904	1
KDM4B	0.025538673	0.981130241	1
CRB3	0	1	1
AIFM1	-0.704749349	0.413806738	1
JUP	0.853799545	0.443898313	1
PDZRN3	-1.069948584	0.523052226	1
HIPK3	-0.665711145	0.548080131	1
TMEM147	-0.218179062	0.82207135	1
CPSF1	0.415561264	0.717157251	1
FAM120A	-0.158650941	0.889384256	1
MARCHF6	-0.174910376	0.805679669	1
CAMSAP1	-0.468069325	0.629931151	1
KDM5C	-0.326432386	0.747974303	1
HNRNPA3	0.648876214	0.551646603	1
PRSS22	-1.830686833	0.396685726	1
GOT1	-0.821662498	0.462768718	1
VPS13D	0.063323346	0.948915568	1
SF3B2	0.364974545	0.837168292	1
SYMPK	0.602196242	0.603555446	1
SMG5	-0.094414149	0.935906124	1
HNRNPAB	-0.146689745	0.878125697	1
ARID1A	0.065001234	0.94484939	1
PDHX	-0.802734715	0.630966342	1
FBXO32	-0.562334635	0.612830135	1
CIITA	0	1	1
DDX31	1.261002967	0.346959187	1
HBS1L	-0.383853269	0.71059198	1
CHMP4B	0.314284211	0.763099008	1
SRRT	0.340931861	0.740230103	1
PIGC	-0.922349936	0.354835386	1
AMZ2	-0.699432223	0.541250701	1
NDUFA13	-0.099565581	0.927284864	1
BZW1	0.854430775	0.398016398	1
SF3B3	-0.044295656	0.950780978	1
CDC42BPG	0.713656088	0.520752903	1
ATXN2L	0.850307978	0.479508617	1
DUSP1	-1.002664541	0.309910803	1
MAN2B1	0.622922458	0.537195933	1
MMADHC	-0.28380258	0.770534721	1
TERT	0	1	1
ENSBTAG00000008274	0	1	1
CDC42BPB	-0.029086401	0.974756596	1
RDH5	0.676132827	0.555205075	1
MAP7D1	-0.061525906	0.962247314	1
HSD17B8	-0.220686827	0.787652516	1
OBSL1	-0.151879297	0.906257513	1
GATAD2A	0.478366598	0.644431927	1
LTBP4	-0.433830209	0.724469091	1
DNAJA2	-0.204982404	0.811277242	1

CKAP5	0.463799094	0.710423245	1
KIAA0319	1.618273288	0.158437389	1
KIAA2013	0.28021057	0.793897691	1
FASTK	-0.31776395	0.771591047	1
PHB1	0.16872558	0.841835212	1
CHCHD10	-0.614731309	0.616550659	1
RTN2	-0.889832291	0.439794896	1
NARS1	-0.326875258	0.708903038	1
FHL3	-0.723925557	0.582431717	1
ZNF410	-0.127900223	0.91305335	1
SYNE2	0.706439548	0.597247336	1
SLC22A5	1.806517714	0.181343742	1
ST3GAL4	1.365491162	0.233537221	1
TRIM3	0.661628254	0.487778859	1
CYFIP1	-0.010134197	0.991919922	1
DLAT	-0.883446074	0.429482024	1
DYSF	-0.442842087	0.656012838	1
UTRN	0.246984275	0.776795669	1
GRHL1	1.5194008	0.15660604	1
SAFB	0.108496113	0.901454381	1
WASHC5	0.588744907	0.727425701	1
KPNA4	-0.077758297	0.944865267	1
LMAN2	0.730404342	0.335163318	1
PCNT	-0.499580577	0.624588774	1
THYN1	0.264283229	0.808276446	1
POMP	0.10298245	0.908614704	1
IFRD2	0.366048962	0.74495363	1
AKAP13	0.563912541	0.625783323	1
USP22	-0.009046704	0.993420782	1
RPL34	2.17852449	0.115368202	1
NSUN2	-0.463480798	0.645080918	1
TMEM38A	-0.904133699	0.59070133	1
AGRN	1.290680064	0.224381458	1
UBQLN1	0.177180677	0.86770151	1
GFM1	-0.877488049	0.319256026	1
ITGA7	0.004154505	0.99728971	1
KMT2D	-0.054330948	0.954135392	1
EGLN1	-0.789627581	0.335562267	1
PPP2R2A	1.030920673	0.404244354	1
QARS1	0.113828992	0.909883763	1
THBS4	-0.030939964	0.981492404	1
PSMD11	-0.265054984	0.762971634	1
PRDX3	-0.651888591	0.586513816	1
NPTN	-0.179292995	0.883066674	1
RPL36A	0.741064049	0.454554792	1
USP4	0.884381952	0.508859283	1
SUCLG2	-0.430356254	0.695534568	1
ATP5MC1	-1.138649728	0.192946979	1
INPPL1	-0.629338763	0.46153677	1
CIB1	1.253101457	0.183770667	1
TRPC4AP	-0.176245163	0.854123237	1
HNRNPD	-0.256669693	0.803292658	1
TFCP2L1	0	1	1
ARPC2	0.302785351	0.805394517	1
EIF2AK4	2.155290797	0.111643831	1
MAN2A2	-0.32732007	0.765911092	1
TRIP12	-0.33104979	0.73324402	1
GMPR	-0.942087012	0.370249553	1

SUN1	0.41658144	0.672629722	1
SETD7	-0.518302252	0.69681185	1
CHD3	0.489298815	0.81957917	1
NCOR1	0.417488131	0.654798479	1
AKR1B1	1.545957716	0.290735097	1
KLC1	-0.776496185	0.392732431	1
PSMD13	-0.013428157	0.984953712	1
VAPA	-0.616054683	0.514623794	1
PSMD6	-0.594729114	0.507004484	1
MAP7D2	0	1	1
NDUFA3	-0.323555051	0.772488793	1
LOC100848353/HNRNPA1	-0.209525748	0.79923812	1
NFIX	-0.79672496	0.388230205	1
TACC2	-0.386124368	0.731340402	1
RNF123	-0.738721897	0.495516552	1
NCAPD2	-0.220553486	0.895960762	1
ENSBTAG00000048771	1.815078875	0.08626706	1
PTPN23	1.043825922	0.432844756	1
FGFR2	0	1	1
EIF1	-0.880305326	0.240932551	1
G20187	0.808511679	0.707022445	1
USP19	-0.471519656	0.788894993	1
GOLGA2	0.467026343	0.665357515	1
SUPT5H	0.099154493	0.916829463	1
PSMB4	-0.141094575	0.88164833	1
CDK18	-2.849545596	0.152904314	1
EIF2S3	-0.245151534	0.736901975	1
TNKS1BP1	1.322592822	0.192980582	1
DENND4B	-0.607601616	0.601150102	1
C16H1ORF116	2.796016605	0.085785634	1
PRKCSH	1.250863443	0.177816027	1
PI4KA	0.097504268	0.925992909	1
HSPE1	0.297137	0.778595788	1
TRIO	-0.012975819	0.990186267	1
IK	-0.13183877	0.905417898	1
CPD	0.694214896	0.555852982	1
UQCRH	-0.357659501	0.741740469	1
TLN2	-0.95262782	0.249016568	1
EIF2S2	-0.46700278	0.576228584	1
FKBP5	-0.772265012	0.525742984	1
DUSP26	-0.396781471	0.706671172	1
PDK2	-0.752632237	0.530078038	1
HSD17B4	0.243733168	0.804812361	1
PPP1CA	-0.014325025	0.98862751	1
EIF4EBP1	-0.434437821	0.695130303	1
MCL1	0.636723898	0.648226782	1
C3	1.804983385	0.117240974	1
ZSWIM8	1.002951	0.380580282	1
ZNF462	-0.274825399	0.793934637	1
CLPTM1	-0.150584356	0.865666589	1
MAN2A1	1.275156028	0.203087944	1
RPS6KA3	-0.293749025	0.769353296	1
PTGES3	-0.464601772	0.609485188	1
LETM1	0.283766387	0.781011553	1
SEPTIN9	1.427652995	0.098391228	1
LPO	0	1	1
CD9	0.689602747	0.482446242	1
LOC515570	0.339783834	0.679267236	1

SCYL1	0.055408478	0.956579162	1
EIF4H	-0.455321939	0.641720504	1
PDIA4	0.74771048	0.41722639	1
MIA3	0.72134045	0.505344966	1
CDC42	0.457403089	0.692454805	1
SHISA4	-1.130121189	0.218441089	1
KPNB1	-0.458840721	0.643899358	1
MPC2	0.353007289	0.793607539	1
PNPLA2	-0.704152321	0.566660235	1
ABCA3	1.074292653	0.334951537	1
EZR	0.92221306	0.329903743	1
SYNCRIP	0.239872351	0.816466103	1
SMARCD3	-0.926111457	0.428889672	1
CORO6	-0.107608878	0.937723634	1
USP5	-0.022862137	0.979824284	1
ENO1	0.863488378	0.591638378	1
RNF157	-1.014049548	0.507126864	1
CHD8	0.071978376	0.949877083	1
MAP2K2	1.224525636	0.399415486	1
HSDL2	0.887346964	0.515268336	1
RAF1	-0.8062788	0.461014614	1
OXA1L	-0.626620456	0.391288161	1
KXD1	0.941676992	0.419551677	1
DAG1	-0.593435408	0.515926573	1
AP2M1	-0.414641632	0.669266596	1
SMARCC1	0.428007936	0.663198287	1
BTBD1	0.34513491	0.791456769	1
SOD2	-0.789905697	0.398532244	1
ADCY2	-0.621450058	0.583611806	1
USO1	-0.645159937	0.444779195	1
TRAP1	-0.086496989	0.930632774	1
IDH3B	-1.304762801	0.19133776	1
PSMB5	-0.447218132	0.617783099	1
SFPQ	0.049154565	0.957209311	1
PALB2	-0.775128467	0.397665739	1
MYO1B	0.756115195	0.428095533	1
SLC25A12	-1.093569655	0.319255231	1
SMTNL2	-1.117499206	0.329555426	1
ALKBH5	-0.752666139	0.481977391	1
CCT2	-0.181007638	0.85173755	1
PDIA6	0.437291091	0.593315943	1
CTSB	-0.337496344	0.740361534	1
SECISBP2L	1.156405937	0.343492021	1
CST3	0.112765565	0.906898745	1
G18958	-0.019280751	0.985291813	1
NDUFB8	-0.875880611	0.303682203	1
PSMD7	-0.33626802	0.688313041	1
KIAA1522	1.805603308	0.150082319	1
SRRM2	0.398300977	0.708578172	1
VCL	-0.868758762	0.702978467	1
TPM1	-1.342452289	0.262937577	1
PBXIP1	-0.059558135	0.953595768	1
CAT	-0.429150294	0.686839998	1
ABCF1	0.228198439	0.844535993	1
INTS1	1.047120759	0.456934987	1
SCRIB	0.819415714	0.482516533	1
MFSD6	0.841431255	0.614004465	1
MPC1	0.387657263	0.759155599	1

PSMA6	-0.362436476	0.700994621	1
EIF3L	0.997657728	0.439031874	1
PABPC4	-0.088284187	0.944609722	1
PHB2	-0.762741152	0.422976065	1
IGSF9	0.493533799	0.615384219	1
SMG1	0.470395049	0.661555721	1
NRDC	-0.35831398	0.834572672	1
ATP5MJ	0.017281526	0.987791429	1
TACC2	-0.371791856	0.84251158	1
ANXA5	0.440627805	0.669182745	1
HDAC5	0.198096687	0.884558892	1
ZFP91	-0.852769829	0.607429826	1
FDPS	1.52092943	0.174030877	1
CCAR2	-0.074219883	0.940969082	1
TSC22D1	-0.187247222	0.886084793	1
LRRC39	-0.748750571	0.620981247	1
ENSMMSG00000011457	-0.505548653	0.525340692	1
SLTM	-0.371652765	0.702142781	1
MKNK2	-0.287150469	0.775607871	1
LENG8	0.391231863	0.718287071	1
DNAJA4	0.232894835	0.863025645	1
LRP10	0.243223766	0.800997058	1
TMEM109	0.43993647	0.711147658	1
LAPTM4A	0.19486351	0.834874464	1
IARS2	-0.528134102	0.603019087	1
DHX9	0.004905347	0.996063706	1
SH3BP4	2.034493481	0.086677457	1
MAF1	-0.921432374	0.392080094	1
BCKDHA	0.123243849	0.895561769	1
PA2G4	-0.070877163	0.944937318	1
PDLIM7/DBN1	-1.087854609	0.287076518	1
GSPT1	-0.440759929	0.657252753	1
ZZEF1	-0.55336036	0.611034516	1
CCT4	-0.048811695	0.956681752	1
SBF1	-0.026507904	0.980503266	1
TPR	0.799719709	0.519666935	1
TMEM131	0.81512217	0.53589443	1
TUBB4B	-0.036199566	0.973135504	1
LOC783680/B2M	-0.702500915	0.461529445	1
CTNNA1	0.5822217	0.634575436	1
SUPT6H	1.159726172	0.542257982	1
HNRNPUL1	-0.094785361	0.918240677	1
PSMB7	-0.255416405	0.793688514	1
STUB1	-0.461071725	0.622357126	1
TUBB	0.394653729	0.663636042	1
SARAF	0.741364324	0.446261134	1
DCAF8	-0.778140489	0.479301659	1
ATP6AP2	1.391296312	0.187456422	1
GSTA4	1.772105396	0.153699878	1
CLCN7	0.503580178	0.695383888	1
PSMA7	-0.353808806	0.726085758	1
LTBP3	0.712831382	0.463236594	1
SSRP1	-0.389531308	0.692930198	1
HSPB8	-0.458383256	0.728951303	1
MAN2B1	1.241979302	0.244990038	1
GFPT1	-0.206232367	0.827866866	1
CSNK1A1	0.033847962	0.974544673	1
UTP6	-0.404242017	0.678309739	1

UBE2B	-0.126516563	0.906404608	1
ZC3H11A	-0.395693291	0.696153552	1
CALCOCO1	-0.489925038	0.626071061	1
AK1	-1.279450369	0.256159829	1
CSNK2B	0.220610899	0.835155918	1
ADCY9	-0.547841038	0.580473268	1
PDE8B	1.511059489	0.212445512	1
WNK2	-0.341571738	0.793102276	1
PLIN4	-0.252573426	0.887664513	1
POLDIP2	-0.649857418	0.466391631	1
FLOT1	-0.701829685	0.311383628	1
TMEM72	0	1	1
CALCOCO2	-0.38668856	0.696178706	1
ACOT13	1.018441317	0.342496874	1
TCEA3	-0.264449261	0.854833645	1
G18957	-0.008247019	0.994233134	1
UQCRQ	-1.050558948	0.318160644	1
RNH1	1.17712195	0.346458716	1
LOC508916	1.27574405	0.29107786	1
CLTA	0.518233943	0.579118978	1
EIF5A	-0.617509927	0.470809327	1
EP300	-0.182336886	0.851274919	1
TRABD	0.622643104	0.587703434	1
NBR1	0.049171936	0.95502978	1
USP9X	-0.062505335	0.956140882	1
RHOBTB3	1.352083213	0.393363695	1
EHBP1L1	0.361272711	0.800741713	1
LARP1	-0.171702439	0.860896031	1
FBN1	-0.763505123	0.442379395	1
COL4A1	-0.634233416	0.475232321	1
RPL22	0.85455839	0.424458654	1
DCAF6	-1.146847367	0.210984333	1
ERGIC1	1.514968348	0.24802457	1
SBSN	-6.402017658	0.14002022	1
PAIP1	-0.956898724	0.261045043	1
ME1	1.766181848	0.145328504	1
COPB2	0.225123349	0.817054258	1
NME2	0.51017539	0.57705758	1
ZFP36	-0.52870939	0.570796692	1
TEX2	-0.664222959	0.506842157	1
BAG6	0.506772402	0.635713369	1
FDFT1	-0.10438226	0.930401044	1
HMOX2	0.6106683	0.601321964	1
BCAP31	1.212525501	0.132898464	1
NDUFS5	0.124588671	0.910378256	1
TBX5	-1.872534413	0.387756834	1
MYL6	1.955176027	0.182394883	1
PTP4A3	-0.856990852	0.383325896	1
RILPL1	-1.237051549	0.211561369	1
AUP1	0.33162429	0.70304878	1
PKN1	-0.398327183	0.729633679	1
TMEM265	0.11748259	0.892724122	1
RNF103	-0.070643192	0.928934507	1
FAAP100	-0.233532551	0.827808723	1
GRN	0.920444397	0.400705302	1
IMMT	-0.698580728	0.435983386	1
SHANK2	0	1	1
HUWE1	1.214551562	0.343696815	1

TEP1	-0.230590945	0.857000509	1
AFG3L2	-0.797104763	0.412173268	1
DRC1	-1.82433941	0.445933363	1
KLHDC3	-0.86644674	0.271378562	1
ENSMMSG00000000152	0.081114006	0.955588876	1
TP73	-2.848403524	0.131783558	1
CMPK1	1.883108117	0.118898439	1
PAM	-0.115413855	0.915565137	1
SUCLA2	-0.831739862	0.384869343	1
NFIC	-0.101605262	0.91217832	1
MFSD6	0.708717444	0.57717683	1
ACAA2	1.002603369	0.434386624	1
GPAT4	-0.054330082	0.939263407	1
NCKAP1	-0.163037221	0.879340844	1
WNK1	-0.524717443	0.523947298	1
ACOX3	2.541863891	0.088685973	1
PSMD3	-0.480192757	0.615614578	1
CLK1	-0.221893291	0.824352559	1
HNRNPH3	-0.045227945	0.965527484	1
TECR	0.128754487	0.903292314	1
ACIN1	0.442639407	0.659634295	1
NSFL1C	-0.376411194	0.681374584	1
ACACA	1.518864686	0.191447735	1
ATP5MC3	-1.014008086	0.288591927	1
SYNE1	-0.690136439	0.690041229	1
KIF1B	-1.806246129	0.412838818	1
RILPL1	-0.284164191	0.855004498	1
AHSA1	-0.469036941	0.600817117	1
RNF128	2.1676775	0.082372097	1
RPS28	0.833016811	0.484738646	1
AMDHD2	1.046617501	0.284699981	1
SDHC	-0.696837282	0.505249088	1
ATP2C1	0.203708397	0.848545671	1
CPT1B	-0.055338903	0.965800978	1
RBM5	0.17855207	0.848253172	1
IDH1	2.094291743	0.12444934	1
PDHB	-0.948353074	0.301705509	1
SLC9A3R1	1.879413088	0.182681371	1
NSA2	0.254545094	0.831105096	1
LONP1	-0.06787918	0.939105344	1
GABRP	0	1	1
ENSBTAG00000047849	0	1	1
ARIH2	-0.867879772	0.400768312	1
ARHGAP35	0.923869058	0.477196829	1
ANK1	-1.154277069	0.44994771	1
NPM1	-0.466890368	0.632459214	1
SKP1	0.772938863	0.552911602	1
KIF5B	-0.265800474	0.79308491	1
NDUFS3	-0.979083145	0.326266779	1
ERP29	0.764872228	0.406446048	1
HRC	-0.878656394	0.490862354	1
PER1	-0.213141062	0.831195527	1
ALDH1A1	0.298515484	0.823699281	1
ANXA6	-0.379661675	0.710980351	1
RETREG2	0.231552972	0.848906991	1
KEAP1	-0.630993286	0.542804513	1
BTF3	-0.23024254	0.821000892	1
POLR2B	0.335121226	0.766034585	1

CALD1	1.193905023	0.218034711	1
DCAF11	-0.512065779	0.547633392	1
SUCLG1	-0.901765041	0.317412965	1
ISCU	-0.712167331	0.439274051	1
CAPRIN1	-0.207592019	0.848913726	1
FARSB	0.5846816	0.636057125	1
MEGF8	0.686099888	0.561060523	1
CASTOR2	-0.993267837	0.366040047	1
FHOD1	-0.735867816	0.472345446	1
CTNNB1	-0.219885091	0.832759459	1
PEBP1	-0.154527241	0.886024373	1
PARK7	-0.718382979	0.370279179	1
LOC100848353/HNRNPA1	0.117107136	0.882310578	1
DLST	-0.82235387	0.46632166	1
ETFA	-0.863785974	0.339432664	1
IDH3G	-0.783196828	0.412589905	1
BAG3	0.178373025	0.896572079	1
CHD4	-0.37789132	0.694920251	1
UFD1	1.40668849	0.212993119	1
RPL37A	0.97005027	0.405612622	1
VIPR1	1.763579681	0.450101933	1
ETFDH	-0.589649376	0.595605169	1
CCT5	-0.002556547	0.998064915	1
MFN1	-0.395985546	0.682695401	1
NDUFB9	-0.599727319	0.578917443	1
ATP6V1E1	1.126646448	0.286825035	1
AMFR	-0.486731016	0.634316614	1
CLU	0.78445292	0.497660972	1
THRAP3	0.41719409	0.716477666	1
COL6A2	0.050660879	0.95702263	1
DDX39B	0.737909495	0.452416343	1
FAH	1.08883937	0.332575888	1
GPX4	-0.018384045	0.984896815	1
COX7A1	-1.258917906	0.29560325	1
CAVIN1	-0.86717834	0.308008307	1
CTSC	0.809222789	0.420212708	1
COPS3	-0.790651399	0.325993128	1
DCTN1	-0.126230101	0.929315921	1
MLXIP	-0.833988843	0.516583774	1
HERPUD1	-0.119007493	0.912678702	1
CALR	0.70569317	0.460855846	1
KRTDAP	-4.290667808	0.326422812	1
SMARCA5	0.2955033	0.769416747	1
MYL6	0.648731185	0.475342457	1
HYOU1	1.049023942	0.215964386	1
EIF5B	0.831897746	0.515031151	1
TAGLN	0.424733592	0.651909895	1
LPIN1	-1.029655008	0.320416152	1
UBAP2	-0.225899742	0.832982961	1
PFN1	0.296363043	0.753923773	1
KLHL40	-0.303105577	0.855340306	1
TUBGCP2	0.110534138	0.9010755	1
AHNAK	-0.611406654	0.480254903	1
NEXN	0.263871266	0.853442102	1
SERF2	0.421532134	0.668397977	1
UQCRFS1	-0.779276424	0.444628268	1
CLUH	-0.212976577	0.838437122	1
WIF1	0	1	1

PTP4A2	-0.942837435	0.337870968	1
EIF3M	-0.16861881	0.867996259	1
BRD4	0.11560922	0.909547363	1
CD164	0.558941234	0.631736952	1
DPP7	1.375419277	0.227117812	1
STT3B	0.823053702	0.396965062	1
CCDC88C	-1.858776226	0.383968224	1
FOS	-0.928734058	0.395682812	1
DCTN1	1.758024036	0.434435038	1
LAMB2	0.211461663	0.842355362	1
GNPTAB	1.118942787	0.268388186	1
ABRA	-0.464890825	0.757586523	1
CDC34	-0.837046076	0.407073518	1
BCL6	-0.347567896	0.7384387	1
HNRNPUL2	-0.217336111	0.804808165	1
GPD1	-1.027960878	0.346332213	1
SNRNP70	0.860727878	0.435343107	1
ATP6V0D1	0.520340983	0.542761421	1
HADHB	0.387335691	0.763851303	1
EIF5	-0.540172569	0.50355354	1
CUL3	-0.658382267	0.474115556	1
LIMCH1	-0.590328115	0.591364757	1
COX5A	-0.908394573	0.322449524	1
UBXN1	-0.717410218	0.567443298	1
TMEM259	-0.507651522	0.629629148	1
SOD1	0.214004587	0.80530168	1
MACROD1	-0.095925787	0.942367973	1
JSRP1	-0.48171958	0.720780011	1
AGL	-0.878457137	0.398441843	1
SPEN	0.015774926	0.987546789	1
ARF1	0.240731534	0.838769759	1
ATP9A	0.16432321	0.910833491	1
ALDH3B1	-0.295327017	0.806173834	1
PACSIN3	-0.75531877	0.477571514	1
RRBP1	1.221113386	0.177583704	1
SLC22A15	0	1	1
ECH1	-0.259068762	0.795857114	1
EPN1	-0.602239907	0.586869988	1
SCUBE1	0	1	1
NISCH	0.97992358	0.272127764	1
SDF4	0.469979186	0.563611017	1
RAPGEF1	-0.888961629	0.404604957	1
OPTN	-0.374697924	0.779647299	1
KARS1	-0.222305892	0.899557615	1
RANBP2	-0.194420403	0.856106817	1
ADGRB2	0	1	1
STAC3	-0.907968608	0.574628677	1
ACO2	-0.538576903	0.614767445	1
LOC508916	1.447611867	0.386548835	1
HNRNPH1	-0.272484298	0.783689164	1
ASB5	0.068745743	0.960588487	1
CCT6A	0.178378669	0.861048531	1
FBP2	-1.106635884	0.393693157	1
SRPRA	0.59781047	0.595694879	1
FLNA	0.877938236	0.46739265	1
ALDH2	-0.9056661	0.383331847	1
G9694	-0.510427583	0.679456758	1
EHBP1	0.612592023	0.707531988	1

CAPNS1	-0.184120186	0.839822853	1
RBM42	-0.916093888	0.59268679	1
CCT8	-0.183749465	0.859826962	1
BBS5	3.006750302	0.260484288	1
COL12A1	0.27955594	0.784260812	1
PLD3	1.426750608	0.188129371	1
PPP6R1	0.388865486	0.731582208	1
LRRC31	0	1	1
G22449	1.021624109	0.347338698	1
NDUFS7	-0.827652246	0.473191668	1
HSPB7	0.219520497	0.903388493	1
XRN2	1.172853033	0.324220952	1
ATP5MG	-0.64633916	0.467612923	1
ATP6AP1	0.712651203	0.51474293	1
PDCD6IP	0.354209641	0.759117279	1
COPA	0.314059725	0.794757845	1
OXCT1	0.55422869	0.682806937	1
NADK	0.828582362	0.472339657	1
DNAJA1	-0.082845722	0.933388621	1
BCO2	1.05482629	0.270227682	1
MYO1C	0.027004717	0.983771654	1
DDX3X	-0.40635795	0.656418252	1
KMT2C	0.095822151	0.935265481	1
KMT2C	5.915884838	0.156920599	1
WNT7B	0	1	1
NUCB1	-0.259785769	0.795522848	1
PARP3	-0.834042919	0.488171895	1
SNPH	0	1	1
ATP5MF	-0.504248998	0.628254934	1
NR4A1	-1.197680113	0.214662211	1
CCT7	0.468982648	0.695281743	1
NDUFV2	-0.70507938	0.453725721	1
C9H6ORF118	0	1	1
MCM3AP	0.271509505	0.808956044	1
STYXL2	-0.224374597	0.891956024	1
TUFM	-0.506028062	0.617927878	1
CFHR5	0	1	1
ALDH7A1	0.101084621	0.916602132	1
SUN2	0.538282211	0.687408411	1
EIF3K	0.736159284	0.542687202	1
RPL36AL	1.732374756	0.172812751	1
HNRNPK	0.309687114	0.802283556	1
ANAPC2	1.397941799	0.286707956	1
USP13	0.027175628	0.98350831	1
PLXNB2	0.818698473	0.375634186	1
FOXQ1	-1.88508997	0.423355232	1
RPL3L	-0.227208647	0.874743773	1
BCKDHB	1.256902091	0.138471883	1
ANO1	1.614375855	0.145044611	1
KIF1B	0.496070605	0.699389258	1
ECHS1	-0.042014642	0.965168381	1
ATP5PD	-0.689178671	0.514109732	1
RAD23B	-0.74657971	0.449967235	1
RB1CC1	-0.818102826	0.336667004	1
TRIP12	0.092170158	0.935641657	1
PSMC2	-0.396527523	0.644209191	1
AARS1	-0.211243817	0.790349076	1
TCF25	0.351268899	0.768813965	1

CHCHD2	-0.871328257	0.328689782	1
PTPN21	-0.899890602	0.458856629	1
LAMA5	0.235369849	0.825253396	1
RPL38	0.450576963	0.702173618	1
RNF10	-0.557834356	0.524038291	1
YWHAE	-0.226791509	0.81388006	1
MYO18B	-0.283736256	0.866515726	1
PCCB	0.461952911	0.607829887	1
IQGAP1	0.669063749	0.481914961	1
CD63	-0.142712476	0.88206133	1
TCP1	-0.277686188	0.771996403	1
TM9SF2	0.39095393	0.683319484	1
GPCPD1	0.039505306	0.976671455	1
ARPC1B	1.423275312	0.201498733	1
PPP1CB	-0.510960075	0.630314219	1
ATP5PF	-0.642762209	0.510171111	1
SLC25A11	-0.829378524	0.424678689	1
CYCS	-1.048601081	0.305482343	1
TXNL1	-0.148807416	0.875204657	1
ECI2	0.78090514	0.477193753	1
TRDN	-0.450308824	0.772027967	1
TNFRSF21	-0.104244493	0.936481898	1
ENSMMSG00000010492	-0.132924722	0.899546948	1
MFN2	0.107775526	0.938239854	1
SCP2	1.487662625	0.266169498	1
CAPSL	0	1	1
PSMC1	-0.287350245	0.761057273	1
ATP5IF1	-0.557168791	0.618884796	1
FH	-0.911511208	0.288487336	1
DCTN2	-0.215418655	0.820291952	1
NFE2L1	0.114247689	0.934251078	1
AKAP9	1.183593275	0.401660136	1
PSMB1	0.083968749	0.931833062	1
CUL7	0.428808668	0.694210086	1
CRHR1	0	1	1
PSME4	-0.692264237	0.517590244	1
ETFB/VSIG10L	0.582864208	0.549793387	1
IVNS1ABP	-0.844930918	0.41307441	1
COPG1	-0.152216135	0.87127411	1
SLN	-1.327643819	0.379779493	1
RPS3-A	0.572061195	0.709792729	1
HADHA	0.102548056	0.931278442	1
MATR3	0.627898917	0.6222577	1
EPRS1	0.077487674	0.934280855	1
USP47	-0.447949644	0.668665139	1
GDF10	-1.87195188	0.386114733	1
PDE4DIP	-0.47023904	0.672897086	1
MIDN	-0.756224203	0.393900883	1
HNRNPL	-0.185919786	0.832470755	1
CD81	0.373418792	0.683165475	1
NACA	-0.229386046	0.896694839	1
CNOT1	-0.293786504	0.771749623	1
CSNK1G1	1.641883396	0.138120197	1
GCN1	-0.213159286	0.834605398	1
GCAT	1.190423957	0.341474186	1
RNPEPL1	0.142315973	0.902755199	1
PPL	1.628439075	0.128232697	1
SLC25A3	0.777881441	0.644466153	1

OVOL2	0	1	1
EIF3H	0.726835908	0.563633685	1
PHKB	-1.159694865	0.389491101	1
LOC782609/NAP1L1	-0.259787468	0.789913269	1
PPDPF	0.954173663	0.487524119	1
CCNG1	0.06392906	0.957235641	1
FERMT3	-0.171390464	0.873095397	1
LMNA	0.446298915	0.689046896	1
JAK1	-0.325320895	0.767043335	1
DDB1	-0.014419461	0.986709869	1
WDR6	0.922338222	0.366607329	1
SCN4A	-0.930302186	0.541506056	1
WWP1	-0.974953341	0.364604618	1
MAPKAPK2	0.071481389	0.955260093	1
OVOL1	-1.87485945	0.394305388	1
PLEKHM2	-0.845848769	0.403350222	1
NDUFA9	-0.80123947	0.32935199	1
ZNF106	0.284733287	0.836116695	1
MROH1	1.702079449	0.151676074	1
IPO7	0.32586271	0.784160986	1
CALM2-B	-0.628854765	0.530728068	1
CCNI	0.596817064	0.61630591	1
TRAK1	-1.003891471	0.351697864	1
MUSTN1	0.283961518	0.845200651	1
ACOT4	0	1	1
PHKA1	-1.179807369	0.309056068	1
ZPBP2	0	1	1
ACLY	1.928368522	0.343144377	1
PSMC3	-0.001405499	0.998848734	1
FYCO1	-0.412779001	0.749683127	1
PRDX2	0.832279967	0.434671125	1
HSPA4	1.02834209	0.393447956	1
CCDC47	-0.215389432	0.843007038	1
NIPSNAP2	-0.347432152	0.757427244	1
GABARAP	-0.037951147	0.965814037	1
DLD	-0.705365525	0.491329908	1
SEC62	1.582840387	0.113155685	1
MACF1	0.455574888	0.70696908	1
KCNMA1	2.266321281	0.119485633	1
RALGPS1	-2.849116576	0.144429845	1
KAT2B	0.630566067	0.624133459	1
EIF3G	-0.378147183	0.663163376	1
EIF2B2	1.563009647	0.119358858	1
DDX17	-0.02526387	0.983019624	1
NDUFB10	-0.842614048	0.415949825	1
TBC1D24	1.326954227	0.277603001	1
ITGB1	-0.056218643	0.956241818	1
BAG6	-0.8284886	0.645165842	1
PSMD4	-0.376768922	0.729127235	1
CS	-0.8059712	0.460762049	1
PFKFB4	-1.114990925	0.37526363	1
RGL2	0.282771778	0.801306827	1
CRAT	-0.359270941	0.748221846	1
AP3D1	0.291208162	0.784425747	1
NECTIN4	4.102030505	0.100728848	1
LDHB	1.910366804	0.185153843	1
SPARC	-0.559967006	0.562107084	1
SLC17A2	0	1	1

CAP2	-0.964752401	0.394003355	1
ENSMMSG00000013681	2.240585918	0.353161798	1
RTN4	-0.750052538	0.386634171	1
ASB2	-1.039156922	0.324188022	1
CLSTN1	1.931405393	0.245161201	1
PRKAR1A	0.199520013	0.829289953	1
MYO18B	-0.365673062	0.819580971	1
MYH7B	0.653667978	0.675137273	1
HNRNPA2B1	-0.558496561	0.540301557	1
HSPH1	0.817574693	0.491552446	1
HSPA8	0.039179161	0.966627647	1
NOP53	1.628879804	0.222194151	1
PHYH	0.635794406	0.643481049	1
USP25	-0.901303964	0.336064731	1
GOLGA4	-0.590421393	0.560720216	1
UBAC1	-0.905769296	0.375278717	1
PSMC5	-0.204999327	0.846660945	1
HSPB6	-0.140340398	0.915923393	1
ITPR1	1.567826642	0.7066197	1
HSPG2	-0.786783953	0.363255103	1
G36195	2.184369146	0.092207572	1
PSMD1	-0.194439933	0.837065131	1
SORBS1	0.06323737	0.961973968	1
TNNT3	-1.595735625	0.32261185	1
GLB1L3	-1.872671975	0.388148201	1
ATF6B	0.184977718	0.854860232	1
ENSBTAG00000053057	-2.850230217	0.168179883	1
CCDC158	0	1	1
RPL15	0.227057268	0.7977729	1
ADSS1	-1.124883965	0.316176145	1
MYL3	0.205274097	0.891870299	1
JUN	-0.929256085	0.407879802	1
ANK2	-1.801471666	0.286605136	1
MTERF3	-1.016271361	0.283491917	1
HNRNPF	0.111095605	0.885891994	1
GSTP1	0.25826693	0.811481811	1
CALR	0.716724421	0.389433339	1
TTC6	-1.830610573	0.39720738	1
TNS1	-0.513584606	0.740133553	1
KCNG4	0	1	1
PPP2R1A	0.947698251	0.502152748	1
EIF3B	0.314876288	0.76151529	1
DDX1	-0.443474491	0.68731251	1
ERBB2	1.248844017	0.338485373	1
G24175	-0.562105375	0.586949423	1
UNC45B	-0.424812751	0.726953687	1
GNAS	-0.12642897	0.943605786	1
GLUL	-0.921352455	0.380835887	1
EIF4G1	-0.404867173	0.719667203	1
EIF3D	1.258125066	0.360840015	1
TTN	-0.793565461	0.738031076	1
ATF4	-0.617230367	0.518476361	1
RHOD	-2.848583081	0.134843439	1
RPL30	1.275504377	0.282816907	1
TAX1BP1	0.615724808	0.549341004	1
NDUFA10	-0.690485626	0.499539471	1
JPH1	-0.881649679	0.585048087	1
COX5B	-0.747357978	0.414465349	1

SYNM	-0.245941878	0.866644107	1
NUCKS1	1.982962797	0.143770507	1
HNRNPA2B1	0.47732015	0.690642796	1
USP7	-0.466914949	0.609131341	1
MYPN	-0.633797629	0.685568001	1
DCN	-0.290474749	0.768101233	1
TOP2B	0.478176304	0.650316391	1
UBA1	-0.396735532	0.824112091	1
ATP5PB	-0.897620461	0.318105668	1
UBR3	-0.747505094	0.45646983	1
GHITM	-0.270455514	0.813397455	1
TUFT1	0	1	1
SERPINB5	-1.875138343	0.395101137	1
EIF3A	-0.00724443	0.994860676	1
MYOM3	0.386243568	0.845296378	1
ANXA2	0.985129526	0.367172777	1
ANXA1	1.406183744	0.176311693	1
TRIM54	-0.132206275	0.916428827	1
RPN1	-0.065786435	0.945770956	1
NDUFV1	-0.601243804	0.607999122	1
EPHX1	0.558953269	0.57398126	1
RNF19B	1.786171248	0.219029728	1
VDAC2	-0.897471576	0.390902416	1
MORF4L1	-0.437871059	0.638589383	1
VAT1	1.905680863	0.119450765	1
HSP90AB1	-0.337069957	0.766152781	1
CCT3	0.847857718	0.479793364	1
MDH1	-0.777863433	0.268709008	1
GPT2	-0.44111894	0.703760458	1
MYH11	1.577131213	0.35401162	1
VIM	-0.724859706	0.436348517	1
IPO5	0.419950295	0.731274572	1
SF3B1	0.359727608	0.749155103	1
FRMPD1	-1.872932523	0.388883937	1
RUFY4	0	1	1
CFL1	0.976929883	0.280105434	1
SRL	-0.531041702	0.692183772	1
MAP4	-0.966180317	0.551627979	1
GYS1	-1.001885328	0.332482174	1
MPRIP	-0.400658404	0.775092938	1
PINK1	0.039029861	0.977804068	1
PPP1R27	-0.379639786	0.765862667	1
PCBP2	0.48264831	0.712606102	1
MYL1	-1.419626019	0.388581056	1
SYK	0	1	1
PRDX1	1.144415952	0.262427659	1
BRD2	-0.198364404	0.827163449	1
UGP2	-1.133794445	0.211130277	1
USP24	0.376966476	0.773465435	1
FLII	-0.567749521	0.588721126	1
PGK1	-1.021963326	0.305738359	1
ACTN1	2.373068357	0.151478586	1
IRX4	0	1	1
RHOA	-0.178748477	0.841195801	1
RPS23	1.761019871	0.157125634	1
ETV5	1.976281032	0.104220844	1
CRACR2B	1.434122144	0.229170444	1
HNRNPU	0.372983898	0.757254594	1

IPO13	-0.930526051	0.415939011	1
PCBP1	-0.784714461	0.396092173	1
RBBP8NL	0	1	1
XIRP2	-0.20316467	0.861026493	1
ENSBTAG00000048733	1.055808143	0.377527883	1
SDHB	-0.8641658	0.372618406	1
SNRNP200	-0.259030719	0.783640259	1
GDI2	0.19429966	0.845283321	1
SREBF1	1.041463516	0.444085315	1
HECTD1	-0.727009502	0.418221517	1
PLPPR2	0	1	1
PDLIM5	0.496404478	0.805684577	1
APOE	1.611770817	0.165092824	1
TLN1	-0.368522019	0.756431685	1
EIF4A1	1.45759482	0.144287181	1
NCL	0.581646733	0.632183514	1
NUMA1	0.417738982	0.687282874	1
ACTN4	0.37539412	0.642196806	1
HSP90AB1	-0.230309015	0.794847042	1
ZNF592	-0.999227436	0.353003846	1
SPTAN1	0.624626244	0.735923215	1
RPL24	1.175368358	0.350927469	1
APP	2.0726195	0.11905421	1
GPAA1	-0.486066556	0.621805864	1
TMBIM6	0.593562771	0.54603424	1
MYH4	-1.637926612	0.32671451	1
SLC3A2	0.975999399	0.333528788	1
CYB5R1	0.317914935	0.825135315	1
TXLNB	-0.701606991	0.662685107	1
ST13	-0.052709219	0.960216108	1
VWA8	-0.113491401	0.917555122	1
PDK4	-0.919851708	0.427508415	1
EEF1B2	1.404097034	0.300396999	1
MYO18A	0.193769298	0.880502561	1
NDUFS1	-0.855879935	0.338975668	1
EIF3E	1.054582359	0.443799508	1
SPTB	-0.37487958	0.779667883	1
IGF2R	0.17558216	0.892491597	1
CRLF1	0	1	1
COL1A2	0.13267902	0.905758916	1
TUBA1C	0.544934589	0.598021895	1
OAZ1	-0.338493575	0.724707961	1
TBC1D30	0	1	1
CUL5	0.794502637	0.52984101	1
CTSA	0.796802675	0.441574077	1
FXR1	0.290880351	0.822841507	1
LRP1	-0.156346897	0.874303334	1
GNAS	-0.213394597	0.832338551	1
POR	1.109630378	0.156779559	1
ATP5F1C	-0.948474088	0.297069083	1
CAPN3	-0.625744555	0.606971217	1
CLCA2	-1.877632545	0.402149573	1
ATP5PO	-0.506570784	0.605282781	1
SLCO1A2	0	1	1
SQSTM1	-0.866111285	0.463932154	1
VDAC3	-1.105773365	0.324911907	1
ENSMMSG00000013664	2.865783834	0.155831209	1
MBD4	1.542918689	0.21934841	1

RPS27	1.951440087	0.107543682	1
ATP1A2	-0.316140828	0.81433246	1
TALDO1	1.488360322	0.118112703	1
GAPDH	-1.024936882	0.412564667	1
ANKRD23	-0.587779684	0.739175134	1
COX6A2	-0.792769299	0.43135624	1
TMSB10	0.816978368	0.42990072	1
COX7B	-0.524688463	0.574409524	1
IDH2	-1.034464257	0.31345697	1
AMPD1	-0.774563803	0.520211982	1
PLEKHA7	0	1	1
PRRC2A	0.496973516	0.596694529	1
HSP90B1	0.798922116	0.315627505	1
COL1A1	0.754846999	0.54296125	1
RPL36	0.961657128	0.426777521	1
EIF3CL	-0.217442017	0.819805642	1
PDHA1	-0.91329401	0.431462405	1
HSPA5	0.434008521	0.582618446	1
RPL31	1.403814048	0.206518997	1
NDUFA4	-0.752534397	0.426688879	1
SLC25A6	0.908036567	0.395409089	1
LAP3	1.283914325	0.260590419	1
RPS15	1.211239126	0.242410684	1
COL3A1	0.56582727	0.62892614	1
MYOZ2	0.413469437	0.785086623	1
PON2	1.512005224	0.189674945	1
CACNA1S	-0.530114307	0.688723192	1
G31754	0	1	1
HSPD1	0.171395216	0.85950197	1
RPS16	1.299634682	0.287888003	1
ENSMMSG00000008095	0	1	1
VDAC1	-0.925948228	0.391749491	1
ACTA2	1.148426184	0.217049461	1
KXD1	0.82797885	0.468912354	1
OGDH	-0.628856963	0.519285796	1
RPL37	0.899088469	0.44208259	1
RPL35	1.502440084	0.263021643	1
SPEG	-0.783702133	0.5112106	1
GOT2	-0.959982611	0.399201597	1
ELAPOR1	1.418110316	0.155740342	1
FAU	0.624804454	0.539718972	1
SYNPO2L	-0.31968655	0.796096318	1
UQCRC1	-0.862958874	0.446225748	1
ARHGAP1	4.737347933	0.244659348	1
IGFN1	-1.427871288	0.238671417	1
CANX	0.525034801	0.50411605	1
NDRG2	-1.082861417	0.321971923	1
TXNIP	-0.100078786	0.929338778	1
FLNB	1.435601586	0.195213839	1
MAP4	-0.052875765	0.963837177	1
PRPF8	-0.140103762	0.881287755	1
HSPA9	-0.309306183	0.735409472	1
CTSD	0.089566295	0.929397322	1
TMC3	0	1	1
OS9	0.857863329	0.349748415	1
DDX5	0.208561999	0.823892206	1
PPIA	1.779327714	0.1415128	1
UBR4	-0.089602659	0.925083441	1

ZNF185	0	1	1
LMOD2	0.095618364	0.957910401	1
C28H10ORF71	-0.182994922	0.91246233	1
HCN2	-0.379024199	0.690719846	1
CSDE1	0.610638481	0.630140602	1
RPL11	1.392461338	0.23863609	1
MYH11	1.381142491	0.129525468	1
CACNA1H	0	1	1
FLNA	0.383136012	0.824900849	1
RPS21	1.782280126	0.164826585	1
ACSL1	0.678653413	0.626024304	1
RPL13	0.839797551	0.438518482	1
NCAPD2	-1.178725622	0.323840003	1
EEF1D	1.316219351	0.327974387	1
TTN	-0.277333791	0.83598221	1
GPI	-0.840856687	0.407513381	1
RPS10	1.793408615	0.112110787	1
UQCRC2	-0.938891764	0.314970083	1
RPS5	1.382060816	0.221186474	1
EIF4B	0.882612153	0.417837474	1
SLC15A1	-1.880631666	0.410658181	1
APP	0.42919661	0.736861558	1
RPL28	1.743735606	0.162014117	1
CLTC	0.287180121	0.786190761	1
COX4I1	-0.649755215	0.515573186	1
ENSBTAG00000049190	-0.642424351	0.721119208	1
LAMP1	0.116503424	0.901918766	1
TPM1	-1.379388928	0.255811054	1
MSMB	-2.849132769	0.144820453	1
EIF4G1	-0.197850111	0.829266535	1
FMO1	1.304384661	0.304889725	1
RPS27A	0.898380268	0.470056528	1
COL6A1	0.66149263	0.495643138	1
PDIA3	1.052419129	0.179760288	1
RPS9	2.248612798	0.132142228	1
RPS25	0.584760747	0.630623578	1
MYLK2	-1.082767633	0.39804415	1
RPS12	1.638043916	0.16189157	1
ACADM	0.479842378	0.680481176	1
P4HB	0.489342957	0.611703952	1
ACER1	0	1	1
PRDX6	0.570219939	0.593906035	1
RPS11	0.782420598	0.498790039	1
SLC7A8	2.235234183	0.193320205	1
PSMD2	-0.573435604	0.552250347	1
COQ8A	-0.611113617	0.636886793	1
CUL4A	-0.816456292	0.421323557	1
SLC25A3	-0.938868465	0.433898338	1
GSN	-0.520661921	0.620430492	1
RPL18A	1.374648	0.293291123	1
FEM1A	-0.984808897	0.371774138	1
BIN1	-0.917654444	0.438355031	1
LONP2	0.503400248	0.589673915	1
RPLP2	1.614598254	0.177167053	1
SLC44A3	0	1	1
RPL14	1.003172064	0.435109099	1
PDE4DIP	-0.152855047	0.909428776	1
RPL29	0.367764296	0.744867087	1

RPL27	1.559634372	0.192195791	1
RPS17	1.49636022	0.191729883	1
MYH9	0.206196653	0.833581304	1
HSPA1B	-0.902341132	0.316122452	1
NFE2L1	-0.165893497	0.883362153	1
SVIL	0.0379666	0.983799336	1
HSPA1A	-0.81884302	0.368932482	1
VCP	-0.262558377	0.75983477	1
NDUFS2	-0.826095959	0.450373505	1
YBX1	-0.834935792	0.367196375	1
ALAS1	1.787839448	0.164995309	1
PGAM2	-1.275805053	0.27843554	1
MYH6	0.347068979	0.817398306	1
TMOD4	-1.145495044	0.350559452	1
HDLBP	0.168373072	0.861214622	1
ATP1A4	-0.621732898	0.642487102	1
PLEC	-0.865658845	0.835337692	1
KDF1	0	1	1
PLPPR2	0	1	1
IQANK1	-2.850303374	0.170004944	1
NNT	-1.025375867	0.339164464	1
B3GALT4	0.976648616	0.418343155	1
SYNPO	-0.047166674	0.970606974	1
AKR1C4	0	1	1
RPL12	1.111115435	0.378096312	1
PSAP	-0.179146493	0.872768057	1
ENSBTAG00000000835	0	1	1
RNF222	0.665386253	0.557618325	1
RPL18	1.894187156	0.124752612	1
ILDR1	0	1	1
RPL23A	0.883724806	0.415731134	1
MYL12A	0.856234127	0.563128733	1
RPS24	1.482987058	0.260031801	1
PABPC1	1.009621063	0.271830953	1
EIF4G2	-0.728883244	0.455563522	1
IRX5	0	1	1
PNMT	1.837614677	0.176253902	1
TPM1	-1.370063934	0.237249298	1
EMID1	0	1	1
RPS7	1.597308773	0.255919351	1
APLP2	1.204996493	0.242252205	1
ACADVL	0.634451461	0.627249377	1
FKBP4	0.839383559	0.364301158	1
SAT1	1.162439075	0.294052372	1
CKMT2	-1.013785992	0.26812427	1
ACO2	-0.912849121	0.38613463	1
RPL3	1.558487951	0.158717056	1
RPL27A	1.93300158	0.183471703	1
MGC151921	1.582855629	0.371400351	1
RPS14	1.27908904	0.275025109	1
TPI1	-1.175565058	0.244972084	1
TRPV4	-1.829852416	0.402438437	1
RPL9	1.544972323	0.223194158	1
MYH7	0.195435341	0.894511881	1
RPS3A	1.890595196	0.1577039	1
RPL8	1.004279401	0.477776097	1
MARVELD3	-1.824533413	0.444205503	1
OCRL	-1.877373462	0.401416501	1

SDHA	-0.965731641	0.357050668	1
RPS26	1.328534489	0.244816231	1
MARVELD3	0	1	1
PGM1	-1.245479765	0.27019895	1
MDH2	-0.854267927	0.418939235	1
ITM2B	0.859410573	0.329588238	1
RPL7	0.827048351	0.461617387	1
B4GALNT4	0	1	1
HECTD4	1.494993623	0.274903008	1
DYNC1H1	-0.040305998	0.969231062	1
RPS4X	1.635568934	0.208642808	1
EEF1G	1.206206431	0.368350764	1
ACTG1	0.295338476	0.791889997	1
HSP90AB1	-0.293765737	0.80712624	1
AP1M2	-1.827535566	0.419538448	1
SYNPO2	0.176220705	0.900996293	1
RPL19	1.802641679	0.183195824	1
RPL23	1.119633114	0.370386187	1
ACTB	0.165487342	0.870274924	1
RACK1	1.681804686	0.24484727	1
TNNI1	0.359893105	0.815444939	1
EIF4A2	-0.610145975	0.579910142	1
YBX3	-0.805266197	0.42925336	1
DOP1B	-1.872189285	0.386795519	1
LDHA	-1.403618657	0.246339734	1
KIF1C	-1.011603376	0.28692184	1
RPL10A	0.861140589	0.450392157	1
RPS8	2.32399377	0.107368511	1
RPL5	1.64298069	0.236002378	1
PYGM	-1.099095358	0.34425249	1
RPL13A	0.474840351	0.639874066	1
RPS6	1.511612461	0.224701813	1
HSPB1	1.37833525	0.341493444	1
RPLP1	1.672732018	0.177559139	1
MYH2	-1.056600138	0.401716335	1
CBS	0.494737248	0.892414094	1
MYOZ1	-1.137596508	0.295861121	1
RPL10	1.102646513	0.429314831	1
DST	-0.700874887	0.383628307	1
TTN	-0.42852546	0.794936172	1
PDLIM3	-0.853365206	0.515010705	1
PARP6	-1.181115033	0.318139623	1
TNNT3	-1.150376482	0.303689336	1
CSDE1	-0.903973055	0.627293038	1
ENSMMSG00000020610	0	1	1
BBS5	-0.040964698	0.980438243	1
RPL21	2.118772805	0.097778809	1
FHL1	0.094194741	0.948175772	1
TFAP2C	0	1	1
RBM47	0	1	1
UBC	0.365943132	0.787651589	1
RPLP0	2.279053874	0.110405156	1
MYL1	-1.330117668	0.207545094	1
LDB3	-0.227677233	0.853476348	1
ATP5F1A	-0.911214971	0.278167218	1
SLC25A4	-0.990994197	0.374671975	1
ATP2A2	0.212330349	0.88105834	1
ENSMMSG00000006245	0	1	1

AP1M2	0	1	1
SPTBN1	1.421982412	0.269802516	1
TPM1	-1.412714855	0.25620342	1
RPL7A	1.817912321	0.165631043	1
CMYA5	-0.798009071	0.465369145	1
NEK10	0	1	1
RPSA	2.217782517	0.109967335	1
HSP90AA1	-0.08519755	0.933206877	1
MAP7	0	1	1
TPD52L1	0	1	1
RYR1	-0.96583517	0.444447868	1
HSPA8	0.049952176	0.954650845	1
TCAP	-0.112830252	0.926752247	1
AOX4	0	1	1
IGSF5	-1.829056193	0.408126112	1
ATP5F1B	-0.936331123	0.247652891	1
VAV2	-2.848752989	0.137891153	1
MYOM1	0.067921689	0.96329421	1
UNG/ACACB	0.703868681	0.520941089	1
PFKM	-1.264917428	0.253055463	1
TPM3	0.411085441	0.792272428	1
LAMC2	0	1	1
RASGRP2	-1.145205835	0.521553133	1
MYOM2	-0.016305986	0.991060681	1
MYH7	0.235630811	0.874987892	1
RASGRP2	-1.009449754	0.387125372	1
ENO3	-1.25069134	0.291347881	1
TNK1	0	1	1
RPL4	2.011250419	0.157064669	1
EEF2	-0.156924192	0.901991687	1
MYH3	-1.052737029	0.420030109	1
ACTN3	-1.467479699	0.243862214	1
RPS2	1.929615799	0.187945034	1
TNNI2	-1.384499811	0.241426216	1
ACTN2	0.036743524	0.977379024	1
NRAP	-0.082705905	0.947147009	1
TRIM29	0	1	1
ESRP1	0	1	1
MB	-0.317786217	0.821557971	1
MYH7	0.120967144	0.93693074	1
ENSBTAG00000040028	-0.559799449	0.637572409	1
FAM83F	0	1	1
AAK1	-1.829621401	0.404068213	1
TNNC2	-1.410129038	0.251039985	1
GRHL2	0	1	1
LGALS7	-1.827518102	0.419635306	1
MYBPC2	-1.443357312	0.267065846	1
RYR1	-0.821468098	0.446268895	1
TNNT3	-2.507798921	0.273523752	1
NUMA1	1.801366515	0.476224361	1
RAP1GAP	0	1	1
DES	-0.256436064	0.851330414	1
GAPDH	-1.151691874	0.328548417	1
BSPRY	0	1	1
FLNC	0.035056293	0.980797103	1
ESRP2	0	1	1
MYLPF	-1.397023265	0.239437933	1
TMPRSS2	-1.829429046	0.405428288	1

SAO	0	1	1
MYBPC1	0.044598314	0.975671144	1
MYH6	0.302245047	0.847137558	1
TPT1	-0.784007144	0.437232569	1
TPM2	-0.282949089	0.840869592	1
ATP2A1	-1.29526287	0.259218924	1
CAMSAP3	0	1	1
EEF1A1	2.226058214	0.103476968	1
MARK3	0	1	1
TTN	-0.563766687	0.721945204	1
CD44	0	1	1
MYH1	-1.558564868	0.270004759	1
PPP4C	-1.190047859	0.286487551	1
MAPK13	-1.825289303	0.437604177	1
PDE7A	0	1	1
MYH1	-1.513601832	0.259613751	1
CKM	-0.826202804	0.4342398	1
NEB	-0.280536935	0.810363297	1
G6PD	0	1	1
ACTA1	-0.532206142	0.687931503	1
PTPRF	0	1	1
G7552	0	1	NA
LOC786706	0.72382101	0.873446051	NA
ROCK2	0.72382101	0.873446051	NA
C3H1ORF56	0	1	NA
G10812	0.47496572	0.91687955	NA
FLT4	0	1	NA
ENSMMSG00000011978	-1.80775858	0.691386399	NA
RPL10	0	1	NA
G1872	0	1	NA
IP6K2	0	1	NA
G4290	0	1	NA
MGAT4D	0.47496572	0.91687955	NA
RITA1	0.72382101	0.873446051	NA
G5279	0.72382101	0.873446051	NA
SLC22A9	0.72382101	0.873446051	NA
G8293	0	1	NA
IMP3	0	1	NA
LHX5	0	1	NA
G14111	0	1	NA
G19948	0	1	NA
STX8	0	1	NA
ZSWIM2	0.72382101	0.873446051	NA
G29169	0	1	NA
MYT1L	0	1	NA
ASCL4	0.72382101	0.873446051	NA
G34539	0	1	NA
CRYBA2	0.47496572	0.91687955	NA
NRG2	0.47496572	0.91687955	NA
HOXD11	-2.986828858	0.500787551	NA
SPAG6	-2.986828858	0.500787551	NA
ENSMMSG00000017840	-0.827697671	0.854869315	NA
IQCF3	-2.986828858	0.500787551	NA
ENSMMSG00000014462	-0.827697671	0.854869315	NA
DNMT3B	0	1	NA
ENSMMSG00000020435	-2.71530278	0.542516499	NA
RNF186	-2.986828858	0.500787551	NA
MSANTD2	0	1	NA

PALLD	0	1	NA
LRRC56	0	1	NA
LRRC56	0	1	NA
HASPIN	0.723831821	0.873444169	NA
G34010	0	1	NA
G27324	-2.986828858	0.500787551	NA
G28566	-0.827697671	0.854869315	NA
BEND6	0	1	NA
G1330	0	1	NA
P2RX2	0	1	NA
CCT5	0	1	NA
PDE11A	0	1	NA
NRXN3	0	1	NA
CYP3A24	0	1	NA
ENSMMSG00000012212	0	1	NA
ENSMMSG00000020063	0	1	NA
G4651	2.08840461	0.641773357	NA
NEIL2	-2.71530278	0.542516499	NA
G5319	0	1	NA
G5502	0	1	NA
G5681	0.723835332	0.873443558	NA
ENSMMSG00000016582	1.660998231	0.712884489	NA
CFAP77	0	1	NA
DLX2	0	1	NA
GAST	0.723847352	0.873441466	NA
G8659	0	1	NA
CFAP99	0	1	NA
HHLA1	0	1	NA
G10529	0	1	NA
REG3G	0	1	NA
XKR4	0	1	NA
ST6GALNAC1	0	1	NA
SHH	2.08840461	0.641773357	NA
CLSTN1	0	1	NA
OR6V1	0	1	NA
MPPED1	0	1	NA
CACNA2D4	2.08840461	0.641773357	NA
IGF2BP1	0	1	NA
PSMD4	0	1	NA
G14250	0	1	NA
VAT1L	0.987015346	0.827822394	NA
GUCY1B2	-2.71530278	0.542516499	NA
KCNE1	0	1	NA
CYP4A11	0	1	NA
AKR1C4	2.08840461	0.641773357	NA
UOX	0	1	NA
ENSBTAG00000050745	0.987015346	0.827822394	NA
G18997	0.723831821	0.873444169	NA
PPIA	0	1	NA
DNAJC21	0	1	NA
GCM1	2.08840461	0.641773357	NA
G20230	0	1	NA
CDH1	0	1	NA
ENSBTAG00000019009	0	1	NA
G21086	0	1	NA
G21113	0	1	NA
ENSMMSG00000006764	1.660998231	0.712884489	NA
ENSMMSG00000006708	0.987015346	0.827822394	NA

LCN6	0	1	NA
MAT1A	0	1	NA
TMEM179	0	1	NA
FOXB1	0.987015346	0.827822394	NA
DC100299481/LOC10084772	0.987015346	0.827822394	NA
ENSMMSG00000010804	0	1	NA
DNAI2	0	1	NA
TRIM10	2.08840461	0.641773357	NA
G26243	0	1	NA
CCDC182	0.987015346	0.827822394	NA
LOC518437	0	1	NA
ENSMMSG00000016357	0	1	NA
TBX19	0.723835332	0.873443558	NA
CENPA	0	1	NA
G27497	0	1	NA
PTRHD1	0	1	NA
MUC1	0	1	NA
ENSMMSG00000009586	0	1	NA
TMEM132B	0	1	NA
TOMM20	0.987015346	0.827822394	NA
DUSP21	0.723832524	0.873444047	NA
ZFYVE21	0.987015346	0.827822394	NA
SPATC1	0	1	NA
GJA3	0	1	NA
MKRN1	0	1	NA
RIPPLY3	0	1	NA
BMP8B	0	1	NA
GAPDH	0	1	NA
RPL18A	0	1	NA
ELOF1	0	1	NA
LHX4	-2.71530278	0.542516499	NA
CLDN6	0	1	NA
LIM2	0	1	NA
G36096	2.08840461	0.641773357	NA
KCNAB1	0	1	NA
WNT8A	0	1	NA
ENSBTAG00000012971	2.08840461	0.641773357	NA
PAX5	2.694973201	0.545807612	NA
ARC	-0.827682593	0.854871919	NA
SRRM4	2.694973201	0.545807612	NA
SHANK1	0	1	NA
G24089	-3.575751954	0.416447992	NA
SLC34A1	-3.575751954	0.416447992	NA
ANLN	2.229037484	0.618927501	NA
G8241	0	1	NA
KCNK13	0.723847097	0.87344151	NA
KCNMB2	0	1	NA
SCN10A	-3.575751954	0.416447992	NA
LRRFIP1	0.723852567	0.873440558	NA
SLC12A3	-3.575751954	0.416447992	NA
G34561	2.334074369	0.602086526	NA
FFAR3	-0.827685401	0.854871434	NA
ARHGAP33	-1.958812414	0.666348701	NA
WNT9B	-3.575751954	0.416447992	NA
SLC10A2	-3.575751954	0.416447992	NA
SERPINB7	-3.575751954	0.416447992	NA
SLAMF1	-1.958812488	0.666348689	NA
ENSBTAG00000051963	0	1	NA

ADARB2	-3.575751954	0.416447992	NA
ADAMTS14	0	1	NA
PPP2R1A	-0.564508997	0.900587874	NA
LOC100300510	0	1	NA
KCNE5	-3.575751954	0.416447992	NA
ENSMMSG00000013209	0	1	NA
GPR55	0	1	NA
GAS2L2.L	2.229037484	0.618927501	NA
SERPINA10	0	1	NA
ISG12(B)	2.694973201	0.545807612	NA
FCRLA	0.723847097	0.87344151	NA
G1272	0	1	NA
RAB38	0	1	NA
G2034	1.349340976	0.765676006	NA
AQP2	1.349340976	0.765676006	NA
GRID1	0	1	NA
PTCHD1	2.694973201	0.545807612	NA
G3925	0	1	NA
SLC27A2	0	1	NA
TBXT	0	1	NA
CYTL1	0	1	NA
RRM2	0	1	NA
PRPS1	2.694973201	0.545807612	NA
G6857	1.349340976	0.765676006	NA
NOG	-0.676599138	0.881425681	NA
SLC25A48	0	1	NA
G7829	0	1	NA
GOLPH3	0	1	NA
SYNDIG1	0	1	NA
PAX5	1.349340976	0.765676006	NA
RNF182	1.349340976	0.765676006	NA
HAND1	0	1	NA
G10204	1.349340976	0.765676006	NA
G10220	1.349340976	0.765676006	NA
ENSMMSG00000009247	2.694973201	0.545807612	NA
DEUP1	0	1	NA
OPRK1	0	1	NA
ENSBTAG00000050336	-3.295723596	0.455451161	NA
FGF23	0	1	NA
CCDC105	0	1	NA
G13863	0	1	NA
BSX	0	1	NA
TMPRSS5	2.229037484	0.618927501	NA
CRYBB3	2.694973201	0.545807612	NA
CLIC6	0	1	NA
TMED2	1.349340976	0.765676006	NA
CTNNA2	1.349340976	0.765676006	NA
NR2E3	0	1	NA
CCDC175/RTN1	0	1	NA
NEPN	0	1	NA
IFNAC	0	1	NA
LRRN4	1.349340976	0.765676006	NA
TMEM9	1.349340976	0.765676006	NA
KCNS2	0	1	NA
UGT3A2	0	1	NA
ENSMMSG00000001237	0	1	NA
G22963	0	1	NA
ENSMMSG00000006136	0	1	NA

SFTPA1	0	1	NA
ENSMMSG00000022956	0	1	NA
ENSMMSG00000017283	0	1	NA
IL13	0	1	NA
CRYGN	0	1	NA
BOVIN	0	1	NA
495184/LOC101732638/LO(	2.334074369	0.602086526	NA
TRIM15	1.349340976	0.765676006	NA
CLEC2A	0	1	NA
SPOP	1.349340976	0.765676006	NA
RPSA	0	1	NA
ENSMMSG00000013503	1.349340976	0.765676006	NA
PGC	0	1	NA
G27401	0	1	NA
G28154	0	1	NA
ADD2	0	1	NA
G29184	0	1	NA
G29335	0	1	NA
OCSTAMP	0	1	NA
G31526	0	1	NA
MRAP	0	1	NA
TPM3	0	1	NA
SLC1A7	0	1	NA
STPG1	0	1	NA
ENSMMSG00000012830	0	1	NA
CYP2C87	2.694973201	0.545807612	NA
G33984	0	1	NA
G34346	-3.295723596	0.455451161	NA
APEX1	1.349340976	0.765676006	NA
AXDND1	0	1	NA
PGRMC2	0	1	NA
TEKT5	0	1	NA
PRM2	0	1	NA
FAM83E	2.694973201	0.545807612	NA
CRYBB2	0	1	NA
G36396	0	1	NA
SAXO1	0	1	NA
CYP4A25	0	1	NA
KRT83	-1.958802096	0.666350352	NA
ENSMMSG00000021793	0	1	NA
PLPP4	-3.992835228	0.362230715	NA
SPACA7	-3.992835228	0.362230715	NA
GAP43	-3.992835228	0.362230715	NA
FFAR4	2.646032747	0.553287698	NA
UXS1	0	1	NA
CBLN2	-3.992835228	0.362230715	NA
APRT	-0.676579095	0.881429154	NA
ENSMMSG00000008192	-3.992835228	0.362230715	NA
LOC525599	-3.992835228	0.362230715	NA
PPYR1	-3.992835228	0.362230715	NA
BTN1A1	1.658983002	0.713222293	NA
TPM1	-0.491099588	0.910344967	NA
VDAC3	-3.926347187	0.370556977	NA
CNTD1	0	1	NA
ENSBTAG00000054949	0.987028851	0.827820064	NA
NKX3-2	-3.992835228	0.362230715	NA
FKBP6	-3.992835228	0.362230715	NA
ENSBTAG00000049518	-3.992835228	0.362230715	NA

PACRG	-3.992835228	0.362230715	NA
G4852	0	1	NA
F12	-3.708363428	0.398699846	NA
LOC509881	-1.958807421	0.6663495	NA
SPRYD7	-0.212056769	0.962487851	NA
SERPINB6	-3.992835228	0.362230715	NA
LSM2	-3.992835228	0.362230715	NA
G223	0	1	NA
LOC615853	0	1	NA
SLC25A45	-3.708363428	0.398699846	NA
PPP1R32	2.646032747	0.553287698	NA
ENSMMSG00000006742	0	1	NA
SERPINA3-1	0	1	NA
NMD3	3.121031177	0.48286634	NA
ASCL1	0	1	NA
GAD2	0	1	NA
ENSMMSG00000022495	0	1	NA
G2836	0	1	NA
LIMS1	0	1	NA
G3203	0	1	NA
G3209	0	1	NA
G3211	0	1	NA
G3250	0	1	NA
NXNL1	2.646032747	0.553287698	NA
G3398	0	1	NA
GALNTL6	0	1	NA
G4609	0	1	NA
SHEEP	0	1	NA
ENSBTAG00000049741	0	1	NA
SYT10	0	1	NA
EPYC	2.646032747	0.553287698	NA
C22H3ORF20	1.658983002	0.713222293	NA
C1QTNF8	0	1	NA
MSLN	0	1	NA
CDH12	3.121031177	0.48286634	NA
KCNS1	0	1	NA
KCNK15	3.121031177	0.48286634	NA
CARD14	0	1	NA
BECN1	2.156250555	0.630710498	NA
NR0B1	-3.708363428	0.398699846	NA
PAX5	0	1	NA
LOC508459	3.121031177	0.48286634	NA
GRID2	0	1	NA
ENSMMSG00000012326	3.121031177	0.48286634	NA
CLRN2	0	1	NA
PRLH	0	1	NA
BTBD17	2.646032747	0.553287698	NA
GLRA1	3.121031177	0.48286634	NA
ENSBTAG00000037632	0	1	NA
TMEM121B	0	1	NA
G12690	0	1	NA
SPTLC3	1.658983002	0.713222293	NA
SLC6A11	0	1	NA
G14274	3.121031177	0.48286634	NA
DMRTA1	0	1	NA
OSM	0	1	NA
G14911	3.121031177	0.48286634	NA
LOC516849	0	1	NA

CLRN3	1.658983002	0.713222293	NA
TRHDE	3.121031177	0.48286634	NA
TAS1R2	0	1	NA
G6PC2	0	1	NA
LOC618985/LOC781778	0.987026468	0.827820476	NA
G17389	0	1	NA
ENSMMSG00000021204	0	1	NA
RBP3	0	1	NA
ACR	-3.708363428	0.398699846	NA
ENSMMSG00000005265	3.121031177	0.48286634	NA
ENSMMSG00000003958	0.987024218	0.827820864	NA
HAPLN1	1.658983002	0.713222293	NA
IGKV2D-29	0	1	NA
IFNW1	0	1	NA
G20793	0	1	NA
G21043	0	1	NA
ENSMMSG00000002563	0	1	NA
UPK1A	1.658983002	0.713222293	NA
G21310	0	1	NA
1700020L24RIK	0.987024218	0.827820864	NA
NOL4	0	1	NA
EIF3C	1.658983002	0.713222293	NA
FABP6	0	1	NA
ENSMMSG00000005068	0	1	NA
G24504	0	1	NA
RPL36	0	1	NA
ENSMMSG00000019522	1.658983002	0.713222293	NA
ENSBTAG00000052892	0	1	NA
ENSBTAG00000053701	0	1	NA
G25673	0	1	NA
CHRNA4	3.121031177	0.48286634	NA
KCNK18	0	1	NA
G26483	1.658983002	0.713222293	NA
PLG	1.658983002	0.713222293	NA
KCNK16	0	1	NA
ENSBTAG00000012748	3.121031177	0.48286634	NA
GABRA3	0	1	NA
GPA33	0	1	NA
ENSBTAG00000048343	1.658983002	0.713222293	NA
TF	3.121031177	0.48286634	NA
GRIK1	2.145590513	0.632443691	NA
ENSBTAG00000037933	0	1	NA
CCKBR	0	1	NA
AOC3	0	1	NA
HBM	0	1	NA
G29427	2.857491968	0.521329411	NA
NCBP2	2.088419155	0.641770977	NA
ALLC	2.646032747	0.553287698	NA
DPEP1	2.646032747	0.553287698	NA
NDUFB8	2.088422606	0.641770413	NA
MRPL53	0	1	NA
SHROOM3	0	1	NA
PCARE	2.646032747	0.553287698	NA
FMN2	1.658983002	0.713222293	NA
UBE2R2	3.121031177	0.48286634	NA
BMP8B	1.658983002	0.713222293	NA
G33214	0	1	NA
HS6ST2	0	1	NA

ADGRE3	-3.708363428	0.398699846	NA
SPAG17	0	1	NA
G34768	1.658983002	0.713222293	NA
ENSMMSG00000004216	0.987024218	0.827820864	NA
MAT1A	0	1	NA
CNNM1	0	1	NA
TMEM114	0	1	NA
ALPI	0	1	NA
NCOA4	-2.715290124	0.542518382	NA
LSAMP	1.658983002	0.713222293	NA
SLC6A19	0	1	NA
CPN1	0	1	NA
ABCC2	-1.584128364	0.721270803	NA
EEF2	-2.856263636	0.520642965	NA
PLPPR5	-4.316017102	0.323488428	NA
MASP1	0	1	NA
GATA6	1.658988709	0.713221336	NA
CACNA1A	1.349352876	0.765673971	NA
CTBP2	-0.202184988	0.964229133	NA
G38085	0	1	NA
MID2	1.944533816	0.665473865	NA
G640	0	1	NA
ENSMMSG00000015745	0	1	NA
FAM166C	0	1	NA
EVX2	3.449657288	0.437153403	NA
ENSMMSG00000011131	3.449657288	0.437153403	NA
CYP24A1	1.944533816	0.665473865	NA
ENSMMSG00000012449	1.3493555	0.765673523	NA
G4930	-4.028800939	0.357777226	NA
G5336	0	1	NA
STK32B	0	1	NA
G6541	-3.295695538	0.455454998	NA
TUBAL3	0	1	NA
FMN2	0	1	NA
UNC5C	3.449657288	0.437153403	NA
G10915	0	1	NA
G11571	0	1	NA
GPR15	1.944533816	0.665473865	NA
G12384	1.944533816	0.665473865	NA
PRMT8	1.944533816	0.665473865	NA
LGI3	0	1	NA
DDN	3.449657288	0.437153403	NA
G13712	0	1	NA
CFAP100	0.987028733	0.827820085	NA
ATP6V0D2	0	1	NA
CIBAR2	2.088436401	0.641768156	NA
GRIN2B	0	1	NA
G16980	0	1	NA
SKOR1	0	1	NA
RPS13	0	1	NA
SORCS1	1.944533816	0.665473865	NA
ST6GALNAC5	0	1	NA
ENSBTAG00000021432	2.088423712	0.641770232	NA
PPY	0	1	NA
G19884	0	1	NA
MAP3K19	0	1	NA
KRT4	-0.676569023	0.881430899	NA
G20584	0	1	NA

CLEC2L	0	1	NA
ESR2	0	1	NA
RRAGC	1.944533816	0.665473865	NA
G22611	0	1	NA
G24647	0	1	NA
NXPH2	0	1	NA
KCNQ2	0	1	NA
ENSBTAG00000014397	3.449657288	0.437153403	NA
G26735	0	1	NA
PLG	3.449657288	0.437153403	NA
KCNK17	0	1	NA
CLIC5	0	1	NA
ENSMMSG00000003093	2.969071246	0.504854692	NA
ENSMMSG00000012019	0	1	NA
PHYHIPL	0	1	NA
RXFP4	1.944533816	0.665473865	NA
G28724	0	1	NA
ENSBTAG00000005609	1.944533816	0.665473865	NA
G29166	0	1	NA
TOX3	0	1	NA
H2AC1	0	1	NA
G30137	0	1	NA
NPY	2.229054675	0.618924729	NA
RPL38	0	1	NA
CLIC6	3.449657288	0.437153403	NA
TADA2B	0	1	NA
C1ORF232	1.944533816	0.665473865	NA
HNRNPA1	0	1	NA
HDAC3	2.088423712	0.641770232	NA
ENSMMSG00000017963	0	1	NA
ENSMMSG00000015249	-4.028800939	0.357777226	NA
ABCC6	0	1	NA
ARL14EPL	2.088436401	0.641768156	NA
PTPN5	0	1	NA
AQP9	0	1	NA
NXNL2	0.723860687	0.873439145	NA
PPP2R1A	0	1	NA
CNTNAP2	-4.57989767	0.293986127	NA
SERPINA5	-4.57989767	0.293986127	NA
SLC12A8	3.717192258	0.401866625	NA
TTC21A	0	1	NA
ENSMMSG00000015057	-4.57989767	0.293986127	NA
CACNG4	-4.57989767	0.293986127	NA
PHOX2A	0	1	NA
FGF17	1.349357623	0.76567316	NA
G10980	0.474986097	0.916875986	NA
MOV10L1	0	1	NA
TCF23	3.546236016	0.424211927	NA
RPS7	0.109481106	0.980566632	NA
MRO	3.717192258	0.401866625	NA
NPPB	0	1	NA
LOXHD1	0	1	NA
SYNDIG1L	2.646047589	0.553285422	NA
G3767	2.196266521	0.624221409	NA
IDO2	3.232816867	0.467030049	NA
HAO2	3.717192258	0.401866625	NA
CYP19A1	3.132598419	0.481214184	NA
DNAH11	3.717192258	0.401866625	NA

STOX1	0	1	NA
ELFN2	0	1	NA
EF1A	0	1	NA
ADORA3/TMIGD3	0.987034851	0.827819029	NA
MYO1H	1.944531737	0.66547421	NA
GPR142	0	1	NA
GNRH1	0	1	NA
OSTC	0	1	NA
EN2	3.35338059	0.450279702	NA
TAS2R40	3.717192258	0.401866625	NA
ENSMMSG00000022095	0	1	NA
G14986	0	1	NA
LIPH	0	1	NA
PRSS1	0	1	NA
GRM3	3.232816867	0.467030049	NA
RGS8	0	1	NA
FABP1	2.088430788	0.641769074	NA
ENSMMSG00000008649	2.196266521	0.624221409	NA
DNAH6	3.717192258	0.401866625	NA
BARHL1	3.717192258	0.401866625	NA
ENSMMSG00000003901	2.145605658	0.632441227	NA
G25819	0	1	NA
CCT5	0	1	NA
PRPH2	0	1	NA
GNG4	0	1	NA
ENSBTAG00000031377	2.196266521	0.624221409	NA
CNGB1	0	1	NA
CAPN8	0	1	NA
BCAN	0	1	NA
G28852	2.694977141	0.545807011	NA
LCP1	0.474993043	0.916874771	NA
LOC100847115	0	1	NA
SMPDL3B	0	1	NA
ENSMMSG00000012084	0	1	NA
PCDH15	2.196266521	0.624221409	NA
CRP	0	1	NA
FRMD7	0	1	NA
GRIK2	3.717192258	0.401866625	NA
DPM3	0	1	NA
LOC101907227	0	1	NA
NYX	0	1	NA
HTR5B	3.121040769	0.482864969	NA
NANOS2	0	1	NA
G35825	0	1	NA
IFNW1	0	1	NA
RCVRN	3.717192258	0.401866625	NA
CUBN	0	1	NA
ENSMMSG00000019035	0	1	NA
LHFPL3	-3.99281585	0.362233048	NA
G10632	0	1	NA
CCDC170	3.94280882	0.37349413	NA
ENSMMSG00000010221	-2.23617935	0.606899398	NA
ENSMMSG00000007418	-0.047400617	0.991167451	NA
IHH	0	1	NA
KIF17	2.41021185	0.59000416	NA
LOC789612	-1.509447893	0.72861455	NA
LOC536660	3.089374203	0.487403679	NA
OXER1	-3.575732563	0.416450519	NA

AGPAT4	0	1	NA
ASPHD1	2.540145595	0.569638403	NA
KATNAL2	0	1	NA
PCDH8	-4.512478834	0.301341844	NA
GPR158	0	1	NA
G6748	3.94280882	0.37349413	NA
ATOH1	0	1	NA
ENSBTAG00000054184	0	1	NA
GBX2	0	1	NA
RPS10	0	1	NA
LOC112443425	0	1	NA
EN2-A	2.41021185	0.59000416	NA
G11531	0	1	NA
SYCN	0	1	NA
G12843	0	1	NA
GHRHR	0	1	NA
ENSMMSG00000002326	0	1	NA
MS4A10	0	1	NA
G15454	0	1	NA
G15484	0	1	NA
LRRC10	0	1	NA
TMBIM7	0	1	NA
GDF2	0	1	NA
H2BE1	0	1	NA
G19937	0	1	NA
CCDC177	0	1	NA
LOC617141	0	1	NA
KCNJ16	0	1	NA
LOC531152	0	1	NA
JAKMIP1	0	1	NA
TEKT2	0	1	NA
MBOAT4	0	1	NA
BHLHE22	0	1	NA
ENSMMSG00000021444	0	1	NA
NLRP14	0	1	NA
ENSMMSG00000002226	0	1	NA
ARID3C	0	1	NA
COLEC11	0	1	NA
ST18	0	1	NA
G29691	0	1	NA
LCT	0	1	NA
HEPACAM	0	1	NA
LOC112445104	0	1	NA
G34294	3.94280882	0.37349413	NA
G34867	0	1	NA
CLDN22	0	1	NA
SNAP25	0	1	NA
SLC15A2	0	1	NA
G37469	0	1	NA
G37575	2.41021185	0.59000416	NA
MYO7A	0	1	NA
STRA8	-0.828907005	0.84238182	NA
MDK/LOC618377	-0.423244235	0.922623912	NA
SCN10A	0	1	NA
TTLL9	-4.704566192	0.280711481	NA
GPR12	-4.996005676	0.251322394	NA
PSMD4	-3.992793001	0.362235795	NA
PDZRN3	4.137885829	0.350000309	NA

CALHM6	-1.958788649	0.666352504	NA
CHIC1	3.121053533	0.482863144	NA
CRMP1	-1.855644063	0.674478906	NA
SLC22A20P	0	1	NA
CCNO	3.551525827	0.423509688	NA
SERPINA6	4.137885829	0.350000309	NA
G1519	3.648704202	0.410731617	NA
PROX2	1.658997625	0.713219842	NA
SLC6A15	0	1	NA
ATP12A	0	1	NA
FOXI1	0	1	NA
TTLL2	3.648704202	0.410731617	NA
G5911	0	1	NA
GLYATL3	0	1	NA
SMIM32	1.944552851	0.665470708	NA
LBP	1.65900334	0.713218884	NA
RBFOX3	4.084552949	0.356327197	NA
CACNB4	3.551525827	0.423509688	NA
IGFBPL1	0	1	NA
NNMT	0	1	NA
KCNA1	0	1	NA
GPR179	3.720927294	0.401386527	NA
ORAI2	1.659002592	0.713219009	NA
STOML3	3.648704202	0.410731617	NA
G13866	0	1	NA
CCDC42	4.137885829	0.350000309	NA
G14015	0	1	NA
XCR1	0	1	NA
ATP6V0D2	0	1	NA
CDH18	2.596286848	0.560941158	NA
C17ORF114	0	1	NA
RHOC	0	1	NA
SHISA7	0	1	NA
XKR5	0	1	NA
FAM187A	-2.715278651	0.542520086	NA
MINDY4B	4.137885829	0.350000309	NA
SLC7A3	0	1	NA
FAT3	2.596286848	0.560941158	NA
KIF20A	0.987038329	0.827818429	NA
ZIC1	2.646057164	0.553283954	NA
ENSMMSG00000003059	-3.708340993	0.398702699	NA
IFNAC	0	1	NA
GATA5	4.029305727	0.362957587	NA
CASTOR2	1.658997625	0.713219842	NA
PPP3R2	0	1	NA
ERICH3	4.137885829	0.350000309	NA
SLC39A4	2.196247082	0.624224555	NA
FCRL6	0	1	NA
PIP5KL1	0	1	NA
ENSMMSG00000003741	0	1	NA
ONECUT2	1.659001662	0.713219165	NA
SHC3	0	1	NA
OR2I1P	0	1	NA
SLC6A19	0	1	NA
ENSMMSG00000016912	-4.802886498	0.270540601	NA
ANKRD63	-3.992799296	0.362235038	NA
TMPRSS7	-0.135857365	0.974527295	NA
SLC14A2	-2.307171241	0.590071437	NA

RNF32	0	1	NA
GSC	1.661029328	0.712879277	NA
SAMD4B	0.107472183	0.980923497	NA
CDKN3	0	1	NA
FMNL1	0	1	NA
C28H10ORF99	-3.575715348	0.416452799	NA
TESC	0	1	NA
UPB1	-4.316003008	0.32348999	NA
COX6B1	0.109482774	0.980566336	NA
CRYGA	4.309700317	0.330111798	NA
PCDH11X	0	1	NA
KCNG3	3.942823211	0.373492361	NA
LHFPL4	0	1	NA
QRFP	2.761000095	0.53579508	NA
DNA2	0	1	NA
SSTR3	0	1	NA
LCT	0	1	NA
MAGEB1	0	1	NA
RPH3A	4.309700317	0.330111798	NA
CTNNB1	-1.588526404	0.710033798	NA
ENSBTAG00000031362	0.723865121	0.873438373	NA
BNC1	0	1	NA
G14745	3.640778502	0.411765032	NA
ENSBTAG00000035945	2.761000095	0.53579508	NA
G16416	-4.874052951	0.263341894	NA
KSR2	2.196280601	0.62421913	NA
RFX6	0	1	NA
ENSBTAG00000027274	0	1	NA
GPR84	0	1	NA
G20963	0	1	NA
SH2D6	3.121054391	0.482863022	NA
RIMS2	0	1	NA
PAX1	4.309700317	0.330111798	NA
FXYD6	1.659011954	0.71321744	NA
ENSBTAG00000053785	3.121050934	0.482863516	NA
C1H3ORF80	0	1	NA
SLC6A5	-4.028781548	0.35777954	NA
NLRP5	0	1	NA
ETV3L	0	1	NA
EGR4	0	1	NA
NR5A1	0	1	NA
JPH3	3.945026128	0.373221661	NA
IFNW1	0.987035149	0.827818978	NA
NALCN	4.309700317	0.330111798	NA
DCAF12L2	0	1	NA
G34866	0	1	NA
TVP23A	0	1	NA
SHISA9	0	1	NA
LOC785933	0	1	NA
CDH20	0	1	NA
WDR55	0	1	NA
PCSK1	0	1	NA
ME3	2.761000095	0.53579508	NA
TMEM89	3.449702963	0.437147228	NA
NTNG2	0	1	NA
TSNARE1	2.196283524	0.624218657	NA
ENSMMSG00000003543	0.677521354	0.879319294	NA
GAS8	0	1	NA

TACR1	-4.579878279	0.293988171	NA
DNM1	-5.025704602	0.248455479	NA
HSPB9	-2.986791925	0.500792881	NA
C1QTNF12	-3.992803059	0.362234582	NA
NAA40	-0.906581558	0.831304946	NA
KCNQ4	2.908745863	0.513727674	NA
DLK1	-5.318577472	0.221429239	NA
VAT1L	2.908745863	0.513727674	NA
ENSMMSG00000004151	1.659001078	0.713219263	NA
G22282	0	1	NA
ENSBTAG00000011144	-3.295698131	0.455454605	NA
DNAJC22	0	1	NA
CIB4	0	1	NA
HAPLN4	0	1	NA
PRSS38	2.69497866	0.54580678	NA
G4955	1.661024077	0.712880157	NA
G5670	2.410197667	0.590006401	NA
NKX1-1	0	1	NA
GNAL	1.349361099	0.765672565	NA
UNC5C	2.94481513	0.508412701	NA
G10451	0	1	NA
G10888	0	1	NA
RPL12	0	1	NA
IRS4	0	1	NA
G14561	1.659002665	0.713218997	NA
ENSBTAG00000048777	0	1	NA
RGS17	0	1	NA
ACTL8	0	1	NA
CCDC81	4.137900151	0.34999862	NA
ENSMMSG00000009931	0	1	NA
MYH3	0	1	NA
TMEM244	2.694983166	0.545806094	NA
CD6	3.456072605	0.436286706	NA
PTPN2	2.722442302	0.485645995	NA
ODF3L1	2.088439159	0.641767705	NA
KRT76	3.971117458	0.370024842	NA
IGKV3-11	0	1	NA
BOVIN	3.12105636	0.48286274	NA
DOCK5	0.987035048	0.827818995	NA
SPACA9	0	1	NA
BRINP2	0	1	NA
CALHM3	2.908745863	0.513727674	NA
RSPO4	0	1	NA
C5H12ORF56	2.69497866	0.54580678	NA
G27732	0.475005387	0.916872612	NA
BRINP3	2.596295222	0.560939866	NA
JPH3	4.463225052	0.312979117	NA
GPATCH3	0.270489565	0.949421551	NA
ADORA2B	2.760997367	0.535795491	NA
CYP2C18	0	1	NA
LHX4	3.942827474	0.373491837	NA
OPN1LW	-1.67694858	0.697456102	NA
ENSBTAG00000015045	0	1	NA
G36063	0	1	NA
PTPRN	2.088435577	0.641768291	NA
CFAP65	0	1	NA
ENSBTAG00000053811	0	1	NA
G38201	2.908745863	0.513727674	NA

PROZ	-5.456317144	0.209488396	NA
ENSMMSG00000020529	4.601967146	0.298014455	NA
ENSBTAG00000009266	1.349363626	0.765672133	NA
SLC25A2	2.41023351	0.590000738	NA
RPGRIP1L	2.857517141	0.521325663	NA
ATP1B4	0	1	NA
OTOGL	-3.992809288	0.362233834	NA
CAMP	2.410228697	0.590001499	NA
PPM1E	3.04268054	0.494138074	NA
ENSMMSG00000022994	1.696233348	0.678574136	NA
ENSBTAG00000055058	2.196291601	0.62421735	NA
KDM4D	-1.855628119	0.674481514	NA
SERPINA3-8	2.410228697	0.590001499	NA
GRK1	4.10879355	0.353442541	NA
KCND2	0	1	NA
KHDRBS2	0	1	NA
MIS12	3.546255342	0.42420936	NA
RIMS1	0	1	NA
HBZ	0	1	NA
ENSMMSG00000001019	0	1	NA
GRPR	0	1	NA
AGRP	4.078337288	0.357069278	NA
AMBN	4.601967146	0.298014455	NA
G10760	3.551544551	0.423507203	NA
LOC522334	0	1	NA
CYP4F2	0	1	NA
PHEX	3.942830493	0.373491466	NA
GALNT5	3.121054439	0.482863015	NA
ENSMMSG00000006347	3.299815477	0.457679071	NA
ZSCAN9	0	1	NA
G20715	2.90873836	0.513728783	NA
ANOS1	2.744956137	0.522043341	NA
ENSMMSG00000012385	0	1	NA
DNAH6	3.468467644	0.434614994	NA
KCNA4	0	1	NA
SALL3	4.137899721	0.34999867	NA
SMIM43	4.601967146	0.298014455	NA
ENSBTAG00000046739	3.884293964	0.380729557	NA
CORIN	3.251592734	0.46439881	NA
ENSMMSG00000002172	0	1	NA
ALDH1L1	1.944560268	0.665469478	NA
IL1RL1	1.661020724	0.712880719	NA
EOMES	0	1	NA
G32001	2.596302515	0.56093874	NA
GLRA1	0	1	NA
RLN3	2.761003413	0.535794579	NA
TMEM217	2.088436568	0.641768128	NA
G37320	3.636391573	0.412337703	NA
ENSBTAG00000003640	-5.58204628	0.199009489	NA
G22635	-1.12319057	0.792185239	NA
GCFC2	3.456075299	0.436286343	NA
QRFPR	1.141276103	0.788693955	NA
NKX6-3	4.373820296	0.322882964	NA
TRBV12-3	-1.230136055	0.779842601	NA
G29177	2.694998784	0.545803714	NA
TRIM67	3.165225361	0.476570739	NA
COLEC11	-3.992801126	0.362234818	NA
ENSMMSG00000019757	-4.315985428	0.323491979	NA

DISP3	0	1	NA
G100	0	1	NA
ENSMMSG00000001746	0	1	NA
CTNND2	1.659004781	0.713218642	NA
KRT40	0	1	NA
DBX2	2.196292669	0.624217177	NA
G3662	0	1	NA
BPIFB6	0	1	NA
ENSMMSG00000002818	0	1	NA
CPN2	0	1	NA
IGFBP1	0	1	NA
ZYG11A	3.83591929	0.386776417	NA
TNFSF11	2.19628768	0.624217985	NA
G11733	2.196289209	0.624217737	NA
LOC112449027	-5.288206037	0.224127807	NA
ICA1L	0	1	NA
G16568	2.875362907	0.518672351	NA
G17092	2.334111224	0.602080651	NA
TAC1	0	1	NA
LOC518623	3.121060066	0.48286221	NA
TPM4	0	1	NA
CPLX3	3.165225361	0.476570739	NA
ENSMMSG00000015665	0	1	NA
BFSP1	2.088436045	0.641768214	NA
ENSBTAG00000054224	0	1	NA
G23464	3.353401884	0.450276774	NA
SH2D4B	0	1	NA
POLR1H	0.752375384	0.860622898	NA
G27191	0	1	NA
TMEM132E	2.229065973	0.618922907	NA
CA10	0	1	NA
G30375	0	1	NA
ITGAD	-2.638270749	0.540526219	NA
CYP11B1	3.704134202	0.403547832	NA
ENSMMSG00000011695	4.728541225	0.284790415	NA
PPP2R2C	3.400426231	0.443837602	NA
CDX1	2.196292669	0.624217177	NA
FSTL4	0	1	NA
G35750	3.165225361	0.476570739	NA
MGC133636	-1.136087558	0.789278807	NA
DNAJB13	1.944556828	0.665470049	NA
ALKAL2	-3.26905092	0.449040661	NA
PADI3	0	1	NA
DGKB	-2.986776183	0.500795138	NA
PIK3R6	-5.697688271	0.189720006	NA
LRTM2	-1.345702655	0.750828463	NA
MS4A1	0	1	NA
TRERF1	0	1	NA
CD3G	2.229075736	0.618921333	NA
RETREG1	0	1	NA
ASMT	-3.295688598	0.455455947	NA
XKR6	-1.230145883	0.779840911	NA
ADSL	0	1	NA
SLC22A8	4.844890358	0.272993552	NA
PLPPR4	4.414141074	0.318391102	NA
SLC2A9	0	1	NA
441486/LOC112445612/LO(	0.723890092	0.873434026	NA
GALNT5	0.952680816	0.82616415	NA

SLC7A11	0	1	NA
NOBOX	0	1	NA
G13859	0	1	NA
G15774	3.949398333	0.372684757	NA
OPRM1	0	1	NA
DHRS9	3.724614844	0.400912874	NA
TTLL8	0	1	NA
G18721	0	1	NA
HOXC12	4.844890358	0.272993552	NA
LOC618559	0	1	NA
ENSMMSG00000018840	0	1	NA
CHRM2	2.875370122	0.51867128	NA
G23654	0	1	NA
RAX2	0	1	NA
G24306	1.944558223	0.665469817	NA
NINL	3.121062087	0.482861921	NA
SOX21	2.969099003	0.504850628	NA
CFAP43	0	1	NA
CSTA	0	1	NA
RUNX2	2.761021589	0.535791835	NA
ENSMMSG00000002223	-1.807723714	0.691392049	NA
AQP10	-4.512454705	0.301344429	NA
ADGRD2	0	1	NA
ENSBTAG00000039994	3.121062838	0.482861814	NA
LOC538679	0.98705293	0.827815911	NA
LOC516378	2.932758851	0.510186045	NA
G34315	3.278139337	0.460692782	NA
LOC529488	-2.352723567	0.583714971	NA
G36169	0	1	NA
LSAMP	4.414556308	0.31834506	NA
G37789	2.761016812	0.535792556	NA
LOC100335490	0	1	NA
NGP	0	1	NA
HOXB9	-4.857250862	0.265029115	NA
DNER	4.95255801	0.26238162	NA
GNGT1	-3.445526806	0.4225639	NA
HDHD2	0	1	NA
OLIG3	-5.80474651	0.181413077	NA
GSG1L	-4.995973507	0.251325447	NA
G38354	-5.80474651	0.181413077	NA
LOC783125	-5.80474651	0.181413077	NA
DNALI1	-4.995975858	0.251325224	NA
ENSMMSG00000019835	-3.575713888	0.416452989	NA
SH2D5	0	1	NA
SLC22A9	3.45572146	0.43633412	NA
CACNA2D4	4.95255801	0.26238162	NA
ENSMMSG00000022436	0	1	NA
KCNIP1	4.108813855	0.353440131	NA
XKR7	4.463852832	0.312910298	NA
ENSMMSG00000012892	0	1	NA
ENSMMSG00000003805	0	1	NA
EPHA8	0	1	NA
PLD5	4.601982506	0.298012825	NA
G13432	3.121070624	0.482860701	NA
CPO	0	1	NA
CYP3A24	-5.510213526	0.204947622	NA
ENSMMSG00000011902	0	1	NA
DGKI	0	1	NA

TCF24	3.121073184	0.482860335	NA
SLC44A4	3.121061838	0.482861957	NA
ASCL3	0	1	NA
VAX2	0	1	NA
RPL23A	4.689605808	0.288814843	NA
ZDHHC19	0	1	NA
RTBDN	0	1	NA
G34568	3.449687561	0.43714931	NA
G36442	0	1	NA
GLIPR1L2	0	1	NA
G37839	4.577783029	0.300587573	NA
TPM3	0	1	NA
SLC6A7	0	1	NA
F2RL3	0	1	NA
SMC1B	-1.139014676	0.787563668	NA
TCTE1	-0.542834774	0.898735249	NA
ADAMTS20	2.738716956	0.516894745	NA
ENSMMSG00000017346	-1.079988649	0.802797522	NA
SLC10A4	-1.274531957	0.763685836	NA
ZP3	-5.613188757	0.196475167	NA
DRAXIN	5.052747684	0.252768251	NA
SLC22A6	5.052747684	0.252768251	NA
LRCOL1	0	1	NA
G3881	3.942837752	0.373490574	NA
LOC100299025	0	1	NA
ENSMMSG00000012831	0.987043231	0.827817584	NA
SCN9A	3.042701863	0.494134987	NA
LOC525286	3.165239087	0.476568791	NA
G8979	2.22906378	0.618923261	NA
ZIC2	0	1	NA
G13033	1.65901054	0.713217677	NA
ANKS1B	0	1	NA
CCDC63	5.052747684	0.252768251	NA
GRIN2B	0	1	NA
NR1I2	0	1	NA
ENSBTAG00000031234	0	1	NA
G19925	0	1	NA
FAM187B	2.596339099	0.560933093	NA
ENSMMSG00000011147	1.338710285	0.730879149	NA
G22311	4.309725999	0.330108882	NA
ENSMMSG00000006398	-0.246711872	0.954004118	NA
SLC26A4	2.694989862	0.545805074	NA
LY6G6F	0	1	NA
IL11	0	1	NA
GSG1	4.029328204	0.362954873	NA
FCAR	2.596313076	0.56093711	NA
UPK3BL1	0	1	NA
ENSMMSG00000002011	3.121061903	0.482861948	NA
G31938	0	1	NA
NETO1	4.768132109	0.280737714	NA
ENSMMSG00000012159	2.410235753	0.590000384	NA
DUSP9	0	1	NA
KIF28	3.480404885	0.433008558	NA
AHRR	0	1	NA
KRT72	-3.295694863	0.455455052	NA
IRF8	-4.579861702	0.293989918	NA
SLC34A2	1.558416284	0.716905463	NA
FSHR	-2.348787025	0.581276966	NA

ENSMMSG00000016319	-0.420289769	0.921445392	NA
BRD2	0	1	NA
FGF14	3.556750683	0.422816745	NA
CEP78	0	1	NA
DNMT3B	-4.290787321	0.326409352	NA
ENSBTAG00000047316	-5.582026889	0.199011049	NA
ENPP1	0	1	NA
COL24A1	0.109713472	0.979537531	NA
IDO1	-1.263832942	0.766250387	NA
KCNK4	2.596321345	0.560935834	NA
FAM181A	4.143014565	0.349395709	NA
CGAS	0	1	NA
TNIP3	3.998049152	0.366743166	NA
CFAP58	2.694990745	0.545804939	NA
STK32B	0	1	NA
ENSMMSG00000008495	2.410236961	0.590000193	NA
ENSMMSG00000010227	0	1	NA
ATMIN	3.942835889	0.373490803	NA
HTR2A	-0.890813648	0.835939879	NA
CA1	1.427499955	0.736792405	NA
OTP	2.196292961	0.62421713	NA
SERPINB6	-0.704740643	0.869618883	NA
DRGX	5.065044229	0.251605666	NA
LAMC3	2.422102657	0.588126949	NA
FOXD2	4.137927739	0.349995366	NA
MGAM2	0	1	NA
SFTPB	0	1	NA
HAND2	3.382847358	0.446238502	NA
ENSMMSG00000011503	0	1	NA
NPBWR2	0	1	NA
POU3F3	0	1	NA
ENSMMSG00000020063	0	1	NA
RPL23A	2.596310682	0.56093748	NA
G33437	0	1	NA
G33568	0	1	NA
ENSMMSG00000014899	3.949412123	0.372683064	NA
PHACTR3	-4.315973677	0.323493279	NA
INA	-0.191483233	0.963961077	NA
ENSMMSG00000010863	1.944564485	0.665468779	NA
DIRAS3	-4.995953819	0.25132732	NA
MORN5	4.312508931	0.329792954	NA
ENSMMSG00000003929	-5.51019747	0.204948942	NA
ATP10D	3.657755701	0.409553318	NA
DNAH3	4.309739633	0.330107333	NA
G2100	3.449685449	0.437149596	NA
NRXN3	3.717233543	0.401861317	NA
HS3ST2	3.042725365	0.494131585	NA
G5525	2.196300631	0.624215889	NA
SYT1	0	1	NA
BECN1	-0.293796952	0.939934053	NA
CLCNKB	3.717225948	0.401862293	NA
OTOP2	0	1	NA
CLSTN1	0	1	NA
KCNJ4	0	1	NA
ENSMMSG00000010189	-0.111725121	0.979640079	NA
G14461	3.657755701	0.409553318	NA
SEC14L3	2.69498617	0.545805636	NA
GAL3ST1/LOC101906447	0	1	NA

NPSR1	0	1	NA
TMEM178B	3.029679498	0.496021978	NA
ZSWIM5	2.410242685	0.589999289	NA
CPB1	3.042701177	0.494135086	NA
G22148	4.078358089	0.357066793	NA
LGR6	2.19629609	0.624216624	NA
CCDC81	4.76794184	0.280757096	NA
REG4	0	1	NA
EML6	0	1	NA
ENSMMSG00000019833	0	1	NA
TMEM88B	-2.986783602	0.500794074	NA
MAMDC2	-2.638817661	0.535075446	NA
G24313	0	1	NA
ENSMMSG00000003573	3.738854964	0.399086968	NA
KCNU1	1.294824716	0.760976944	NA
GATA1	0	1	NA
PLAAT5	0	1	NA
G1840	3.382847927	0.446238424	NA
GRID1	0	1	NA
POLR3D	0.256970062	0.951576985	NA
TMEM35B	0	1	NA
ENSMMSG00000019076	-2.456861915	0.56258833	NA
COL25A1	2.694986923	0.545805521	NA
ENPP7	1.944553854	0.665470542	NA
RPL23A	2.875371754	0.518671037	NA
LOC615223	0	1	NA
AMER2	4.464481171	0.312841427	NA
CYP7A1	3.1210602	0.482862191	NA
SLC6A12	-5.162886428	0.235516233	NA
SLC5A9	0	1	NA
CHRM5	0	1	NA
HRK	0	1	NA
KRT7.L	0	1	NA
PROKR2	3.717222532	0.401862733	NA
FFAR2	1.659008372	0.71321804	NA
DNAH5	2.196260598	0.624222367	NA
EIF4A2	-0.136053384	0.967339476	NA
ENSMMSG00000019535	1.349382855	0.765668845	NA
EDN3	0	1	NA
NTM	0.98704178	0.827817834	NA
CHRNG	3.94283601	0.373490788	NA
C3H1ORF94	0	1	NA
KIAA1549L	4.124940851	0.351529308	NA
G34402	-0.127384852	0.976417461	NA
TKTL2	3.121065446	0.482861441	NA
AVPR2	2.908800665	0.513719577	NA
DAPP1	0	1	NA
C1QL2	0	1	NA
CYP26A1	-5.581998555	0.199013332	NA
MCHR1	-4.579856048	0.293990515	NA
LHX2	-3.575707777	0.416453786	NA
FAAP20	1.659021214	0.713215887	NA
PLA2G5	3.462346185	0.435440125	NA
CEP135	3.875860483	0.381779499	NA
ARNT2	4.076809311	0.357251853	NA
FGF16	1.622547958	0.70572936	NA
CHRDL1	-5.789893774	0.182548859	NA
GRIA2	0	1	NA

ERN2	2.596316489	0.560936583	NA
SDCBP	0.723898679	0.873432532	NA
XKR4	2.695007804	0.54580234	NA
RS1	2.196298357	0.624216257	NA
THEGL	0	1	NA
S100Z	0	1	NA
G17509	3.165250594	0.476567157	NA
DIRAS2	1.65900897	0.71321794	NA
ENSMMSG00000008861	0	1	NA
TNFRSF9	0	1	NA
HMX1	0	1	NA
PRSS54	0	1	NA
TRIM58	4.916668059	0.265886565	NA
C15H11ORF1	0	1	NA
MUC4	0	1	NA
LOC529425	1.659010003	0.713217767	NA
G34103	3.97401742	0.369670592	NA
BBS4	-0.420206958	0.912352747	NA
SOWAHD	-0.799483716	0.849690525	NA
RBM17	1.659014405	0.713217029	NA
MAK	-0.049033571	0.987734522	NA
PPP4R4	-0.23930799	0.93625656	NA
OBSL1	0	1	NA
SLC9A3	-2.986759298	0.500797559	NA
G36147	3.449712537	0.437145934	NA
G27348	0	1	NA
ENSMMSG00000009042	3.382851959	0.446237872	NA
SLC6A15	0	1	NA
ENSMMSG00000003533	-3.295671419	0.455458258	NA
G5873	0	1	NA
G7024	3.361018778	0.449230167	NA
TNR	0	1	NA
DPP6	0	1	NA
G13446	4.081417595	0.356701401	NA
TEX13B	1.960278122	0.662864895	NA
PDCD1LG2	4.863071046	0.251046923	NA
G18615	-3.708327605	0.398704403	NA
KRT84	0	1	NA
KRT86	0	1	NA
NWD1	4.72856475	0.284787995	NA
LOC614741	0	1	NA
HPD	2.088455062	0.641765103	NA
HTR2C	0	1	NA
CDH22	4.881293447	0.269372905	NA
ADAM22	3.888521178	0.380203957	NA
ENSBTAG00000049371	1.409235785	0.744700717	NA
BLOC1S4	0	1	NA
LOC617402	-2.284603371	0.590757958	NA
TPO	-4.489398712	0.303888507	NA
ZNF335	3.239843355	0.447995896	NA
PDE10A	4.669674767	0.290889864	NA
ENSMMSG00000008498	3.95790333	0.371641736	NA
TNFSF9	-0.393030609	0.926204628	NA
LOC785540	-0.779298391	0.854086035	NA
C1QTNF4	-3.575681098	0.416457263	NA
LOC511175	4.137914604	0.349996915	NA
GLRB	3.938362659	0.374040862	NA
SP9	2.08844302	0.641767073	NA

RPS10	4.159529588	0.298003223	NA
HMGCL	-4.512444694	0.301345501	NA
PPFIA1	-0.490421055	0.889603893	NA
ENSMMSG00000000795	4.687372803	0.289046818	NA
CLDN11	-3.295691021	0.455455578	NA
PRKCA	0.723880054	0.873435774	NA
ANKS1B	0	1	NA
IPCEF1	3.717219332	0.401863144	NA
MGAM	3.717226867	0.401862175	NA
CALHM1	0	1	NA
TRIM31	0	1	NA
GRIK1	2.694992254	0.545804709	NA
NTM	0	1	NA
OPCML	0	1	NA
SLC5A7	0	1	NA
ADAM28	0	1	NA
H3-3D	2.08844348	0.641766998	NA
NCR1	-1.985375302	0.641633167	NA
G22691	2.596318522	0.560936269	NA
ADARB2	0	1	NA
FGF16	-3.235361077	0.447606087	NA
G35317	-1.704629275	0.68725444	NA
LEMD1	0	1	NA
G2072	0	1	NA
CHRNB4	-2.715241675	0.542525584	NA
PRR35	1.659012718	0.713217312	NA
G10580	4.310440226	0.330027781	NA
NDST4	0.927211831	0.8285587	NA
OR10AC1	2.694992696	0.545804642	NA
G14401	2.410243505	0.589999159	NA
COL6A5	0	1	NA
G16231	3.717227902	0.401862042	NA
ENSMMSG00000013231	0	1	NA
NOL4	0	1	NA
TG	3.278180986	0.46068698	NA
495184/LOC101732638/LO(	0	1	NA
RIMS2	0	1	NA
ENSBTAG00000054034	0	1	NA
G14008	0.541562153	0.900210906	NA
CRTAM	-0.420267592	0.921449512	NA
HDC	-1.223865803	0.777612421	NA
BOD1	-0.586965616	0.881690351	NA
PRSS46P	0	1	NA
TESMIN	-4.290779289	0.326410243	NA
LOXHD1	1.944557741	0.665469897	NA
ENSMMSG00000023592	0	1	NA
GRM7	3.949427489	0.372681178	NA
BARX1	-0.70471595	0.86962338	NA
CDH6	3.187756937	0.473378495	NA
TM4SF4	0	1	NA
G23250	3.581508215	0.419542426	NA
G28382	2.196282328	0.624218851	NA
LOC101905611	1.944565104	0.665468676	NA
SSC4D	0	1	NA
ZBTB8B	3.878452598	0.381456598	NA
G32670	5.051039368	0.252930063	NA
RHBDL2	0	1	NA
HDX	4.087380435	0.355989952	NA

CCR7	-0.752794403	0.851757424	NA
G3452	4.137929609	0.349995145	NA
TMEM163	2.196266262	0.624221451	NA
KLHDC7B	-3.020779689	0.477999609	NA
ENSMMSG00000015285	0	1	NA
G2073	0	1	NA
G3412	3.717237456	0.401860814	NA
SCN2A	0	1	NA
C28H10ORF105	3.278167431	0.460688868	NA
ENSMMSG00000000832	0	1	NA
ENSMMSG00000004437	3.802401562	0.341237098	NA
MAP3K15	0	1	NA
MGAT3	0	1	NA
MKRN2OS	0	1	NA
CALN1	-1.687200015	0.694107166	NA
ZDHHC6	-1.10077471	0.791354702	NA
LRRTM1	0	1	NA
ENSMMSG00000005130	4.198915272	0.342849416	NA
G20541	0	1	NA
LOC100848132	1.349376369	0.765669954	NA
ZSCAN30	0	1	NA
GSC2	0	1	NA
G26975	3.756805823	0.396792483	NA
IL18RAP	0	1	NA
NCR3LG1	3.159315912	0.372868056	NA
G32643	0	1	NA
G34599	0	1	NA
DEPDC1B	2.410213296	0.590003932	NA
LAMB4	0	1	NA
BPTF	4.206185373	0.34200391	NA
G3091	3.728262481	0.400444682	NA
G31964	-4.290777511	0.326410458	NA
BRINP3	1.6137257	0.715477814	NA
LY86	-0.827617058	0.854883233	NA
G7875	1.661045655	0.712876541	NA
TTLL9	-3.575711438	0.416453273	NA
GRK7	-0.861731265	0.81568144	NA
TIAM1	3.165256398	0.476566333	NA
ENSMMSG00000023821	-5.466475429	0.195138368	NA
G28212	4.806327445	0.276865535	NA
ENSMMSG00000001463	-2.447575795	0.55780481	NA
MYOM1	-0.590416848	0.872880925	NA
AQP6/AQP5	0	1	NA
SMIM10L2B	0	1	NA
G5737	3.717229297	0.401861863	NA
LOC100299732	-0.562891709	0.868425686	NA
ENSMMSG00000023977	3.888544281	0.380201085	NA
G10550	2.410245794	0.589998798	NA
KDM4D	2.62792515	0.471272977	NA
THSD7B	3.738876938	0.399084154	NA
HOXC13	0	1	NA
ENSMMSG00000020907	0	1	NA
LMNTD2	0	1	NA
LCN8	4.078367602	0.357065657	NA
ENSMMSG00000014847	0	1	NA
NTRK3	0	1	NA
NLRC4	0	1	NA
CA5A	-4.290782253	0.326409909	NA

G30341	0	1	NA
ENSMMSG00000001654	0	1	NA
G31919	0	1	NA
CMTM5	-0.704726062	0.869621538	NA
HRH3	0	1	NA
KIF2C	0	1	NA
NKX6-2	1.349371432	0.765670798	NA
EPHB2	0.972029076	0.810076304	NA
ADAMTS16	0.181540994	0.966561033	NA
CLDN18	0	1	NA
NCAN	0	1	NA
LRRC3C	3.94944225	0.372679366	NA
G18032	3.468501761	0.434610398	NA
G21080	0	1	NA
CELA2A	1.659012783	0.713217301	NA
ALKBH1	2.969132088	0.504845783	NA
AARSD1	-1.227385231	0.778606975	NA
ACSM2B	4.262106187	0.335545529	NA
DOK6	1.659012293	0.713217383	NA
G36975	1.944559371	0.665469627	NA
CEP250	0.97533561	0.806142931	NA
TMEM106A	3.053119548	0.341146925	NA
NFASC	0	1	NA
ENSBTAG00000053458	-3.575675122	0.416458042	NA
G26781	-0.478356807	0.909085683	NA
XCL2	0	1	NA
WNT11	1.009784232	0.821067151	NA
FCMR	4.787299295	0.278789957	NA
BMP3	4.687299546	0.289054431	NA
UNC5C	0	1	NA
ENSBTAG00000022502	0	1	NA
ENSMMSG00000007916	0	1	NA
GRM8	1.944564279	0.665468813	NA
CAMTA1	0	1	NA
PNMA2	0	1	NA
SCP2D1	0.307089719	0.917680718	NA
TNFSF4	-2.986761386	0.50079726	NA
ENSMMSG00000001959	0	1	NA
ENSBTAG00000012559	0.140596571	0.97350577	NA
TMEM132D	3.888544457	0.380201064	NA
RFX4	4.36782577	0.323554325	NA
G2630	0	1	NA
SV2C	2.695017841	0.545800811	NA
G4556	-0.70130552	0.848825686	NA
SLC35G3	2.64607045	0.553281917	NA
RAC1	0.987047871	0.827816783	NA
TEX33	3.717230801	0.401861669	NA
KCNV2	3.942842248	0.373490021	NA
PCP2	1.944575262	0.665466992	NA
CACNA1B	0	1	NA
ENSMMSG00000021009	2.596340531	0.560932872	NA
G31687	0	1	NA
GJB6	3.581513062	0.419541787	NA
ADAM3A	4.262118019	0.335544171	NA
C13H20ORF144	0	1	NA
CATIP	0.556612553	0.883044334	NA
ENSMMSG00000022093	-2.194983917	0.488046706	NA
CD72	3.333656221	0.399418338	NA

DENND1C	-1.312365245	0.758598154	NA
SYT13	0	1	NA
G1438	2.76102839	0.535790809	NA
ENSMMSG00000011634	0	1	NA
ALX1	2.76102839	0.535790809	NA
KCNH1	2.76103442	0.535789898	NA
DRD3	0	1	NA
CYP2W1	0	1	NA
TMSB10	1.94459273	0.665464095	NA
SPEF2	0	1	NA
LOC518134	4.49254528	0.309775669	NA
EDN2	0	1	NA
G37247	3.251609717	0.464396433	NA
CREB3L3	-1.411545252	0.740173884	NA
G19981	2.969110354	0.504848966	NA
NRL	-0.630515864	0.88200031	NA
ENSBTAG00000053264	-0.817036335	0.800436877	NA
IDI1	1.868047084	0.650765966	NA
ABCB1	0	1	NA
SYN1	4.145553658	0.349096638	NA
UCP1	0	1	NA
TMEM71	2.908780681	0.51372253	NA
NKX6-1	2.969133474	0.504845581	NA
G20170	0	1	NA
ENSMMSG00000000172	2.968981929	0.499311597	NA
ENSMMSG00000016425	0.563528262	0.887639855	NA
ENSMMSG00000013849	4.879799305	0.269520855	NA
LONRF2	0	1	NA
ENSMMSG00000002994	0	1	NA
ENSBTAG00000054356	0	1	NA
SLC35D3	0	1	NA
ENSMMSG00000013903	0	1	NA
G28580	-2.98677794	0.500794886	NA
CHADL	0	1	NA
TMEM158	0	1	NA
PRF1	4.016132989	0.290415765	NA
GRIN2B	0	1	NA
TMEM273	4.539924622	0.210040115	NA
LRRC18	1.944561608	0.665469256	NA
SLC43A3	2.196325546	0.624211857	NA
CLEC5A	0	1	NA
KCNAB2	0	1	NA
G26161	3.951584269	0.372416507	NA
CDH8	3.966000602	0.37065042	NA
ENSMMSG00000018740	3.878454951	0.381456305	NA
KLK4	1.349376614	0.765669912	NA
SLC22A7	2.694996633	0.545804042	NA
ADCYAP1R1	3.480454406	0.433001901	NA
CXCR3	-0.914832812	0.805910587	NA
TMEM255B	0	1	NA
RAB39B	3.121070789	0.482860677	NA
G1378	0.723924831	0.873427979	NA
VSX2	0	1	NA
ZNF536	-0.981083774	0.757543607	NA
NRSN2	0	1	NA
XDH	4.031927481	0.362641187	NA
ENSMMSG00000003016	1.944567283	0.665468315	NA
FHAD1	0	1	NA

KCNE2	0.956619924	0.757806126	NA
G742	0	1	NA
ANO3	0	1	NA
SORCS3	2.410217664	0.590003242	NA
RPL12	4.255594071	0.32357884	NA
GPR182	0	1	NA
TAFA3	4.511833327	0.294407271	NA
ACTR3	3.912334258	0.175541662	NA
G15347	0	1	NA
MLNR	3.888566032	0.380198382	NA
ENSMMSG00000018930	4.41458892	0.318341444	NA
KIF6	2.596323882	0.560935442	NA
TNFSF15	4.983159178	0.25941872	NA
CPLX4	0	1	NA
AIMP1	0	1	NA
G20470	3.406413229	0.380754456	NA
CACNA1A	3.480436963	0.433004246	NA
GRID1	3.942846241	0.37348953	NA
MC2R	0	1	NA
HECW1	4.367846087	0.323552048	NA
PCYT1B	0	1	NA
G11083	3.311348023	0.447404673	NA
G16631	1.944576176	0.66546684	NA
NDST3	2.196289537	0.624217684	NA
LOC112446467	3.888571142	0.380197747	NA
CD96	1.349374197	0.765670325	NA
DDX4	0	1	NA
GP9	-0.950608574	0.815453167	NA
ENSMMSG00000008615	3.165281133	0.476562823	NA
TTC29	0	1	NA
LTA	0	1	NA
DLGAP1	4.805429258	0.276956167	NA
NLRC4	3.809754458	0.390071591	NA
MBNL3	0	1	NA
LAMA1	0	1	NA
PAK5	1.659036124	0.713213388	NA
LRCH2	0	1	NA
IPCEF1	0	1	NA
G17127	0	1	NA
SP7	4.349144508	0.139640902	NA
ARNTL2	0.453062598	0.917107627	NA
G33805	2.373860484	0.595759418	NA
ARNT2	0	1	NA
G4561	0	1	NA
ENSMMSG00000009839	0	1	NA
PTPN22	3.278175674	0.46068772	NA
EPHA6	0	1	NA
FNDC8	0	1	NA
SLC4A5	2.761039698	0.535789102	NA
ENSBTAG00000049991	2.909363873	0.510905066	NA
KIF15	0	1	NA
ENSBTAG00000047410	0	1	NA
SMIM33	0	1	NA
C26H10ORF82	2.908787943	0.513721457	NA
NRXN1	3.382867212	0.446235786	NA
FOXF2	0	1	NA
GARRE1	3.776087838	0.241667472	NA
HOXD10	-0.828547433	0.845406403	NA

SPRED3	1.944575819	0.6654669	NA
LOC616413	3.278183753	0.460686595	NA
ADRB3	2.196277783	0.624219586	NA
GRM1	0	1	NA
CYP2S1	4.315968106	0.329400534	NA
CNTNAP4	0	1	NA
GABRD	0	1	NA
DGKI	0	1	NA
MASP2	2.065701077	0.630294393	NA
AKAP17B	0	1	NA
ZKSCAN4	0	1	NA
AKR1E2	3.149489835	0.314832446	NA
RAPGEF5	4.913059016	0.26624081	NA
SSTR2	4.804300628	0.277070082	NA
LOC618939	0	1	NA
ADD2	3.888574953	0.380197273	NA
PCDHA13	3.551046397	0.414732998	NA
CDCA7	0	1	NA
KCNT2	4.148022994	0.34880594	NA
G9595	3.799665174	0.375435439	NA
LOC527981	0	1	NA
NRG1	0	1	NA
ASCL4	4.315969741	0.329400349	NA
IKZF1	3.042721331	0.494132169	NA
G6795	0	1	NA
HTR2A	0	1	NA
ANKRD6	5.014198912	0.256437335	NA
SIPA1L1	5.014198912	0.256437335	NA
TSSK2	2.433156999	0.522457037	NA
H2AC14	4.262139871	0.335541663	NA
G27294	4.804310556	0.277069079	NA
LINGO2	2.229096839	0.61891793	NA
CMPK1	3.091596705	0.343940481	NA
KCNQ4	-4.31596984	0.323493727	NA
LOC101910047	2.90975493	0.509112195	NA
RIMS4	1.659034368	0.713213682	NA
TBX20	0	1	NA
TG	3.571854744	0.420817357	NA
CADPS	0	1	NA
NEGR1	0	1	NA
CRHBP	1.660590876	0.709135411	NA
LOC516599	3.657795811	0.409548101	NA
SLC4A10	0	1	NA
GRIA1	0	1	NA
UEVLD	0	1	NA
TCF7	0	1	NA
ATP2B3	0	NA	NA
UMOD	0	NA	NA
ENSMMSG00000004749	0	NA	NA
WDR72	0	NA	NA
SORBS2	20.65633369	NA	NA
PAX3	0	NA	NA
NRCAM	21.11195667	NA	NA
HOXC4	1.615017184	NA	NA
SELE	3.96006861	NA	NA
PTPRD	6.381336639	NA	NA
AKR1E2	3.16527985	NA	NA
ITIH1	0	NA	NA

RASSF6	0	NA	NA
RPL27A	0	NA	NA
BHMT2	3.705954161	NA	NA
ANKRD26	0	NA	NA
G22153	0	NA	NA
RYR3	21.84581482	NA	NA
TCP11	-0.325684785	NA	NA
MAGI2	0	NA	NA
CLASP2	21.35890372	NA	NA
DAB1	8.289366353	NA	NA
BHLHA15	-3.105559651	NA	NA
LTBP1	22.00529669	NA	NA
SMIM10L2B	1.523199567	NA	NA
JAKMIP3	2.652829296	NA	NA
CD1B	6.635293168	NA	NA
C10H15ORF48	4.883046211	NA	NA
EXO1	3.815681349	NA	NA
MBNL3	5.484457561	NA	NA
G32329	-1.75154242	NA	NA
R3HDML	0	NA	NA
PKD1L3	0	NA	NA
ENSMMSG00000022051	21.08847601	NA	NA
C16H1ORF105	-1.334663935	NA	NA
ADAM22	0	NA	NA
NFASC	0.484467435	NA	NA
PTPRD	20.97123922	NA	NA
GRIP2	22.33554089	NA	NA
MAEL	1.221550334	NA	NA
TNMD	-1.932598806	NA	NA
CHL1	-15.7576193	NA	NA
G26037	22.22393139	NA	NA
ANKS4B	2.732088079	NA	NA
TSGA10IP	6.824424663	NA	NA
H2AC12	6.224244258	NA	NA
SYNDIG1	0	NA	NA
NRSN1	-1.743960855	NA	NA
POU6F2	6.00297678	NA	NA
CNTNAP2	5.549393826	NA	NA
ZNF618	5.308017608	NA	NA
GDPD5	-1.181948363	NA	NA
ROBO2	0	NA	NA
PRKCB	-17.94226942	NA	NA
ENSMMSG00000002989	0	NA	NA
ENSMMSG00000003766	-0.2415055	NA	NA
LOC529792	21.17108974	NA	NA
PRCD	-2.500862959	NA	NA
DAB1	22.67298525	NA	NA
CEP152	0	NA	NA
AAK1	3.293792974	NA	NA
ENSMMSG00000017825	1.299867011	NA	NA
TRPV2	-0.763992055	NA	NA
RC3H2	20.75927208	NA	NA
LY6G6E	4.695542188	NA	NA
CEMIP	0	NA	NA
OSCP1	1.032701429	NA	NA
ENSMMSG00000009649	0.92599023	NA	NA
RYR3	8.770598444	NA	NA
CHRNG	0.776361436	NA	NA

DCLK1	21.56964904	NA	NA
APBB1IP	20.91365127	NA	NA
INHCA	0	NA	NA
GLIS3	6.384019807	NA	NA
ADAMTS20	0.634055024	NA	NA
ADGRL1	0	NA	NA
KCNK12	-0.569420451	NA	NA
ADGRB3	9.32366667	NA	NA
ATP8B4	17.48481296	NA	NA
CRISP3	0	NA	NA
IL17F	-1.937201626	NA	NA
POU4F1	0	NA	NA
LOC100847119	4.015477326	NA	NA
BMX	0	NA	NA
TEX14	6.754009334	NA	NA
ENSBTAG00000047700	2.346250165	NA	NA
C1GALT1	0.757721779	NA	NA
G2231	-1.813182242	NA	NA
ANKHD1	0	NA	NA
SLC22A3	0.876871375	NA	NA
ANO2	0.918896578	NA	NA
ADAM22	-0.256951483	NA	NA
C1QL3	4.649043227	NA	NA
FNTA	-1.874792831	NA	NA
LOC100848610	18.20175954	NA	NA
RTP4	-0.849852259	NA	NA
TTC21B	2.669052763	NA	NA
G32962	0.407525989	NA	NA
MAPT	2.314189137	NA	NA
CHRNB3	0.586679347	NA	NA
INHBB	0.252909796	NA	NA
SPIRE1	8.521427244	NA	NA
C2H2ORF80	1.372402329	NA	NA
RCOR3	-2.869210723	NA	NA
ENSBTAG00000050723	20.65377984	NA	NA
PSMD10	0	NA	NA
LEFTY2	0	NA	NA
CDH17	0.459108257	NA	NA
CLEC6A	0.518523043	NA	NA
NOTCH4	19.55464039	NA	NA
CLEC18C	-1.813486521	NA	NA
ERO1B	22.14146198	NA	NA
MARK4	22.22554796	NA	NA
RNPS1	0	NA	NA
SEMA6A	22.22343289	NA	NA
TBC1D8B	0.282704541	NA	NA
OTOG	-0.901595656	NA	NA
ASB18	0.733650599	NA	NA
RAPGEFL1	7.048541298	NA	NA
DYNC2LI1	0.879116713	NA	NA
ITGA9	2.139524333	NA	NA
GET1	-2.854260613	NA	NA
PSMB11	0	NA	NA
C1QL1	0	NA	NA
DMKN	6.466464515	NA	NA
RASIP1	0	NA	NA
G33358	-0.765249787	NA	NA
ADGRE3	0	NA	NA

SARG	0	NA	NA
CSRNP3	10.25462327	NA	NA
LOC101904413	1.228359684	NA	NA
GABBR1	0.853820861	NA	NA
ARID4B	0.600220883	NA	NA
G34389	10.45160174	NA	NA
NUMBL	-0.569432642	NA	NA
ZNF185	0	NA	NA
PLA2G6	-1.096272358	NA	NA
HABP2	0.1888185	NA	NA
CCDC87	4.611600418	NA	NA
FGF9	3.902674452	NA	NA
SLAIN1	0.88395625	NA	NA
DISP1	-0.013228844	NA	NA
REEP1	0.269588714	NA	NA
PVRIG	1.710971487	NA	NA
MOGAT1	0.434302114	NA	NA
LOC515418	-0.269006951	NA	NA
HEPH	1.636242523	NA	NA
AANAT	0	NA	NA
SBF1	0	NA	NA
TUB	0.002691424	NA	NA
ENPP3	4.683584594	NA	NA
G6915	-1.958741966	NA	NA
RGS7BP	0.714877384	NA	NA
TBC1D4	-0.633790908	NA	NA
ASB18	0.490768138	NA	NA
ENSMMSG00000020212	-1.958722801	NA	NA
SERTAD2	0.250574261	NA	NA
RPL23A	2.911452779	NA	NA
FADS2	-14.6255773	NA	NA
ENSBTAG00000053533	-1.958730636	NA	NA
RAB40B	0.833801559	NA	NA
NAALAD2	0.735884278	NA	NA
LYPD6B	0	NA	NA
ANKRD33B	-0.701838255	NA	NA
OLFML2A	-2.246757171	NA	NA
RPRML	-1.958730099	NA	NA
C2CD5	3.469259916	NA	NA
RALGPS1	22.56618827	NA	NA
FRYL	2.173460573	NA	NA
CDC42SE2	-0.060848328	NA	NA
HBBC	6.164387961	NA	NA
RAPGEF2	-2.944613713	NA	NA
MAPK8IP3	19.01699311	NA	NA
PTPRD	-0.323594776	NA	NA
DOK7	-2.749920046	NA	NA
GRIA3	0.800858324	NA	NA
DLG2	10.9708226	NA	NA
PORCN	8.40179519	NA	NA
NCAM1	-1.557887582	NA	NA
SLC37A2	1.077608735	NA	NA
KCNH2	-1.558705327	NA	NA
SNAP91	5.359384662	NA	NA
FGF1	0.177810735	NA	NA
CREB5	-0.125143651	NA	NA
SDK2	0	NA	NA
KIF26A	24.49491402	NA	NA

ATP6V0A4	2.947377715	NA	NA
PIGH	0.326121006	NA	NA
AMPD2	-3.548982843	NA	NA
NCAM1	-0.239963592	NA	NA
SETD5	-23.8050673	NA	NA
LTBP4	-0.231917199	NA	NA
KIAA2026	0.201378202	NA	NA
SLC28A1	2.824799646	NA	NA
MAFB	-0.219360108	NA	NA
BTG3	0.113440649	NA	NA
ACER2	0.459822046	NA	NA
EPB41L1	0.917604109	NA	NA
MTNR1A	21.3716076	NA	NA
CYP3A24	0.058205023	NA	NA
CYFIP2	-0.256500096	NA	NA
SLC35E4	0.665975208	NA	NA
G14510	0	NA	NA
GRIP1	22.67898646	NA	NA
PKP2	10.40663252	NA	NA
PROM1	-0.164460138	NA	NA
LOC521081	21.98807578	NA	NA
RAPGEF4	0.76754808	NA	NA
L3MBTL3	-0.200083977	NA	NA
PCDHGA10	0.712712324	NA	NA
SASH1	-1.919921334	NA	NA
PRKAG3	-2.224649055	NA	NA
CTIF	-0.190508936	NA	NA
AADACL4	4.309844115	NA	NA
SPTAN1	7.558301322	NA	NA
CA6	0	NA	NA
RPL4	4.417756159	NA	NA
AURKB	7.124923842	NA	NA
USP46	-0.270487363	NA	NA
PAXIP1	0.774998974	NA	NA
CAMKK2	0.217091626	NA	NA
MTCL1	0	NA	NA
FSD1L	12.12177435	NA	NA
DGKA	0	NA	NA
AP1AR	-18.63349588	NA	NA
CCNH	3.323864553	NA	NA
USP19	1.338659431	NA	NA
PLIN1	-0.107082742	NA	NA
ENSMMSG00000016565	0	NA	NA
SYN2	0	NA	NA
ENSMMSG00000023698	0	NA	NA
NIPSNAP3A	1.395586132	NA	NA
ST8SIA5	-0.098294192	NA	NA
GPRIN2	0	NA	NA
HECTD1	-5.555515181	NA	NA
ENSBTAG00000017443	0	NA	NA
SDR16C5	20.39643393	NA	NA
ENSBTAG00000048135	3.881460685	NA	NA
DZANK1	-5.113511439	NA	NA
FNBP4	-0.032024791	NA	NA
KIFC3	3.352727149	NA	NA
RICTOR	0.487461324	NA	NA
NAA50	0	NA	NA
ADAM33	-0.94778796	NA	NA

CSRP2	1.638404491	NA	NA
LOC618076	-2.715126931	NA	NA
ADIPOQ	-0.660464205	NA	NA
MYO5A	26.12168775	NA	NA
SHF	2.675128554	NA	NA
SMARCAD1	1.255256885	NA	NA
ENSMMSG00000003583	-4.585720088	NA	NA
RSPO3	0.675232856	NA	NA
ENSMMSG00000019730	-3.725632278	NA	NA
PPFIBP2	-0.825706651	NA	NA
CP	0.920255268	NA	NA
SDR16C6	-1.099622754	NA	NA
WDSUB1	1.745716292	NA	NA
DAPL1	-14.27681537	NA	NA
CCDC120	3.762548912	NA	NA
KLK10	5.472624781	NA	NA
TREM2	0.873592341	NA	NA
DEPDC5	-0.152706643	NA	NA
ENSMMSG00000019868	-5.434186455	NA	NA
MOGAT2	0.784767121	NA	NA
CARNS1	-0.824089657	NA	NA
CPEB3	-0.754223395	NA	NA
TNFRSF12A	0.297139477	NA	NA
C3H1ORF210	2.945961418	NA	NA
ARG2	1.08250857	NA	NA
IP6K1	0.105444283	NA	NA
NIN	26.27092158	NA	NA
SERPINE2	0.563662645	NA	NA
KRT42	0	NA	NA
CCDC69	-0.697279794	NA	NA
ABLIM2	-0.502144808	NA	NA
FAM169A	4.103707757	NA	NA
NCOA2	0.006695286	NA	NA
ETV4	4.104236579	NA	NA
CCND1	0	NA	NA
MRC2	-0.006255364	NA	NA
NDEL1	-24.25469201	NA	NA
CNOT4	-0.23370001	NA	NA
LRBA	-0.266667178	NA	NA
ENSMMSG00000004237	0.276352962	NA	NA
FCHO2	-0.201906225	NA	NA
ZFP91	-0.578925912	NA	NA
TNPO2	1.624346134	NA	NA
TRAPPC13	-0.474626845	NA	NA
HSD17B6	16.99148121	NA	NA
ADGRB1	0.804854247	NA	NA
ENSMMSG00000019737	-5.435690658	NA	NA
LOC104970284	-5.434313582	NA	NA
UVSSA	0.299748938	NA	NA
BACH1	-0.217723318	NA	NA
LTBP1	0.359710335	NA	NA
PPFIBP1	-0.00429351	NA	NA
RC3H1	1.131582535	NA	NA
KRT77	0	NA	NA
AHSG	0	NA	NA
NPNT	-2.511686933	NA	NA
MYCBP2	0	NA	NA
ENSMMSG00000024355	2.763774406	NA	NA

ATP8A1	21.67489707	NA	NA
NEB	-0.155748222	NA	NA
BROX	0.302220662	NA	NA
AQP7	0.296781791	NA	NA
ERN1	1.017928343	NA	NA
LPIN3	0	NA	NA
KLF9	-0.517244802	NA	NA
ATP2B2	-3.860190474	NA	NA
ACADSB	-0.851154312	NA	NA
ZFP91	27.1483605	NA	NA
ELOVL3	-3.976805125	NA	NA
CYP51A1	2.069422127	NA	NA
CLEC16A	-0.952379983	NA	NA
ENAH	0.906365093	NA	NA
MYOG	-0.105582073	NA	NA
KRT79	18.12124615	NA	NA
ETV3	-0.072893015	NA	NA
SIT1	4.289754763	NA	NA
CD47	1.390621921	NA	NA
CNFN	-15.38054572	NA	NA
NT5C1A	-1.037309784	NA	NA
ZNF655	-14.27251192	NA	NA
TTC39A	3.77728313	NA	NA
LOC507527	-14.27033441	NA	NA
CLASP2	9.713029947	NA	NA
G21094	-5.387909715	NA	NA
PUM2	0.277492327	NA	NA
NCOR2	-0.239451696	NA	NA
MINDY3	2.28402607	NA	NA
AGO4	-0.375238713	NA	NA
CASQ2	1.596599736	NA	NA
PARVB	-0.949289026	NA	NA
MAP4K4	-1.09644888	NA	NA
HSPB2	-0.023463409	NA	NA
MINK1	1.4863924	NA	NA
RPL35	1.11362213	NA	NA
PARD3	5.150840997	NA	NA
SHROOM2	1.808409862	NA	NA
PALMD	0.862202336	NA	NA
MIA2	26.6883693	NA	NA
LOC785756	-4.948502735	NA	NA
RCAN1	0.356696449	NA	NA
MICAL3	-0.363775257	NA	NA
CAMSAP2	-0.274062637	NA	NA
GRID2IP	3.737589644	NA	NA
IQSEC1	-0.449104172	NA	NA
WBP4	0.332667695	NA	NA
RALGAPA2	-0.364785094	NA	NA
TSC22D1	-0.595796675	NA	NA
WDTC1	-0.635830304	NA	NA
METTL21C	2.681881711	NA	NA
MGST3	1.313756689	NA	NA
SRCAP	0.408405239	NA	NA
USP28	-0.017297353	NA	NA
RETREG1	0.199099597	NA	NA
NOTCH2	0.778153445	NA	NA
EML4	1.503478972	NA	NA
MYF6	0.040020974	NA	NA

LGALS1	0.406751357	NA	NA
NES	0.106192231	NA	NA
ANK2	-1.915717698	NA	NA
MYH2	-1.044271653	NA	NA
HOMER2	0.379327574	NA	NA
HUWE1	-0.077358363	NA	NA
MYH4	-0.879216888	NA	NA
RRAD	0.641200577	NA	NA
HERC2	-0.182388137	NA	NA
LOC101908350	-1.284365153	NA	NA
KIAA1217	0.127343173	NA	NA
MAP7	3.992078088	NA	NA
TWF2	0.073629592	NA	NA
LMCD1	0.30674395	NA	NA
ANKRD2	0.732479928	NA	NA
VWF	-0.400980042	NA	NA
APOBEC2	-0.012384914	NA	NA
BICDL2	5.45962041	NA	NA
NEURL4	-1.323391679	NA	NA
FABP4	4.654377815	NA	NA
PKP3	7.161563721	NA	NA
FABP3	0.289270541	NA	NA
LOC512548	8.596322728	NA	NA
MYBPH	0.407726852	NA	NA
LOC783399	-0.22445396	NA	NA
LOC104970193	-4.255496127	NA	NA
RPL35A	1.36900314	NA	NA
G34504	-0.921447579	NA	NA
SVIL	-0.954919187	NA	NA
RPS3	27.74697694	NA	NA
SLC39A8	5.599057243	NA	NA
XIRP1	0.106927655	NA	NA
CSRP3	0.633426213	NA	NA
TNNI3/DNAAF3	0.558446672	NA	NA
HNRNPH2	16.49157231	NA	NA
RPS15A	2.144063903	NA	NA
TUBA4A	0.948296173	NA	NA
MYL6B	0.909769603	NA	NA
LOC100847677	2.64016999	NA	NA
TNNT1	0.487153624	NA	NA
CRYAB	0.354060842	NA	NA
MYOT	-0.247161484	NA	NA
TNNC1	0.352394485	NA	NA
PLEC	0.642209915	NA	NA
EEF1A2	-0.166756208	NA	NA
CA3	0.286524051	NA	NA
MYL2	0.413699517	NA	NA
EIF4B	-7.230303932	NA	NA
