# Supplementary material for: Integrated multi-omics analysis reveals insights into Chinese forest musk deer (Moschus berezovskii) genome evolution and musk synthesis
Source: Front Cell Dev Biol. 2023 May 9;11:1156138. doi: 10.3389/fcell.2023.1156138 (PMC10203155; doi:10.3389/fcell.2023.1156138)
Supplement: Supplementary file 1 [file DataSheet1.zip › Data Sheet 1/Table S5_2023_The comparison of the upregulated DEGs_RE_C_1.pdf]

	J_muscle_1	J_muscle_2	A_muscle_3	A_muscle_1	A_muscle_2	J_musk_glan	J_musk_glan	A_musk_glan	A_musk_glan	A_musk_glan	Avg_muscle	Avg_J_musk_	Avg_A_musk_gland
MYO3B	0	0	0	0	0	0	9.911924772	0	23.92858435	0	0	4.955962386	7.976194785
GRIA1	0	0	0	0	6.28114324	0	7.433943579	0	9.939565809	20.06531736	1.256228648	3.716971789	10.00162772
SRD5A1	0	0	0	0	7.85142905	12.86802994	0.825993731	17.15248438	26.87364089	4.256279439	1.57028581	6.847011837	16.0941349
MFSD2A	0	0	0	0	21.98400134	9.651022458	3.303974924	29.83040762	0	31.01003591	4.396800268	6.477498691	20.28014785
TRIM6	0	0	0	4.627777477	0	0	4.955962386	26.84736686	6.994509273	27.96983631	0.925555495	2.477981193	20.60390415
CDH1	0	0	0	0	0	3.217007486	0	25.35584648	17.30220715	64.4522315	0	1.608503743	35.70342838
DSCAML1	0	0	0	0	0	0	42.95167401	0	64.05497966	63.23615166	0	21.47583701	42.43037711
P2RY6	8.502001556	0	6.643121628	25.19567737	1.57028581	28.95306737	18.99785581	64.88113658	26.50550882	74.18087022	8.382217273	23.97546159	55.18917188
OTUD7A	0	0	0	0	3.14057162	0	0	58.16929487	20.98352782	91.8140279	0.628114324	0	56.98895019
OTOF	1.417000259	0	2.214373876	0	17.27314391	3.217007486	6.607949848	54.44049392	78.41213027	62.62811174	4.180903609	4.912478667	65.16024531
SEZ6L2	4.251000778	0	23.2509257	0	0	12.86802994	36.34372416	5.966081525	30.55496156	163.5627384	5.500385295	24.60587705	66.69459384
ABCG8	0	0	0	0	0	0	0.825993731	35.05072896	78.0439982	89.98990814	0	0.412996865	67.69487843
TEX261	1.417000259	1.151941477	0	0	0	0	0	79.7963404	41.2307915	103.3667864	0.513788347	0	74.79797276
G20057	0	1.151941477	0	0	0	0	0.825993731	70.84721811	49.69782904	108.2311057	0.230388295	0.412996865	76.25871763
ST18	0	0	0	0	0	0	0.825993731	57.42353468	39.39013117	168.4270578	0	0.412996865	88.41357455
C1ORF53	0	0	0	0	0	0	0.825993731	34.30496877	70.68135686	163.5627384	0	0.412996865	89.51635469
OCA2	2.834000519	3.45582443	0	5.141974974	0	0	2.477981193	119.3216305	82.82971507	82.6934291	2.286359985	1.238990596	94.94825822
RBM11	0	0	0	0	0	0	0.825993731	110.3725082	146.5165627	74.78891014	0	0.412996865	110.559327
RDM1	0	0	0	6.170369969	32.97600201	61.12314223	9.085931041	161.8299614	145.0440344	27.36179639	7.829274396	35.10453664	111.4119307
C9H6ORF118	7.085001297	16.12718067	0	9.769752451	26.69485877	16.08503743	15.69388089	101.4233859	5.153848938	247.4722474	11.93535864	15.88945916	118.0164941
SLC38A8	0	0	0	0	0	0	0.825993731	156.60964	125.5330348	115.5275848	0	0.412996865	132.5567532
PEAK3	0	0	0	0	0	0	0.825993731	55.9320143	172.6539394	174.507457	0	0.412996865	134.3644702
CD164L2	0	0	0	0	0	0	0.825993731	17.15248438	404.2090096	0	0	0.412996865	140.4538313
PRDM12	0	1.151941477	0	0	0	3.217007486	0	63.3896162	121.8517142	252.3365667	0.230388295	1.608503743	145.859299
MAGI1	0	0	0	0	0	0	0	15.660964	107.4945636	315.5727184	0	0	146.2427487
ADCYAP1	1.417000259	0	0	0	0	0	0	60.40657544	171.9176753	208.5576925	0.283400052	0	146.9606477
OR10AH1	0	0	0	0	0	0	0.825993731	70.10145792	113.0165446	264.4973651	0	0.412996865	149.2051225
CNIH3	1.417000259	1.151941477	0	0	0	0	0.825993731	150.6435585	161.9781095	147.1456606	0.513788347	0.412996865	153.2557762
CCDC85C	7.085001297	10.36747329	18.82217795	13.88333243	9.42171486	19.30204492	21.47583701	61.15233563	13.62088648	395.2259479	11.91593996	20.38894096	156.66639
ENSMMSG00	0	0	0	0	0	0	0.825993731	128.2707528	161.9781095	195.7888542	0	0.412996865	162.0125722
LAMA1	0	0	0	0	0	0	0.825993731	102.9149063	115.9616011	289.4270018	0	0.412996865	169.4345031
ENSBTAG00	0	0	0	3.599382482	0	0	4.955962386	169.2875633	139.1539213	200.0451336	0.719876496	2.477981193	169.4955394
G34614	0	5.759707383	0	8.227159959	0	0	7.433943579	228.2026183	173.3902036	111.8793453	2.797373468	3.716971789	171.157389
SYK	21.25500389	33.40630282	38.75154283	154.7734467	21.98400134	154.4163593	123.0730659	213.2874145	77.67586613	225.5828103	54.03405952	138.7447126	172.1820303
KHDC3L	2.834000519	0	0	0	7.85142905	0	2.477981193	164.0672419	211.3078065	141.6733013	2.137085914	1.238990596	172.3494499
BANK1	0	0	0	0	0	0	0.825993731	117.8301101	205.4176934	198.8290538	0	0.412996865	174.0256191
CCDC158	4.251000778	0	7.750308566	6.684567466	0	25.73605989	0	84.27090154	240.3902397	197.612974	3.737175362	12.86802994	174.0913717
C19H17ORF9	0	0	0	0	0	0	0.825993731	125.287712	19.51099955	381.2410297	0	0.412996865	175.3465804
CDK18	8.502001556	0	16.60780407	26.22407237	18.84342972	0	44.60366147	194.6434098	168.2363546	173.2913772	14.03546154	22.30183074	178.7237138
STAG3	0	0	0	5.656172472	6.28114324	0	11.56391223	256.5415056	99.02752602	195.7888542	2.387463142	5.781956117	183.7859619
G13482	0	1.151941477	0	0	1.57028581	0	4.955962386	231.9314193	188.8517504	145.9295808	0.544445457	2.477981193	188.9042501
VEPH1	0	0	9.964682442	8.227159959	0	0	15.69388089	71.5929783	179.6484487	321.6531176	3.63836848	7.846940444	190.9648482
CFAP45	1.417000259	0	7.750308566	0	14.13257229	9.651022458	8.25993731	116.3385897	232.2913343	236.5275288	4.659976223	8.955479884	195.0524843
TLX2	1.417000259	2.303882953	0	12.34073994	0	9.651022458	11.56391223	282.6431122	124.4286386	195.1808143	3.21232463	10.60746735	200.7508551
PODXL2	1.417000259	0	0	0	14.13257229	0	6.607949848	213.2874145	69.94509273	328.9495966	3.10991451	3.303974924	204.0607013
ENSMMSG00	0	0	0	0	0	0	0.825993731	170.0333235	72.89014926	382.4571096	0	0.412996865	208.4601941
SLC5A8	0	0	0	0	0	0	0.825993731	183.4570069	277.5715785	170.8592175	0	0.412996865	210.6292676
SEMA3E	0	1.151941477	0	0	20.41371553	0	8.25993731	205.8298126	316.9617097	119.7838642	4.313131401	4.129968655	214.1917955
NLRP2	1.417000259	1.151941477	0	0	0	0	0.825993731	251.3211842	193.2693352	210.9898522	0.513788347	0.412996865	218.5267905
RAB32	51.01200934	40.31795168	59.78809465	127.5209794	59.67086078	212.3224941	118.1171035	269.2194288	117.0659973	285.1707224	67.66197916	165.2197988	223.8187162
PIMREG	0	0	0	0	0	0	0.825993731	86.50818211	361.1375577	226.1908502	0	0.412996865	224.6121967
SNRPD3	0	0	0	0	0	3.217007486	0.825993731	230.4398989	353.0386522	105.7989461	0	2.021500608	229.7591657
WNK4	0	16.12718067	0	9.255554953	0	0	0	264.7448677	17.30220715	418.3314649	5.076547125	0	233.4595132
BRSK2	0	4.607765907	0	0	17.27314391	0	5.781956117	284.1346326	385.0661421	35.26631535	4.376181963	2.890978058	234.8223633
ATP1A3	0	0	0	0	0	3.217007486	0	73.83025887	408.2584623	236.5275288	0	1.608503743	239.53875
CLDN17	0	0	0	0	0	0	0.825993731	141.6944362	218.6704478	358.7435527	0	0.412996865	239.7028122

ERICH2	0	0	0	0	0	3.217007486	0	101.4233859	228.2418815	402.5224269	0	1.608503743	244.0625648
PIK3C2G	0	0	0	4.627777477	0	9.651022458	5.781956117	239.3890212	120.747318	372.7284709	0.925555495	7.716489287	244.28827
RALGPS1	80.76901478	97.91502551	100.7540114	64.27468718	94.2171486	0	105.7271976	176.7451652	515.3848938	55.93967263	87.58597749	52.86359878	249.3565772
BHMT	1.417000259	0	0	0	0	0	0	134.2368343	25.40111262	588.5826424	0.283400052	0	249.4068631
ENSBTAG00(	1.417000259	1.151941477	0	0	0	3.217007486	0.825993731	96.20306459	140.6264496	529.6027702	0.513788347	2.021500608	255.4774281
OLFM2	1.417000259	1.151941477	0	0	23.55428715	9.651022458	7.433943579	332.609045	206.5220896	235.311449	5.224645777	8.542483018	258.1475279
CABLES1	52.4290096	64.50872269	49.82341221	112.6092519	37.68685944	96.51022458	101.5972289	312.4735199	197.3187879	269.9697244	63.41145117	99.05372674	259.9206774
FOXD4	0	1.151941477	0	0	0	0	0.825993731	178.9824457	63.31871552	555.7484867	0.230388295	0.412996865	266.0165493
MESP2	0	0	0	0	0	0	0.825993731	105.8979471	189.5880145	511.3615726	0	0.412996865	268.9491781
TMEM59L	2.834000519	2.303882953	0	0	36.11657363	19.30204492	5.781956117	414.642666	271.6814654	120.9999441	8.250891421	12.54200052	269.1080252
IL10	0	0	0	0	0	0	0.825993731	175.2536448	308.8628042	325.3013571	0	0.412996865	269.8059354
G24157	2.834000519	2.303882953	0	0	14.13257229	6.434014972	4.129968655	305.7616782	384.69801	121.607984	3.854091152	5.281991813	270.689224
MROH2A	0	0	0	0	0	3.217007486	0.825993731	106.6437073	181.8572411	527.1706105	0	2.021500608	271.8905196
FBXO36	0	0	0	8.227159959	0	0	9.085931041	396.7444214	95.71433742	339.2862753	1.645431992	4.54296552	277.2483447
S100Z	0	0	0	0	0	0	0.825993731	162.5757216	211.3078065	459.6781794	0	0.412996865	277.8539025
CELF6	0	6.91164886	9.964682442	14.91172742	3.14057162	0	21.47583701	288.6091938	424.0881412	142.2813412	6.985726069	10.7379185	284.9928921
ADGRA1	5.668001037	4.607765907	7.750308566	11.31234494	0	0	14.04189343	165.5587623	117.8022614	577.0298839	5.867684091	7.020946713	286.7969692
TEPP	1.417000259	0	0	0	0	0	0	150.6435585	262.1100317	457.8540596	0.283400052	0	290.20255
COX6B2	1.417000259	0	0	0	0	3.217007486	0	307.9989587	382.1210855	197.612974	0.283400052	1.608503743	295.9110061
KCNAB3	0	1.151941477	0	0	0	0	0.825993731	152.8808391	291.192465	451.1656205	0.230388295	0.412996865	298.4129749
HPCA	0	0	0	0	0	0	0.825993731	238.643261	202.4726368	458.4620996	0	0.412996865	299.8593325
LDOC1	0	0	0	0	20.41371553	0	9.085931041	334.1005654	434.027707	145.9295808	4.082743106	4.54296552	304.685951
RGS20	0	0	0	0	0	0	0.825993731	167.7960429	233.7638625	513.1856924	0	0.412996865	304.9151993
TMEM125	0	0	0	0	0	3.217007486	0.825993731	183.4570069	245.9122207	511.3615726	0	2.021500608	313.5769334
TSSK4	0	5.759707383	4.428747752	0	0	0	2.477981193	14.16944362	85.77477161	867.0649257	2.037691027	1.238990596	322.3363803
POSTN	189.8780348	8.063590336	21.03655182	202.593814	65.95200402	0	156.9388089	554.8455818	353.0386522	60.19595207	97.50479898	78.46940444	322.6933954
RPS26	0	1.151941477	0	0	0	3.217007486	0.825993731	322.9141625	272.0495975	404.9545866	0.230388295	2.021500608	333.3061156
FOXN4	0	0	0	0	0	0	0.825993731	271.4567094	198.4231841	538.1153291	0	0.412996865	335.9984075
DBH	0	0	0	0	9.42171486	0	5.781956117	398.2359418	502.8684035	107.6230658	1.884342972	2.890978058	336.2424704
TUFT1	9.919001816	0	0	35.99382482	50.24914592	77.20817966	46.25564893	521.2863732	120.3791859	375.7686705	19.23239451	61.7319143	339.1447432
BRSK1	51.01200934	25.34271249	68.64559015	84.32838958	18.84342972	141.5483294	61.12353609	286.3719132	565.0827228	189.1004151	49.63442625	101.3359327	346.8516837
CCDC92B	0	0	0	0	0	0	0.825993731	232.6771795	137.313261	671.8841114	0	0.412996865	347.2915173
G14658	0	0	0	0	0	0	0.825993731	166.3045225	381.0166893	508.9294129	0	0.412996865	352.0835416
GRIN2C	0	0	0	0	0	3.217007486	0.825993731	146.1689974	419.6705564	496.1605746	0	2.021500608	354.0000428
CD3D	1.417000259	0	0	0	0	0	0.825993731	146.9147576	263.58256	660.9393929	0.283400052	0.412996865	357.1455701
ZMYND15	1.417000259	0	6.643121628	0	0	16.08503743	3.303974924	57.42353468	524.5881955	493.120375	1.612024377	9.694506177	358.3773684
G13771	0	0	0	7.712962461	0	0	9.911924772	328.8802441	220.5111081	531.42689	1.542592492	4.955962386	360.2727474
CLDN23	0	1.151941477	3.321560814	0	0	6.434014972	4.129968655	121.5589111	31.29122569	932.1251971	0.894700458	5.281991813	361.6584446
FAM180A	1.417000259	1.151941477	0	0	0	0	0.825993731	348.270009	509.4947807	230.4471296	0.513788347	0.412996865	362.7373065
DPYSL5	0	0	0	0	0	0	0.825993731	161.0842012	392.7969155	553.924367	0	0.412996865	369.2684945
FOXJ1	0	1.151941477	0	0	0	0	0.825993731	207.321333	139.1539213	770.3865785	0.230388295	0.412996865	372.2872776
MYT1	0	0	4.428747752	0	0	0	4.129968655	11.93216305	666.6871733	449.3415008	0.88574955	2.064984327	375.9869457
MYB	0	11.51941477	0	41.64999729	9.42171486	28.95306737	38.82170536	584.6759894	424.8244053	119.7838642	12.51822538	33.88738636	376.4280863
ATP10B	2.834000519	0	0	1.028394995	9.42171486	0	4.955962386	316.2023208	187.01109	636.0097562	2.656822075	2.477981193	379.7410557
FGFR2	0	0	49.82341221	51.93394724	3.14057162	135.1143144	37.16971789	170.7790837	282.3572954	701.0700276	20.97958621	86.14201615	384.7354689
TBC1D30	56.68001037	35.71018578	76.39589872	143.4611018	59.67086078	80.42518715	175.9366647	307.2531985	38.28573497	815.3815325	74.38361149	128.1809259	386.9734887
TRIB3	0	2.303882953	0	0	28.26514458	0	9.085931041	485.4898841	140.9945817	544.1957283	6.113805507	4.54296552	390.2267313
ENSMMSG00(	0	0	2.214373876	0	0	0	0	919.522315	174.1264677	80.26126942	0.442874775	0	391.3033507
GFAP	1.417000259	1.151941477	0	0	0	0	0.825993731	283.3888724	654.170683	236.5275288	0.513788347	0.412996865	391.3623614
SYTL5	0	0	0	9.255554953	0	12.86802994	6.607949848	528.7439752	534.5277613	111.2713053	1.851110991	9.737989896	391.5143473
TRPV5	0	0	0	0	0	0	0.825993731	238.643261	562.1376663	379.41691	0	0.412996865	393.3992791
HSD17B6	0	0	0	0	0	0	0.825993731	328.8802441	379.176029	479.1354568	0	0.412996865	395.7305766
FAM184A	85.02001556	51.83736645	71.96715097	24.16728238	59.67086078	48.25511229	26.43179939	79.7963404	433.6595749	710.1906264	58.53253523	37.34345584	407.8821806
MC1R	0	1.151941477	0	0	0	3.217007486	0.825993731	247.5923833	39.39013117	950.9744346	0.230388295	2.021500608	412.6523164
TAC1	0	0	0	0	0	0	0.825993731	183.4570069	381.3848214	700.4619877	0	0.412996865	421.7679387
ISL2	0	0	0	0	0	0	0.825993731	376.6088963	741.786115	148.9697804	0	0.412996865	422.4549305
CDHR5	0	0	0	0	0	0	0.825993731	293.8295151	110.8077522	863.4166862	0	0.412996865	422.6846512

EMID1	4.251000778	0	1.107186938	4.113579979	54.96000335	67.55715721	10.7379185	380.3376972	55.58794212	854.9041273	12.88635421	39.14753785	430.2765889
GAL3ST2	0	1.151941477	0	0	0	0	0	216.2704553	295.6100498	812.9493728	0.230388295	0	441.6099593
ADGRB2	29.75700545	18.43106363	54.25215996	187.1678891	18.84342972	398.9089283	64.42751102	479.5238026	119.2747897	731.4720236	61.69030956	231.6682196	443.4235386
CCDC103	0	1.151941477	0	0	0	0	0.825993731	498.9135675	263.58256	598.9193211	0.230388295	0.412996865	453.8051495
MAGEL2	0	0	0	0	0	0	0.825993731	295.3210355	603.0003257	470.014858	0	0.412996865	456.1120731
CCL8	1.417000259	1.151941477	0	0	0	0	0.825993731	175.999405	304.4452194	904.1553608	0.513788347	0.412996865	461.5333284
TNK1	0	8.063590336	39.85872977	22.11049239	62.8114324	45.0381048	61.12353609	346.7784886	457.2200272	617.1605186	26.56884898	53.08082045	473.7196782
FAM181B	1.417000259	1.151941477	0	0	0	0	0.825993731	201.3552515	315.4891814	915.7081193	0.513788347	0.412996865	477.5175174
G10414	0	0	0	0	0	0	0.825993731	205.8298126	228.6100136	1012.386467	0	0.412996865	482.2754309
TP73	0	1.151941477	5.53593469	0	0	12.86802994	3.303974924	63.3896162	142.4671099	1250.130075	1.337575233	8.086002434	485.3289338
SLC5A1	0	0	0	28.79505986	0	16.08503743	21.47583701	824.8107708	0	653.6429138	5.759011971	18.78043722	492.8178949
CCDC65	1.417000259	1.151941477	0	0	0	3.217007486	0	467.5916395	739.2091905	291.2511216	0.513788347	1.608503743	499.3506505
CETN4	0	0	0	0	0	0	0.825993731	181.9654865	837.5004524	482.7836964	0	0.412996865	500.7498784
SH2D3A	11.33600207	8.063590336	31.00123426	42.67839229	4.71085743	35.38708235	42.95167401	303.5243976	89.08796021	1138.85877	19.55801528	39.16937818	510.4903759
SLC6A14	0	0	0	0	0	3.217007486	0.825993731	490.7102054	863.2696971	177.5476566	0	2.021500608	510.5091864
SHANK2	0	0	0	8.227159959	1.57028581	12.86802994	6.607949848	444.4730736	139.8901855	950.9744346	1.959489154	9.737989896	511.7792312
SCML4	0	0	0	20.0537024	0	0	21.47583701	730.0992266	262.8462958	554.5324069	4.01074048	10.7379185	515.8259765
IRX6	0	0	0	0	0	0	0.825993731	250.575424	513.9123655	789.235816	0	0.412996865	517.9078685
CDCP2	0	0	0	0	0	0	0.825993731	501.1508481	910.0224696	144.7135009	0	0.412996865	518.6289395
TGM7	0	0	0	0	0	3.217007486	0.825993731	273.69399	660.0607961	634.7936763	0	2.021500608	522.8494875
COPZ2	7.085001297	1.151941477	0	6.684567466	39.25714525	6.434014972	25.60580566	821.8277301	390.9562551	356.311393	10.8357311	16.01991032	523.0317927
CDHR2	0	0	0	0	0	3.217007486	0	254.304225	388.3793307	935.7734367	0	1.608503743	526.1523308
LYPD1	2.834000519	0	6.643121628	0	50.24914592	12.86802994	17.34586835	469.0831599	180.0165808	930.9091173	11.94525361	15.10694915	526.6696193
PLEKHA7	49.59500908	42.62183464	141.7199281	139.8617193	36.11657363	209.1054866	137.1149593	246.8466231	186.642958	1172.300965	81.98301294	173.110223	535.2635155
IFITM5	0	0	0	0	0	3.217007486	0.825993731	441.4900328	77.67586613	1096.904015	0	2.021500608	538.6899715
TTLL10	0	0	0	0	0	0	0.825993731	428.8121096	227.1374853	990.4970294	0	0.412996865	548.8155415
SYT2	0	0	0	0	0	0	0.825993731	370.6428147	83.19784714	1226.416518	0	0.412996865	560.0857267
DNAL1	0	0	0	0	0	0	0.825993731	292.3379947	524.5881955	881.0498439	0	0.412996865	565.9920113
TRPM8	0	0	0	0	0	0	0.825993731	463.8628386	249.5935414	985.0246702	0	0.412996865	566.16035
KRT80	0	0	0	0	207.2777269	122.2462845	4.129968655	1545.215115	29.08243329	145.9295808	41.45554538	63.18812656	573.409043
KCTD17	2.834000519	0	0	14.91172742	0	16.08503743	13.2158997	679.3875337	338.6815016	712.6227861	3.549145589	14.65046856	576.8972738
AZIN2	1.417000259	1.151941477	0	0	0	3.217007486	0.825993731	378.1004166	116.3297332	1250.738115	0.513788347	2.021500608	581.722755
ART4	0	1.151941477	5.53593469	0	0	0	5.781956117	64.88113658	341.6265582	1348.024502	1.337575233	2.890978058	584.8440657
MMP7	0	0	0	0	0	0	0.825993731	694.3027375	670.368494	390.9696685	0	0.412996865	585.2136333
M-SAA3.2	0	0	0	0	0	3.217007486	0.825993731	356.4733711	950.885129	478.5274169	0	2.021500608	595.2953057
GABRB3	0	0	0	0	54.96000335	0	18.99785581	718.9128238	492.9288377	600.7434408	10.99200067	9.498927906	604.1950341
LRFN1	2.834000519	1.151941477	0	11.31234494	0	16.08503743	9.085931041	698.0315384	278.3078426	845.7835285	3.059657388	12.58548424	607.3743032
GDF15	0	1.151941477	0	0	48.67886011	9.651022458	14.04189343	816.6074087	522.011271	499.2007742	9.966160318	11.84645794	612.6064846
FIGNL2	0	0	0	25.70987487	0	28.95306737	18.17186208	888.200387	151.3022795	842.7433289	5.141974974	23.56246473	627.4153318
C1H3ORF52	0	0	0	0	0	0	0.825993731	219.253496	328.0056717	1440.44657	0	0.412996865	662.5685793
VEZT	0	0	0	2.05678999	3.14057162	0	6.607949848	701.7603394	918.4895071	393.4018281	1.039472322	3.303974924	671.2172249
AOX2	0	0	0	5.141974974	3.14057162	0	8.25993731	678.6417735	295.9781819	1072.582419	1.656509319	4.129968655	682.4007913
DSB	0	0	0	0	0	0	0.825993731	347.5242488	998.3741657	715.6629857	0	0.412996865	687.1871334
KCND3	58.09701063	36.86212725	85.25339422	46.79197226	91.07657698	0	106.5531913	310.2362393	362.610086	1407.004375	63.61621627	53.27659565	693.2835666
TMEM150B	1.417000259	1.151941477	0	0	0	0	0.825993731	615.9979175	382.4892176	1107.240694	0.513788347	0.412996865	701.9092764
IL17REL	0	0	0	9.255554953	0	0	14.04189343	733.0822674	1140.473144	284.5626825	1.851110991	7.020946713	719.3726978
REPS2	0	2.303882953	0	0	98.92800603	0	28.08378685	1062.708272	312.9122569	793.4920954	20.2463778	14.04189343	723.0375413
EVA1A	0	0	7.750308566	0	20.41371553	0	11.56391223	279.6600715	87.61543194	1808.310722	5.632804819	5.781956117	725.1954084
SLC44A3	5.668001037	0	0	28.28086236	23.55428715	64.34014972	27.25779312	1138.030051	523.8519313	532.0349299	11.50063011	45.79897142	731.3056374
ZNF215	0	2.303882953	0	0	32.97600201	0	10.7379185	979.9288905	526.7969879	716.8790655	7.055976993	5.368959251	741.2016479
GPR39	2.834000519	2.303882953	0	11.31234494	18.84342972	12.86802994	24.77981193	835.2514135	74.7308096	1317.622506	7.058731627	18.82392094	742.5349098
DSG1	0	0	0	6.684567466	0	22.5190524	4.129968655	544.4049392	367.3958029	1363.2255	1.336913493	13.32451053	758.3420808
ENSMMSG0(	0	0	0	0	0	0	0.825993731	571.9980662	773.4454727	940.637756	0	0.412996865	762.0270983
TGFA	2.834000519	0	0	4.113579979	51.81943173	9.651022458	28.90978058	745.7601906	78.78026234	1472.064646	11.75340245	19.28040152	765.535033
ACOT4	0	13.82329772	108.5043199	72.50184714	92.64686279	0	175.9366647	395.9986612	896.7697152	1033.059824	57.49526551	87.96833235	775.2760667
BOLA-DYA	0	0	0	0	0	0	0.825993731	83.52514135	2261.435288	0	0	0.412996865	781.6534763
FAM163B	0	0	0	0	0	0	0.825993731	684.607855	294.5056536	1369.3059	0	0.412996865	782.8064694

CYP27C1	2.834000519	4.607765907	22.14373876	14.91172742	0	9.651022458	23.12782447	88.74546268	1757.094356	513.7937323	8.899446522	16.38942346	786.5445169
ISG20	1.417000259	0	0	0	0	3.217007486	0.825993731	651.7944066	1256.434745	530.2108101	0.283400052	2.021500608	812.8133204
BRINP2	1.417000259	0	0	0	0	0	0.825993731	323.6599227	617.7256084	1504.290762	0.283400052	0.412996865	815.225431
LOC787600	0	0	0	0	0	0	0.825993731	390.7783399	274.626522	1785.205205	0	0.412996865	816.8700222
KRT222	0	0	0	0	0	0	0.825993731	689.8281763	1003.159883	775.8589377	0	0.412996865	822.9489989
TRIM29	0	0	32.1084212	31.36604734	0	48.25511229	42.12568028	401.9647427	1025.615939	1053.733181	12.69489371	45.19039628	827.1046208
RMI2	0	1.151941477	0	0	0	0	0.825993731	543.659179	203.9451651	1746.89869	0.230388295	0.412996865	831.5010113
TUBGCP2	0	1.151941477	0	0	0	3.217007486	0.825993731	558.5743828	623.6157215	1320.662706	0.230388295	2.021500608	834.2842701
EDARADD	1.417000259	0	0	0	48.67886011	12.86802994	12.38990596	1060.470991	291.9287291	1168.652726	10.01917207	12.62896795	840.3508154
VTCN1	0	0	0	0	14.13257229	9.651022458	4.955962386	577.9641477	42.3351877	1954.240302	2.826514458	7.303492422	858.1798793
CWH43	0	0	0	0	0	3.217007486	0.825993731	627.1843203	181.120977	1800.406203	0	2.021500608	869.5705
SNRPE	1.417000259	0	0	0	0	0	0.825993731	233.4229397	2451.759566	0.60803992	0.283400052	0.412996865	895.2635152
TRIQK	0	0	0	0	0	0	0.825993731	885.9631065	822.7751697	1014.818626	0	0.412996865	907.8523008
ZNF383	1.417000259	0	0	0	0	0	0.825993731	875.5224638	1567.874473	286.3868023	0.283400052	0.412996865	909.9279131
DNAAF11	1.417000259	0	0	0	0	0	0.825993731	596.6081525	788.1707554	1361.401381	0.283400052	0.412996865	915.3934295
PTPRZ1	7.085001297	8.063590336	29.89404733	42.67839229	12.56228648	25.73605989	56.16757371	890.4376676	1380.127119	477.3113371	20.05666354	40.9518168	915.9587079
HNF1A	0	0	0	0	0	0	0.825993731	810.6413272	1327.116101	611.6881594	0	0.412996865	916.4818627
SPATA13	0	0	0	0	0	0	0.825993731	748.7432314	585.3299865	1450.783249	0	0.412996865	928.2854889
SCNN1G	0	0	0	0	0	3.217007486	0.825993731	680.879054	252.90673	1864.250394	0	2.021500608	932.6787261
ODAD2	2.834000519	0	0	0	59.67086078	0	20.64984327	1031.386344	639.4454004	1139.46681	12.50097226	10.32492164	936.7661846
SYNGR4	1.417000259	0	0	0	0	3.217007486	0.825993731	931.4544781	1176.918218	702.2861074	0.283400052	2.021500608	936.8862679
TMEM221	0	0	0	0	0	0	0.825993731	970.234008	1255.69848	601.9595207	0	0.412996865	942.6306697
PAK6	1.417000259	0	0	2.05678999	39.25714525	32.17007486	12.38990596	879.2512647	73.25828133	1877.627273	8.5461871	22.27999041	943.3789395
G30177	0	0	0	0	0	0	0.825993731	811.3870874	463.4782723	1560.838474	0	0.412996865	945.2346113
LOC784417	0	1.151941477	0	0	0	0	0.825993731	759.9296342	1005.368675	1072.582419	0.230388295	0.412996865	945.9602426
PLPPR2	4.251000778	2.303882953	12.17905632	40.6216023	127.1931506	93.29321709	47.9076364	1199.182387	917.0169789	726.6077042	37.30973859	70.60042674	947.6023565
SH2D4A	0	0	26.57248651	16.45431992	0	9.651022458	33.86574297	155.8638798	442.4947445	2303.255216	8.605361286	21.75838271	967.2046136
CHRNA9	0	0	0	0	0	0	0.825993731	863.5903007	1292.143555	749.1051813	0	0.412996865	968.279679
SLC29A4	0	0	0	0	0	0	0.825993731	469.0831599	705.7091724	1786.421285	0	0.412996865	987.0712056
TSNARE1	131.7810241	91.00337665	203.7223966	105.9246845	458.5234565	450.381048	232.1042384	532.4727761	39.75826323	2393.245125	198.1909877	341.2426432	988.4920546
CLCA1	0	0	0	0	0	0	0.825993731	1003.793217	1122.802804	874.3614047	0	0.412996865	1000.319142
STS	1.417000259	0	0	0	0	3.217007486	0.825993731	376.6088963	549.9893081	2098.953803	0.283400052	2.021500608	1008.517336
ACOXL	0	0	0	0	0	0	0.825993731	1028.403303	1709.237187	290.6430817	0	0.412996865	1009.427857
TMIGD2	0	0	0	0	0	3.217007486	0.825993731	1108.945403	1359.143591	563.6530057	0	2.021500608	1010.580667
ENSBTAG00(	0	0	0	0	73.80343307	0	21.47583701	1639.180899	507.6541204	941.2457959	14.76068661	10.7379185	1029.360272
NEK10	0	0	0	18.51110991	20.41371553	0	32.21375551	906.8443918	70.31322479	2115.978921	7.784965087	16.10687775	1031.045513
CELSR3	4.251000778	5.759707383	0	29.30925735	12.56228648	12.86802994	35.51773043	1206.639988	157.5605247	1732.913772	10.3764504	24.19288019	1032.371428
GUCA1A	0	0	0	0	0	3.217007486	0.825993731	120.0673907	2977.084026	0	0	2.021500608	1032.383805
NRG1	0	0	0	17.48271491	0	0	23.12782447	1166.368938	1404.791968	552.1002472	3.496542982	11.56391223	1041.087051
CALY	0	0	0	0	0	0	0.825993731	926.9799169	1831.457033	411.6430257	0	0.412996865	1056.693325
CATSPERG	14.17000259	9.215531813	77.50308566	20.5678999	103.6388635	80.42518715	85.90334802	401.2189826	196.5825238	2630.380693	45.01907668	83.16426759	1076.060733
LOC100300(	0	0	0	0	0	3.217007486	0.825993731	495.1847666	1971.715351	770.9946184	0	2.021500608	1079.298245
RTN4R	0	0	0	0	0	3.217007486	0	513.0830111	617.3574763	2116.586961	0	1.608503743	1082.342483
MSX2	0	1.151941477	0	0	0	0	0.825993731	835.9971737	654.9069472	1767.572047	0.230388295	0.412996865	1086.158723
FAM178B	1.417000259	1.151941477	0	0	0	0	0.825993731	404.9477835	367.7639349	2545.863144	0.513788347	0.412996865	1106.191621
SCUBE1	4.251000778	0	37.64435589	58.10431721	6.28114324	22.5190524	73.51344206	829.285332	496.9782904	2100.169883	21.25616342	48.01624723	1142.144502
CCL25	0	0	0	0	0	3.217007486	0.825993731	1191.724785	1500.874437	742.4167421	0	2.021500608	1145.005321
AIFM3	0	1.151941477	0	29.30925735	0	9.651022458	31.38776178	1537.011753	630.2420987	1292.08483	6.092239766	20.51939212	1153.112894
ELOVL4	0	0	0	20.5678999	0	28.95306737	11.56391223	671.9299318	213.1484668	2578.6973	4.113579979	20.2584898	1154.5919
ENSMMSG0(	0	0	0	0	0	0	0.825993731	898.6410297	1342.94578	1243.441636	0	0.412996865	1161.676149
ENSMMSG0(	0	0	0	0	0	0	0.825993731	759.9296342	145.0440344	2584.777699	0	0.412996865	1163.250456
PALM3	5.668001037	3.45582443	14.39343019	58.6185147	26.69485877	57.90613475	72.68744832	1607.113211	633.5552873	1278.707951	21.76612583	65.29679154	1173.125483
HMGN5	1.417000259	3.45582443	0	45.24937977	0	73.99117218	18.99785581	2088.874294	1022.670882	420.7636245	10.02444089	46.494514	1177.436267
POC1A	9.919001816	10.36747329	35.42998202	70.44505715	6.28114324	122.2462845	49.55962386	1164.131658	1173.973162	1196.014522	26.4885315	85.90295416	1178.039781
G31995	0	0	0	0	0	3.217007486	0.825993731	1229.012794	1718.440489	601.9595207	0	2.021500608	1183.137601
RASSF6	0	4.607765907	18.82217795	2.05678999	72.23314726	0	34.6917367	955.3188042	2229.039666	400.6983072	19.54397622	17.34586835	1195.018926
SEC14L3	0	0	0	0	0	0	0.825993731	1051.521869	1439.02825	1103.592455	0	0.412996865	1198.047524

BOLA-DOB	0	0	0	24.68147988	0	9.651022458	23.9538182	1962.095062	927.3246767	747.8891014	4.936295975	16.80242033	1212.43628
PLA2G3	0	0	0	0	0	3.217007486	0.825993731	405.6935437	1081.203881	2161.581915	0	2.021500608	1216.15978
V-MPL	0	0	0	0	0	0	0.825993731	1127.589408	736.264134	1788.853444	0	0.412996865	1217.568995
KAZN	42.51000778	20.73494658	140.6127411	108.495672	9.42171486	0	161.8947713	221.4907766	1039.604957	2419.998881	64.35501646	80.94738563	1227.031538
ENSMMSG00	0	1.151941477	0	0	39.25714525	3.217007486	17.34586835	1124.606367	530.4783085	2030.245292	8.081817345	10.28143792	1228.443323
NUMB	501.6180918	541.412494	717.4571358	273.0388711	982.9989171	83.64219464	723.5705083	627.9300805	428.873858	2651.054051	603.305102	403.6063515	1235.952663
G20171	0	0	0	0	0	0	0.825993731	919.522315	220.8792402	2624.300294	0	0.412996865	1254.900616
DSC1	0	0	0	0	0	3.217007486	0.825993731	1026.166022	784.1213027	1965.793061	0	2.021500608	1258.693462
DENND2D	7.085001297	9.215531813	49.82341221	85.87098207	14.13257229	125.463292	79.29539817	1239.453437	1422.094175	1125.481892	33.22549994	102.3793451	1262.343168
SHD	0	1.151941477	0	0	0	3.217007486	0.825993731	858.3699794	1522.226097	1451.999329	0.230388295	2.021500608	1277.531802
ENSMMSG00	0	0	0	0	0	0	0.825993731	1338.639542	1726.907526	775.2508978	0	0.412996865	1280.265989
TEKT4	0	1.151941477	0	0	0	3.217007486	0.825993731	941.8951208	276.0990502	2669.295248	0.230388295	2.021500608	1295.76314
GJB4	0	0	0	0	0	3.217007486	0.825993731	481.015323	1197.901746	2258.868302	0	2.021500608	1312.595124
DLX3	0	0	0	0	0	3.217007486	0.825993731	1281.216007	1729.484451	947.934235	0	2.021500608	1319.544898
KCTD14	0	0	0	15.94012242	12.56228648	41.82109732	20.64984327	1663.790985	319.1705021	1979.777979	5.70048178	31.2354703	1320.913155
KRT6A	0	0	0	0	0	3.217007486	0.825993731	771.1160371	335.7364451	2888.797659	0	2.021500608	1331.88338
PLCH2	2.834000519	0	0	44.73518227	0	86.85920212	14.04189343	1983.722107	707.5498328	1327.959185	9.513836559	50.45054777	1339.743708
GAT	0	0	0	0	0	0	0.825993731	1246.165279	2578.028865	200.0451336	0	0.412996865	1341.413092
PTPRS	7.085001297	0	0	32.39444234	25.12457296	0	46.25564893	1747.316127	1051.385183	1336.471744	12.92080332	23.12782447	1378.391018
TSPOAP1	5.668001037	0	0	38.05061481	9.42171486	9.651022458	42.95167401	1744.333086	188.1154862	2222.993947	10.62806614	26.30134823	1385.147506
ITPRID1	0	0	0	0	10.99200067	3.217007486	5.781956117	941.1493606	117.0659973	3118.636749	2.198400134	4.499481801	1392.284036
FGF21	0	0	0	0	0	3.217007486	0	1287.182089	1343.682045	1590.02439	0	1.608503743	1406.962841
PDZD7	1.417000259	0	0	0	0	0	0.825993731	369.8970545	520.5387427	3332.058761	0.283400052	0.412996865	1407.498186
DPYSL4	1.417000259	1.151941477	0	0	0	0	0.825993731	662.2350493	1155.566558	2406.013963	0.513788347	0.412996865	1407.938523
AADAT	65.18201193	61.05289826	197.079275	166.0857917	260.6674445	0	371.6971789	1375.181792	1937.110936	925.436758	150.0134843	185.8485895	1412.576495
ENSMMSG00	0	1.151941477	0	0	0	0	0.825993731	1082.098037	1386.753496	1791.285604	0.230388295	0.412996865	1420.045712
EPB41L4B	5.668001037	0	13.28624326	32.90863983	20.41371553	22.5190524	49.55962386	1422.164684	281.9891633	2573.832981	14.45531993	36.03933813	1425.995609
GSTT4	0	0	0	0	0	0	0.825993731	910.5731928	365.5551425	3116.204589	0	0.412996865	1464.110975
LRRC1	9.919001816	20.73494658	50.93059915	46.27777477	92.64686279	38.60408983	117.2911098	1739.858525	1744.945998	991.1050694	44.10183702	77.94759981	1491.969864
ADRB1	0	0	0	0	0	0	0.825993731	1235.724636	349.7254636	3013.445843	0	0.412996865	1532.965314
DNAH9	2.834000519	2.303882953	0	32.39444234	43.96800268	9.651022458	48.73363013	2153.755431	120.0110538	2363.451168	16.3000657	29.19232629	1545.739218
GJB2	0	0	0	25.19567737	6.28114324	67.55715721	14.04189343	1850.231033	482.9892719	2315.416015	6.295364123	40.79952532	1549.54544
WNT3A	0	0	0	0	0	0	0.825993731	1469.147576	2918.182895	274.8340438	0	0.412996865	1554.054838
CAMK2N2	0	0	0	0	0	0	0.825993731	1619.045374	2301.929815	794.1001353	0	0.412996865	1571.691775
PRR36	0	2.303882953	0	19.5395049	21.98400134	51.47211978	16.51987462	2284.263464	1001.687354	1442.87873	8.765477839	33.9959972	1576.276516
MAB21L3	1.417000259	0	0	0	0	0	0.825993731	882.9800657	122.2198462	3775.927902	0.283400052	0.412996865	1593.709271
TMEM190	0	0	0	0	0	3.217007486	0.825993731	1618.299614	2542.320055	634.1856364	0	2.021500608	1598.268435
FUT1	0	0	0	20.0537024	9.42171486	51.47211978	9.911924772	2475.178073	2085.836292	301.5878002	5.895083452	30.69202227	1620.867388
COL26A1	0	1.151941477	0	0	0	3.217007486	0.825993731	1688.401072	2424.517793	769.1704986	0.230388295	2.021500608	1627.363121
COLCA2	2.834000519	0	14.39343019	20.5678999	0	0	33.03974924	1246.911039	2318.495758	1324.310945	7.559066122	16.51987462	1629.905914
LCN2	1.417000259	0	0	0	0	3.217007486	0.825993731	1735.383964	991.7477885	2227.250226	0.283400052	2.021500608	1651.460659
LRATD1	14.17000259	9.215531813	32.1084212	28.28086236	76.94400469	28.95306737	73.51344206	1654.841863	2967.14446	333.813916	32.14376453	51.23325471	1651.933413
KCNH8	0	0	0	0	0	0	0.825993731	763.6584352	1862.748259	2447.360677	0	0.412996865	1691.255791
QDPR	398.1770729	336.3669112	377.5507458	299.777141	1249.947505	1447.653369	489.8142825	1321.487058	107.4945636	3657.968158	532.3638751	968.7338256	1695.649926
GSTO2	1.417000259	0	0	0	0	0	0.825993731	1805.485422	2199.220968	1083.527137	0.283400052	0.412996865	1696.077842
C9	0	0	0	0	0	0	0.825993731	1176.809581	517.2255541	3412.32003	0	0.412996865	1702.118388
ATP13A4	0	0	0	5.141974974	69.09257564	16.08503743	32.21375551	2340.195478	2628.462958	201.2612135	14.84691012	24.14939647	1723.30655
BCL2L14	0	0	0	13.36913493	0	0	15.69388089	2151.51815	509.4947807	2621.260094	2.673826987	7.846940444	1760.757675
LIPH	0	0	0	0	0	3.217007486	0.825993731	1363.995389	1104.396201	2830.425827	0	2.021500608	1766.272472
CRYBA4	1.417000259	1.151941477	0	0	0	3.217007486	0.825993731	727.861946	1293.247951	3280.375368	0.513788347	2.021500608	1767.161755
BICDL1	0	0	0	0	0	0	0.825993731	1319.995537	711.9674176	3389.822553	0	0.412996865	1807.261836
G33790	0	0	0	0	0	0	0.825993731	1163.385897	154.6154681	4138.319695	0	0.412996865	1818.773687
SYT5	9.919001816	11.51941477	63.10965546	103.353697	7.85142905	86.85920212	106.5531913	1527.31687	1372.764478	2577.48122	39.15063962	96.70619671	1825.854189
TCHH	0	0	0	95.64073452	0	16.08503743	95.81527279	2708.601012	1035.187372	1811.350921	19.1281469	55.95015511	1851.713102
VAV2	63.76501167	73.7242545	284.5470431	240.1302313	257.5268728	0	499.7262072	1714.502678	3405.589752	596.4871614	183.9386827	249.8631036	1905.52653
CDCP1	0	0	0	22.11049239	7.85142905	12.86802994	30.56176805	2088.874294	629.5058345	3004.933284	5.992384288	21.71489899	1907.771137
OVOL3	0	0	0	0	0	3.217007486	0	956.0645644	2711.292673	2066.119648	0	1.608503743	1911.158962

INSRR	0	0	0	0	0	0	0.825993731	2109.755579	1556.830511	2070.983967	0	0.412996865	1912.523353
RASL10A	4.251000778	5.759707383	33.21560814	22.62468989	31.4057162	0	56.16757371	833.0141329	106.0220353	4801.083207	19.45134448	28.08378685	1913.373125
CHRM3	2.834000519	0	0	23.13888738	4.71085743	0	27.25779312	2815.99048	2346.473795	608.6479598	6.136749066	13.62889656	1923.704078
VSNL1	0	0	0	0	0	0	0.825993731	2028.467718	1809.737241	1958.496582	0	0.412996865	1932.233847
POU2F3	0	0	0	4.113579979	21.98400134	12.86802994	17.34586835	2119.450462	3222.996246	527.1706105	5.219516264	15.10694915	1956.539106
ARHGEF16	0	6.91164886	36.53716895	93.06974703	14.13257229	109.3782545	84.25136056	2177.619757	967.8192041	2748.340438	30.13022743	96.81480754	1964.593133
LOC509506	0	0	0	0	0	0	0.825993731	931.4544781	2160.935233	2835.290146	0	0.412996865	1975.893286
IGSF5	0	0	0	0	59.67086078	0	18.17186208	3035.243976	1917.968069	992.9291891	11.93417216	9.085931041	1982.047078
LOC529036	0	1.151941477	0	0	29.83543039	32.17007486	3.303974924	1773.417733	174.8627318	4048.329786	6.197474373	17.73702489	1998.870084
ENSMMSG0(	1.417000259	1.151941477	0	0	0	3.217007486	0.825993731	1185.012943	4811.854248	0	0.513788347	2.021500608	1998.95573
ARHGEF38	0	0	0	3.599382482	69.09257564	0	32.21375551	2473.686552	797.7421892	2792.119312	14.53839162	16.10687775	2021.182684
ITPKC	0	1.151941477	0	0	0	3.217007486	0.825993731	1885.281762	1058.011561	3124.717148	0.230388295	2.021500608	2022.670157
TMEM79	12.75300233	14.9752392	71.96715097	116.7228319	10.99200067	102.9442396	109.8571662	1756.265249	1511.182135	2842.586625	45.48204502	106.4007029	2036.678003
SLC13A1	0	0	0	0	0	0	0.825993731	1315.520976	1127.588521	3729.716868	0	0.412996865	2057.608789
SYNPR	0	0	0	0	0	0	0.825993731	936.6747994	1459.643646	3788.088701	0	0.412996865	2061.469049
ACE2	0	0	8.857495504	67.35987216	7.85142905	6.434014972	72.68744832	3225.412824	2067.79782	933.9493169	16.81375934	39.56073165	2075.719987
DKK1	0	0	0	4.627777477	0	0	8.25993731	2349.1446	3117.342343	764.9142192	0.925555495	4.129968655	2077.133721
TMEM130	0	1.151941477	0	0	0	0	0.825993731	1674.977388	3491.732655	1076.838698	0.230388295	0.412996865	2081.182914
MCTP2	0	10.36747329	34.32279508	55.01913222	17.27314391	12.86802994	75.99142325	1531.045671	267.2638806	4476.99793	23.3965089	44.4297266	2091.769161
CES2	2.834000519	1.151941477	9.964682442	0	0	6.434014972	9.911924772	76.06753944	1159.984143	5094.766488	2.790124887	8.172969872	2110.272724
TFAP2B	0	0	6.643121628	0	0	9.651022458	4.955962386	43.99985125	3395.650186	2905.822777	1.328624326	7.303492422	2115.157605
CCN4	1.417000259	1.151941477	0	0	0	3.217007486	0.825993731	1695.858673	922.5389599	3771.063583	0.513788347	2.021500608	2129.820405
CASKIN1	4.251000778	3.45582443	49.82341221	32.39444234	0	16.08503743	67.73148594	462.3713182	1742.737205	4216.756844	17.98493595	41.90826168	2140.621789
CCIN	0	0	0	0	0	0	0.825993731	2025.484678	2874.375179	1634.411305	0	0.412996865	2178.090387
BLNK	1.417000259	0	0	46.79197226	0	0	47.9076364	3352.192057	856.6433199	2403.581803	9.641794505	23.9538182	2204.13906
CDS1	0	0	0	25.19567737	21.98400134	0	47.08164266	3302.971884	1742.000941	1605.225388	9.435935743	23.54082133	2216.732738
S100A5	0	0	0	0	0	3.217007486	0.825993731	1390.842756	739.5773226	4574.284317	0	2.021500608	2234.901465
SEPTIN10	478.9460877	463.0804736	760.6374264	204.650604	3226.93734	398.9089283	1399.23338	2324.534514	3725.496518	707.7584667	1026.850386	899.0711543	2252.5965
TMEM54	0	1.151941477	0	19.0253074	0	48.25511229	6.607949848	3159.040167	2530.907961	1131.562291	4.035449776	27.43153107	2273.836806
TCN1	0	0	0	0	0	0	0.825993731	1568.333681	1340.000724	3995.430313	0	0.412996865	2301.254906
ENSMMSG0(	0	0	0	46.27777477	9.42171486	25.73605989	47.9076364	2807.041358	316.9617097	3803.289699	11.13989793	36.82184814	2309.097589
BREH1	1.417000259	0	0	0	0	3.217007486	0.825993731	2247.721215	2433.721095	2349.46625	0.283400052	2.021500608	2343.636187
ACOT2	0	0	0	0	0	0	0.825993731	1474.367897	471.2090457	5134.897123	0	0.412996865	2360.158022
FHAD1	1.417000259	0	0	0	0	0	0.825993731	1906.163047	886.4620173	4361.470345	0.283400052	0.412996865	2384.69847
NRN1	4.251000778	0	24.35811264	23.13888738	20.41371553	22.5190524	46.25564893	1460.944213	182.2253732	5525.866792	14.43234327	34.38735067	2389.678793
BEND6	0	0	0	0	0	0	0.825993731	2243.992414	4415.744144	525.9545307	0	0.412996865	2395.230363
NRG2	0	2.303882953	0	43.19258978	6.28114324	28.95306737	43.77766774	2924.871468	914.0719223	3393.470793	10.3555232	36.36536756	2410.804728
VIPR1	11.33600207	13.82329772	64.2168424	100.268512	0	0	153.634834	1404.266439	1510.077739	4327.42011	37.92893084	76.81741698	2413.921429
PAX3	0	0	0	0	0	0	0.825993731	1712.265398	374.0221801	5348.319135	0	0.412996865	2478.202238
CELSR2	0	0	0	30.33765235	0	0	42.95167401	1876.33264	1585.17668	3979.013236	6.067530469	21.47583701	2480.174185
G7298	0	1.151941477	0	0	0	3.217007486	0.825993731	2722.024696	3587.446993	1175.949205	0.230388295	2.021500608	2495.140298
DNAH2	45.3440083	31.10241987	57.57372077	203.1080115	26.69485877	93.29321709	202.3684641	2942.769712	1172.868765	3390.430593	72.76460384	147.8308406	2502.023024
TNFRSF18	1.417000259	0	0	0	0	3.217007486	0.825993731	2615.380989	3830.782289	1148.587409	0.283400052	2.021500608	2531.583562
CCDC96	1.417000259	1.151941477	0	0	0	3.217007486	0.825993731	2542.29649	1555.726115	3514.470737	0.513788347	2.021500608	2537.497781
FNDC10	12.75300233	10.36747329	24.35811264	33.42283733	144.4662945	225.190524	69.3834734	3764.597442	2634.353071	1362.00942	45.07354402	147.2869987	2586.986645
STK33	0	0	0	0	0	0	0.825993731	1846.502232	2210.26493	3709.043511	0	0.412996865	2588.603558
ELF5	0	3.45582443	28.78686039	19.0253074	287.3623032	73.99117218	114.8131286	2830.159923	459.4288196	4567.595878	67.72605909	94.40215039	2619.06154
KRT83	0	0	12.17905632	0	127.1931506	54.68912726	28.08378685	2544.53377	1134.58303	4251.41512	27.87444139	41.38645706	2643.51064
OSBPL6	0	0	5.53593469	0	0	9.651022458	4.129968655	42.50833087	4869.28285	3094.923192	1.107186938	6.890495556	2668.904791
PSPH	36.84200674	27.64659544	79.71745953	142.9469043	53.38971754	209.1054866	146.2008904	2759.312705	1256.066613	4011.847391	68.10853671	177.6531885	2675.742236
HGSNAT	0	0	0	0	0	3.217007486	0	1121.623327	3062.858797	3952.259479	0	1.608503743	2712.247201
RCE1	7.085001297	5.759707383	38.75154283	128.0351769	86.36571955	25.73605989	179.2406396	3634.089409	1997.484595	2635.245013	53.19942958	102.4883498	2755.606339
ENSBTAG00	1.417000259	1.151941477	0	0	0	3.217007486	0.825993731	2663.109641	3082.001665	2643.757572	0.513788347	2.021500608	2796.289626
PNCK	0	1.151941477	0	0	0	0	0.825993731	2516.940643	1949.995559	3926.721802	0.230388295	0.412996865	2797.886002
TPD52L1	0	0	0	21.59629489	51.81943173	102.9442396	26.43179939	4314.968463	3636.408558	459.6781794	14.68314532	64.68801947	2803.685067
DOP1B	454.8570833	630.1119877	677.598406	482.3172526	827.5406219	180.1524192	841.6876119	1727.926362	933.2147898	5981.288692	614.4850703	510.9200155	2880.809948
SLC22A15	0	0	0	34.96542982	0	0	35.51773043	4633.408064	1398.533722	2861.435863	6.993085965	17.75886522	2964.459217

OTX1	0	0	0	9.769752451	0	3.217007486	14.86788716	2287.246505	297.0825781	6351.585003	1.95395049	9.042447322	2978.638028
RASEF	0	0	0	1.542592492	59.67086078	3.217007486	23.9538182	3288.802441	2789.336672	2945.345372	12.24269065	13.58541284	3007.828161
IRX1	0	0	8.857495504	0	0	16.08503743	4.955962386	242.372062	4124.551679	4881.952516	1.771499101	10.52049991	3082.958752
AP1M2	0	0	0	0	0	0	0.825993731	3124.735199	5164.8929	968.6075923	0	0.412996865	3086.078564
GRB7	0	4.607765907	46.50185139	6.684567466	205.7074411	141.5483294	52.86359878	2038.908361	695.4014745	6544.941697	52.70032518	97.20596408	3093.083844
DNAJC5B	0	0	0	0	0	0	0.825993731	2897.278341	5582.722796	809.3011333	0	0.412996865	3096.43409
INPP5J	1.417000259	1.151941477	0	0	0	3.217007486	0.825993731	3143.379203	4370.832031	1827.767999	0.513788347	2.021500608	3113.993078
MAST1	0	1.151941477	0	0	0	3.217007486	0.825993731	3015.108451	3048.501647	3293.144206	0.230388295	2.021500608	3118.918101
CLDN1	4.251000778	8.063590336	0	3.085184984	260.6674445	12.86802994	75.99142325	4144.189379	802.159774	4427.138656	55.21344411	44.4297266	3124.495937
SOWAHB	0	0	0	0	0	0	0.825993731	2193.280721	421.8793488	6819.775741	0	0.412996865	3144.978603
G11978	0	0	0	42.16419479	17.27314391	0	55.34157997	3600.5302	287.5111443	5653.555175	11.88746774	27.67078999	3180.532173
STAC2	7.085001297	8.063590336	0	7.712962461	211.9885844	0	80.94738563	4582.696371	1145.626992	3900.576086	46.97002769	40.47369282	3209.63315
RTN4RL2	0	0	0	0	0	0	0.825993731	3107.582714	1074.209371	5738.680764	0	0.412996865	3306.824283
CBS	260.7280477	392.8120435	269.0464259	76.61542711	3577.111075	295.9646887	1080.3998	3772.800804	646.4399096	5607.344141	915.2626039	688.1822444	3342.194952
WNT10A	0	0	0	0	0	3.217007486	0.825993731	2559.448974	795.1652647	6708.504436	0	2.021500608	3354.372892
GRHL2	0	0	0	1.542592492	59.67086078	6.434014972	25.60580566	4198.629873	5038.991733	1002.657828	12.24269065	16.01991032	3413.426478
EPHB3	58.09701063	31.10241987	100.7540114	253.4993662	34.54628782	183.3694267	249.4501068	4253.816127	4208.48579	2060.647288	95.59981918	216.4097667	3507.649735
SARDH	5.668001037	2.303882953	18.82217795	0	169.5908675	3.217007486	61.94952982	3021.074532	1772.924035	5896.163103	39.27698588	32.58326865	3563.387223
RAB11FIP4	1.417000259	0	0	21.59629489	0	0	27.25779312	3570.699793	1943.369182	5176.243838	4.60265903	13.62889656	3563.437604
ZBTB42	17.00400311	11.51941477	76.39589872	141.4043118	23.55428715	38.60408983	175.9366647	2825.685362	716.0168703	7371.267948	53.97558311	107.2703773	3637.656727
SEC14L4	0	9.215531813	128.4336848	116.2086344	17.27314391	160.8503743	139.5929405	1666.028266	6290.640761	3215.923136	54.22619899	150.2216574	3724.197388
BPTF	31.17400571	0	0	0	0	0	0	0	5212.381936	6195.926783	6.234801141	0	3802.769573
CAMSAP3	70.85001297	111.7383232	269.0464259	521.3962624	81.65486212	781.7328191	349.3953482	3597.54716	1233.610556	6700.599917	210.9371773	565.5640836	3843.919211
CRISPLD1	1.417000259	1.151941477	0	0	0	0	0.825993731	3566.225232	3377.611715	4629.00791	0.513788347	0.412996865	3857.614952
IRX5	0	17.27912215	238.0451917	41.13579979	332.9005917	164.0673818	234.5822196	472.8119609	8824.493778	2345.209971	125.8721411	199.3248007	3880.83857
ESRP1	0	0	0	10.28394995	91.07657698	35.38708235	52.03760505	5683.438413	4314.139693	1729.265532	20.27210539	43.7123437	3908.947879
ENSMMSG00	0	0	0	0	0	0	0.825993731	4052.460876	3088.628042	4751.831974	0	0.412996865	3964.306964
PPP2R2B	5.668001037	4.607765907	0	13.36913493	292.0731607	28.95306737	108.2051788	6534.35079	4418.6892	955.2307141	63.14361251	68.57912307	3969.423568
MYO5B	17.00400311	12.67135624	64.2168424	211.3351714	54.96000335	289.5306737	198.2384954	5350.829368	2319.968286	4265.400038	72.03747531	243.8845846	3978.732564
CLDN7	7.085001297	4.607765907	26.57248651	92.04135204	21.98400134	90.07620961	91.68530414	5417.202025	3612.111841	2910.687096	30.45812142	90.88075687	3980.000321
FGFR3	0	0	8.857495504	0	0	0	9.085931041	17.89824457	844.8630937	11125.91445	1.771499101	4.54296552	3996.225264
PCYT2	43.92700804	39.16601021	11.07186938	183.5685066	39.25714525	96.51022458	162.720765	6453.80869	2273.583646	3415.96827	63.39810789	129.6154948	4047.786868
DYNC1I2	0	0	0	5.141974974	25.12457296	25.73605989	16.51987462	5406.761382	3188.0237	3567.97825	6.053309587	21.12796725	4054.254444
ASCL2	0	0	21.03655182	0	97.35772022	38.60408983	27.25779312	2179.857037	637.236608	9665.402566	23.67885441	32.93094148	4160.83207
MYMK	4.251000778	0	0	8.741357456	81.65486212	0	52.86359878	5010.016961	6798.663013	779.5071772	18.92944407	26.43179939	4196.062384
STEAP4	28.34000519	17.27912215	119.5761893	94.61233952	169.5908675	38.60408983	244.4941444	4268.731331	7013.284008	1318.230546	85.87970473	141.5491171	4200.081962
RELCH	12.75300233	0	0	68.38826716	6.28114324	41.82109732	59.47154863	6160.724935	2219.1001	4233.781962	17.48448255	50.64632297	4204.535665
MOCOS	12.75300233	9.215531813	109.6115069	187.6820866	31.4057162	99.72723207	221.3663199	3223.921304	954.5664497	8505.262399	70.13356875	160.546776	4227.916718
RNASE4	0	0	0	0	0	0	0.825993731	4528.255877	5480.382081	2741.043959	0	0.412996865	4249.893972
KIF12/AMBP	0	0	0	0	0	3.217007486	0.825993731	2326.026035	6885.174049	3981.445395	0	2.021500608	4397.548493
FAM110A	5.668001037	8.063590336	28.78686039	91.52715454	7.85142905	57.90613475	87.55533548	4923.508779	1754.149299	6569.263294	28.37940707	72.73073512	4415.640457
OGDHL	0	1.151941477	0	0	0	0	0.825993731	2868.193693	2656.440995	7811.48885	0.230388295	0.412996865	4445.374513
ENSBTAG00	0	0	0	0	0	3.217007486	0.825993731	540.6761382	12855.90804	0	0	2.021500608	4465.528061
PTK7	28.34000519	19.5830051	143.9343019	159.4012242	14.13257229	276.6626438	134.6369781	2111.99286	2007.792293	9363.814766	73.07822174	205.649811	4494.533306
IQGAP2	0	17.27912215	183.7930317	170.7135691	48.67886011	0	270.9259438	2529.618567	8683.131064	2425.47124	84.09291662	135.4629719	4546.073624
TMEM238	0	0	0	0	0	3.217007486	0.825993731	4254.561888	1932.32522	7484.971413	0	2.021500608	4557.286174
TINCR	0	0	0	0	0	3.217007486	0.825993731	4677.407916	7791.515198	1239.793397	0	2.021500608	4569.57217
NHLH2	0	0	0	0	0	0	0.825993731	2815.24472	264.3188241	10747.10558	0	0.412996865	4608.889709
ANO4	2.834000519	2.303882953	45.39466446	63.24629218	4.71085743	12.86802994	86.72934175	2803.312557	1974.292275	9053.714407	23.69793951	49.79868585	4610.439746
BPIFB1	0	0	0	0	28.26514458	0	9.085931041	6023.50506	81.3571868	7820.001409	5.653028916	4.54296552	4641.621219
C4BPB	0	0	0	0	0	3.217007486	0.825993731	4876.525886	5257.662181	3859.229371	0	2.021500608	4664.47248
ANKRD22	0	0	0	0	0	3.217007486	0.825993731	4232.189082	7166.426948	2713.682162	0	2.021500608	4704.099397
ARRDC5	0	13.82329772	71.96715097	79.7006121	70.66286145	28.95306737	147.8528778	4326.900626	7832.009725	2052.742769	47.23078445	88.40297261	4737.217707
PRR7	1.417000259	1.151941477	0	0	0	3.217007486	0.825993731	4233.680602	5958.953768	4286.681435	0.513788347	2.021500608	4826.438602
CORO2A	0	2.303882953	34.32279508	35.99382482	0	0	65.25350475	2152.26391	4448.507897	8153.207285	14.52410057	32.62675237	4917.993031
TMEM91	0	1.151941477	0	0	0	0	0.825993731	1551.926957	3176.243474	10075.82951	0.230388295	0.412996865	4934.666648
CCDC159	2.834000519	3.45582443	68.64559015	20.5678999	208.8480127	183.3694267	83.42536683	4226.96876	3418.106242	7405.926224	60.87026555	133.3973968	5017.000409

USP32	0	207.3494658	181.5786578	392.846888	251.2457296	125.463292	572.4136556	5881.810623	1892.935088	7307.423757	206.6041482	348.9384738	5027.389823
KRT15	0	1.151941477	39.85872977	0	0	144.7653369	3.303974924	802.4379651	3259.073189	11218.33652	8.202134249	74.0346559	5093.282558
PPFIA4	0	0	5.53593469	0	0	0	5.781956117	7.457601906	1133.110502	14239.07884	1.107186938	2.890978058	5126.548982
SLC13A2	0	0	0	22.62468989	0	0	24.77981193	7274.89066	1070.159919	7163.926336	4.524937977	12.38990596	5169.658971
LAMC2	0	0	8.857495504	0	0	0	9.911924772	413.1511456	6740.866279	8558.161872	1.771499101	4.955962386	5237.393099
PCDH20	0	0	0	0	0	0	0.825993731	3431.988397	654.170683	11726.65789	0	0.412996865	5270.938992
NRTN	0	0	0	0	0	3.217007486	0.825993731	5313.541358	2720.495975	7867.428523	0	2.021500608	5300.488619
ARFGEF3	0	18.43106363	0	119.2938194	23.55428715	0	135.4629719	7529.194885	6175.783556	2521.541548	32.25583404	67.73148594	5408.839996
GRIK4	0	1.151941477	0	15.42592492	64.38171821	0	51.21161132	7840.176884	2632.512411	6077.358999	16.19191692	25.60580566	5516.682765
SLCO1A2	0	0	0	0	0	0	0.825993731	5824.387089	4649.876138	6080.399199	0	0.412996865	5518.220808
CDC42BPG	1633.801299	1749.799103	2940.688507	4733.187964	648.5280395	8907.893729	2059.202371	4485.001786	2404.270529	9689.724163	2341.200983	5483.54805	5526.33216
DSG3	0	0	0	11.31234494	0	6.434014972	12.38990596	6315.843054	5531.552439	4860.671119	2.262468989	9.411960468	5569.355537
PIP	0	0	0	0	0	3.217007486	0.825993731	4203.850195	7248.520399	5430.404524	0	2.021500608	5627.591706
LRRC8E	2.834000519	0	27.67967345	0	222.980585	19.30204492	44.60366147	4408.188487	3983.557097	8708.955772	50.6988518	31.95285319	5700.233785
PSAT1	0	3.45582443	93.00370279	73.01604463	56.53028916	22.5190524	147.8528778	4168.799466	7514.679883	5513.097953	45.2011722	85.18596512	5732.192434
FAM78B	0	1.151941477	0	0	0	3.217007486	0.825993731	2769.007588	5025.738979	9798.563308	0.230388295	2.021500608	5864.436625
DSC3	0	0	25.46529957	12.85493744	0	22.5190524	23.12782447	136.4741149	9403.933651	8125.237449	7.664047402	22.82343843	5888.548405
PERP	49.59500908	57.59707383	343.2279508	42.67839229	760.0183321	344.219801	346.0913733	2390.161411	6400.344117	8880.423029	250.6233516	345.1555871	5890.309519
NIPAL2	77.93501427	62.20483974	295.6189124	358.3956557	89.50629117	234.8415465	439.4286649	4441.747695	3897.414193	9455.628794	176.7321427	337.1351057	5931.596894
ATP6V1G3	0	0	9.964682442	6.170369969	78.5142905	32.17007486	42.12568028	8060.9219	8342.977034	1405.788295	18.92986858	37.14787757	5936.56241
ASNS	39.67600726	46.07765907	201.5080227	299.777141	287.3623032	466.4660855	421.2568028	6991.501787	7117.465383	3706.611351	174.8802267	443.8614441	5938.526174
GEMIN7	0	1.151941477	0	0	0	0	0.825993731	2314.093872	5027.579639	10488.08058	0.230388295	0.412996865	5943.251363
LOC782545	0	0	0	0	0	0	0.825993731	5856.454777	9711.692059	2353.11449	0	0.412996865	5973.753775
BAIAP2L1	4.251000778	0	45.39466446	15.94012242	268.5188735	38.60408983	136.2889656	6680.519788	2331.748512	9035.473209	66.82093223	87.44652772	6015.913836
LRRC15	1.417000259	0	0	0	0	0	0.825993731	3646.767332	3132.80389	11552.15044	0.283400052	0.412996865	6110.573886
SSPO	0	0	0	10.28394995	31.4057162	16.08503743	23.9538182	4972.728951	266.5276165	13261.95869	8.33793323	20.01942781	6167.071753
SLC25A5	174.2910319	150.9043334	325.5129598	878.2493256	243.3943006	488.9851379	990.3664834	8144.447042	5930.607599	4534.153682	354.4703902	739.6758106	6203.069441
CD44	787.8521442	481.5115372	869.1417463	1308.632631	1271.931506	3265.262598	1219.166747	6718.553557	2743.688295	9284.769576	943.813913	2242.214673	6249.00381
DSG2	0	0	0	3.599382482	36.11657363	0	25.60580566	6813.265102	2134.061592	9802.819588	7.943191223	12.80290283	6250.048761
LARGE2	4.251000778	0	0	40.6216023	124.052579	64.34014972	120.5950847	10128.16915	5061.079657	3679.857595	33.78503641	92.46761722	6289.702134
CRABP1	1.417000259	0	11.07186938	0	0	32.17007486	3.303974924	1089.555639	6037.365899	11890.22063	2.497773928	17.73702489	6339.04739
GALNT3	0	0	32.1084212	101.296907	43.96800268	22.5190524	140.4189343	8774.614403	8355.125392	1915.325748	35.47466617	81.46899333	6348.355181
CCDC180	0	0	0	13.88333243	0	0	20.64984327	5661.065607	528.2695161	13357.42096	2.776666486	10.32492164	6515.585361
CCND1	24.08900441	12.67135624	164.9708538	192.8240615	17.27314391	283.0966588	191.6305456	4003.240703	7723.410765	8000.589265	82.36568397	237.3636022	6575.746911
G9826	0	0	0	0	0	3.217007486	0.825993731	4479.035705	1081.572013	14290.1542	0	2.021500608	6616.920638
G20187	0	76.02813746	84.14620729	0	116.2011499	41.82109732	50.38561759	38.03376972	16049.08559	4728.118417	55.27509894	46.10335745	6938.412593
COL17A1	0	1.151941477	0	13.88333243	0	16.08503743	13.2158997	8442.005358	10433.96717	2006.531736	3.007054781	14.65046856	6960.834756
CLDN3	0	0	0	11.82654244	70.66286145	38.60408983	40.47369282	10129.66067	2700.248711	8340.483581	16.49788078	39.53889132	7056.797654
IGFBP2	2.834000519	2.303882953	0	5.656172472	417.6960255	276.6626438	23.9538182	12119.34886	4296.101222	5162.866959	85.69801628	150.308231	7192.772346
ISL1	0	0	0	0	0	0	0.825993731	6910.213926	3958.524116	10787.84426	0	0.412996865	7218.860767
ESYT3	0	0	83.03902035	8.227159959	675.2228983	51.47211978	224.6702948	6368.792028	1824.094392	13688.19468	153.2978157	138.0712073	7293.693699
RAP1GAP	7.085001297	5.759707383	52.03778608	14.39752993	105.2091493	70.77416469	70.20946713	5103.982745	6526.613416	10302.02036	36.89783479	70.49181591	7310.872174
CRACR2A	0	5.759707383	100.7540114	122.3790044	23.55428715	0	158.5907963	5100.999704	5763.843773	11156.31645	50.48940205	79.29539817	7340.386642
ARHGEF5	0	2.303882953	0	2.570987487	350.1737356	115.8122695	67.73148594	10639.01488	2475.320018	9231.262063	71.00972122	91.77187772	7448.53232
C8H9ORF152	0	0	0	29.30925735	0	0	34.6917367	9668.780871	6611.651923	6085.263518	5.86185147	17.34586835	7455.232104
LIPM	0	0	0	0	0	3.217007486	0.825993731	4725.136568	2983.710403	14986.96794	0	2.021500608	7565.271638
EVPL	2.834000519	1.151941477	121.7905632	84.84258707	180.5828682	534.0232427	36.34372416	6303.910891	9239.746749	7196.760491	78.24039208	285.1834834	7580.139377
TERT	0	0	0	0	0	3.217007486	0.825993731	7142.145346	12526.42984	3211.058817	0	2.021500608	7626.544669
SLC2A10	25.50600467	13.82329772	243.5811264	63.76048968	441.2503126	19.30204492	344.4393858	4791.509225	12322.11655	6200.183063	157.5842462	181.8707154	7771.269611
EPHA1	5.668001037	4.607765907	0	211.8493689	23.55428715	414.9939657	66.07949848	11479.48661	2034.297802	9830.789424	49.13588461	240.5367321	7781.524614
CIC	0	1.151941477	0	0	0	3.217007486	0	3810.088814	5970.365862	13868.78253	0.230388295	1.608503743	7883.079069
GSTA3	0	0	0	14.39752993	40.82743106	25.73605989	33.86574297	12458.66974	6281.805591	5211.510153	11.0449922	29.80090143	7983.995163
PRSS22	0	1.151941477	0	0	0	3.217007486	0.825993731	6806.55326	9840.170151	7327.489074	0.230388295	2.021500608	7991.404162
ENSMMSG00	0	0	0	0	0	3.217007486	0.825993731	6217.402709	2604.534374	15171.20404	0	2.021500608	7997.713708
TMEM184A	4.251000778	3.45582443	33.21560814	14.39752993	124.052579	9.651022458	94.16328533	8292.85332	2643.188241	13126.97383	35.87450845	51.90715389	8021.00513
LOC1049722	0	2.303882953	56.46653384	0	183.7234398	61.12314223	91.68530414	3158.294407	6772.525636	14348.52603	48.49877131	76.40422319	8093.115357
MAP7D2	0	0	0	0	0	3.217007486	0.825993731	8356.242936	12770.13327	3242.068853	0	2.021500608	8122.81502

SUSD2	2.834000519	1.151941477	143.9343019	99.240117	0	125.463292	213.1063826	2551.245612	16267.01978	5781.243558	49.43207219	169.2848373	8199.836315
SGPP2	0	0	0	11.82654244	6.28114324	6.434014972	23.12782447	10079.69474	11673.09971	2923.455935	3.621537136	14.78091972	8225.416794
MDFI	0	3.45582443	0	9.255554953	191.5748688	45.0381048	75.99142325	13416.22583	6793.141032	4650.897347	40.85724964	60.51476403	8286.754736
SLC52A3	0	0	0	14.39752993	21.98400134	3.217007486	36.34372416	10647.21824	13390.80394	1327.351145	7.276306254	19.78036582	8455.124441
FAM83H	21.25500389	19.5830051	221.4373876	393.3610855	28.26514458	234.8415465	447.6886022	6877.400478	4837.991624	13687.58664	136.7803253	341.2650743	8467.659579
EPS8L2	5.668001037	12.67135624	22.14373876	457.6357727	62.8114324	633.7504747	295.7057557	13916.63092	6545.756283	5365.344253	112.1860602	464.7281152	8609.243818
C21H15ORF3	94.93901738	80.63590336	386.4082414	532.1944098	100.4982918	167.2843893	645.9270976	7658.957158	11063.10488	7123.795701	238.9351728	406.6057434	8615.285912
LOC534967	7.085001297	6.91164886	106.289946	157.3444342	78.5142905	302.3987037	164.3727525	8814.139693	14730.0684	3216.531176	71.22906418	233.3857281	8920.246422
PAX9	0	0	0	0	0	0	0.825993731	7060.111725	2803.693822	17057.95191	0	0.412996865	8973.919153
CIITA	0	0	0	0	0	0	0.825993731	3179.175693	4139.645093	19973.50333	0	0.412996865	9097.441371
SLC17A3	0	0	0	0	0	0	0.825993731	6397.130915	1825.56692	19262.70466	0	0.412996865	9161.800832
LOC508879	4.251000778	5.759707383	57.57372077	109.0098695	7.85142905	19.30204492	134.6369781	8136.24368	5624.689852	15017.36994	36.88914549	76.96951153	9592.767824
CRB3	0	0	0	0	0	0	0.825993731	6825.197265	1771.819638	20227.05597	0	0.412996865	9608.024292
CCDC125	38.259007	47.22960054	187.1145925	258.6413412	233.9725857	67.55715721	484.8583201	11739.01116	8634.537631	8468.780004	153.0434254	276.2077386	9614.109599
RFX2	0	1.151941477	0	0	0	0	0.825993731	1168.606219	28064.54813	0	0.230388295	0.412996865	9744.384782
STXBP2	28.34000519	12.67135624	150.5774236	477.6894751	31.4057162	897.5450886	238.7121882	11944.09521	8917.263059	8435.945848	140.1367953	568.1286384	9765.76804
PRR15L	0	0	23.2509257	49.87715725	18.84342972	0	75.99142325	9109.460728	1621.253623	18641.8959	18.39430253	37.99571162	9790.870085
TMPRSS13	0	1.151941477	0	6.170369969	91.07657698	16.08503743	49.55962386	10858.26838	1089.302786	17825.29829	19.67977769	32.82233064	9924.289817
MAP7	11.33600207	4.607765907	34.32279508	13.88333243	331.3303059	51.47211978	152.8088402	13644.42845	13851.33715	2961.15441	79.09604028	102.14048	10152.30667
TRPM1	21.25500389	26.49465396	204.8295835	255.0419587	494.6400302	77.20817966	554.2417935	11683.07915	15061.75539	3712.691751	200.4522461	315.7249866	10152.50876
TBX5	1.417000259	0	0	0	0	3.217007486	0.825993731	10705.38754	13205.26537	6888.484252	0.283400052	2.021500608	10266.37905
TFCP2L1	0	0	0	0	0	3.217007486	0.825993731	7739.499258	2032.457142	21240.05048	0	2.021500608	10337.33563
EFS	48.17800882	28.79853692	332.1560814	535.7937923	65.95200402	373.1728684	554.2417935	8885.732671	6377.888061	15924.5655	202.1756847	463.7073309	10396.06208
CKMT1A	0	0	14.39343019	0	0	45.0381048	4.955962386	2071.72181	8856.8894	20822.32706	2.878686039	24.99703359	10583.64609
C16H1ORF11	8.502001556	12.67135624	0	124.9499919	95.78743441	112.595262	199.8904829	12180.50119	946.0994122	19781.97075	48.38215682	156.2428725	10969.52379
ENSBTAG00	0	0	0	0	0	3.217007486	0.825993731	11910.536	13041.07847	8096.659573	0	2.021500608	11016.09135
RNPEP	111.9430205	93.30725961	569.0940861	524.9956449	268.5188735	482.5511229	777.2601008	7174.958794	6231.73963	19752.1768	313.5717769	629.9056119	11052.95841
ELOVL6	4.251000778	5.759707383	0	332.1715833	42.39771687	22.5190524	333.7014673	18269.63315	8574.532104	6486.569865	76.91600167	178.1102599	11110.24504
WIF1	0	0	0	0	0	0	0.825993731	10722.54002	17072.12461	5561.741147	0	0.412996865	11118.80192
HSD17B14	1.417000259	3.45582443	35.42998202	77.64382211	0	19.30204492	100.7712352	10215.42309	8550.971652	15097.63121	23.58932576	60.03664005	11288.00865
CLDN4	2.834000519	3.45582443	1.107186938	93.58394453	12.56228648	16.08503743	97.46726025	15351.47352	3134.64455	15409.55569	22.70864858	56.77614884	11298.55792
AMZ2	0	9.215531813	126.2193109	463.2919452	0	656.2695271	316.355599	10732.98066	6603.553018	17563.84112	119.7453576	486.3125631	11633.45827
DEGS2	8.502001556	10.36747329	170.5067884	56.04752722	216.6994418	257.3605989	174.2846772	4396.256324	1519.28104	29935.62937	92.42464646	215.8226381	11950.38891
DSC2	0	0	0	48.33456476	26.69485877	0	67.73148594	18055.59998	12160.8747	6090.735877	15.00588471	33.86574297	12102.40352
TMEM72	0	0	0	0	0	0	0.825993731	8175.76897	1548.363474	26746.45999	0	0.412996865	12156.86415
RBM47	8.502001556	10.36747329	141.7199281	297.2061535	32.97600201	0	338.6574297	12363.9582	7630.273352	17821.65005	98.15431168	169.3287148	12605.29387
CFHR5	0	0	0	0	0	0	0.825993731	12093.24725	19315.88955	6651.348683	0	0.412996865	12686.8285
MLPH	0	2.303882953	0	5.656172472	263.8080161	0	88.38132921	14753.37385	1326.379837	22110.15561	54.3536143	44.19066461	12729.96976
DNASE1	26.92300493	35.71018578	150.5774236	547.1061372	196.2857263	183.3694267	712.0065961	16460.41893	4856.76636	16958.23336	191.3204956	447.6880114	12758.47288
PXMP4	66.59901219	46.07765907	359.8357548	570.2450246	83.22514793	54.68912726	671.5329033	12363.9582	14183.02414	12297.60738	225.1965197	363.1110153	12948.19657
DRC1	0	1.151941477	0	0	0	3.217007486	0.825993731	8699.292624	1101.451144	29267.3935	0.230388295	2.021500608	13022.71242
PRODH	19.83800363	16.12718067	110.7186938	73.01604463	303.0651613	411.7769582	190.8045519	14593.03541	5114.826939	19594.69446	104.5530168	301.290755	13100.85227
ESRP2	17.00400311	16.12718067	85.25339422	259.6697362	111.4902925	215.5395016	337.0054422	13632.49628	1802.3746	24315.51639	97.90892134	276.2724719	13250.12909
EPN3	0	3.45582443	124.0049371	8.227159959	1089.778352	395.6919208	247.7981193	14052.35927	7951.652647	17930.4892	245.0932547	321.74502	13311.50037
GABRP	0	0	0	0	0	3.217007486	0.825993731	7189.873998	856.6433199	31995.06058	0	2.021500608	13347.19263
CYP4F2	0	2.303882953	32.1084212	30.85184984	0	0	40.47369282	5305.337996	14999.54107	21550.75888	13.0528308	20.23684641	13951.87931
C23H6ORF13	11.33600207	10.36747329	200.4008358	208.7641839	31.4057162	228.4075315	223.8443011	6689.46891	10874.98939	24305.78776	92.45484226	226.1259163	13956.74869
DHCR24	38.259007	21.88688806	316.6554643	547.1061372	222.980585	90.07620961	796.2579566	13725.71631	9661.257966	18740.39837	229.3776163	443.1670831	14042.45755
LRRC31	0	0	0	0	0	0	0.825993731	9923.830857	9333.988558	23515.33586	0	0.412996865	14257.71843
BBS5	1.417000259	0	12.17905632	4.113579979	0	3.217007486	13.2158997	0	5909.255939	36890.38998	3.541927311	8.216453591	14266.54864
SNPH	0	0	0	0	0	0	0.825993731	11846.40063	10307.69788	21337.33687	0	0.412996865	14497.14512
CYB561	8.502001556	13.82329772	118.4690024	126.4925844	127.1931506	35.38708235	254.4060691	9483.832344	1085.253333	33182.56255	78.89600732	144.8965757	14583.88274
CGN	5.668001037	12.67135624	101.8611983	198.9944315	23.55428715	57.90613475	219.7143324	14080.69816	6567.107943	24095.40594	68.54985484	138.8102336	14914.40402
ZPBP2	0	0	0	0	0	0	0.825993731	15593.09983	23073.41356	6163.700668	0	0.412996865	14943.40469
REEP6	5.668001037	14.9752392	207.0439574	258.6413412	100.4982918	48.25511229	390.6950347	14479.67986	20056.93941	10437.00522	117.3653661	219.4750735	14991.20816
PROM2	0	5.759707383	139.5055542	74.55863712	78.5142905	54.68912726	177.5886522	6477.673016	17090.53121	22767.44676	59.66763784	116.1388897	15445.21699
GDF10	1.417000259	0	0	0	0	0	0.825993731	14603.47605	15315.03025	16530.17326	0.283400052	0.412996865	15482.89319

SPINT1	4.251000778	4.607765907	0	146.5462868	15.7028581	28.95306737	143.7229092	22106.56933	4296.469354	20217.32734	34.22158231	86.33798828	15540.12201
CAPSL	0	0	0	0	0	3.217007486	0.825993731	13667.54701	12194.00659	20964.00036	0	2.021500608	15608.51799
RAB25	0	1.151941477	25.46529957	28.79505986	0	96.51022458	14.86788716	12507.14416	14820.62888	20208.81478	11.08246018	55.68905587	15845.52927
WNT7B	0	0	0	0	0	3.217007486	0.825993731	11380.30051	1886.308711	35567.29511	0	2.021500608	16277.96811
OVOL2	0	0	0	0	0	0	0.825993731	16803.46862	13826.6723	18282.54431	0	0.412996865	16304.22841
LOC512548	0	0	25.46529957	0	248.105158	205.8884791	18.99785581	15518.52381	16909.41023	16528.95718	54.71409151	112.4431675	16318.96374
KCNG4	0	0	0	0	0	0	0.825993731	16400.01235	27004.69591	5736.248604	0	0.412996865	16380.31895
KMO	0	0	58.68090771	49.87715725	205.7074411	6.434014972	177.5886522	20524.81197	20418.4451	8862.181832	62.85310122	92.01133356	16601.81296
FOXQ1	1.417000259	0	0	0	0	3.217007486	0.825993731	10725.52306	2142.896762	37022.94268	0.283400052	2.021500608	16630.45417
GLB1L3	1.417000259	0	0	0	0	3.217007486	0.825993731	15520.76109	23183.85318	11600.18559	0.283400052	2.021500608	16768.26662
ETV5	77.93501427	129.0174454	581.2731424	390.790098	249.6754438	86.85920212	735.9604143	6740.180603	39013.53206	4622.319471	285.7382288	411.4098082	16792.01071
ADH1C	0	3.45582443	83.03902035	117.7512269	467.9451714	70.77416469	382.4350974	22087.92533	26244.13506	2116.586961	134.4382486	226.6046311	16816.21578
SDC1	41.09300752	38.01406873	226.9733223	823.7443909	50.24914592	144.7653369	892.8992232	21542.02887	15339.32697	13592.12437	236.0147871	518.83228	16824.4934
GCGR	4.251000778	8.063590336	115.1474415	148.0888793	10.99200067	86.85920212	186.6745832	12162.60295	20439.79676	18504.47888	57.30858252	136.7668927	17035.6262
CLDN8	0	2.303882953	26.57248651	31.36604734	12.56228648	0	56.16757371	11946.33249	3027.518119	36616.16397	14.56094066	28.08378685	17196.67153
OVOL1	1.417000259	0	0	0	0	0	0.825993731	15795.9466	10066.57137	26827.3293	0.283400052	0.412996865	17563.28242
CRHR1	0	0	0	0	0	0	0.825993731	14759.33993	3612.848105	34677.73271	0	0.412996865	17683.30692
BICDL2	0	1.151941477	0	78.6722171	3.14057162	99.72723207	45.4296552	24296.12125	5443.937007	23679.50664	16.59294604	72.57844363	17806.52163
TTC6	0	1.151941477	0	0	0	0	0.825993731	18183.12497	19554.071	16176.90207	0.230388295	0.412996865	17971.36601
SERPINB5	1.417000259	0	0	0	0	3.217007486	0.825993731	18711.12318	27629.78416	8055.920898	0.283400052	2.021500608	18132.27608
OSBPL3	7.085001297	18.43106363	60.89528159	202.0796165	89.50629117	80.42518715	278.3598873	19948.33934	2708.347617	34215.01433	75.59945083	179.3925372	18957.23376
NECTIN4	0	0	12.17905632	0	0	9.651022458	11.56391223	589.1505506	4268.123185	52295.68939	2.435811264	10.60746735	19050.98771
RUFY4	0	0	0	0	0	0	0.825993731	18846.10578	29360.373	8961.292339	0	0.412996865	19055.92371
PKP1	0	2.303882953	0	8.227159959	232.4022999	131.8973069	65.25350475	27869.05832	21292.39062	8046.192259	48.58666856	98.57540584	19069.21374
SCNN1A	0	3.45582443	0	61.18950219	84.79543374	32.17007486	134.6369781	30682.81152	18531.40012	9098.709361	29.88815207	83.4035265	19437.64033
PTPRF	42.51000778	27.64659544	285.65423	368.1654081	185.2937256	283.0966588	540.1999001	11294.53809	1098.506088	47104.85259	181.8539934	411.6482794	19832.63226
SLC17A2	0	0	0	0	0	0	0.825993731	13389.37846	4487.161765	41760.78974	0	0.412996865	19879.10999
RHOD	1.417000259	1.151941477	0	0	0	3.217007486	0.825993731	20768.67555	13603.9524	26372.51544	0.513788347	2.021500608	20248.38113
ENSBTAG00	1.417000259	1.151941477	0	0	0	3.217007486	0.825993731	15333.57528	3499.095297	44406.97947	0.513788347	2.021500608	21079.88335
FRMPD1	1.417000259	0	0	0	0	3.217007486	0.825993731	17690.17748	15940.1185	31883.78928	0.283400052	2.021500608	21838.02842
SLC39A8	15.58700285	23.03882953	228.0805092	78.15801961	2506.176153	93.29321709	960.6307091	29910.94973	30546.12639	5514.314033	570.2081028	526.9619631	21990.46338
ENSBTAG00	4.251000778	8.063590336	84.14620729	168.1425817	73.80343307	12.86802994	247.7981193	26993.53586	29957.48322	9099.92544	67.68136263	130.3330746	22016.98151
RBBP8NL	0	0	0	0	0	3.217007486	0.825993731	21984.26466	24253.64497	20500.67394	0	2.021500608	22246.19452
CLCA2	1.417000259	0	0	0	0	3.217007486	0.825993731	22856.05832	39515.6642	7367.011669	0.283400052	2.021500608	23246.24473
IRX4	0	0	0	0	0	3.217007486	0.825993731	22179.65383	12111.17687	37338.5154	0	2.021500608	23876.4487
SPDEF	0	2.303882953	0	9.255554953	127.1931506	12.86802994	69.3834734	35328.15175	11604.25902	25540.10879	27.7505177	41.12575167	24157.50652
CRLF1	0	0	0	0	0	3.217007486	0.825993731	18344.20917	28283.58671	25845.34483	0	2.021500608	24157.71357
FOXA1	0	1.151941477	42.07310364	39.0790098	0	3.217007486	71.03546086	9875.356444	24090.56246	38815.44436	16.46081098	37.12623417	24260.45442
CES3	0	0	83.03902035	1.542592492	331.3303059	22.5190524	124.7250534	13653.37757	20773.32441	39067.17289	83.18238375	73.62205289	24497.95829
ARHGAP1	0	0	18.82217795	0	0	0	19.82384954	36.54224934	50560.36248	25718.87253	3.764435589	9.911924772	25438.59242
HSD17B2	0	3.45582443	25.46529957	77.64382211	50.24914592	122.2462845	109.8571662	35621.23551	32045.16017	8943.051141	31.36281841	116.0517253	25536.48227
GALNT8	0	2.303882953	77.50308566	19.0253074	86.36571955	9.651022458	96.64126652	10816.5058	31645.73687	35421.36553	37.03959911	53.14614449	25961.20274
GJB1	0	1.151941477	45.39466446	55.01913222	0	12.86802994	85.90334802	16049.50506	28482.00989	34463.09462	20.31314763	49.38568898	26331.53652
CYP4F21	0	0	0	19.0253074	133.4742939	0	87.55533548	35487.74443	14235.66703	30481.64922	30.49992025	43.77766774	26735.02023
KCNK1	1.417000259	0	55.3593469	0	257.5268728	6.434014972	111.5091537	16749.77388	18889.22449	47381.51075	62.860644	58.97158433	27673.50304
G31754	0	0	0	0	0	0	0.825993731	29218.13851	36398.32186	17549.24817	0	0.412996865	27721.90284
ELMO3	0	5.759707383	50.93059915	7.712962461	694.066328	286.3136663	151.1568528	29048.10519	4177.930828	50546.35854	151.6939194	218.7352595	27924.13152
ERBB3	76.51801401	40.31795168	540.3072257	336.2851633	546.4594619	141.5483294	830.9496933	13918.12244	3879.00759	70516.21362	307.9775633	486.2490114	29437.78122
MAP6	1.417000259	0	0	0	103.6388635	0	37.99571162	27197.12839	2480.105735	60334.58517	21.01117274	18.99785581	30003.93976
SFN	2.834000519	1.151941477	74.18152484	131.1203618	12.56228648	315.2667336	56.99356744	27144.92518	15100.40926	49251.23351	44.37002303	186.1301505	30498.85598
NUPR1	90.6880166	89.85143518	496.0197482	817.5740209	635.9657531	514.7211978	1286.072239	37208.21319	42030.37435	13154.94367	426.0197948	900.3967184	30797.84374
ENTPD3	2.834000519	3.45582443	0	41.13579979	136.6148655	57.90613475	110.6831599	33037.17644	2736.693786	59057.70134	36.80809804	84.29464735	31610.52386
MSMB	1.417000259	1.151941477	0	0	0	3.217007486	0.825993731	13097.78623	50498.14816	34312.30072	0.513788347	2.021500608	32636.07837
S100A2	26.92300493	6.91164886	159.4349191	135.2339418	403.5634532	958.6682308	41.29968655	23959.7834	2152.836328	73207.39831	146.4133936	499.9839587	33106.67268
ZNF185	0	0	0	0	0	0	0.825993731	33661.37772	32328.98999	33473.20563	0	0.412996865	33154.52445
CFAP52	1.417000259	1.151941477	0	0	0	0	0.825993731	10806.06516	0	89152.63717	0.513788347	0.412996865	33319.56744
ADAMTSL2	99.19001816	52.98930792	415.1951017	73.53024213	2912.880178	540.4572576	1054.793994	36258.11471	10799.52232	55358.38646	710.7569695	797.625626	34138.6745

ENSMMSG00	0	0	0	0	0	3.217007486	0.825993731	26851.84142	5255.82152	71790.6653	0	2.021500608	34632.77608
MAL2	0	0	0	43.70678728	67.52228983	19.30204492	90.03331668	53171.21007	46677.30543	5795.228476	22.24581542	54.6676808	35214.58133
PGRMC1	134.6150246	110.5863818	853.6411292	1391.418428	199.4262979	90.07620961	1620.5997	34966.45806	37958.83369	34237.51181	537.9374523	855.3379549	35720.93452
TMC3	0	0	0	0	0	0	0.825993731	7466.551029	4620.793705	99134.82853	0	0.412996865	37074.05776
GGT6	0	1.151941477	0	0	108.3497209	25.73605989	32.21375551	52552.22911	45574.38176	14492.02345	21.90033247	28.9749077	37539.54477
GCLC	128.9470236	110.5863818	753.9943048	804.7190834	1549.872095	119.029277	1882.439713	42917.75321	59486.82884	11302.85407	669.6237776	1000.734495	37902.47871
SNX31	0	0	59.78809465	37.53641731	72.23314726	25.73605989	100.7712352	24482.5613	29905.20846	59725.93721	33.91153184	63.25364753	38037.90232
CACNA1H	0	0	0	0	0	0	0.825993731	25039.64416	3128.386305	91066.74684	0	0.412996865	39744.92577
SLC15A1	1.417000259	0	0	0	0	3.217007486	0.825993731	29814.74666	9990.368034	82931.17271	0.283400052	2.021500608	40912.0958
GABRR2	0	3.45582443	0	46.27777477	172.7314391	6.434014972	140.4189343	61920.46863	40288.00528	22877.50198	44.49300766	73.42647462	41695.3253
EPCAM	2.834000519	3.45582443	38.75154283	60.67530469	108.3497209	38.60408983	161.0687775	57501.09374	57771.70154	13861.48605	42.81327867	99.83643368	43044.76044
WWC1	0	6.91164886	35.42998202	57.07592221	72.23314726	32.17007486	132.158997	35991.87832	3101.512664	90163.80756	34.33014007	82.16453591	43085.73285
KRT18	18.42100337	42.62183464	137.2911803	147.0604843	2404.107575	1180.641747	655.8390224	65955.77702	34192.10638	29435.82056	549.9004155	918.2403849	43194.56799
ACER1	0	0	0	0	0	3.217007486	0.825993731	41952.73952	18215.54281	74757.29207	0	2.021500608	44975.19147
SPINT2	230.9710423	146.2965675	854.7483161	884.4196955	714.4800436	1122.735613	1399.23338	28969.80037	5113.722543	101463.0134	566.183133	1260.984496	45182.17876
BCAT2	86.43701582	116.3460891	983.1820009	1618.693722	340.7520208	289.5306737	2094.720102	47085.80692	62210.26987	27924.84136	629.0821697	1192.125388	45740.30605
AKR1C4	0	0	0	0	0	0	0.825993731	18793.1568	52017.4292	66609.55714	0	0.412996865	45806.71438
AKR1A1	100.6070184	76.02813746	752.8871178	1608.409772	182.153154	1466.955414	1524.784427	51724.4353	36674.42091	57841.6215	544.0170399	1495.86992	48746.8259
ILDR1	0	0	0	0	0	3.217007486	0.825993731	43608.32715	52463.60526	50436.30331	0	2.021500608	48836.07857
GGT5	49.59500908	51.83736645	591.2378249	716.7913114	565.3028916	765.6477817	1150.609267	39417.15488	9736.356908	100432.3857	394.9528807	958.1285244	49861.96583
TRPV6	0	2.303882953	71.96715097	0	381.5794518	154.4163593	121.4210785	32931.2785	47258.21784	75649.89467	91.17009715	137.9187189	51946.46367
OCRL	1.417000259	0	0	0	0	0	0.825993731	52174.1287	83892.51235	19819.06119	0.283400052	0.412996865	51961.90074
GLYATL2	0	5.759707383	39.85872977	34.45123233	73.80343307	9.651022458	112.3351474	64855.03498	44500.17239	46611.12418	30.77462051	60.99308493	51988.77718
TJP3	15.58700285	29.95047839	302.2620341	84.84258707	1342.594368	395.6919208	646.7530913	53683.54732	21388.47309	84378.30772	355.047294	521.2225061	53150.10938
KRT5	0	0	224.7589484	14.39752993	577.8651781	302.3987037	215.5843638	19818.57707	89292.64164	54493.14566	163.4043313	258.9915337	54534.78812
KDF1	0	0	0	0	0	3.217007486	0.825993731	39310.51117	7138.080779	117429.5336	0	2.021500608	54626.04186
IQANK1	1.417000259	1.151941477	0	0	0	3.217007486	0.825993731	39775.86553	8386.048486	117677.6139	0.513788347	2.021500608	55279.84265
KRT8	11.33600207	29.95047839	178.257097	43.70678728	3139.001334	299.1816962	948.2408031	87962.41448	55217.96939	35162.34053	680.4503398	623.7112497	59447.5748
SAT1	799.1881463	934.2245375	926.7154671	4318.744781	573.1543207	1814.392222	4042.413319	76359.87744	20804.2475	82948.80587	1510.40545	2928.402771	60037.6436
TRPV4	0	1.151941477	0	0	0	3.217007486	0.825993731	53055.61724	37672.79508	93024.63538	0.230388295	2.021500608	61251.0159
TFAP2A	0	0	0	42.16419479	135.0445797	22.5190524	138.7669468	87819.97429	93717.58909	10241.21637	35.44175489	80.6429996	63926.25991
B4GALNT4	0	0	0	0	0	0	0.825993731	24126.08793	35594.68956	133132.7726	0	0.412996865	64284.5167
MGC151921	0	187.7664607	281.2254822	131.6345593	431.8285978	115.8122695	373.3491664	23044.73565	2056.017594	168144.9273	206.49102	244.5807179	64415.22684
LOC538060	93.52201712	20.73494658	282.3326692	352.2252857	836.9623367	173.7184042	783.042057	76465.02963	112229.1101	8037.679701	317.1554511	478.3802306	65577.27313
IL17RE	5.668001037	16.12718067	297.8332863	312.1178809	246.5348722	176.9354117	556.7197747	48911.42786	21027.33553	127986.9308	175.6562442	366.8275932	65975.23139
RDH16	0	5.759707383	76.39589872	154.7734467	133.4742939	22.5190524	281.6638623	58433.29398	5101.206052	138107.1472	74.08066934	152.0914573	67213.88241
ACSM3	0	4.607765907	209.2583313	418.5567629	92.64686279	115.8122695	536.0699314	76312.89455	71962.82459	57816.08382	145.0139446	325.9411004	68697.26765
LRRC26	0	0	0	124.4357944	45.53828849	102.9442396	142.0709217	77463.60252	5509.832647	125370.535	33.99481657	122.5075806	69447.99005
MARVELD3	0	0	0	0	0	3.217007486	0.825993731	54628.42548	12795.90252	142060.0147	0	2.021500608	69828.11424
MFSD4A	8.502001556	13.82329772	204.8295835	169.6851741	100.4982918	54.68912726	310.5736428	49085.18999	126720.997	38269.42452	99.46766976	182.6313851	71358.53718
STRA6	0	3.45582443	257.9745565	259.6697362	307.7760188	67.55715721	548.4598374	59135.05432	24260.27135	131018.0098	165.7752272	308.0084973	71471.11181
LSS	60.93101115	61.05289826	662.0977889	536.3079898	529.186318	80.42518715	1096.919675	37331.26362	7748.443746	173828.8844	369.9152012	588.6724309	72969.53061
ACY1	590.8891082	345.582443	1863.395617	2789.521423	727.04233	1910.902447	3298.192968	75392.62647	94792.53472	50036.82108	1263.286184	2604.547707	73407.32743
KRT14	1.417000259	1.151941477	78.6102726	0	240.2537289	341.0027935	16.51987462	38040.48156	87344.48674	110798.2503	64.28658865	178.7613341	78727.73953
ST14	8.502001556	5.759707383	45.39466446	121.8648069	86.36571955	90.07620961	207.3244265	74416.42638	7127.036817	155028.8982	53.57737997	148.700318	78857.45379
ATP1A1	520.0390952	582.8823872	2079.29707	5066.387942	510.3428883	1901.251424	5292.967828	91017.79399	59708.07621	91746.53547	1751.789876	3597.109626	80824.13522
TFAP2C	0	0	0	0	0	0	0.825993731	89511.3584	134956.1114	31154.14137	0	0.412996865	85207.20371
LOC507550	0	4.607765907	0	239.1018363	120.9120074	51.47211978	352.6993231	154653.5111	76910.15144	54133.79406	72.92432192	202.0857214	95232.48552
DSP	162.9550298	195.830051	2327.306944	2158.086897	486.7886011	1701.79696	2619.226121	42265.21304	163255.5277	87264.67322	1066.193504	2160.51154	97595.138
KRT17	0	1.151941477	157.2205452	0	340.7520208	357.0878309	84.25136056	16757.23148	103471.9845	178902.3695	99.82490149	220.6695958	99710.5285
MBOAT2	17.00400311	25.34271249	376.4435589	628.3493418	380.009166	427.8619956	941.6328533	115964.9639	160286.9108	24665.13935	285.4297565	684.7474245	100305.6713
AOX4	0	0	0	0	0	0	0.825993731	104115.5802	162864.5715	34235.07965	0	0.412996865	100405.0771
ENSMMSG00	0	0	0	0	0	0	0.825993731	99610.4429	71026.29661	143639.7024	0	0.412996865	104758.814
ENSMMSG00	0	0	0	0	0	3.217007486	0.825993731	86030.89559	25406.6346	208139.361	0	2.021500608	106525.6304
AZGP1	0	4.607765907	115.1474415	189.7388765	83.22514793	141.5483294	279.1858811	149220.6481	86375.19501	153821.3309	78.54384639	210.3671052	129805.7247
LRP2	14.17000259	0	214.794266	526.5382373	452.2423133	93.29321709	878.8573297	184797.8837	148794.5639	69797.51044	241.5489638	486.0752734	134463.3193
GGT1	4.251000778	5.759707383	168.2924146	87.92777206	281.08116	131.8973069	295.7057557	82611.58512	14520.96938	327409.4315	109.462411	213.8015313	141513.9953

ABCC11	0	12.67135624	139.5055542	391.818493	544.8891761	221.9735165	761.5662199	213649.1082	127272.4589	101511.6566	217.7769159	491.7698682	147477.7412
LPO	0	5.759707383	83.03902035	322.4018309	202.5668695	25.73605989	521.2020442	223498.363	51301.78046	252010.6573	122.7534856	273.4690521	175603.6003
LGALS7	0	1.151941477	0	0	0	3.217007486	0.825993731	145283.78	346823.1104	53202.88495	0.230388295	2.021500608	181769.9251
AAK1	0	1.151941477	0	0	0	0	0.825993731	195028.222	260223.723	109041.6229	0.230388295	0.412996865	188097.856
GRIN1	5.668001037	3.45582443	105.1827591	159.4012242	310.9165904	77.20817966	392.3470222	161129.6925	9117.526903	421929.2371	116.9248798	234.7776009	197392.1522
KRT19	9.919001816	21.88688806	344.3351377	374.8499756	923.3280563	540.4572576	956.5007405	254943.3415	270353.2449	79391.77234	334.8638119	748.4789991	201562.7863
ENSMMSG0	0	8.063590336	236.9380047	409.3012079	742.7451881	51.47211978	893.7252169	274798.4608	224681.3082	108595.9297	279.4095982	472.5986683	202691.8996
ENSMMSG0	0	0	0	118.2654244	519.7646031	0	261.8400127	274356.225	473.7859702	413346.7536	127.6060055	130.9200064	229392.2549
FAM83F	0	0	0	0	0	3.217007486	0.825993731	174625.7147	43882.07865	473241.7259	0	2.021500608	230583.1731
NUMA1	0	6.91164886	36.53716895	0	0	70.77416469	16.51987462	43.99985125	59880.36202	737830.297	8.689763563	43.64701966	265918.2196
SAO	0	0	0	0	0	0	0.825993731	299392.8861	518791.9561	96166.37764	0	0.412996865	304783.7399
BSPRY	0	0	0	0	0	3.217007486	0.825993731	283031.6533	89365.16366	692278.9864	0	2.021500608	354891.9345
TMPRSS2	0	1.151941477	0	0	0	0	0.825993731	362572.198	199032.4427	518836.2073	0.230388295	0.412996865	360146.9493
ENSMMSG0	0	13.82329772	355.4070071	1090.612892	111.4902925	64.34014972	1120.873493	517945.3676	210446.7455	568007.7876	314.2666979	592.6068213	432133.3002
KRT7	0	28.79853692	525.9137955	1762.669021	255.956587	1158.122695	1727.978885	595282.1909	386427.4945	324366.1917	514.6675881	1443.05079	435358.6257
MARK3	0	0	0	0	0	0	0.825993731	553109.4521	481571.9634	712579.6152	0	0.412996865	582420.3436
MAPK13	0	1.151941477	0	0	0	3.217007486	0.825993731	693356.3678	126971.3268	2133268.536	0.230388295	2.021500608	984532.0769
G6PD	0	0	0	0	0	0	0.825993731	1384679.793	1125172.839	1524990.265	0	0.412996865	1344947.632
FASN	1072.669196	1025.227914	26375.40724	46505.05006	6474.288395	3715.643646	56340.2064	4668149.303	2555236.336	8641140.472	16290.52856	30027.92502	5288175.37
