# Supplementary material for: Integrated multi-omics analysis reveals insights into Chinese forest musk deer (Moschus berezovskii) genome evolution and musk synthesis
Source: Front Cell Dev Biol. 2023 May 9;11:1156138. doi: 10.3389/fcell.2023.1156138 (PMC10203155; doi:10.3389/fcell.2023.1156138)
Supplement: Supplementary file 1 [file DataSheet1.zip › Data Sheet 1/Table S6_The KEGG analysis of upregulating DEG_RE_C.pdf]

Table S6. The KEGG analysis of upregulating DEGs in adult musk gland compared to the juvenile

KEGG ID	Description	Enrichment Fold	*P*-value
bta00350	Tyrosine metabolism	6.65	7.02E-05
bta00062	Fatty acid elongation	6.39	9.58E-04
bta01040	Biosynthesis of unsaturated fatty acids	6.18	1.13E-03
bta05033	Nicotine addiction	5.56	6.44E-04
bta00260	Glycine, serine and threonine metabolism	4.94	1.22E-03
bta00480	Glutathione metabolism	4.78	2.40E-04
bta00982	Drug metabolism - cytochrome P450	4.19	1.33E-03
bta05150	Staphylococcus aureus infection	3.46	6.02E-04
bta04530	Tight junction	3.33	7.02E-05
bta04514	Cell adhesion molecules	3.25	9.58E-04
