# Supplementary material for: Integrated multi-omics analysis reveals insights into Chinese forest musk deer (Moschus berezovskii) genome evolution and musk synthesis
Source: Front Cell Dev Biol. 2023 May 9;11:1156138. doi: 10.3389/fcell.2023.1156138 (PMC10203155; doi:10.3389/fcell.2023.1156138)
Supplement: Supplementary file 1 [file DataSheet1.zip › Data Sheet 1/Table S7_2023_The comparison of the upregulated DEGs_RE_C_2.pdf]

	J_muscle_1	J_muscle_2	A_muscle_3	A_muscle_1	A_muscle_2	J_musk_glanc	J_musk_glanc	A_musk_glan	A_musk_glan	A_musk_glan	Avg_muscle	Avg_J_musk_	Avg_A_musk_
KRT81	0	0	0	0	0	0	63.60151728	0	0	0	0	31.80075864	0
LIPK	0	0	0	0	0	0	63.60151728	0	0	12.76883832	0	31.80075864	4.256279439
ENSMMSG00	0	0	0	0	0	141.5483294	62.77552355	0	0	0	0	102.1619265	0
PGLYRP3	0	0	0	0	0	199.4544641	4.955962386	0	0	0	0	102.2052133	0
LIPF	0	0	0	0	0	221.9735165	4.129968655	0	0	0	0	113.0517426	0
SCEL	0	0	0	0	0	193.0204492	58.6455549	0	0	0	0	125.833002	0
LOC524771	0	0	0	0	0	234.8415465	18.99785581	0	0	0	0	126.9197011	0
TM4SF5	0	0	0	0	0	257.3605989	6.607949848	0	0	0	0	131.9842744	0
KRT85	0	0	0	0	0	12.86802994	253.5800754	0	0	0	0	133.2240527	0
MAB21L4	0	0	0	0	0	337.785786	4.955962386	0	0	0	0	171.3708742	0
KRT33B	0	0	0	0	0	0	391.5210285	0	0	0	0	195.7605142	0
EREG	0	0	0	0	0	408.5599507	4.955962386	0	1.472528268	0	0	206.7579566	0.490842756
MGC157368	0	0	0	0	0	453.5980555	0	0	0	0	0	226.7990278	0
SLC5A9	0	0	0	0	0	460.0320705	3.303974924	0	0	2.432159679	0	231.6680227	0.810719893
ERG28	35.42500648	32.25436135	93.00370279	105.410487	136.6148655	411.7769582	85.90334802	201.3552515	171.9176753	148.3617404	80.54168461	248.8401531	173.8782224
ENSBTAG000	0	0	0	0	0	495.4191528	17.34586835	0	0	0	0	256.3825106	0
KRT36	5.668001037	0	0	0	0	546.8912726	11.56391223	0	0	0	1.133600207	279.2275924	0
KRT9	0	0	0	0	0	595.1463849	8.25993731	0	0	1.82411976	0	301.7031611	0.60803992
L3MBTL1	0	0	0	0	0	694.873617	0	0	0	0	0	347.4368085	0
ENSMMSG00	0	0	0	0	0	0	718.6145459	0	0	0	0	359.307273	0
IMMT	0	0	0	0	0	0	838.3836369	0	0	0	0	419.1918185	0
AVP	0	0	0	0	0	846.0729688	0	0	0	0	0	423.0364844	0
PLA2G4F	0	0	0	0	0	865.3750137	52.03760505	0	0	6.080399199	0	458.7063094	2.026799733
GSDMC	0	0	0	0	0	936.1491784	0	0	0	0	0	468.0745892	0
IL36G	0	0	0	0	0	939.3661859	0	0	0	0	0	469.683093	0
G984	0	0	0	0	0	958.6682308	31.38776178	0	0	0	0	495.0279963	0
ENSBTAG000	0	0	0	0	0	1167.773717	0	0	0	0	0	583.8868587	0
VPS36	0	0	0	1.542592492	0	0	1181.171035	0	1.104396201	0	0.308518498	590.5855176	0.368132067
IL1F10	0	0	0	0	0	1290.020002	17.34586835	0	0	0	0	653.6829351	0
G29879	0	0	0	0	0	1341.492122	18.99785581	0	0	0	0	680.2449887	0
DMKN	0	1.151941477	156.1133583	0	0	1389.747234	0.825993731	0	22.82418815	24.92963671	31.45305995	695.2866138	15.91794162
SLC35F3	0	0	0	0	0	1605.286736	0	0	0	0	0	802.6433678	0
RBP2	0	0	0	0	0	1717.881998	0	0	0	0	0	858.9409988	0
G24434	0	0	0	0	0	2017.063694	47.9076364	0	0	3.648239519	0	1032.485665	1.21607984
SAA4	0	3.45582443	0	0	0	2261.556263	0	0	0	0	0.691164886	1130.778131	0
LOC10029610	0	0	0	0	0	2300.160352	6.607949848	0	0	0	0	1153.384151	0
KRT25	0	0	0	0	0	0	2562.232553	0	0	0	0	1281.116277	0
LOC789175	0	0	0	0	0	2570.388981	9.085931041	0	0	0	0	1289.737456	0
CDSN	0	0	0	0	0	2415.972622	312.2256303	0	0	6.080399199	0	1364.099126	2.026799733
NRDC	0	0	0	0	0	2847.051625	0	0	0	0	0	1423.525813	0
CSTB	0	0	0	4.113579979	0	2904.95776	27.25779312	0	0.736264134	0	0.822715996	1466.107776	0.245421378
LOC519132	0	0	0	0	0	3200.922449	0	0	0	0	0	1600.461224	0
KRT74/KRT7	0	4.607765907	0	0	0	0	3365.924454	0	0	0	0.921553181	1682.962227	0
LYPD2	21.25500389	0	0	2.05678999	0	4912.370431	0	0	5.521981005	0	4.662358776	2456.185216	1.840660335
SERPINA12	0	0	0	0	0	5211.552127	8.25993731	0	7.36264134	0	0	2609.906032	2.45421378
ACP7	0	0	0	8.227159959	0	5967.548887	39.64769909	0	14.35715061	24.92963671	1.645431992	3003.598293	13.09559578
WNT3	0	0	0	0	0	7289.738963	2.477981193	0	0	1.82411976	0	3646.108472	0.60803992
TGM3	0	0	0	0	0	7421.63627	62.77552355	0	0	2.432159679	0	3742.205897	0.810719893
WFDC5	0	0	0	0	0	7836.630236	0	0	0	0	0	3918.315118	0
CFAP57	0	0	0	0	0	7875.234326	0	0	2.945056536	0	0	3937.617163	0.981685512
SLPI	0	16.12718067	0	18.51110991	25.12457296	8508.9848	49.55962386	0	2.945056536	2.432159679	11.95257271	4279.272212	1.792405405
SLC36A2	0	0	0	0	4.71085743	9795.787795	0	0	0	0	0.942171486	4897.893897	0
PDZK1IP1	0	11.51941477	0	0	0	12144.20326	159.4167901	0	4.785716871	57.15575247	2.303882953	6151.810025	20.64715645
CDK5RAP2	1663.558305	0	0	0	0	12777.95373	0	0	0	0	332.7116609	6388.976867	0
IL36RN	0	0	0	0	0	12768.30271	23.9538182	0	2.945056536	0	0	6396.128265	0.981685512

SPINK7	0	0	0	0	0	16924.67638	0	0	0	0	0	8462.338192	0
ALOX12B	0	0	0	0	0	17404.0105	104.0752101	0	1.104396201	4.864319359	0	8754.042855	1.989571853
CASP14	0	0	0	0	0	20514.85674	5.781956117	0	0	0	0	10260.31935	0
KRT1	0	8.063590336	0	0	4.71085743	37700.11073	186.6745832	0	0	9.120598798	2.554889553	18943.39266	3.040199599
FABP9	0	0	0	8.741357456	0	47766.12715	346.0913733	0	0	0	1.748271491	24056.10926	0
CATHL4	563.9661032	3.45582443	0	0	0	72102.78878	1.651987462	0	0	0	113.4843855	36052.22039	0
ENSMMSG00	0	8.063590336	0	0	0	91224.68128	26.43179939	0	0	0	1.612718067	45625.55654	0
LY6D	0	2.303882953	0	0	0	106695.2703	6.607949848	0	0	3.040199599	0.460776591	53350.93912	1.013399866
G978	0	12.67135624	0	0	0	120177.7487	246.1461318	0	0	2.432159679	2.534271249	60211.94739	0.810719893
G993	0	8.063590336	0	0	0	134692.8864	122.2470722	0	0	0	1.612718067	67407.56675	0
SULT2B1	0	4.607765907	0	0	0	135574.3465	119.769091	0	0	13.98491816	0.921553181	67847.05779	4.661639386
SBSN	0	29.95047839	0	0	0	193281.0268	497.248226	0	0	17.63315768	5.990095678	96889.1375	5.877719225
KRTDAP	0	21.88688806	0	0	0	256852.3117	121.4210785	0	0	0	4.377377611	128486.8664	0
CALML5	0	26.49465396	0	0	0	516802.6016	159.4167901	0	0	17.63315768	5.298930792	258481.0092	5.877719225
LOC786350	0	245.3635345	0	0	0	2768556.642	90.85931041	0	0	0	49.0727069	1384323.751	0

gland
