# Supplementary figures and images for: Integrated multi-omics analysis reveals insights into Chinese forest musk deer (Moschus berezovskii) genome evolution and musk synthesis
Source: Front Cell Dev Biol. 2023 May 9;11:1156138. doi: 10.3389/fcell.2023.1156138 (PMC10203155; doi:10.3389/fcell.2023.1156138)

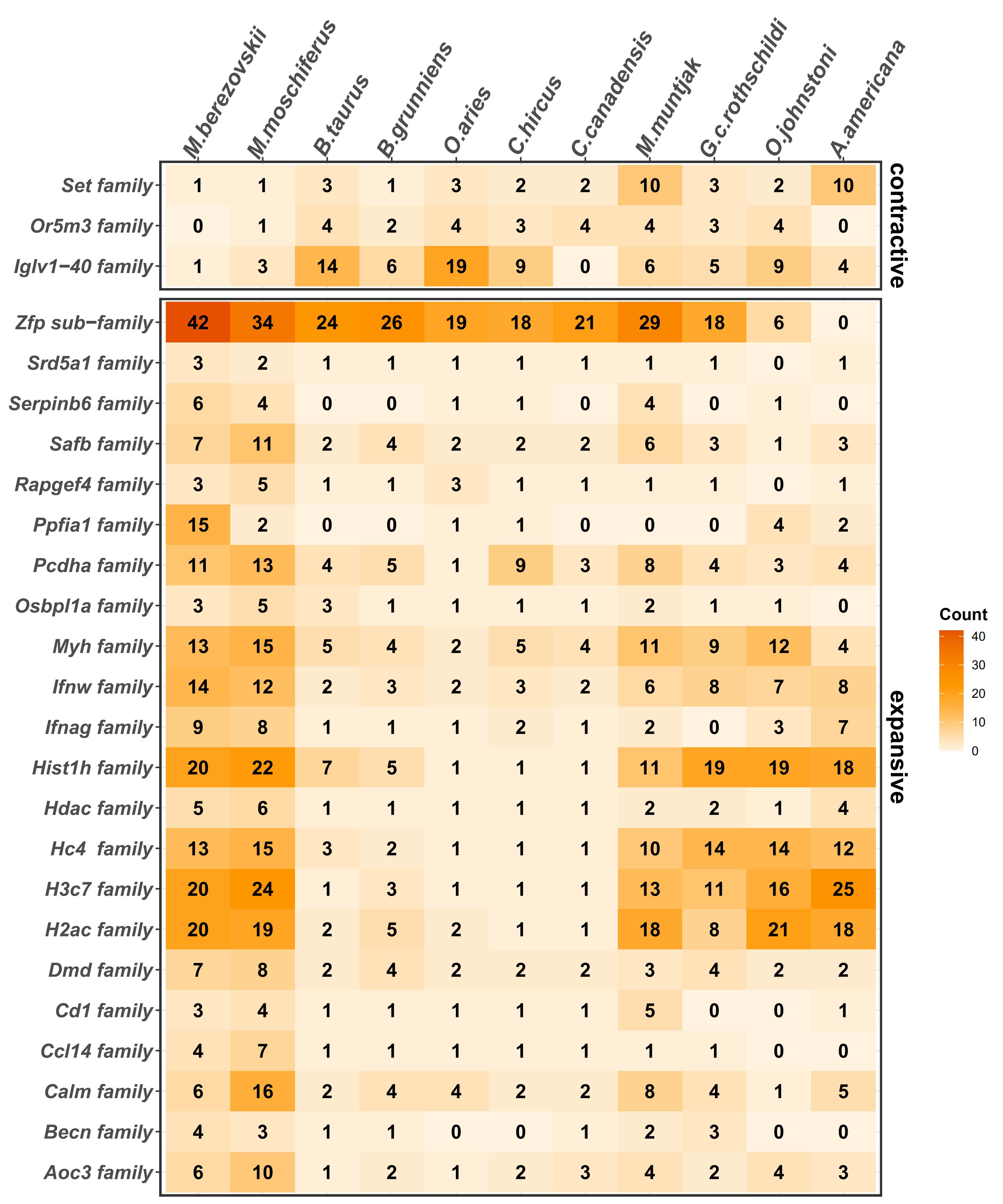

Supplement: Supplementary file 1 [file DataSheet1.zip › Data Sheet 1/Figure S1_2023_RE_C.jpg]

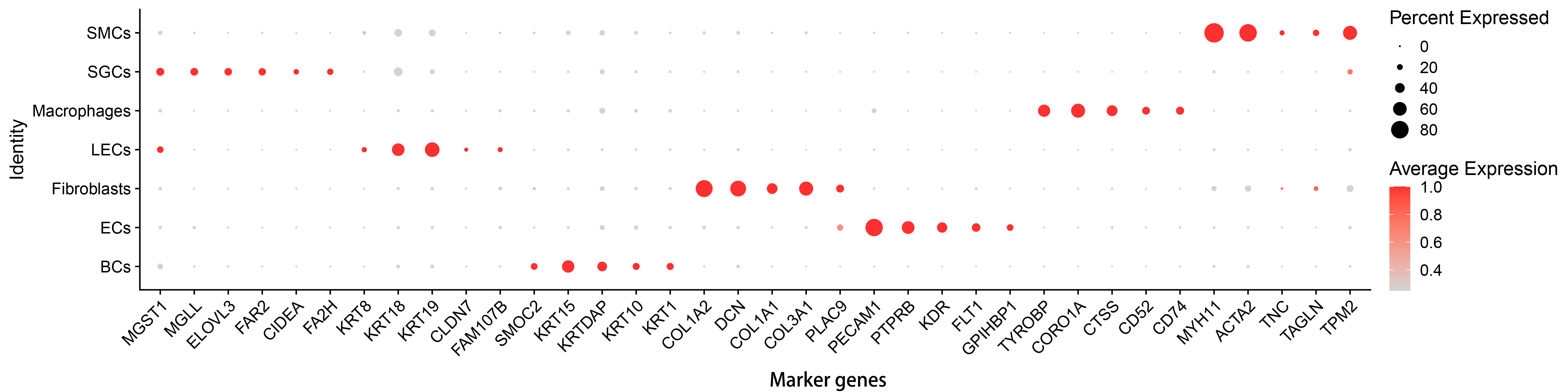

Supplement: Supplementary file 1 [file DataSheet1.zip › Data Sheet 1/Figure S2_cell_type_markers_RE_C.jpg]
